# Enterovirus D68 2A protease causes nuclear pore complex dysfunction and independently contributes to motor neuron toxicity

**DOI:** 10.7554/eLife.108672

**Published:** 2026-06-18

**Authors:** Katrina M Zinn, Mathew W McLaren, Michael T Imai, Malavika M Jayaram, Jeffery D Rothstein, Matthew J Elrick

**Affiliations:** 1 Hugo W. Moser Research Institute, Michael V. Johnston Center for Developmental Neuroscience, Kennedy Krieger Institute Baltimore United States; 2 Department of Neurology, Johns Hopkins School of Medicine Baltimore United States; The University of Texas Southwestern Medical Center United States; The University of Texas Southwestern Medical Center United States

**Keywords:** enterovirus, acute flaccid myelitis, nuclear pore complex, virus-host interaction, Human, Viruses

## Abstract

Enterovirus D68 (EV-D68) is an important pathogen associated with acute flaccid myelitis (AFM). The pathogenesis of AFM involves infection of spinal motor neurons and motor neuron death; however, the mechanisms linking EV-D68 infection to selective neurotoxicity are not well understood. Dysfunction of the nuclear pore complex (NPC) has been implicated in motor neuron injury in neurodegenerative diseases such as amyotrophic lateral sclerosis, and the NPC is also modified by picornavirus proteases during infection. We therefore sought to determine the impact of EV-D68 proteases on NPC composition and function. We demonstrate widespread disruption of NPC composition by EV-D68 2A and 3C proteases via direct cleavage of a relatively small number of nucleoporins, notably Nup98 and POM121, by 2A^pro^. Using reporter systems, we demonstrate that 2A^pro^ inhibits nuclear transport of protein cargoes and disrupts the permeability barrier of the NPC, while having no apparent effect on RNA export. Independently, we show 2A^pro^ is toxic to induced pluripotent stem cell-derived motor neurons by demonstrating a rescue of toxicity with the 2A^pro^ inhibitor telaprevir at concentrations insufficient to inhibit viral replication. These findings expand our understanding of EV-D68 neuropathogenesis and provide a rationale for studying the NPC or 2A^pro^ as therapeutic targets in AFM.

## Introduction

Enterovirus D68 (EV-D68) has caused multiple outbreaks of severe respiratory disease and the polio-like disorder acute flaccid myelitis (AFM) during the past decade. Though much work has been done to demonstrate the association between EV-D68 and AFM and to characterize the molecular evolution of EV-D68 leading to the increased virulence of contemporary strains, the mechanisms underlying its neuropathogenesis remain poorly understood (Elrick et al., 2021). EV-D68 has been shown to infect spinal motor neurons in mouse models (Hixon et al., 2017) and human autopsy tissue (Vogt et al., 2022), and the dysfunction or death of motor neurons is thought to be the proximate cause of paralysis in AFM.

EV-D68 is a member of the family Picornaviridae, which have a non-enveloped icosahedral capsid and a single-stranded positive-sense RNA genome (Oberste et al., 2004). Picornaviruses alter the composition and function of the nuclear pore complex (NPC) during the course of their life cycle (reviewed in Lizcano-Perret and Michiels, 2021). The purpose of this is felt to be twofold. First, it allows for the redistribution of nuclear proteins such as Sam68 (McBride et al., 1996), polypyrimidine tract binding protein (Back et al., 2002), and the La protein (Meerovitch et al., 1993) to the cytoplasm, where they participate in the translation or replication of viral RNA. Second, it may disrupt nuclear translocation of transcription factors that mediate innate immunity, including interferon response factor 3 (Delhaye et al., 2004) and nuclear factor κB (Watters et al., 2017).

The NPC is a macromolecular structure greater than 100 MDa in size constructed in eightfold symmetry from subunits known as nucleoporins (Nups). The NPC includes a symmetric core that is comprised of transmembrane Nups that anchor the NPC to the nuclear envelope, inner and outer rings, and a central channel. The central channel consists primarily of phenylalanine-glycine (FG) repeat domains contributed by multiple Nups that confer an intrinsically disordered structure. The central channel creates a permeability barrier against passive diffusion of macromolecules 40 kDa and larger. The central channel also permits active nucleocytoplasmic transport (NCT). NCT is mediated by transport adapters known as importins and exportins, which interact with FG-repeats and require a Ran-GTP/Ran-GDP gradient across the nuclear envelope. On the nuclear face of the NPC resides the nuclear basket, which plays roles in chromatin organization and RNA quality control, while the cytoplasmic face is adorned with cytoplasmic filaments that are important for transport receptor docking and remodeling of ribonucleoproteins during export (reviewed in Lin and Hoelz, 2019).

Disruptions of nuclear import by picornaviruses were first reported in cells infected with poliovirus (PV) and coxsackievirus B3 (CV-B3) (Belov et al., 2000). These deficits were later correlated to cleavage of Nup153 and Nup62 during infection with PV (Gustin and Sarnow, 2001) or human rhinovirus 14 (RV14) (Gustin and Sarnow, 2002), implicating viral proteases in disruption of the NPC. PV 2A protease (2A^pro^) was shown to disrupt the permeability barrier of the NPC, allowing passive diffusion across the nuclear lamina (Belov et al., 2004). RV 3C and 3CD proteases were found to impair nuclear import (Ghildyal et al., 2009). There have been conflicting reports as to whether or not PV 2A^pro^ activity also impairs RNA export (Park et al., 2008; Castelló et al., 2009). The list of Nups targeted by enterovirus proteases was expanded to include Nup98 by PV 2A^pro^ (Park et al., 2008) and RanBP2, Nup214, and Nup153 by RV 3C^pro^ and 3CD^pro^ (Ghildyal et al., 2009). A further study in PV-infected cells demonstrated cleavage of Nup98, Nup153, TPR, Nup96, Nup58, Nup35, RanBP2, Nup214, and POM121, without attributing cleavage events to specific proteases (Krull et al., 2010). The cleavage of Nup62 (Park et al., 2010) and Nup98 (Park et al., 2015) by RV2 was found to selectively remove the FG-repeat domain, signifying a potential mechanism for the disruption of transport functions or of the NPC permeability barrier.

Interestingly, though all picornaviruses studied to date alter the NPC, the specific Nup targets and functional outcomes vary between strains. Among several RV strains, there were differential kinetics for the degradation of Nup98, Nup153, and Nup62 by 2A^pro^. Most strikingly, some strains showed little or no cleavage of Nup62 while it was efficiently degraded by others (Watters and Palmenberg, 2011). Further, the consequences of Nup degradation vary among RV strains, showing differential effects on trafficking mediated by various transport receptors, including importin-αβ, transportin 1, transportin 3, or Crm1/exportin 1 (Watters et al., 2017). This suggests that the impact of infection by specific picornavirus strains has unique effects on which cargoes are mislocalized, and on the relative balance of import vs export deficits. These differences may have implications for the efficiency of viral replication, as well as the potential deleterious effects on host cells.

Few studies have attempted to comprehensively survey the impact of a picornavirus infection on the NPC. A proteomic analysis in cells overexpressing CVB3 2A^pro^ identified several interacting partners of 2A^pro^ and showed that eukaryotic translation initiation factor 4G (eIF4G) and Nup98 were the most rapidly cleaved host proteins. This was associated with bidirectional deficits of NCT and redistribution of nuclear mRNA into cytoplasmic stress granules (Serganov et al., 2022).

Disruption of the NPC has been implicated in degenerative disorders of the motor neuron. In *C9orf72*-related amyotrophic lateral sclerosis (ALS), three independent screens identified deficits of NCT contributing to neurodegeneration (Freibaum et al., 2015; Jovičić et al., 2015; Zhang et al., 2015). These results have since been extended to sporadic ALS (Coyne et al., 2021) and other neurodegenerative disorders, including Huntington, Alzheimer, and Parkinson diseases (Grima et al., 2017; Eftekharzadeh et al., 2018; Shani et al., 2019). Furthermore, mutations in multiple Nup genes are sufficient to cause neurodegeneration, including several motor neuron diseases of childhood (Juhlen and Fahrenkrog, 2018).

Given the well-established role of NPC dysfunction in both picornavirus infection and neurotoxicity, and the similarity of disrupted NCT dysfunction in these settings, we wondered whether NPC dysfunction may be a mechanistic link between EV-D68 infection and neurotoxicity. As a first step in this line of inquiry, we sought to comprehensively characterize the impact of EV-D68 proteases on NPC composition and function, and to determine the contribution of EV-D68 proteases to motor neuron toxicity in models of AFM. Here, we show that 2A^pro^ is primarily responsible for wide-ranging deficits of NCT of protein cargoes via cleavage of a small number of Nups, and is neurotoxic in induced pluripotent stem cell (iPSC)-derived human spinal motor neurons.

## Results

### EV-D68 proteases disrupt NPC composition

**Figure 1.**
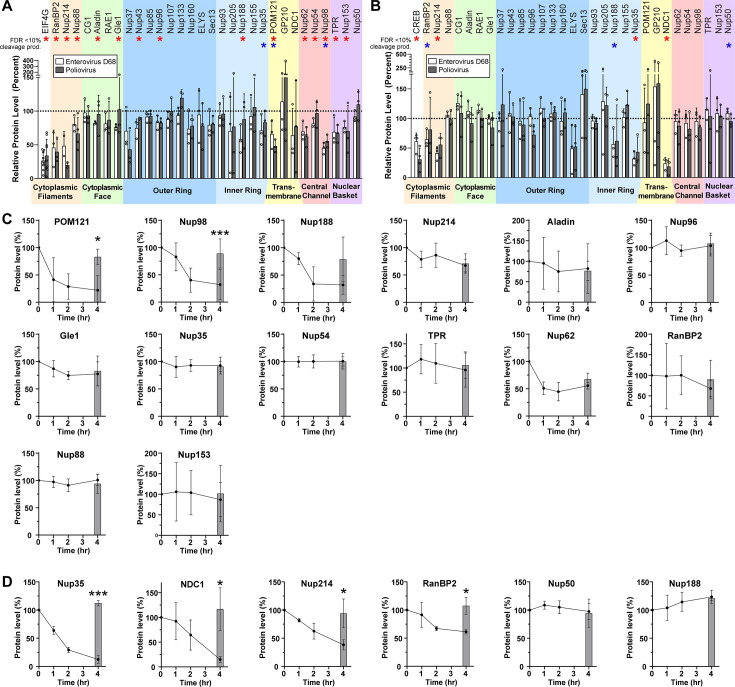
Enterovirus D68 proteases cleave several nucleoporins. (**A**) HEK cells were transfected with Enterovirus D68 2A^pro^, Poliovirus 1 2A^pro^, or empty vector control, and the indicated nucleoporins were quantified by western blot, normalized to control = 100%. EIF4G was included as a non-nucleoporin positive control. Mean ± SD of three independent replicates. Statistical comparison was by multiple t-tests of the EV-D68 2A^pro^ vs control, with multiple comparisons correction by the false discovery rate (FDR) method. Red asterisks represent significance with FDR<10%, blue asterisks represent samples for which a cleavage product was visible on the western blot irrespective of significance. (**B**) As in A, except transfecting with 3C^pro^. The positive control was CREB. (**C**) Nuclear lysates from HEK cells were incubated at 37°C with recombinant EV-D68 2A^pro^ at a ratio of 1:50 (protease:lysate by mass) for the indicated time. Gray bars represent lysate incubated for 4 hr at 37°C in the absence of protease as a negative control. Mean ± SD of three independent replicates. Statistical comparison was by t-test between the with- and without-protease samples at 4 hr. (**D**) As in C, except with EV-D68 3C^pro^ at a ratio of 1:200. *p<0.05, **p<0.01, ***p<0.001. Figure 1—source data 1.Raw data from quantifications of western blot data by densitometry for Figure 1A–D, reported as percent of control. Figure 1—source data 2.Labeled uncropped western blots for Figure 1A–D. Figure 1—source data 3.Original western blot images for Figure 1A. Figure 1—source data 4.Original western blot images for Figure 1B (part 1). Figure 1—source data 5.Original western blot images for Figure 1B (part 2). Figure 1—source data 6.Original western blot images for Figure 1B (part 3). Figure 1—source data 7.Original western blot images for Figure 1C (part 1). Figure 1—source data 8.Original western blot images for Figure 1C (part 2). Figure 1—source data 9.Original western blot images for Figure 1C (part 3). Figure 1—source data 10.Original western blot images for Figure 1C (part 4). Figure 1—source data 11.Original western blot images for Figure 1D (part 1). Figure 1—source data 12.Original western blot images for Figure 1D (part 2).

We screened for disruption of the NPC by expressing in HEK293T cells the 2A^pro^ and 3C^pro^ proteases from US/MO/2014-18947, a well-characterized neuropathogenic strain of EV-D68 that has been studied in animal and cell culture models of AFM (Liu et al., 2015; Brown et al., 2018; Hixon et al., 2019). We measured the levels of 30 Nups in whole-cell lysates by western blot. As positive controls, we used the homologous proteases from PV 1 Mahoney strain. To confirm protease activity, we also measured the levels of EIF4G (Ventoso et al., 1998) and CREB (Yalamanchili et al., 1997), well-established non-Nup substrates of 2A^pro^ and 3C^pro^, respectively. After correction for multiple comparisons, we identified 14 Nups whose levels were decreased after expression of EV-D68 2A^pro^ and four Nups whose levels were decreased after expression of 3C^pro^, revealing a widespread disruption of NPC composition following EV-D68 protease expression. In addition, western blots showed putative cleavage products of three Nups for each protease that were detectable only in the presence of protease (Figure 1A and B). Including these proteins, our total list of candidate Nup substrates of EV-D68 proteases numbered 15 for 2A^pro^ (RanBP2, Nup214, Nup88, Aladin, Gle1, Nup43, Nup96, Nup188, Nup35, POM121, Nup62, Nup54, Nup98, TPR, Nup153) and 6 for 3C^pro^ (RanBP2, Nup214, Nup188, Nup35, NDC1, Nup50).

**Figure 1—figure supplement 1.**
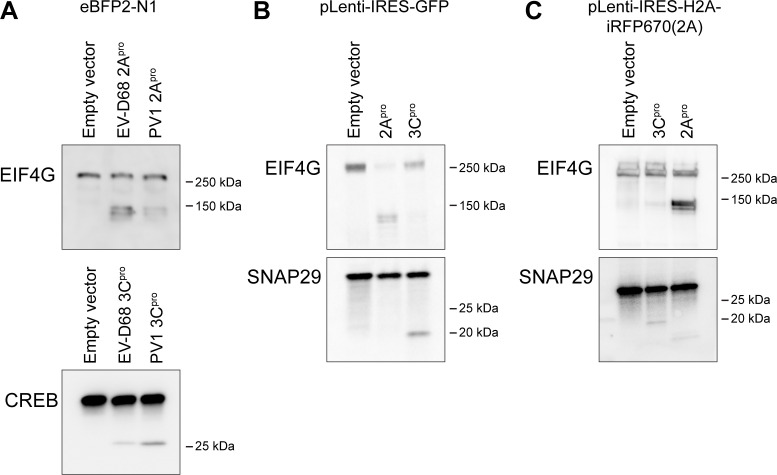
Western blots demonstrating evidence of 2A^pro^ and 3C^pro^ protease activity on well-established substrates following transfection with each of the expression vectors used in this study. Figure 1—figure supplement 1—source data 1.Labeled uncropped western blots for Figure 1—figure supplement 1. Figure 1—figure supplement 1—source data 2.Original western blot images for Figure 1—figure supplement 1.

We considered that alterations in the level of each Nup may be either a direct result of proteolytic cleavage or a secondary effect such as loss of a binding partner in the complex. We therefore sought to determine which of the candidate Nups are substrates of their respective EV-D68 protease. We performed *in vitro* assays of proteolytic cleavage for each Nup of interest by incubating HEK293T cell nuclear-fractionated lysates with recombinant 2A^pro^ or 3C^pro^ and quantifying Nup levels over time by western blot. All candidate Nups were detectable by this method with the exception of Nup43, which was excluded from further analysis. Surprisingly, of the 14 remaining candidate substrates of 2A^pro^, only Nup98 and POM121 were significantly proteolyzed in these nuclear preparations (Figure 1C). For 3C^pro^, four out of six candidates demonstrated significant evidence of proteolysis, including Nup35, NDC1, Nup214, and RanBP2 (Figure 1D).

### 2A protease alters nucleocytoplasmic trafficking of protein substrates

**Figure 2.**
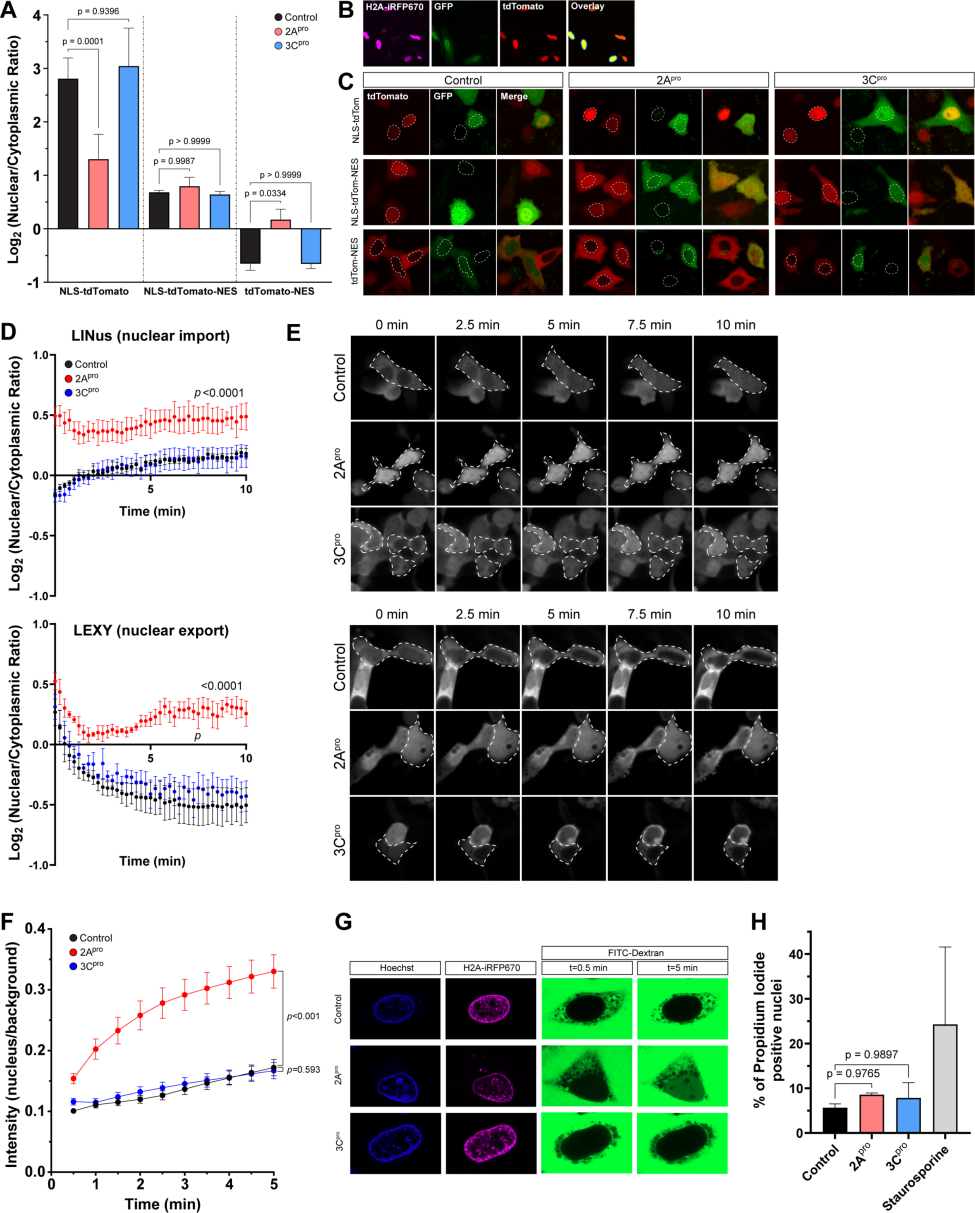
Enterovirus D68 2A protease impairs nucleocytoplasmic transport of protein cargoes and disrupts the nuclear pore complex permeability barrier. (**A**) tdTomato reporter assay showing nuclear-cytoplasmic ratios at steady state 20 hr after transfection of IRES-GFP, IRES-GFP-2A^pro^, or IRES-GFP-3C^pro^. Mean values of three independent replicates n=29,404 GFP+ cells in total, error bars are SD. Statistical comparison was by two-way ANOVA with Šídák’s multiple comparisons test. (**B**) Example output of image analysis protocol. In the overlay image, yellow represents the nuclei of transfected cells, defined by the area of expression of H2A-iRFP670 in GFP+ cells only. The surrounding dark blue ring is the perinuclear cytoplasm. The intensity of the reporter construct was measured in the red channel and reported as a ratio of nuclear to perinuclear cytoplasmic signal. (**C**) Example images from A. Dotted outlines mark the nuclei. (**D**) Kinetics of nuclear import (left) and export (right) measured by photoinducible LINus and LEXY reporter assays, respectively, in HeLa cells 20 hr after transfection with IRES-GFP, IRES-GFP-2A^pro^, or IRES-GFP-3C^pro^. Mean values of three biologically independent replicates, n=999 GFP+ cells for LINus and 1059 for LEXY in total. Error bars are SEM. Statistical comparison was by two-way ANOVA. (**E**) Example images from D. Dotted outlines mark GFP+ cells. (**F**) Dextran exclusion assay was performed in HeLa cells 15 hr after transfection with IRES-iRFP670-H2A, IRES-iRFP670-H2A-(2A)-2A^pro^, or IRES-iRFP670-H2A-(2A)-3C^pro^. n=45–46 cells per group pooled from three biologically independent replicates. Statistical comparison was by two-way ANOVA. (**H**) Propidium iodide viability assay. Mean values of three independent replicates, n=8042 cells in total. Error bars are SD. Statistical comparison was by one-way ANOVA with Tukey’s multiple comparison test. Figure 2—source data 1.Raw data used to generate Figure 2A. Figure 2—source data 2.Raw data used to generate Figure 2D. Figure 2—source data 3.Raw data used to generate Figure 2F. Figure 2—source data 4.Raw data used to generate Figure 2H.

We next sought to understand the functional impacts of Nup cleavage and altered NPC composition on NCT. We began by screening for steady-state alterations of nucleocytoplasmic localization using a reporter system based on the previously reported Shuttle-tdTomato (Zhang et al., 2015). We expressed NLS-tdTomato, tdTomato-NES, or Shuttle-tdTomato (hereafter referred to as NLS-tdTomato-NES) in HeLa cells, followed by GFP-labeled versions of each of the EV-D68 proteases or an empty vector GFP control. NLS-tdTomato is typically localized to the nucleus and tdTomato-NES to the cytoplasm, serving as readouts of nuclear import and export, respectively. NLS-tdTomato-NES is transported bidirectionally with an approximately uniform nucleocytoplasmic distribution at steady state. Its localization is expected to change during altered NCT only when there are unequal effects on nuclear import vs export. GFP-2A^pro^-expressing cells demonstrated significant alterations, wherein the NLS-tdTomato and tdTomato-NES reporters were significantly less partitioned into the nucleus or cytoplasm (Figure 2A–C, Figure 2—figure supplement 1).

**Figure 2—figure supplement 1.**
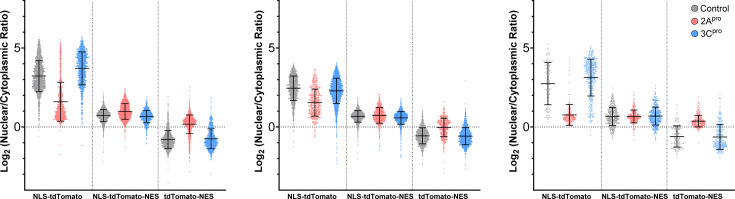
Source data for Figure 2A, showing individual cell-level data for three independent replicates. Bars are mean ± SD.

**Figure 2—figure supplement 2.**
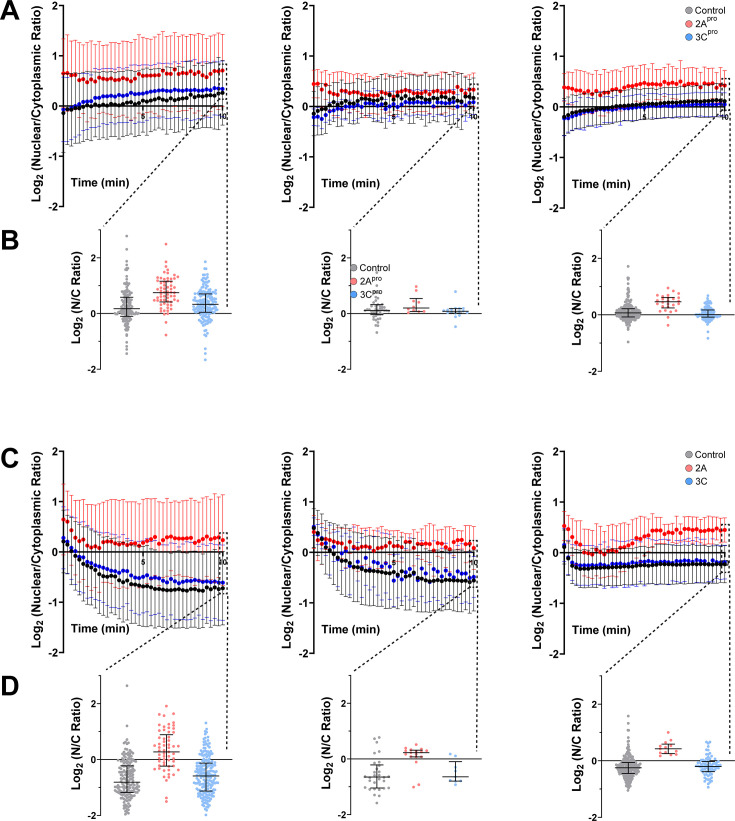
Source data for Figure 2D, showing mean ± SD values from three independent replicates for LINus (**A**) and LEXY (**C**) nucleocytoplasmic transport reporters, individual cell-level data is also presented at t=10 min for LINus (**B**) and LEXY (**D**). Bars are mean ± SD.

The above findings have two potential explanations which are not mutually exclusive: (1) 2A^pro^ impairs receptor-mediated nuclear import and export, or (2) 2A^pro^ disrupts the permeability barrier of the NPC, allowing for passive diffusion of macromolecules across the nuclear envelope. To test the first of these possibilities, we utilized a photoinducible reporter system to measure the kinetics of NCT. We used pDB22 (mCherry-LINus) for nuclear import (Niopek et al., 2014) and pDN122 (NLS-mCherry-LEXY) for nuclear export (Niopek et al., 2016). Both mCherry-LINus and NLS-mCherry-LEXY failed to translocate in HeLa cells following expression of 2A^pro^, but not 3C^pro^ (Figure 2D and E, Figure 2—figure supplement 2). These findings demonstrate deficits in both nuclear import and nuclear export of protein cargoes induced by 2A^pro^.

### 2A protease disrupts the permeability barrier of the NPC

To assess the integrity of the nuclear permeability barrier, we measured the exclusion of a high-molecular-weight fluorescently labeled dextran from the nucleus (Lénárt and Ellenberg, 2006) following selective permeabilization of the plasma membrane with digitonin (Adam et al., 1990). As expected, in digitonin-permeabilized HeLa cells expressing an empty vector control or 3C^pro^, FITC-dextran diffused rapidly into the cytoplasm but was excluded from the nucleus. By contrast, in 2A^pro^-expressing cells, FITC-dextran accumulated in the nucleus, indicating a disruption of the permeability barrier against diffusion of macromolecules (Figure 2F and G).

In sum, the above data indicate that disruption of the NPC specifically by 2A^pro^ leads to deficits in bidirectional NCT of protein cargoes, as well as disruption of the NPC permeability barrier against passive diffusion. To exclude the possibility that these phenomena are simply non-specific sequelae of cell death, we measured the uptake of propidium iodide in GFP+ HeLa cells transfected with IRES-GFP, IRES-GFP-2A^pro^, or IRES-GFP-3C^pro^, imaged and analyzed identically to those in the tdTomato and LINus/LEXY reporter assays, and found no significant increase in nuclear propidium iodide uptake between control and protease-expressing cells (Figure 2H).

### EV-D68 proteases do not disrupt RNA export

**Figure 3.**
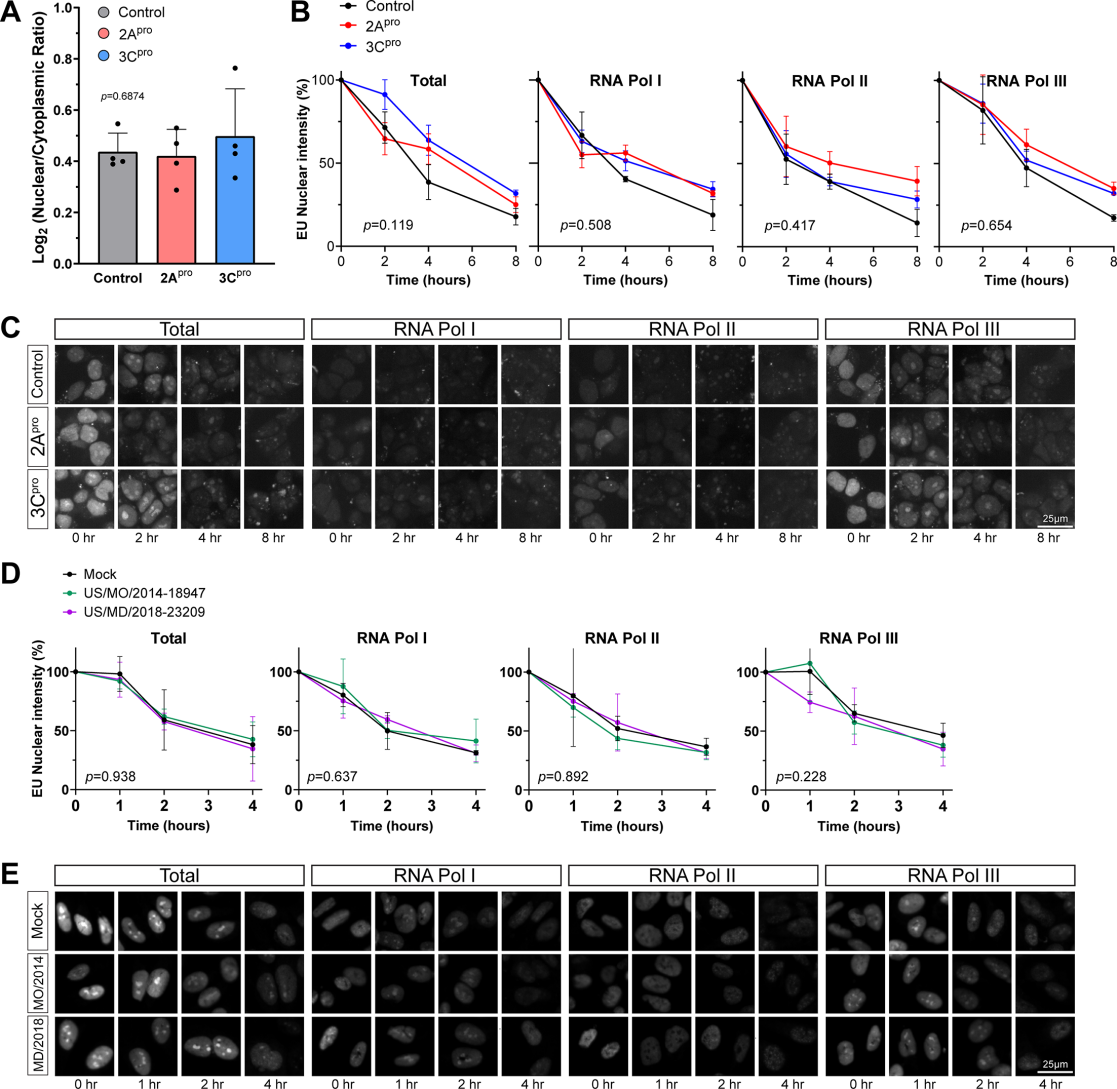
Enterovirus D68 proteases do not significantly alter RNA export. (**A**) Steady-state mRNA levels were measured by poly-A FISH in HeLa cells 20 hr after transfection with IRES-GFP, IRES-GFP-2A^pro^, or IRES-GFP-3C^pro^. Data represent the mean ± SD of four independent replicates, n=55,177 GFP+ cells in total. Statistical comparison was by one-way ANOVA with Šídák’s multiple comparisons test. (**B**) Nuclear export of EU-labeled RNAs in HEK cells by 1 hr pulse followed by 0–8 hr chase. EU labeling began 20 hr after transfection with IRES-GFP, IRES-GFP-2A^pro^, or IRES-GFP-3C^pro^. Pulse labeling of total RNA or primarily the products of the listed RNA polymerases was performed in the presence of pairs of combinations of actinomycin D, α-amanitin, and CAS 577784-91-9 as described in Materials and methods. Data represent the mean ± SEM of three independent replicates. n=253,015 GFP+ cells analyzed in total. Statistical comparison was by two-way ANOVA. (**C**) Representative images from the experiment described in B. (**D**) Nuclear export of EU-labeled RNAs in RD cells by 1 hr pulse followed by 0–4 hr chase. EU labeling began 24 hr after infections with EV-D68 strains at MOI 5 or mock infection as indicated. Data represent the mean ± SEM of three independent replicates. n=125,401 cells analyzed in total. Statistical comparison was by two-way ANOVA. (**E**) Representative images from the experiment described in D. Figure 3—source data 1.Raw data used to generate Figure 3A. Figure 3—source data 2.Raw data used to generate Figure 3B. Figure 3—source data 3.Raw data used to generate Figure 3D.

Given the substantial disruption of NCT of protein cargoes by 2A^pro^, we next asked whether RNA export from the nucleus is similarly altered. We used two complementary measurements of RNA transport in HEK293T cells transfected with EV-D68 proteases: poly-A RNA-FISH to label the steady-state distribution of native mRNA and 5-ethynyl uridine (EU) pulse-chase labeling of nascent RNAs to measure the kinetics of RNA export. In the case of the latter experiment, we also used combinations of RNA polymerase inhibitors actinomycin D, α-amanitin, and CAS 577784-91-9 to selectively label the products of RNA polymerase I, II, or III. In all cases, there was no significant alteration of nuclear-cytoplasmic localization of RNA (Figure 3A–C, Figure 3—figure supplement 1). We also performed the EU pulse-chase assay in RD cells infected with EV-D68. To control for potential strain-specific effects, we used EV-D68 strains from two different subclades and outbreak years, US/MO/2014-18947 (clade B1, year 2014) and US/MD/2018-23209 (clade B3, year 2018) (Figure 3D and E).

**Figure 3—figure supplement 1.**
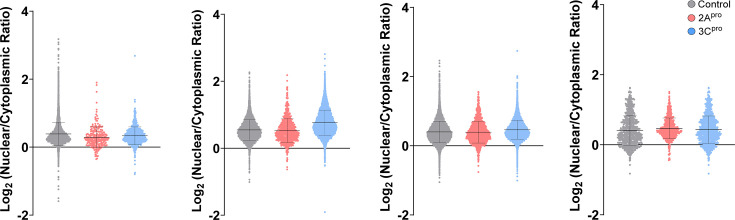
Source data for Figure 3A, demonstrating individual cell-level data across four independent replicates. Bars are mean ± SD.

### EV-D68 infection recapitulates the effect of 2A protease on NCT

**Figure 4.**
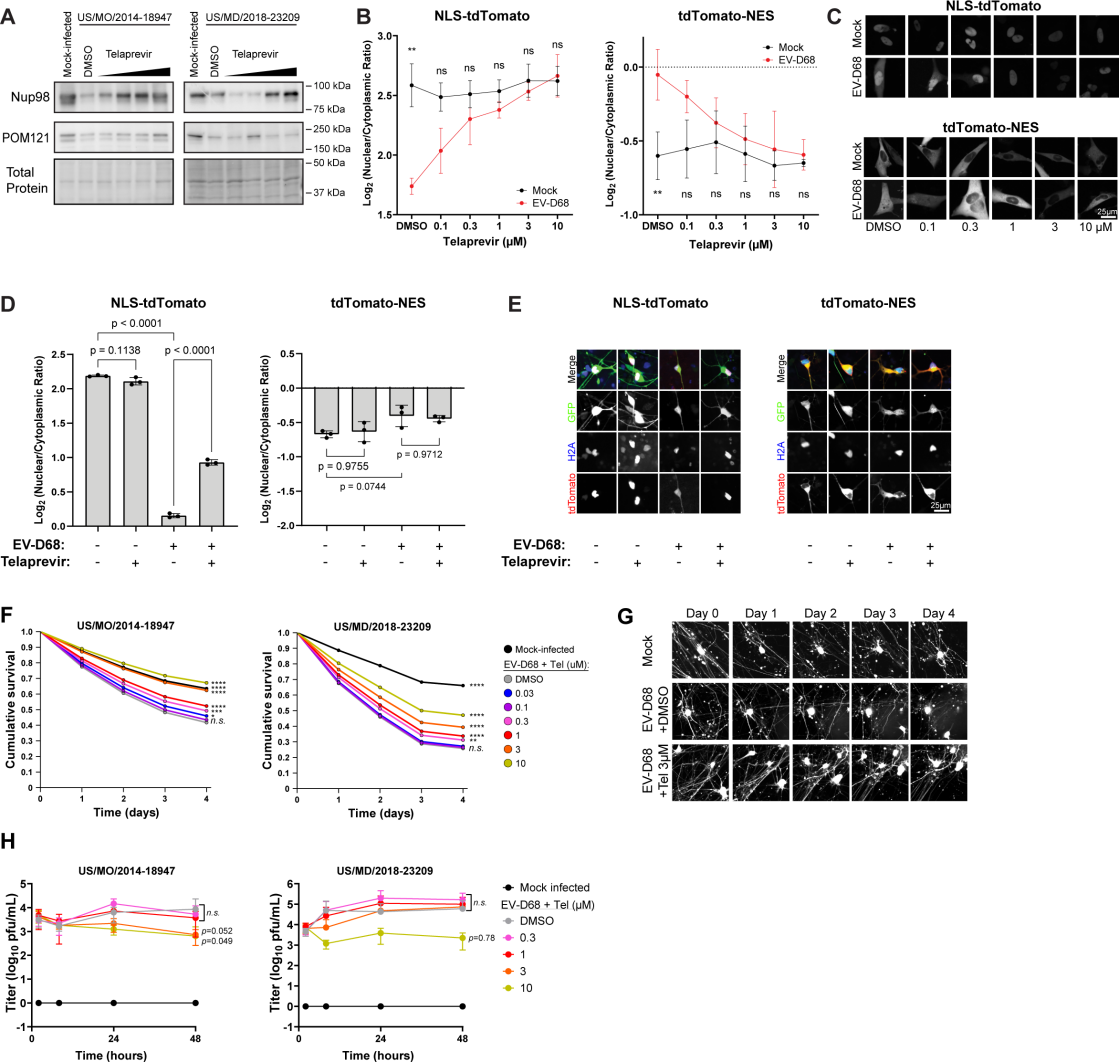
Effects of 2A protease during Enterovirus D68 (EV-D68) infection on nuclear pore complex (NPC) function and motor neuron toxicity. (**A**) Western blots of direct-induced motor neuron (diMN) lysates collected 24 hr after infection with the indicated strains of EV-D68 at MOI 5 followed by treatment with telaprevir at 0.3, 1, 3, and 10 µM. Total protein loading control was Bio-Rad stain-free imaging. One representative example out of three biologically independent replicates. (**B**) tdTomato reporter assays in RD cells showing nuclear-cytoplasmic ratios 24 hr after mock infection or infection with EV-D68 US/MO/2014-18947. Telaprevir was added following infection at the indicated concentrations. Data are mean of three independent replicates, n=11,847 cells in total for NLS-tdTomato and n=8941 for tdTomato-NES. (**C**) Representative images of tdTomato signal from B. (**D**) tdTomato reporter assays in diMN showing nuclear-cytoplasmic ratios 24 hr after mock infection or infection with EV-D68 US/MO/2014-18947, treated with DMSO or 3 µM telaprevir. Means of three replicates from independent iPSC lines. n=3388 cells in total for NLS-tdTomato and n=3371 for tdTomato-NES. (**E**) Representative images from panel D. (**F**) Survival of diMNs following infection with EV-D68 at MOI 5 with the indicated EV-D68 strains in the presence of varying concentrations of telaprevir or DMSO control. diMNs were generated from four independent iPSC lines. n*=*8512 and 7046 cells counted for US/MO/2014-18947 and US/MD/2018-23209, respectively. Statistical comparisons were by Cox proportional hazard regression. (**G**) Representative images of diMNs from the experiment presented in panel F. Note the fragmentation of neurites and loss of cell body shape, signifying cell death following EV-D68 infection, and the relative preservation following treatment with 3 µM telaprevir. (**H**) Growth curve of EV-D68 in the presence of varying concentrations of telaprevir or DMSO control. Infections were performed with the indicated strains at MOI 0.5, using diMNs generated from four independent iPSC lines for each virus. Error bars are SEM. Statistical comparisons were by two-way ANOVA with Dunnett’s multiple comparisons test between DMSO and telaprevir-treated groups. *p*<*0.05, **p*<*0.01, ***p*<*0.001, ****p*<*0.0001. Figure 4—source data 1.Labeled uncropped western blots for Figure 4A. Figure 4—source data 2.Original western blot images for Figure 4A. Figure 4—source data 3.Raw data used to generate Figure 4B. Figure 4—source data 4.Raw data used to generate Figure 4D. Figure 4—source data 5.Raw data used to generate Figure 4F. Figure 4—source data 6.Raw data used to generate Figure 4H.

We next sought to determine whether key deficits seen during transfection of recombinant proteases were also present during live virus infection. We also focused most of these experiments on a disease-relevant cell type, human iPSC-derived spinal motor neurons (direct-induced motor neurons [diMNs]), using multiple independent iPSC lines to control for the potential variability of these cells. We found that Nup98 and POM121 levels were decreased by EV-D68 infection in a similar manner as 2A^pro^-transfected cell lines, and this was at least partially reversed by the 2A^pro^ inhibitor telaprevir in a dose-dependent manner (Figure 4A). We also evaluated whether NCT disruption occurs during live virus infection by performing the NLS-tdTomato and tdTomato-NES reporter assays in RD cells infected with EV-D68. These cells demonstrated disrupted localization of both markers, and the deficits were rescued in a concentration-dependent fashion by telaprevir (Figure 4B and C). In diMNs, there was a strong disruption of NLS-tdTomato localization in response to infection but little, if any, alteration of tdTomato-NES localization in diMNs. The mislocalization of NLS-tdTomato was partially rescued by 3 µM telaprevir (Figure 4D and E, Figure 4—figure supplement 1).

**Figure 4—figure supplement 1.**
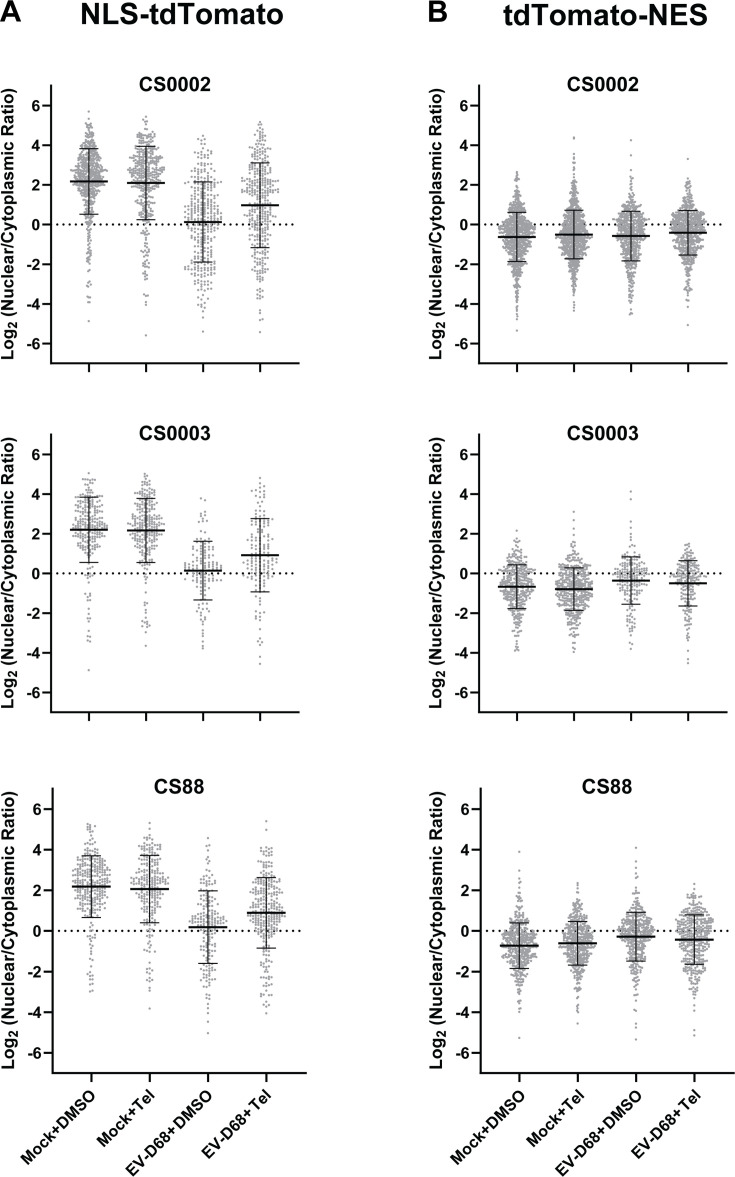
Source data for Figure 4D, showing individual cell-level data for the nuclear/cytoplasmic ratio of NLS-tdTomato (**A**) or tdTomato-NES (**B**) for three independent replicates in direct-induced motor neurons (diMNs) derived from induced pluripotent stem cell (iPSC) lines CS0002, CS0003, and CS88. Bars are mean ± SD.

### Inhibition of 2A protease rescues the toxicity of EV-D68 to spinal motor neurons out of proportion to its potential antiviral effect

In the above experiments, we demonstrated that EV-D68 2A^pro^ disrupts the composition of the NPC and has deleterious consequences on the maintenance of the NPC permeability barrier and nuclear transport of protein cargoes in cell lines and human spinal motor neurons. We next sought to determine the extent to which 2A^pro^ activity independently contributes to motor neuron injury during EV-D68 infection. GFP-labeled diMNs were infected with EV-D68 and imaged daily by high-content imaging with automated cell counting. These diMNs demonstrated gradual yet robust cell death in culture. The EV-D68 2A^pro^ inhibitor telaprevir led to a dose-dependent reduction in cell death, with statistically significant neuroprotection occurring at concentrations of 0.3 µM and above (Figure 4F and G). We considered the possibility that the rescue of NCT deficits and motor neuron toxicity may simply be due to an antiviral effect of telaprevir. However, the growth curve of EV-D68 in diMNs showed modest inhibition by telaprevir that achieved statistical significance in only one of two strains at a concentration of 10 µM, and in neither strain at lower concentrations (Figure 4H). We therefore infer that telaprevir protects motor neurons from EV-D68-induced cell death primarily because it inhibits the effects of 2A^pro^ activity on host targets.

## Discussion

Here, we have presented a detailed evaluation of the effects of EV-D68 proteases on the composition and function of the NPC. Our findings on Nup cleavage are compatible with previous findings in other picornaviruses and extend the literature by presenting one of the most comprehensive evaluations of Nups to date. Interestingly, we found widespread disruption of the NPC by EV-D68 proteases. The levels of up to 16 Nups were decreased by one or both proteases. By contrast, only 6 Nups were found to be direct proteolytic substrates of 2A^pro^ or 3C^pro^. This finding suggests that the remaining Nup alterations may be a secondary effect. For example, some of the altered Nups may be anchored to the NPC by other Nups that are themselves targets for proteolysis.

Our study also provides a mechanistic explanation for disrupted localization of nucleocytoplasmic cargoes. By using several complementary reporter systems to detect abnormalities of NCT dynamically and at steady state, we showed that the deficits are multifactorial, resulting from both disruption of the NPC’s permeability barrier against passive diffusion of macromolecules and from loss of receptor-mediated nuclear import and export.

It is striking that functional deficits in the NPC were entirely attributable to 2A^pro^, even though we only observed two definitive substrates of 2A^pro^: Nup98 and POM121. Nup98 has been well studied as a target of picornavirus 2A proteases, though studies have drawn differing conclusions regarding the extent and nature of the impact of Nup98 cleavage on NPC function (Park et al., 2008; Castelló et al., 2009; Serganov et al., 2022). The finding that 2A^pro^ also cleaves POM121 is especially interesting because of its role in motor neuron degeneration in ALS. POM121 appears to function as a ‘keystone’ for the NPC. In models of ALS, removal of POM121 from the NPC is sufficient to cause loss of multiple Nups, including Nup98, Nup107, Nup50, TPR, Nup133, GP210, and NDC1 (Coyne et al., 2020), a list that overlaps with what we observed following 2A^pro^ expression. In these ALS models, overexpression of POM121 is sufficient to reverse NPC deficits, and the mechanism of POM121 removal appears to be inappropriate activation of the ESCRT III pathway (Coyne et al., 2021; Coyne and Rothstein, 2021; Baskerville et al., 2024). In the context of EV-D68 infection, it remains to be determined whether Nup98 or POM121 is the primary driver of NPC pathology, if the mechanism of POM121 loss involves the ESCRT pathway or is only due to its cleavage by 2A^pro^, and whether deficits of NPC function can be reversed via repair of Nups or prevention of their cleavage. These questions will be a focus of future studies.

We have also demonstrated that 2A^pro^ activity contributes to NCT dysfunction and separately to cell death in motor neurons infected with EV-D68. Our approach was motivated by the fact that similar NPC deficits have been shown to be toxic in neurodegenerative disorders of the motor neuron. We therefore hypothesize that the 2A^pro^-dependent toxicity that we witnessed in diMNs is at least partially due to these deficits of NPC function. Concordant with our observation of 2A^pro^-dependent toxicity, a recent study demonstrated amelioration of the AFM phenotype in EV-D68-infected mice treated with telaprevir. The protection of spinal motor neurons in these mice occurred despite minimal effects on viral replication in the spinal cord, providing further support for a model in which telaprevir’s mechanism of action is primarily neuroprotective rather than antiviral (Frost et al., 2023). A significant limitation of our study, however, is that we cannot exclude potentially toxic effects of 2A^pro^ on aspects of host neuronal biology aside from the NPC. For example, 2A^pro^ impairs 5’-cap-dependent RNA translation via eIF4G cleavage (Ventoso et al., 1998) and inhibits stress granule formation (Visser et al., 2019), which could also contribute to toxicity in EV-D68-infected neurons. Further work will be required to disentangle the relative contributions of these pathways to motor neuron injury.

### Conclusion

We have built upon prior observations in several picornaviruses to show that EV-D68 proteases cause broad disruption of the composition and function of the NPC, and that these phenomena are attributable to the cleavage of a relatively small number of Nups by 2A^pro^. Further, 2A^pro^ contributes to motor neuron death in a model of EV-D68 infection. These findings motivate future work to evaluate the NPC or 2A^pro^ as potential therapeutic targets to treat or prevent paralysis in AFM.

## Materials and methods

### Plasmid construction

Plasmids were constructed from the components listed in the Key reagents table using T4 DNA Ligase (New England Biolabs) for MAP2-H2A-iRFP670 or In-Fusion Snap Assembly (Takara Bio) for all other plasmids following the manufacturer’s protocol. The products of restriction digests and PCRs were purified using 1% agarose gel electrophoresis. The band of interest was visualized using SYBR Safe dye (Thermo Fisher) under blue light to avoid UV exposure, cut out of the gel, and recovered using the GeneJet Gel Extraction Kit (Thermo Fisher). Transfections of non-neuronal cell lines were performed using FuGene HD per the manufacturer’s protocol (Promega). Transfection of motor neurons was by Lonza Nucleofection as described below. Of note, expression of EV-D68 proteases using a CMV promoter and a Kozak sequence as the ribosome binding site, while sufficient to introduce measurable protease activity (Figure 1—figure supplement 1), did not allow a sufficiently bright signal for imaging assays using fluorescently tagged proteases. This is a commonly encountered problem, owing to the inhibition of transcription and 5’-cap-dependent translation by enterovirus proteases, which we resolved by incorporating an internal ribosome entry site (IRES) in front of the fusion protein (Castelló et al., 2006).

### Viruses and titering

Viral strains were US/MO/2014-18947 (BEI Resources, NR-49129) and US/MD/2018-23209 (gift of Dr. Andrew Pekosz, Johns Hopkins Bloomberg School of Public Health). Viral stocks were prepared by inoculating RD cells cultured to approximately 90% confluency in T75 flasks at 33°C and 5% CO_2_ at an MOI of 0.01 in Infection Media (IM). Once cytopathic effect was evident (3–5 days), lysates were collected by three freeze-thaw cycles. Titering was performed by methylcellulose plaque assay. RD cells were plated in six-well tissue culture plates and grown to confluency. A ten-fold dilution series of each viral sample was prepared in IM. RD cells were washed with PBS+, and the viral dilutions were added in a volume of 250 µL/well and then placed in the 33°C incubator for 1 hr with gentle rocking every 15 min. The viral inoculum was aspirated, 2 mL of 1% methylcellulose in MEM was added to each well as an overlay medium, and the plates were returned to the 33°C incubator for 3 days. To fix and harvest the plates, 2 mL of 10% neutral buffered formalin (VWR, 10015-194) was added dropwise and incubated overnight at room temperature. The formalin/cellulose mixture was swirled and decanted off, and then the cells were stained with naphthol blue black (Millipore Sigma) overnight and washed with water. Plaques were counted manually in a well containing 30–100 plaques, and pfu/mL was calculated by (number of plaques)/(0.25 mL) × (dilution factor).

### Cell lines and iPSCs

RD cells, HEK293T cells, and HeLa cells were from ATCC (CCL-136, CRL-3216, and CRM-CCL-2, respectively). Authentication of these lines was by STR profiling. iPSC lines were from the Cedars Sinai Biomanufacturing Center repository and included lines CS0002, CS0003, CS88, CS0YX7, and CS8VTR. Authentication of iPSCs was by immunocytochemistry, karyotype G banding, alkaline phosphatase staining, PluriTest global gene expression profiling, pluripotency analysis by Scorecard assay, and STR profiling. All cell lines had negative testing for mycoplasma contamination. iPSCs were maintained in Essential 8 Flex media (Thermo Fisher) on six-well tissue culture plates coated with Cultrex UltiMatrix Reduced Growth Factor Basement Membrane Extract (R&D Systems BME001), manually cleaned to remove differentiated cells, and passaged as colonies using ReLeSR passaging reagent (STEMCELL Technologies). Motor neuron differentiation was performed following the diMN protocol reported previously (Li et al., 2021). In brief, iPSCs were grown to 30–40% confluency and then fed daily with S1 media for 6 days for neuroepithelial induction. On day 6, neuroepithelial cells were collected following incubation with Accutase (Millipore Sigma), using gentle pipetting to release cells from the centers of the colonies. Neuroepithelial cells were triturated to a single-cell suspension and then collected by centrifugation at 200×*g*. These cells were cryopreserved using Cryostor (STEMCELL Technologies) for storage in liquid nitrogen until later use. Day 6 neuroepithelial cells were thawed and replated into UltiMatrix-coated tissue culture dishes in S2 media with ROCK inhibitor Y-27632 (Cayman Chemical) at 20 µM, and then fed daily on days 7–12 with S2 without ROCK inhibitor. This stage generates motor neuron precursor cells. On day 12, motor neuron precursors were collected as a single-cell suspension with 0.05% Trypsin and replated onto the desired format for each experiment. Where indicated, nucleofection was performed at this step, prior to replating. From day 12 onward, cells were fed with S3 media to promote terminal maturation and maintenance of motor neurons. The diMN protocol produces cultures containing approximately 80% Tuj1^+^ neurons, including 40% Islet-1^+^ mature motor neurons and 40% Nkx6.1^+^ immature motor neurons (Li et al., 2021).

### Western blotting

Cells were harvested by scraping in ice-cold PBS, pelleted by centrifugation at 500×*g* for 5 min at 4°C, and PBS was aspirated off. Lysis was achieved by resuspension in ice-cold RIPA buffer (Millipore Sigma) supplemented with cOmplete Protease Inhibitor Cocktail (Millipore Sigma) followed by sonication. Protein concentrations were measured by DC protein assay (Bio-Rad). Samples were prepared by diluting a constant protein quantity by mass with deionized water and 4× Laemmli buffer (Bio-Rad) to a final concentration of 1×. Protein electrophoresis was performed in Bio-Rad Criterion Stain-Free gels and then transferred onto low-fluorescence PVDF membranes using the Transblot Turbo system (Bio-Rad). Total protein stain for confirmation of proper transfer and normalization of quantitative data was by Ponceau S stain (Bio-Rad; Figure 1A and B) or by stain-free imaging (Figure 1C and D, Figure 1—figure supplement 1, Figure 4A). Blots were blocked in 5% non-fat milk in TBS with 0.1% Tween-20 (TBS-T) for 1 hr. Primary antibodies were applied in TBS-T overnight at 4°C. Blots were then washed three times in TBS-T and incubated for 1 hr in secondary antibody at a concentration of 1:20,000. Blots were washed three additional times with TBS-T, twice with TBS, and then developed using Clarity Western ECL Substrate (Bio-Rad) or SuperSignal West Atto ECL substrate (Thermo Fisher). Images were acquired using a GE Healthcare ImageQuant LAS 4000 (Figure 1A and B) or Bio-Rad ChemiDoc MP Imaging System (Figure 1C and D, Figure 1—figure supplement 1). Quantification of western blot images was performed using ImageJ software and normalized to the total protein stain.

### Recombinant EV-D68 proteases

Recombinant proteases were produced based on the sequence of US/MO/2014-18947. 3C^pro^ was produced by Biomatik. 2A^pro^ produced by standard methods did not demonstrate evidence of protease activity. We therefore generated a SUMO-2A^pro^ fusion protein, based on a strategy previously reported to circumvent this technical limitation thought to be due to improper folding (Musharrafieh et al., 2019). The sequence of 2A^pro^ was codon-optimized for *Escherichia coli* using the IDT codon optimization tool (https://www.idtdna.com/pages/tools/codon-optimization-tool) and generated as synthetic DNA (Integrated DNA Technologies) for In-Fusion Snap Assembly into the peSUMOstar plasmid as detailed above to encode His_6_-SUMO-2A^pro^. BL21(DE3) competent cells (New England BioLabs) were transformed with peSUMOstar-2A^pro^, grown in a shaker incubator at 37°C in the presence of kanamycin 50 µg/mL to an optical density of 0.6–0.75, cooled to 18°C, and then protein expression was induced with 0.5 mM IPTG for 24 hr. Cells were harvested by centrifugation at 3500×*g* for 20 min. The cell pellet was resuspended and lysed in 50 mM sodium phosphate, 300 mM sodium chloride, 10 nM imidazole, 1 mg/mL lysozyme (Roche), and 3 units/mL Benzonase nuclease (Millipore Sigma) at pH 8.0 and incubated on ice for 30 min. The lysate was loaded onto Ni-NTA spin columns (QIAGEN) and washed with a buffer containing 50 mM sodium phosphate, 300 mM sodium chloride, and 20 nM imidazole at pH 8.0, then eluted with a buffer containing 50 mM sodium phosphate, 300 mM sodium chloride, and 500 nM imidazole at pH 8.0.

### Recombinant protease digestion assay

To generate a substrate enriched for Nups, a nuclear lysate was collected from HEK293T cells using the NE-PER extraction kit (Thermo Fisher). Nuclear lysates and recombinant proteases were dialyzed overnight at 4°C into reaction buffer (20 mM HEPES pH 7.4, 150 mM potassium acetate, and 1 mM DTT). Lysates were diluted to a concentration of 1 µg/µL in reaction buffer with proteases added at a ratio (protease:lysate by mass) of 1:50 for SUMO-2A^pro^ and 1:200 for 3C^pro^. Proteases were omitted in the case of negative control samples. The samples were incubated at 37°C with rotation at 300 rpm for the indicated times. The reaction was stopped via the addition of 4× Laemmli buffer to a final concentration of 1×, followed by incubation for 5 min at 100°C. Samples were analyzed by western blotting.

### NCT assays

HeLa cells or RD cells were plated onto UltiMatrix-coated 96-well glass-bottom plates (Cellvis), cultured in Complete Media, and transfected on day 1 with a reporter construct (NLS-tdTomato, NLS-tdTomato-NES, tdTomato-NES, mCherry-LINus, NLS-mCherry-LEXY) and the nuclear marker H2A-iRFP670. On day 2, they were transfected with the protease-containing vector or empty vector control, or infected with EV-D68 or mock-infected. Imaging was completed at the indicated number of hours after the second transfection or infection on a Molecular Devices ImageXpress Confocal high-content imaging system. For photoinducible reporters (mCherry-LINus and NLS-mCherry-LEXY), images were acquired every 15 s, each beginning with a 1 s pulse of the 488 nm laser at maximum intensity to maintain the reporter in its activated state. Image analysis was performed in an automated fashion using MetaXpress software (Molecular Devices). A custom analysis protocol was developed to select only GFP+ cells with size and morphology typical of live cells, to define the area of the nucleus using the H2A-iRFP670 signal, exclude nuclei with a condensed or mitotic morphology, and to define the perinuclear cytoplasm as a concentric ring outside of the nucleus. The average intensity of the reporter signal in the nucleus and perinuclear cytoplasm was measured and reported as Log_2_ of the nucleus/cytoplasm ratio. Cells were excluded from the analysis when the intensity of the reporter signal was too low to be distinguished from background or high enough to create shine-through artifact in the green channel, up to a maximum of 50% of the pixel depth of the image to ensure signal in the linear range. Due to inherent variability in signal intensity, these minimum and maximum intensity values were determined empirically for each independent biological replicate.

For assays in diMN, cells were nucleofected with hMAP2-pGreenZero, MAP2-H2A-iRFP670, and the reporter construct on day 12 of differentiation and plated to UltiMatrix-coated 96-well glass-bottom plates at a density of 2.5×10^5^ cells/well. On day 18, they were infected with EV-D68 at an MOI of 2.5. Imaging was performed at 24 hr post-infection. Analysis was as above, except that the perinuclear cytoplasm was defined as the intersection between the cytoplasmic GFP signal and a concentric ring outside the nucleus.

### Dextran exclusion assay

Digitonin permeabilization was carried out as previously described (Hayes et al., 2020) with some adjustments. Briefly, HeLa cells were seeded onto an UltiMatrix-coated 96-well glass-bottom plate (Cellvis) and maintained in Complete Media. Cells were transfected 15 hr before imaging at 60–70% confluency with bicistronic reporter constructs encoding a nuclear marker and the protease of interest or empty vector (pLenti-IRES-H2A-iRFP, pLenti-IRES-H2A-iRFP(P2A)2Apro, or pLenti-IRES-H2A-iRFP(P2A)3Cpro). To permeabilize, cells were kept on ice and washed with ice-cold PBS for 5 min. Digitonin was added at varying concentrations for 10 min in permeabilization buffer (PB; 20 mM HEPES, 110 mM KOAc, 5 mM Mg(OAc)2, 0.5 mM EGTA, 250 mM sucrose) to identify the concentration that permeabilized the plasma membrane while leaving the nuclear envelope intact. The optimized digitonin concentration varied depending on the cell density, but the same concentration was used across each biological replicate, with 30–40 µg/mL being used for analysis. Permeabilized cells were washed twice for 5 min with modified transport buffer (TRB; 20 mM HEPES, 110 mM KOAc, 2 mM Mg(OAc)2, 5 mM NaOAc, 0.5 mM EGTA, 250 mM sucrose). Cells were kept in TRB with Hoechst (5 µg/mL) on ice until imaging.

Imaging was performed on a Zeiss LSM880 Airyscan confocal microscope and processed using ImageJ. FITC-conjugated 70 kDa dextran (Millipore Sigma) was diluted in TRB and added to each well immediately before imaging at a final concentration of 0.6 mg/mL. Images were acquired every 30 s for 5 min. For each field, cells were analyzed only if the nuclei expressed H2A-iRFP670, had intact and visible nuclear membranes, were regularly shaped, and had clear and sharp boundaries between the nucleus and the cytoplasm. Images were excluded if a majority of the cells in the field were permeabilized regardless of transfection status, indicating general over-permeabilization. Cells were also excluded if they were impermeabilized (indicated by the absence of a visible nuclear membrane), if nuclei were not completely within the plane of imaging, or if there was drift of the focal plane during time course imaging. Data were reported as the average FITC intensity in the nucleus divided by the average FITC intensity of the extracellular background.

### Propidium iodide viability assay

HeLa cells were transfected as in the NCT assays, except omitting the reporter construct. Staurosporine 2 µM was added to the positive control group 2 hr before propidium iodide as a positive control. Propidium iodide 100 µg/mL was added to all groups 20 hr post-transfection and 15 min prior to imaging. Image acquisition and analysis were the same as for the NCT assays, selecting only GFP+ cells with normal size and morphology. Instead of calculating the nuclear-cytoplasmic ratio of signal in the red channel, the intensity of signal in the nucleus was recorded. To determine the minimum intensity cutoff for propidium iodide positivity, histograms were plotted for signal intensity in the staurosporine-treated group, and a cutoff for positive cells was determined by identifying the inflection point in the biphasic distribution.

### Poly-A FISH

HeLa cells were plated on UltiMatrix-coated 96-well glass-bottom plates (Cellvis) and transfected with pLenti-IRES-GFP, pLenti-IRES-GFP-2A^pro^, or pLenti-IRES-GFP-3C^pro^. Twenty hours later, they were washed with PBS, fixed for 30 min in 4% paraformaldehyde, washed two more times with PBS, then incubated for 10 min each with ice-cold 100% methanol followed by 100% ethanol. Cells were incubated with 1 ng/µL of Cy3-oligo-dT (Genelink) in hybridization buffer (30 µM sodium citrate, 300 µM sodium chloride, 10% formamide, 10% dextran sulfate, 50 µg yeast tRNA (Millipore Sigma) in DEPC-treated water) for 1 hr at 37°C. To enhance the GFP signal, the cells were then immunofluorescently stained for GFP. Cells were washed with PBS and then blocked in blocking solution (5% goat serum, 1% bovine serum albumin in PBS) for 1 hr at room temperature and then incubated in primary antibody against GFP diluted 1:200 in 1/4× blocking solution overnight at 4°C. The cells were then washed twice with PBS and then incubated with Alexa Fluor488-conjugated secondary antibody for 1 hr in 1/4× blocking solution. Finally, the cells were washed with PBS, counterstained with Hoechst 33342 nuclear stain (Cell Signaling) at 5 µg/mL for 15 min, and then mounted with PBS glycerol for imaging. Imaging was performed and analyzed on a Molecular Devices ImageXpress Confocal high-content imaging system as described above for NCT assays.

### EU pulse chase

HEK293T cells were plated on UltiMatrix-coated 96-well glass-bottom plates (Cellvis) and transfected with pLenti-IRES-GFP, pLenti-IRES-GFP-2A^pro^, or pLenti-IRES-GFP-3C^pro^. Twenty hours after transfection, the cell culture medium was replaced with fresh media containing 1 mM EU (Vector Laboratories) for a 1 hr pulse to label nascent RNA. To preferentially label the products of each RNA polymerase, inhibitors of the other two RNA polymerases were included during the EU pulse. These were 0.05 ng/mL actinomycin D, 0.05 ng/mL α-amanitin, and 50 µM CAS 577784-91-9 to inhibit RNA polymerases I, II, and III, respectively. The EU-containing media was aspirated off, washed once with PBS, and then chased with fresh Complete Media for 0, 2, 4, or 8 hr. For RD cells, the cells were infected with EV-D68 at MOI 5, and the pulse-chase labeling was begun 24 hr after infection, with the chase being completed at 0, 1, 2, or 4 hr after the end of the pulse. Cells were collected by fixation with 3% paraformaldehyde for 15 min, washed twice with PBS, and then permeabilized with 0.5% Triton X-100 for 15 min. EU was fluorescently labeled with AZDye594-azide via click chemistry using the Click&Go cell reaction kit following the manufacturer’s instructions (Vector Laboratories). The cells were immunofluorescently co-stained for GFP and counterstained with Hoechst 33342 as described above for the poly-A-FISH assay. Imaging and analysis were likewise performed as above, except nuclear signal intensity of the EU stain was reported instead of nuclear/cytoplasmic ratio, because the cytoplasmic signal was not consistently distinguishable from background. Intensities were normalized such that the intensity at t=0 was considered 100%.

### Motor neuron toxicity

Day 12 diMN precursors were transfected with hMAP2-pGreenZero using a Lonza Nucleofector 4D X unit, with the P3 primary cell kit on program DC-104. Cells were replated into UltiMatrix-coated 96-well glass bottom plates at a density of 100,000 cells/well in S3 media with 20 µM Y-27632. After 24 hrs, the cells were re-fed without Y-27632 and maintained in S3 media until day 32 of differentiation. These diMNs were infected with EV-D68 at MOI 5 in IM for 1 hr at 37°C with rocking every 15 min, then fed with S3 media. diMNs were imaged on a Molecular Devices ImageXpress Confocal high-content imaging system immediately following infection (considered t=0) and then once daily for 4 additional days. GFP+ neurons were counted via automated image analysis in MetaXpress software.

### Growth curves on motor neurons

Day 12 diMN precursors were plated onto UltiMatrix-coated 96-well plastic tissue culture plates at a density of 75,000 cells/well and maintained in S3 media until day 32 of differentiation. diMNs were infected with EV-D68 at MOI 0.5 as described above. Virus was collected at the indicated times by subjecting the plates to three freeze-thaw cycles. Viral titers were measured by methylcellulose plaque assay.

### Statistics

Statistical analyses and preparation of graphs were performed in GraphPad Prism 10 software. Statistical methods for each experiment are described in the corresponding figure legend.

### Materials availability statement

All plasmids newly created for this study are available by contacting the corresponding author. All other materials are available at the sources indicated in the Key resources table (Appendix 1).

## Data Availability

Source data for all figures have been included in the supporting files and are also deposited in the Johns Hopkins Data Repository, publicly available at https://doi.org/10.7281/T1OANANJ. The following dataset was generated: ZinnKM McLarenMW ImaiM JayaramMM RothsteinJ ElrickM 2026Data associated with the publication: Enterovirus D68 2A protease causes nuclear pore complex dysfunction and independently contributes to motor neuron toxicityJohns Hopkins Research Data Repository10.7281/T1OANANJPMC1327873742312942

## Additional files

MDAR checklist

## Appendix 1

**Appendix 1—key resources table:**

Reagent type (species) or resource	Designation	Source or reference	Identifiers	Additional information
Gene (Enterovirus D68)	2A	GenBank	KM851225.1	Nucleotides 3280–3720 of genomic cDNA
Gene (Enterovirus D68)	3C	GenBank	KM851225.1	Nucleotides 5341–5979 of genomic cDNA
Strain, strain background (Enterovirus D68)	US/MO/2014-18947	BEI Resources	NR-49129	
Strain, strain background (Enterovirus D68)	US/MD/2018-23209	Gift of Andrew Pekosz, Johns Hopkins School of Public Health		
Cell line (*Homo sapiens*)	RD	ATCC	CCL-136	
Cell line (*Homo sapiens*)	HEK293T	ATCC	CRL-3216 RRID:CVCL_0063	
Cell line (*Homo sapiens*)	HeLa	ATCC	CRM-CCL-2 RRID:CVCL_0030	
Cell line (*Homo sapiens*)	iPSC	Cedars Sinai Biomanufacturing Center	CS0002	
Cell line (*Homo sapiens*)	iPSC	Cedars Sinai Biomanufacturing Center	CS0003	
Cell line (*Homo sapiens*)	iPSC	Cedars Sinai Biomanufacturing Center	CS88	
Cell line (*Homo sapiens*)	iPSC	Cedars Sinai Biomanufacturing Center	CS0YX7	
Cell line (*Homo sapiens*)	iPSC	Cedars Sinai Biomanufacturing Center	CS8VTR	
Antibody	Aladin (rabbit polyclonal)	Bethyl Laboratories	A304-514A RRID:AB_2620708	(1:500)
Antibody	CG1 (NUPL2; Nup42) (rabbit polyclonal)	Thermo	PA5-88035 RRID:AB_2804606	(1:1000)
Antibody	CREB (rabbit polyclonal)	Cell Signaling	9197 RRID:AB_331277	(1:1000)
Antibody	eIF4G (rabbit polyclonal)	Cell Signaling	2498S RRID:AB_2096025	(1:1000)
Antibody	ELYS (rabbit polyclonal)	Bethyl Laboratories	A300-166A RRID:AB_2225879	(1:1000)
Antibody	GAPDH (mouse monoclonal)	Abcam	ab8245 RRID:AB_2107448	(1:10,000)
Antibody	GFP (mouse monoclonal)	Takara Bio	632375 RRID:AB_2756343	(1:20,000)
Antibody	Gle1 (rabbit polyclonal)	Novus	NBP2-47449 RRID:AB_3311113	(1:250)
Antibody	GP210 (rabbit polyclonal)	Bethyl Laboratories	A301-795A RRID:AB_1211450	(1:100)
Antibody	mAb414 (rabbit polyclonal)	Abcam	Ab24609 RRID:AB_448181	(1:1000)
Antibody	NDC1 (TMEM48) (rabbit polyclonal)	Novus Biologicals	NBP1-91603 RRID:AB_11030742	(1:1000)
Antibody	Nucleolin (rabbit polyclonal)	Cell Signaling	14574S RRID:AB_2798519	(1:10,000)
Antibody	Nup107 (rabbit polyclonal)	Thermo Fisher Scientific	19217-1-AP RRID:AB_10597702	(1:1000)
Antibody	Nup133 (mouse monoclonal)	Santa Cruz	sc-376699 RRID:AB_11149388	(1:1000)
Antibody	Nup153 (mouse monoclonal)	Abcam	Ab24700 RRID:AB_2154467	(1:1000)
Antibody	Nup155 (rabbit polyclonal)	Abcam	ab199528 RRID:AB_3751167	(1:1000)
Antibody	Nup160 (rabbit polyclonal)	Bethyl Laboratories	A301-790A RRID:AB_1211264	(1:100)
Antibody	Nup188 (rabbit polyclonal)	Bethyl Laboratories	A302-323A-T RRID:AB_1850233	(1:2500)
Antibody	Nup205 (rabbit polyclonal)	Abcam	ab157090 RRID:AB_3751168	(1:1000)
Antibody	Nup214 (rabbit polyclonal)	Abcam	ab70497 RRID:AB_1269607	(1:1000)
Antibody	Nup35 (Nup53) (rabbit polyclonal)	Proteintech	19819–1-AP RRID:AB_2878611	(1:1000)
Antibody	Nup37 (rabbit polyclonal)	Thermo Fisher Scientific	PA5-66007 RRID:AB_2664134	(1:200)
Antibody	Nup43 (mouse polyclonal)	Abcam	ab69447 RRID:AB_1269608	(1:1000)
Antibody	Nup50 (mouse monoclonal)	Santa Cruz	sc-398993 RRID:AB_2941760	(1:1000)
Antibody	Nup54 (rabbit polyclonal)	Sigma-Aldrich	HPA035929 RRID:AB_10671236	(1:200)
Antibody	Nup62 (rabbit polyclonal)	Millipore	MABE1043 RRID:AB_3751169	(1:2000)
Antibody	Nup85 (rabbit polyclonal)	Proteintech	19370–1-AP RRID:AB_10859826	(1:100)
Antibody	Nup88 (mouse monoclonal)	Santa Cruz	sc-365868 RRID:AB_10842170	(1:200)
Antibody	Nup93 (mouse monoclonal)	Santa Cruz	sc-374399 RRID:AB_10990113	(1:200)
Antibody	Nup96 (rabbit polyclonal)	Bethyl	A301-785A RRID:AB_1211486	(1:1000)
Antibody	Nup98 (rat polyclonal)	Abcam	ab50610 RRID:AB_881769	(1:5000)
Antibody	POM121 (rabbit polyclonal)	GeneTex	GTX102128 RRID:AB_10732546	(1:1000)
Antibody	RAE1 (mouse monoclonal)	Santa Cruz	sc-393252 RRID:AB_3751170	(1:200)
Antibody	RanBP2 (mouse monoclonal)	Santa Cruz	sc74518 RRID:AB_2176784	(1:200)
Antibody	Sec13 (rabbit polyclonal)	Proteintech	15397–1-AP RRID:AB_2186234	(1:1000)
Antibody	SNAP29 (rabbit polyclonal)	Abcam	ab181151 RRID:AB_2687668	(1:5000)
Antibody	TPR (rabbit polyclonal)	Bethyl Laboratories	A300-828A RRID:AB_597886	(1:1000)
Antibody	Goat Anti-Mouse IgG H&L HRP	Abcam	ab205719 RRID:AB_2755049	(1:20,000)
Antibody	Goat Anti-Rabbit IgG H&L HRP	Abcam	ab205718 RRID:AB_2819160	(1:20,000)
Antibody	Goat Anti-Rat IgG H&L HRP	Abcam	ab205720 RRID:AB_2941939	(1:20,000)
Antibody	Goat anti-Mouse IgG (H+L) Highly Cross-Adsorbed Secondary Antibody, Alexa Fluor 488	Thermo Fisher	A32723 RRID:AB_2633275	(1:500)
Recombinant DNA reagent	pmTurquoise-H2A (plasmid)	Addgene (Goedhart et al., 2012)	36207	
Recombinant DNA reagent	FU-MAP2-Gateway (plasmid)	Addgene (Addis et al., 2011)	43915	
Recombinant DNA reagent	eBFP2-N1 (plasmid)	Addgene (Michael Davidson)	54595	
Recombinant DNA reagent	pDB22 (mCherry-LINus) (plasmid)	Addgene (Niopek et al., 2014)	61342	
Recombinant DNA reagent	pDN122 (NLS-mCherry-LEXY) (plasmid)	Addgene (Niopek et al., 2016)	72655	
Recombinant DNA reagent	pLenti-DsRed-IRES-GFP (plasmid)	Addgene (Rousseaux et al., 2016)	92194	
Recombinant DNA reagent	hMAP2-pGreenZero (plasmid)	System Biosciences	SR10047PA-1	
Recombinant DNA reagent	Shuttle-tdTomato (plasmid)	Zhang et al., 2015		
Recombinant DNA reagent	peSUMOstar (plasmid)	LifeSensors, Inc		
Recombinant DNA reagent	CMV-(EV-D68)2Apro	This paper		Backbone: NheI/AgeI digest of eBFP2-N1 Insert: Synthetic DNA 1
Recombinant DNA reagent	CMV-(EV-D68)3Cpro	This paper		Backbone: NheI/AgeI digest of eBFP2-N1 Insert: Synthetic DNA 7
Recombinant DNA reagent	CMV-(PV1)2Apro	This paper		Backbone: NheI/AgeI digest of eBFP2-N1 Insert: Synthetic DNA 2
Recombinant DNA reagent	CMV-(PV1)3Cpro	This paper		Backbone: NheI/AgeI digest of eBFP2-N1 Insert: Synthetic DNA 8
Recombinant DNA reagent	pLenti-IRES-GFP	This paper		Backbone: PCR amplification of pLenti-DsRed-IRES-GFP (primers MJE_69, MJE_71) Recircularization via In-Fusion Snap Assembly
Recombinant DNA reagent	pLenti-IRES-GFP-2Apro	This paper		Backbone: BsrGI digest of pLenti-IRES-GFP Insert: Synthetic DNA 11
Recombinant DNA reagent	pLenti-IRES-GFP-3Cpro	This paper		Backbone: BsrGI digest of pLenti-IRES-GFP Insert: Synthetic DNA 12
Recombinant DNA reagent	pLenti-IRES-H2A-iRFP	This paper		Backbone: PCR amplification of pLenti-IRES-GFP (primers MTI_1, MTI_7) Insert: PCR amplificaion of H2A-iRFP from MAP2-H2A-iRFP670 with addition of P2A site (primers MTI_5, MTI_13)
Recombinant DNA reagent	pLenti-IRES-H2A-iRFP(P2A)2Apro	This paper		Backbone: PCR amplification of pLenti-IRES-GFP-2A (primers MTI_2, MTI_8) Insert: PCR amplificaion of H2A-iRFP from MAP2-H2A-iRFP670 with addition of P2A site (primers MTI_5, MTI_13)
Recombinant DNA reagent	pLenti-IRES-H2A-iRFP(P2A)3Cpro	This paper		Backbone: PCR amplification of pLenti-IRES-GFP-3C (primers MTI_12, MTI_9) Insert: PCR amplificaion of H2A-iRFP from MAP2-H2A-iRFP670 with addition of P2A site (primers MTI_5, MTI_13) Insert: Synthetic DNA 23
Recombinant DNA reagent	NLS-tdTomato	This paper		PCR amplification of Shuttle-tdTomato, followed by recircularization (primers MJE_10, MJE_11)
Recombinant DNA reagent	tdTomato-NES	This paper		PCR amplification of Shuttle-tdTomato, followed by recircularization (primers MJE_8, MJE_53)
Recombinant DNA reagent	MAP2-pmTurquoise-H2A	This paper		Backbone: PCR amplification of pmTurquoise-H2A (primers KEB_1, KEB_2) Insert: PCR amplification of FU-MAP2-Gateway (primers KEB_3, KEB_4)
Recombinant DNA reagent	MAP2-H2A-iRFP670	This paper		Backbone: AgeI/NotI digest of MAP2-pmTurqoise-H2A Insert: Synthetic DNA 22, digested with AgeI/NotI
Recombinant DNA reagent	peSUMOstar-2Apro	This paper		Backbone: BsaI digest of peSUMOSTAR Insert: Synthetic DNA 23
Sequence-based reagent	KEB_1	This paper	PCR primers	CGGAACTCCATATATGGGCTATGAACTAAT
Sequence-based reagent	KEB_2	This paper	PCR primers	GGTTTAGTGAACCGTCAGATCCGCA
Sequence-based reagent	KEB_3	This paper	PCR primers	ATATATGGAGTTCCGCAGCTGGCCTTTTTGGTTCTCAT
Sequence-based reagent	KEB_4	This paper	PCR primers	GGTTTAGTGAACCGTTCGGTTAGAGACAAGCTGAAGAATCTACCG
Sequence-based reagent	MJE_10	This paper	PCR primers	CTCGAGCAGAAACTCATCTCAGAAGAGGATC
Sequence-based reagent	MJE_11	This paper	PCR primers	TGAGTTTCTGCTCGAGCTGTAGCTTGTACAGCTCGTCCATGC
Sequence-based reagent	MJE_53	This paper	PCR primers	CATGCCCCCGCCTCCCATGGTGGCACGCGTCG
Sequence-based reagent	MJE_69	This paper	PCR primers	CACCGGCGGCCTAGCCGCCCCTCTC
Sequence-based reagent	MJE_70	This paper	PCR primers	CACCGGCGGCCTAGCAACCATGG
Sequence-based reagent	MJE_8	This paper	PCR primers	GGAGGCGGGGGCATGGTGAG
Sequence-based reagent	MTI_1	This paper	PCR primers	GAGAACCCTGGACCTTCTAGATAACTGATCCTCTCCC
Sequence-based reagent	MTI_12	This paper	PCR primers	GAGAACCCTGGACCTGGTCCAGGCTTCGGAGGAGTTTTTG
Sequence-based reagent	MTI_13	This paper	PCR primers	AGGTCCAGGGTTCTCCTCCACGTCGCCAGCCTGCTTCAGCAGGCTGAAGTTAGTAGCGCGTTGGTGGTGGG
Sequence-based reagent	MTI_2	This paper	PCR primers	GAGAACCCTGGACCTGGTCCAGGCTTCGG
Sequence-based reagent	MTI_5	This paper	PCR primers	AATATGGCCACAACCATGTCGGGACGTGGC
Sequence-based reagent	MTI_7	This paper	PCR primers	GAGAACCCTGGACCTGGACCAGG
Sequence-based reagent	MTI_8	This paper	PCR primers	GAGAACCCTGGACCTGGACCAGGGTTCG
Sequence-based reagent	MTI_9	This paper	PCR primers	GAGAACCCTGGACCTGGACCAGGGTTCGATT
Sequence-based reagent	1	This paper	Synthetic DNA	GTCAGATCCGCTAGCGCCACCATGGGTCCAGGCTTCGGAGGAGTTTTTGTAGGGTCTTTTAAAATAATCAACTATCACTTGGCCACTACAGAAGAGAGACAGTCAGCTATCTATGTGGATTGGCAATCAGACGTCTTGGTTACCCCCATTGCTGCTCATGGAAGGCACCAAATAGCAAGATGCAAGTGCAACACAGGGGTTTACTATTGTAGGCACAAAAACAGAAGTTACCCGATTTGCTTTGAAGGCCCAGGGATTCAATGGATTGAACAAAATGAATATTACCCAGCAAGGTACCAGACCAATGTACTTTTGGCAGTTGGTCCTGCGGAAGCAGGAGATTGCGGTGGTTTACTAGTTTGTCCACATGGGGTAATCGGTCTTCTTACAGCAGGAGGGGGTGGAATTGTAGCTTTCACTGATATCAGGAATTTGCTATGGTTAGATACTGATGCTATGGAACAATGGGATCCACCGGTCG
Sequence-based reagent	2	This paper	Synthetic DNA	GTCAGATCCGCTAGCGCCACCATGGGATTCGGACACCAAAACAAAGCGGTGTACACTGCAGGTTACAAAATTTGCAACTACCACTTGGCCACTCAGGATGATTTGCAAAACGCAGTGAACGTCATGTGGAGTAGAGACCTCTTAGTCACAGAATCAAGAGCCCAGGGCACCGATTCAATCGCAAGGTGCAATTGCAACGCAGGGGTGTACTACTGCGAGTCTAGAAGGAAATACTACCCAGTATCCTTCGTTGGCCCAACGTTCCAGTACATGGAGGCTAATAACTATTACCCAGCTAGGTACCAGTCCCATATGCTCATTGGCCATGGATTCGCATCTCCAGGGGATTGTGGTGGCATACTCAGATGTCACCACGGGGTGATAGGGATCATTACTGCTGGTGGCGAAGGGTTGGTTGCATTTTCAGACATTAGAGACTTGTATGCCTACGAAGAAGAAGCCATGGAACAATGGGATCCACCGGTCG
Sequence-based reagent	7	This paper	Synthetic DNA	GTCAGATCCGCTAGCGCCACCATGGGACCAGGGTTCGATTTTGCACAAGCCATAATGAAGAAAAATACCGTCATTGCAAGGACTGAAAAGGGTGAGTTCACCATGCTGGGTGTATATGATAGGGTAGCGGTCATCCCCACACACGCATCTGTTGGAGAAACCATTTACATTAATGATGTAGAGACTAAAGTTTTAGATGCGTGTGCACTTAGAGACTTGACTGATACAAACTTAGAGATAACCATAGTCAAATTAGACCGTAATCAAAAATTTAGAGATATCAGACATTTTCTGCCCAGATATGAGGATGATTACAATGACGCTGTGCTTAGCGTACATACATCAAAATTCCCAAATATGTATATCCCAGTTGGACAAGTCACCAATTATGGCTTCTTGAACCTAGGTGGTACACCGACGCACCGCATTTTAATGTATAACTTCCCAACAAGAGCTGGCCAGTGTGGTGGTGTGGTGACAACTACAGGTAAGGTGATAGGAATACATGTAGGTGGAAATGGAGCTCAAGGATTTGCAGCAATGCTACTACACTCTTACTTTTCCGATACACAAGGTGAGATAGTTAGTAGTGAAAAGAGTGGGGTGTGCATTAACGCACCGGCAAAGACTAAACTCCAACCTAGTGTTTTCCATCAAGTTTTTTAATGGGATCCACCGGTCG
Sequence-based reagent	8	This paper	Synthetic DNA	GTCAGATCCGCTAGCGCCACCATGGGACCAGGGTTCGATTACGCAGTGGCTATGGCTAAAAGAAACATTGTTACAGCAACTACTAGCAAGGGAGAGTTCACTATGTTAGGAGTCCACGACAACGTGGCTATTTTACCAACCCACGCTTCACCTGGTGAAAGCATTGTGATCGATGGCAAAGAAGTGGAGATCTTGGATGCCAAAGCGCTCGAAGATCAAGCAGGAACCAATCTTGAAATCACTATAATCACTCTAAAGAGAAATGAAAAGTTCAGAGACATTAGACCACATATACCTACTCAAATCACTGAGACAAATGATGGAGTCTTGATCGTGAACACTAGCAAGTACCCCAATATGTATGTTCCTGTCGGTGCTGTGACTGAACAGGGATATCTAAATCTCGGTGGGCGCCAAACTGCTCGTACTCTAATGTACAACTTTCCAACCAGAGCAGGACAGTGTGGTGGAGTCATCACATGTACTGGGAAAGTCATCGGGATGCATGTTGGTGGGAACGGTTCACACGGGTTTGCAGCGGCCCTGAAGCGATCATACTTCACTCAGAGTCAATAATGGGATCCACCGGTCG
Sequence-based reagent	11	This paper	Synthetic DNA	ATGGACGAGCTGTACAAGTCCGGACTCAGATCTCGAGCTCAAGCTTCGAATTCTGCAGTCGACGGTACCGCGGGCCCGGGTCCAGGCTTCGGAGGAGTTTTTGTAGGGTCTTTTAAAATAATCAACTATCACTTGGCCACTACAGAAGAGAGACAGTCAGCTATCTATGTGGATTGGCAATCAGACGTCTTGGTTACCCCCATTGCTGCTCATGGAAGGCACCAAATAGCAAGATGCAAGTGCAACACAGGGGTTTACTATTGTAGGCACAAAAACAGAAGTTACCCGATTTGCTTTGAAGGCCCAGGGATTCAATGGATTGAACAAAATGAATATTACCCAGCAAGGTACCAGACCAATGTACTTTTGGCAGTTGGTCCTGCGGAAGCAGGAGATTGCGGTGGTTTACTAGTTTGTCCACATGGGGTAATCGGTCTTCTTACAGCAGGAGGGGGTGGAATTGTAGCTTTCACTGATATCAGGAATTTGCTATGGTTAGATACTGATGCTATGGAACAATAAGTACAAGTAATCTAG
Sequence-based reagent	12	This paper	Synthetic DNA	ATGGACGAGCTGTACAAGTCCGGACTCAGATCTCGAGCTCAAGCTTCGAATTCTGCAGTCGACGGTACCGCGGGCCCGGGACCAGGGTTCGATTTTGCACAAGCCATAATGAAGAAAAATACCGTCATTGCAAGGACTGAAAAGGGTGAGTTCACCATGCTGGGTGTATATGATAGGGTAGCGGTCATCCCCACACACGCATCTGTTGGAGAAACCATTTACATTAATGATGTAGAGACTAAAGTTTTAGATGCGTGTGCACTTAGAGACTTGACTGATACAAACTTAGAGATAACCATAGTCAAATTAGACCGTAATCAAAAATTTAGAGATATCAGACATTTTCTGCCCAGATATGAGGATGATTACAATGACGCTGTGCTTAGCGTACATACATCAAAATTCCCAAATATGTATATCCCAGTTGGACAAGTCACCAATTATGGCTTCTTGAACCTAGGTGGTACACCGACGCACCGCATTTTAATGTATAACTTCCCAACAAGAGCTGGCCAGTGTGGTGGTGTGGTGACAACTACAGGTAAGGTGATAGGAATACATGTAGGTGGAAATGGAGCTCAAGGATTTGCAGCAATGCTACTACACTCTTACTTTTCCGATACACAATAAGTACAAGTAATCTAG
Sequence-based reagent	22	This paper	Synthetic DNA	ATGGCGCGTAAGGTCGATCTCACCTCCTGCGATCGCGAGCCGATCCACATCCCCGGCAGCATTCAGCCGTGCGGCTGCCTGCTAGCCTGCGACGCGCAGGCGGTGCGGATCACGCGCATTACGGAAAATGCCGGCGCGTTCTTTGGACGCGAAACTCCGCGGGTCGGTGAGCTACTCGCCGATTACTTCGGCGAGACCGAAGCCCATGCGCTGCGCAACGCACTGGCGCAGTCCTCCGATCCAAAGCGACCGGCGCTGATCTTCGGTTGGCGCGACGGCCTGACCGGCCGCACCTTCGACATCTCACTGCATCGCCATGACGGTACATCGATCATCGAGTTCGAGCCTGCGGCGGCCGAACAGGCCGACAATCCGCTGCGGCTGACGCGGCAGATCATCGCGCGCACCAAAGAACTGAAGTCGCTCGAAGAGATGGCCGCACGGGTGCCGCGCTATCTGCAGGCGATGCTCGGCTATCACCGCGTGATGTTGTACCGCTTCGCGGACGACGGCTCCGGGATGGTGATCGGCGAGGCGAAGCGCAGCGACCTCGAGAGCTTTCTCGGTCAGCACTTTCCGGCGTCGCTGGTCCCGCAGCAGGCGCGGCTACTGTACTTGAAGAACGCGATCCGCGTGGTCTCGGATTCGCGCGGCATCAGCAGCCGGATCGTGCCCGAGCACGACGCCTCCGGCGCCGCGCTCGATCTGTCGTTCGCGCACCTGCGCAGCATCTCGCCCTGCCATCTCGAATTTCTGCGGAACATGGGCGTCAGCGCCTCGATGTCGCTGTCGATCATCATTGACGGCACGCTATGGGGATTGATCATCTGTCATCATTACGAGCCGCGTGCCGTGCCGATGGCGCAGCGCGTCGCGGCCGAAATGTTCGCCGACTTCTTATCGCTGCACTTCACCGCCGCCCACCACCAACGCTAA
Sequence-based reagent	23	This paper	Synthetic DNA	GAACAGATTGGAGGTGGTCCTGGTTTTGGTGGCGTCTTTGTGGGTTCTTTCAAAATAATTAACTACCACCTGGCGACGACAGAGGAACGCCAATCAGCGATTTATGTTGACTGGCAAAGCGATGTCTTGGTTACCCCGATTGCAGCACACGGACGTCACCAAATTGCCCGGTGTAAATGCAATACGGGAGTATATTATTGTAGACATAAGAACCGTAGTTACCCCATTTGTTTTGAAGGACCAGGCATCCAATGGATCGAGCAGAACGAGTACTACCCAGCCAGATACCAAACGAACGTACTGCTGGCGGTGGGTCCTGCGGAAGCTGGCGATTGTGGGGGCTTGTTGGTGTGTCCGCACGGCGTAATTGGATTACTGACTGCCGGGGGTGGTGGTATCGTGGCGTTTACTGATATTCGCAATCTTCTGTGGTTAGATACTGATGCCATGGAACAATGATGACTAGAGGATCCGAAT
Other	Complete Media (CM)		Cell culture media	Dulbecco’s modified Eagle medium (Thermo Fisher 11995073) 10% fetal bovine serum (Thermo Fisher 16140071) 1% penicillin/streptomycin (Thermo Fisher 15140122) 2 mM L-glutamine (Thermo Fisher 25030081) 1 mM Sodium Pyruvate (Millipore Sigma S8636)
Other	Infection Media (IM)		Cell culture media	Dulbecco’s modified Eagle medium (Thermo Fisher 11995073) 2.5% fetal bovine serum (Thermo Fisher 16140071) 1% penicillin/streptomycin (Thermo Fisher 15140122) 2 mM L-glutamine (Thermo Fisher 25030081) 1 mM Sodium Pyruvate (Millipore Sigma S8636)
Other	Stage 1 diMN Media (S1)	Li et al., 2021	Cell culture media	50% Iscove’s Modified Dulbecco’s Medium (IMDM) (Thermo Fisher 12440053) 50% Ham’s F12 nutrient mix (Thermo Fisher 11765062) 1× Non-essential amino acids (Thermo Fisher 11140050) 1× Penicillin-Streptomycin (Thermo Fisher 15140122) 1× B27 Supplement (Thermo Fisher 17504044) 1× N2 Supplement (Thermo Fisher 17502048) 0.2 µM LDN-193189 (Stemgent 04-0074) 10 µM SB431542 (STEMCELL Technologies 72234) 3 µM CHIR99021 (Millipore Sigma SML1046)
Other	Stage 2 diMN Media (S2)	Li et al., 2021	Cell culture media	50% IMDM (Thermo Fisher 12440053) 50% Ham’s F12 nutrient mix (Thermo Fisher 11765062) 1× Non-essential amino acids (Thermo Fisher 11140050) 1× Penicillin-Streptomycin (Thermo Fisher 15140122) 1× B27 Supplement (Thermo Fisher 17504044) 1× N2 Supplement (Thermo Fisher 17502048) 0.2 µM LDN-193189 (Stemgent 04-0074) 10 µM SB431542 (STEMCELL Technologies 72234) 3 µM CHIR99021 (Millipore Sigma SML1046) 0.1 µM All-trans retinoic acid (Millipore Sigma R2625) 1 µM SAG (Cayman Chemicals 11914)
Other	Stage 3 diMN Media (S3)	Li et al., 2021	Cell culture media	50% IMDM (Thermo Fisher 12440053) 50% Ham’s F12 nutrient mix (Thermo Fisher 11765062) 1× Non-essential amino acids (Thermo Fisher 11140050) 1× Penicillin-Streptomycin (Thermo Fisher 15140122) 1× B27 Supplement (Thermo Fisher 17504044) 1× N2 Supplement (Thermo Fisher 17502048) 0.1 µM Compound E (Millipore Sigma 565790) 2.5 µM DAPT (Millipore Sigma D5942) 0.1 µM db-cAMP (Millipore Sigma 28745) 0.5 µM All-trans retinoic acid (Millipore Sigma R2625) 1 µM SAG (Cayman Chemicals 11914) 200 ng/mL Ascorbic acid (Millipore Sigma A4544) 10 ng/mL Brain-derived neurotrophic factor (PeproTech 450-02) 10 ng/mL Glial cell-derived neurotrophic factor (PeproTech 450-10)
Other	PBS+		Cell culture solution	Phosphate-buffered saline (Gibco 10010023) 100 g/L MgCl_2_ (Sigma) 100 g/L CaCl_2_ (Sigma)
