# Supplementary figures and images for: Enterovirus D68 2A protease causes nuclear pore complex dysfunction and independently contributes to motor neuron toxicity (part 1 of 4)
Source: eLife. 2026 Jun 18;14:RP108672. doi: 10.7554/eLife.108672 (PMC13278737; doi:10.7554/eLife.108672)

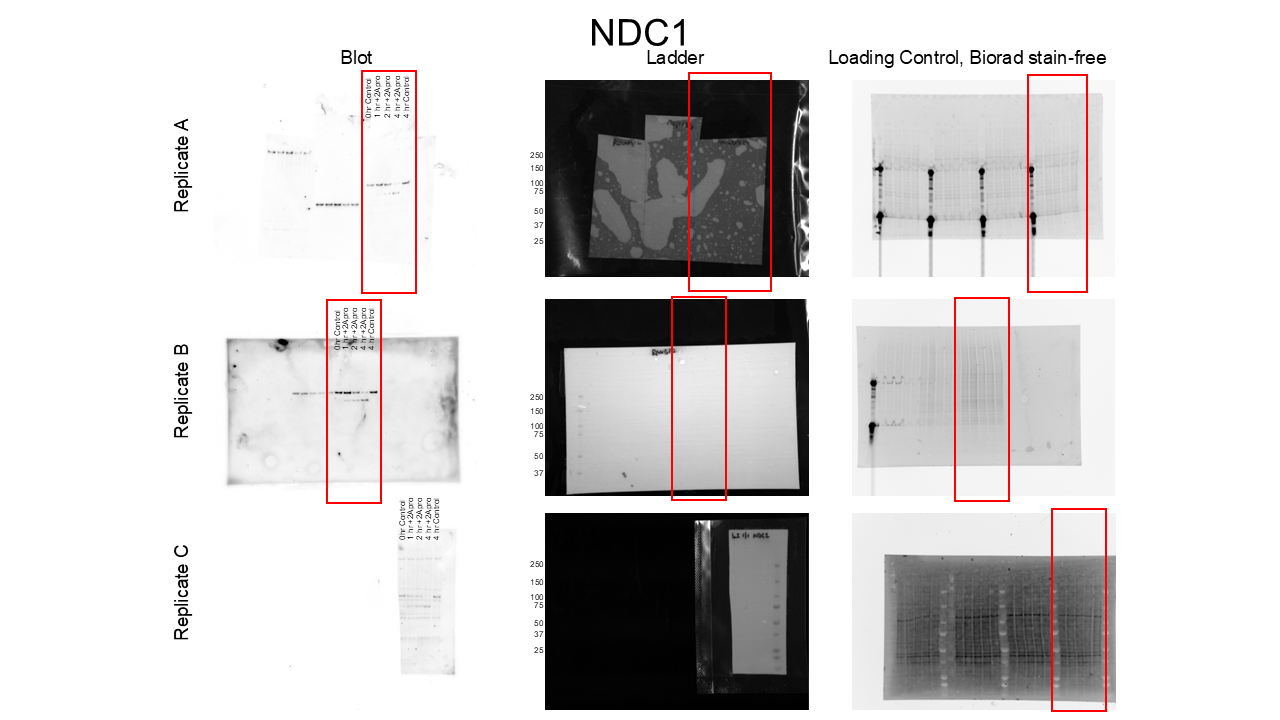

Supplement: Figure 1—source data 2. [file elife-108672-fig1-data2.zip › Figure 1D western blots/NDC1.TIF]

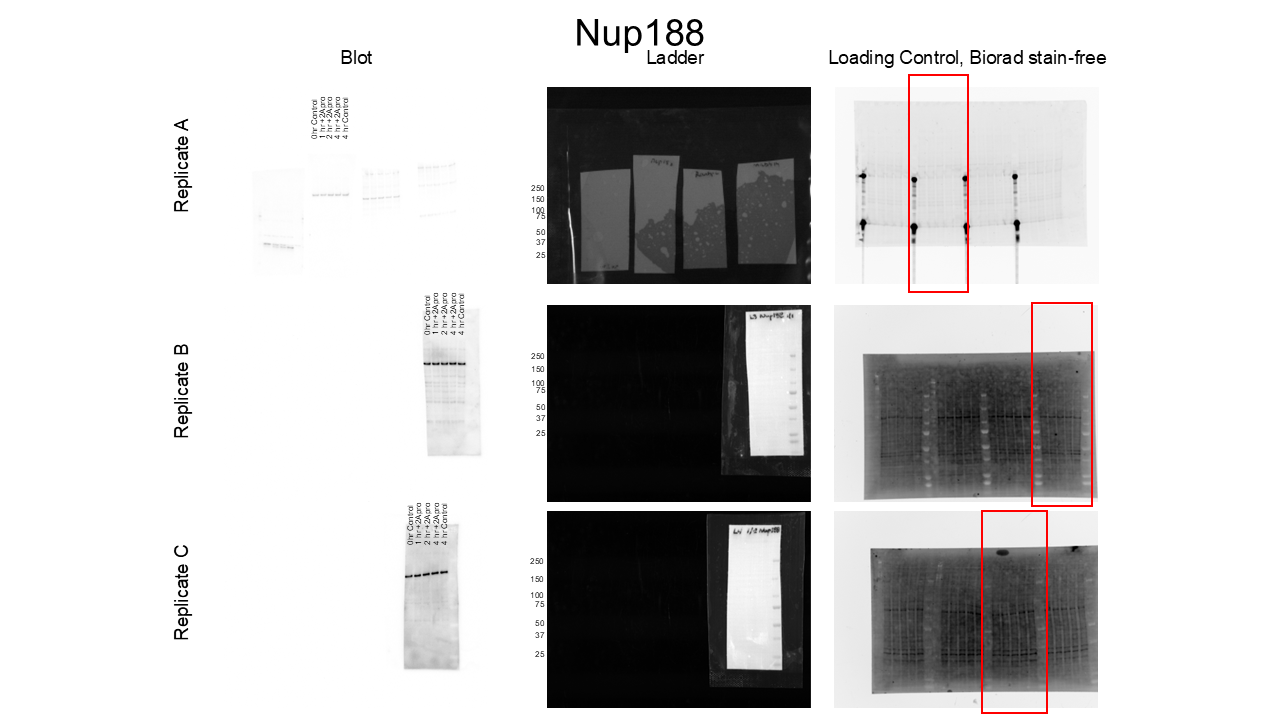

Supplement: Figure 1—source data 2. [file elife-108672-fig1-data2.zip › Figure 1D western blots/Nup188.TIF]

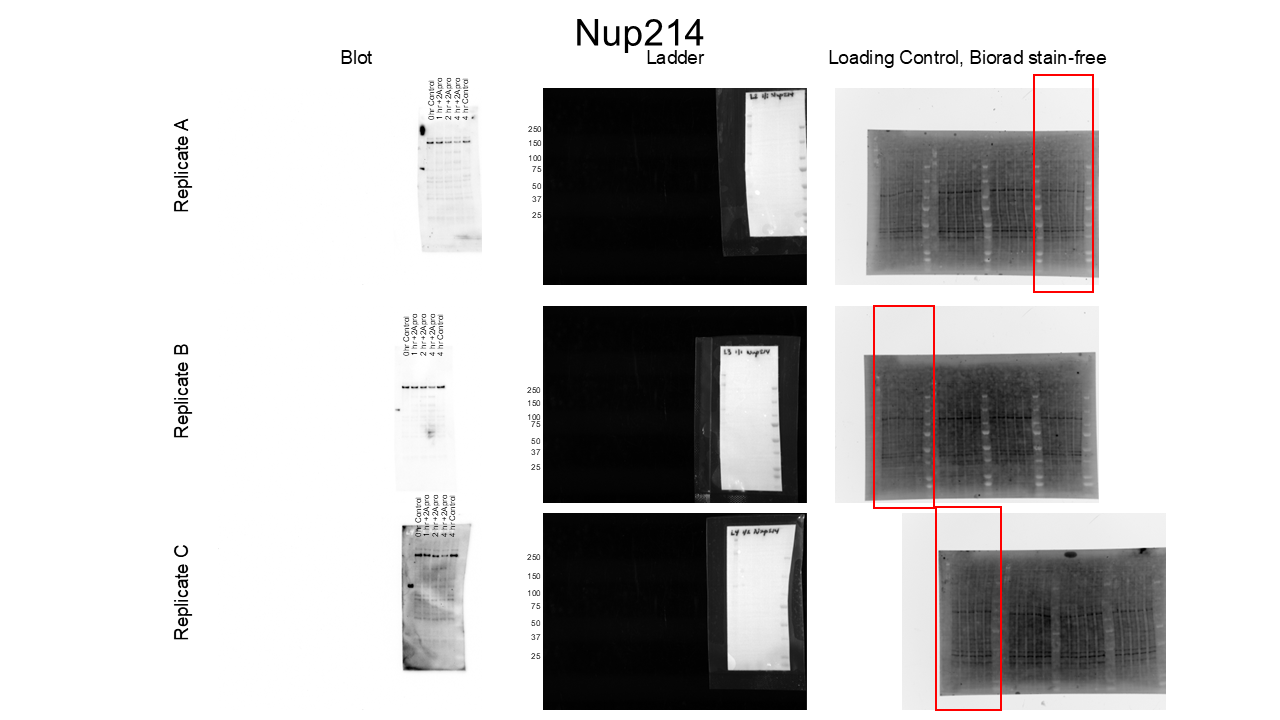

Supplement: Figure 1—source data 2. [file elife-108672-fig1-data2.zip › Figure 1D western blots/Nup214.TIF]

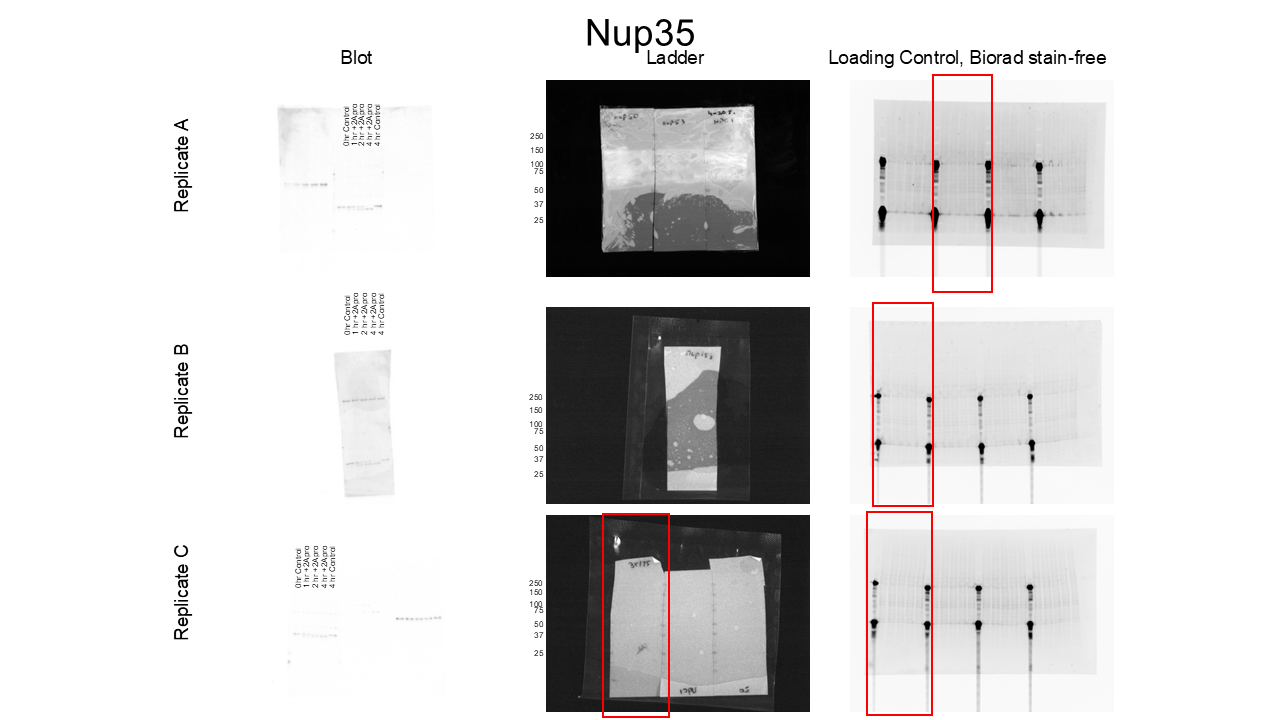

Supplement: Figure 1—source data 2. [file elife-108672-fig1-data2.zip › Figure 1D western blots/Nup35.TIF]

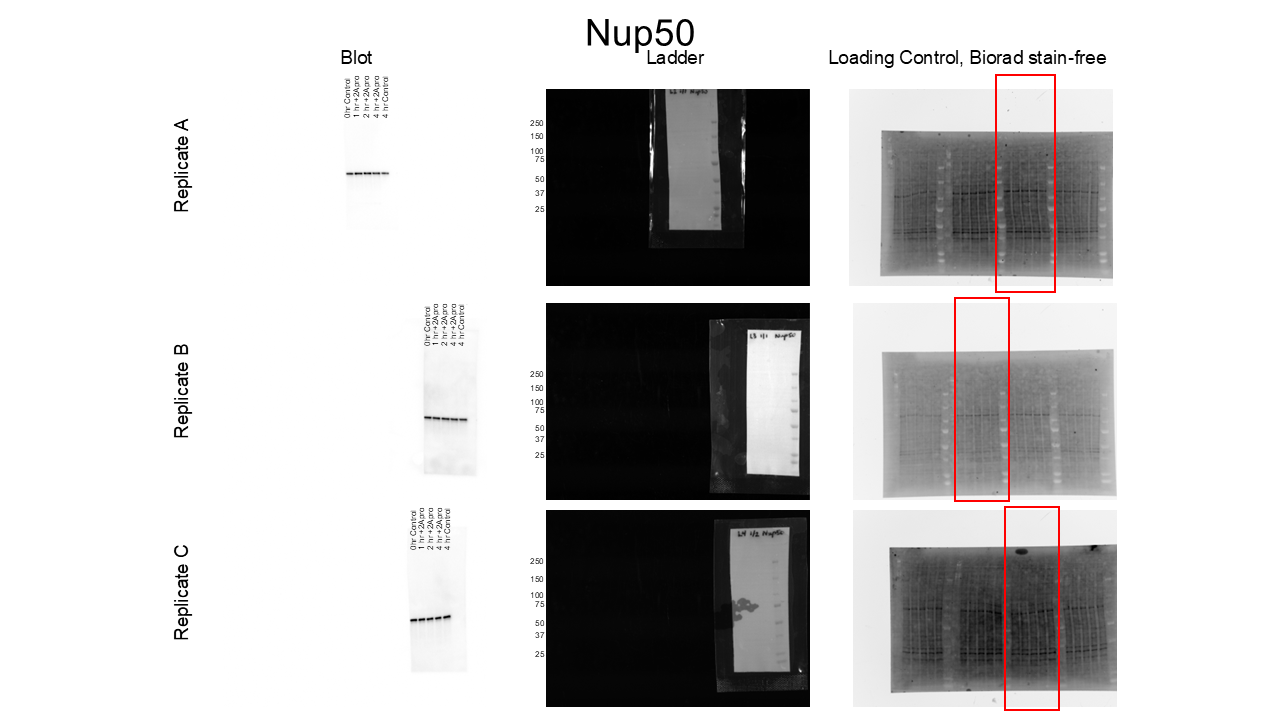

Supplement: Figure 1—source data 2. [file elife-108672-fig1-data2.zip › Figure 1D western blots/Nup50.TIF]

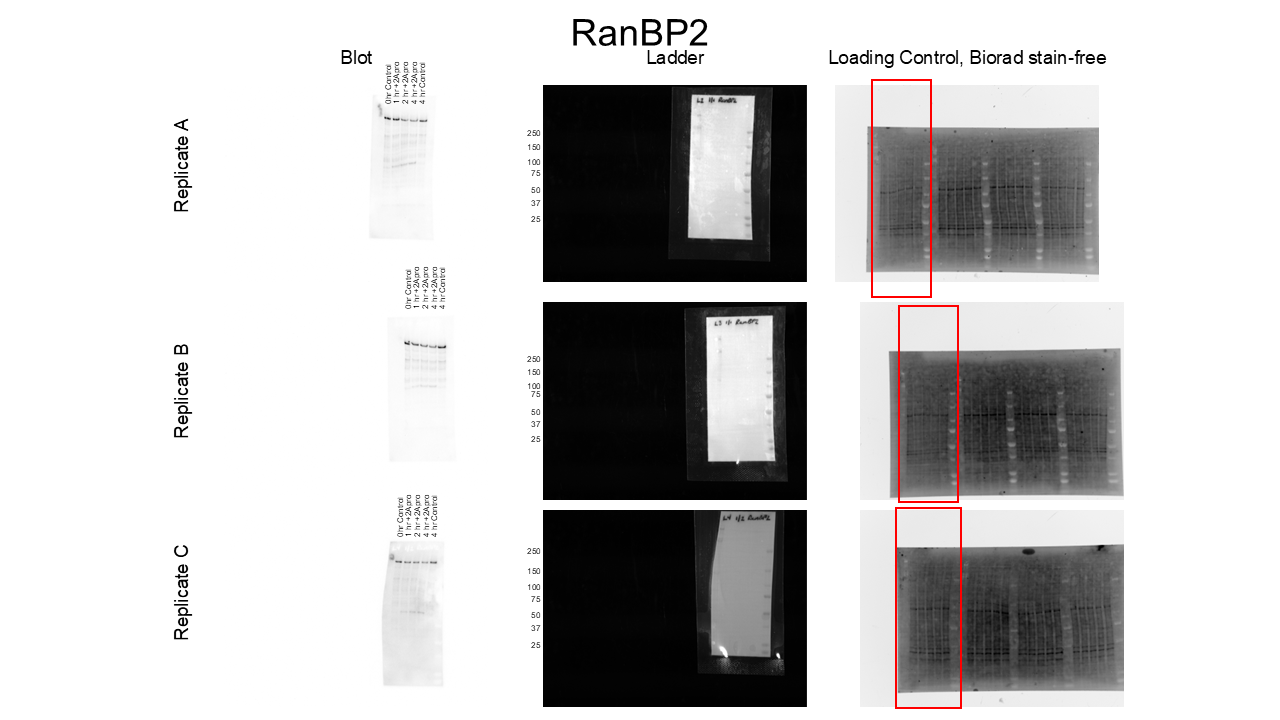

Supplement: Figure 1—source data 2. [file elife-108672-fig1-data2.zip › Figure 1D western blots/RanBP2.TIF]

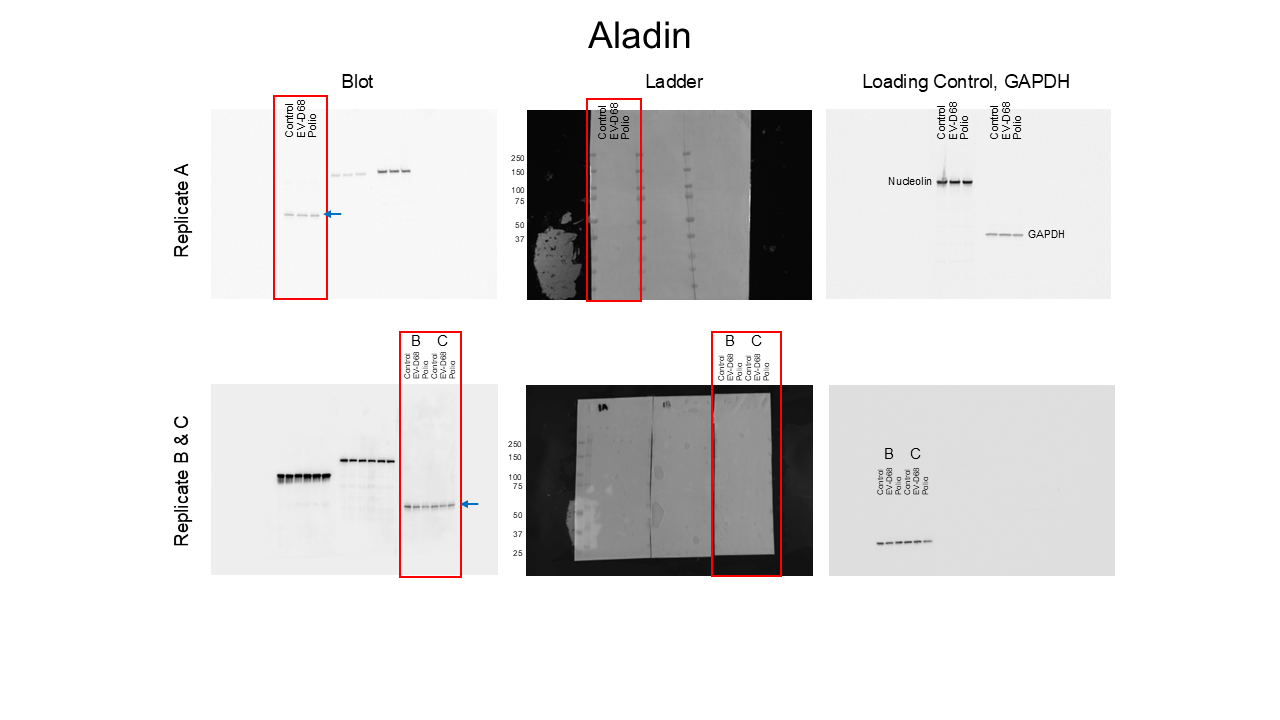

Supplement: Figure 1—source data 2. [file elife-108672-fig1-data2.zip › Figure 1A western blots/Aladin.TIF]

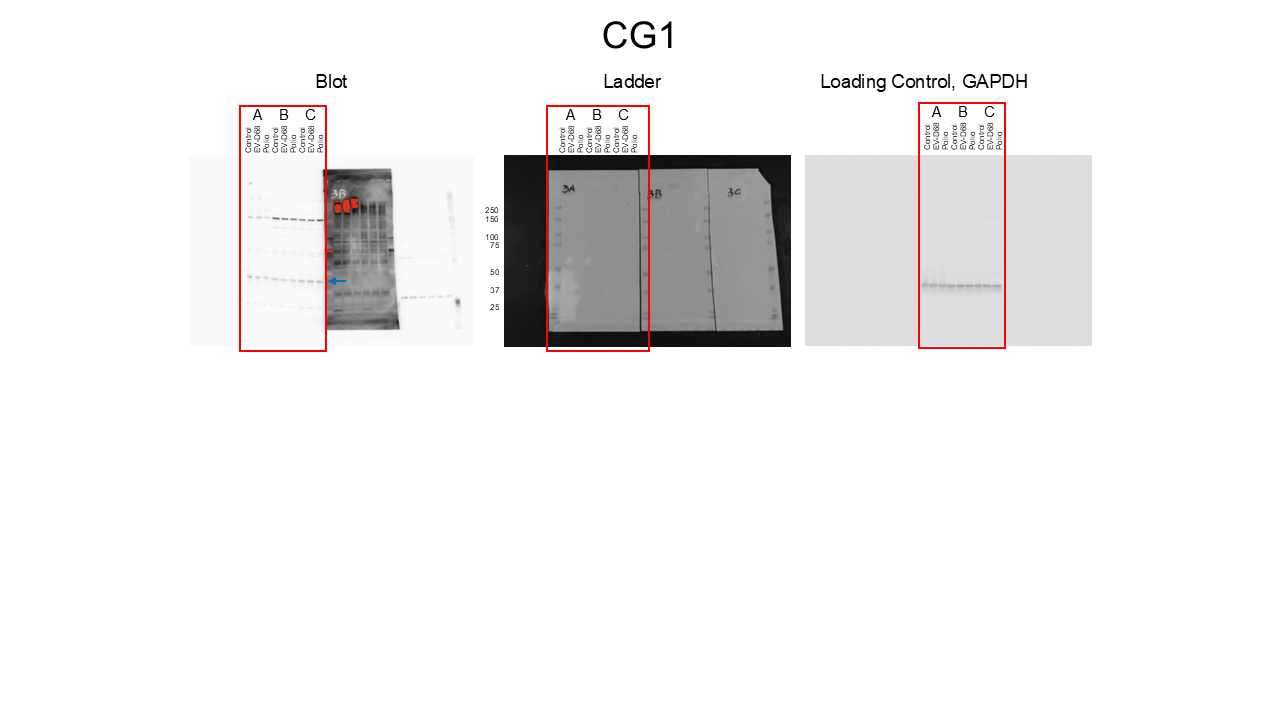

Supplement: Figure 1—source data 2. [file elife-108672-fig1-data2.zip › Figure 1A western blots/CG1.TIF]

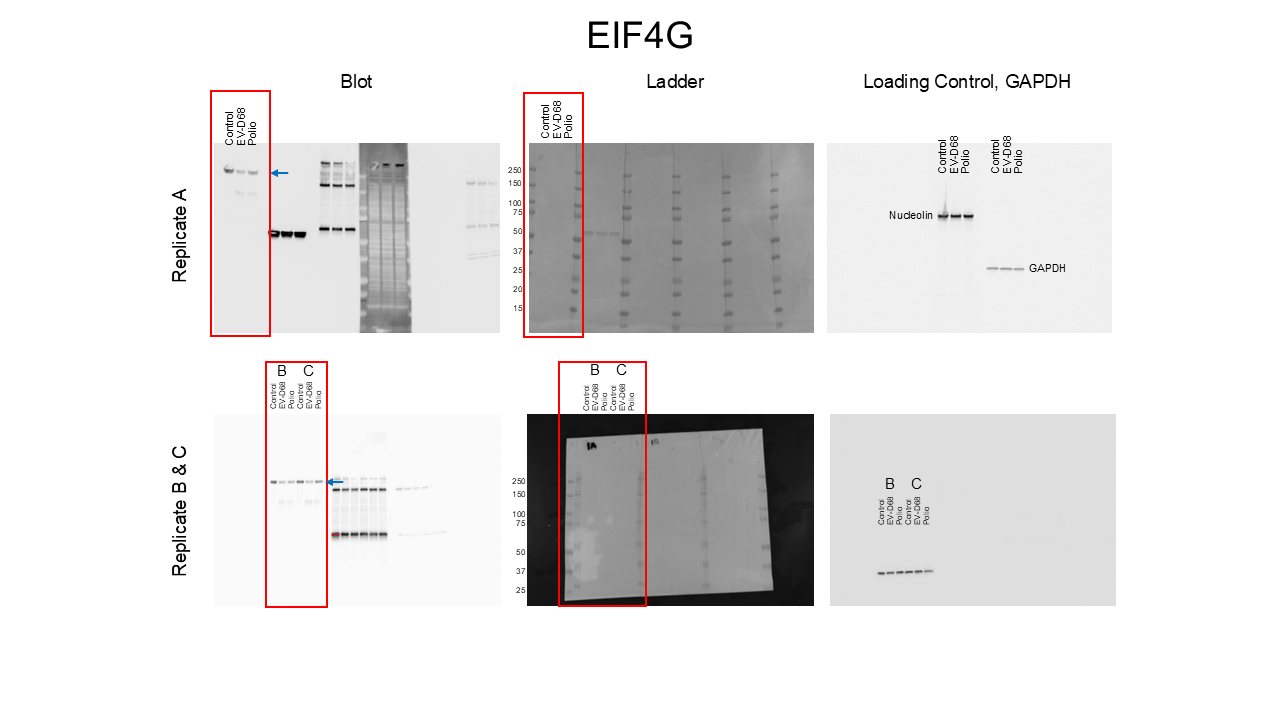

Supplement: Figure 1—source data 2. [file elife-108672-fig1-data2.zip › Figure 1A western blots/EIF4G.TIF]

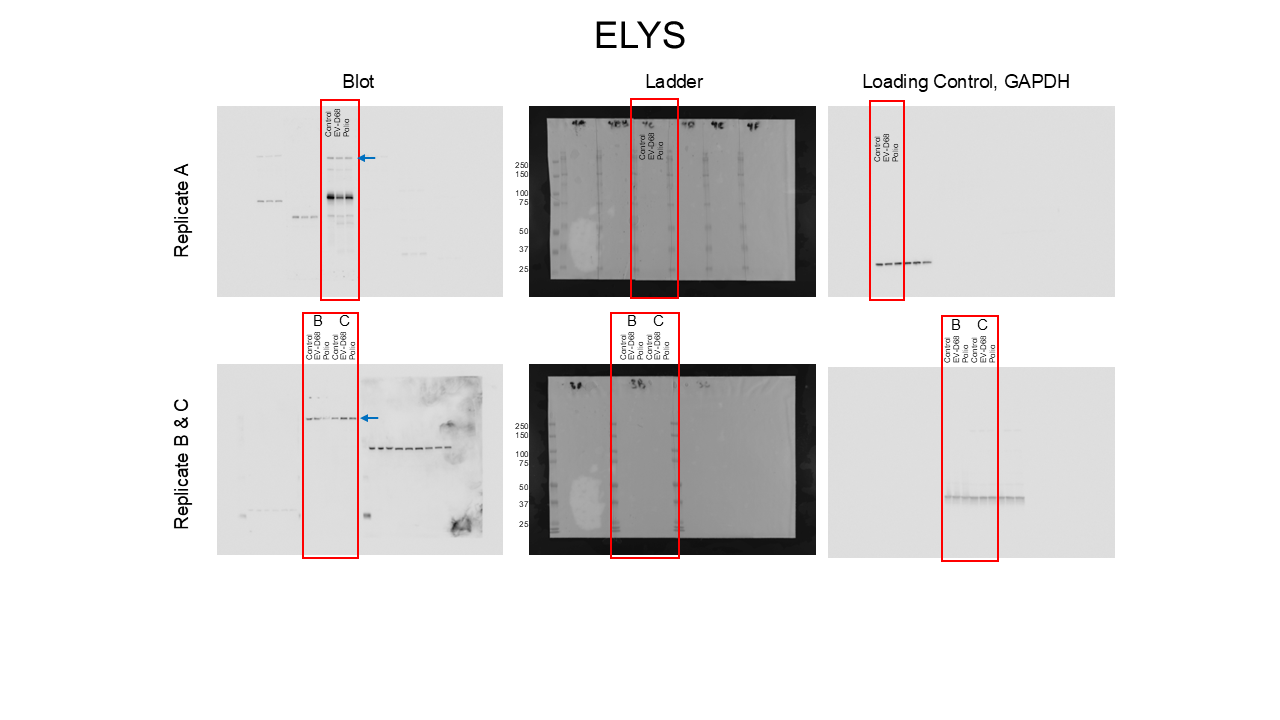

Supplement: Figure 1—source data 2. [file elife-108672-fig1-data2.zip › Figure 1A western blots/ELYS.TIF]

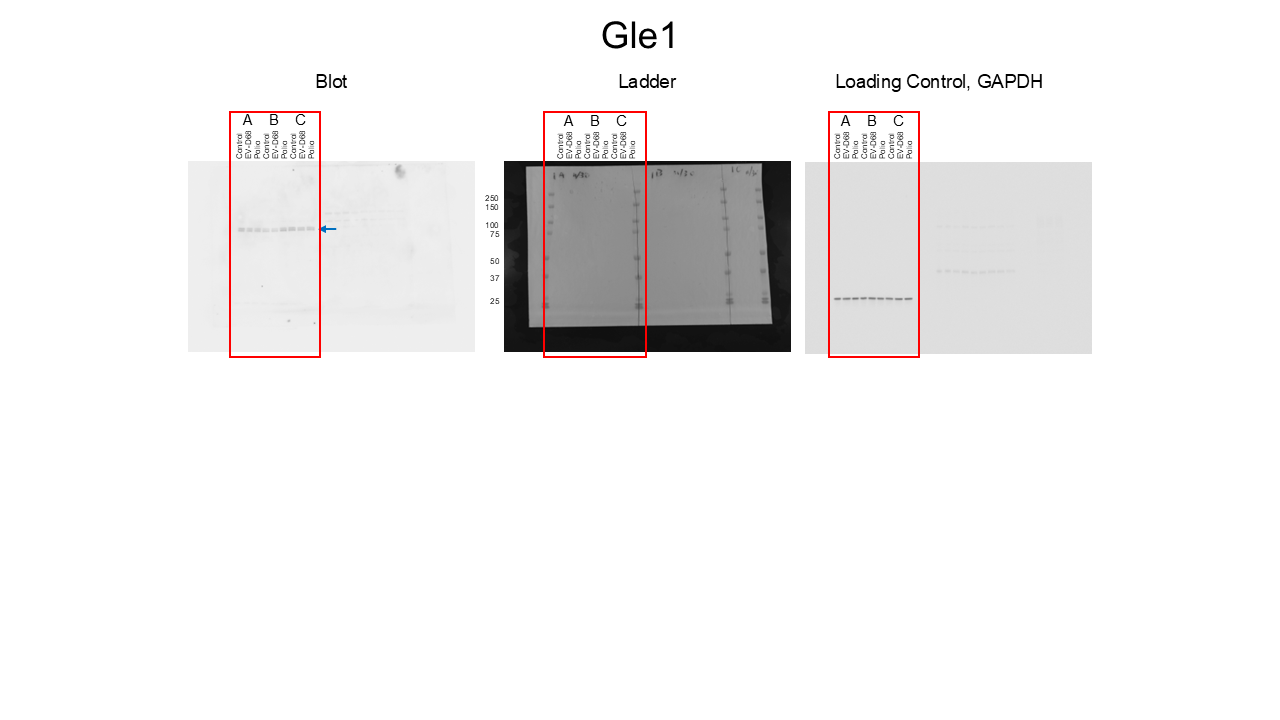

Supplement: Figure 1—source data 2. [file elife-108672-fig1-data2.zip › Figure 1A western blots/Gle1.TIF]

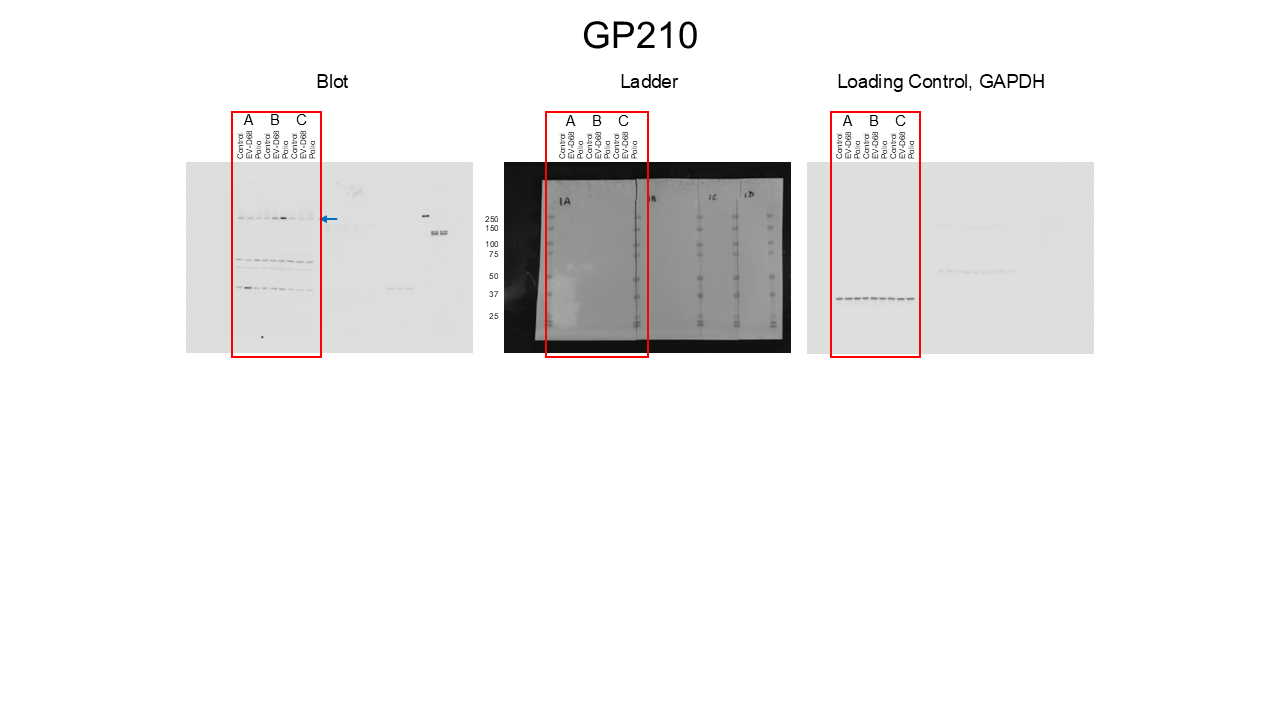

Supplement: Figure 1—source data 2. [file elife-108672-fig1-data2.zip › Figure 1A western blots/GP210.TIF]

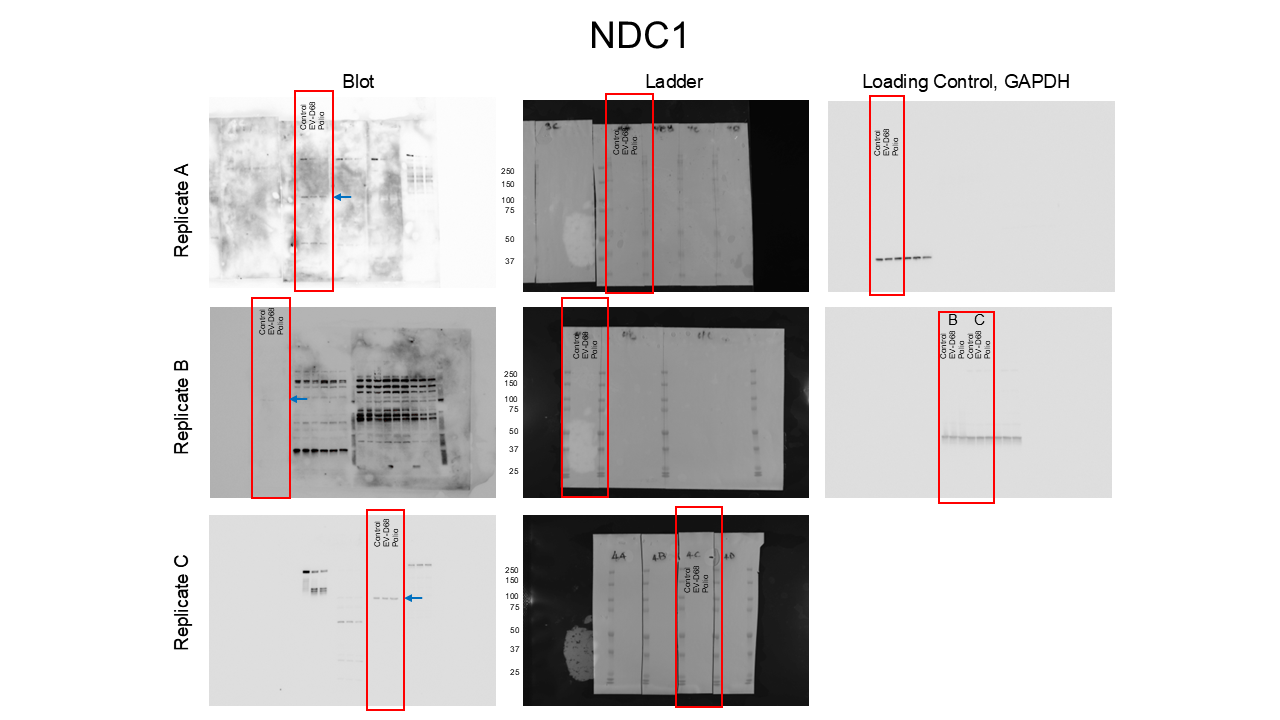

Supplement: Figure 1—source data 2. [file elife-108672-fig1-data2.zip › Figure 1A western blots/NDC1.tif]

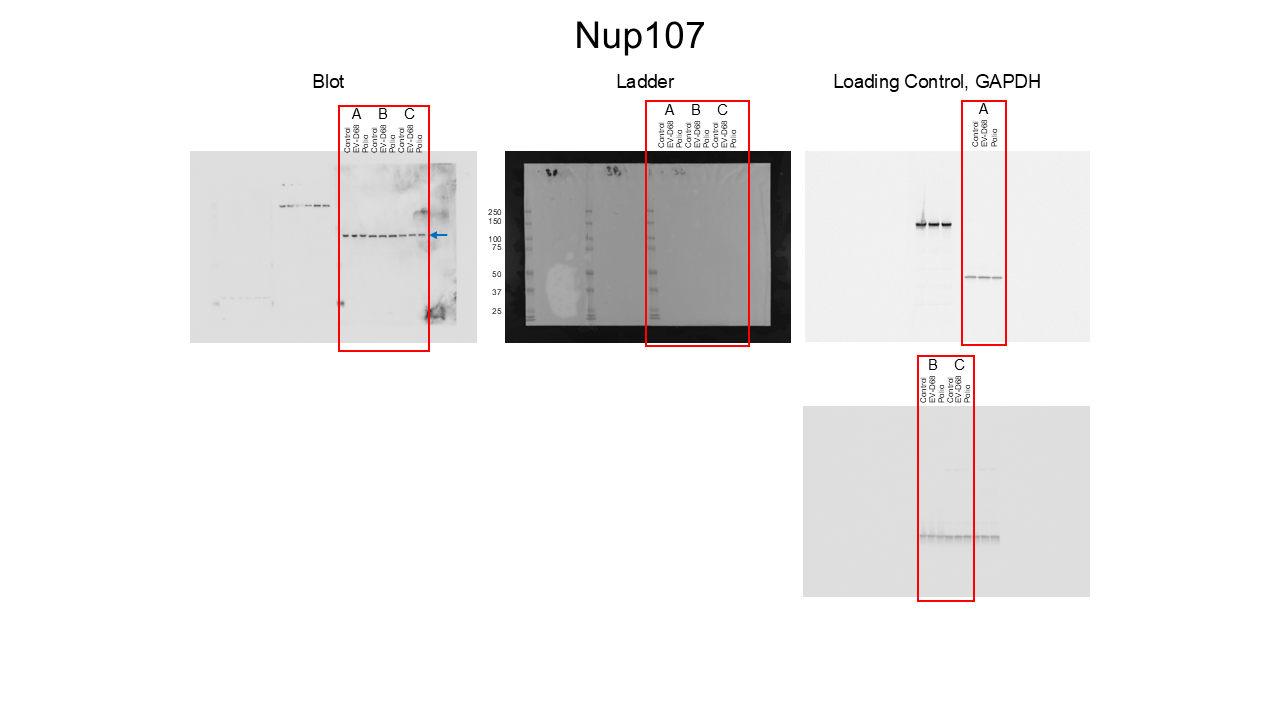

Supplement: Figure 1—source data 2. [file elife-108672-fig1-data2.zip › Figure 1A western blots/Nup107.TIF]

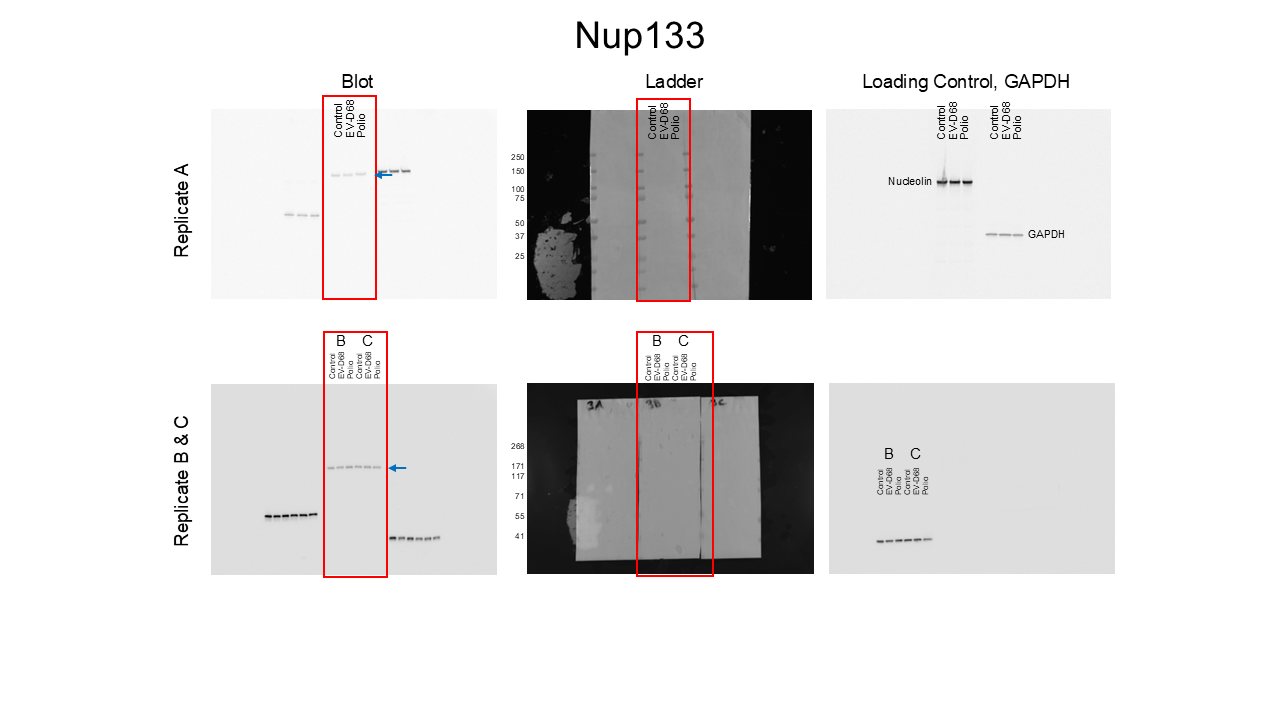

Supplement: Figure 1—source data 2. [file elife-108672-fig1-data2.zip › Figure 1A western blots/Nup133.TIF]

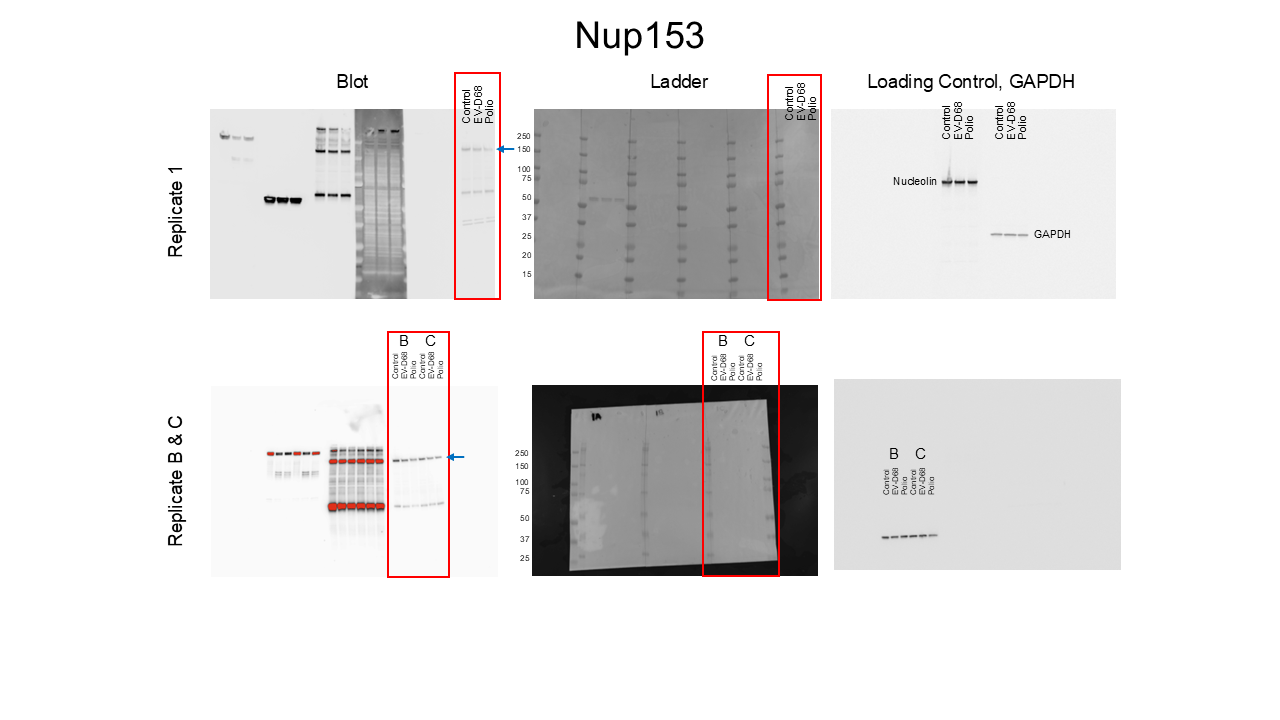

Supplement: Figure 1—source data 2. [file elife-108672-fig1-data2.zip › Figure 1A western blots/Nup153.TIF]

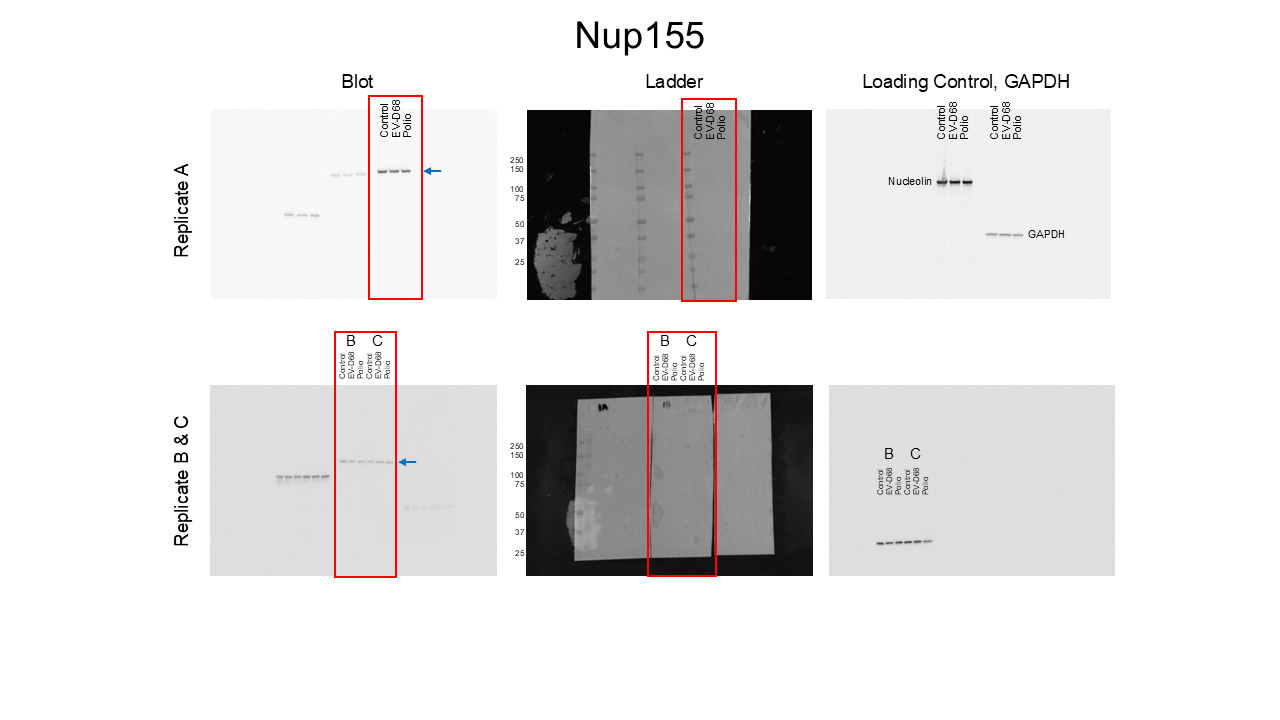

Supplement: Figure 1—source data 2. [file elife-108672-fig1-data2.zip › Figure 1A western blots/Nup155.TIF]

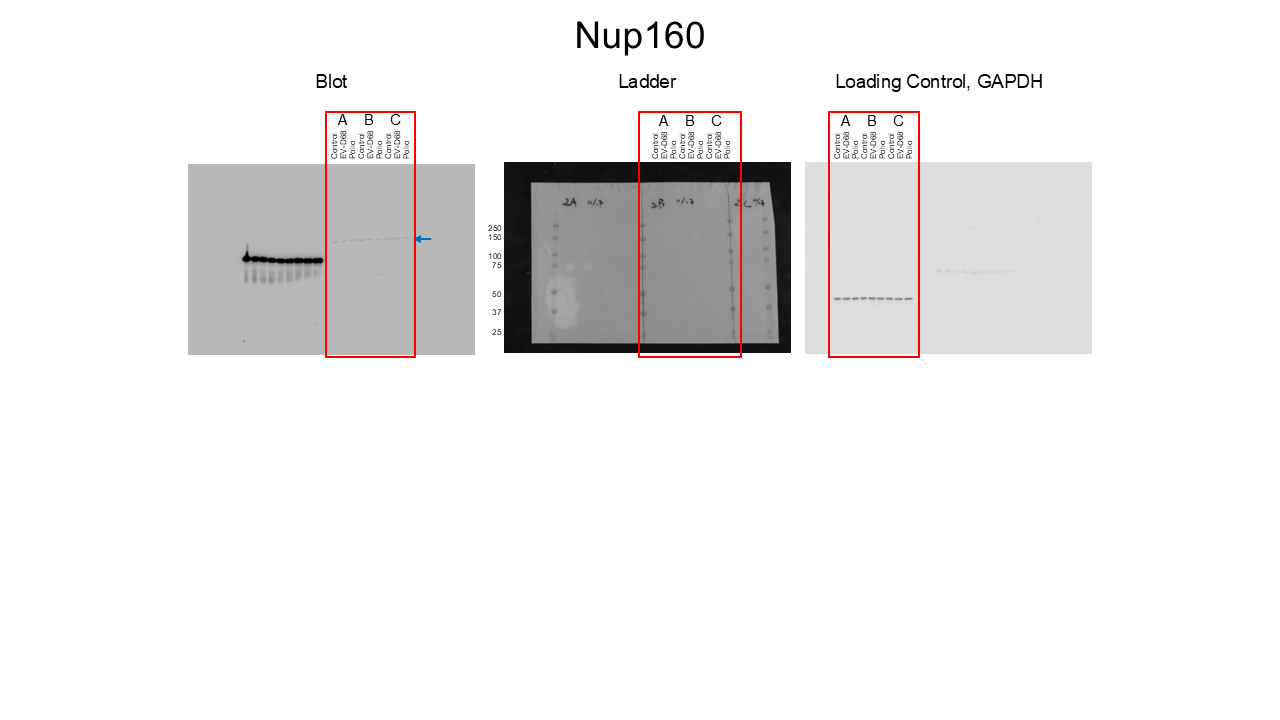

Supplement: Figure 1—source data 2. [file elife-108672-fig1-data2.zip › Figure 1A western blots/Nup160.TIF]

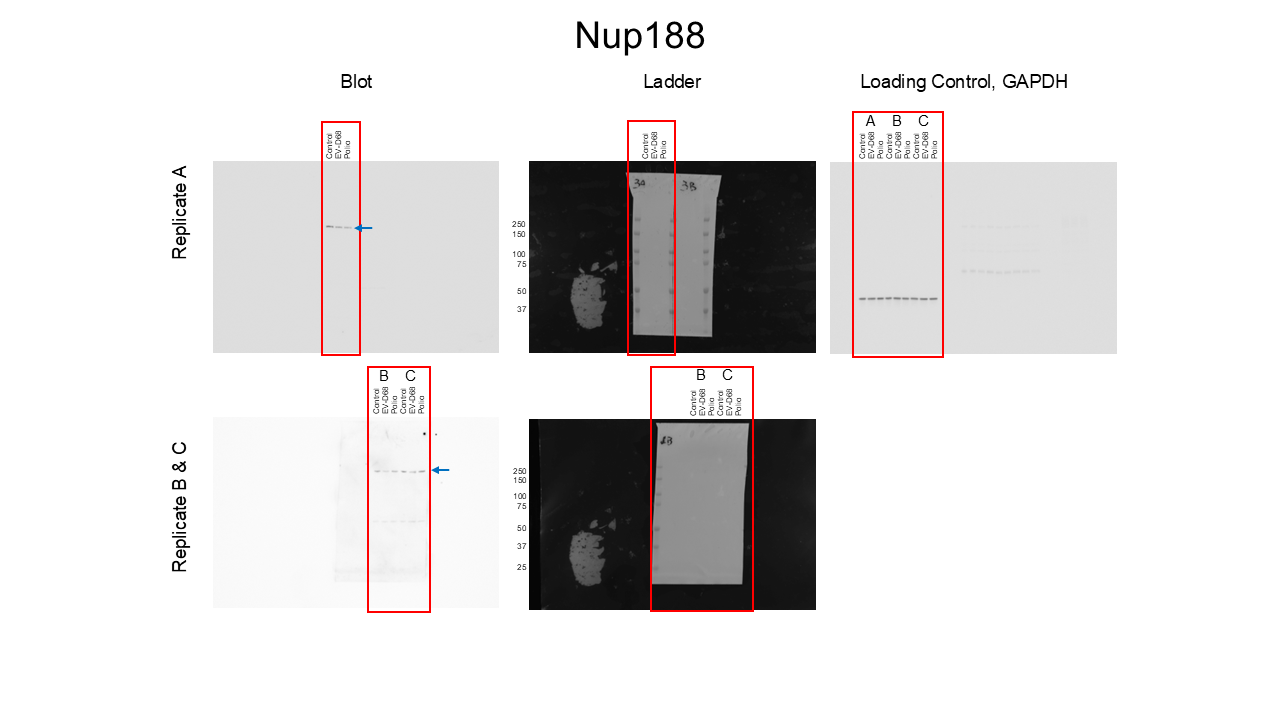

Supplement: Figure 1—source data 2. [file elife-108672-fig1-data2.zip › Figure 1A western blots/Nup188.TIF]

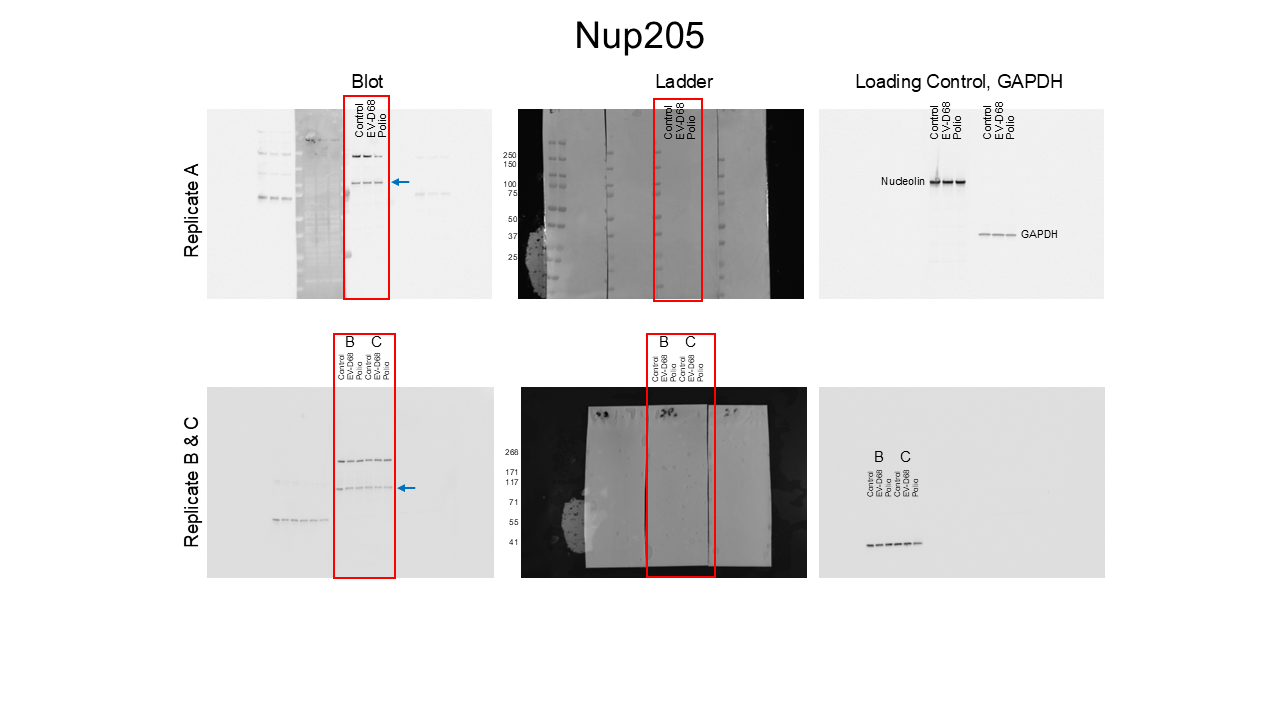

Supplement: Figure 1—source data 2. [file elife-108672-fig1-data2.zip › Figure 1A western blots/Nup205.TIF]

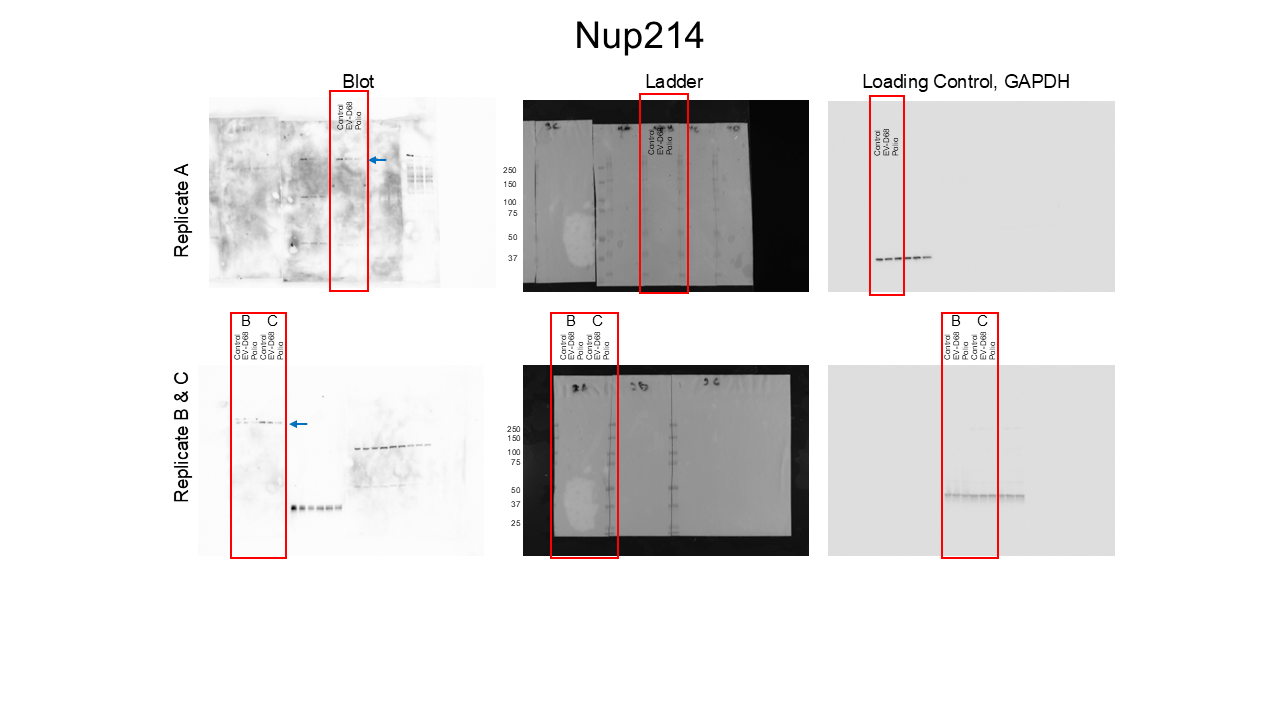

Supplement: Figure 1—source data 2. [file elife-108672-fig1-data2.zip › Figure 1A western blots/Nup214.TIF]

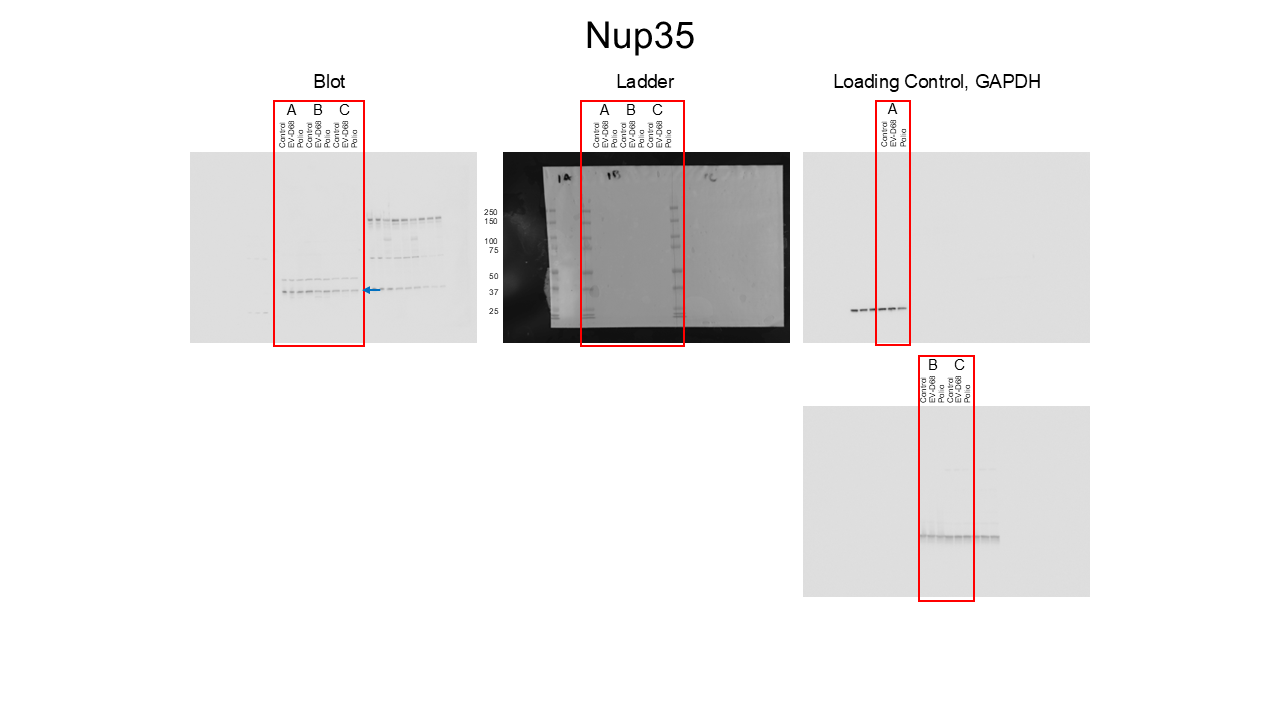

Supplement: Figure 1—source data 2. [file elife-108672-fig1-data2.zip › Figure 1A western blots/Nup35.TIF]

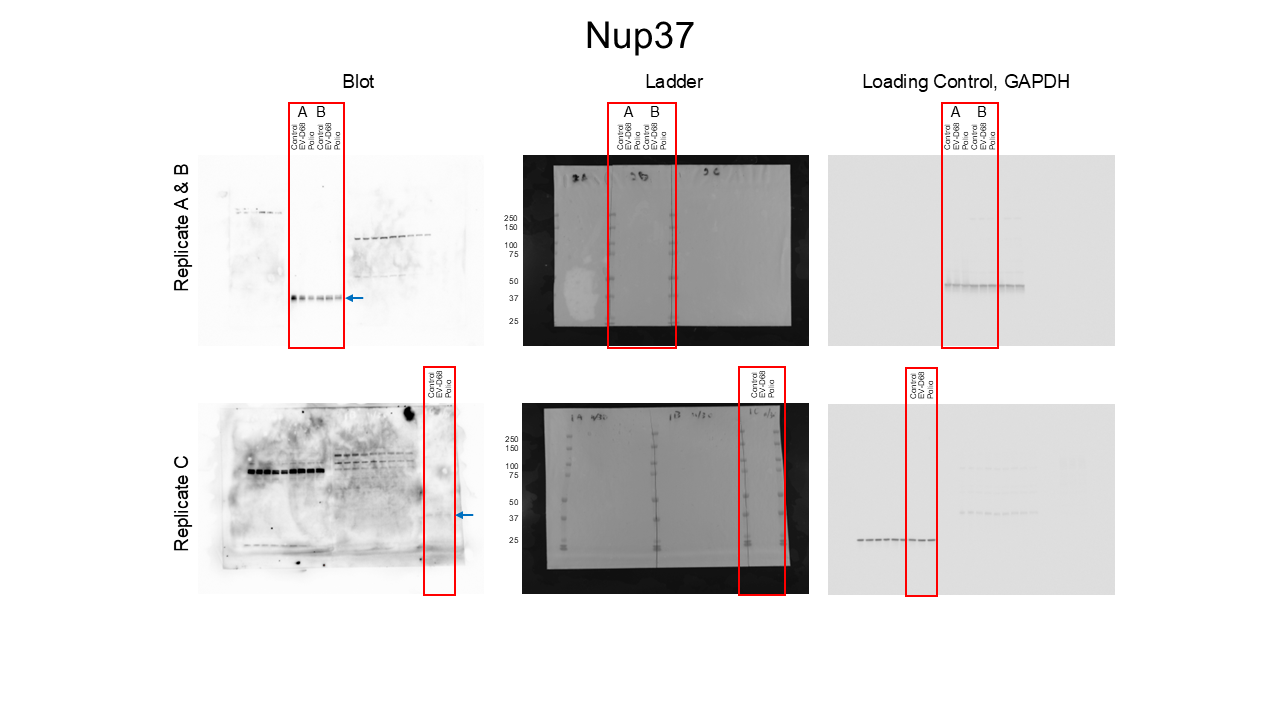

Supplement: Figure 1—source data 2. [file elife-108672-fig1-data2.zip › Figure 1A western blots/Nup37.TIF]

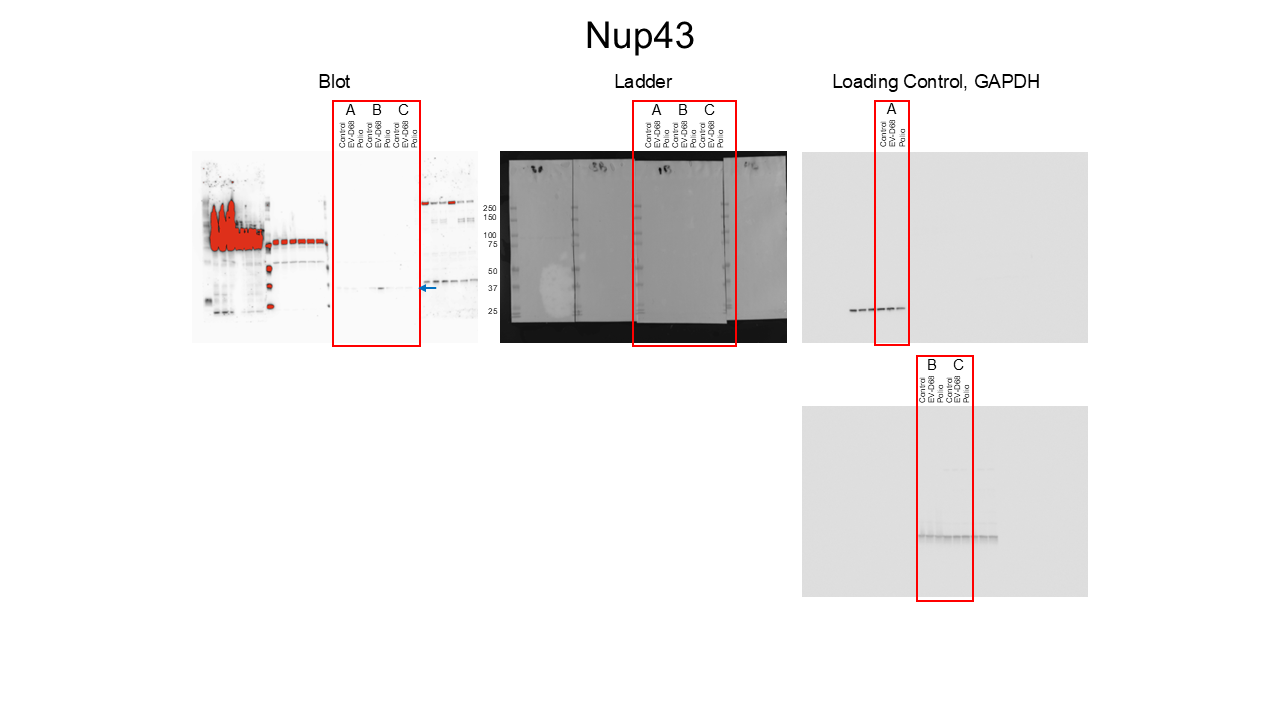

Supplement: Figure 1—source data 2. [file elife-108672-fig1-data2.zip › Figure 1A western blots/Nup43.TIF]

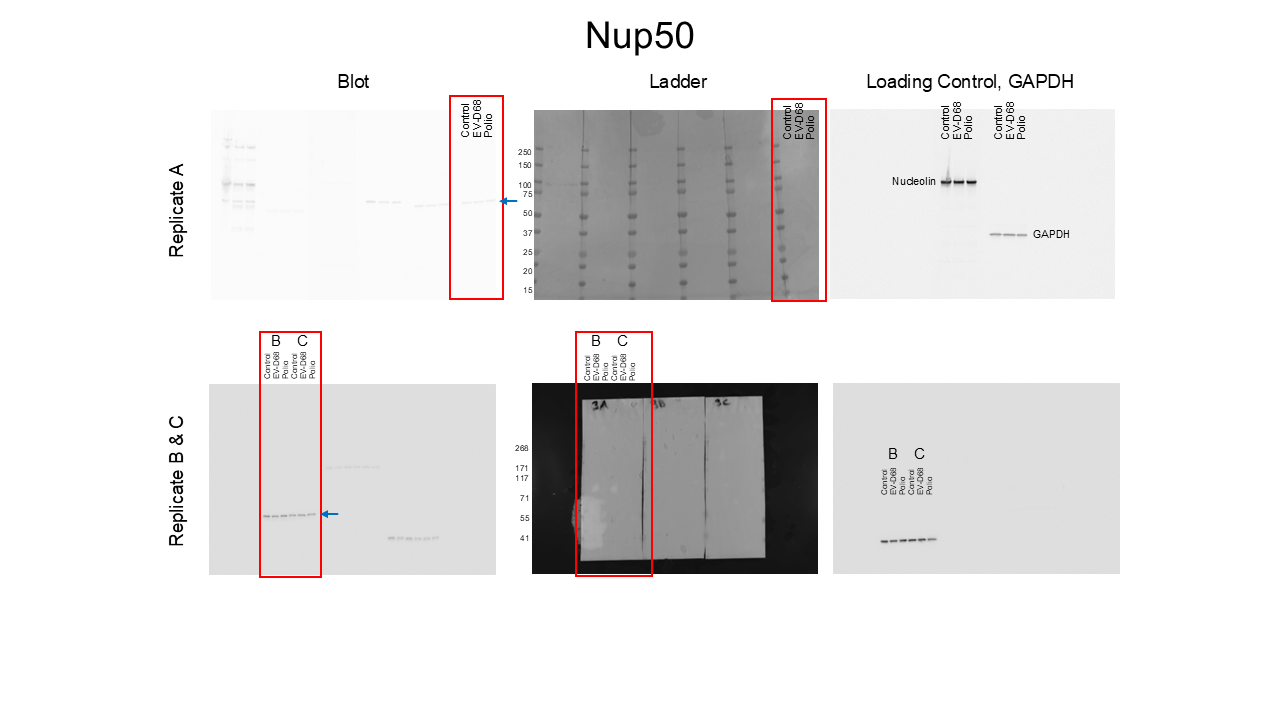

Supplement: Figure 1—source data 2. [file elife-108672-fig1-data2.zip › Figure 1A western blots/Nup50.TIF]

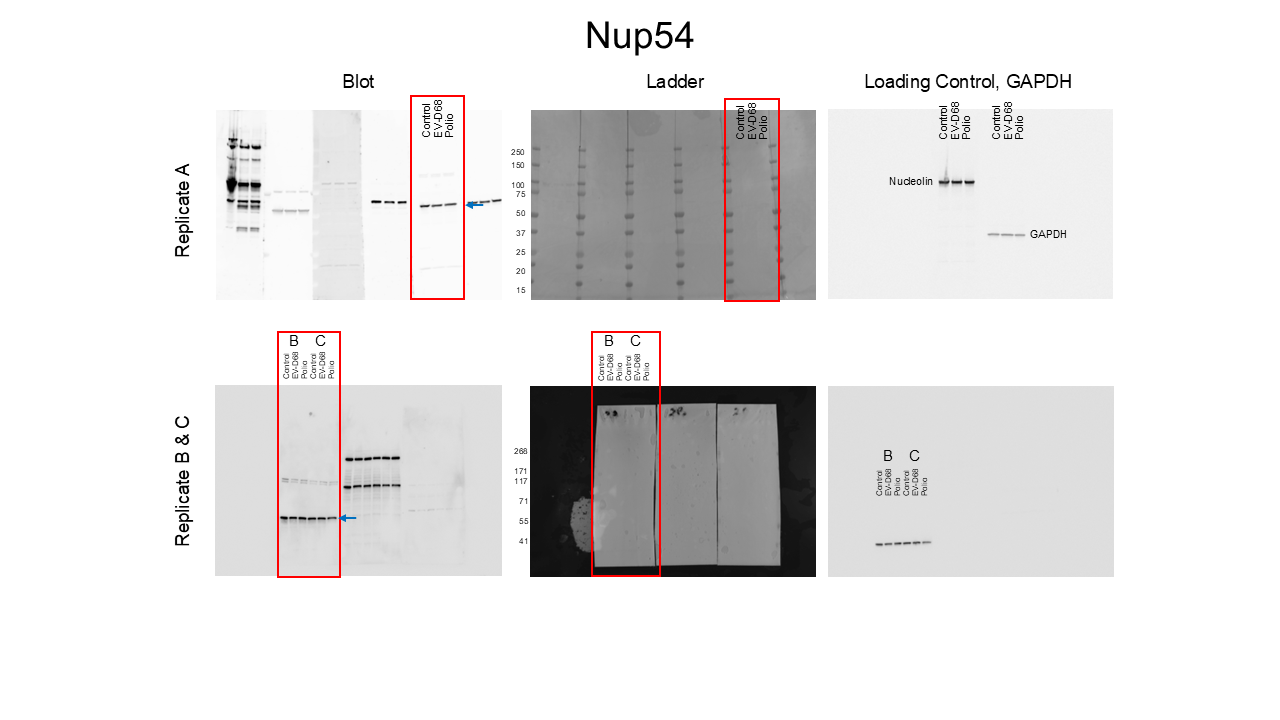

Supplement: Figure 1—source data 2. [file elife-108672-fig1-data2.zip › Figure 1A western blots/Nup54.TIF]

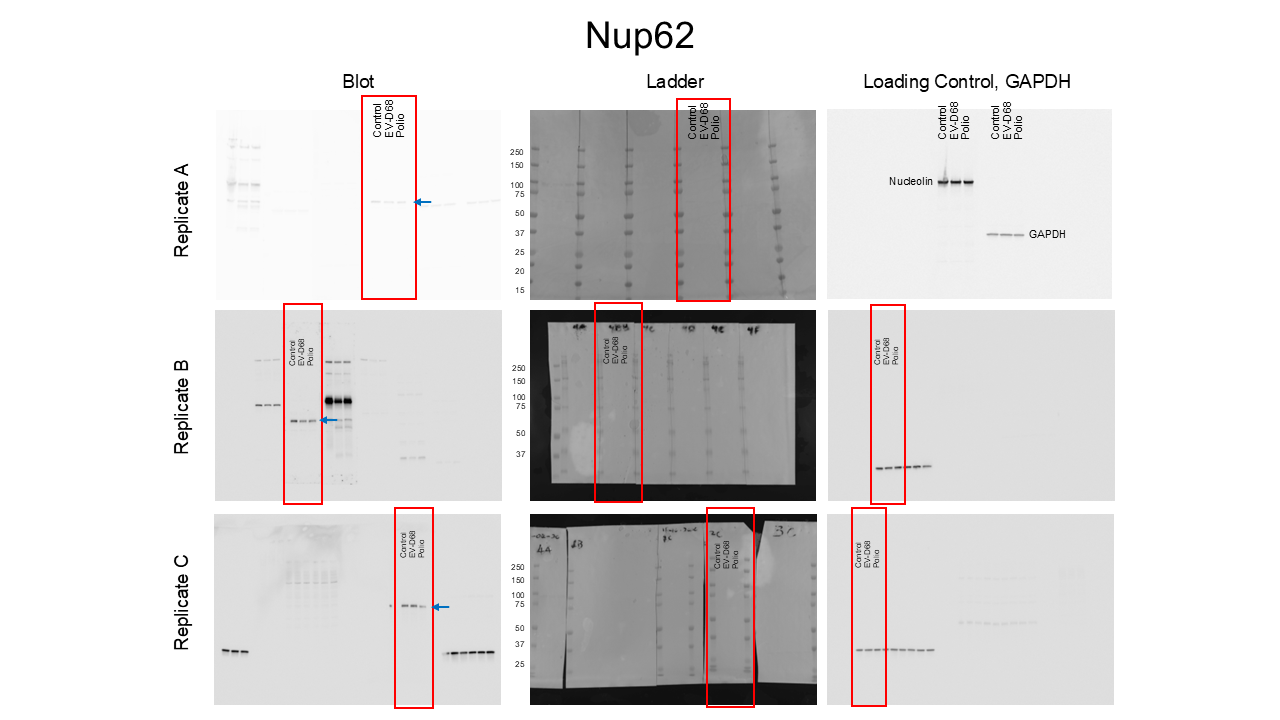

Supplement: Figure 1—source data 2. [file elife-108672-fig1-data2.zip › Figure 1A western blots/Nup62.TIF]

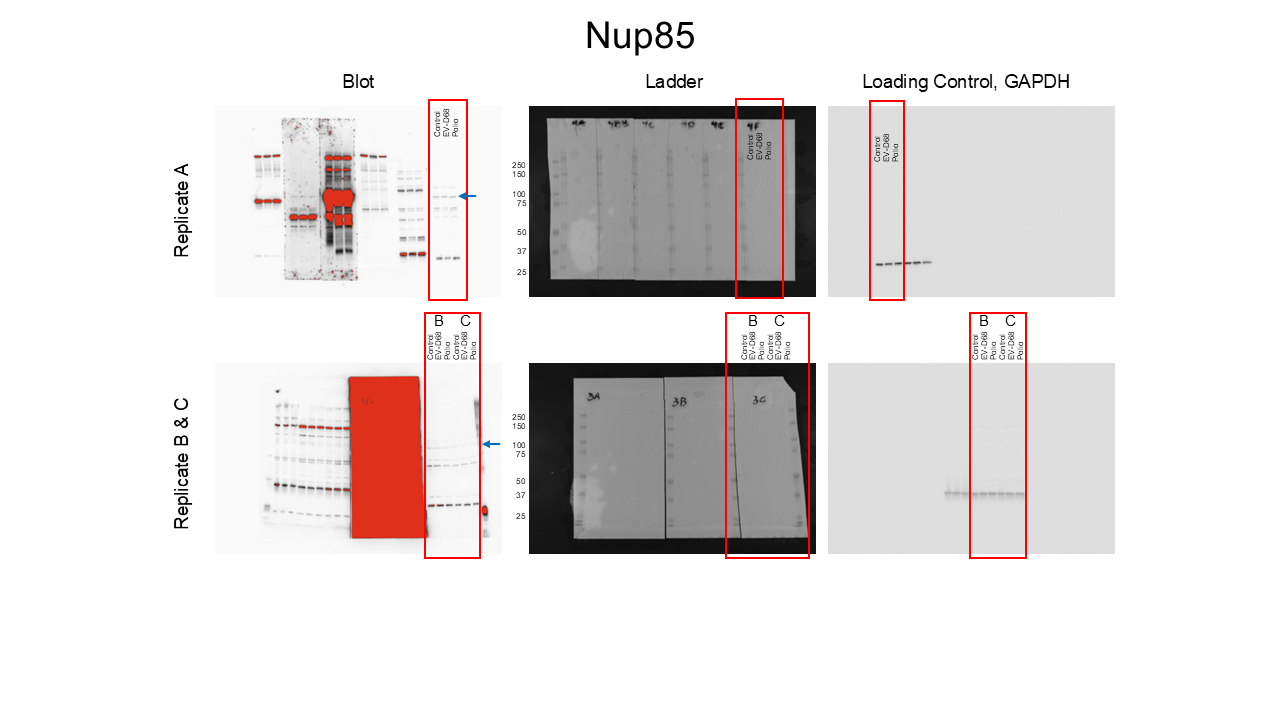

Supplement: Figure 1—source data 2. [file elife-108672-fig1-data2.zip › Figure 1A western blots/Nup85.TIF]

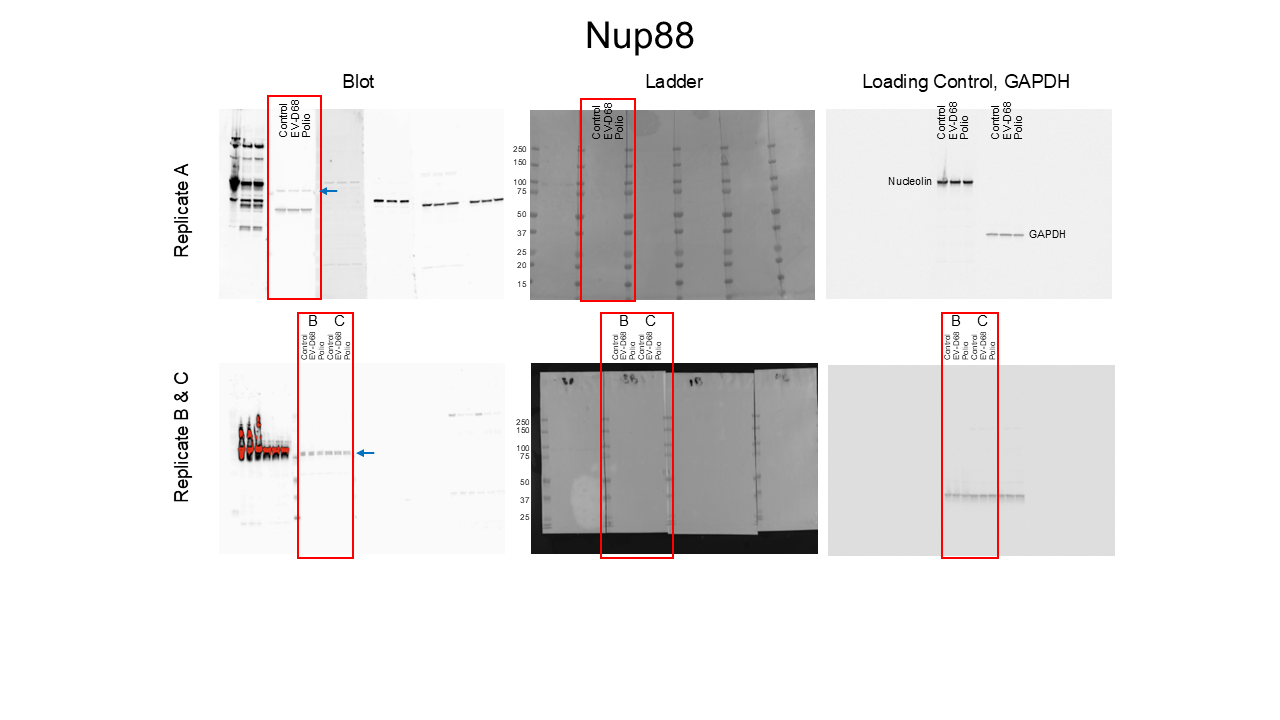

Supplement: Figure 1—source data 2. [file elife-108672-fig1-data2.zip › Figure 1A western blots/Nup88.TIF]

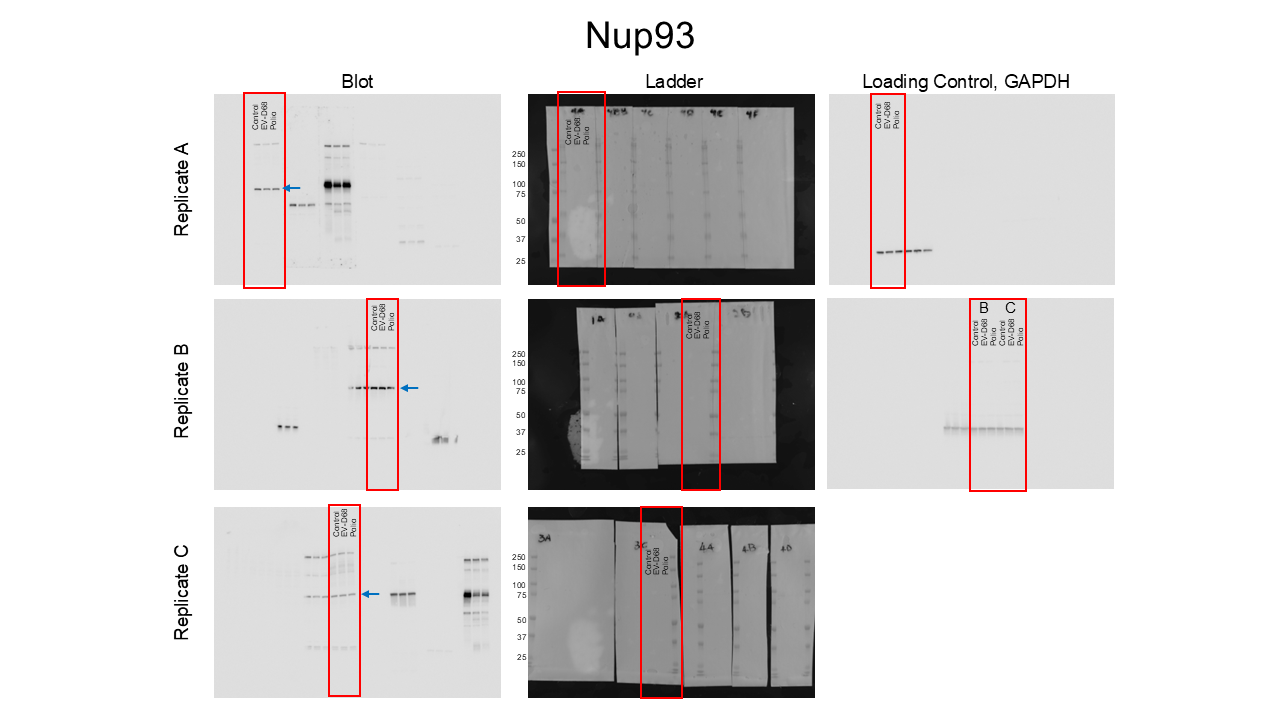

Supplement: Figure 1—source data 2. [file elife-108672-fig1-data2.zip › Figure 1A western blots/Nup93.TIF]

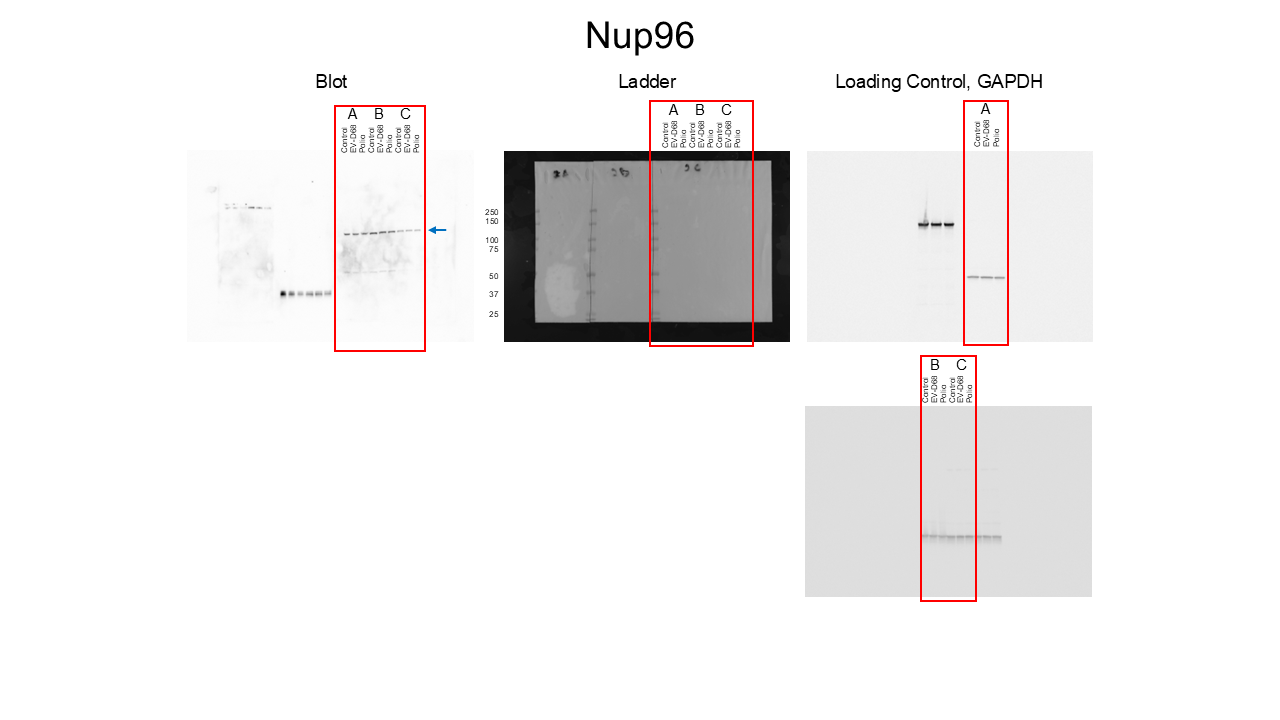

Supplement: Figure 1—source data 2. [file elife-108672-fig1-data2.zip › Figure 1A western blots/Nup96.TIF]

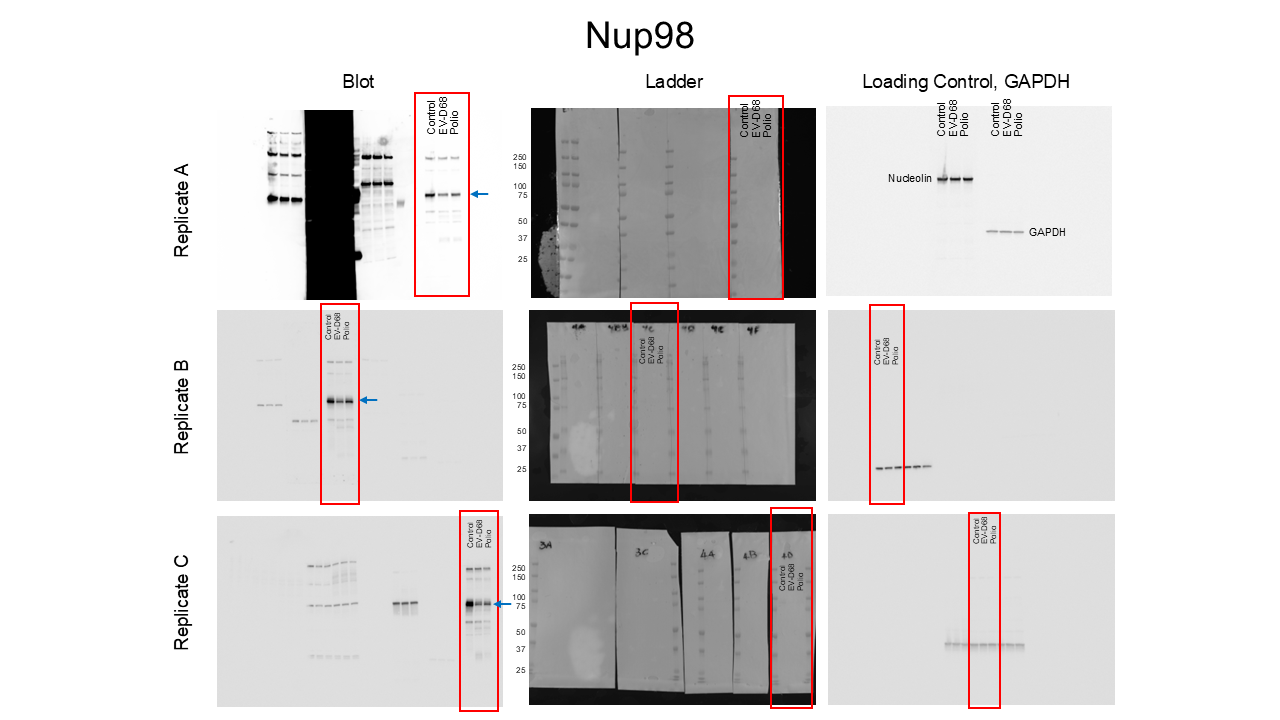

Supplement: Figure 1—source data 2. [file elife-108672-fig1-data2.zip › Figure 1A western blots/Nup98.TIF]

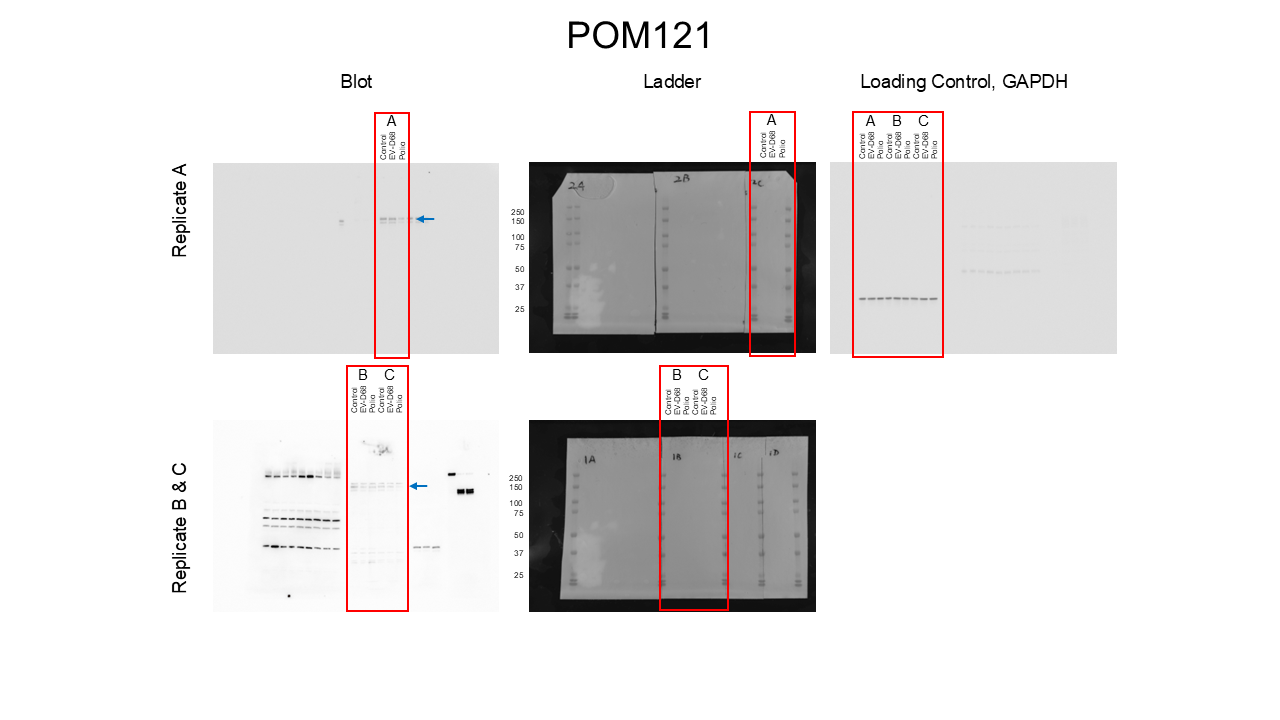

Supplement: Figure 1—source data 2. [file elife-108672-fig1-data2.zip › Figure 1A western blots/POM121.TIF]

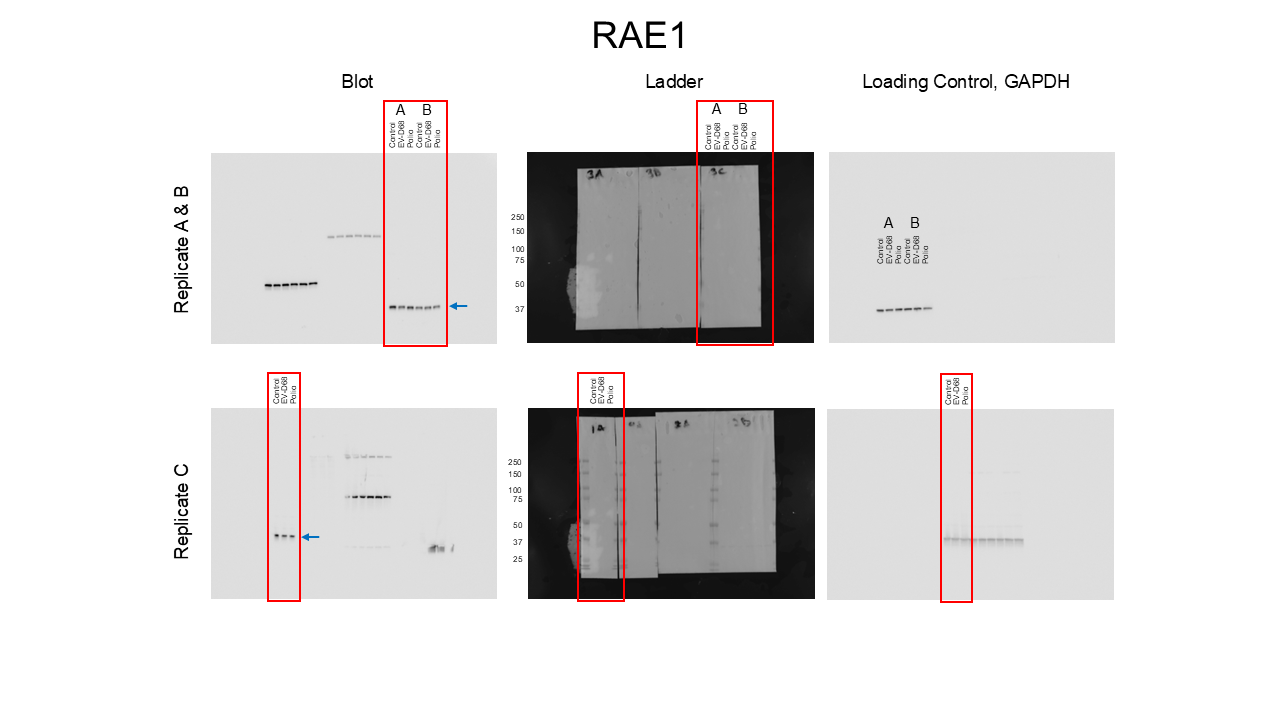

Supplement: Figure 1—source data 2. [file elife-108672-fig1-data2.zip › Figure 1A western blots/RAE1.TIF]

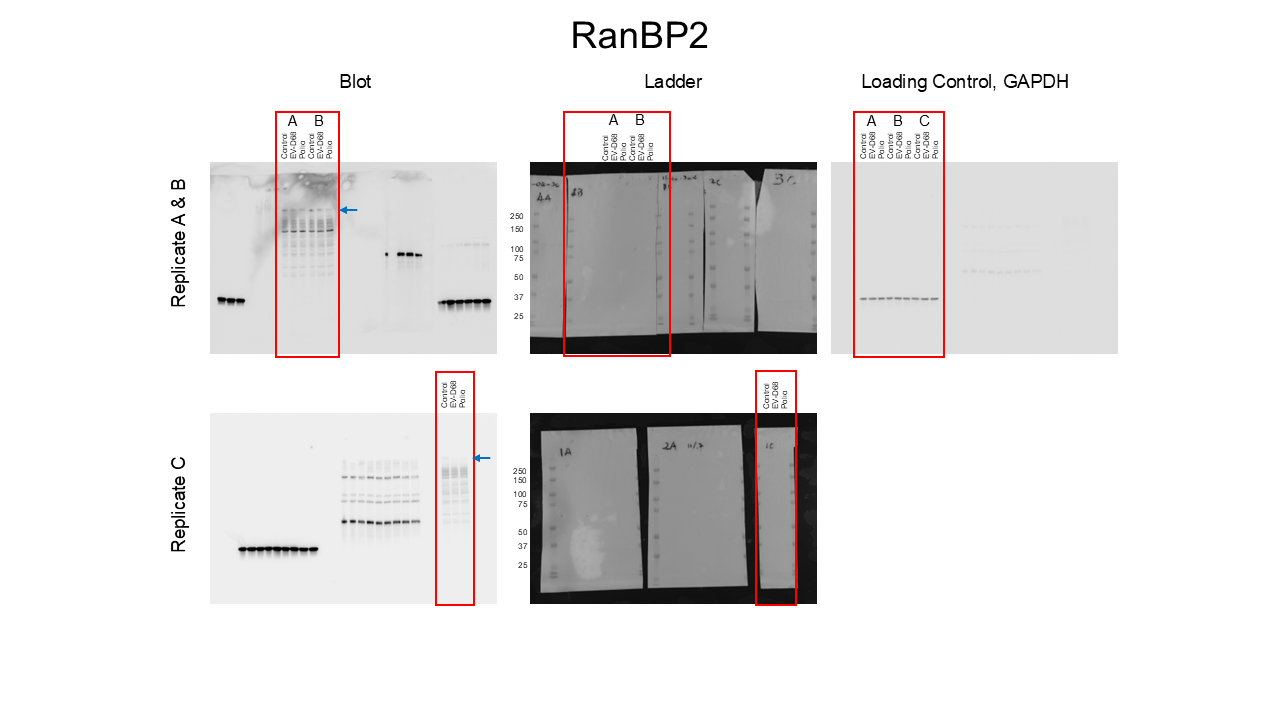

Supplement: Figure 1—source data 2. [file elife-108672-fig1-data2.zip › Figure 1A western blots/RanBP2.TIF]

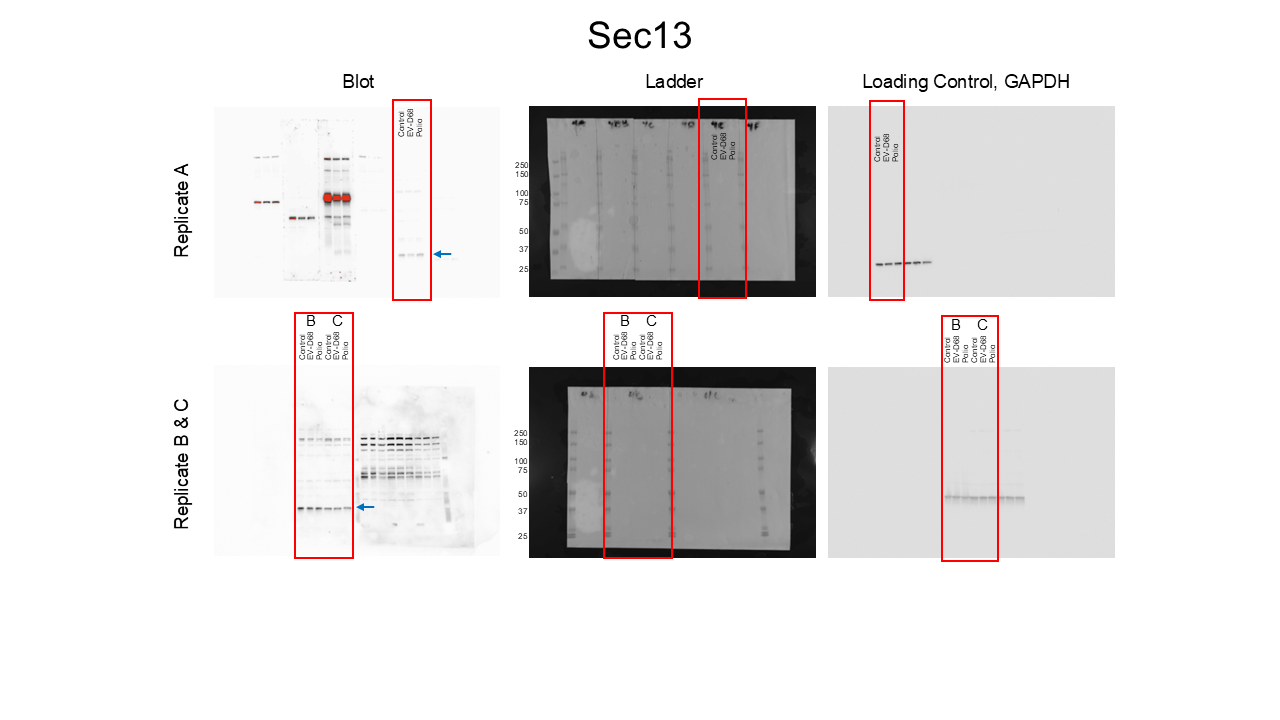

Supplement: Figure 1—source data 2. [file elife-108672-fig1-data2.zip › Figure 1A western blots/Sec13.TIF]

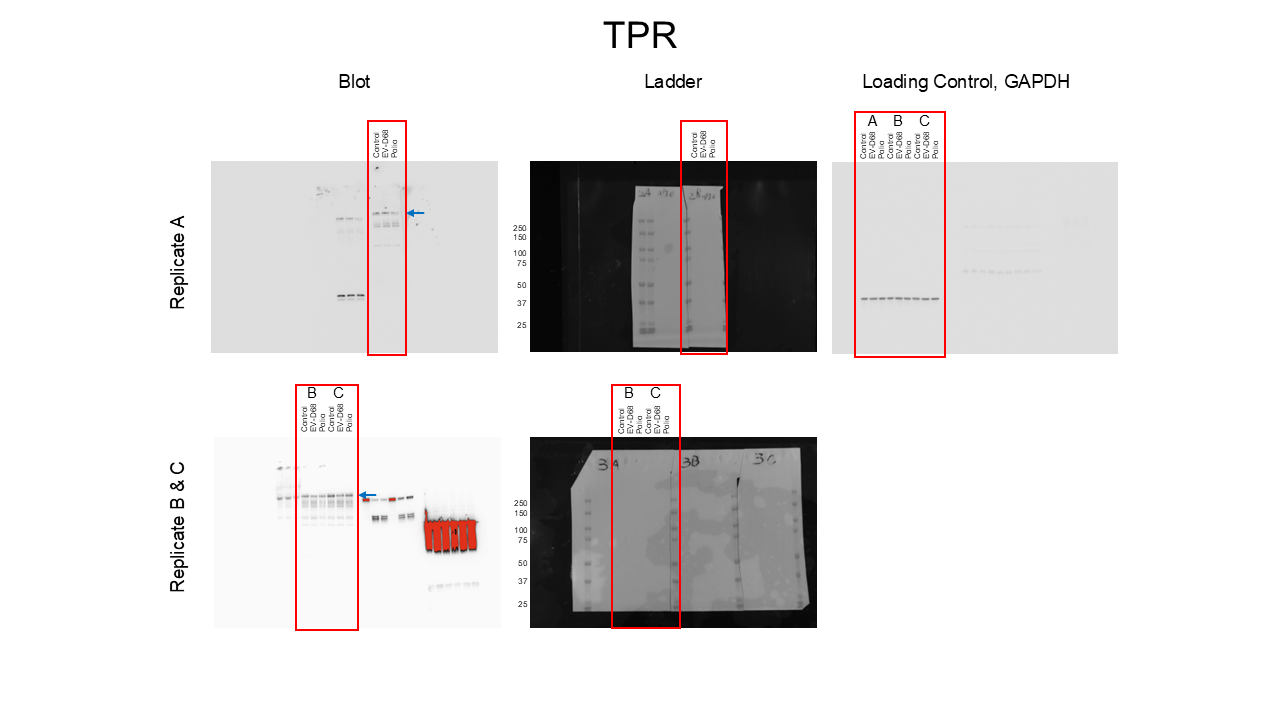

Supplement: Figure 1—source data 2. [file elife-108672-fig1-data2.zip › Figure 1A western blots/TPR.TIF]

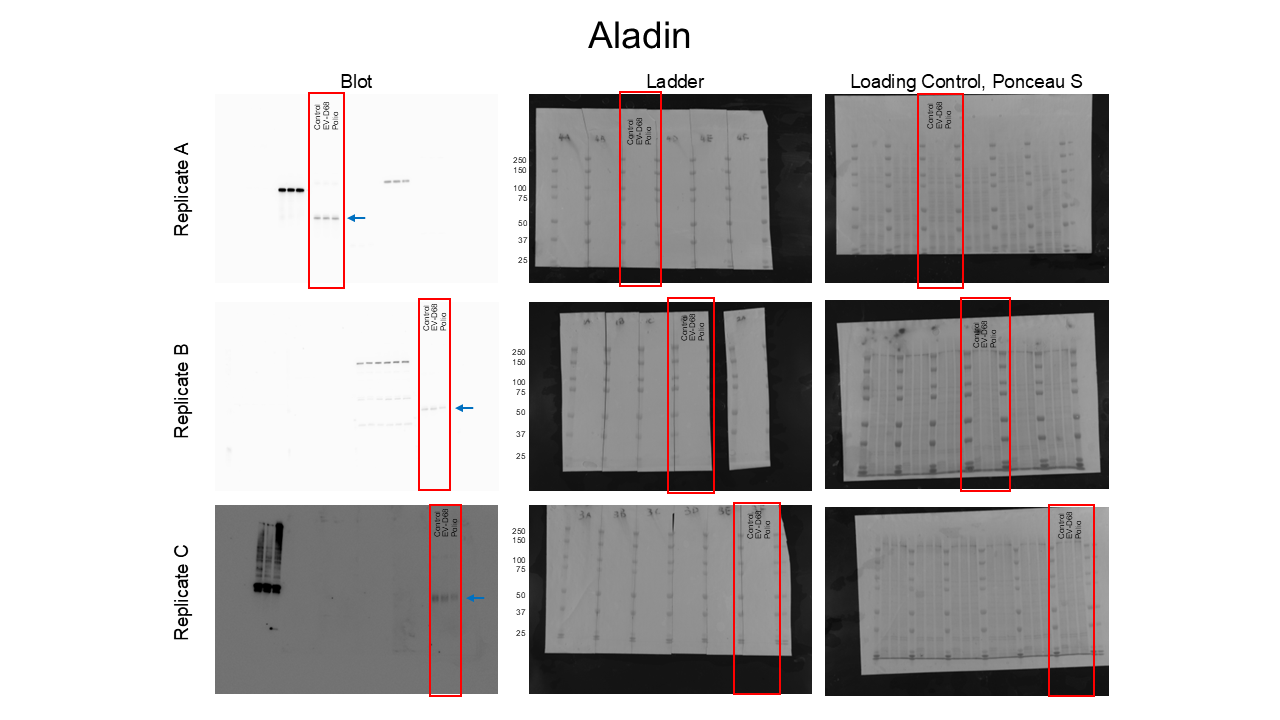

Supplement: Figure 1—source data 2. [file elife-108672-fig1-data2.zip › Figure 1B western blots/Aladin.TIF]

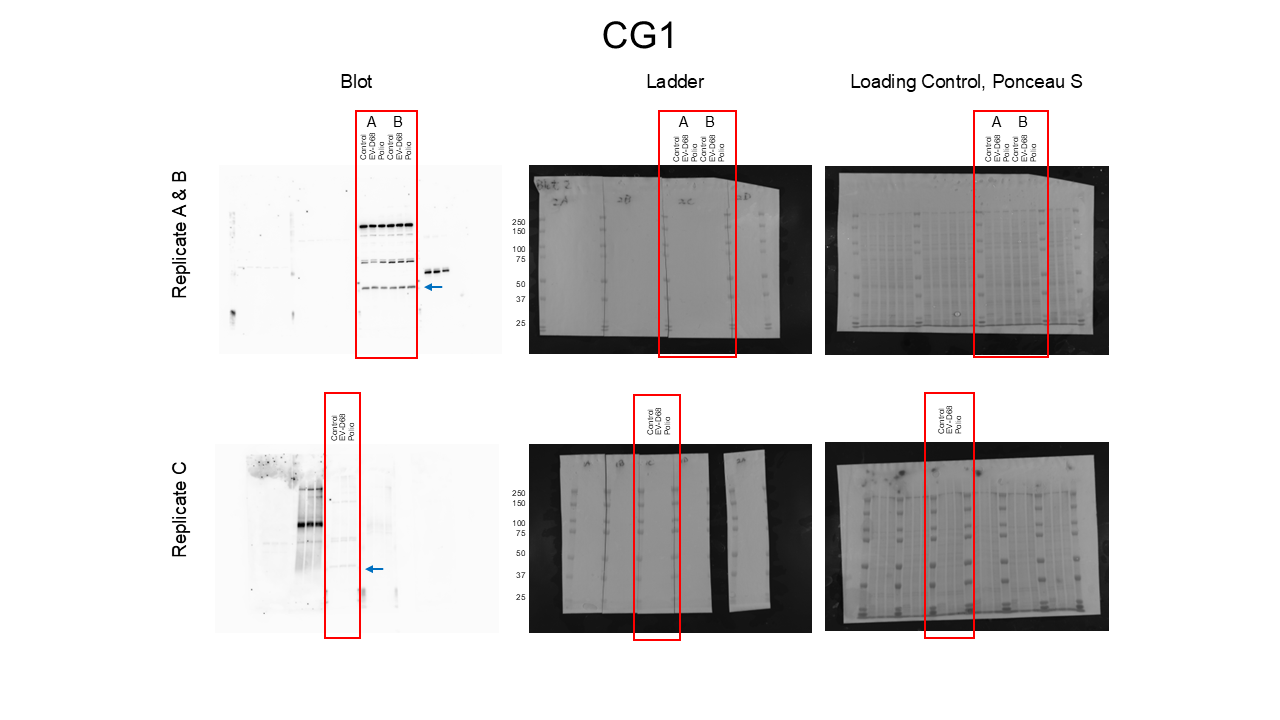

Supplement: Figure 1—source data 2. [file elife-108672-fig1-data2.zip › Figure 1B western blots/CG1.TIF]

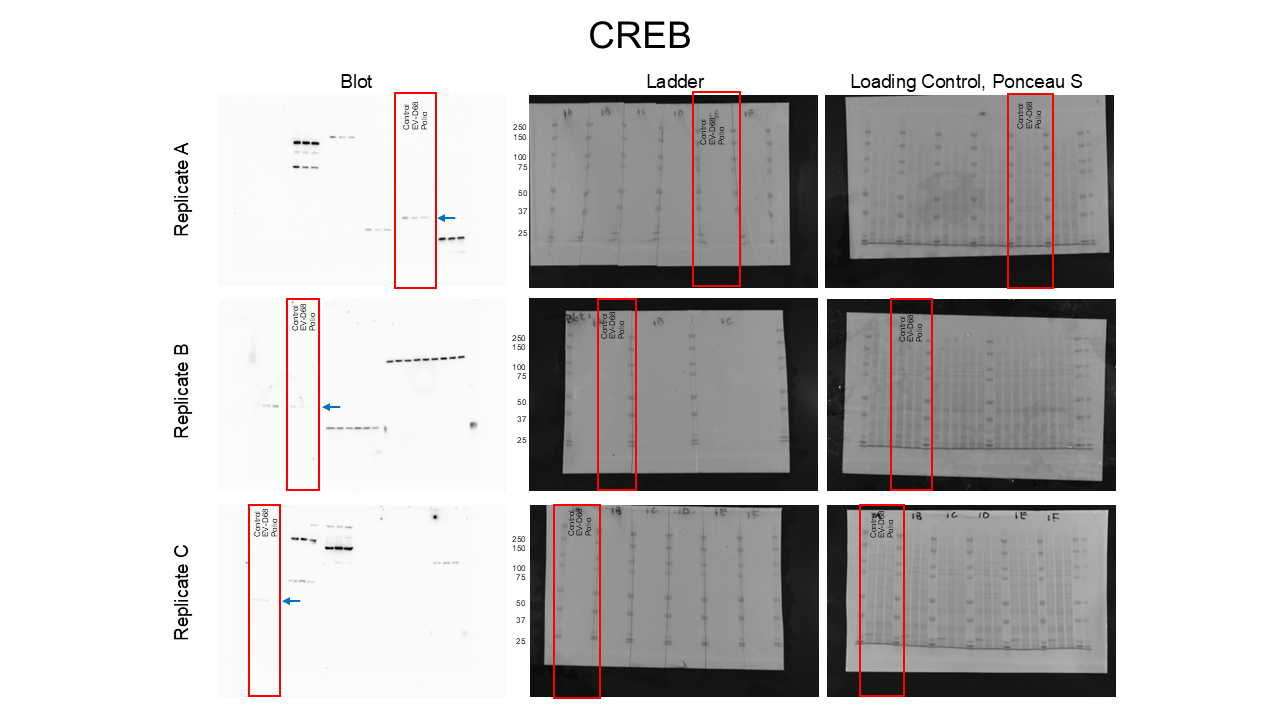

Supplement: Figure 1—source data 2. [file elife-108672-fig1-data2.zip › Figure 1B western blots/CREB.TIF]

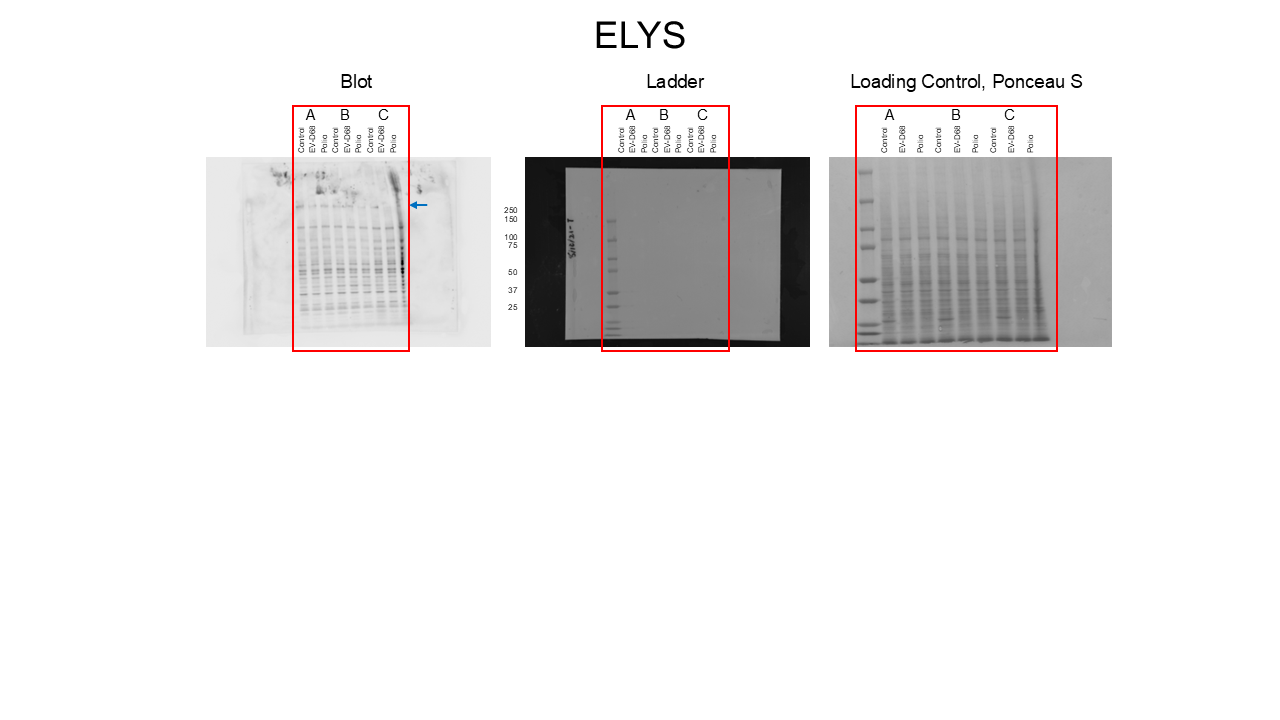

Supplement: Figure 1—source data 2. [file elife-108672-fig1-data2.zip › Figure 1B western blots/ELYS.TIF]

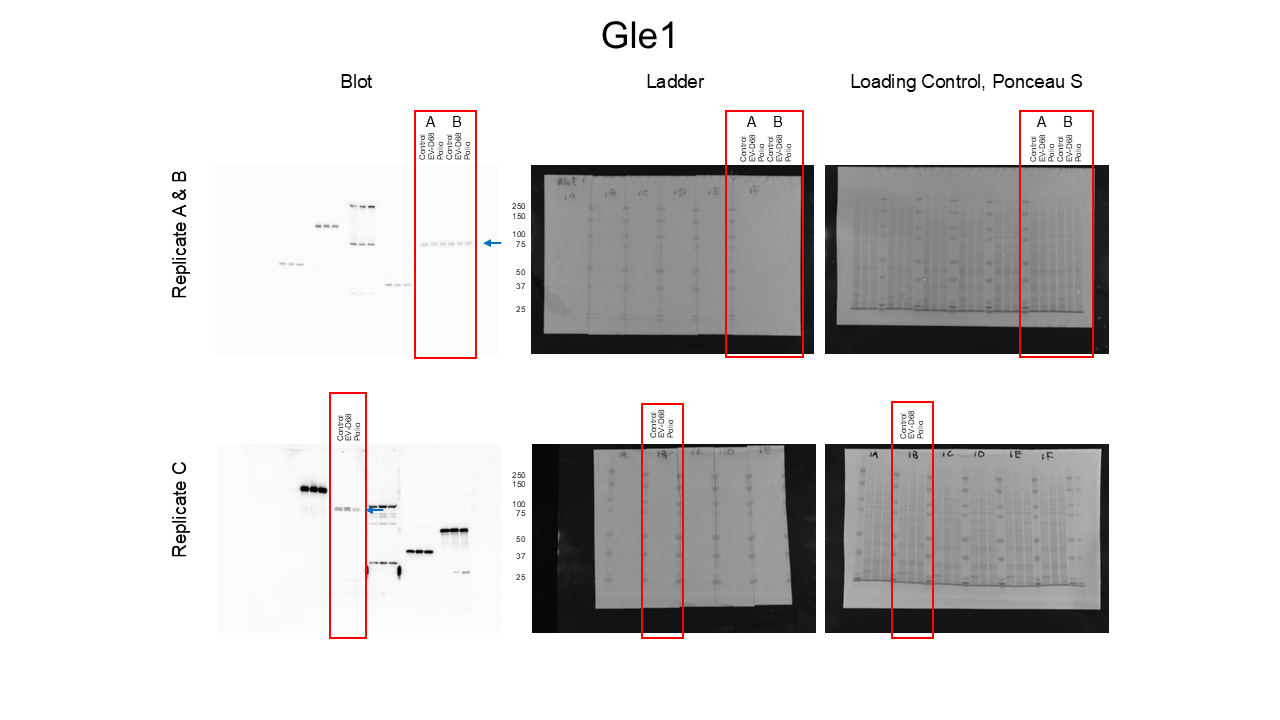

Supplement: Figure 1—source data 2. [file elife-108672-fig1-data2.zip › Figure 1B western blots/Gle1.TIF]

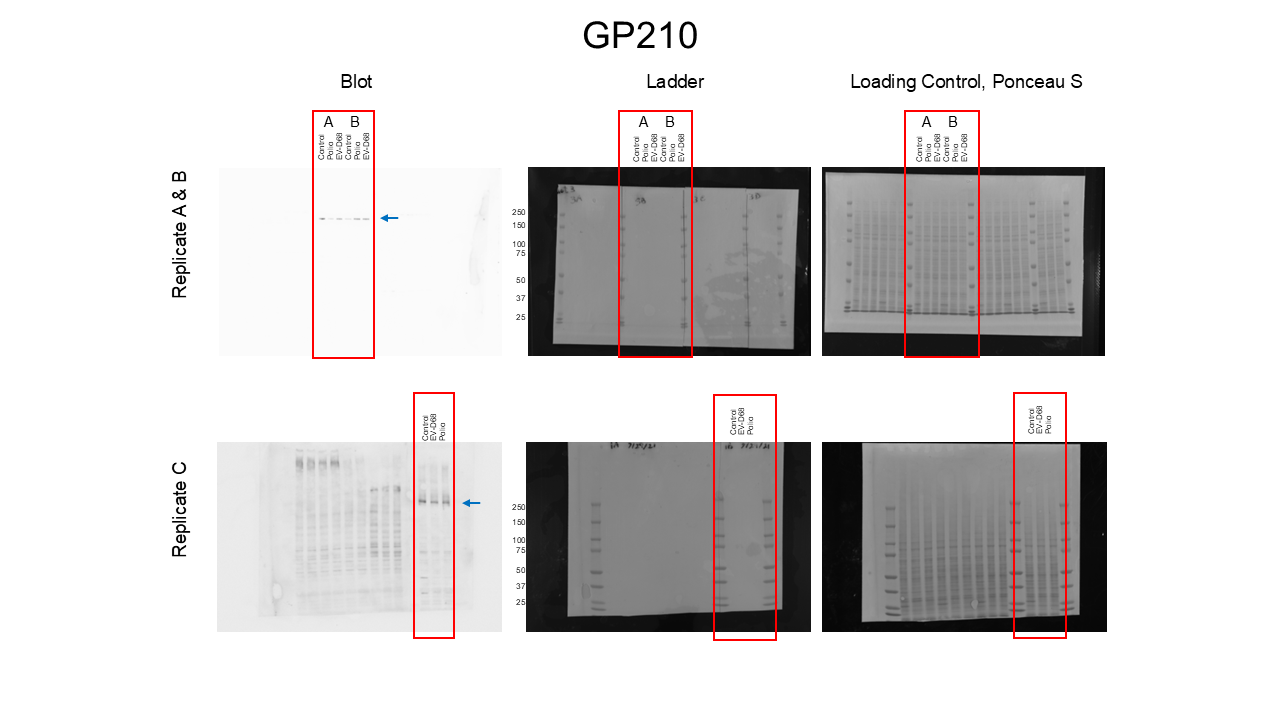

Supplement: Figure 1—source data 2. [file elife-108672-fig1-data2.zip › Figure 1B western blots/GP210.TIF]

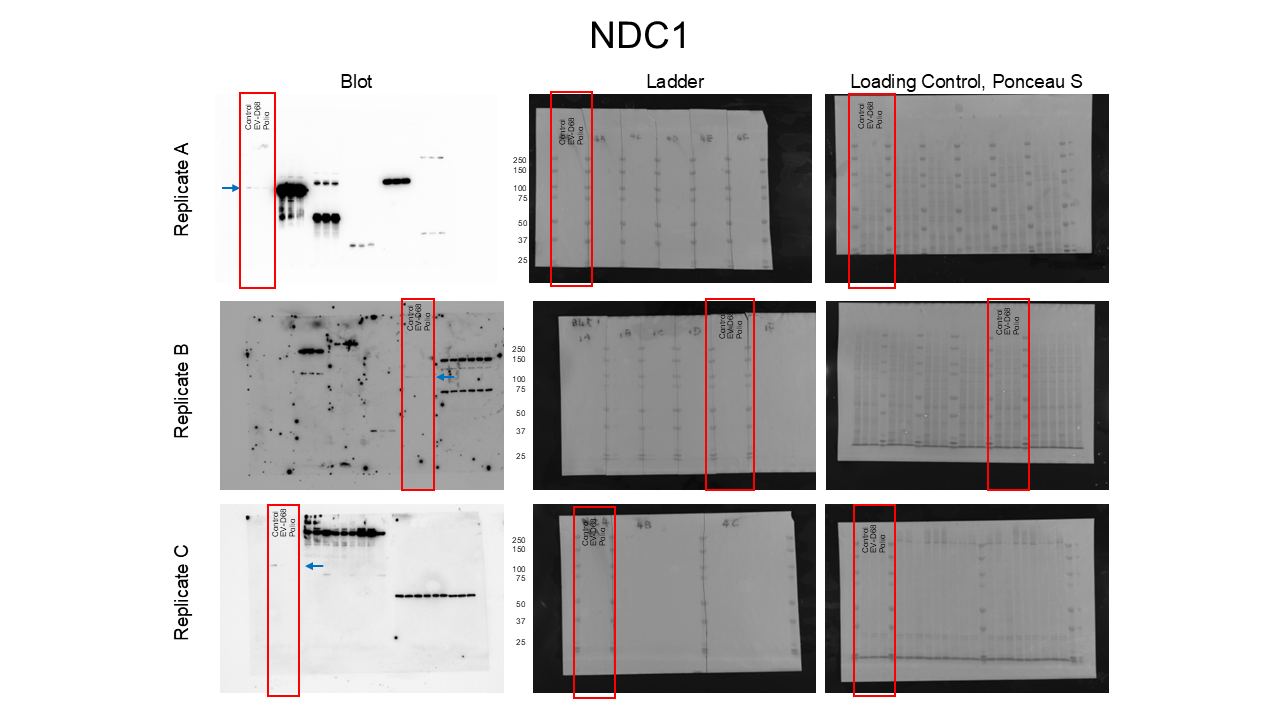

Supplement: Figure 1—source data 2. [file elife-108672-fig1-data2.zip › Figure 1B western blots/NDC1.TIF]

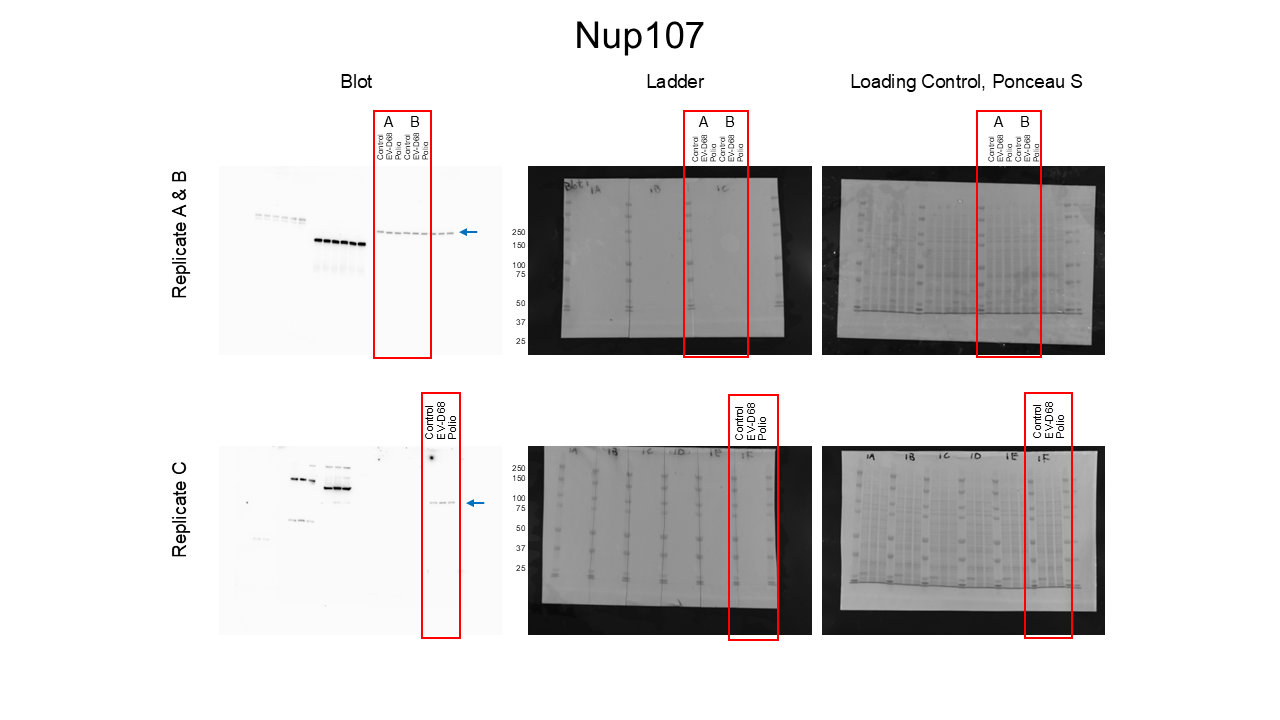

Supplement: Figure 1—source data 2. [file elife-108672-fig1-data2.zip › Figure 1B western blots/Nup107.TIF]

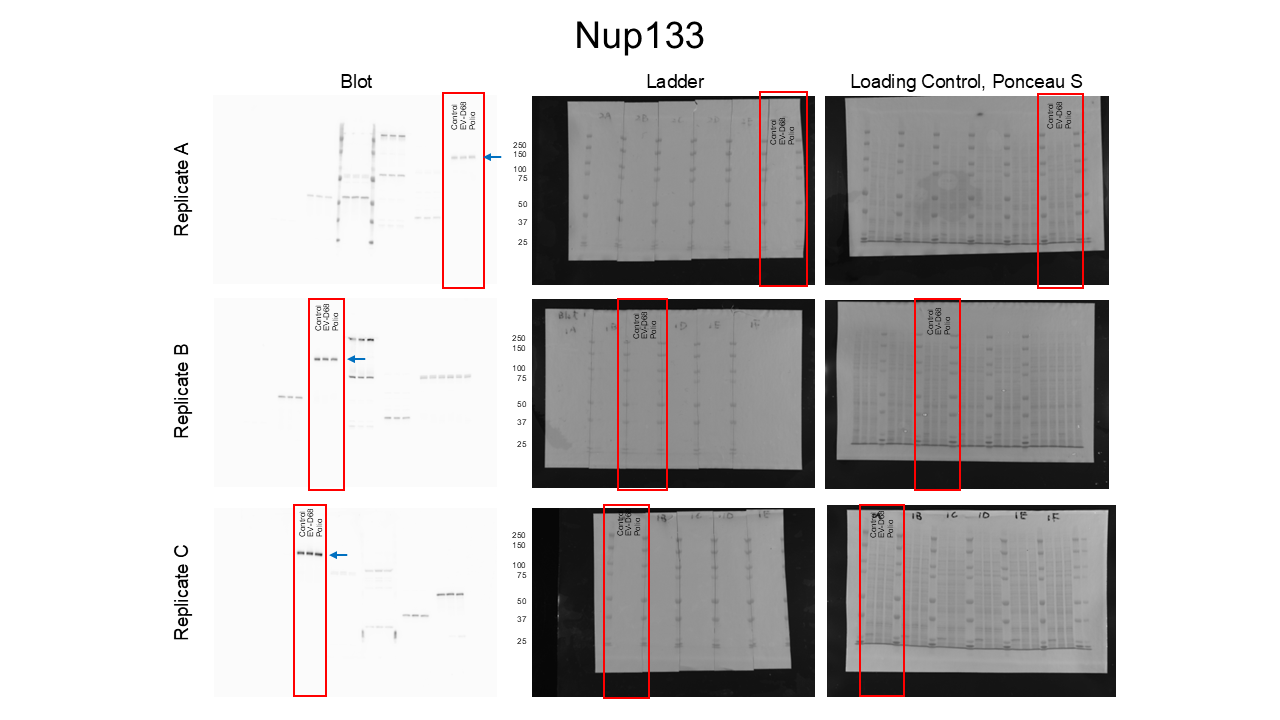

Supplement: Figure 1—source data 2. [file elife-108672-fig1-data2.zip › Figure 1B western blots/Nup133.TIF]

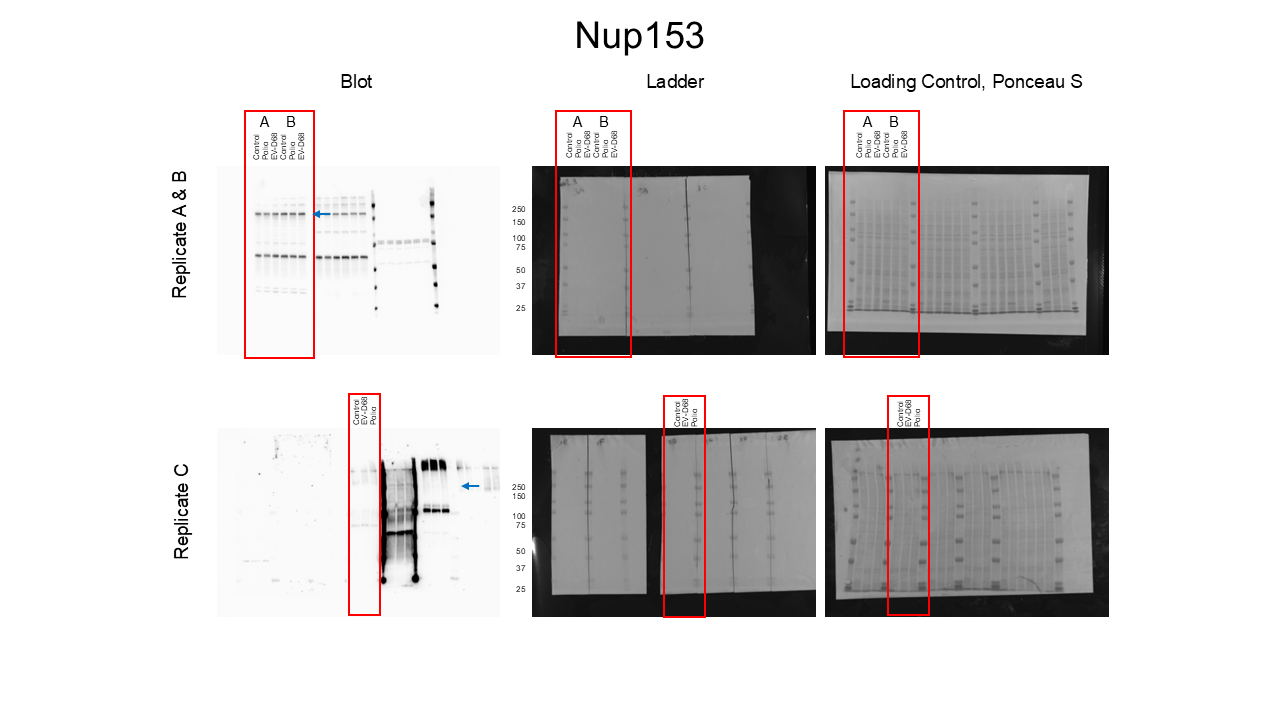

Supplement: Figure 1—source data 2. [file elife-108672-fig1-data2.zip › Figure 1B western blots/Nup153.TIF]

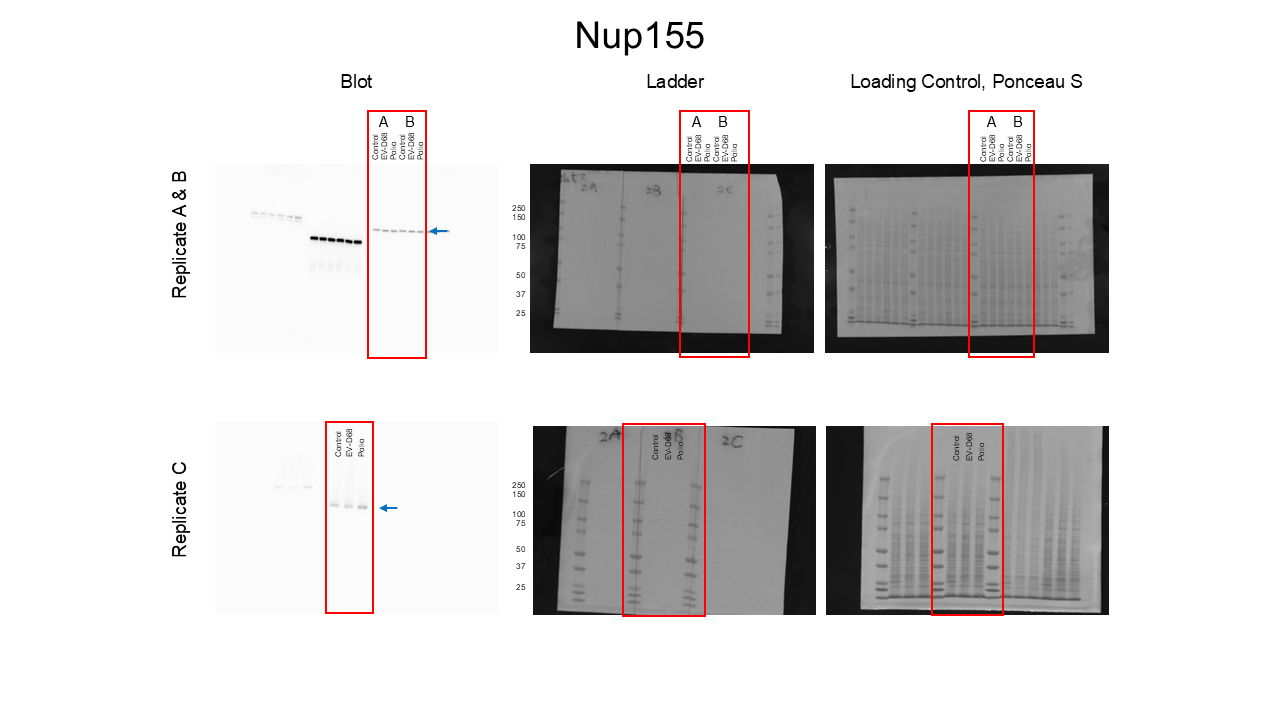

Supplement: Figure 1—source data 2. [file elife-108672-fig1-data2.zip › Figure 1B western blots/Nup155.TIF]

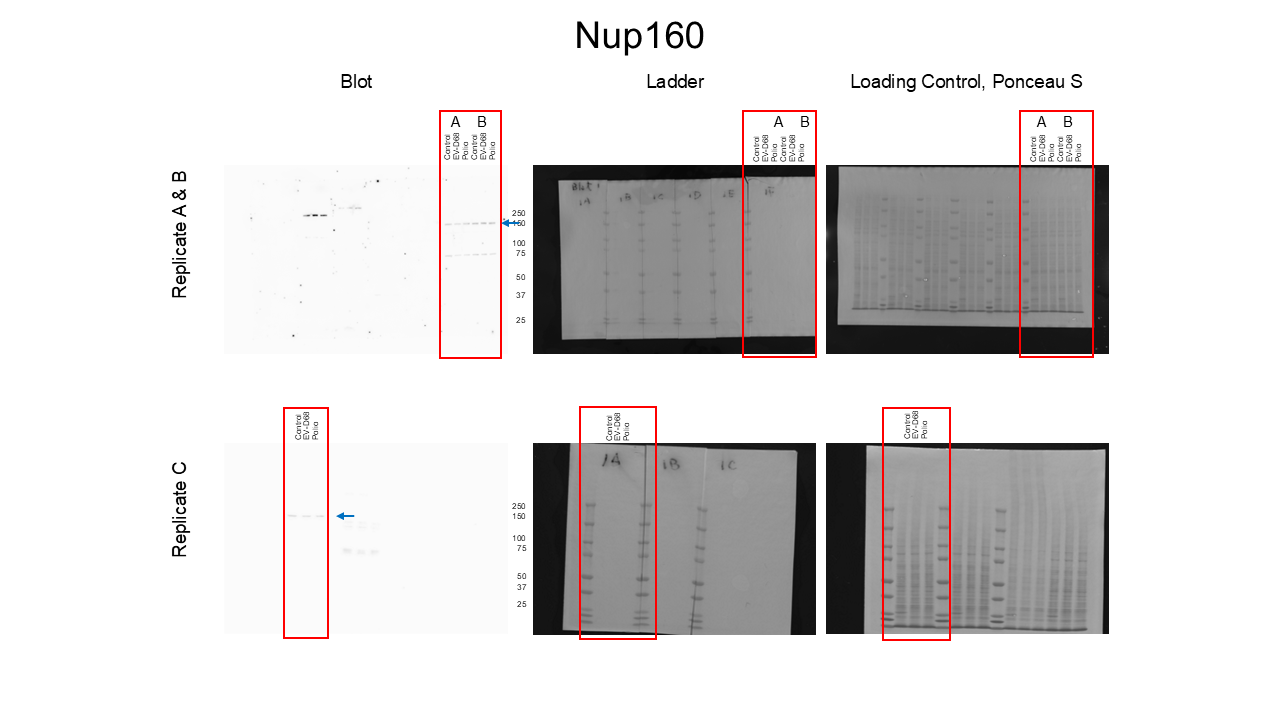

Supplement: Figure 1—source data 2. [file elife-108672-fig1-data2.zip › Figure 1B western blots/Nup160.TIF]

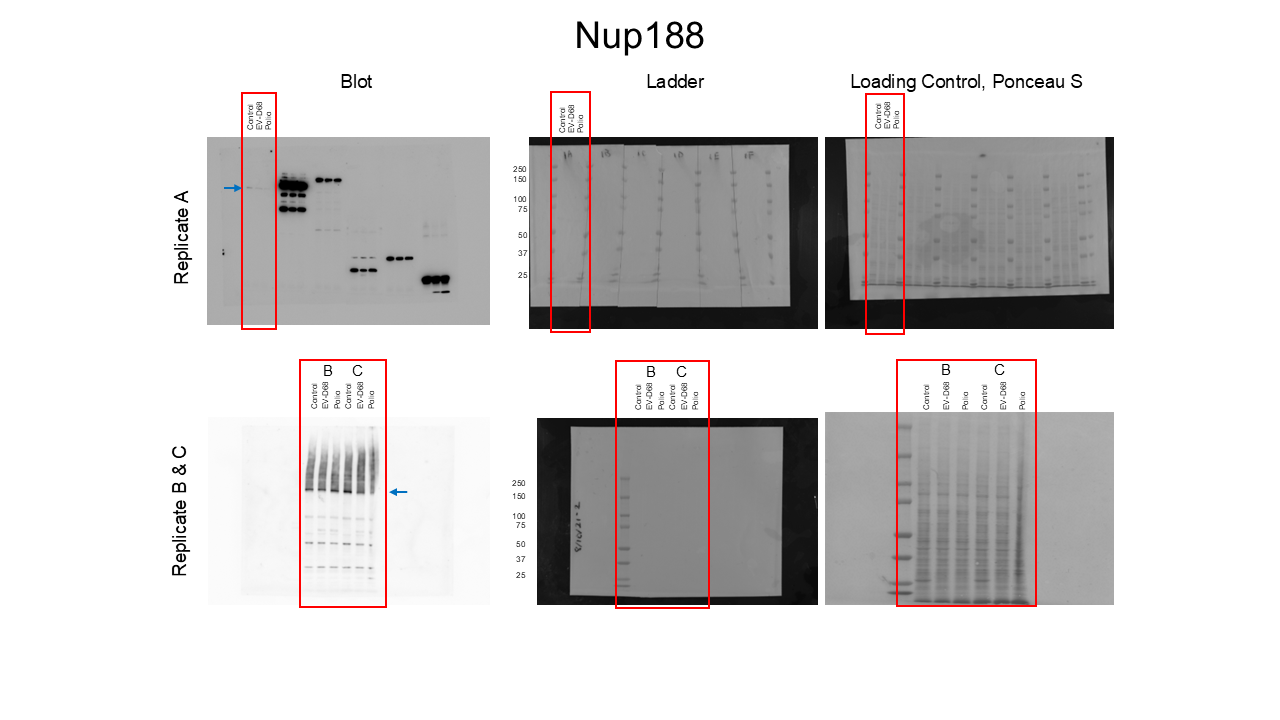

Supplement: Figure 1—source data 2. [file elife-108672-fig1-data2.zip › Figure 1B western blots/Nup188.TIF]

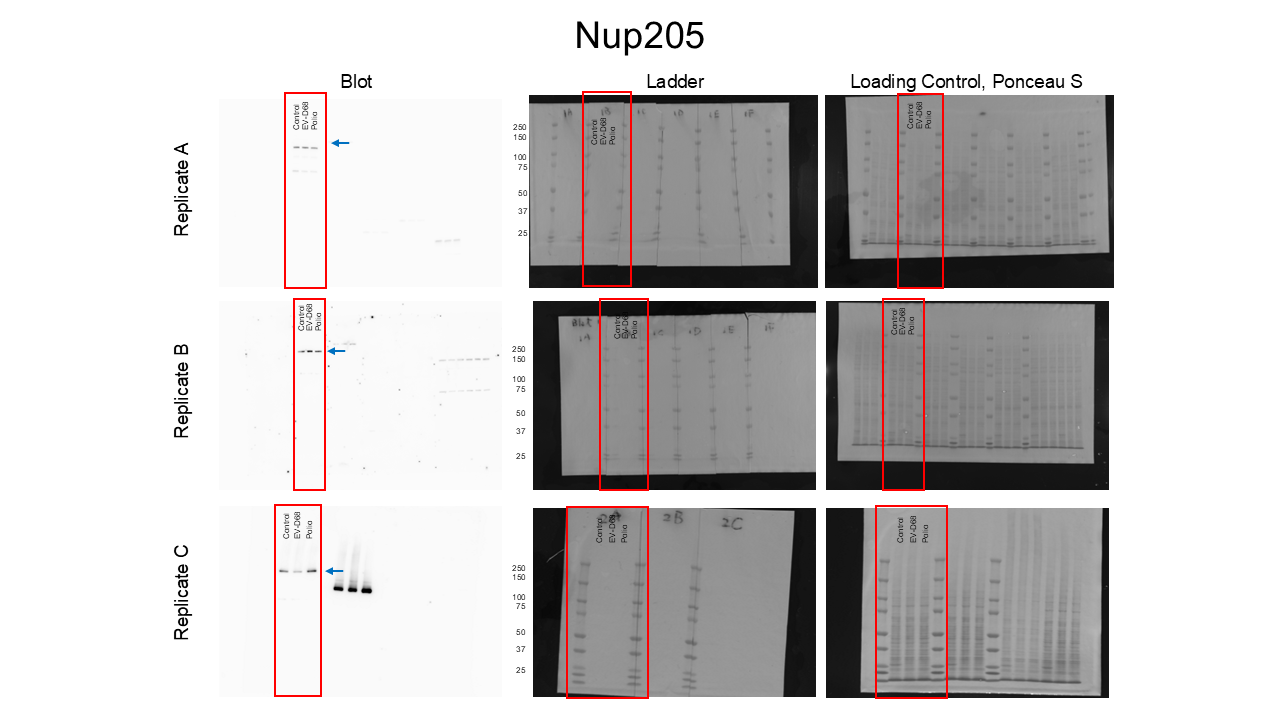

Supplement: Figure 1—source data 2. [file elife-108672-fig1-data2.zip › Figure 1B western blots/Nup205.TIF]

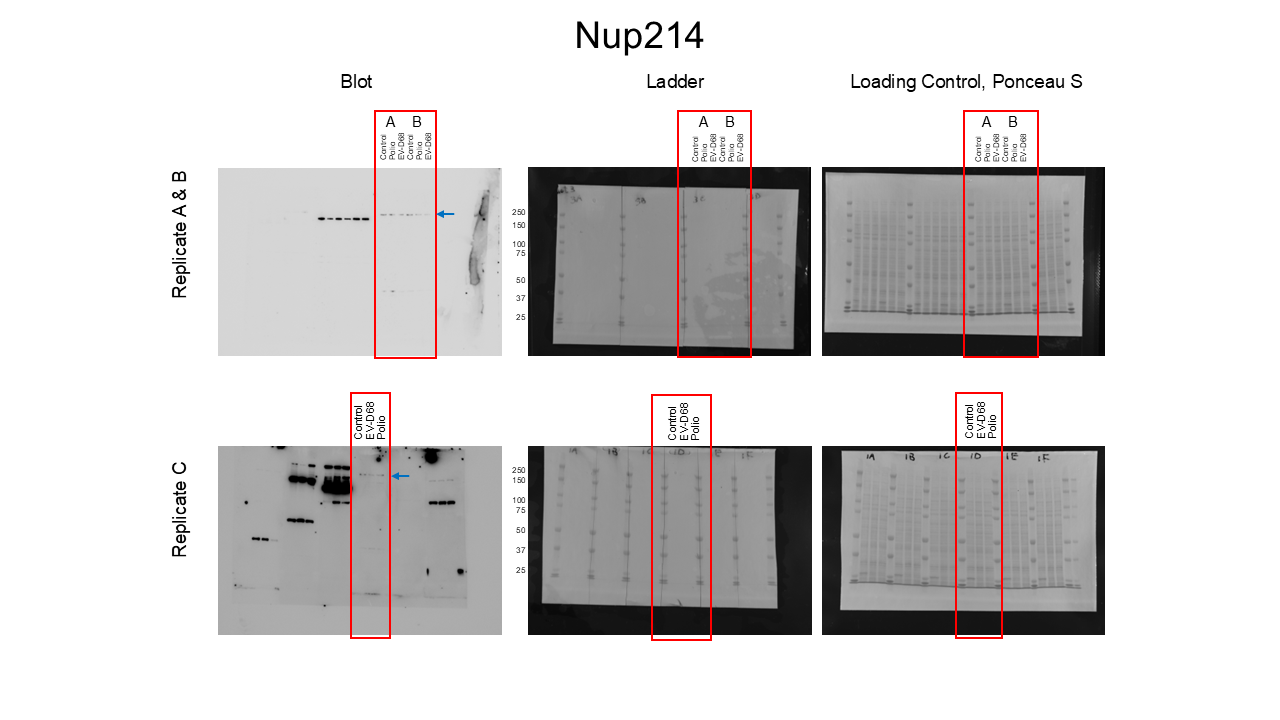

Supplement: Figure 1—source data 2. [file elife-108672-fig1-data2.zip › Figure 1B western blots/Nup214.TIF]

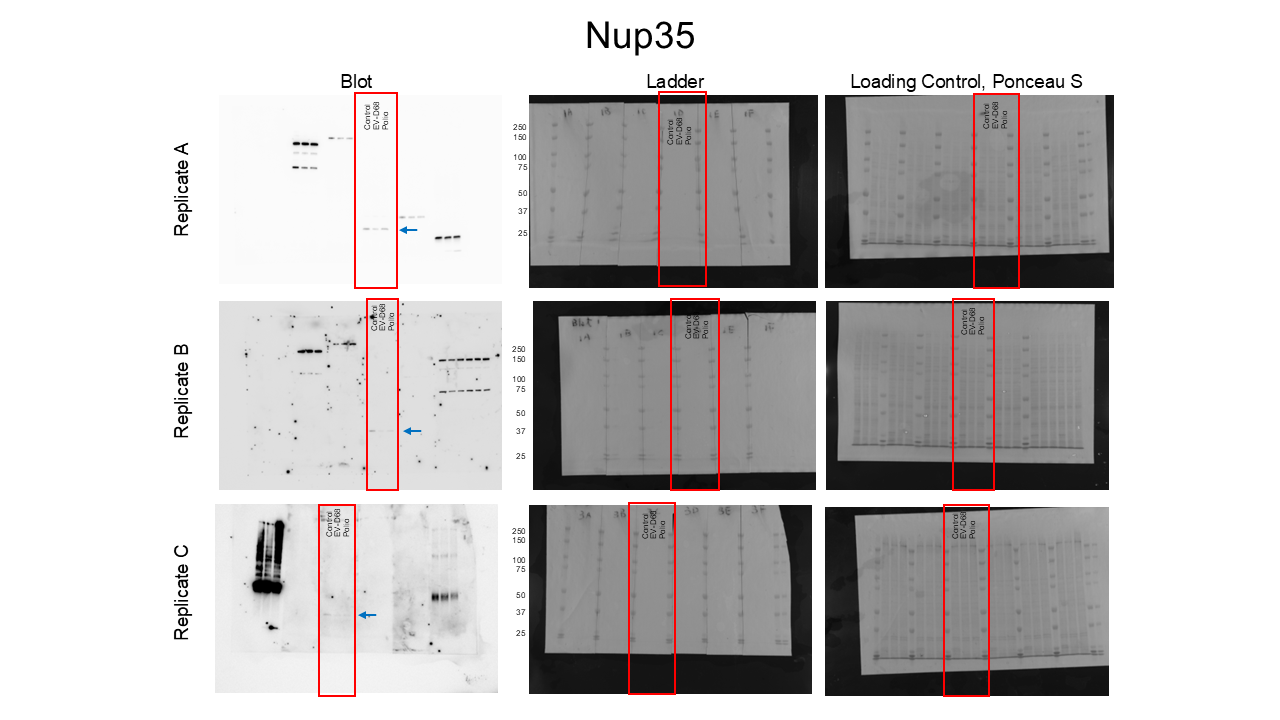

Supplement: Figure 1—source data 2. [file elife-108672-fig1-data2.zip › Figure 1B western blots/Nup35.TIF]

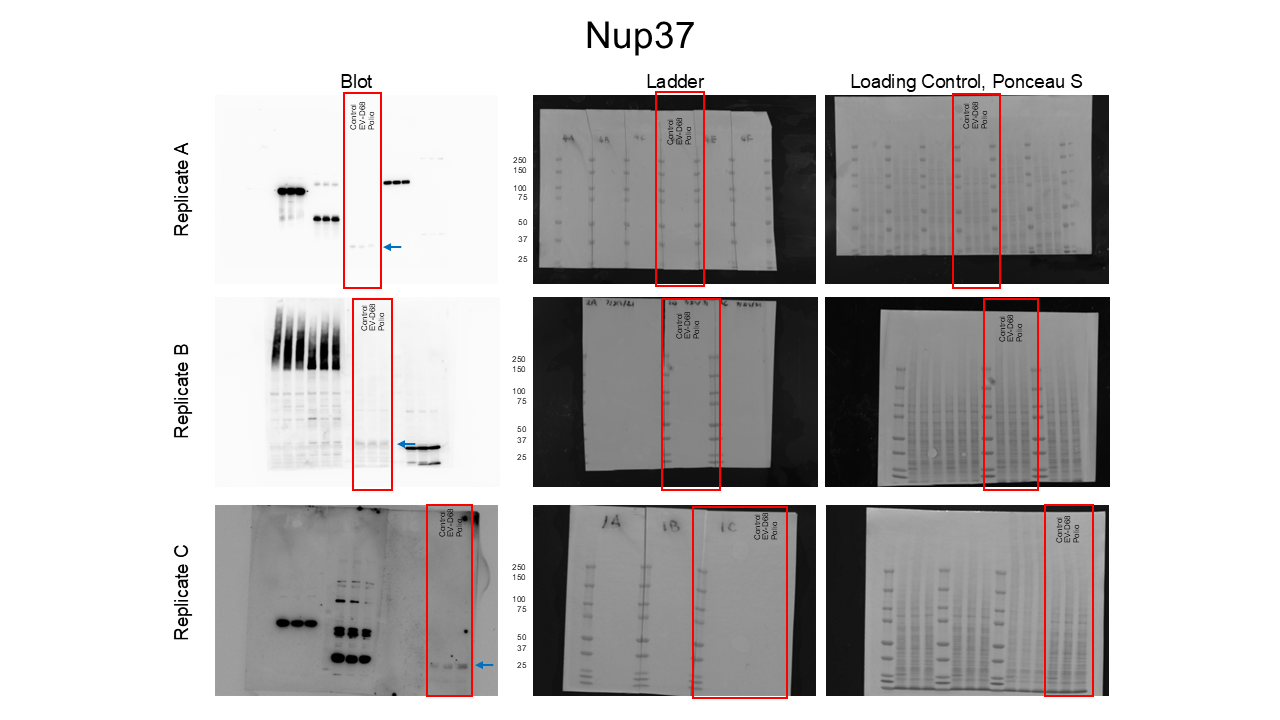

Supplement: Figure 1—source data 2. [file elife-108672-fig1-data2.zip › Figure 1B western blots/Nup37.TIF]

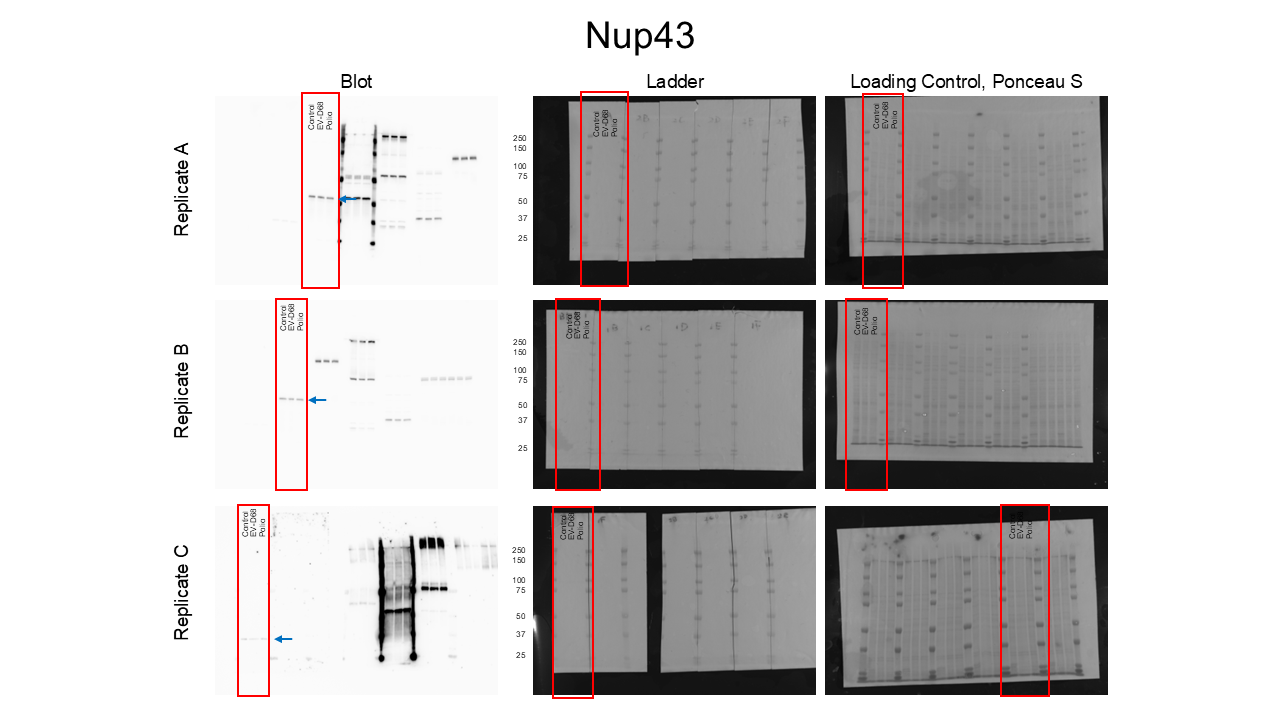

Supplement: Figure 1—source data 2. [file elife-108672-fig1-data2.zip › Figure 1B western blots/Nup43.TIF]

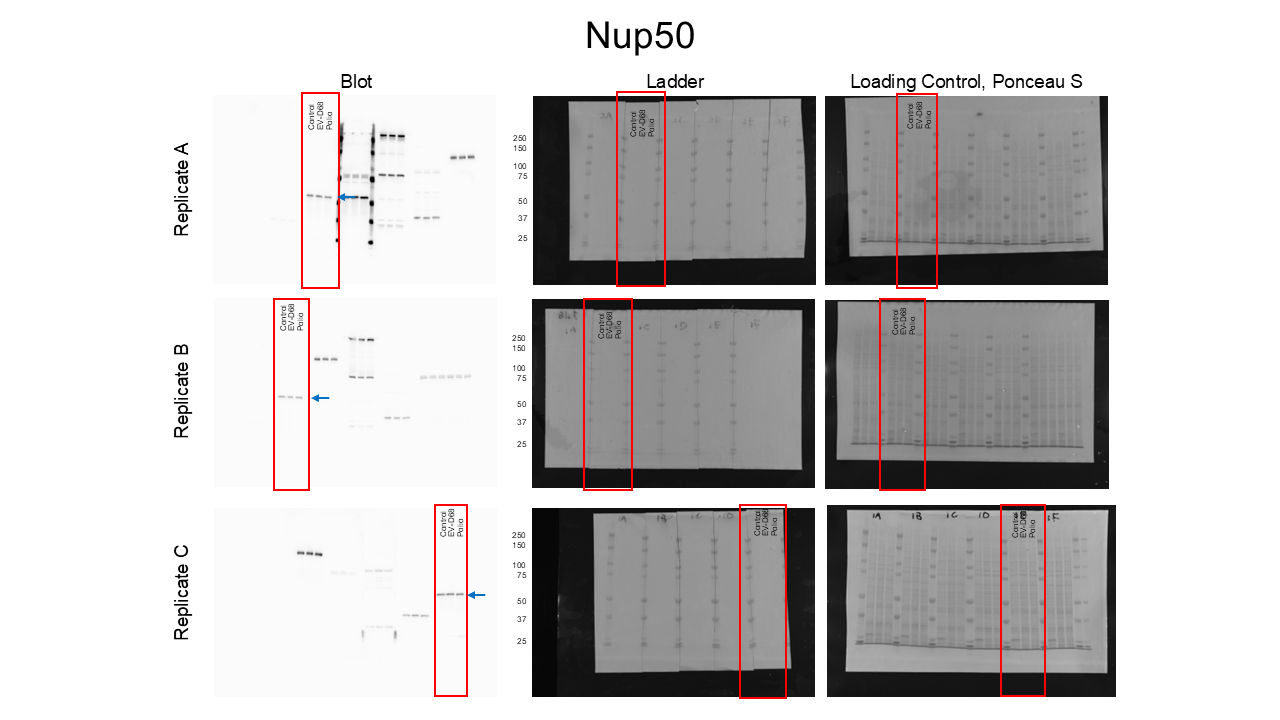

Supplement: Figure 1—source data 2. [file elife-108672-fig1-data2.zip › Figure 1B western blots/Nup50.TIF]

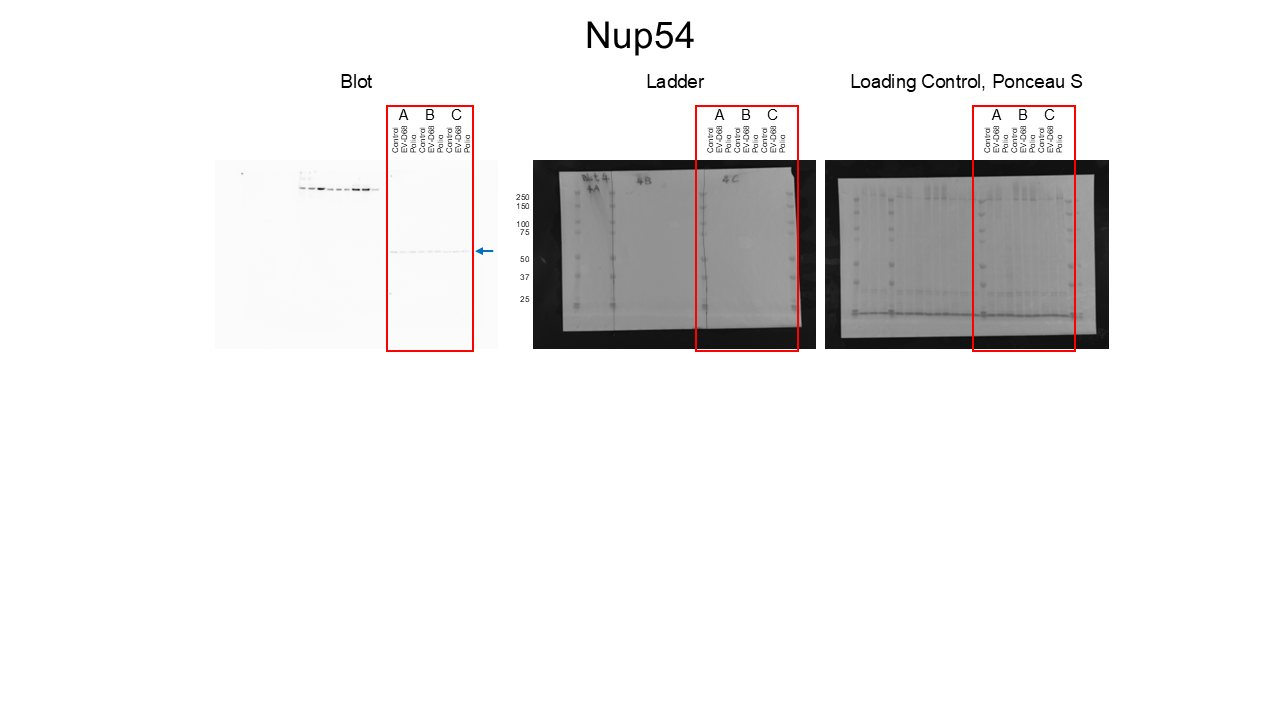

Supplement: Figure 1—source data 2. [file elife-108672-fig1-data2.zip › Figure 1B western blots/Nup54.TIF]

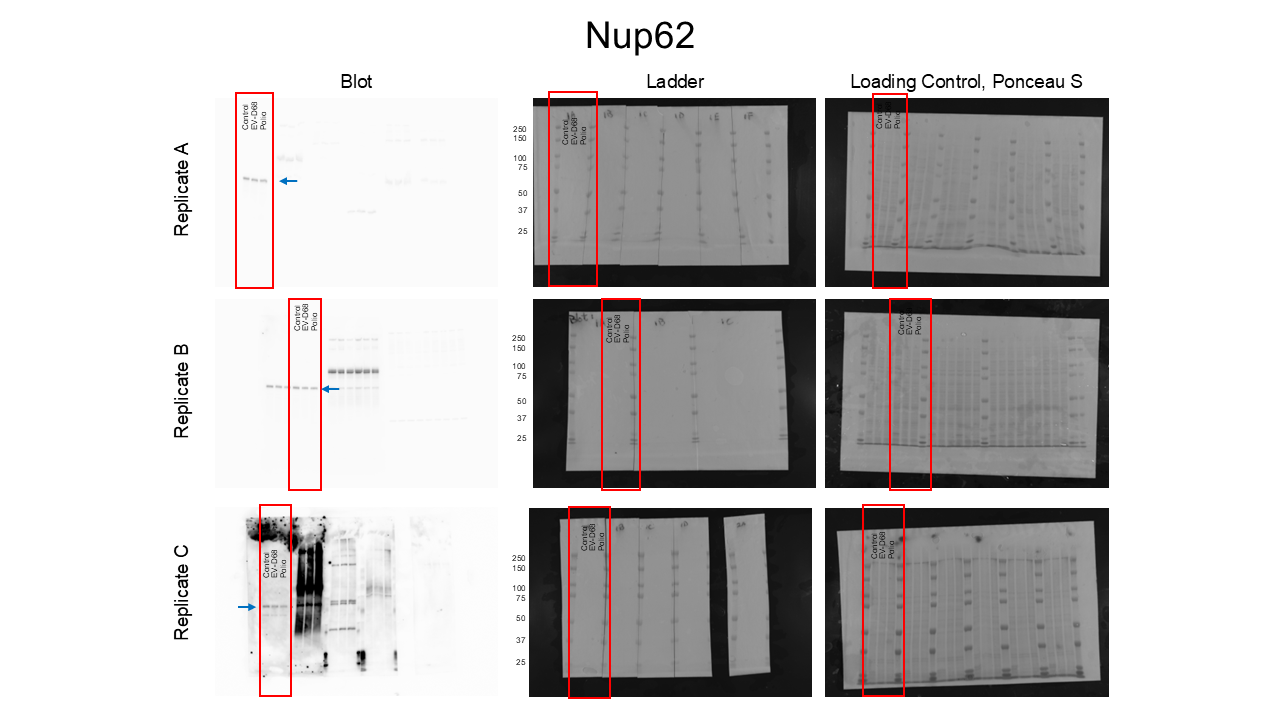

Supplement: Figure 1—source data 2. [file elife-108672-fig1-data2.zip › Figure 1B western blots/Nup62.TIF]

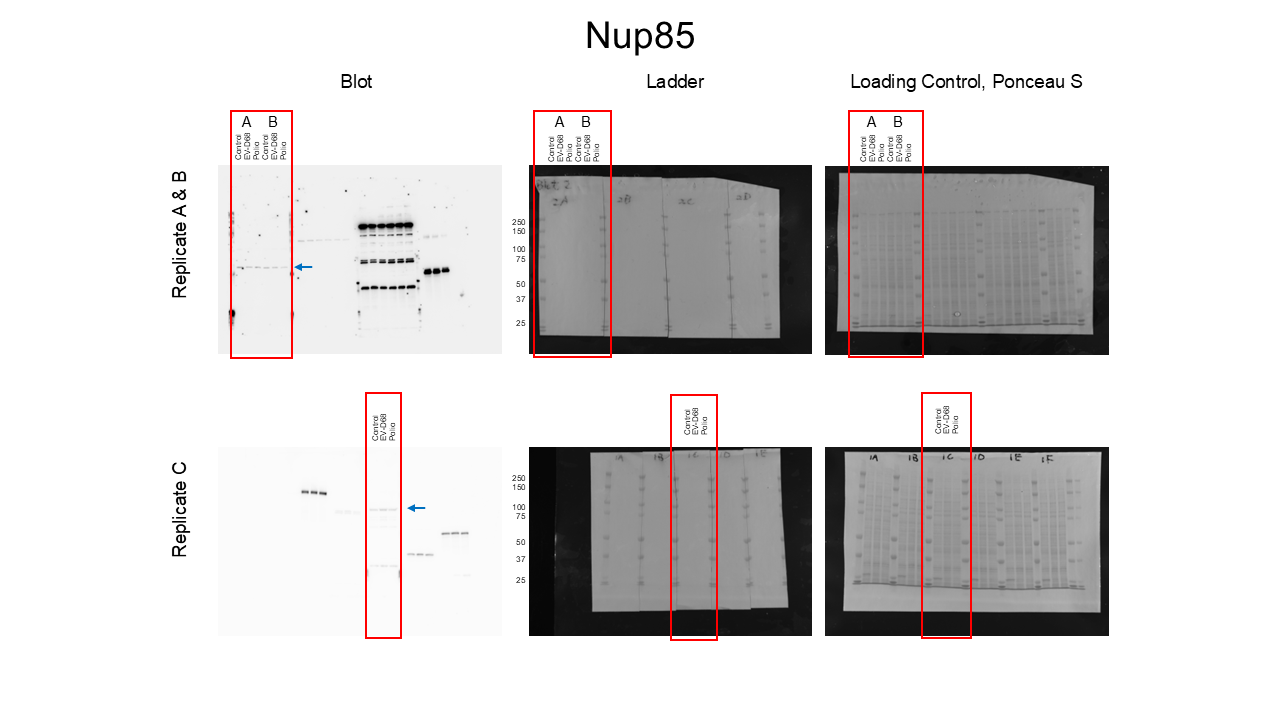

Supplement: Figure 1—source data 2. [file elife-108672-fig1-data2.zip › Figure 1B western blots/Nup85.TIF]

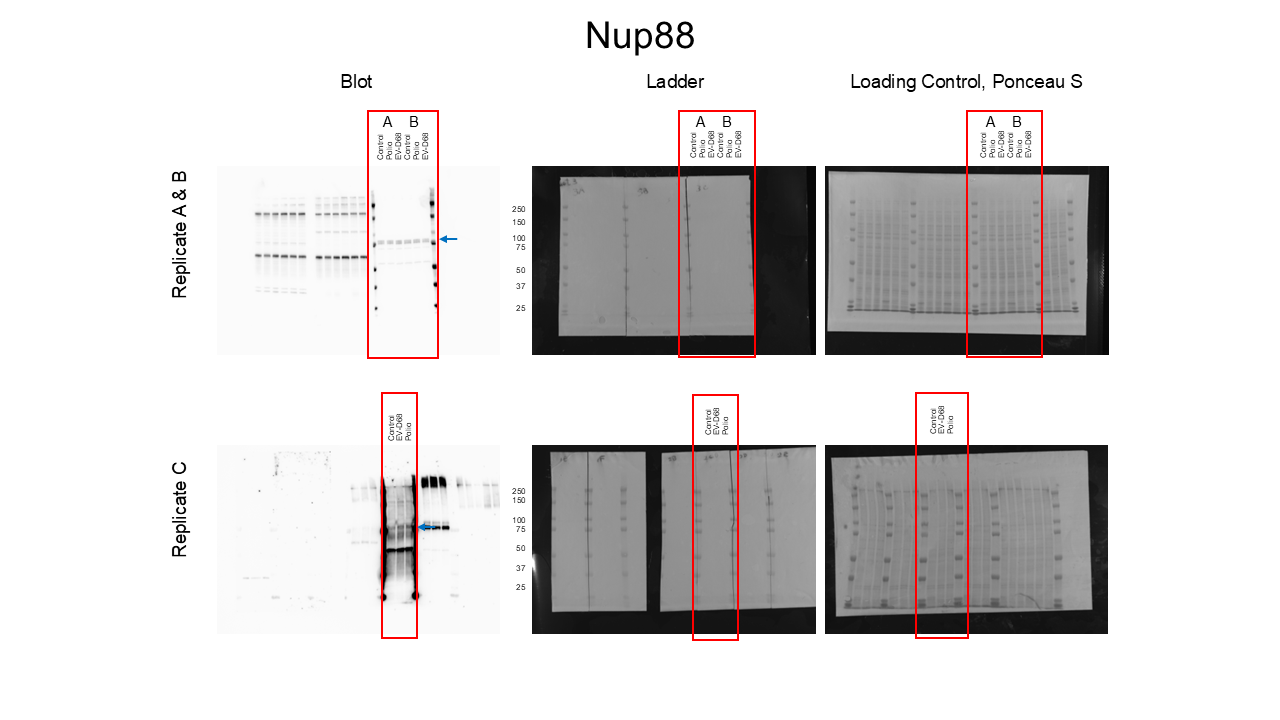

Supplement: Figure 1—source data 2. [file elife-108672-fig1-data2.zip › Figure 1B western blots/Nup88.TIF]

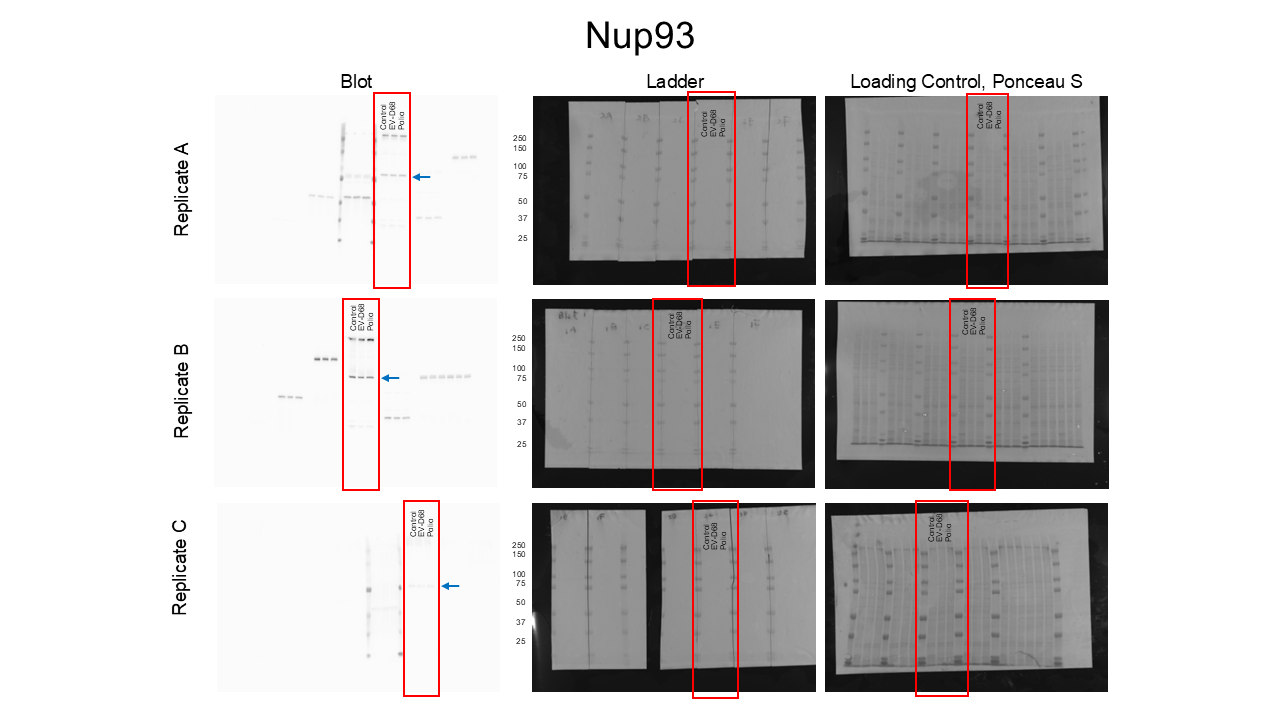

Supplement: Figure 1—source data 2. [file elife-108672-fig1-data2.zip › Figure 1B western blots/Nup93.TIF]

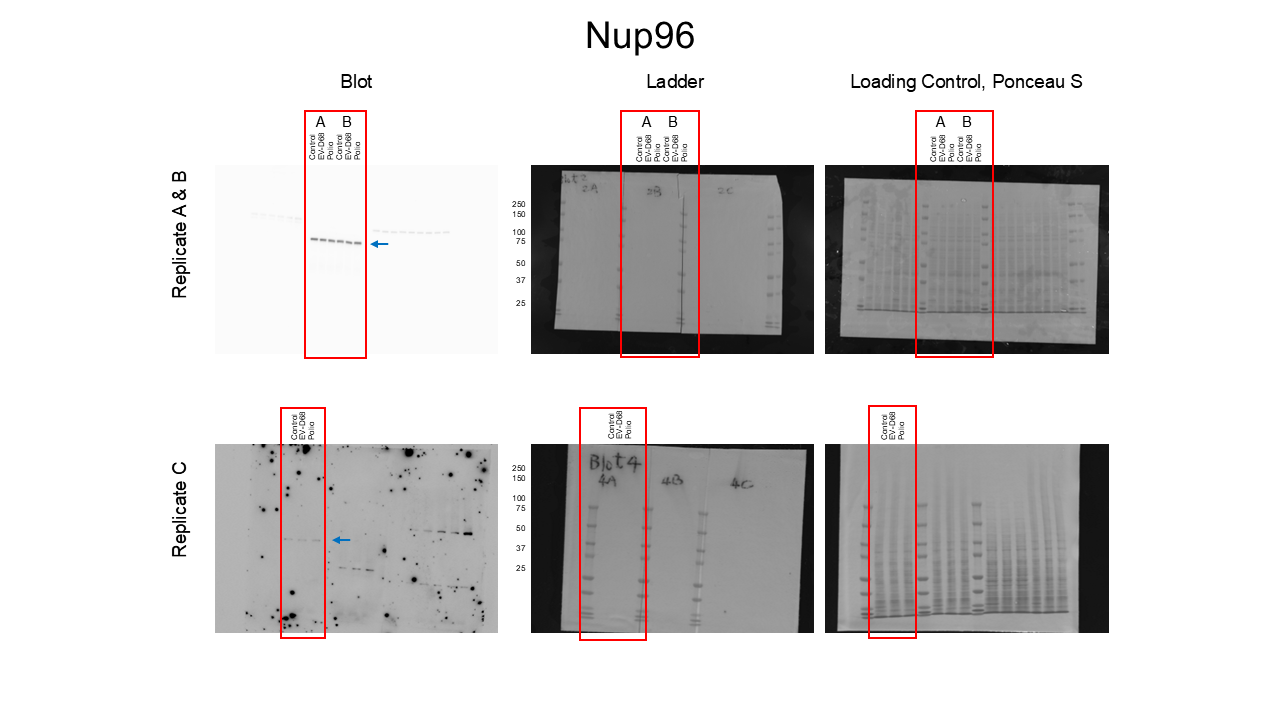

Supplement: Figure 1—source data 2. [file elife-108672-fig1-data2.zip › Figure 1B western blots/Nup96.TIF]

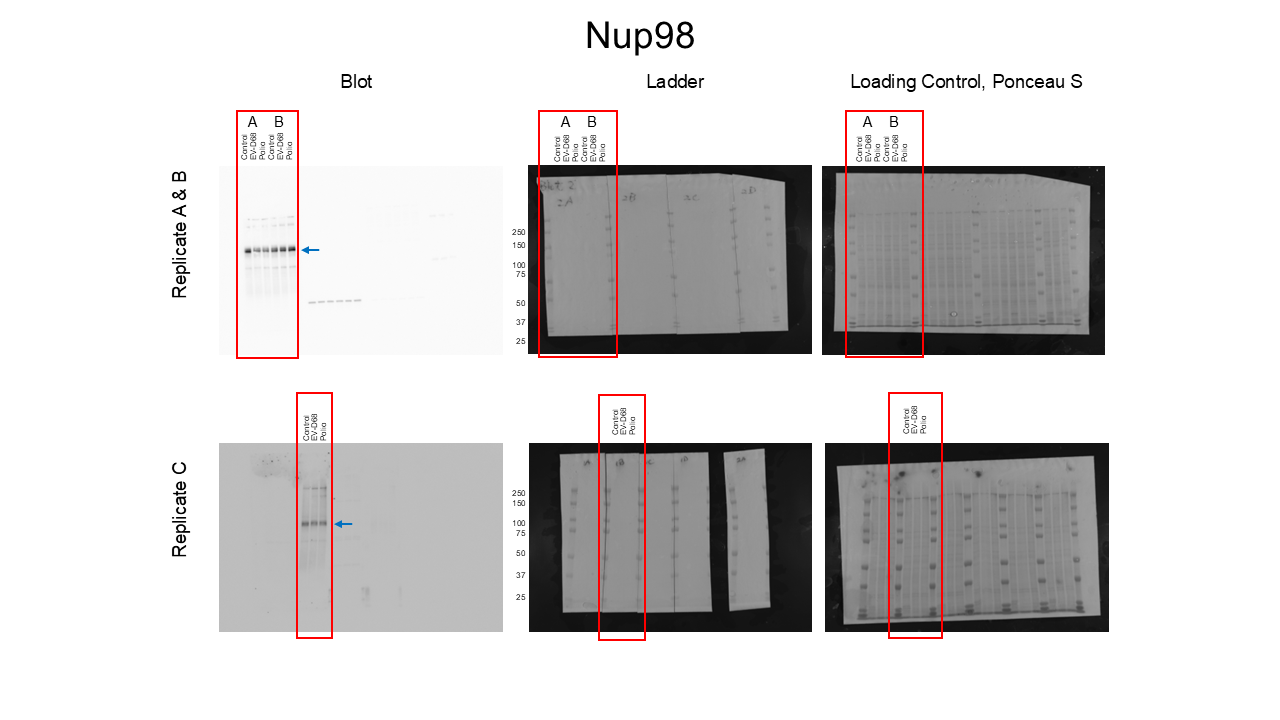

Supplement: Figure 1—source data 2. [file elife-108672-fig1-data2.zip › Figure 1B western blots/Nup98.TIF]

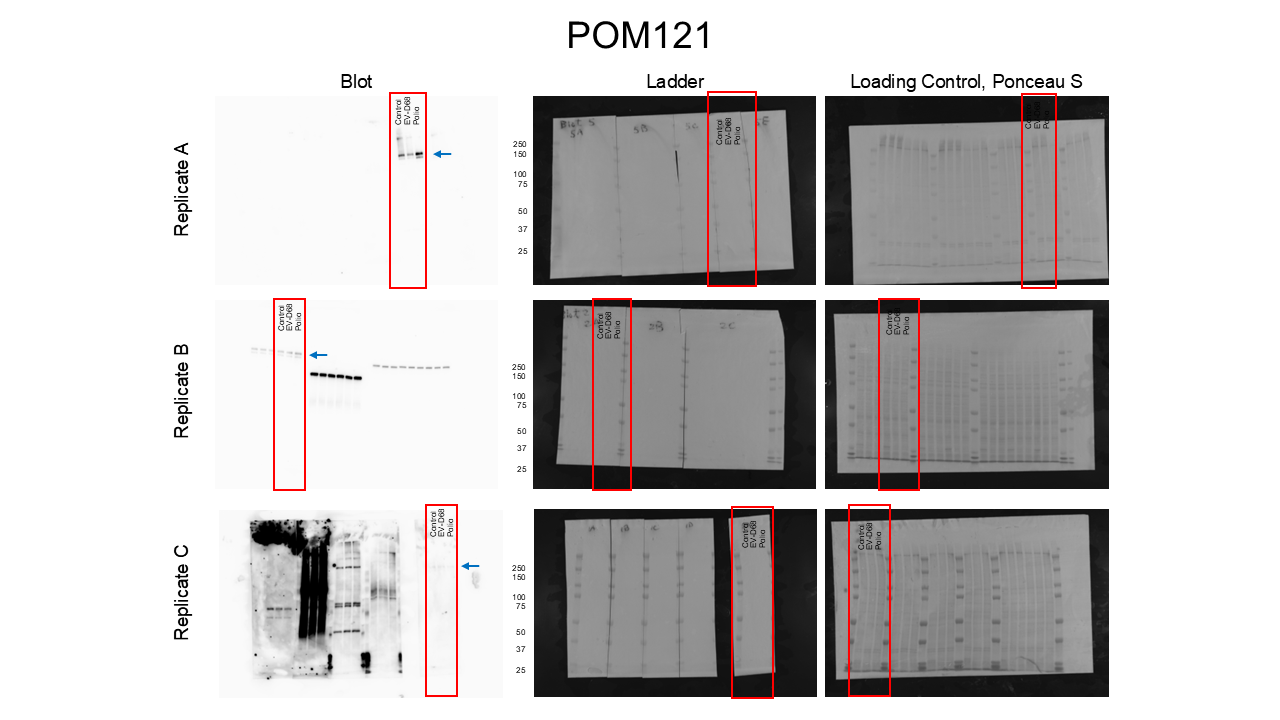

Supplement: Figure 1—source data 2. [file elife-108672-fig1-data2.zip › Figure 1B western blots/POM121.tif]

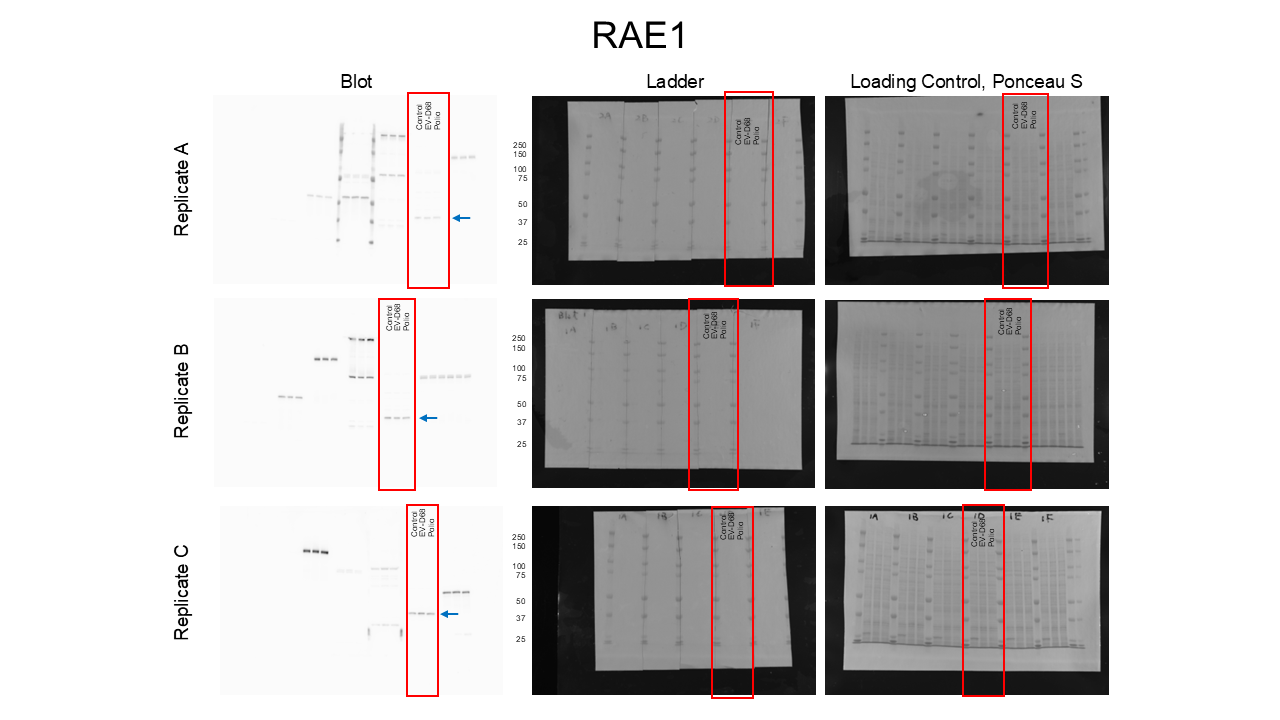

Supplement: Figure 1—source data 2. [file elife-108672-fig1-data2.zip › Figure 1B western blots/RAE1.TIF]

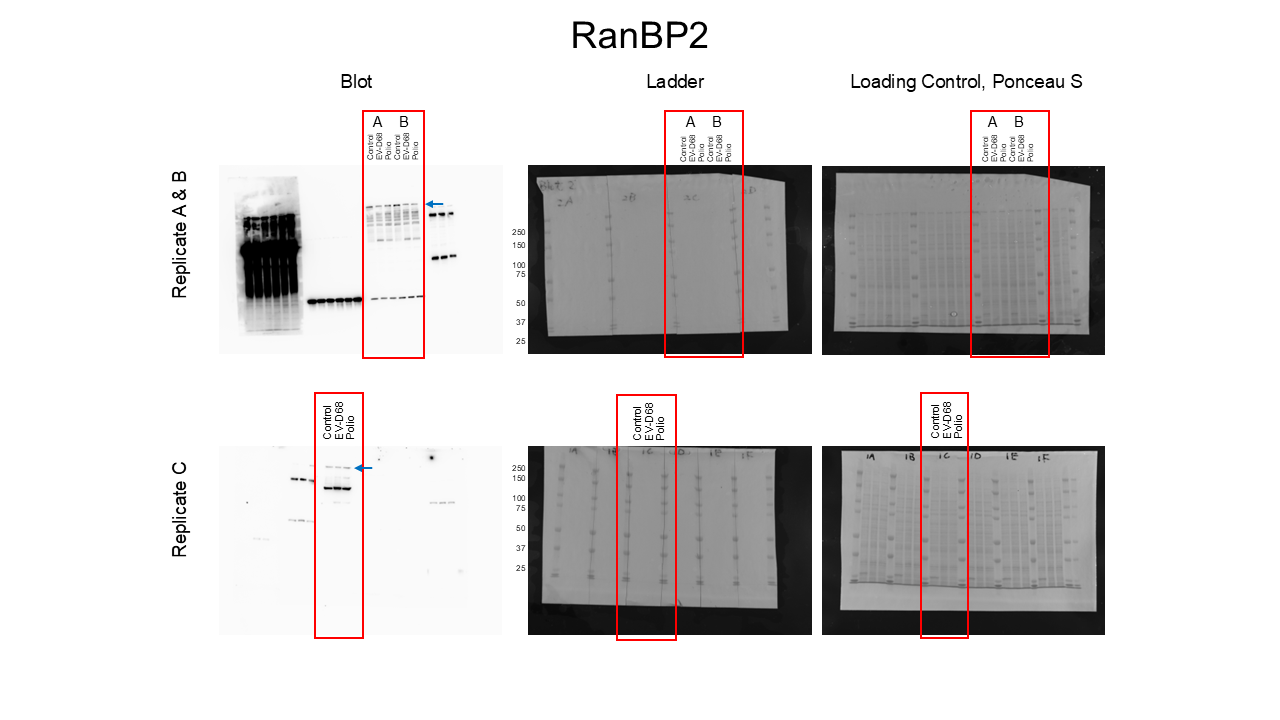

Supplement: Figure 1—source data 2. [file elife-108672-fig1-data2.zip › Figure 1B western blots/RanBP2.TIF]

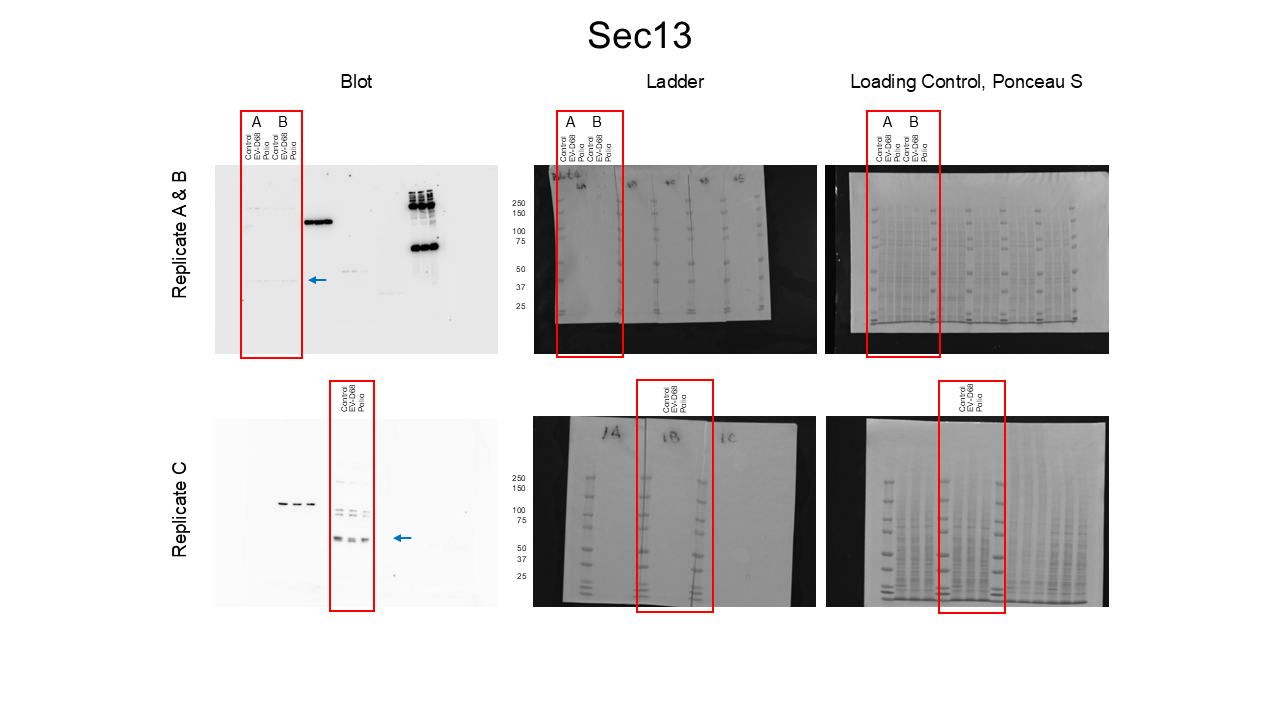

Supplement: Figure 1—source data 2. [file elife-108672-fig1-data2.zip › Figure 1B western blots/Sec13.TIF]

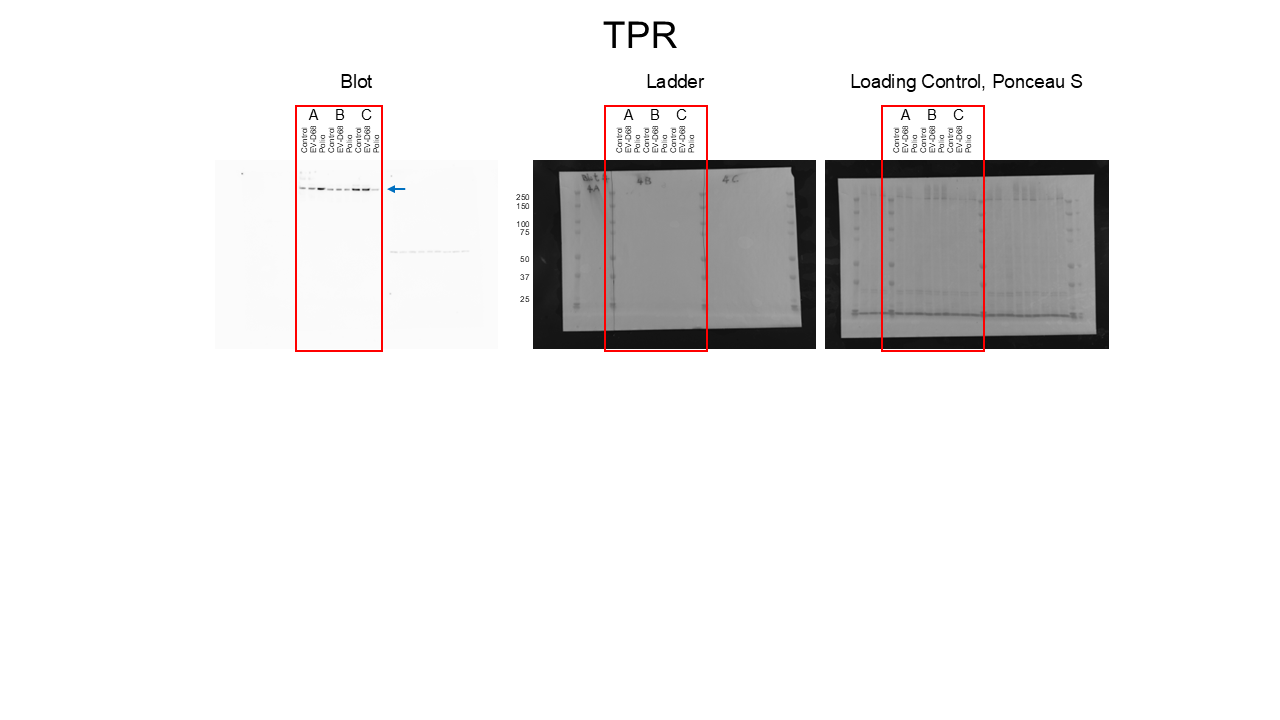

Supplement: Figure 1—source data 2. [file elife-108672-fig1-data2.zip › Figure 1B western blots/TPR.TIF]

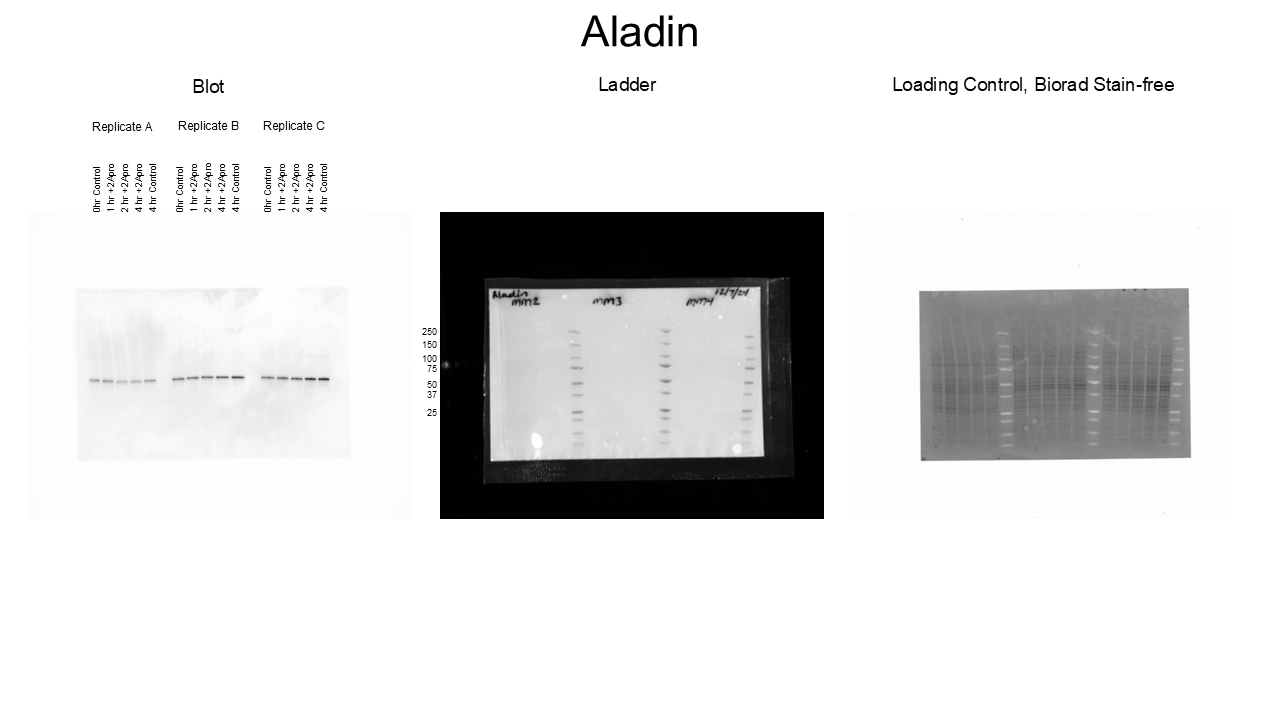

Supplement: Figure 1—source data 2. [file elife-108672-fig1-data2.zip › Figure 1C western blots/Aladin.TIF]

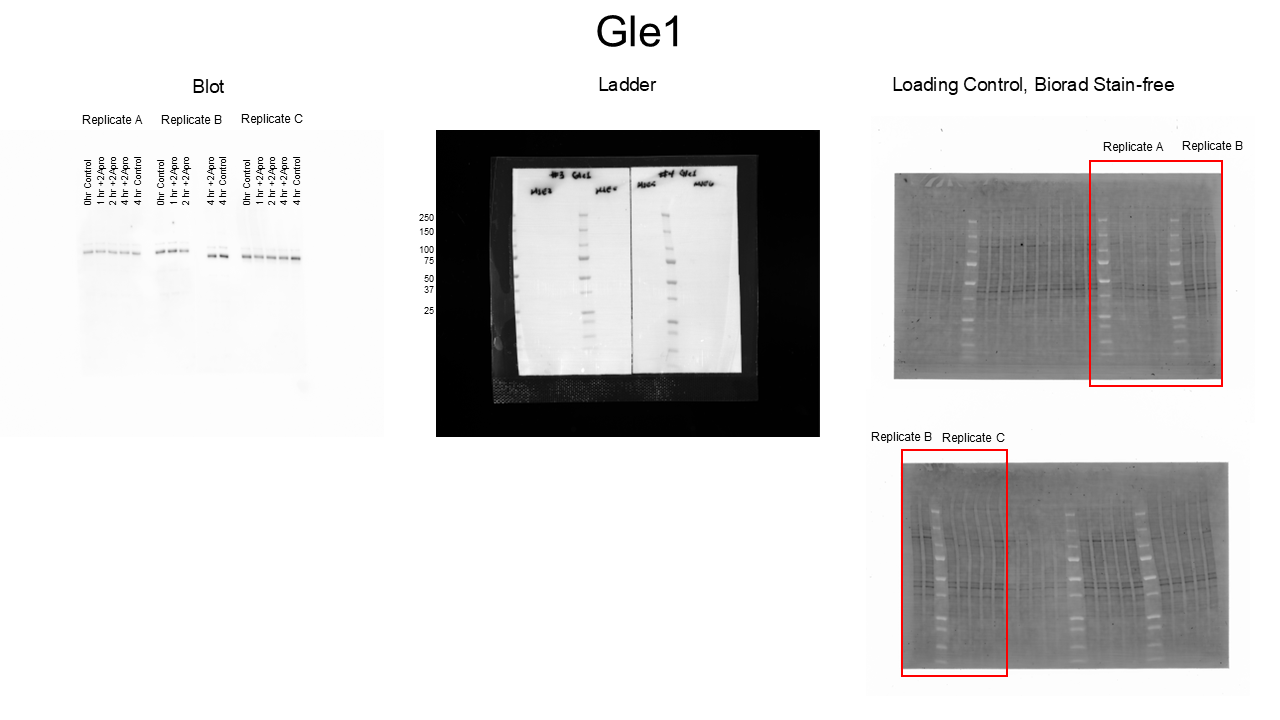

Supplement: Figure 1—source data 2. [file elife-108672-fig1-data2.zip › Figure 1C western blots/Gle1.TIF]

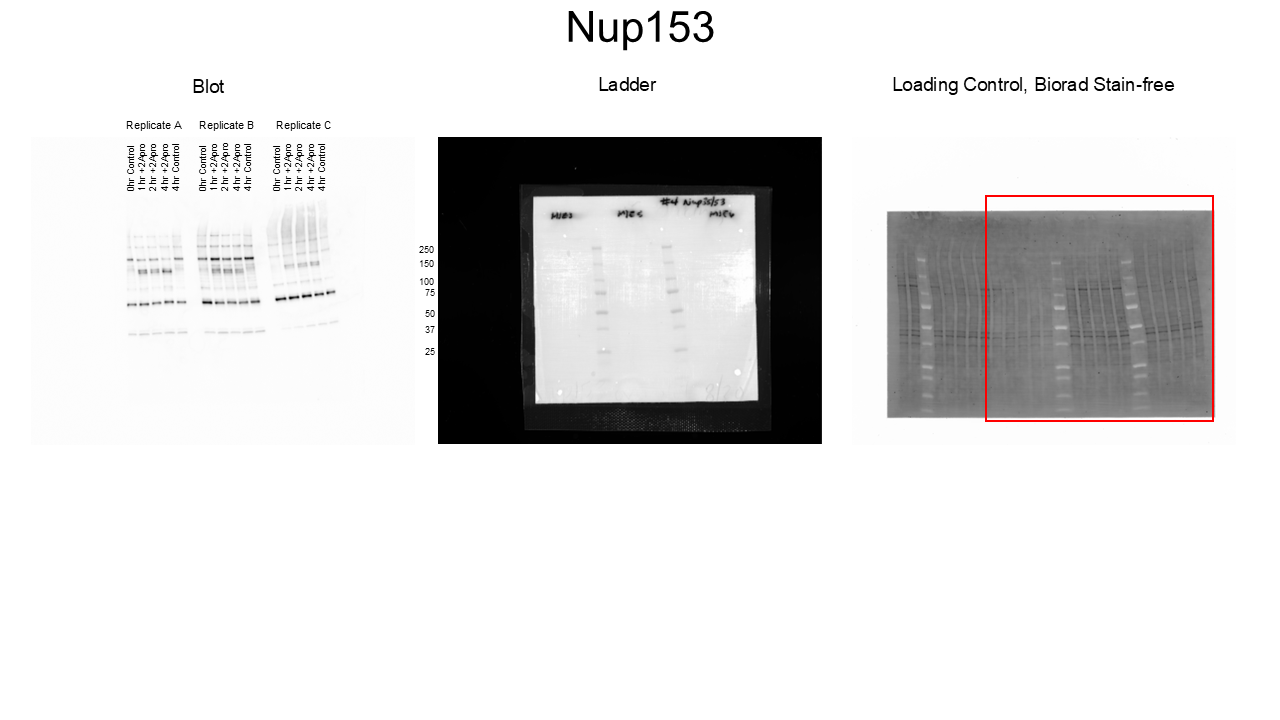

Supplement: Figure 1—source data 2. [file elife-108672-fig1-data2.zip › Figure 1C western blots/Nup153.TIF]

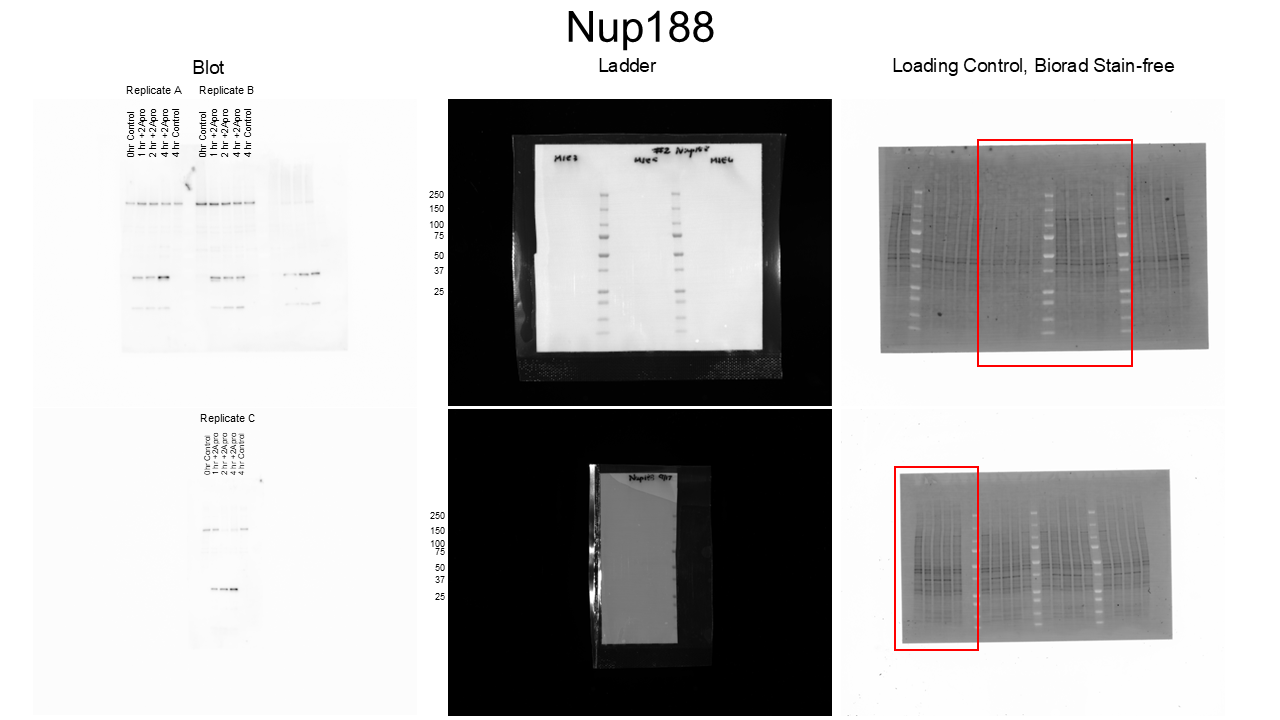

Supplement: Figure 1—source data 2. [file elife-108672-fig1-data2.zip › Figure 1C western blots/Nup188.TIF]

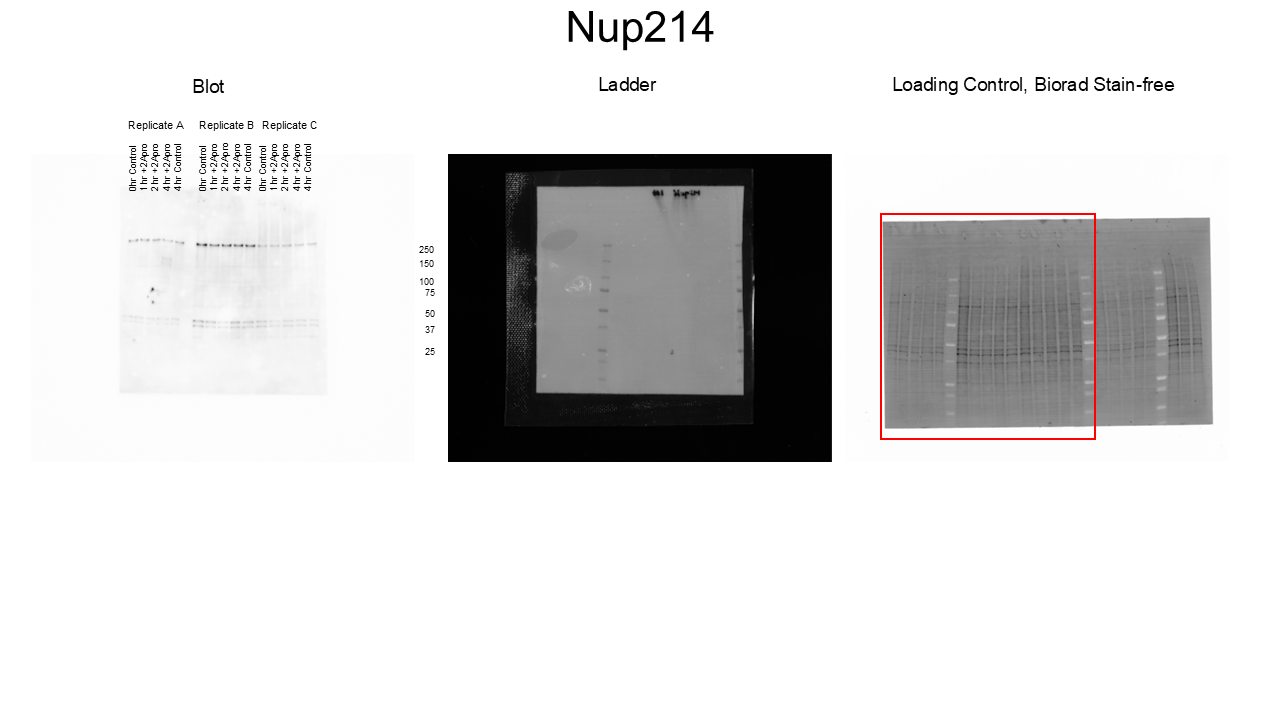

Supplement: Figure 1—source data 2. [file elife-108672-fig1-data2.zip › Figure 1C western blots/Nup214.TIF]

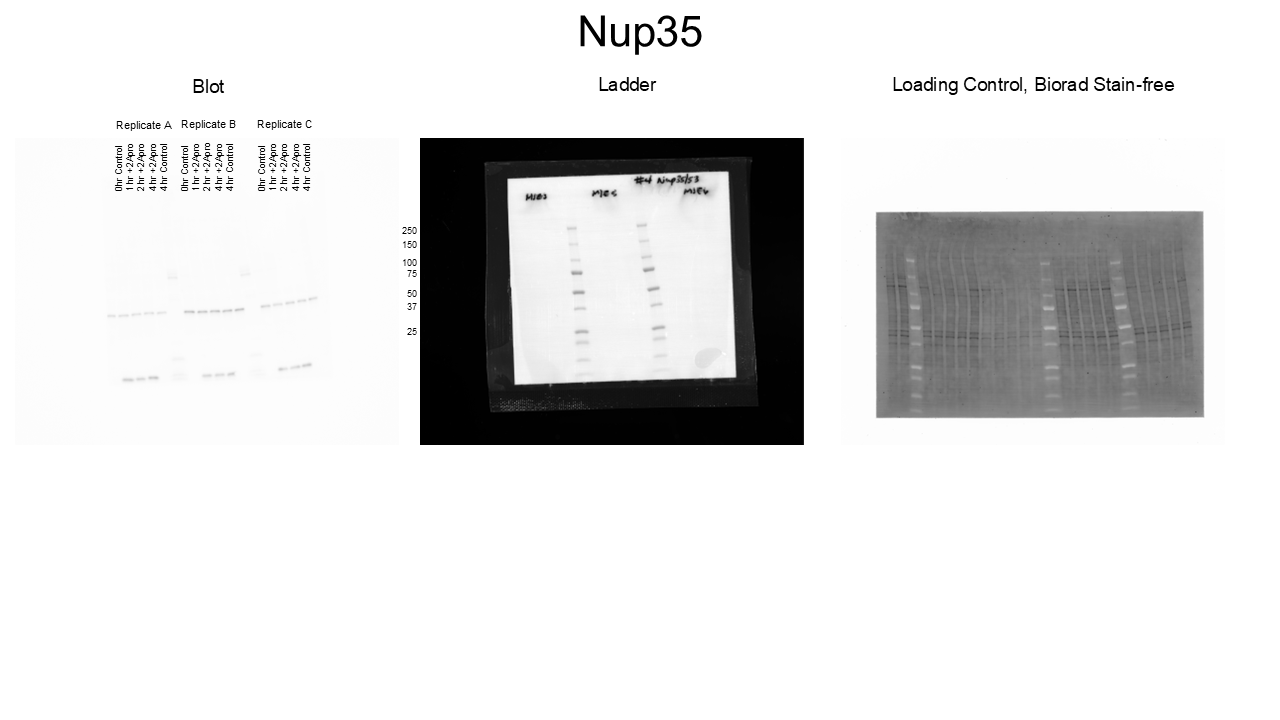

Supplement: Figure 1—source data 2. [file elife-108672-fig1-data2.zip › Figure 1C western blots/Nup35.TIF]

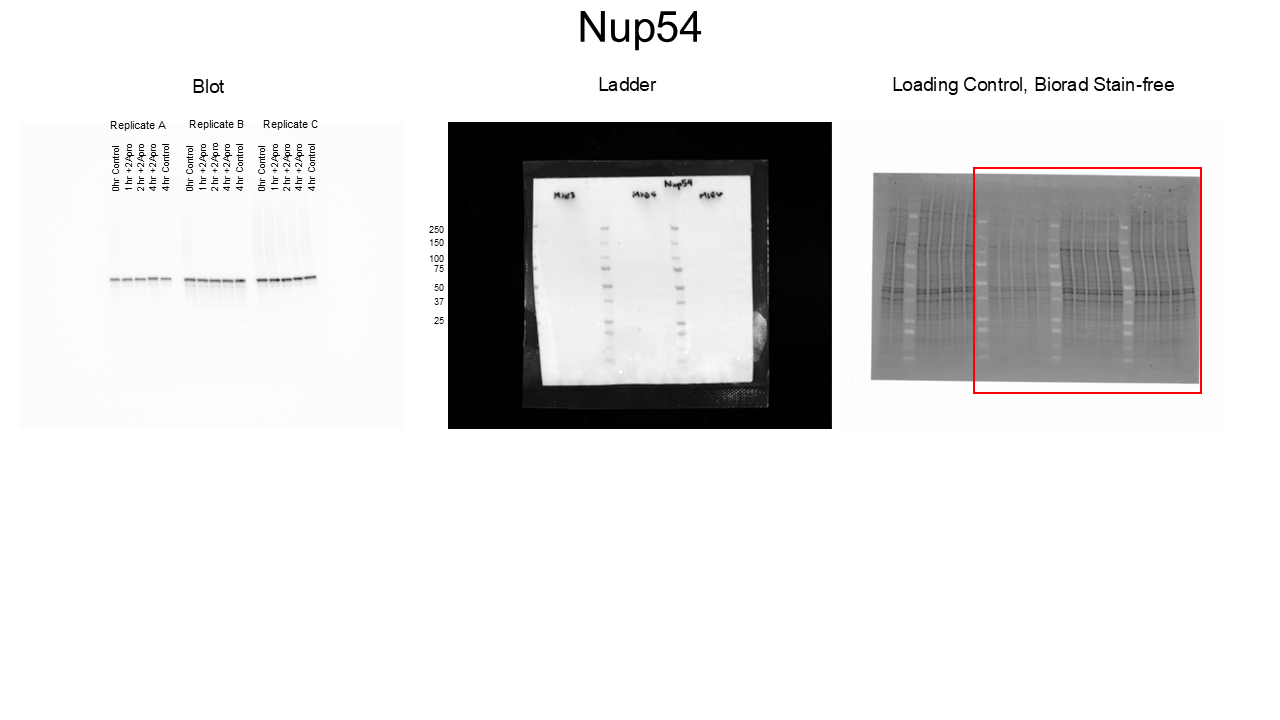

Supplement: Figure 1—source data 2. [file elife-108672-fig1-data2.zip › Figure 1C western blots/Nup54.TIF]

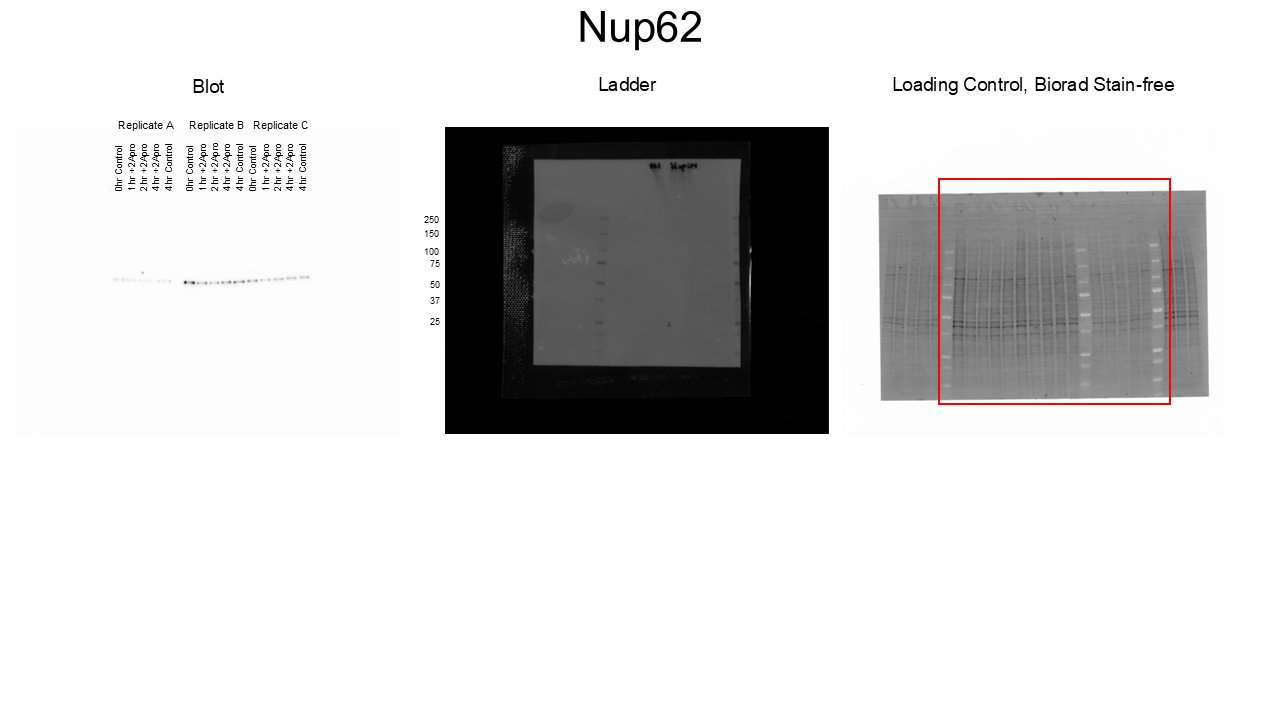

Supplement: Figure 1—source data 2. [file elife-108672-fig1-data2.zip › Figure 1C western blots/Nup62.TIF]

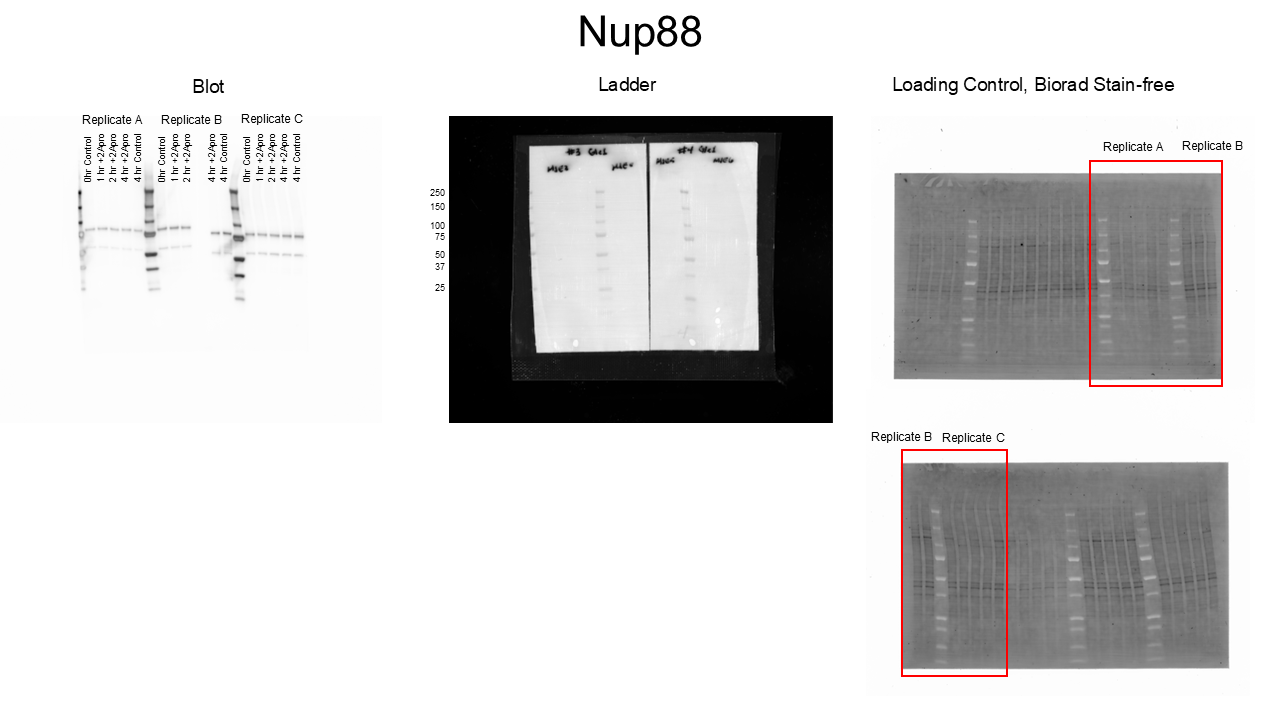

Supplement: Figure 1—source data 2. [file elife-108672-fig1-data2.zip › Figure 1C western blots/Nup88.TIF]

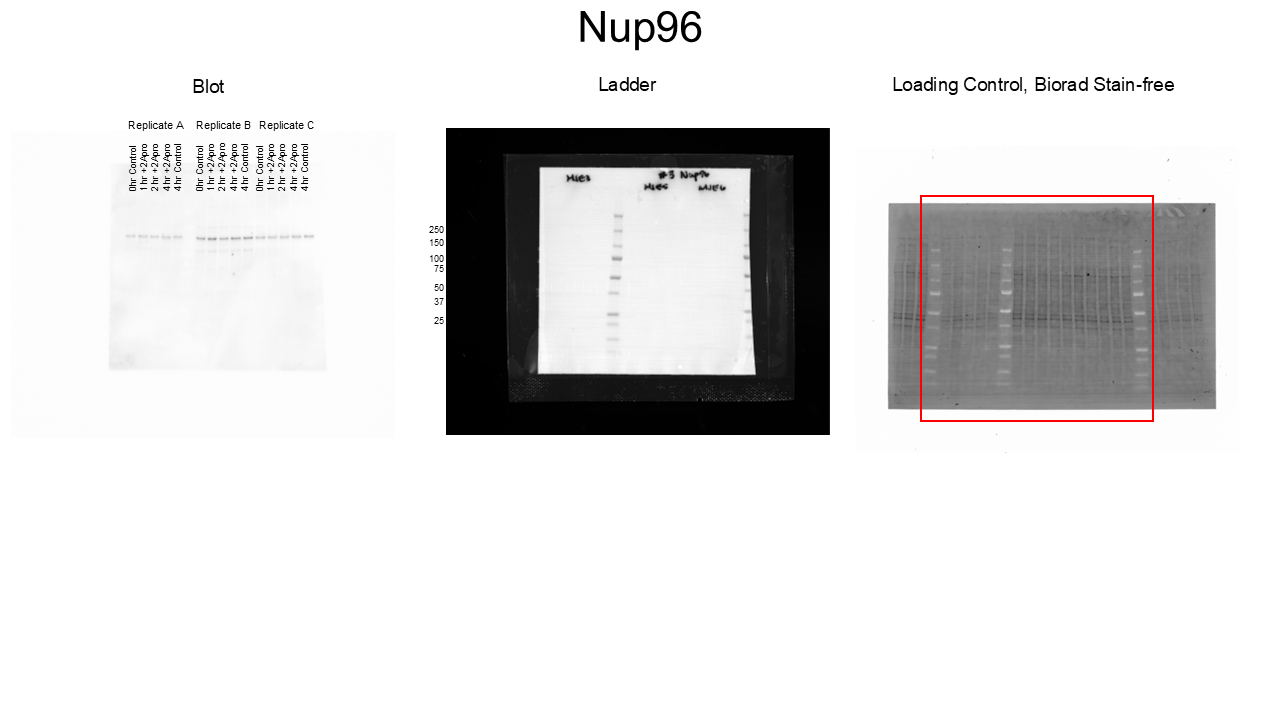

Supplement: Figure 1—source data 2. [file elife-108672-fig1-data2.zip › Figure 1C western blots/Nup96.TIF]

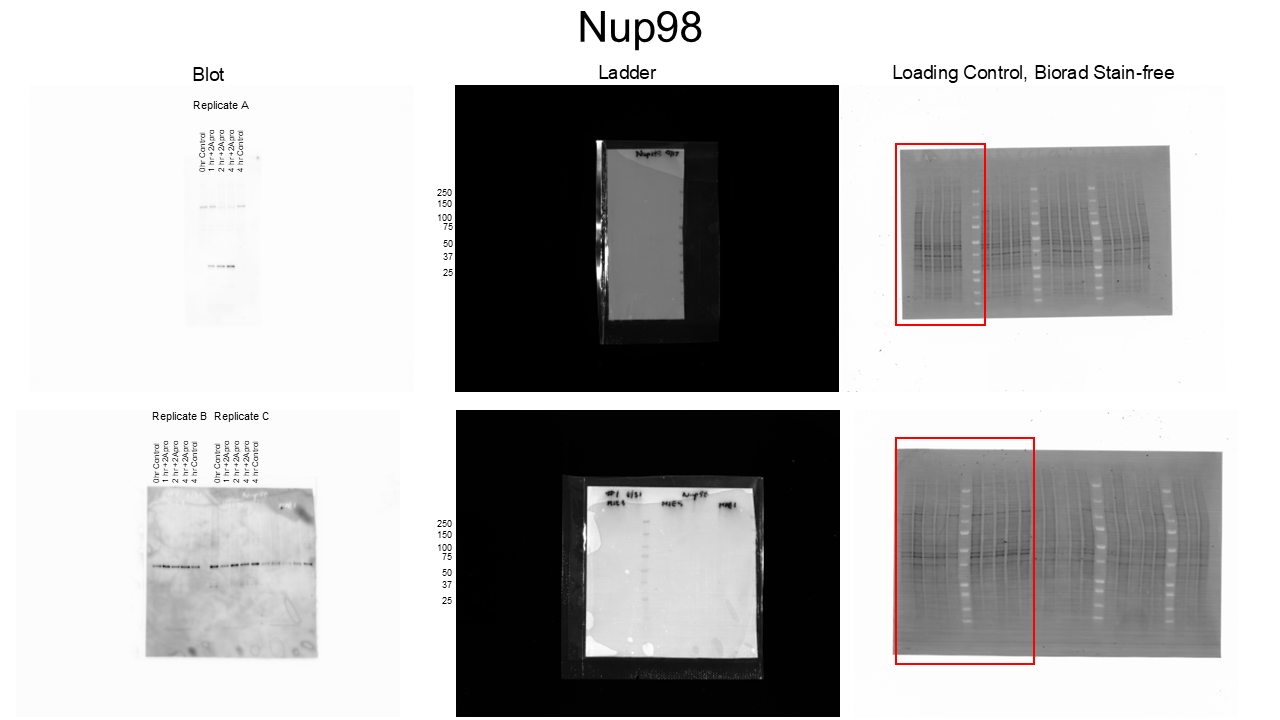

Supplement: Figure 1—source data 2. [file elife-108672-fig1-data2.zip › Figure 1C western blots/Nup98.TIF]

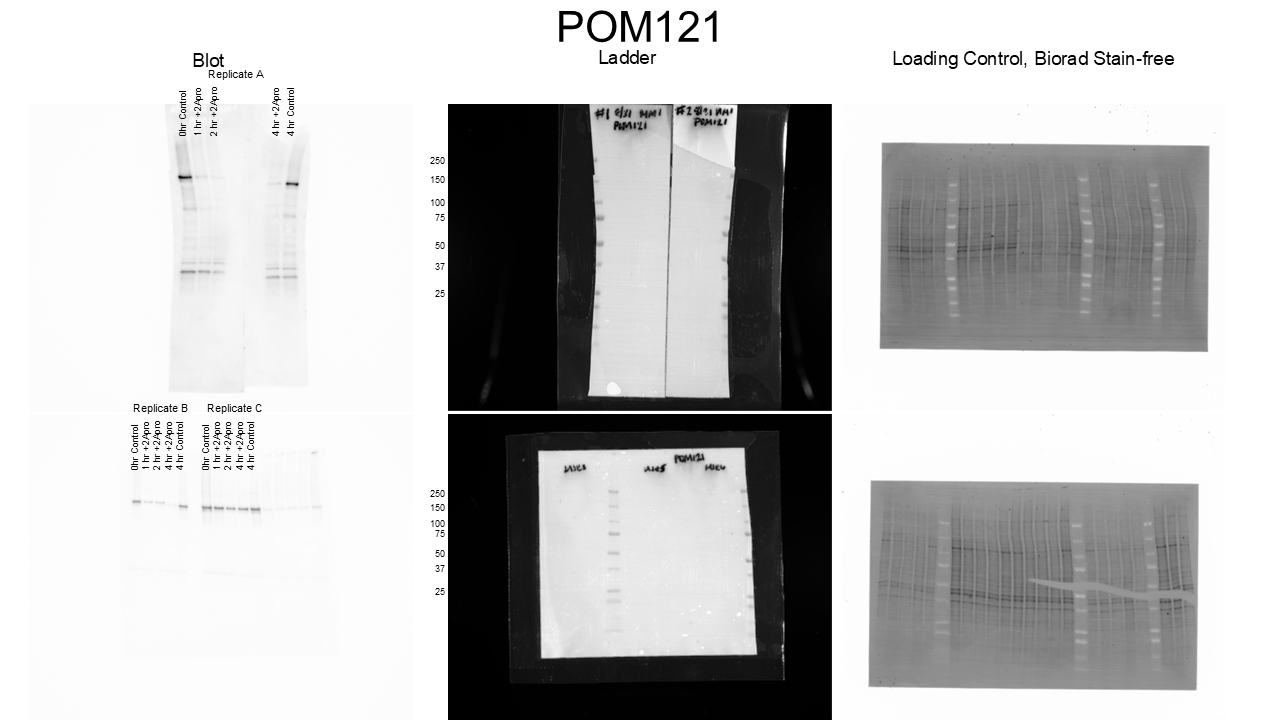

Supplement: Figure 1—source data 2. [file elife-108672-fig1-data2.zip › Figure 1C western blots/POM121.TIF]

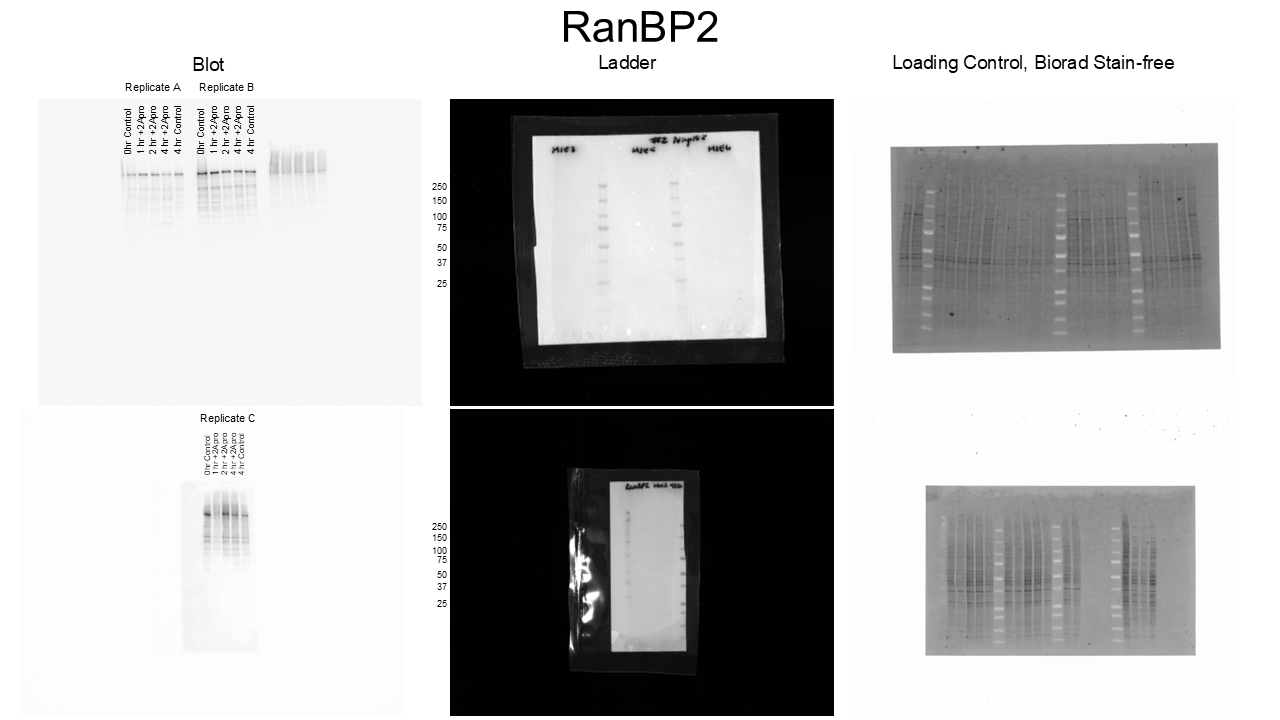

Supplement: Figure 1—source data 2. [file elife-108672-fig1-data2.zip › Figure 1C western blots/RanBP2.TIF]

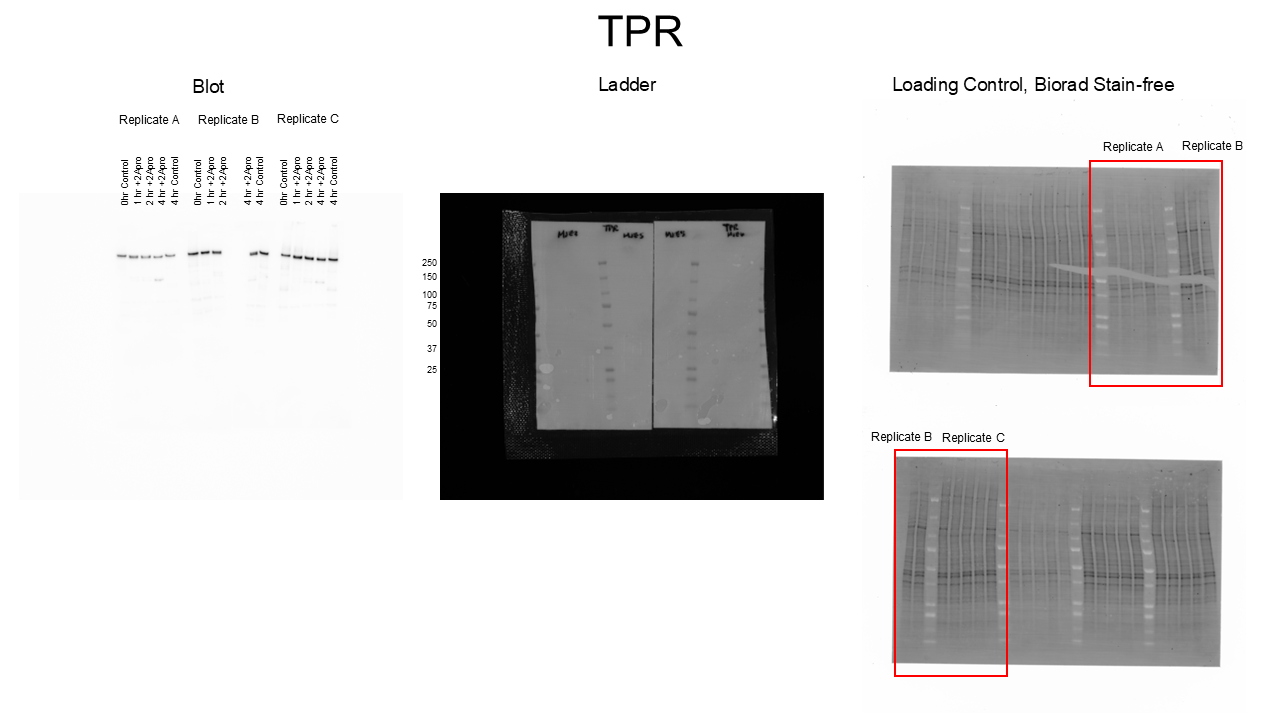

Supplement: Figure 1—source data 2. [file elife-108672-fig1-data2.zip › Figure 1C western blots/TPR.tif]

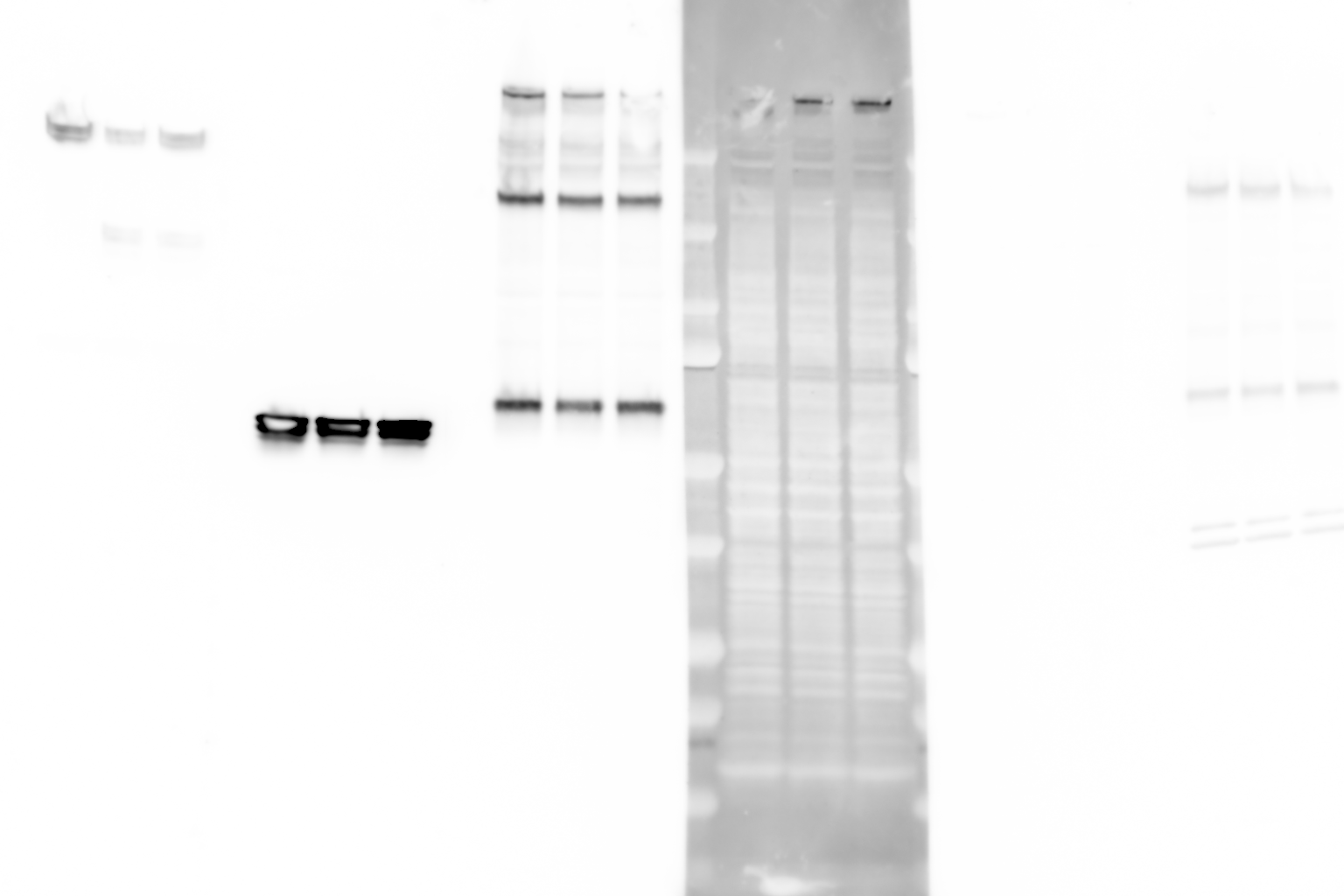

Supplement: Figure 1—source data 3. [file elife-108672-fig1-data3.zip › Fig 1A/20200923_1_15sec.tif]

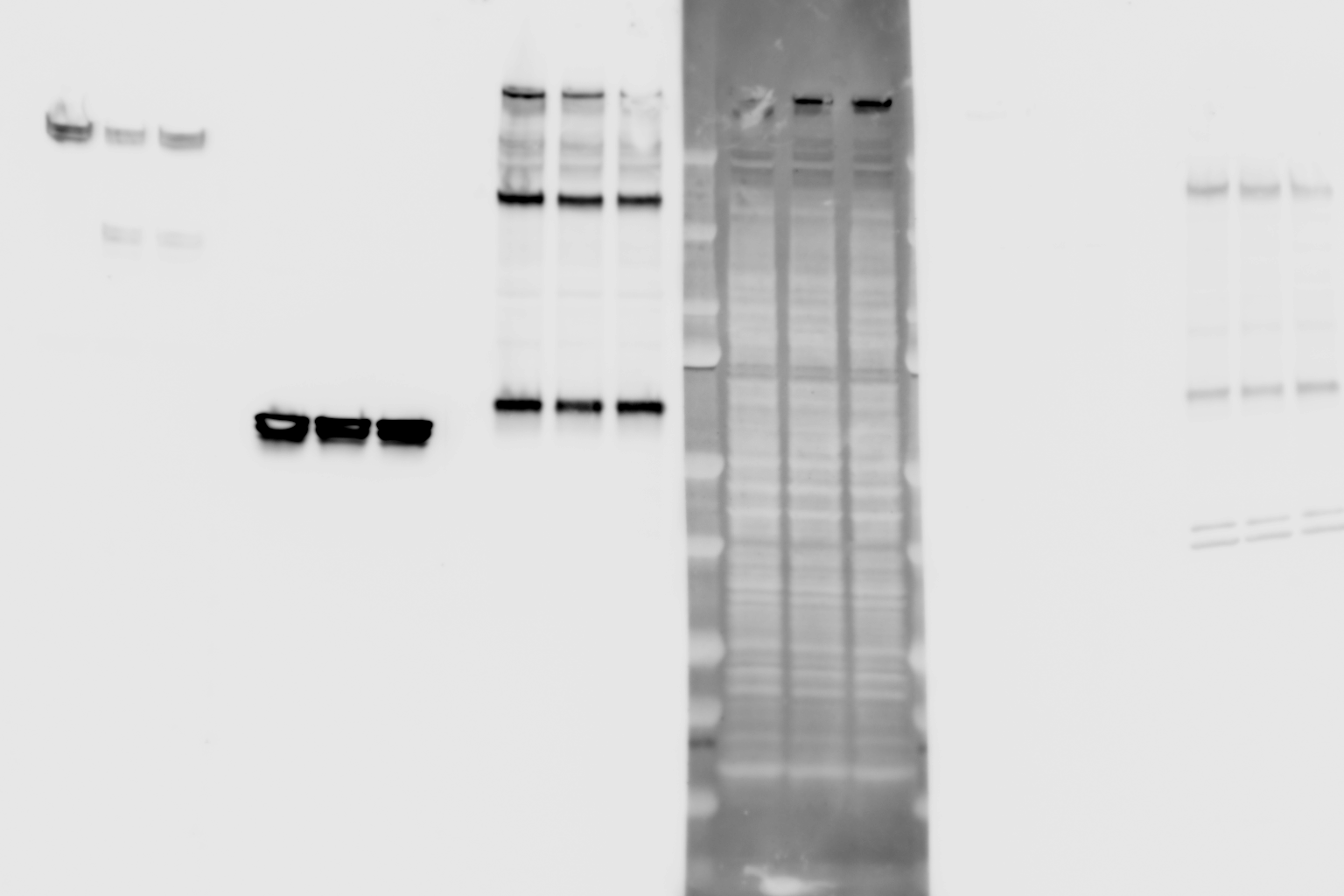

Supplement: Figure 1—source data 3. [file elife-108672-fig1-data3.zip › Fig 1A/20200923_1_15sec_Nup153.tif]

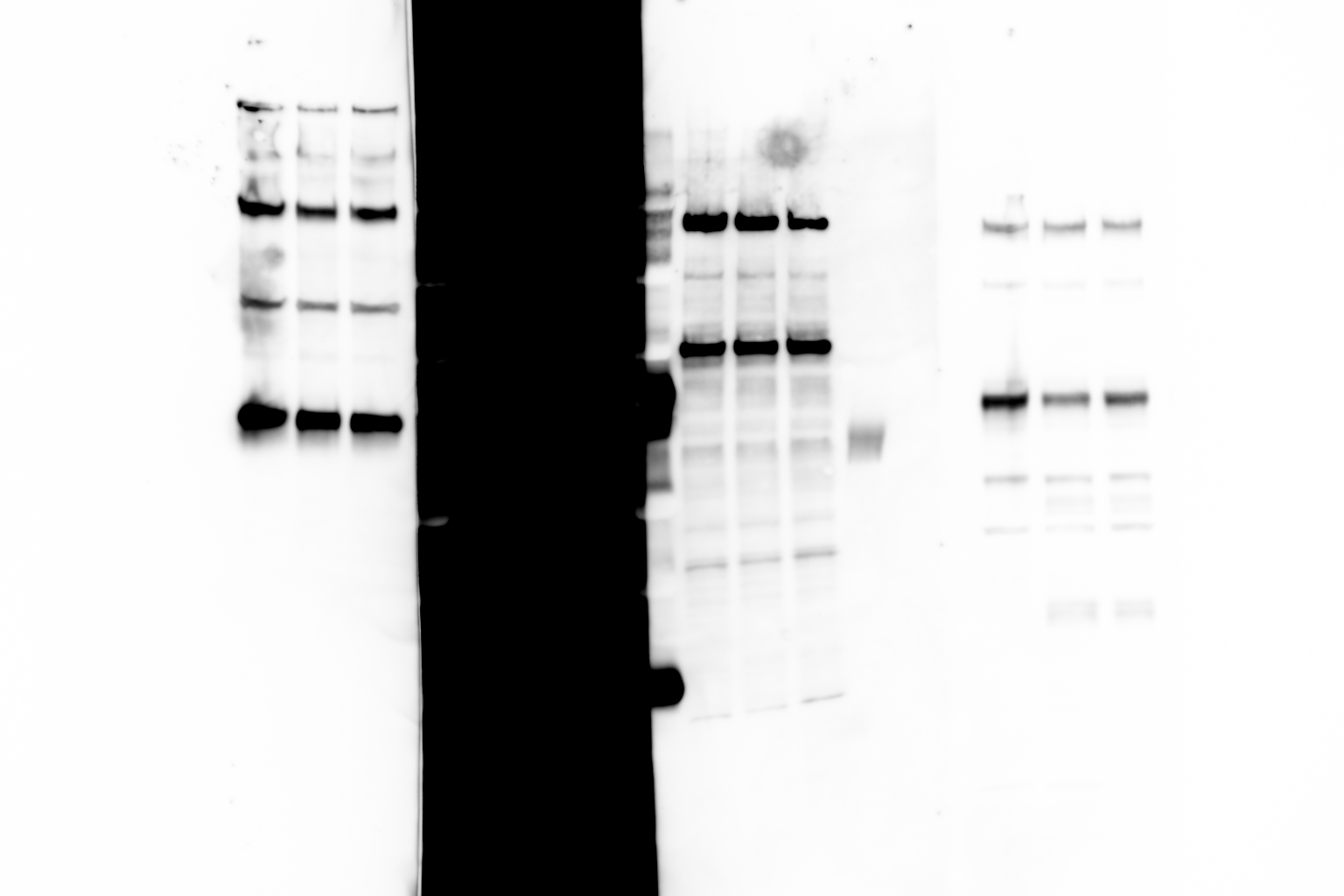

Supplement: Figure 1—source data 3. [file elife-108672-fig1-data3.zip › Fig 1A/20200923_1_5min_Nup98.tif]

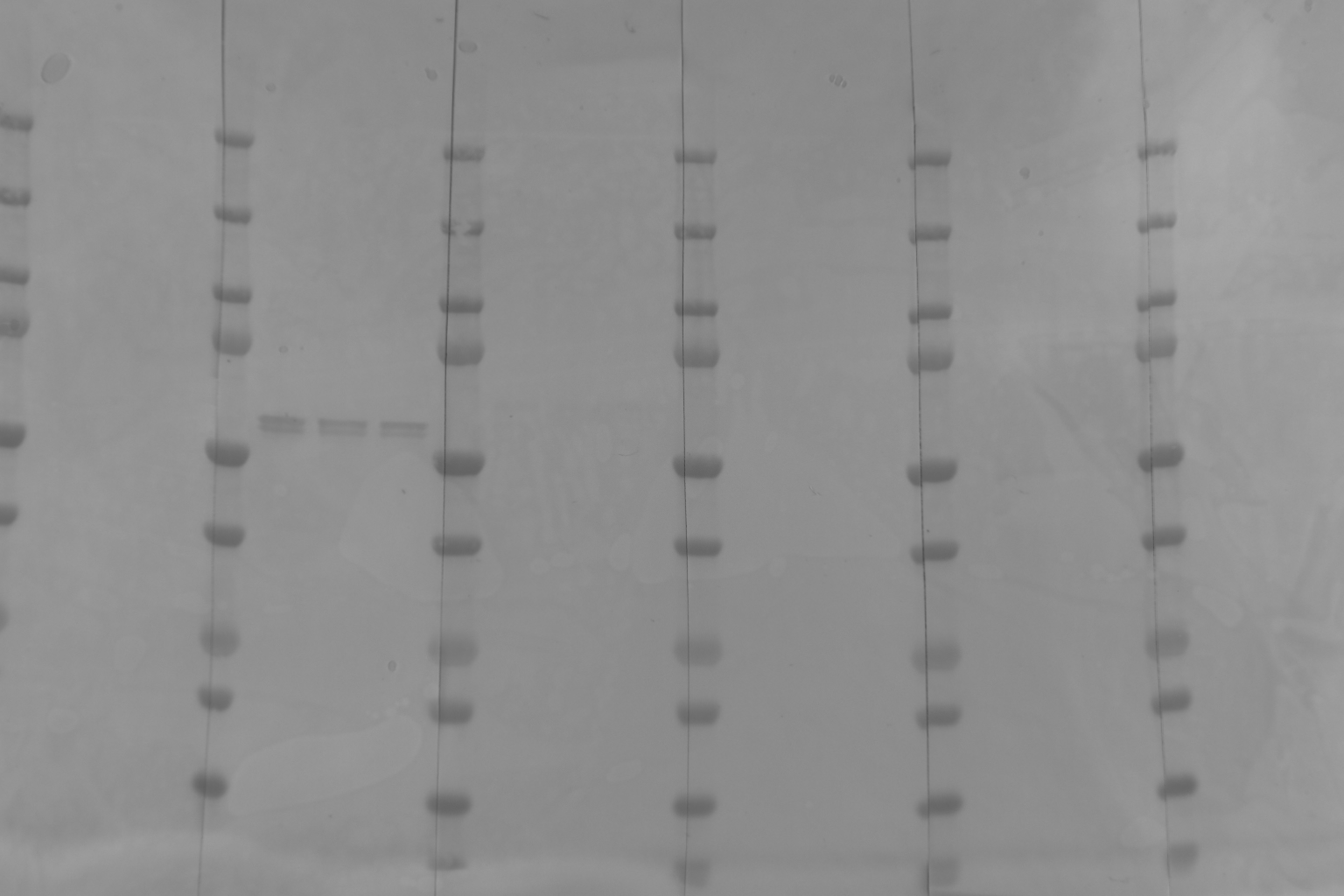

Supplement: Figure 1—source data 3. [file elife-108672-fig1-data3.zip › Fig 1A/20200923_1_ladder.tif]

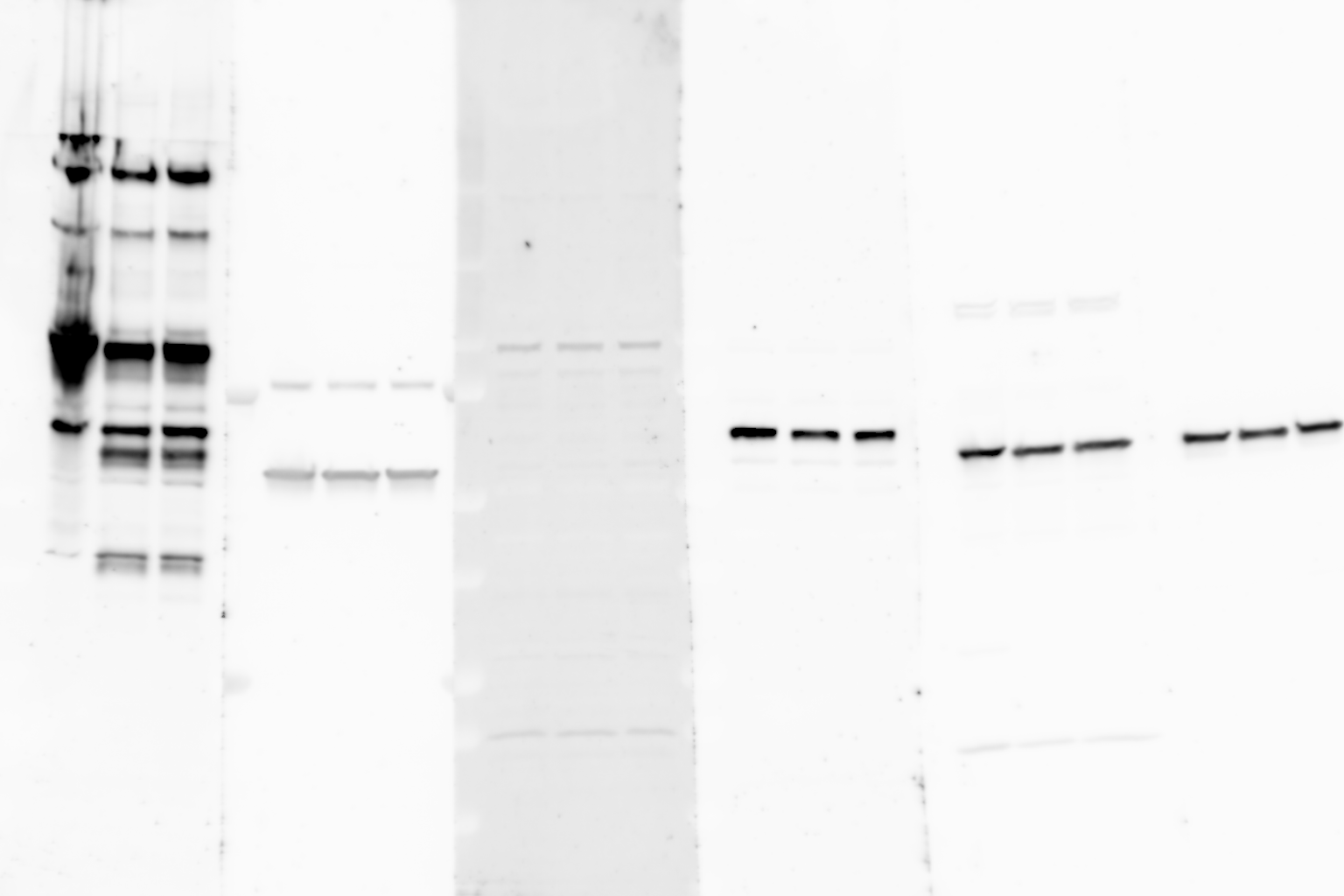

Supplement: Figure 1—source data 3. [file elife-108672-fig1-data3.zip › Fig 1A/20200923_2_15sec.tif]

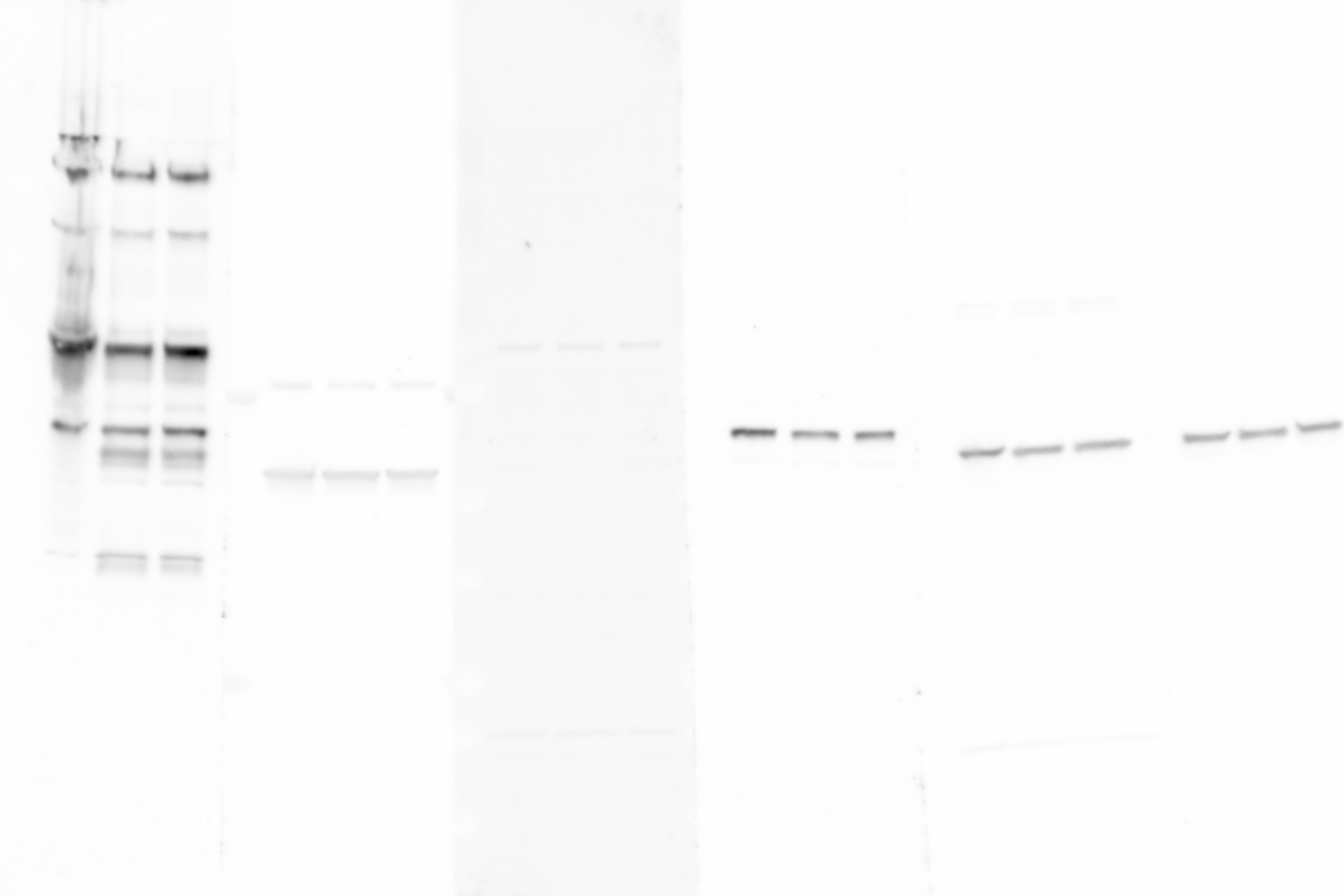

Supplement: Figure 1—source data 3. [file elife-108672-fig1-data3.zip › Fig 1A/20200923_2_1sec.tif]

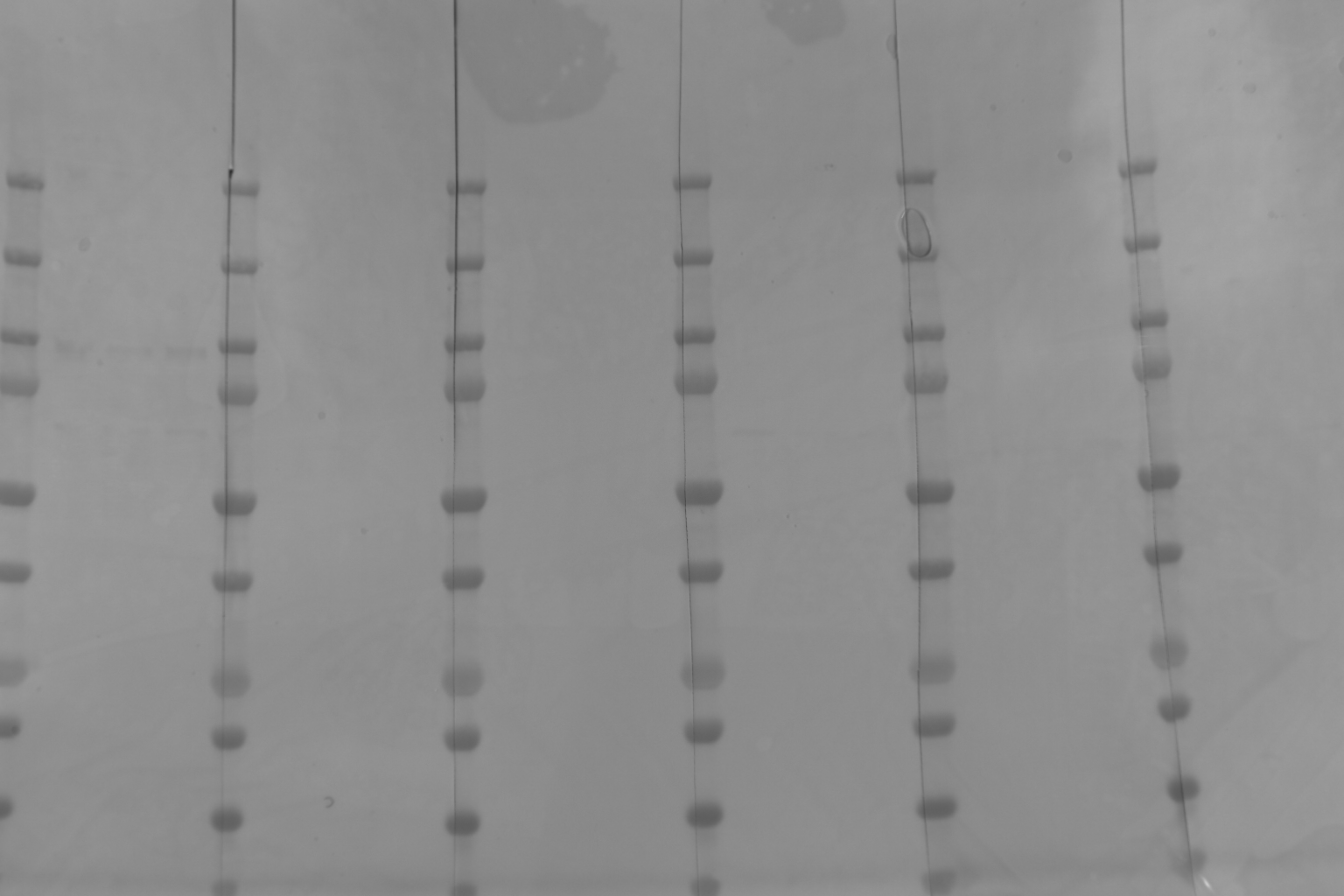

Supplement: Figure 1—source data 3. [file elife-108672-fig1-data3.zip › Fig 1A/20200923_2_ladder.tif]

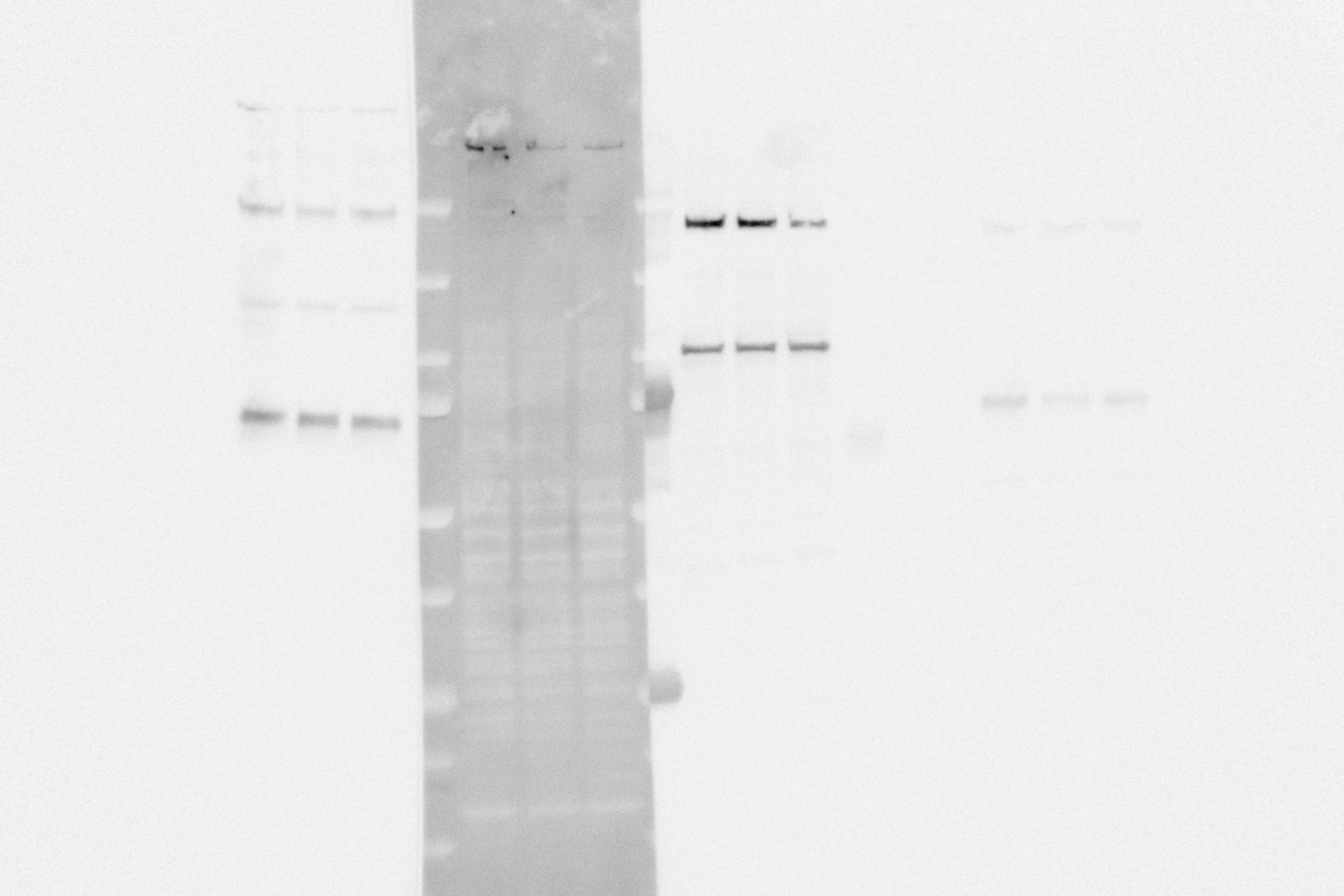

Supplement: Figure 1—source data 3. [file elife-108672-fig1-data3.zip › Fig 1A/20200924_1_1sec.tif]

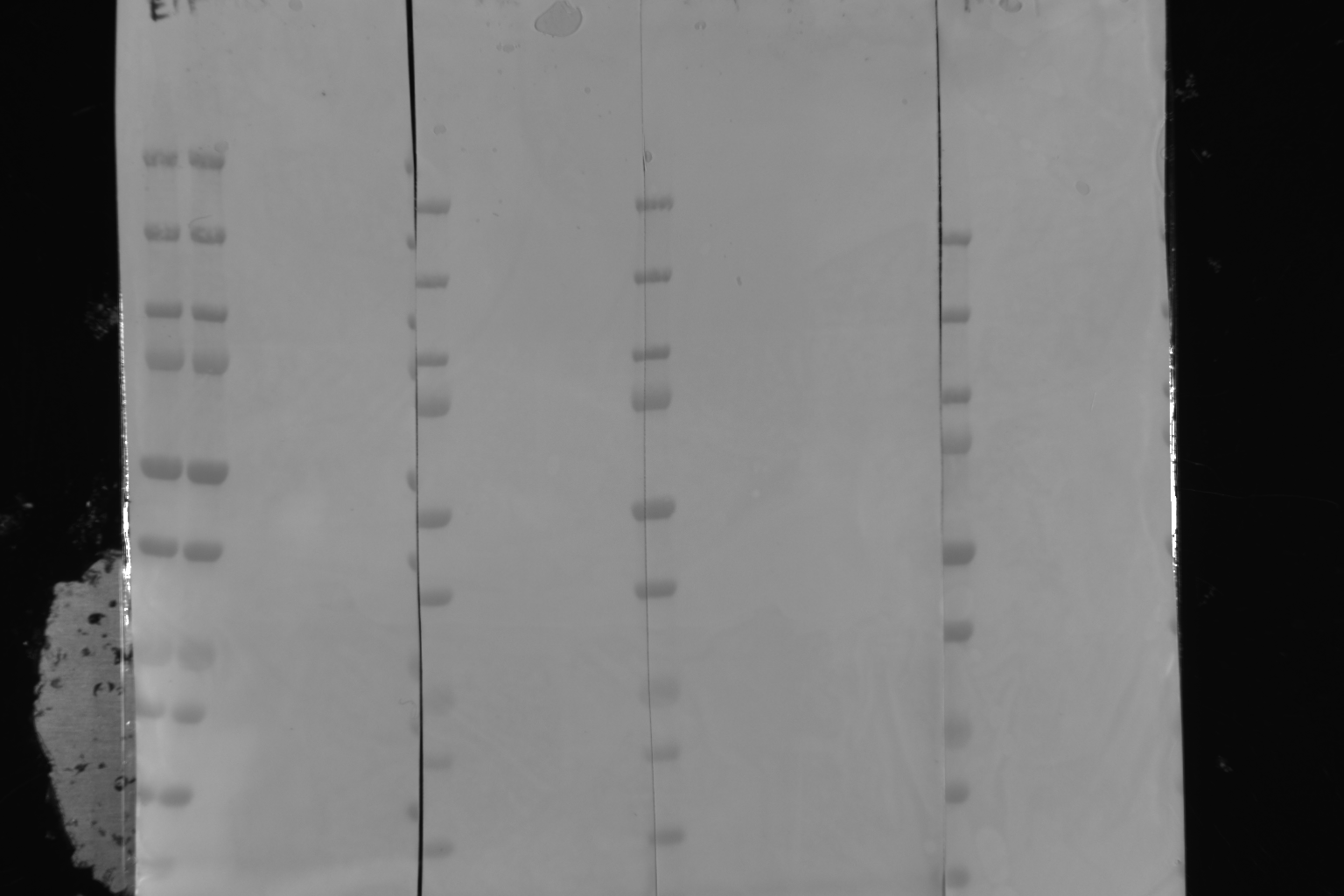

Supplement: Figure 1—source data 3. [file elife-108672-fig1-data3.zip › Fig 1A/20200924_1_ladder.tif]

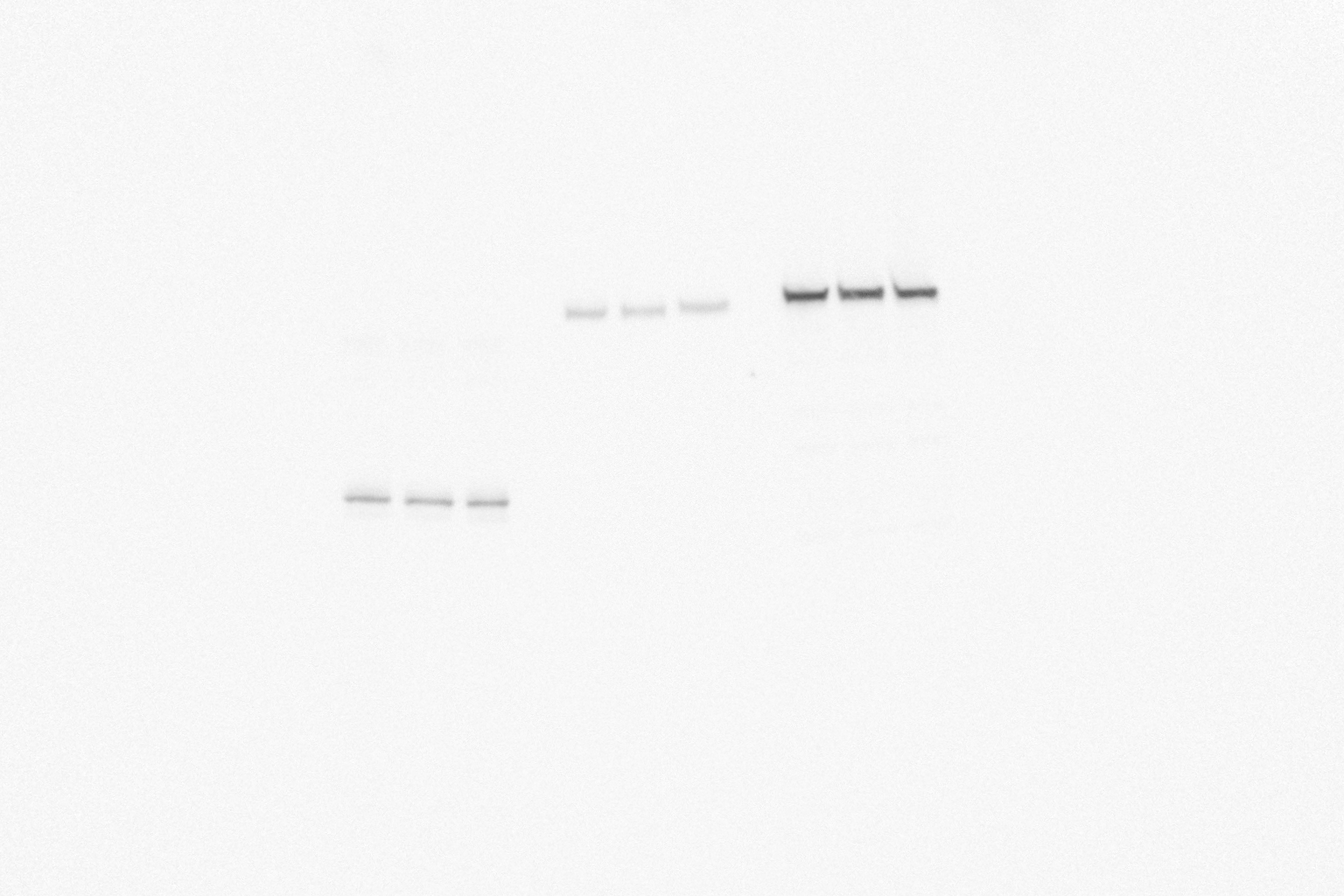

Supplement: Figure 1—source data 3. [file elife-108672-fig1-data3.zip › Fig 1A/20200924_2_1sec_Nup155.tif]

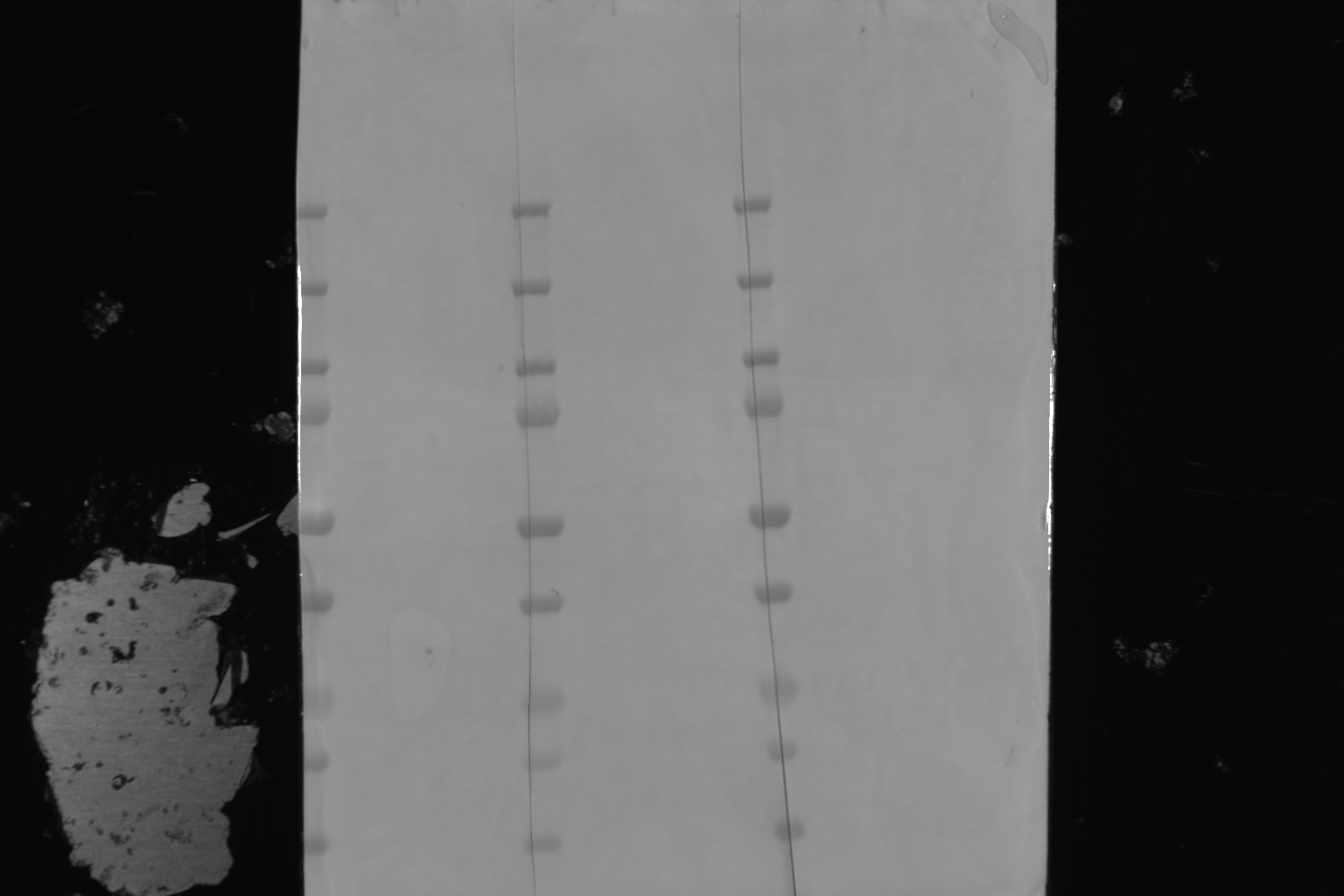

Supplement: Figure 1—source data 3. [file elife-108672-fig1-data3.zip › Fig 1A/20200924_2_ladder.tif]

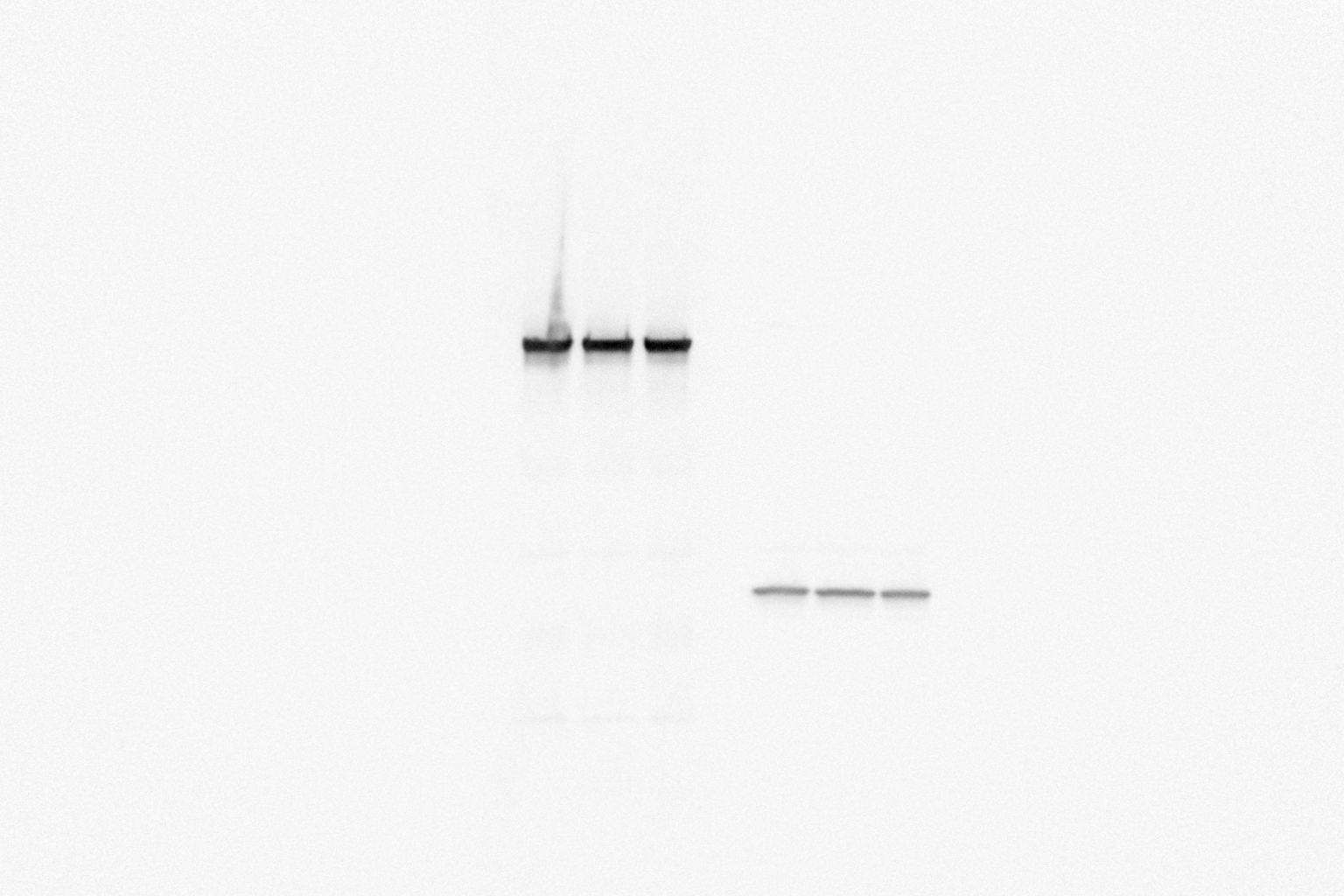

Supplement: Figure 1—source data 3. [file elife-108672-fig1-data3.zip › Fig 1A/20200924_3_500msec.tif]

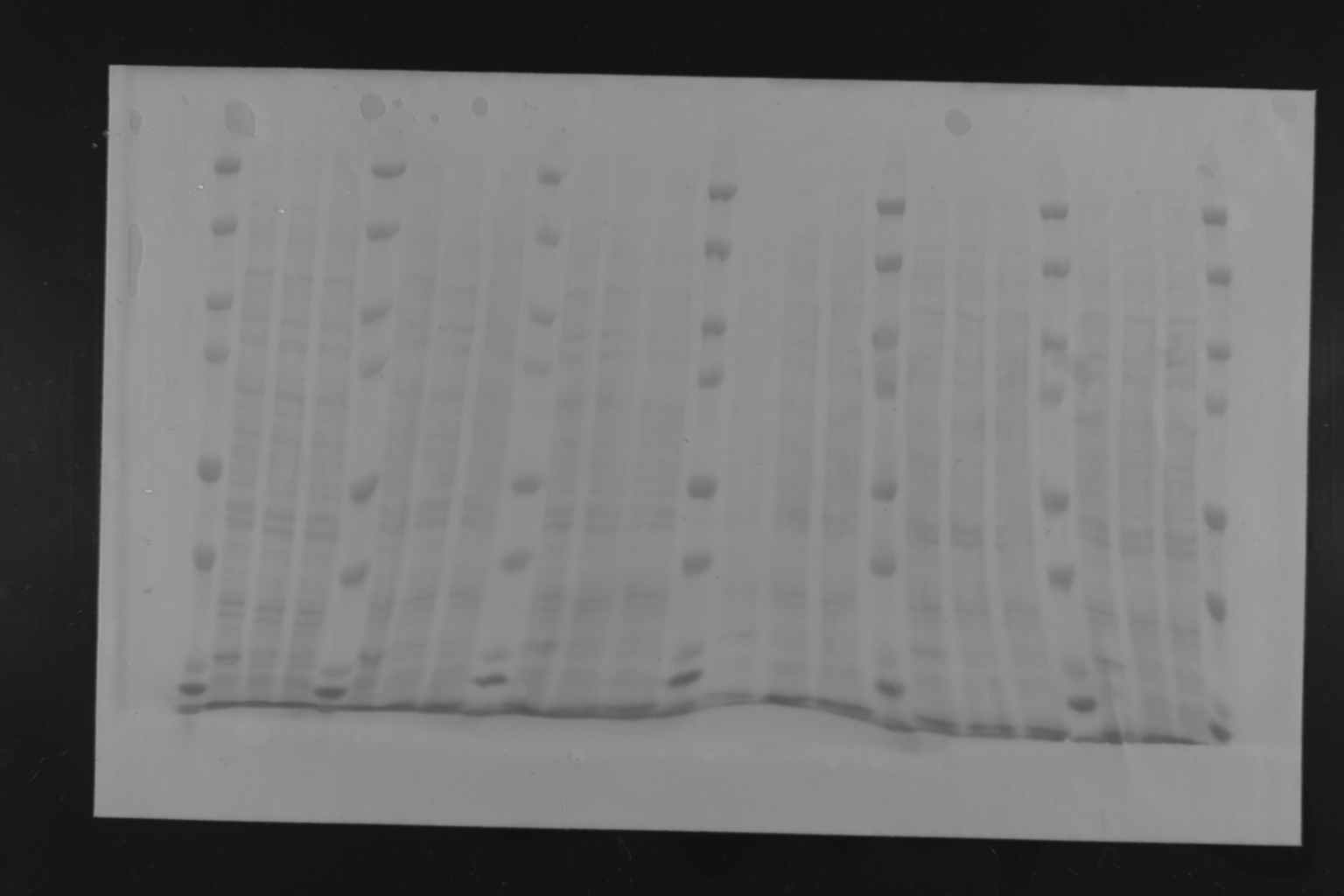

Supplement: Figure 1—source data 4. [file elife-108672-fig1-data4.zip › Fig 1B (part 1)/20210315_blot1 Ponceau.tif]

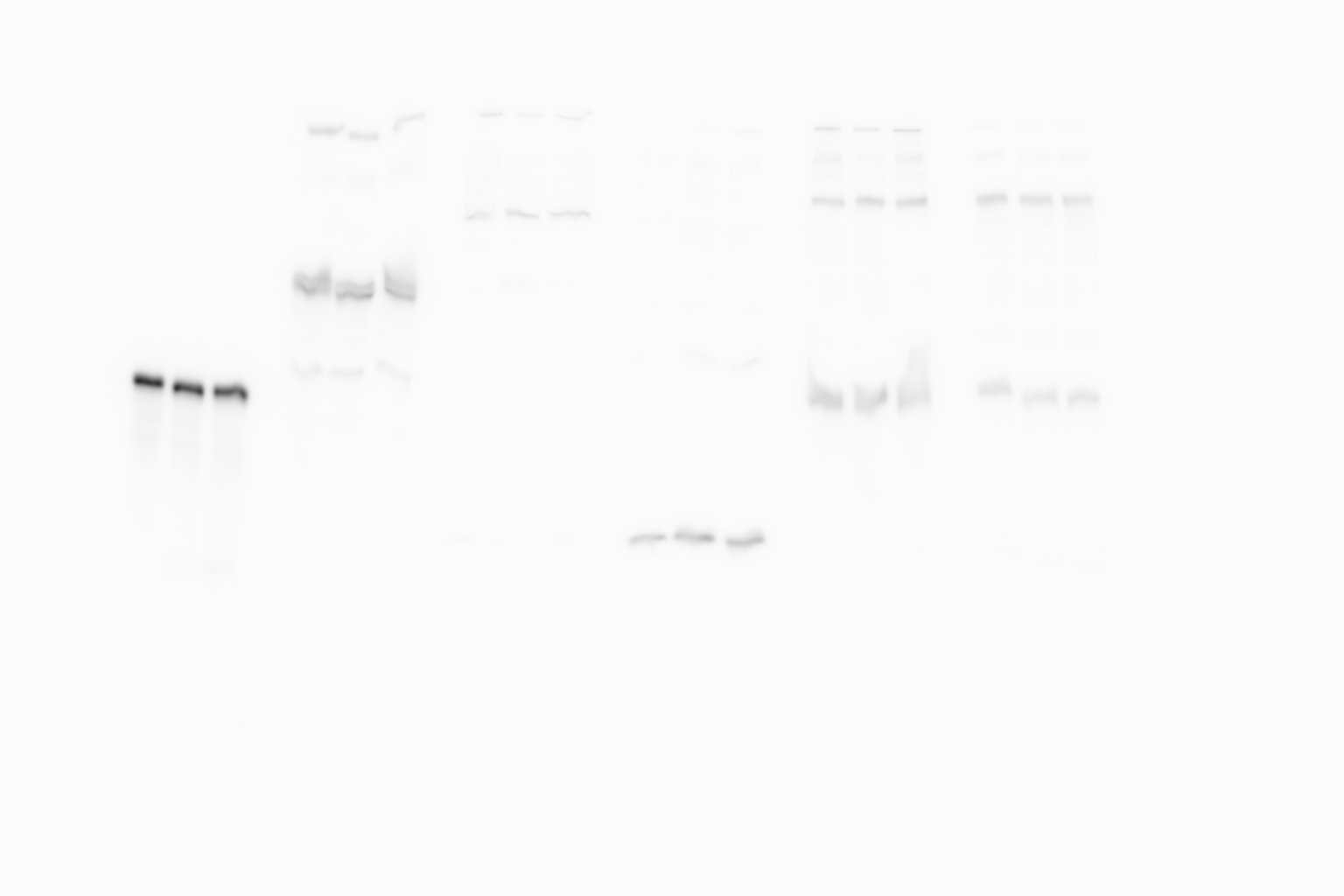

Supplement: Figure 1—source data 4. [file elife-108672-fig1-data4.zip › Fig 1B (part 1)/20210316_Blot1 15sec.tif]

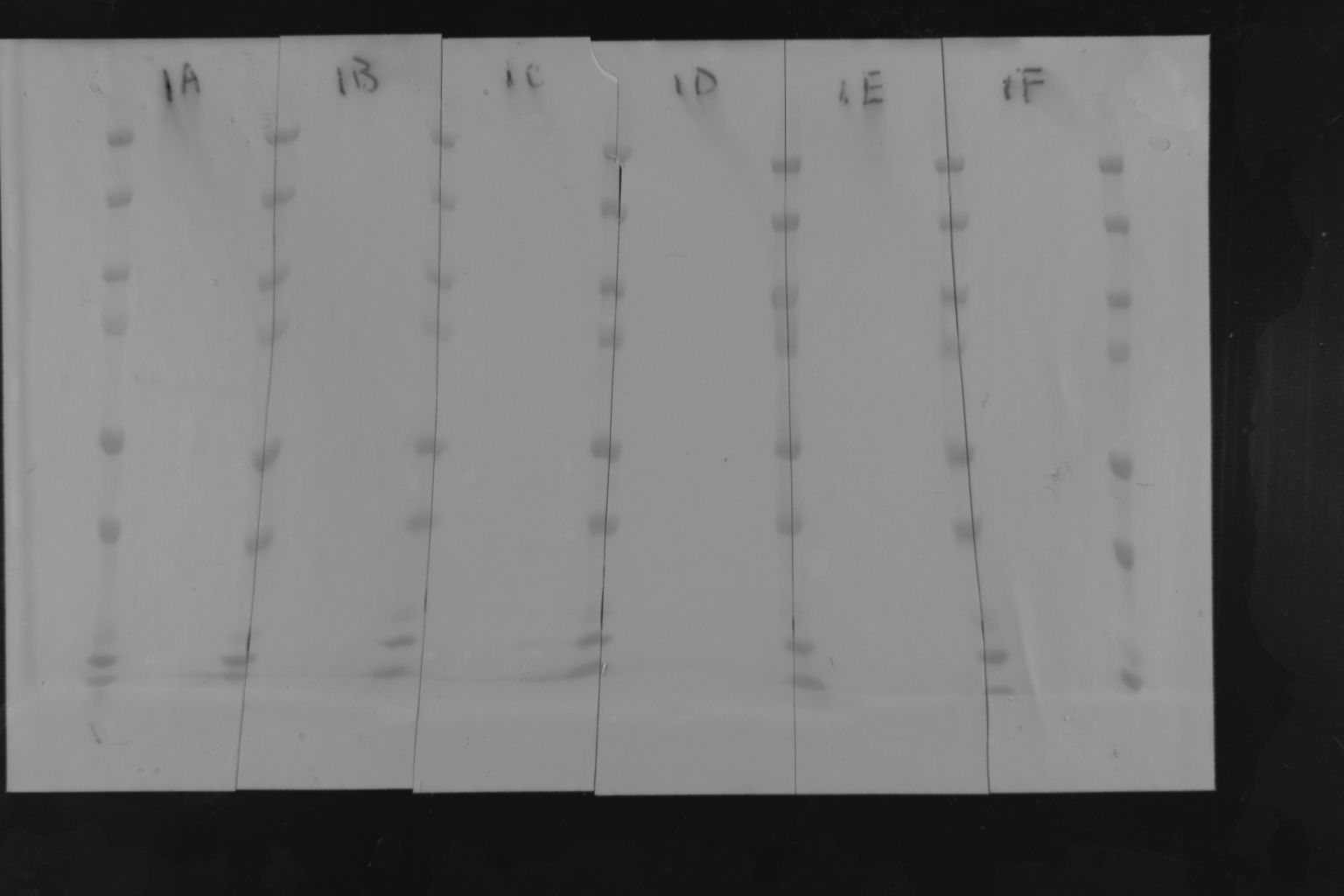

Supplement: Figure 1—source data 4. [file elife-108672-fig1-data4.zip › Fig 1B (part 1)/20210316_Blot1 Ladder.tif]

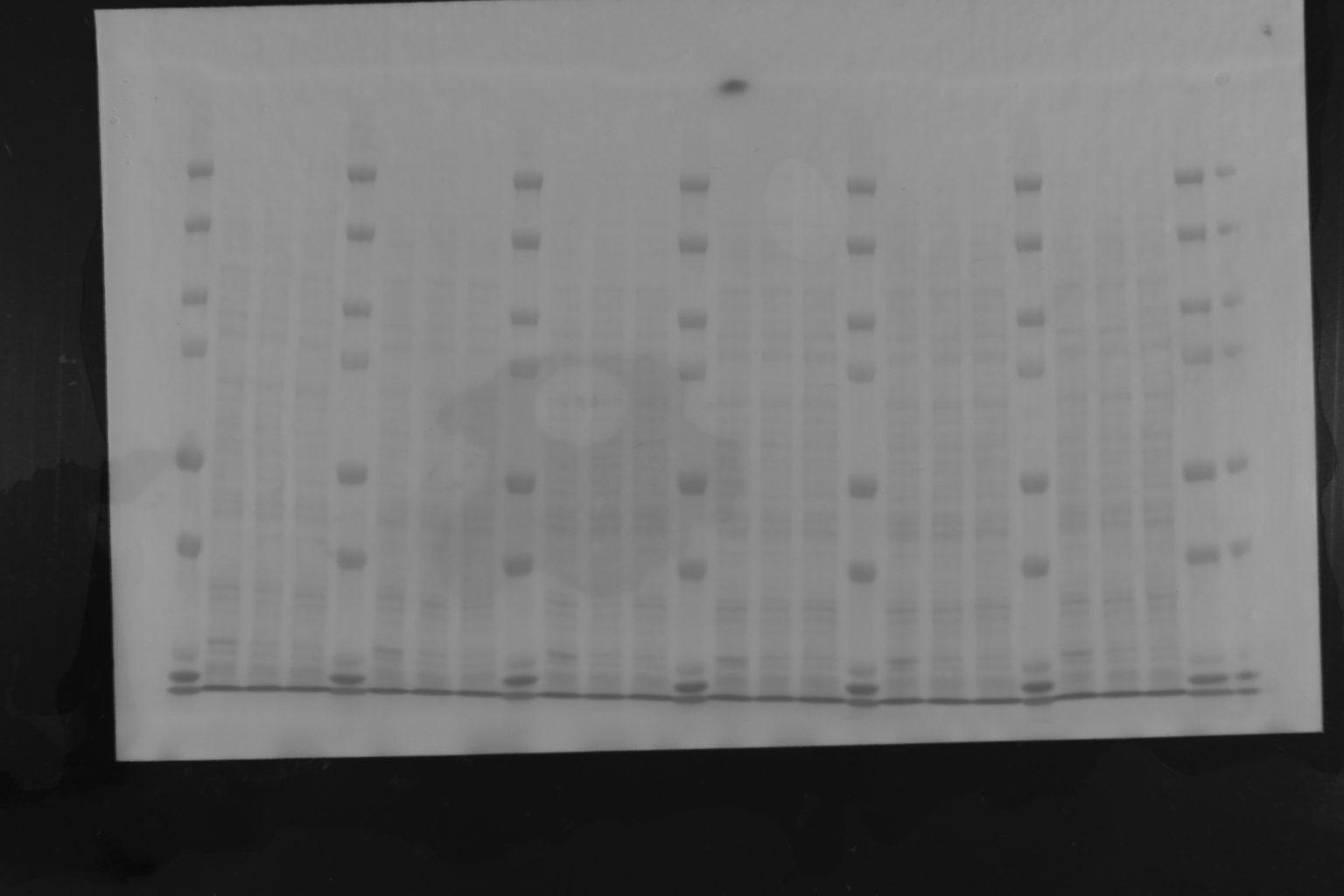

Supplement: Figure 1—source data 4. [file elife-108672-fig1-data4.zip › Fig 1B (part 1)/20210316_Blot2 Ponceau S.tif]

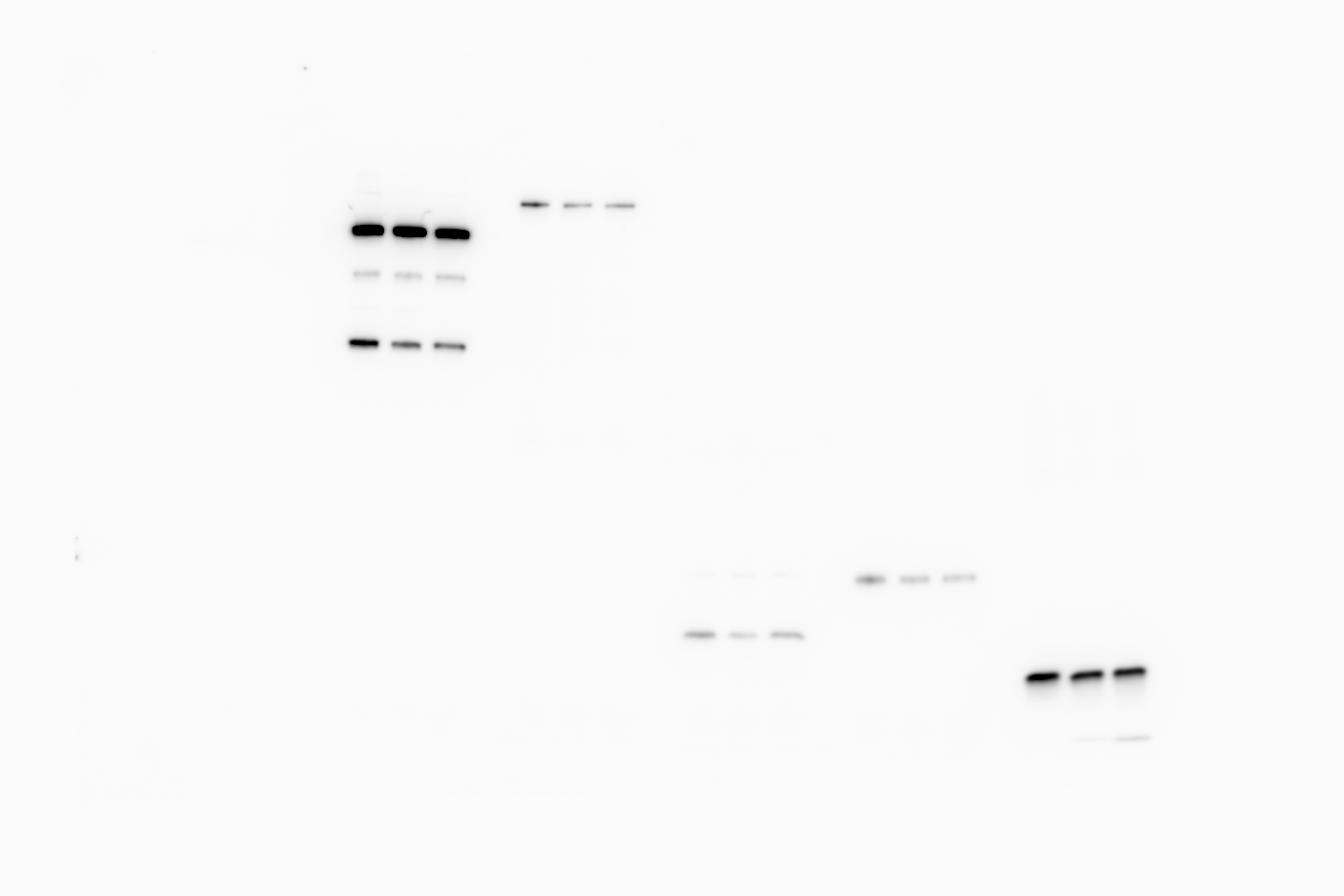

Supplement: Figure 1—source data 4. [file elife-108672-fig1-data4.zip › Fig 1B (part 1)/20210317_blot2 10min.tif]

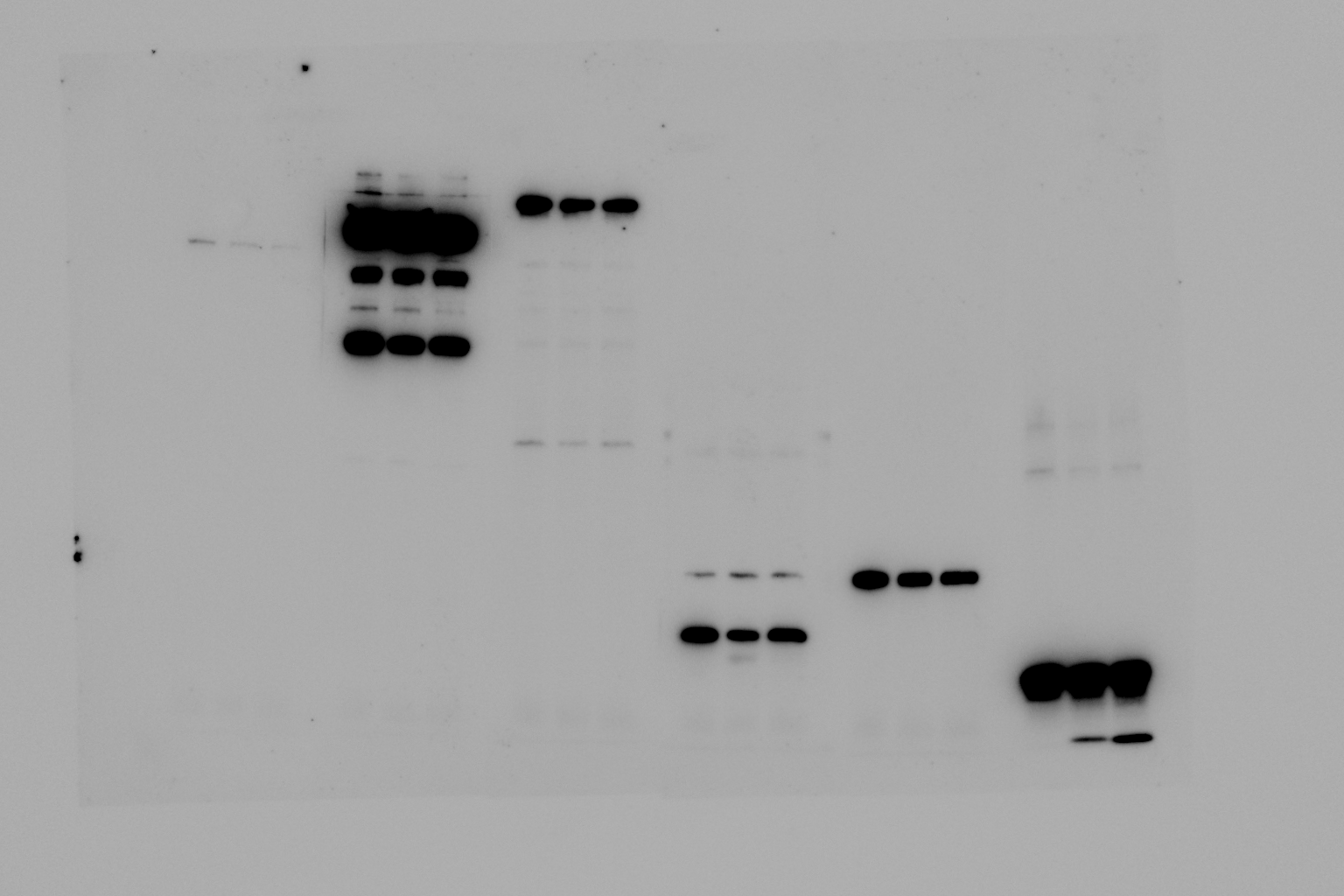

Supplement: Figure 1—source data 4. [file elife-108672-fig1-data4.zip › Fig 1B (part 1)/20210317_blot2 10min_Nup188.tif]
