# Supplementary figures and images for: Enterovirus D68 2A protease causes nuclear pore complex dysfunction and independently contributes to motor neuron toxicity (part 2 of 4)
Source: eLife. 2026 Jun 18;14:RP108672. doi: 10.7554/eLife.108672 (PMC13278737; doi:10.7554/eLife.108672)

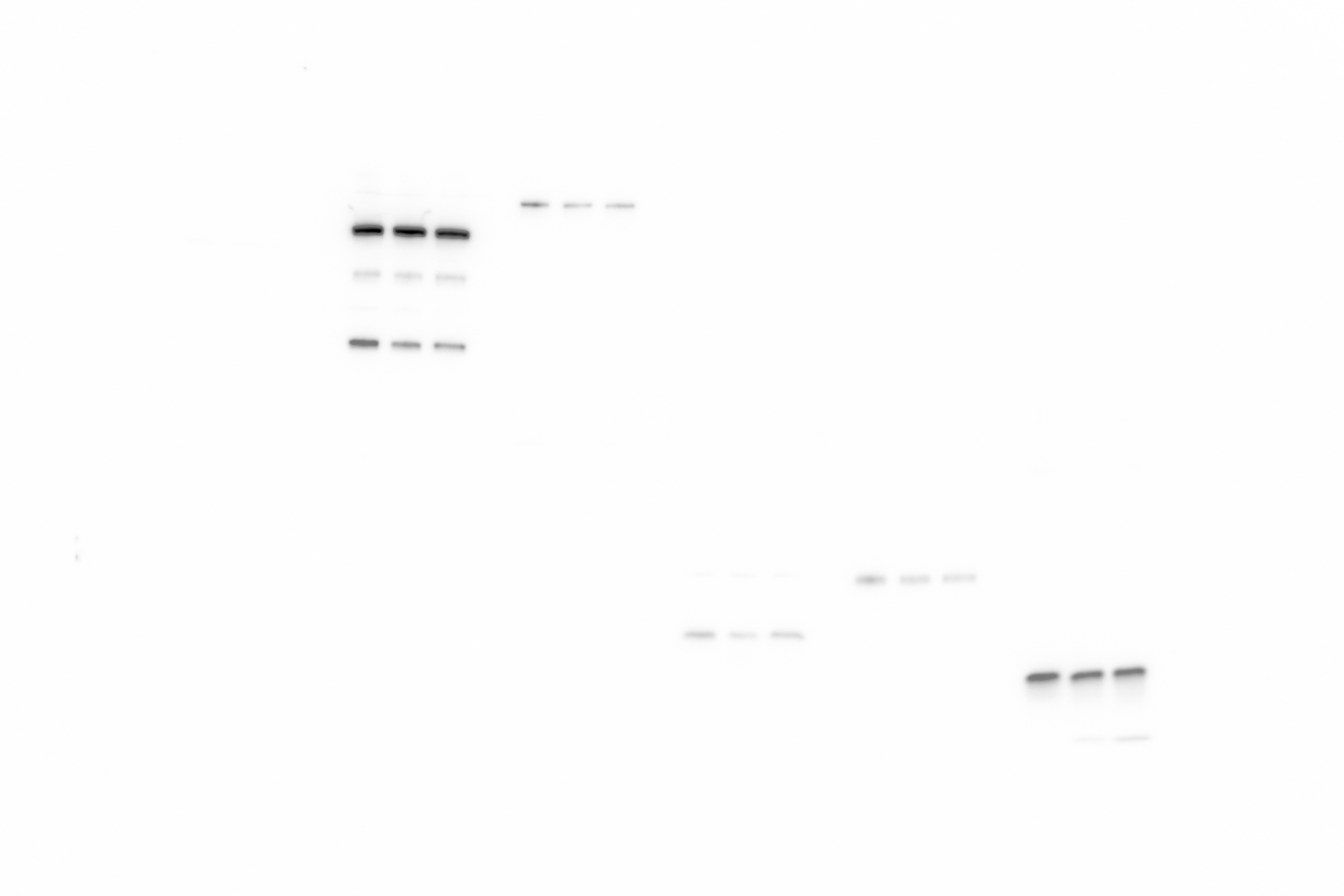

Supplement: Figure 1—source data 4. [file elife-108672-fig1-data4.zip › Fig 1B (part 1)/20210317_blot2 1min.tif]

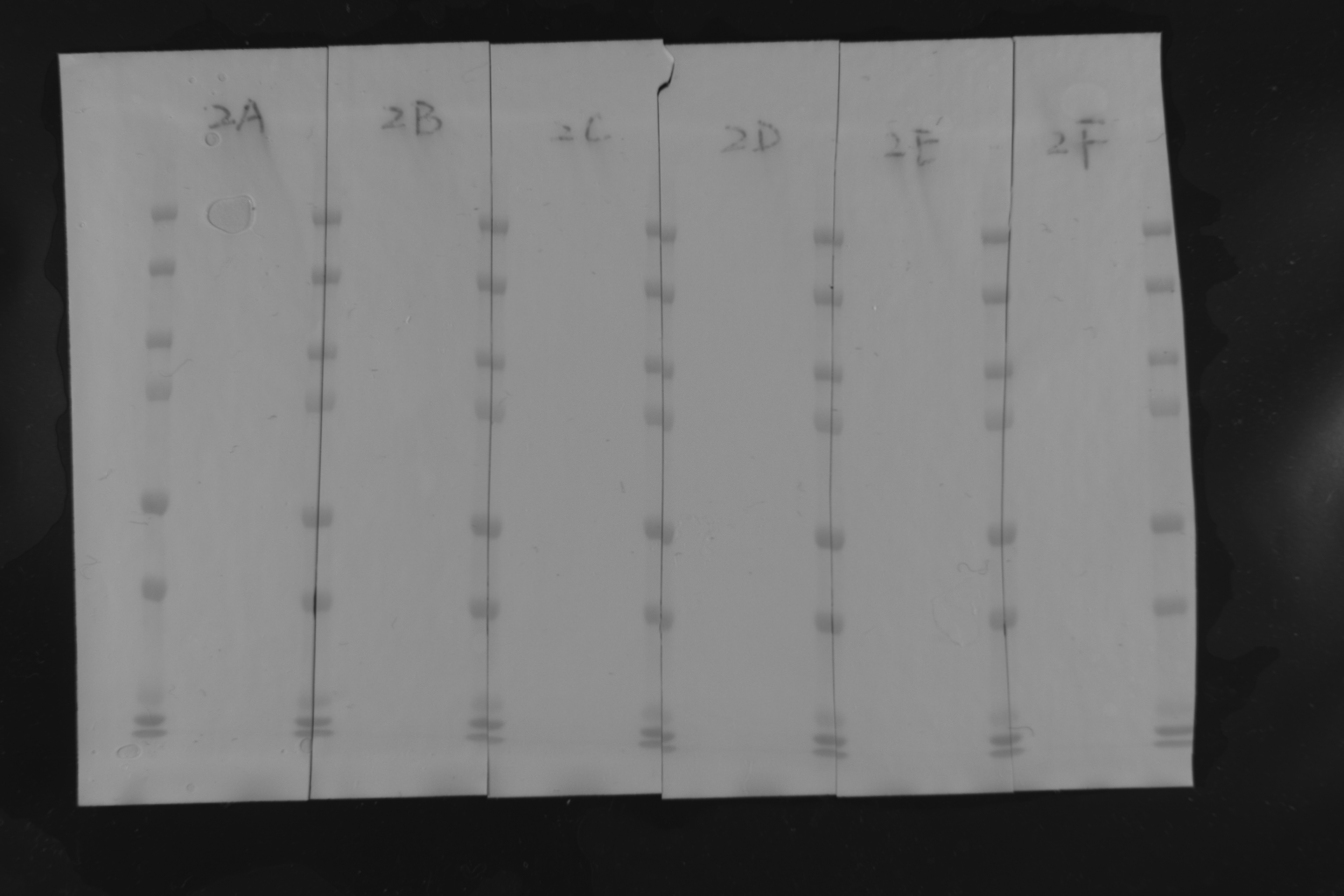

Supplement: Figure 1—source data 4. [file elife-108672-fig1-data4.zip › Fig 1B (part 1)/20210317_blot2 ladder.tif]

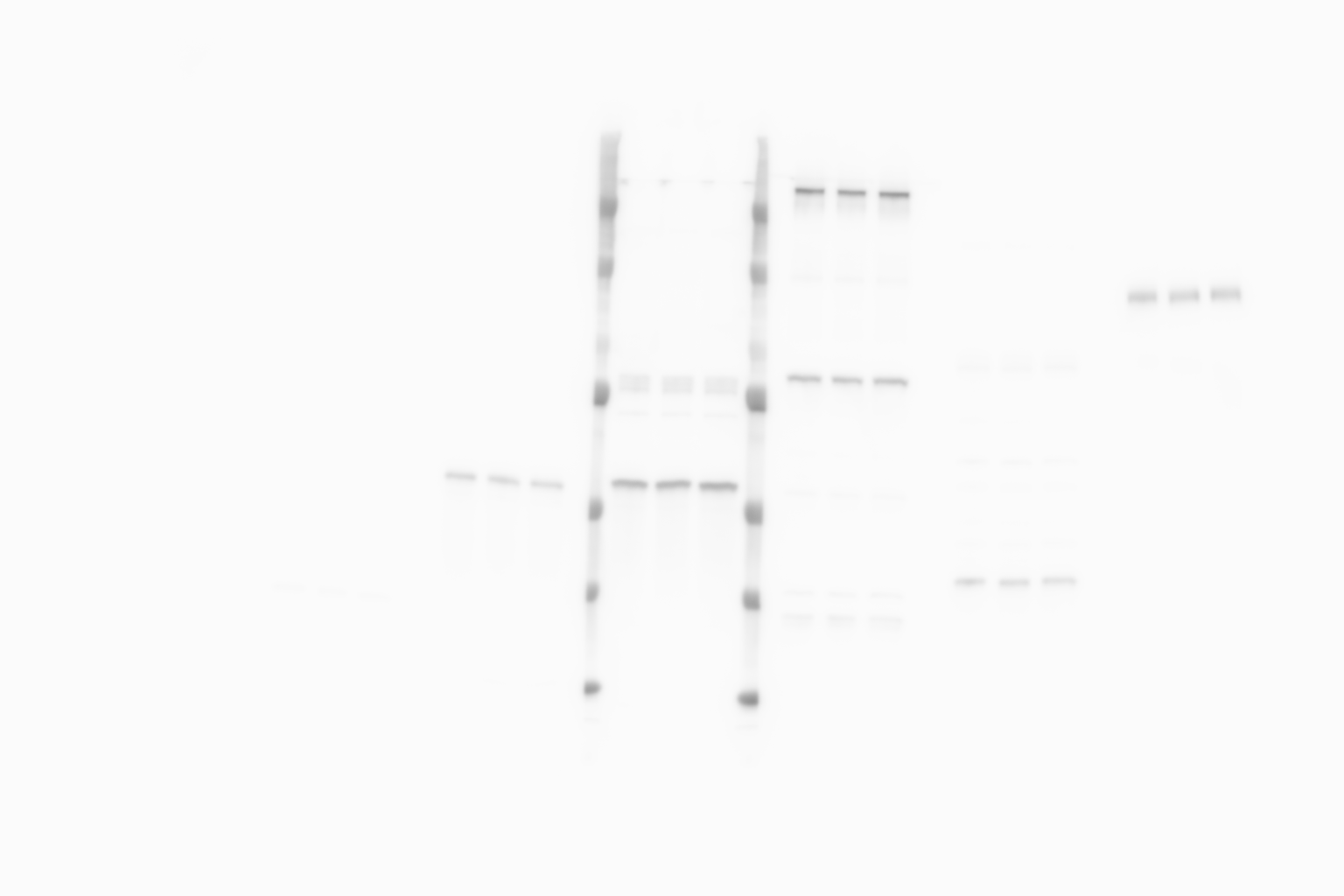

Supplement: Figure 1—source data 4. [file elife-108672-fig1-data4.zip › Fig 1B (part 1)/20210318_Blot 2 overblot 15sec.tif]

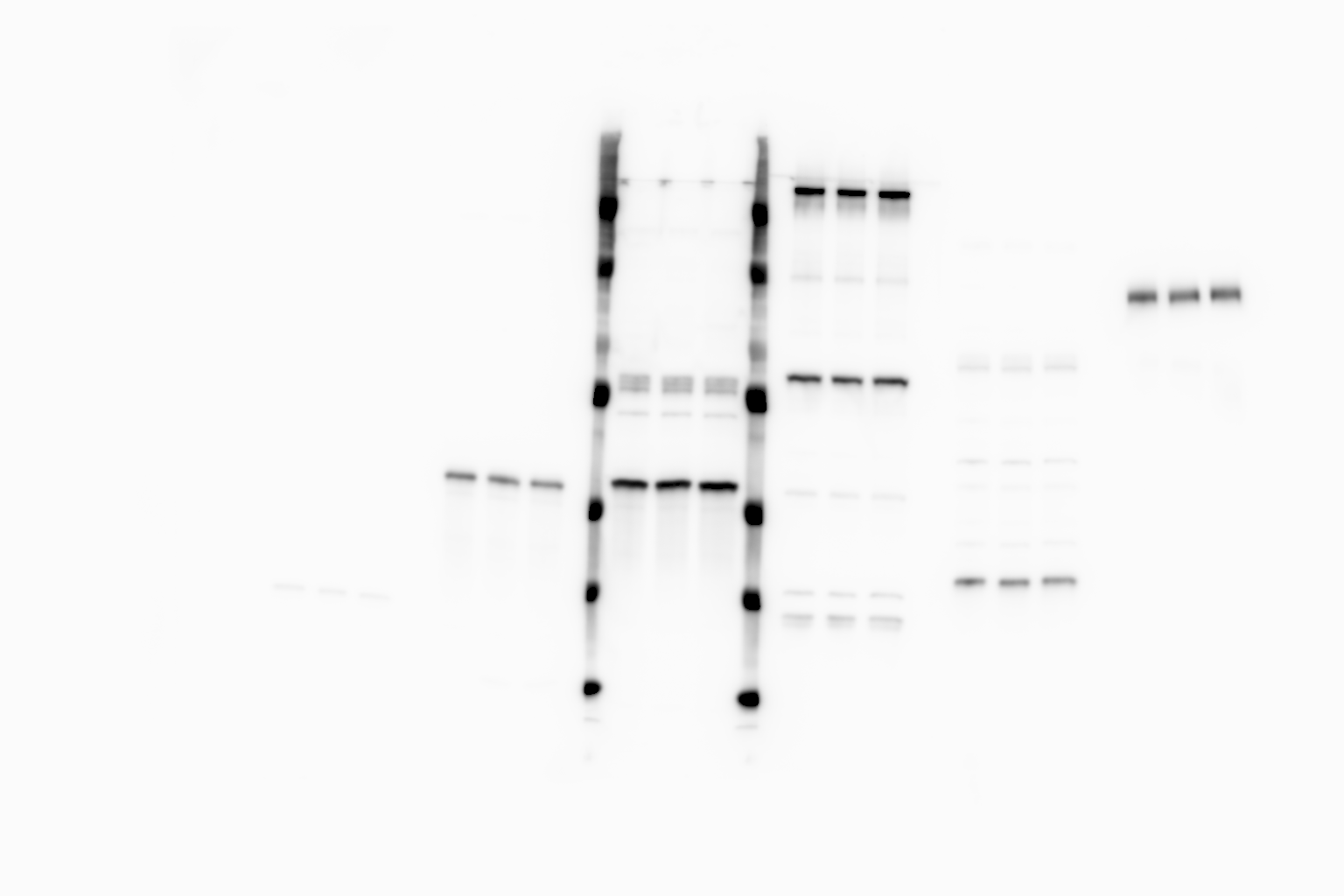

Supplement: Figure 1—source data 4. [file elife-108672-fig1-data4.zip › Fig 1B (part 1)/20210318_Blot 2 overblot 1min.tif]

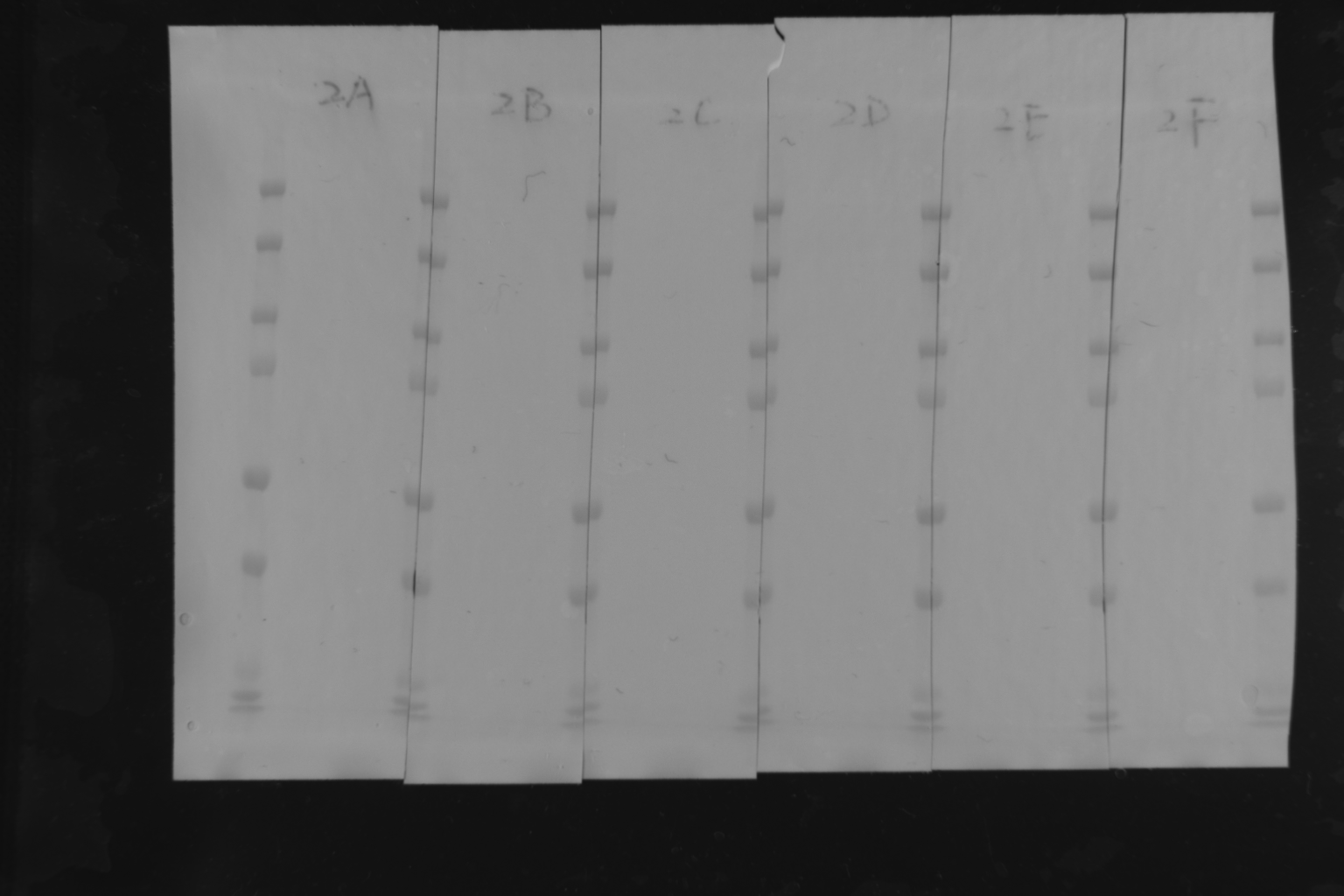

Supplement: Figure 1—source data 4. [file elife-108672-fig1-data4.zip › Fig 1B (part 1)/20210318_Blot 2 overblot ladder.tif]

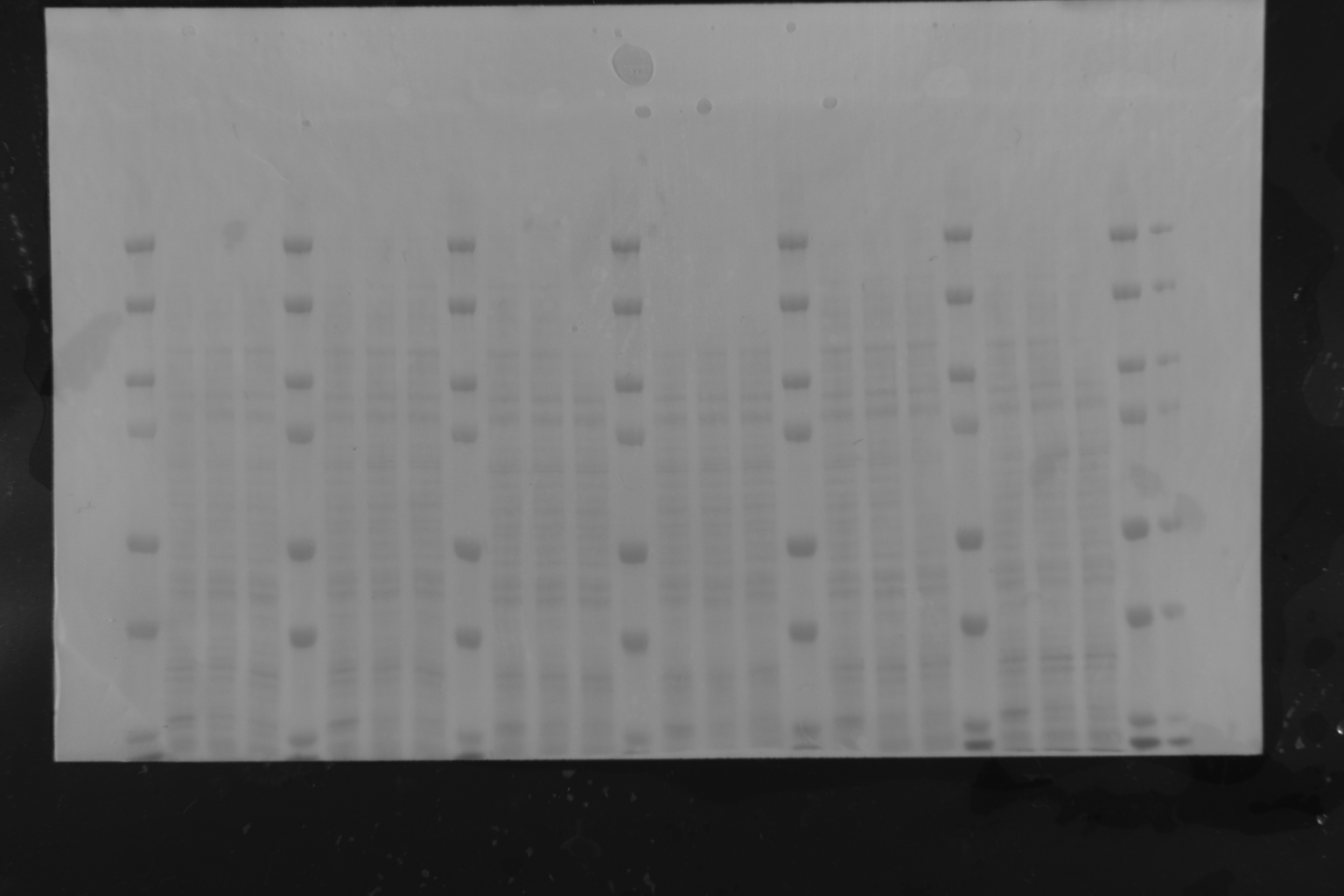

Supplement: Figure 1—source data 4. [file elife-108672-fig1-data4.zip › Fig 1B (part 1)/20210318_Blot 4 Ponceau S.tif]

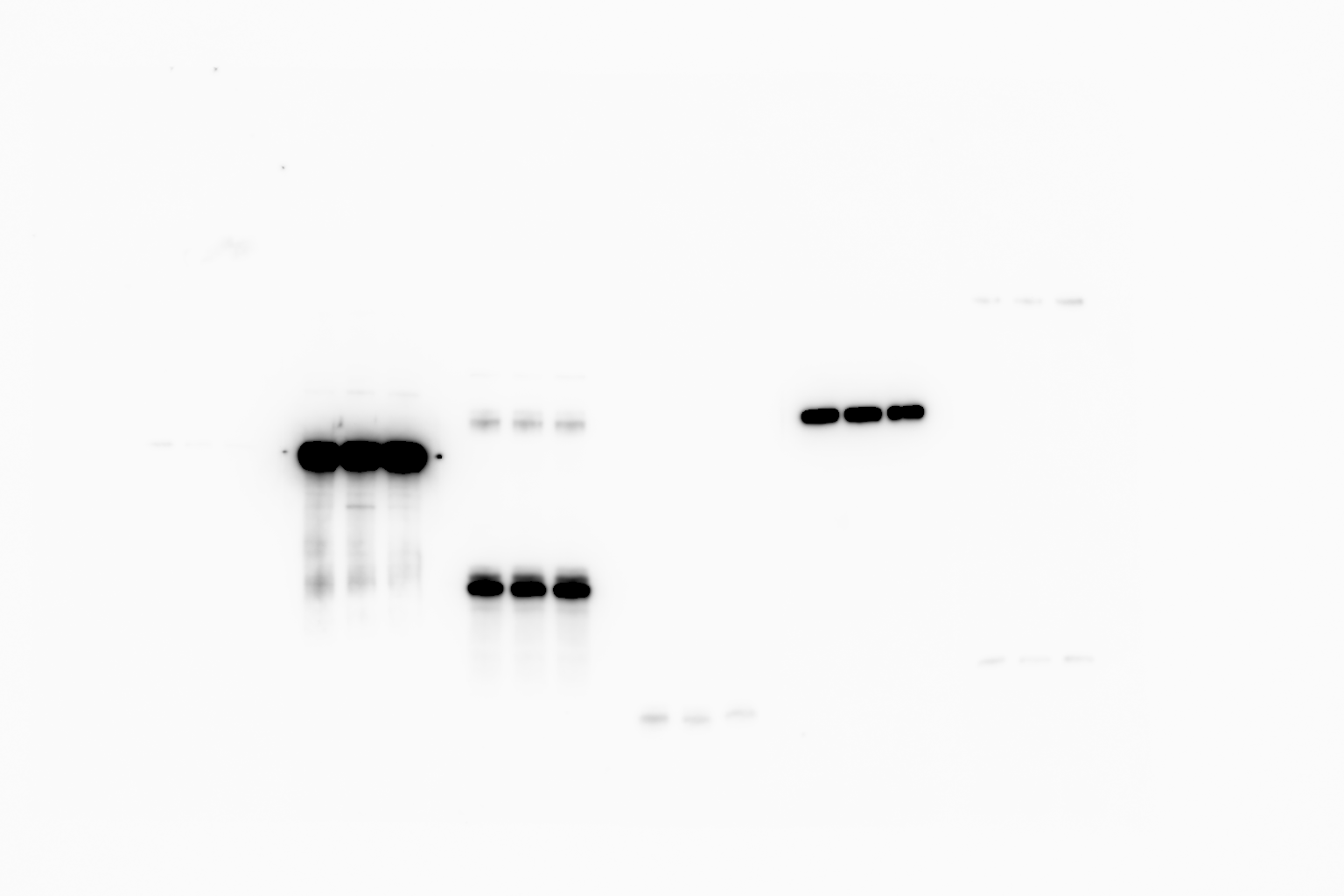

Supplement: Figure 1—source data 4. [file elife-108672-fig1-data4.zip › Fig 1B (part 1)/20210319_Blot4 10min.tif]

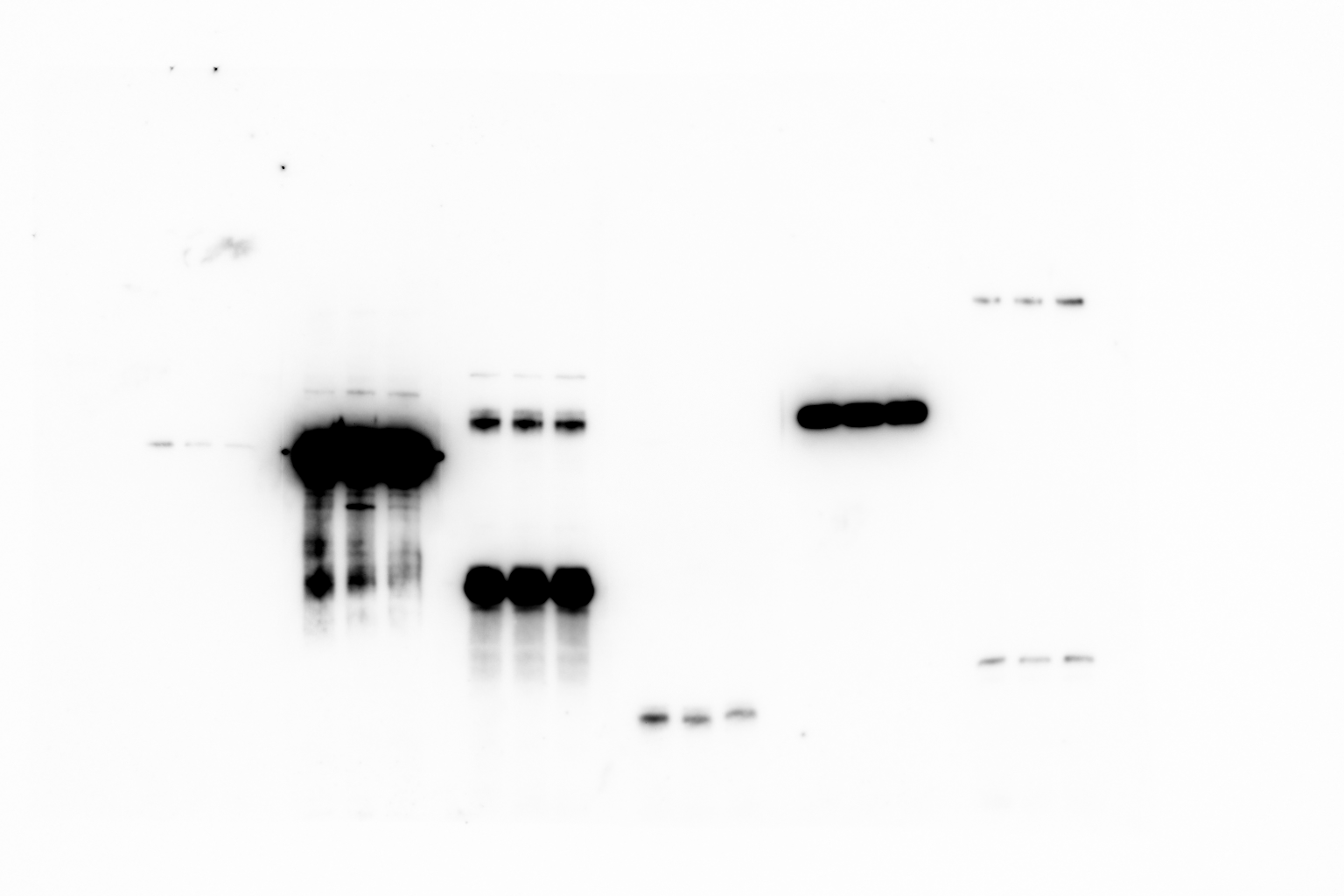

Supplement: Figure 1—source data 4. [file elife-108672-fig1-data4.zip › Fig 1B (part 1)/20210319_Blot4 10min_NDC1.tif]

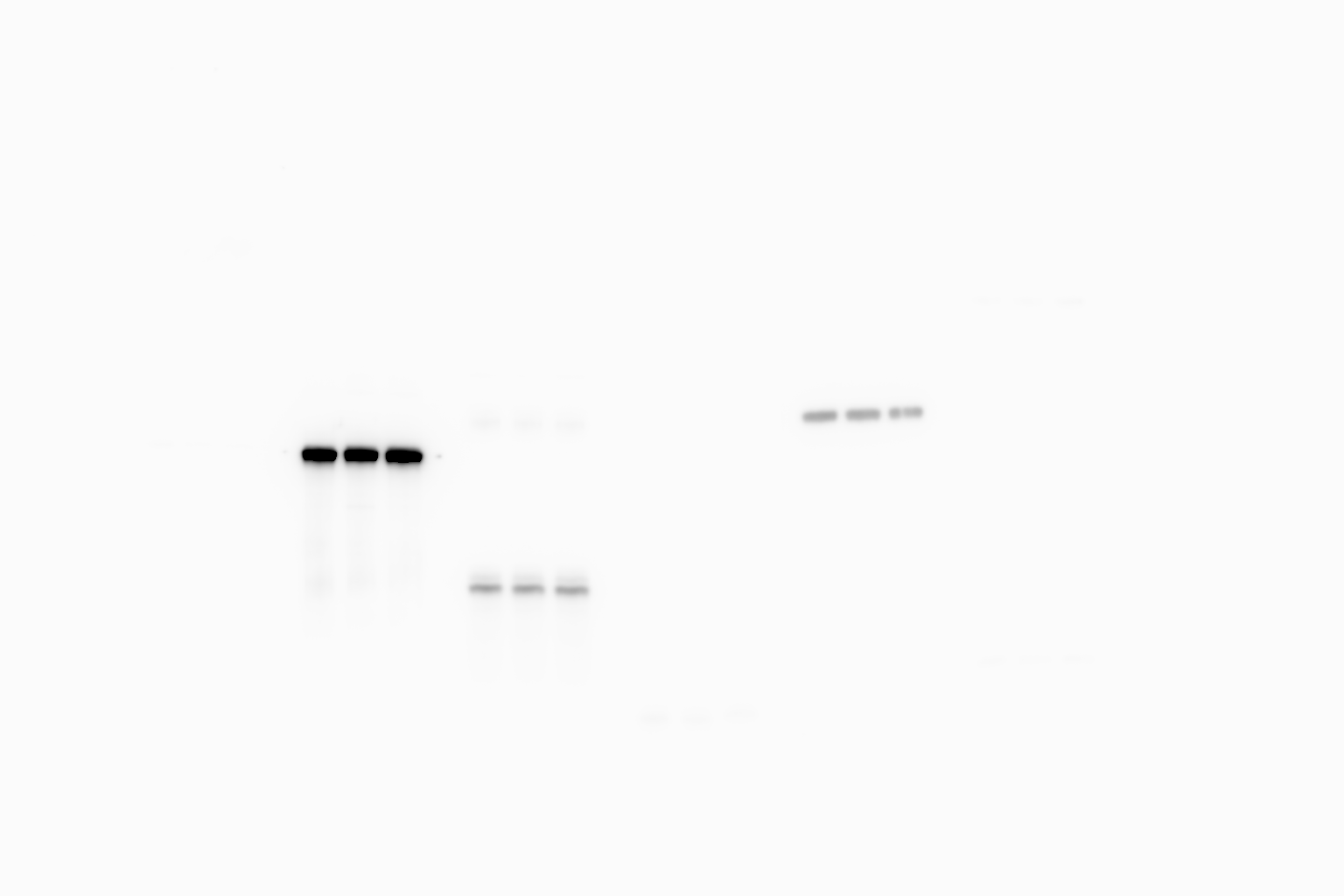

Supplement: Figure 1—source data 4. [file elife-108672-fig1-data4.zip › Fig 1B (part 1)/20210319_Blot4 1min.tif]

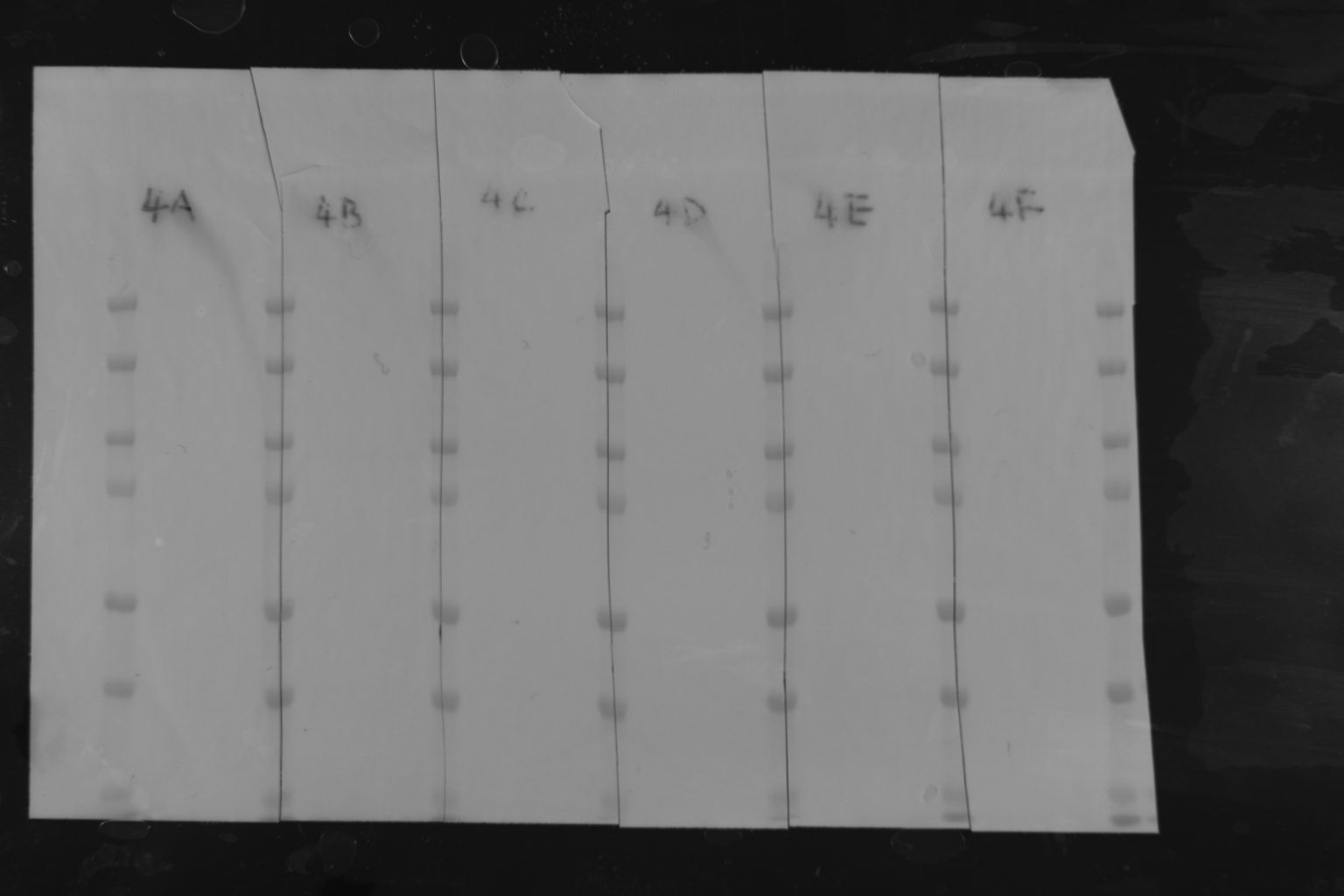

Supplement: Figure 1—source data 4. [file elife-108672-fig1-data4.zip › Fig 1B (part 1)/20210319_Blot4 ladder.tif]

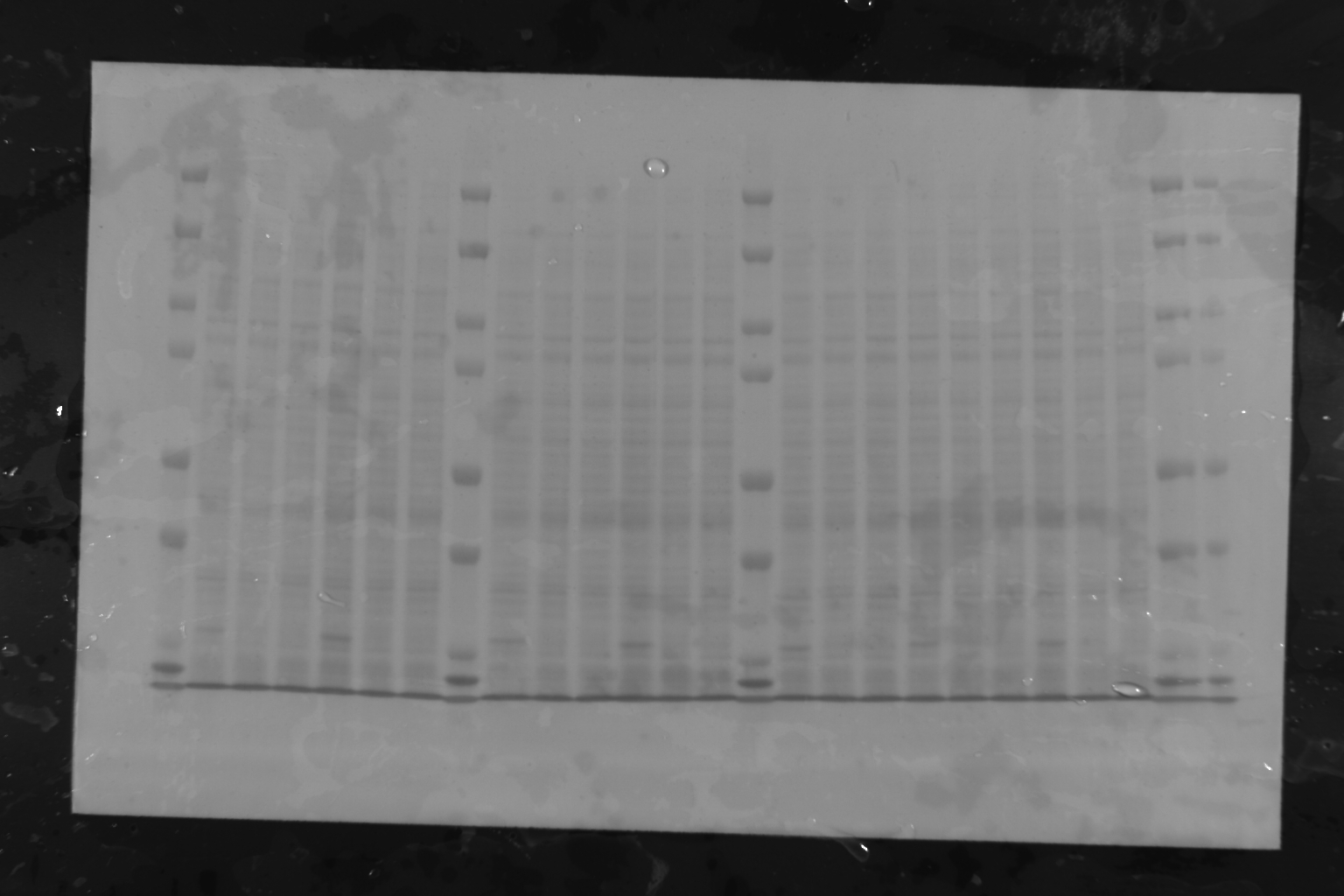

Supplement: Figure 1—source data 4. [file elife-108672-fig1-data4.zip › Fig 1B (part 1)/20210323_blot1 Ponceau.tif]

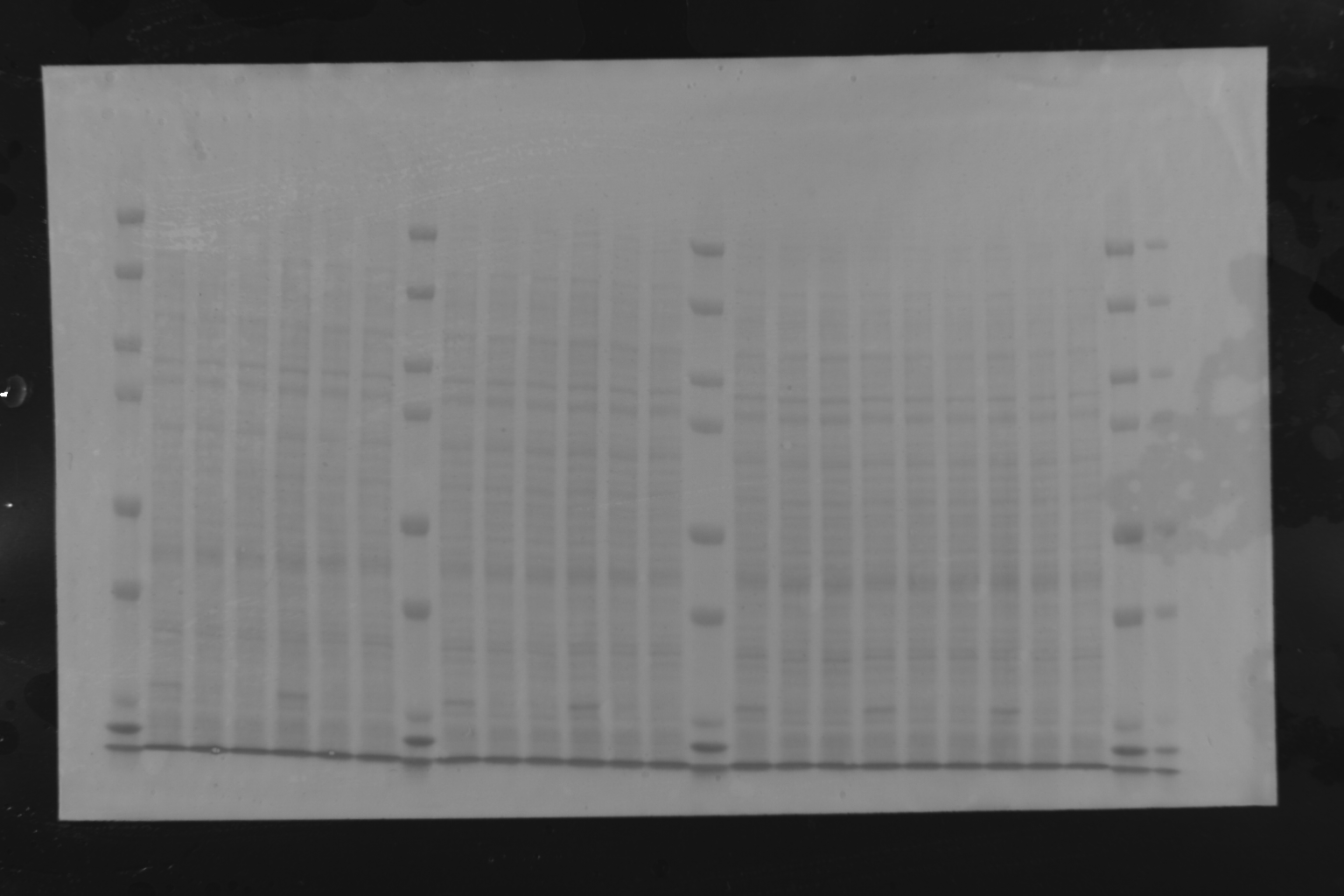

Supplement: Figure 1—source data 4. [file elife-108672-fig1-data4.zip › Fig 1B (part 1)/20210323_blot2 Ponceau.tif]

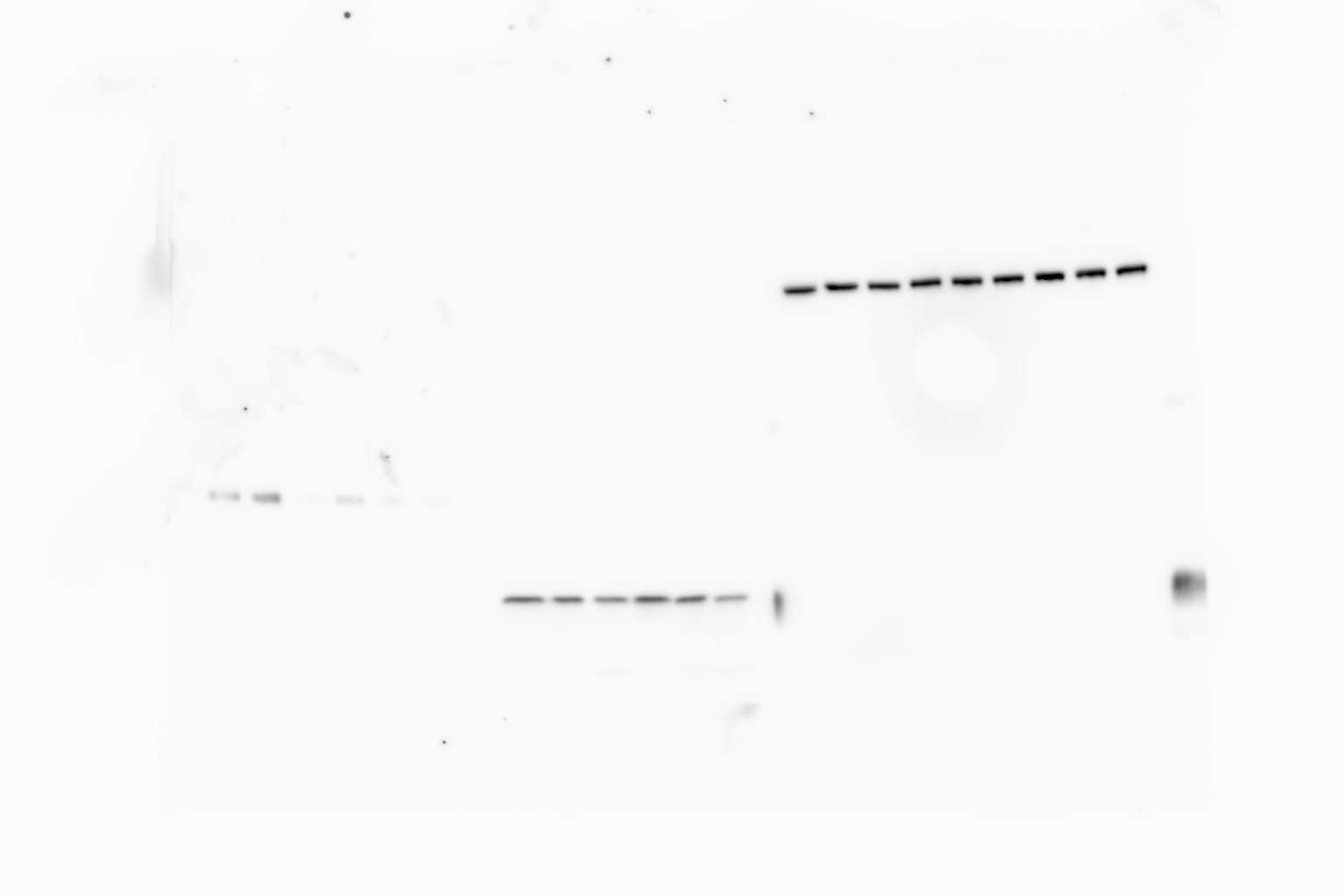

Supplement: Figure 1—source data 4. [file elife-108672-fig1-data4.zip › Fig 1B (part 1)/20210324_blot1 10min.tif]

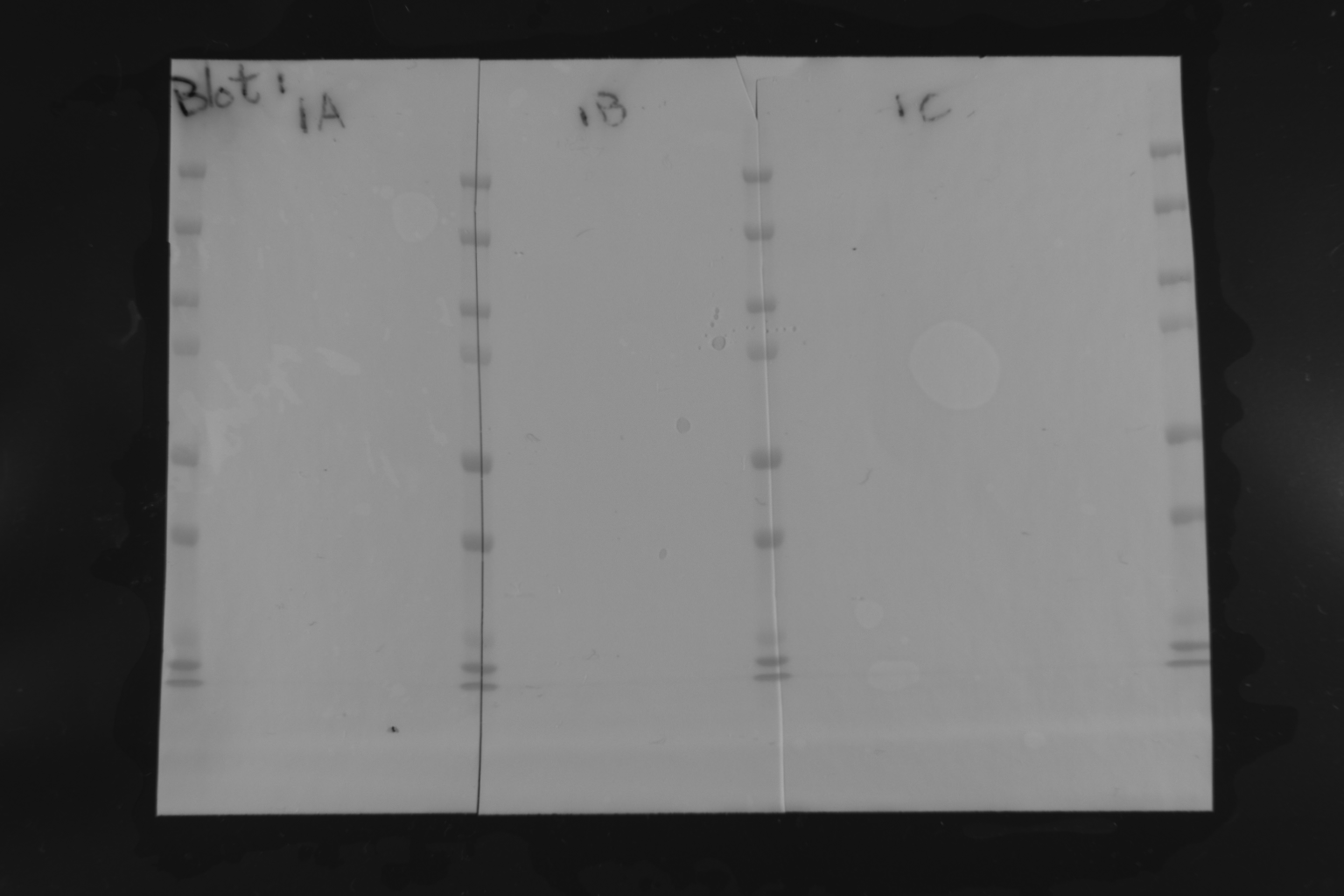

Supplement: Figure 1—source data 4. [file elife-108672-fig1-data4.zip › Fig 1B (part 1)/20210324_blot1 ladder.tif]

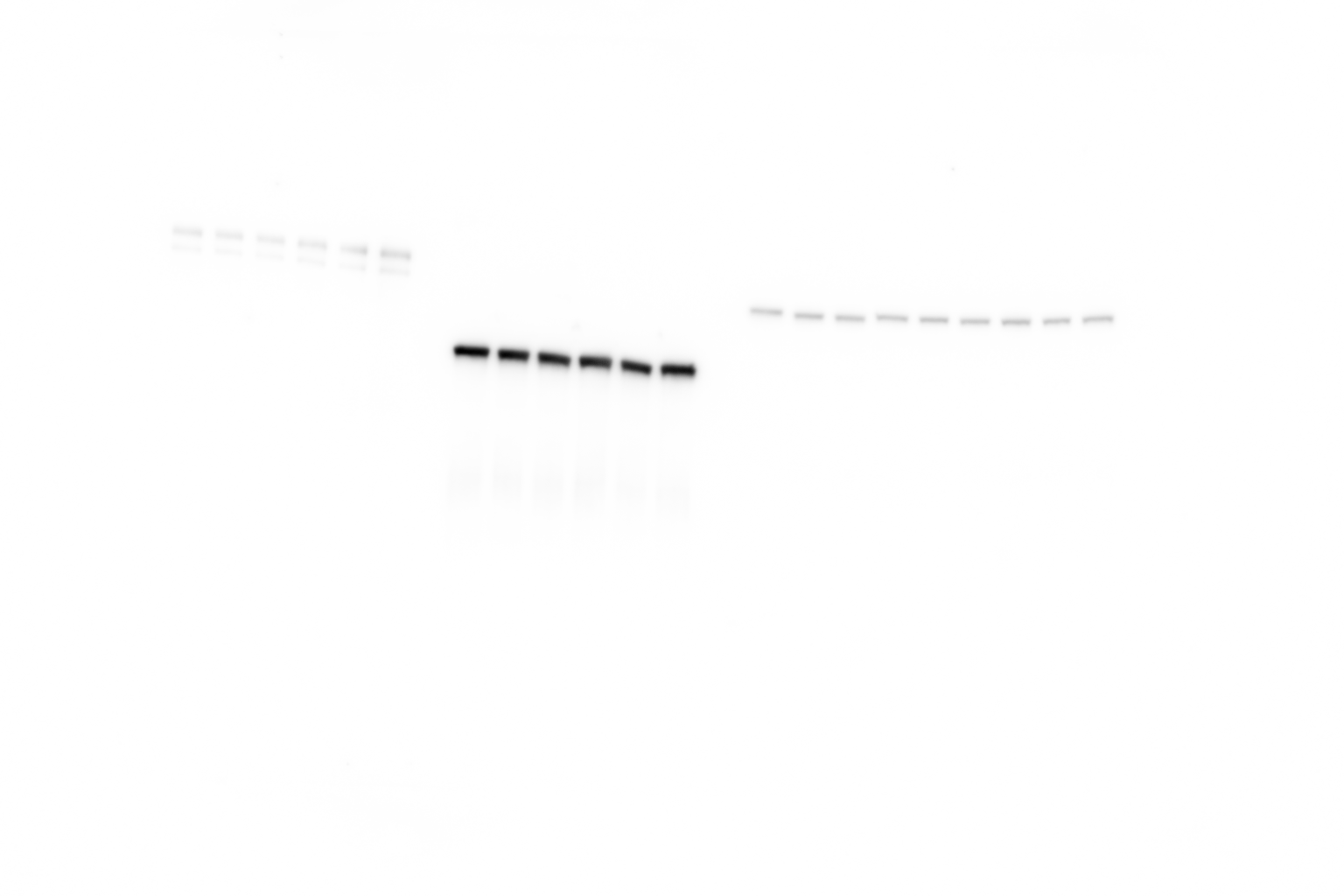

Supplement: Figure 1—source data 4. [file elife-108672-fig1-data4.zip › Fig 1B (part 1)/20210324_blot2 15sec.tif]

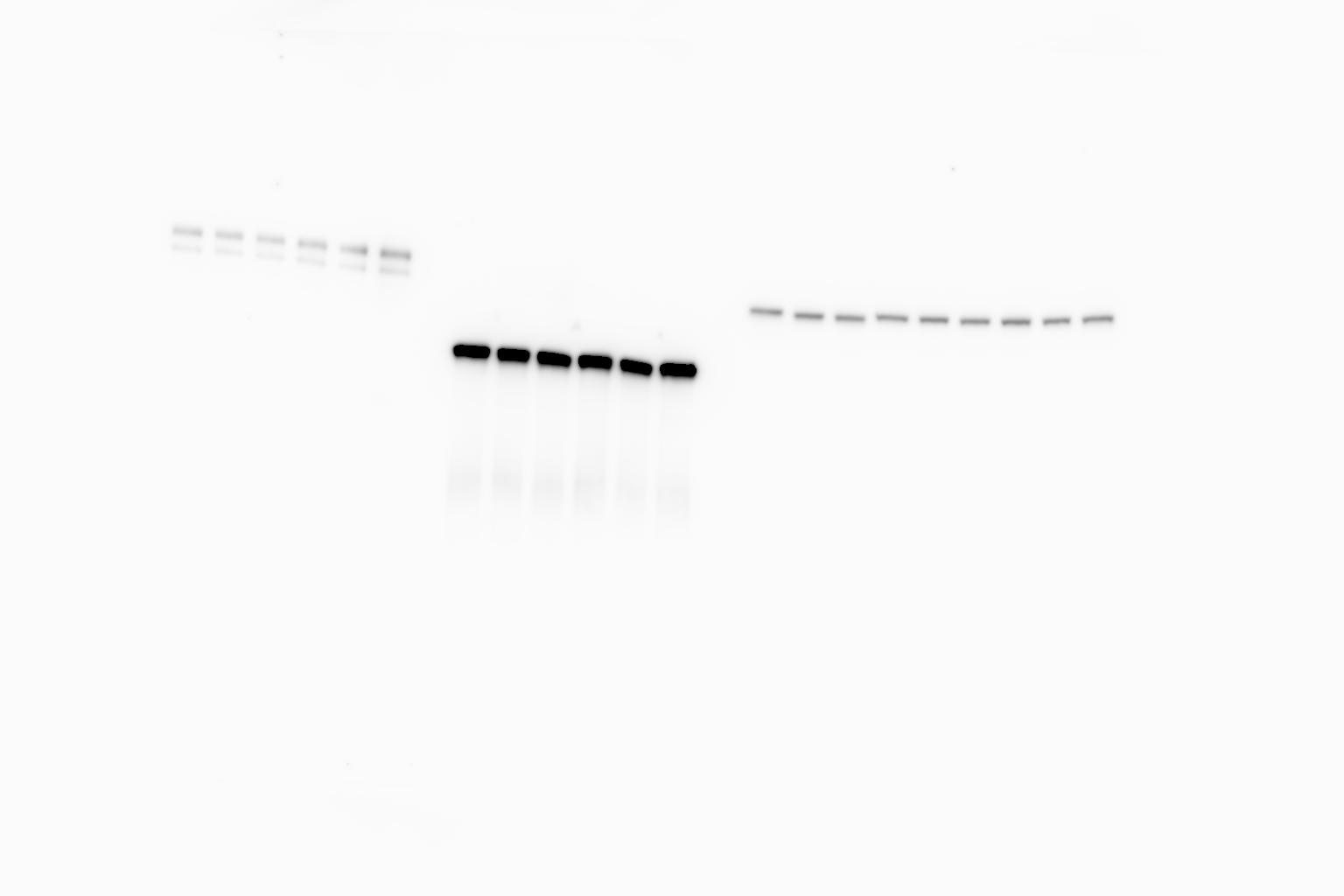

Supplement: Figure 1—source data 4. [file elife-108672-fig1-data4.zip › Fig 1B (part 1)/20210324_blot2 1min.tif]

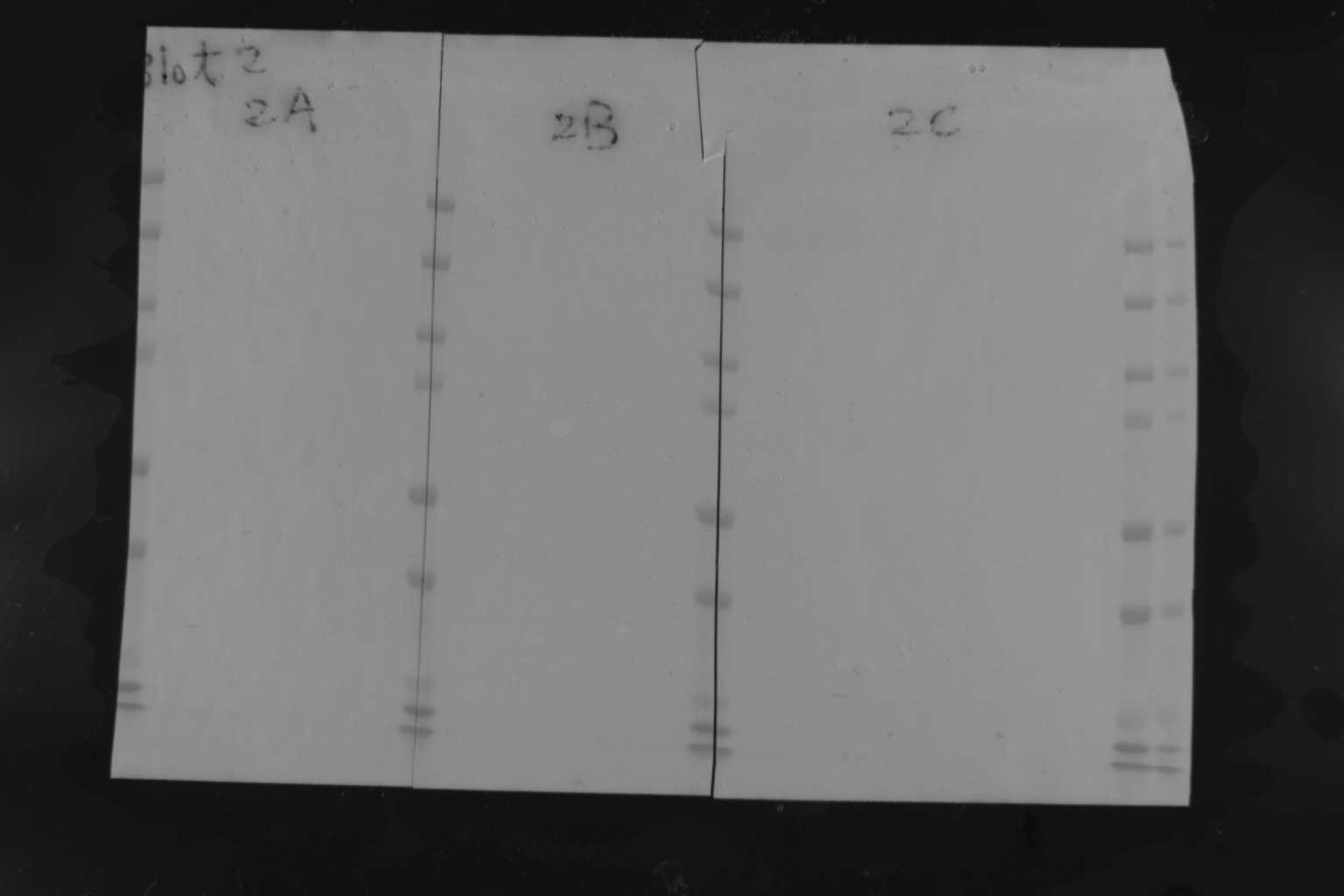

Supplement: Figure 1—source data 4. [file elife-108672-fig1-data4.zip › Fig 1B (part 1)/20210324_blot2 ladder.tif]

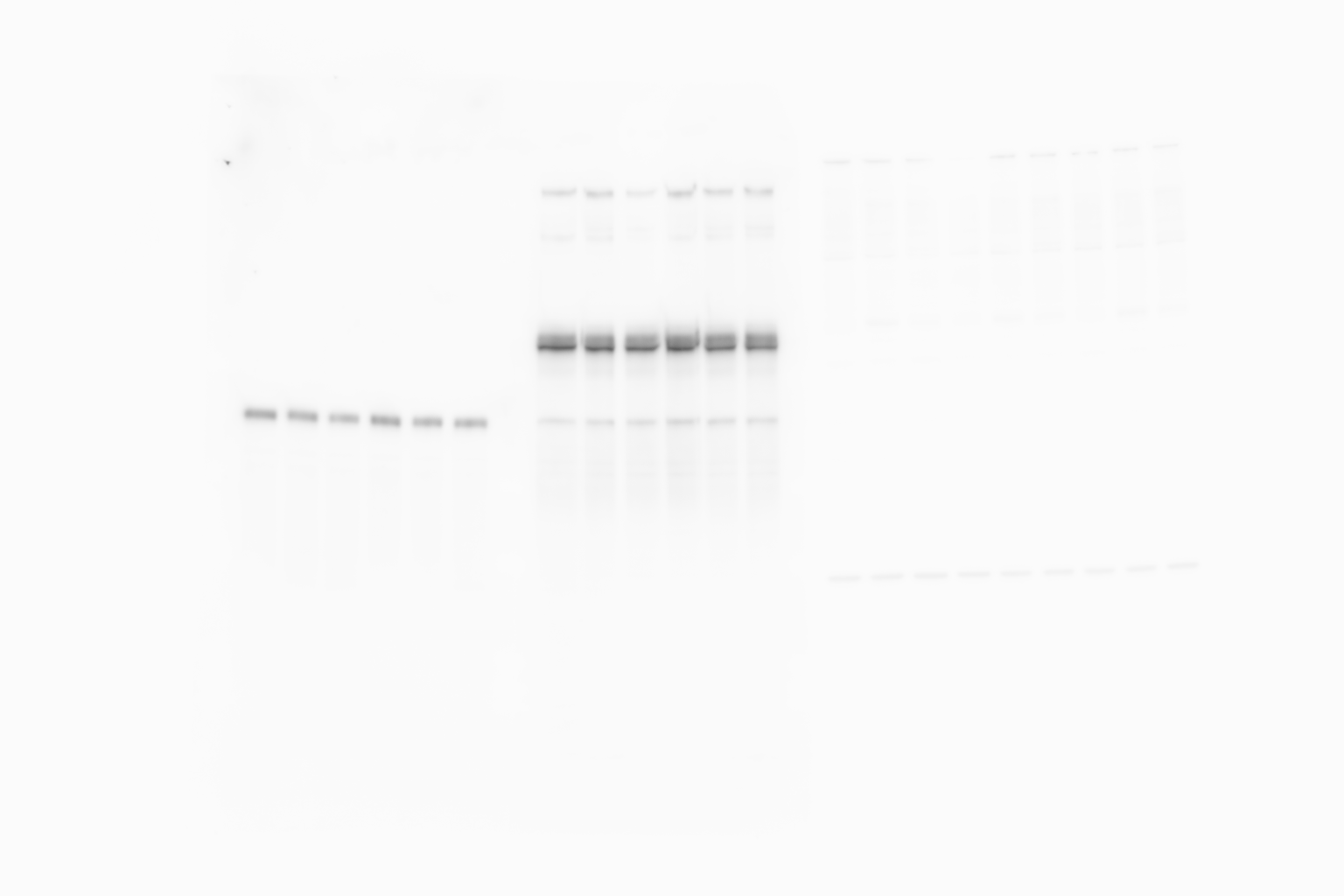

Supplement: Figure 1—source data 4. [file elife-108672-fig1-data4.zip › Fig 1B (part 1)/20210325_overblot1 15sec.tif]

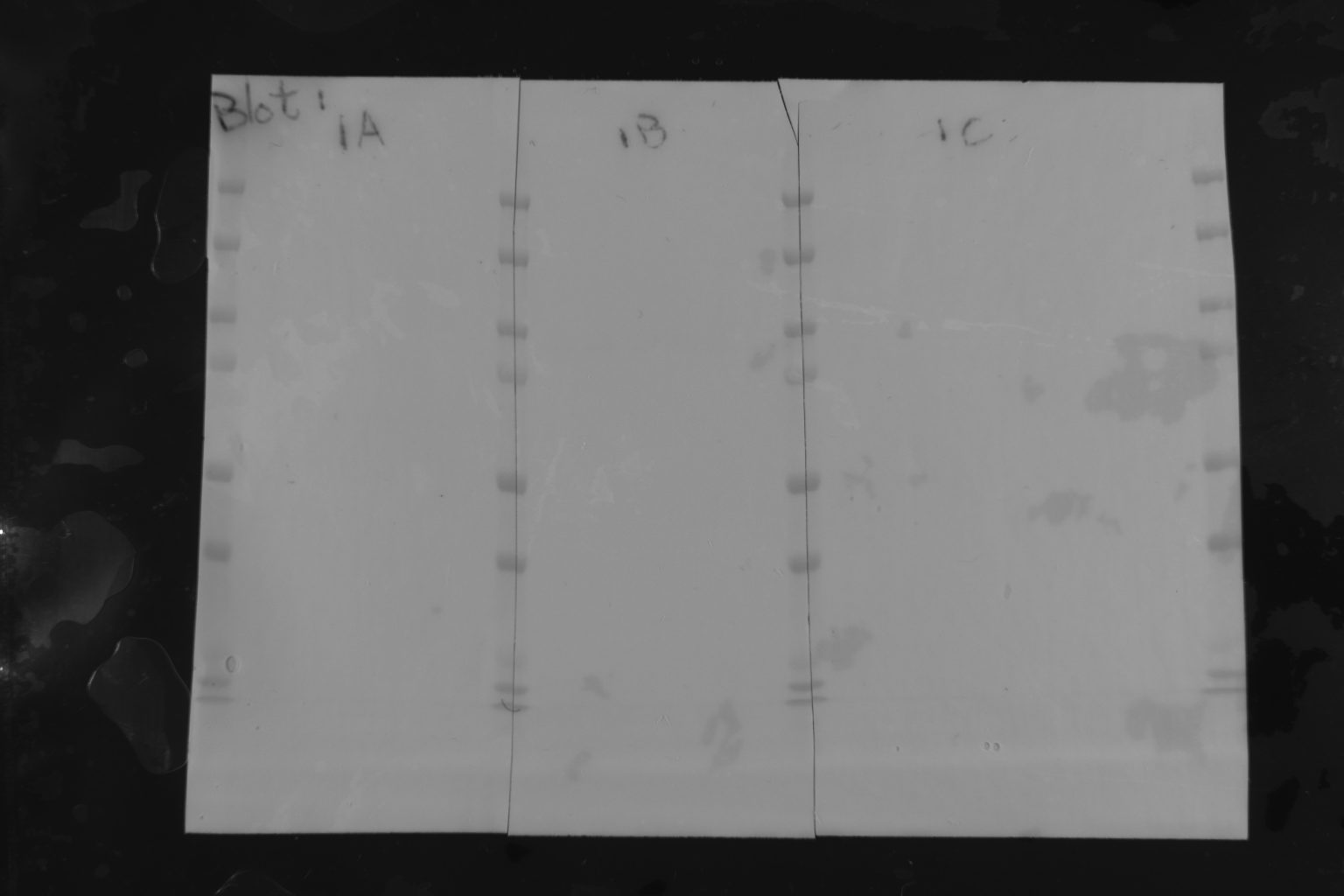

Supplement: Figure 1—source data 4. [file elife-108672-fig1-data4.zip › Fig 1B (part 1)/20210325_overblot1 ladder.tif]

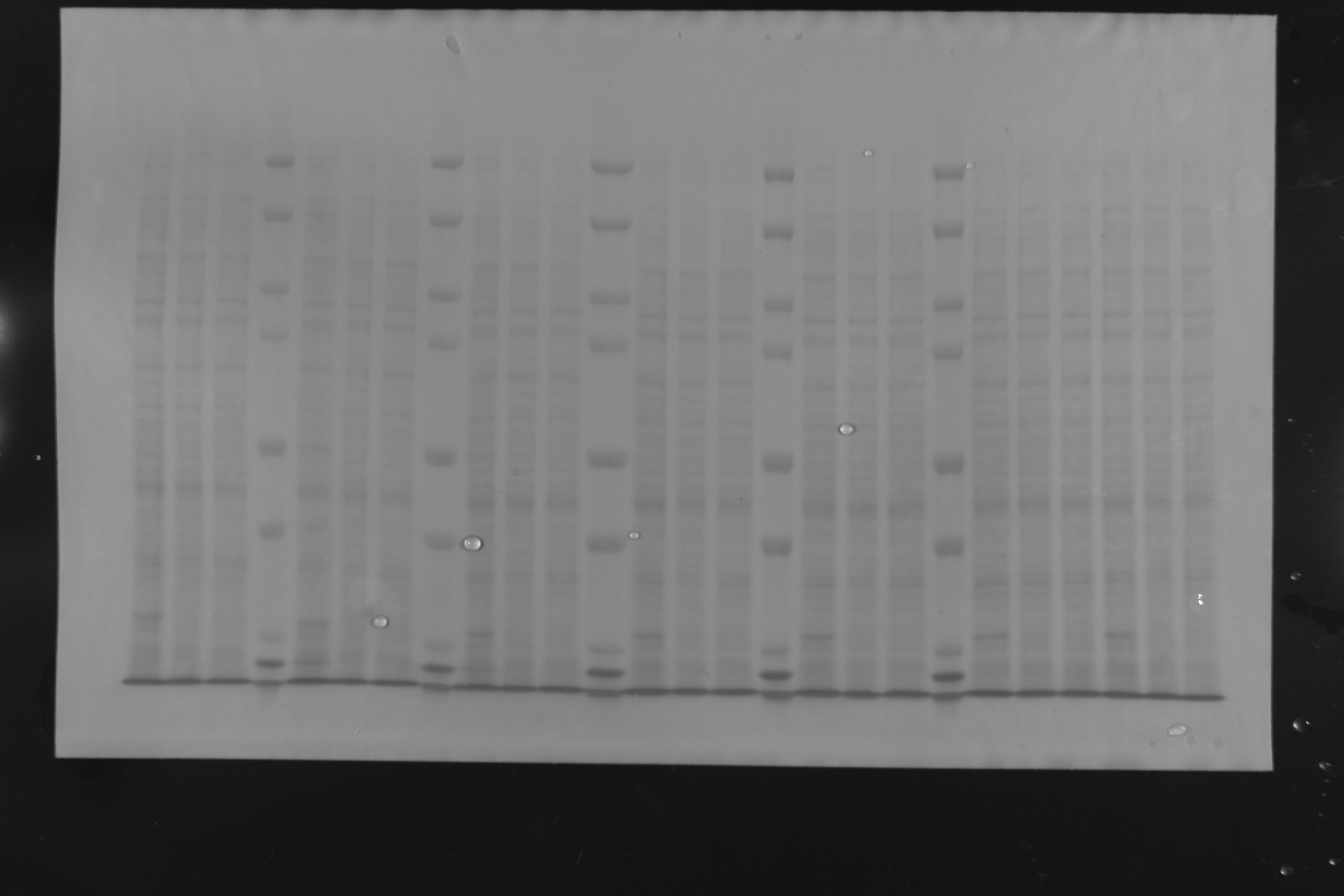

Supplement: Figure 1—source data 4. [file elife-108672-fig1-data4.zip › Fig 1B (part 1)/20210330_blot1 ponceau.tif]

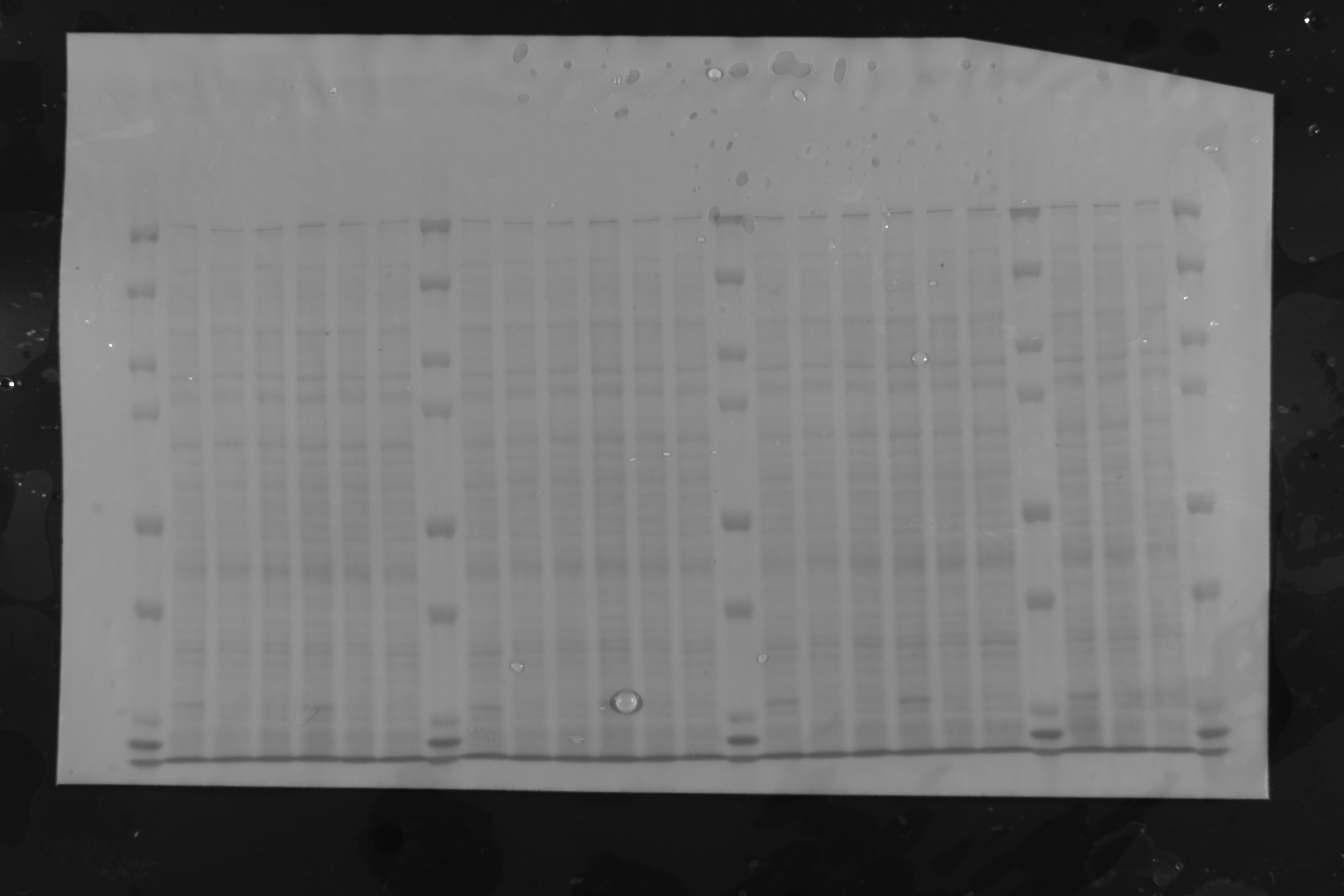

Supplement: Figure 1—source data 4. [file elife-108672-fig1-data4.zip › Fig 1B (part 1)/20210330_Blot2 Ponceau.tif]

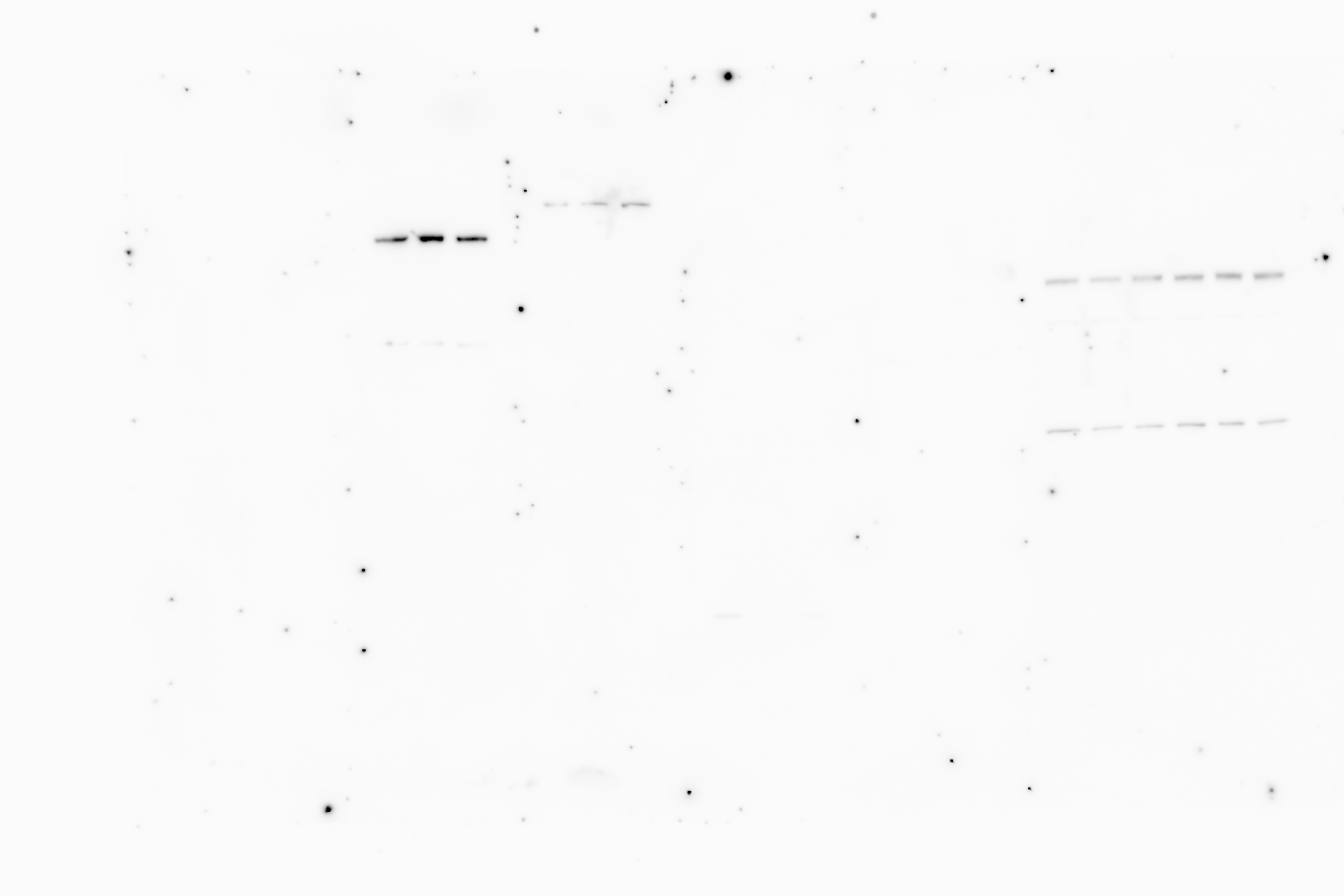

Supplement: Figure 1—source data 4. [file elife-108672-fig1-data4.zip › Fig 1B (part 1)/20210331_Blot1 10min.tif]

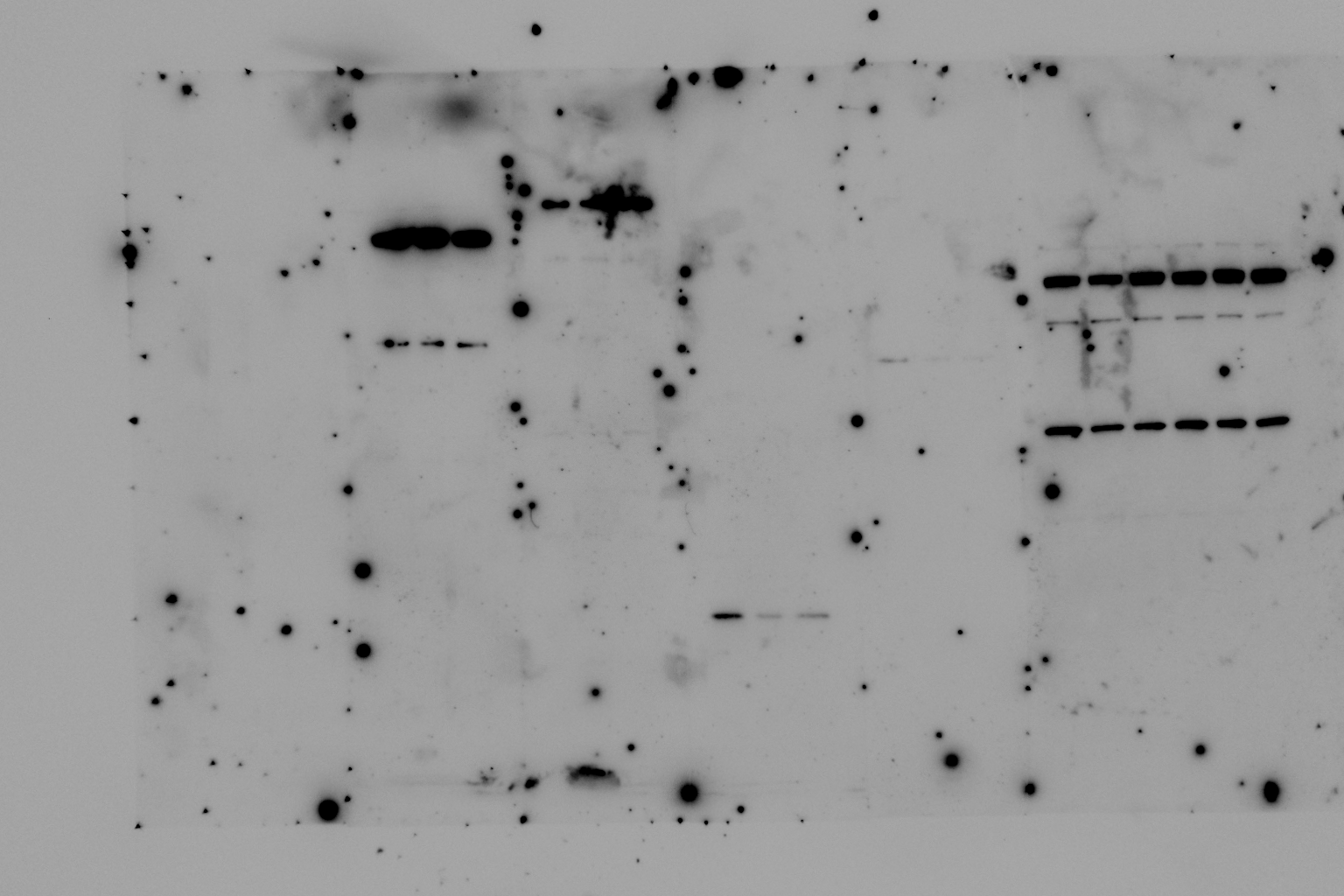

Supplement: Figure 1—source data 4. [file elife-108672-fig1-data4.zip › Fig 1B (part 1)/20210331_Blot1 10min_NDC1.tif]

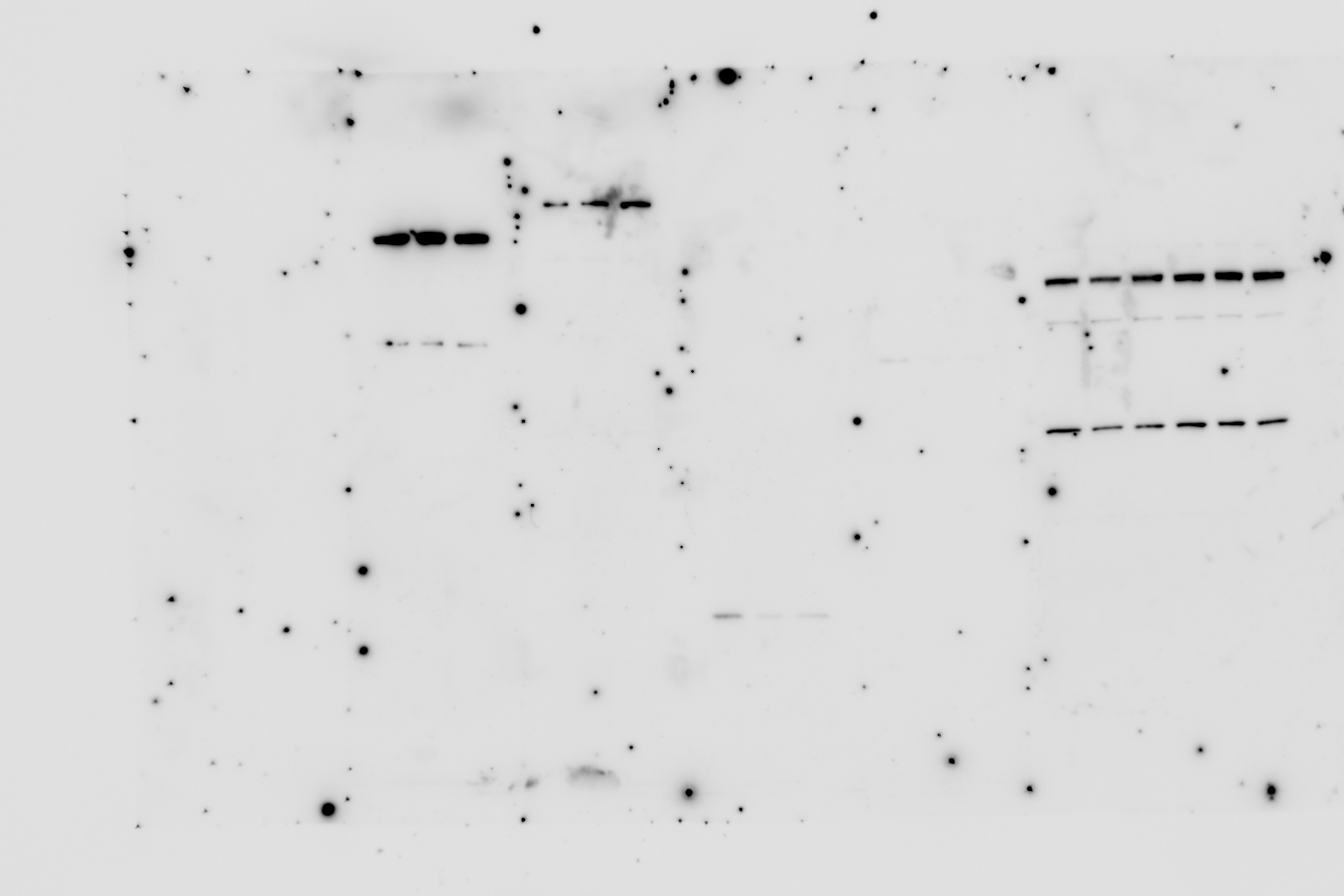

Supplement: Figure 1—source data 4. [file elife-108672-fig1-data4.zip › Fig 1B (part 1)/20210331_Blot1 10min_Nup35.tif]

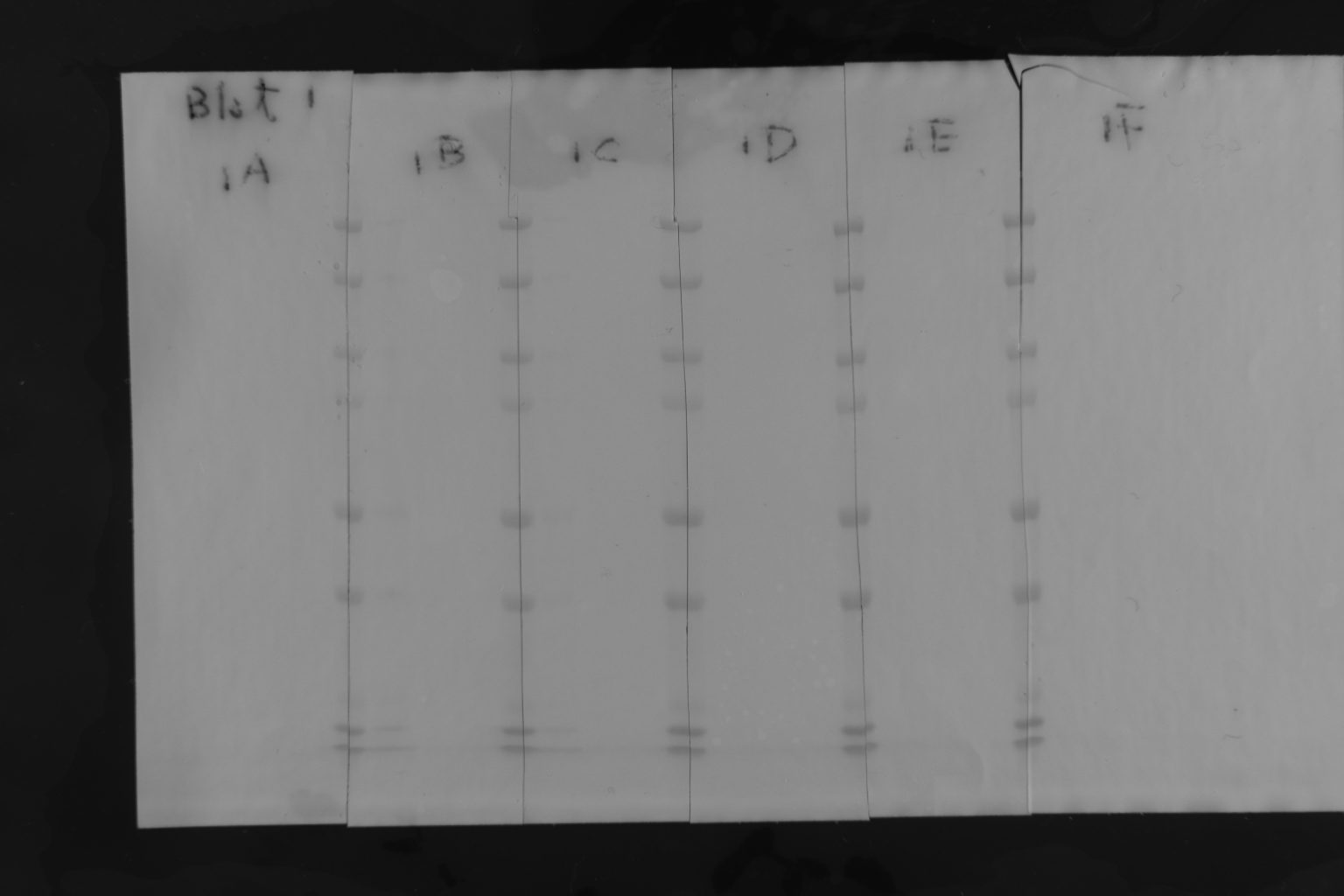

Supplement: Figure 1—source data 4. [file elife-108672-fig1-data4.zip › Fig 1B (part 1)/20210331_Blot1 ladder.tif]

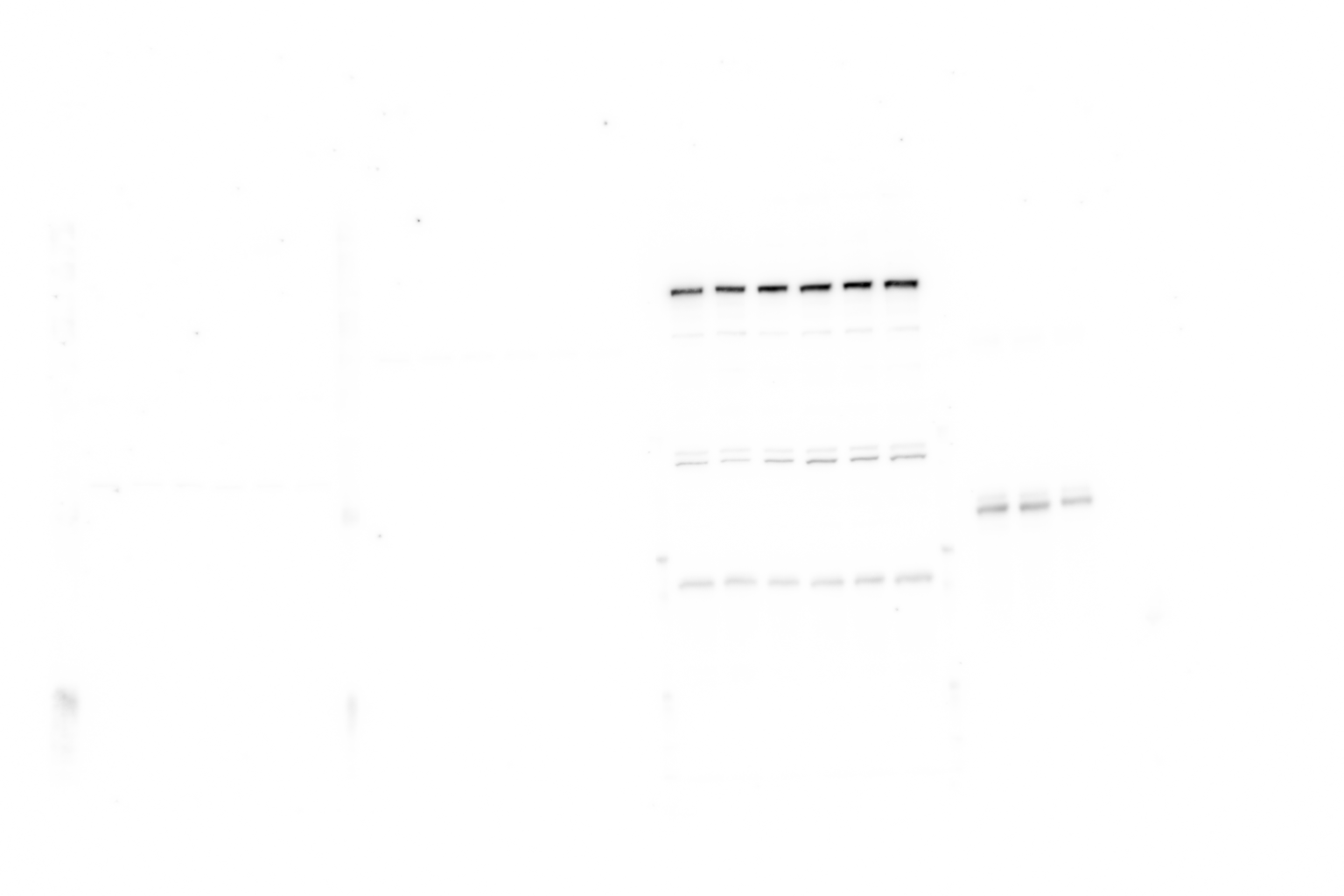

Supplement: Figure 1—source data 4. [file elife-108672-fig1-data4.zip › Fig 1B (part 1)/20210331_Blot2 1min.tif]

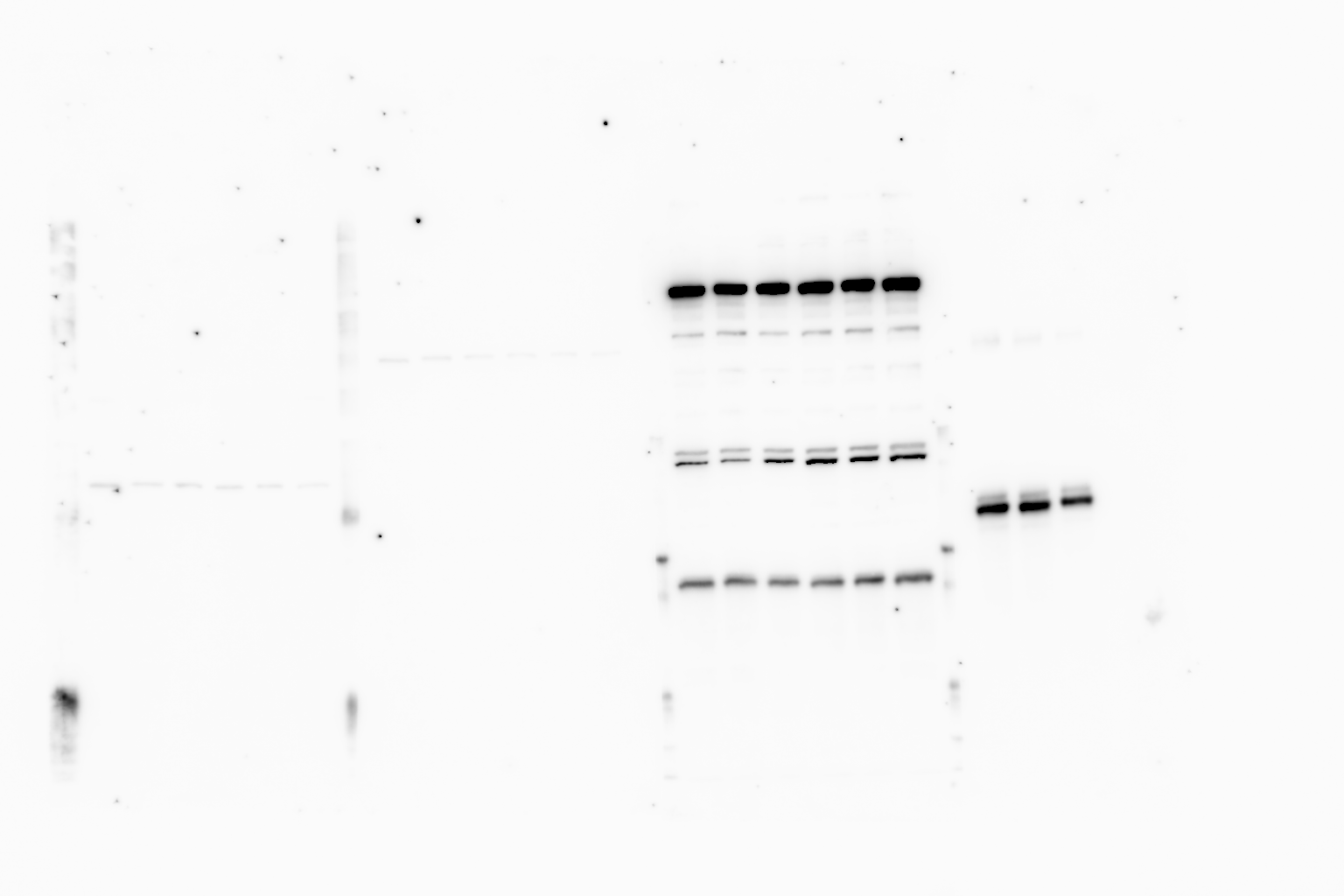

Supplement: Figure 1—source data 5. [file elife-108672-fig1-data5.zip › Fig 1B (part 2)/20210331_Blot2 10min.tif]

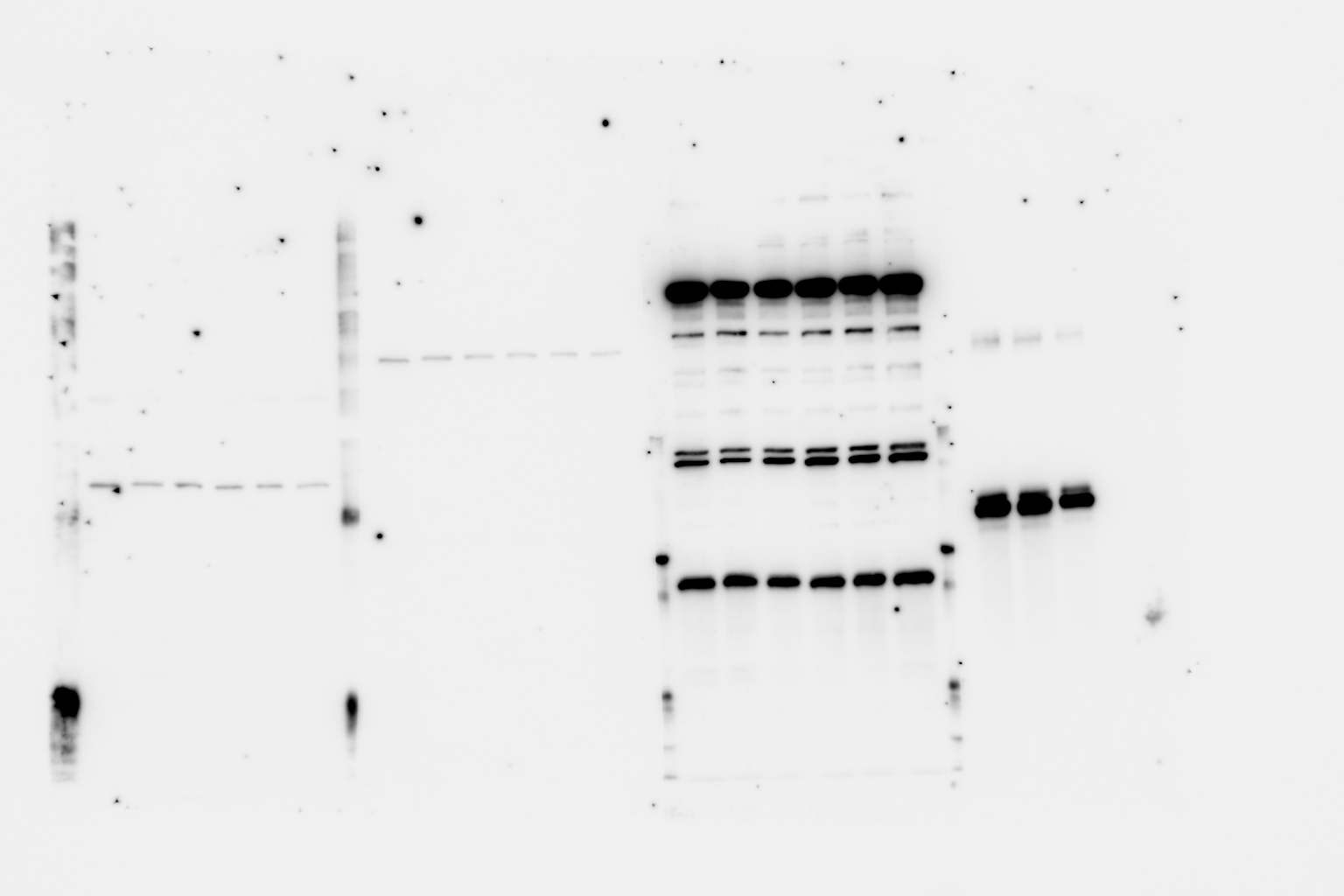

Supplement: Figure 1—source data 5. [file elife-108672-fig1-data5.zip › Fig 1B (part 2)/20210331_Blot2 10min_Nup85.tif]

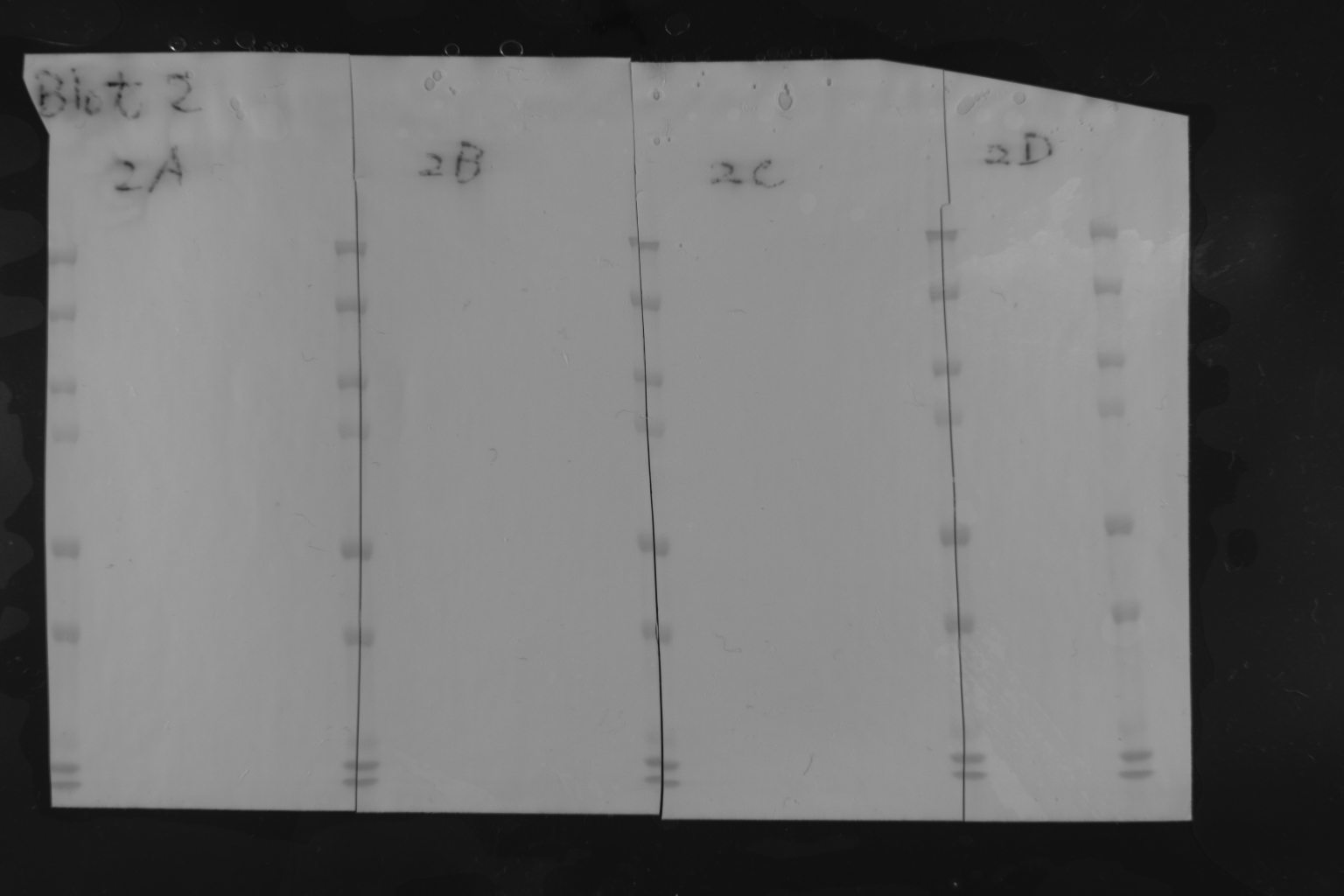

Supplement: Figure 1—source data 5. [file elife-108672-fig1-data5.zip › Fig 1B (part 2)/20210331_Blot2 ladder.tif]

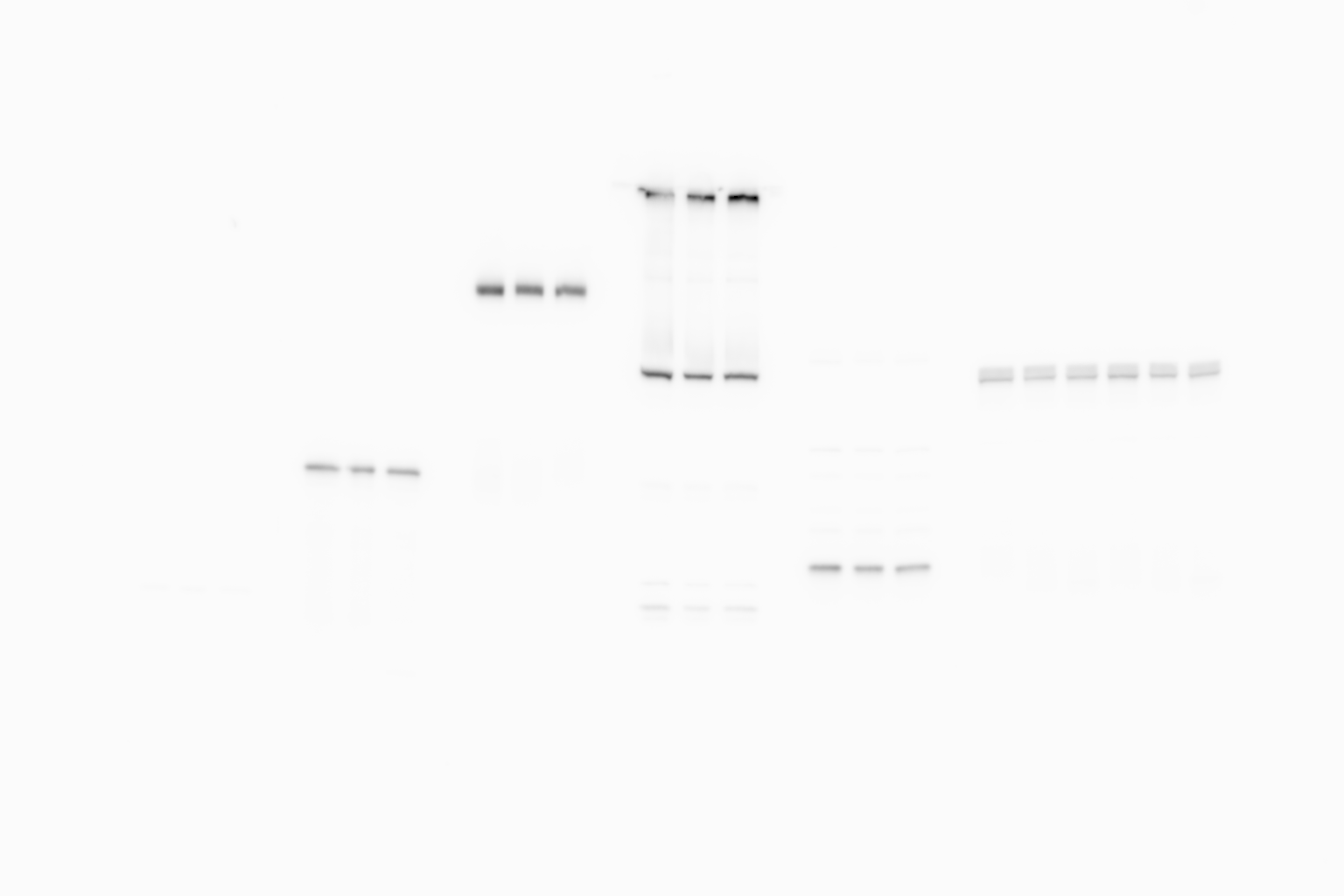

Supplement: Figure 1—source data 5. [file elife-108672-fig1-data5.zip › Fig 1B (part 2)/20210401_overblot1 1min.tif]

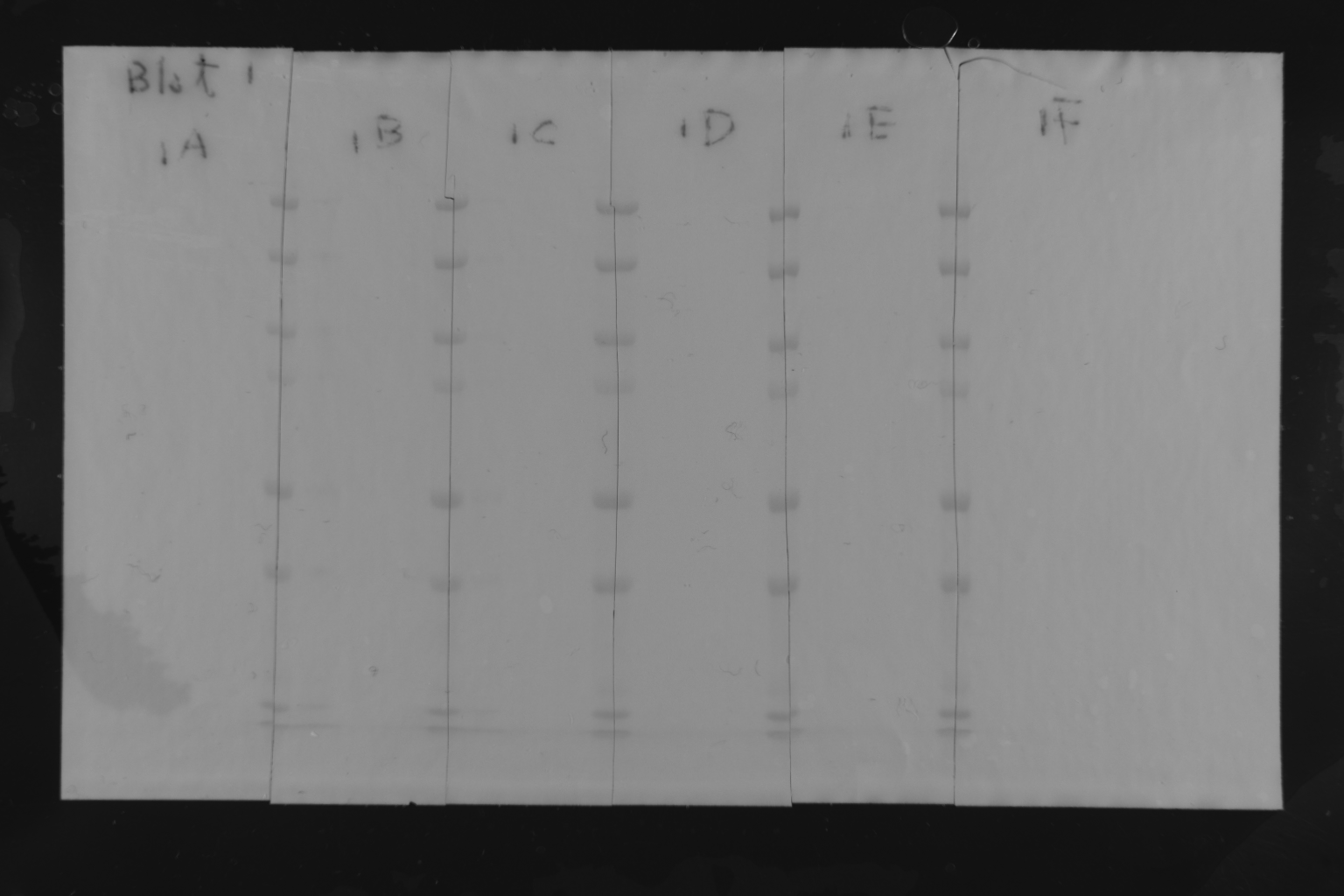

Supplement: Figure 1—source data 5. [file elife-108672-fig1-data5.zip › Fig 1B (part 2)/20210401_overblot1 ladder.tif]

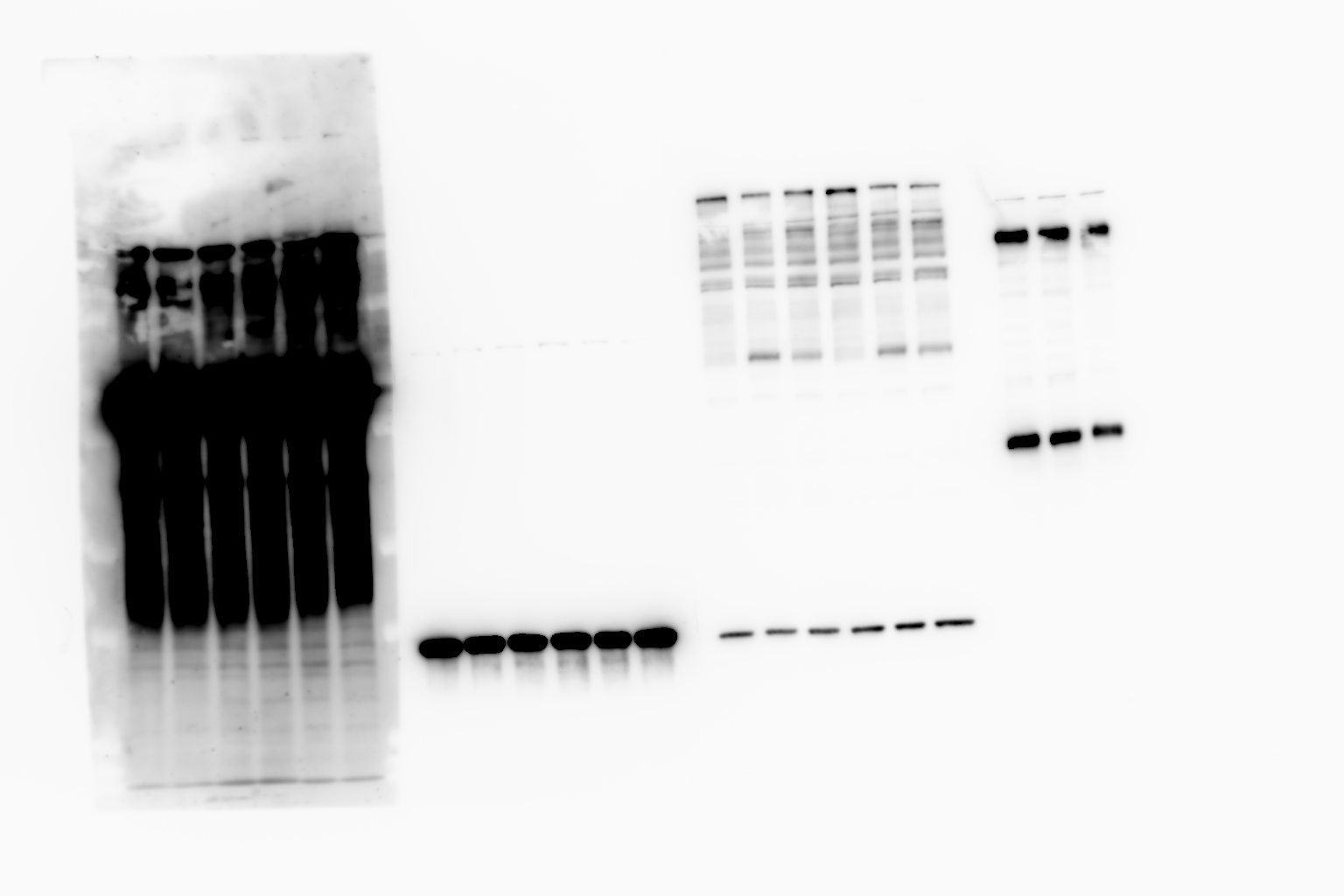

Supplement: Figure 1—source data 5. [file elife-108672-fig1-data5.zip › Fig 1B (part 2)/20210401_overblot2 10min.tif]

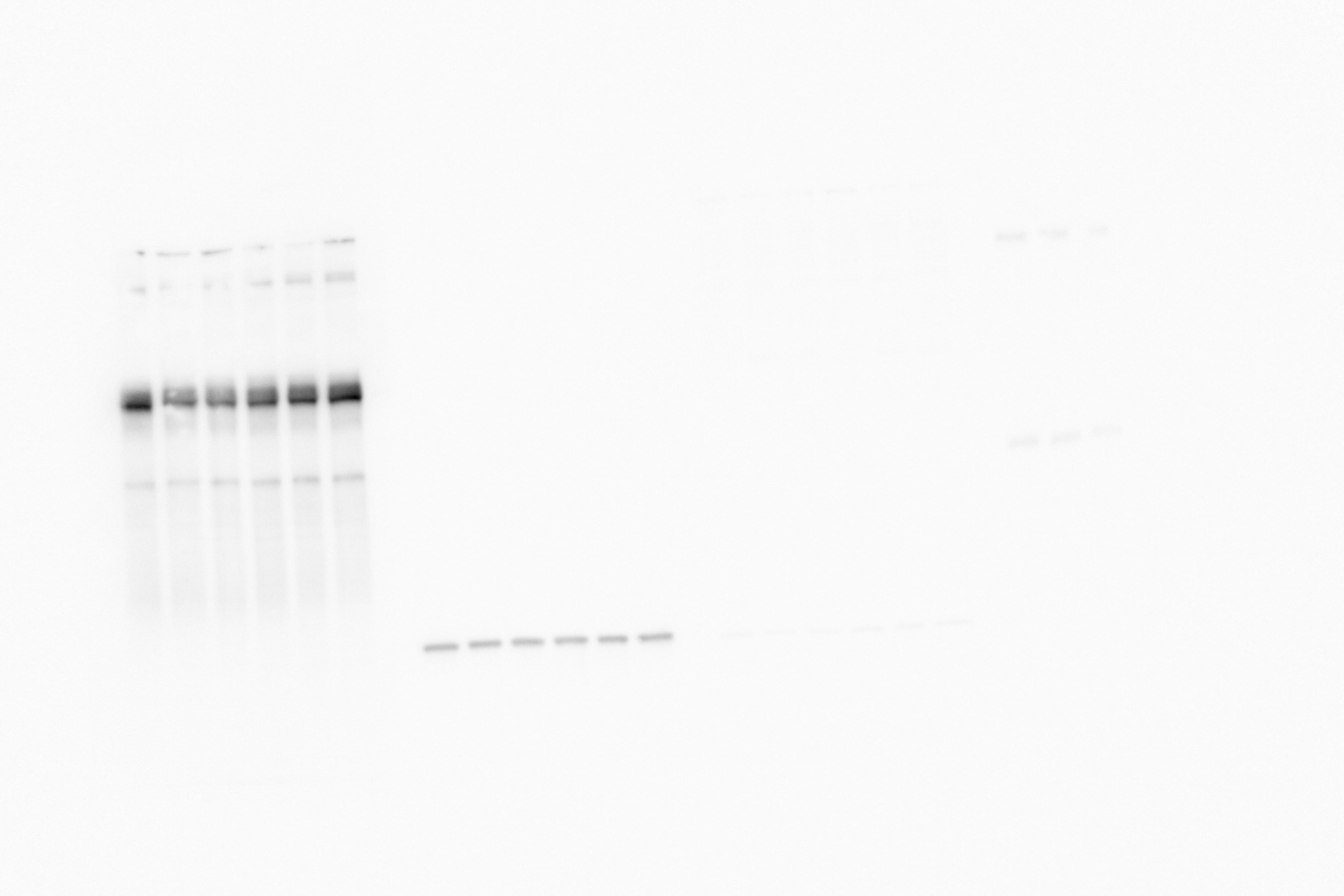

Supplement: Figure 1—source data 5. [file elife-108672-fig1-data5.zip › Fig 1B (part 2)/20210401_overblot2 1sec.tif]

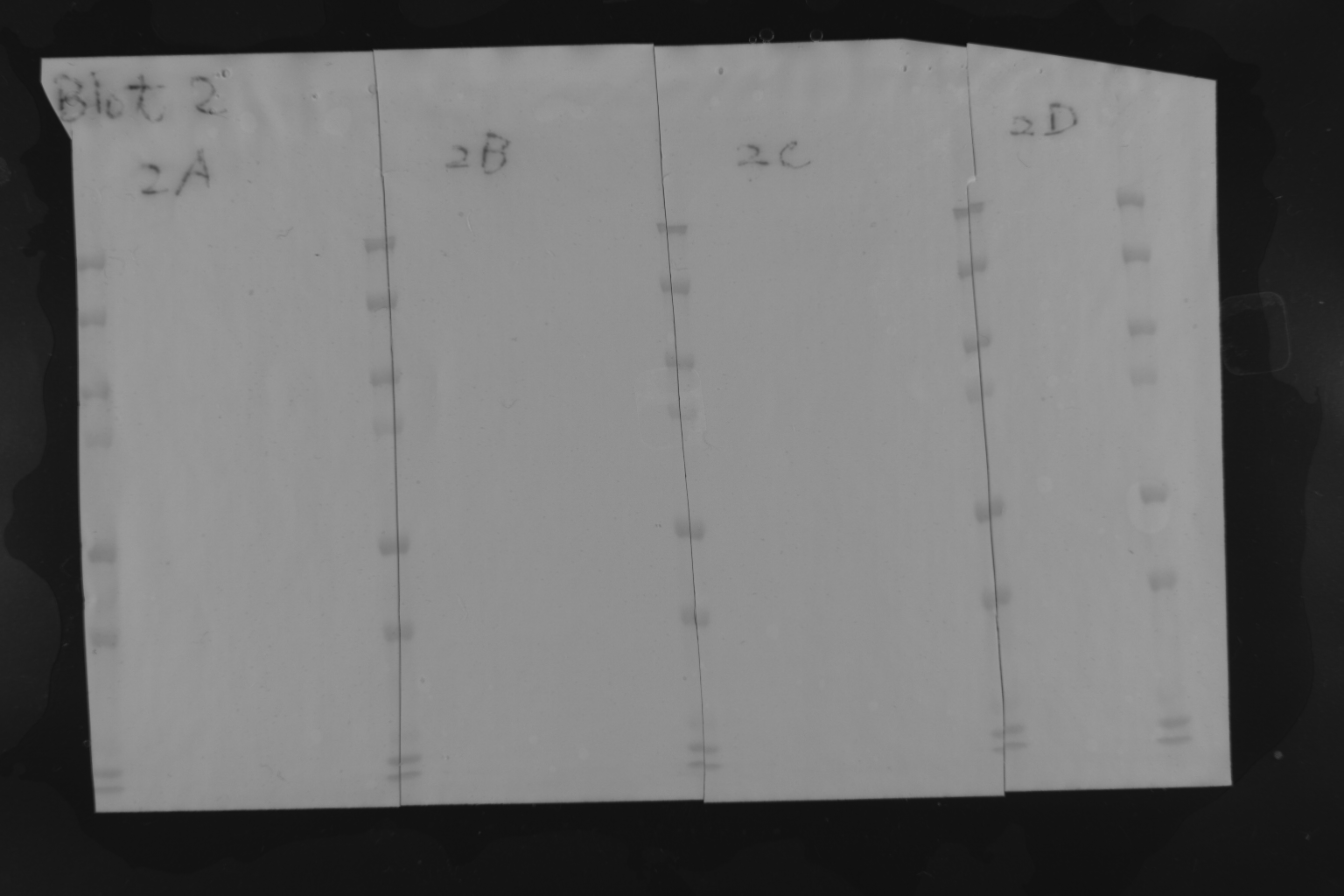

Supplement: Figure 1—source data 5. [file elife-108672-fig1-data5.zip › Fig 1B (part 2)/20210401_overblot2 ladder.tif]

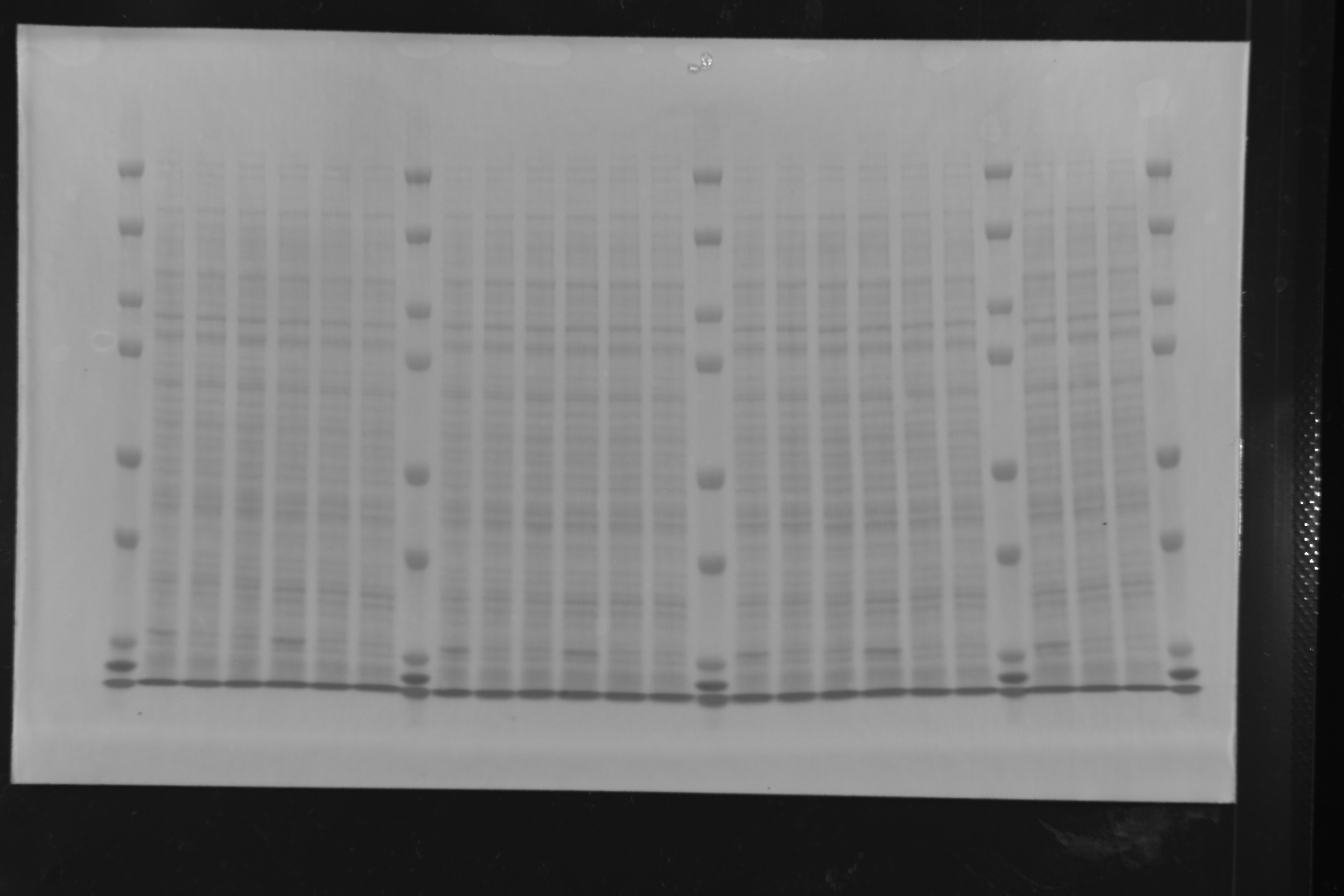

Supplement: Figure 1—source data 5. [file elife-108672-fig1-data5.zip › Fig 1B (part 2)/20210406_Blot3 Ponceau.tif]

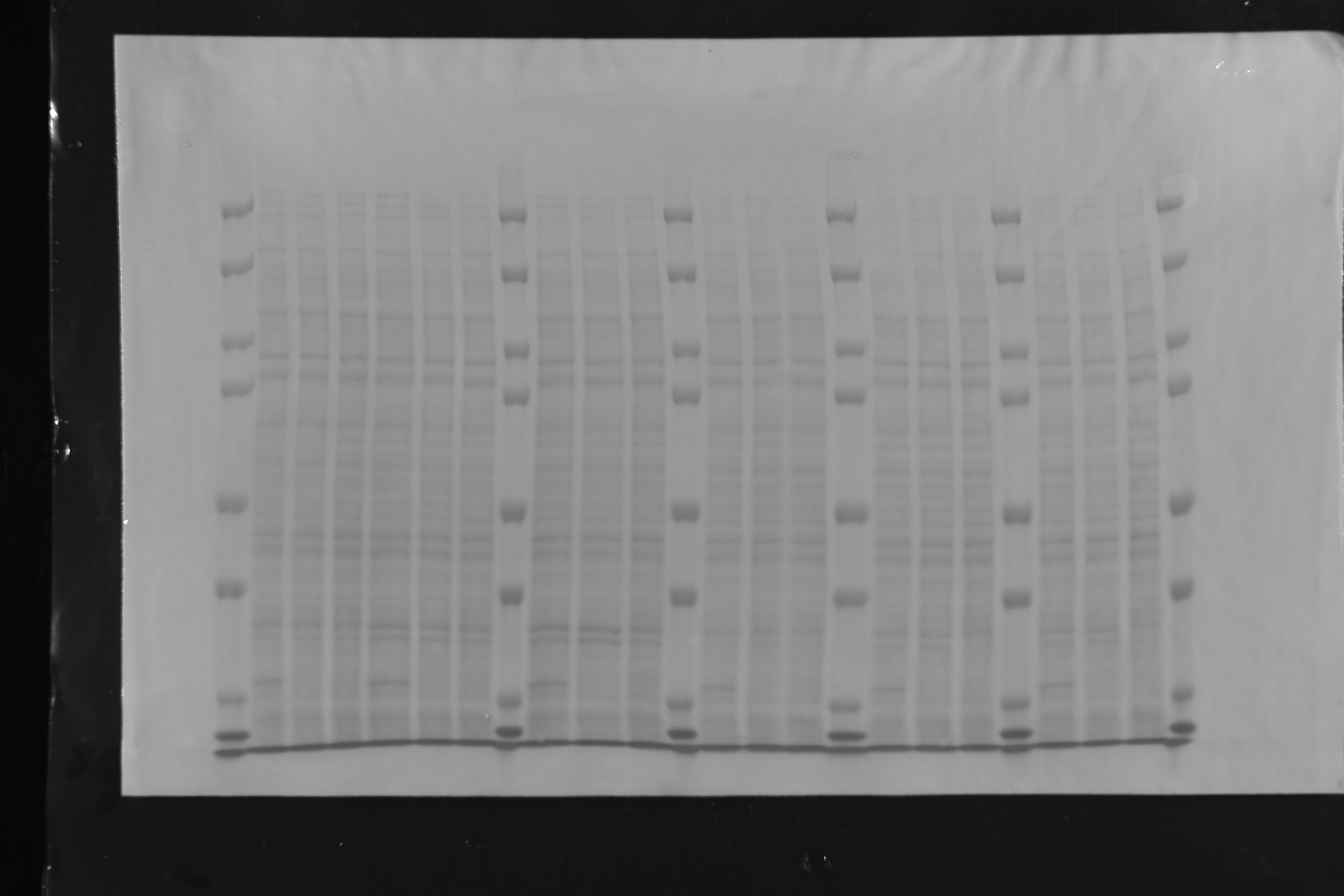

Supplement: Figure 1—source data 5. [file elife-108672-fig1-data5.zip › Fig 1B (part 2)/20210406_Blot4 Ponceau.tif]

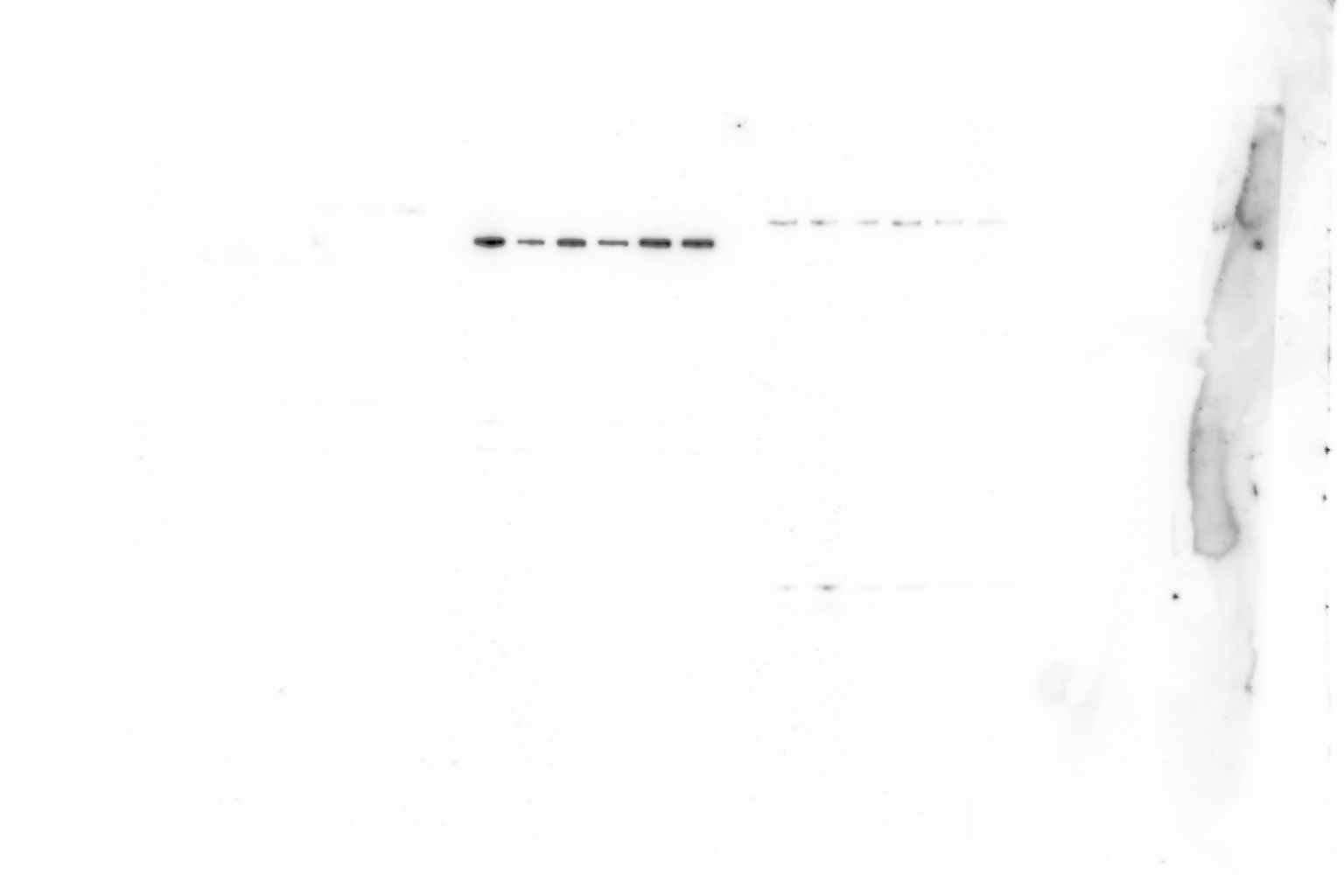

Supplement: Figure 1—source data 5. [file elife-108672-fig1-data5.zip › Fig 1B (part 2)/20210407_Blot3 10min.tif]

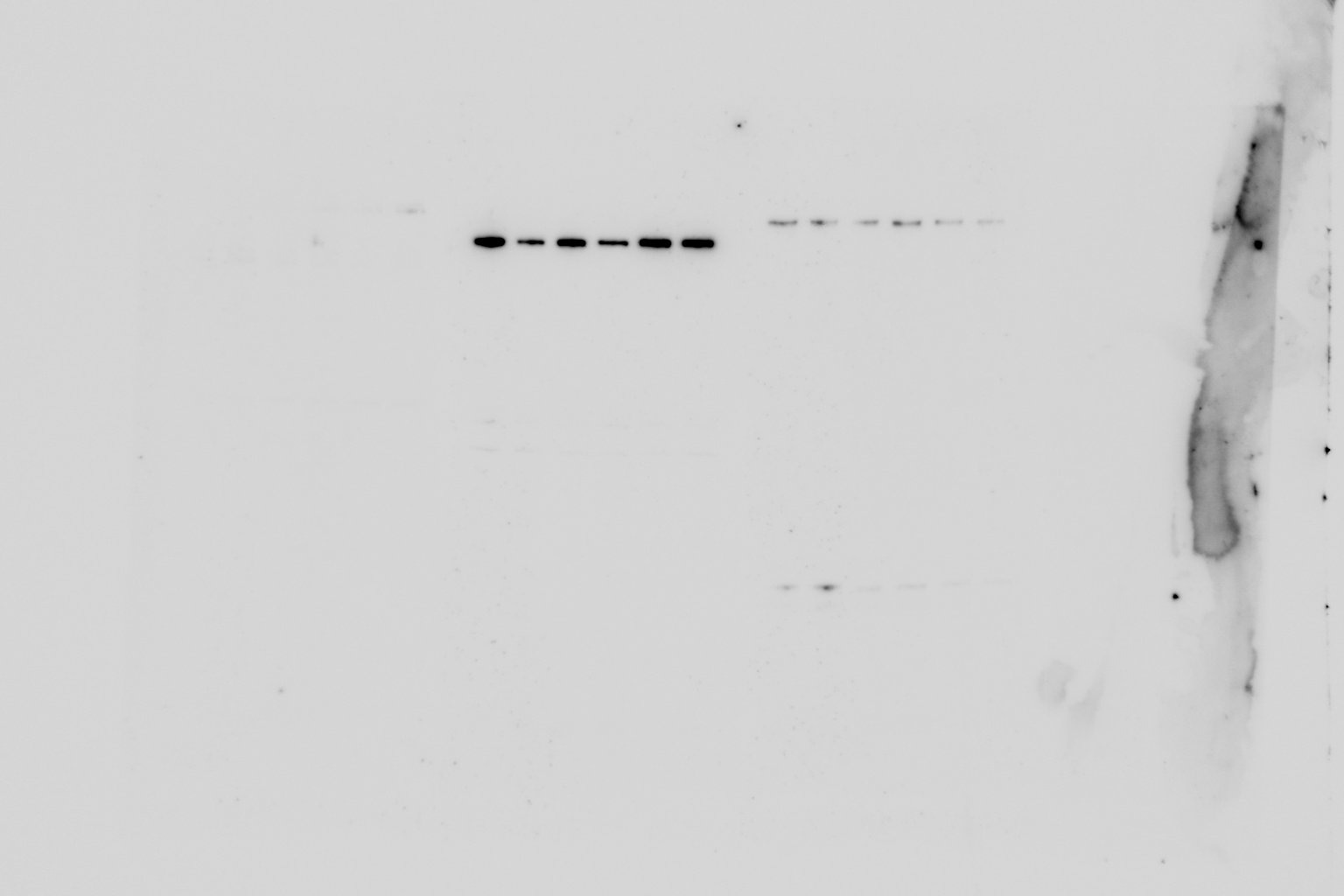

Supplement: Figure 1—source data 5. [file elife-108672-fig1-data5.zip › Fig 1B (part 2)/20210407_Blot3 10min_Nup214.tif]

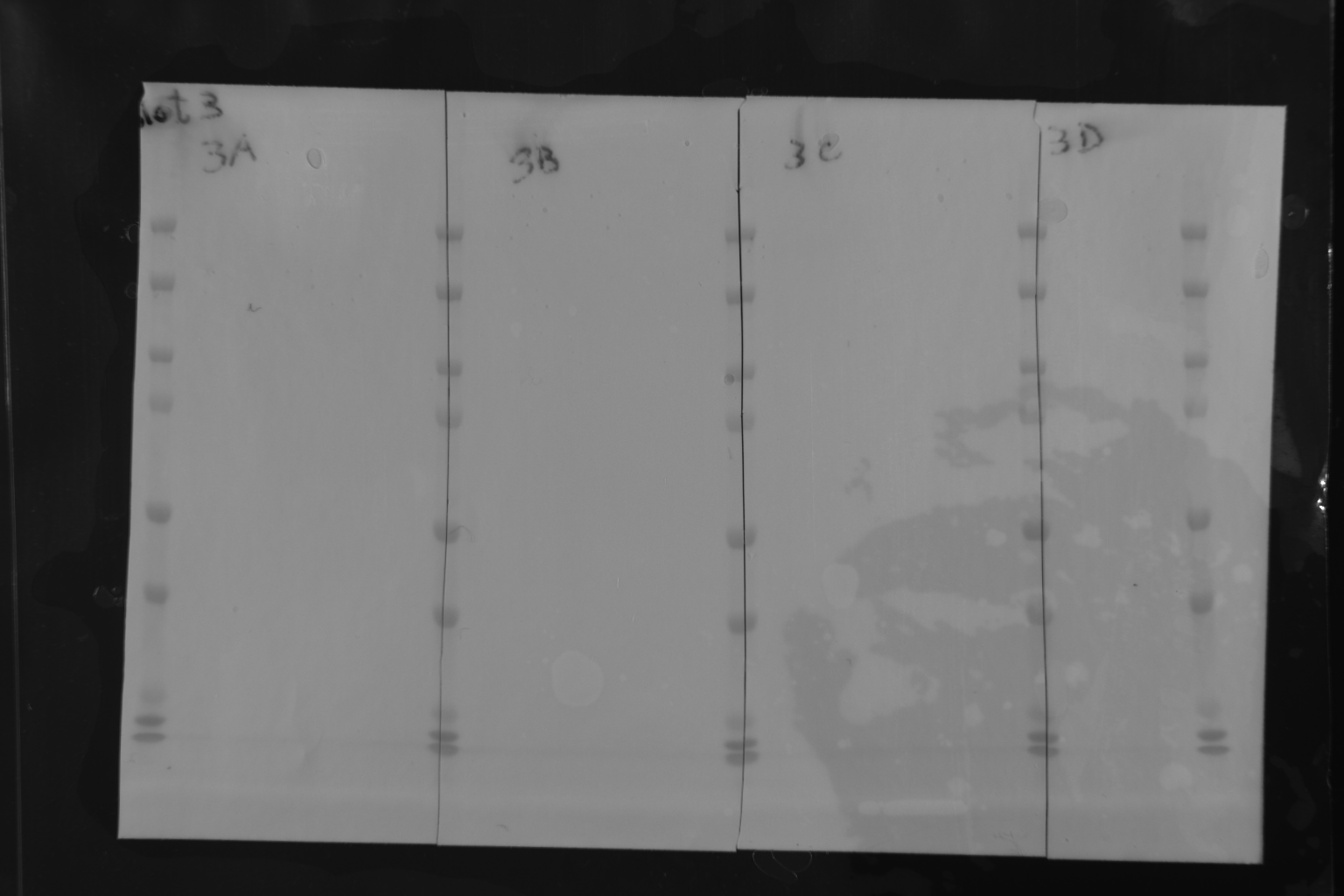

Supplement: Figure 1—source data 5. [file elife-108672-fig1-data5.zip › Fig 1B (part 2)/20210407_Blot3 ladder.tif]

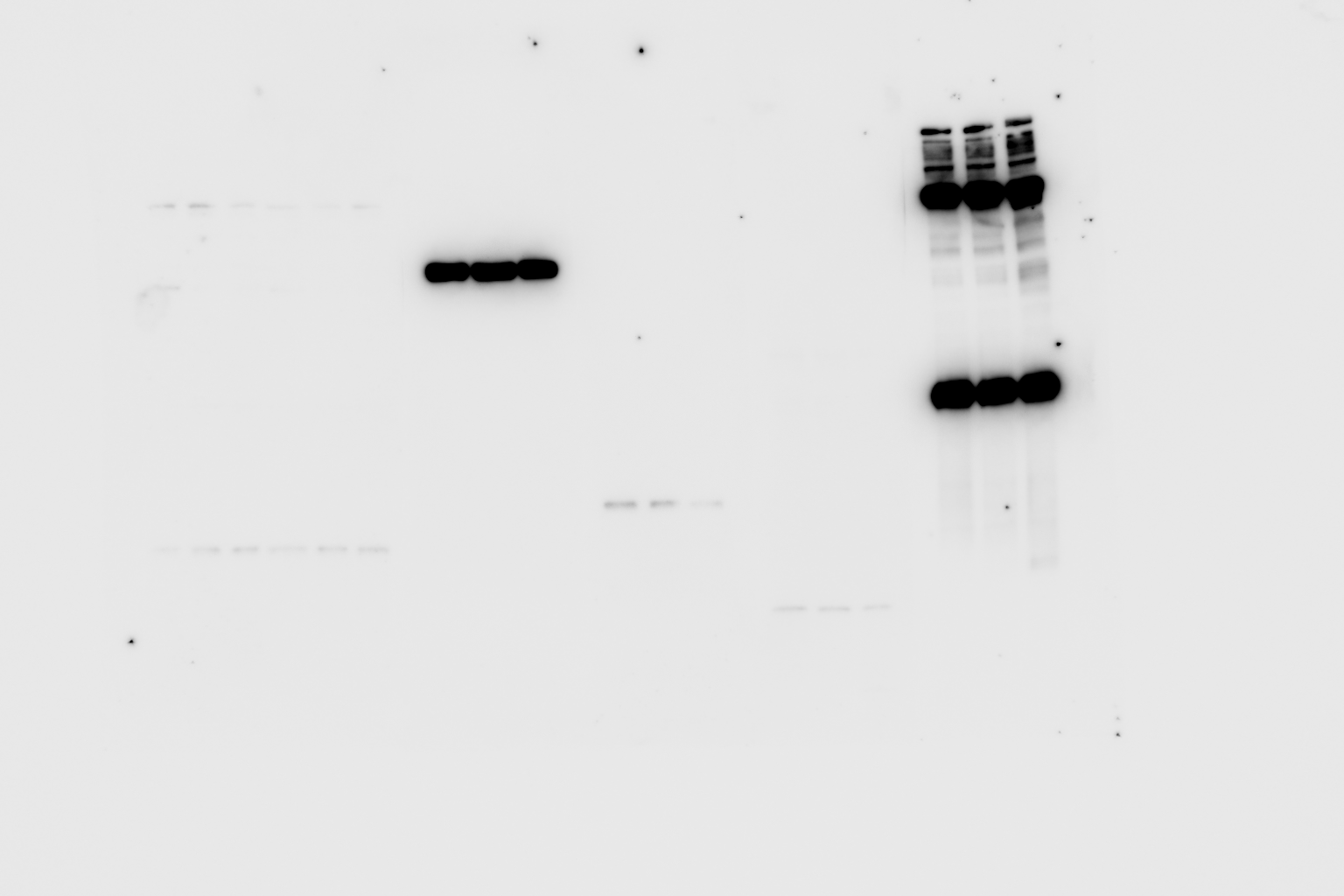

Supplement: Figure 1—source data 5. [file elife-108672-fig1-data5.zip › Fig 1B (part 2)/20210407_Blot4 10min_Sec13.tif]

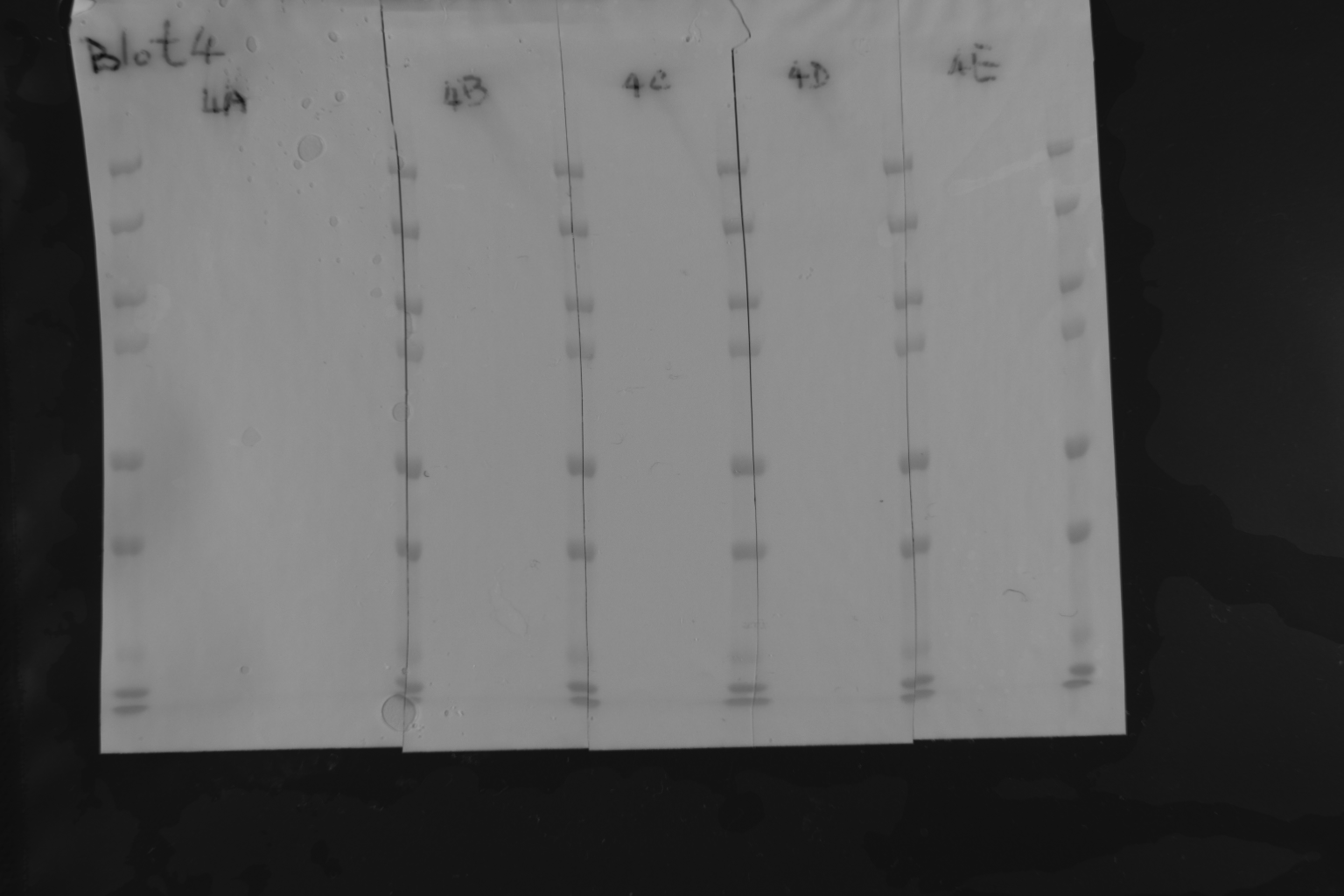

Supplement: Figure 1—source data 5. [file elife-108672-fig1-data5.zip › Fig 1B (part 2)/20210407_Blot4 ladder.tif]

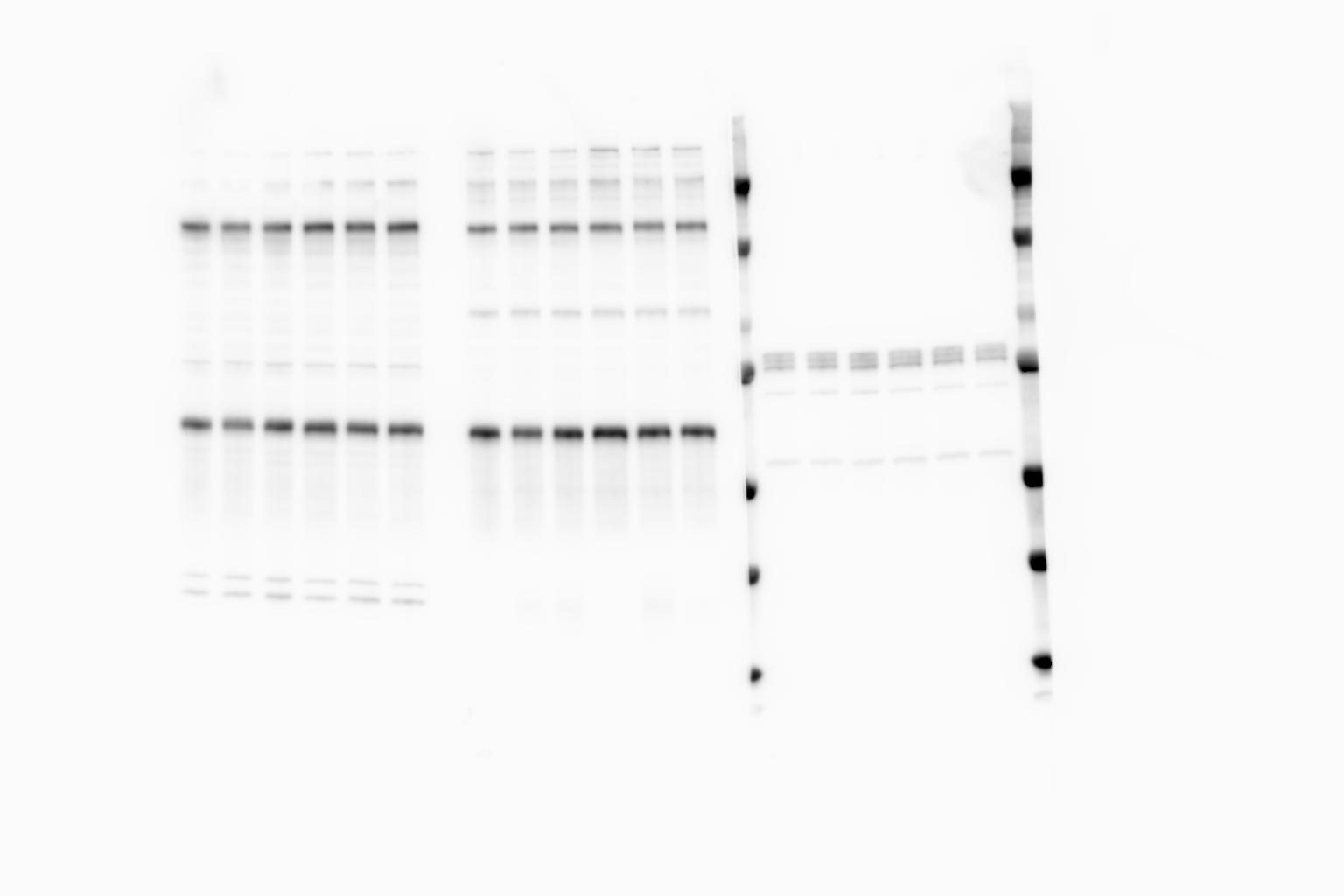

Supplement: Figure 1—source data 5. [file elife-108672-fig1-data5.zip › Fig 1B (part 2)/20210408_overBlot3 1min.tif]

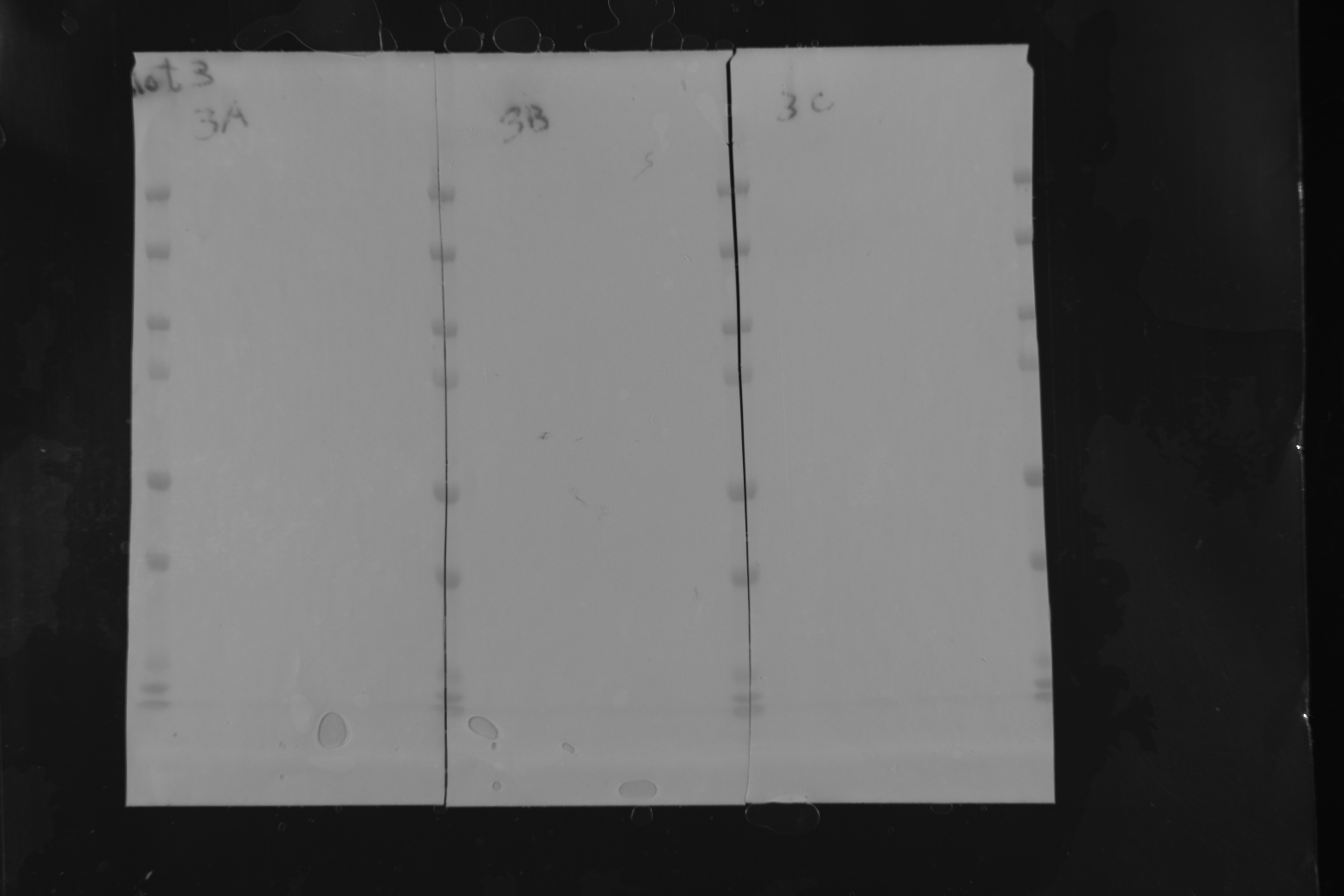

Supplement: Figure 1—source data 5. [file elife-108672-fig1-data5.zip › Fig 1B (part 2)/20210408_overBlot3 ladder.tif]

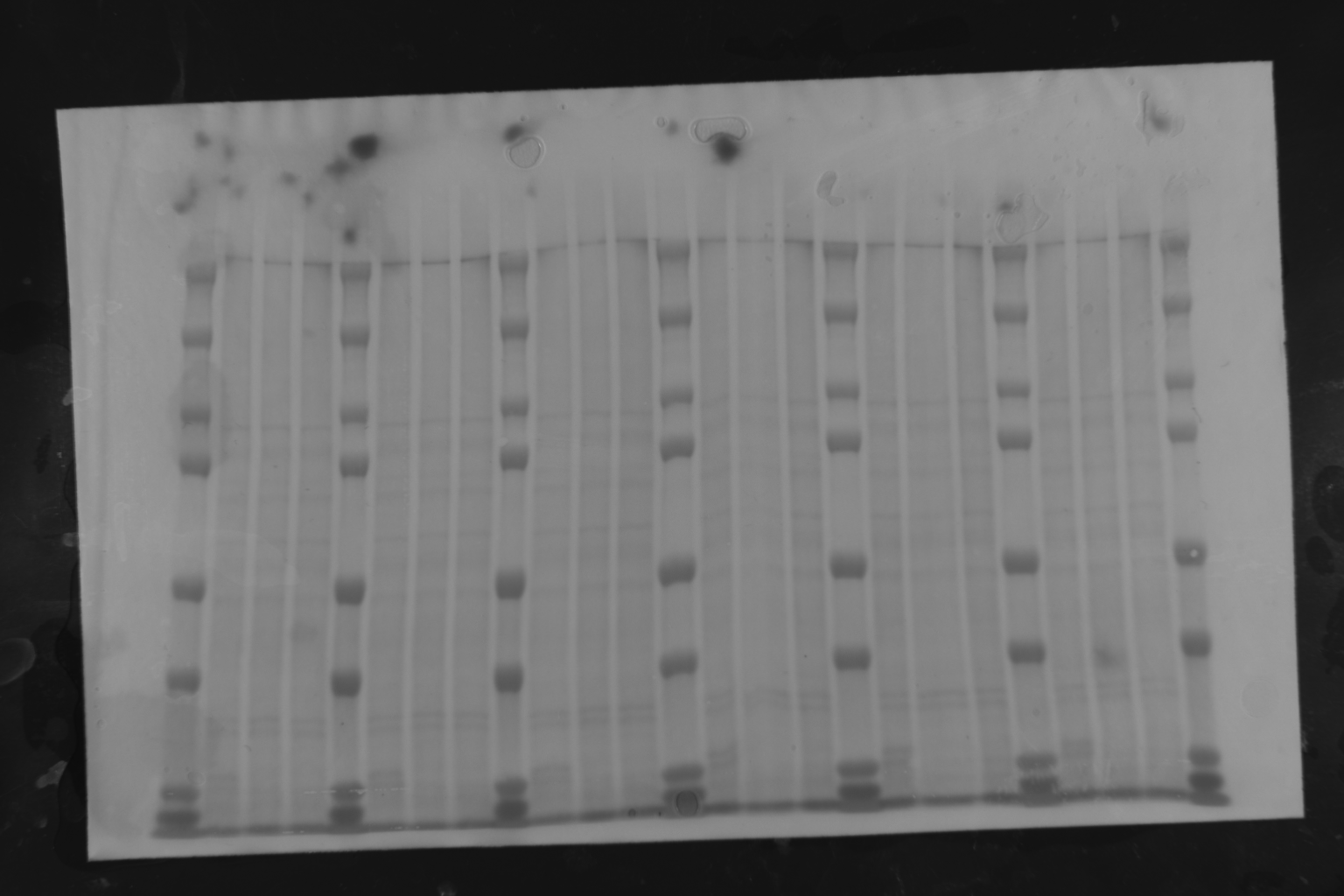

Supplement: Figure 1—source data 5. [file elife-108672-fig1-data5.zip › Fig 1B (part 2)/20210427_1_Ponceau.tif]

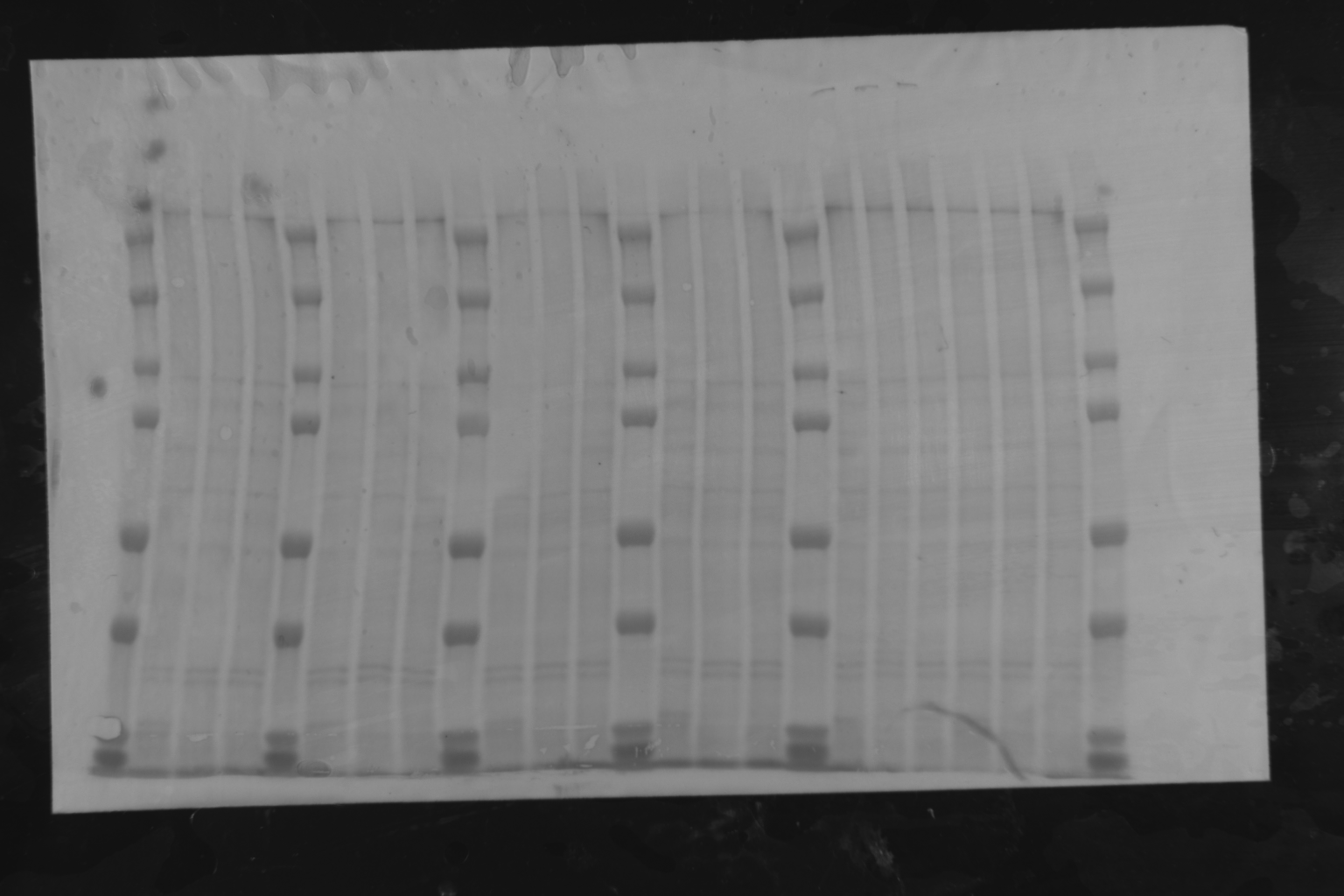

Supplement: Figure 1—source data 5. [file elife-108672-fig1-data5.zip › Fig 1B (part 2)/20210427_2_Ponceau.tif]

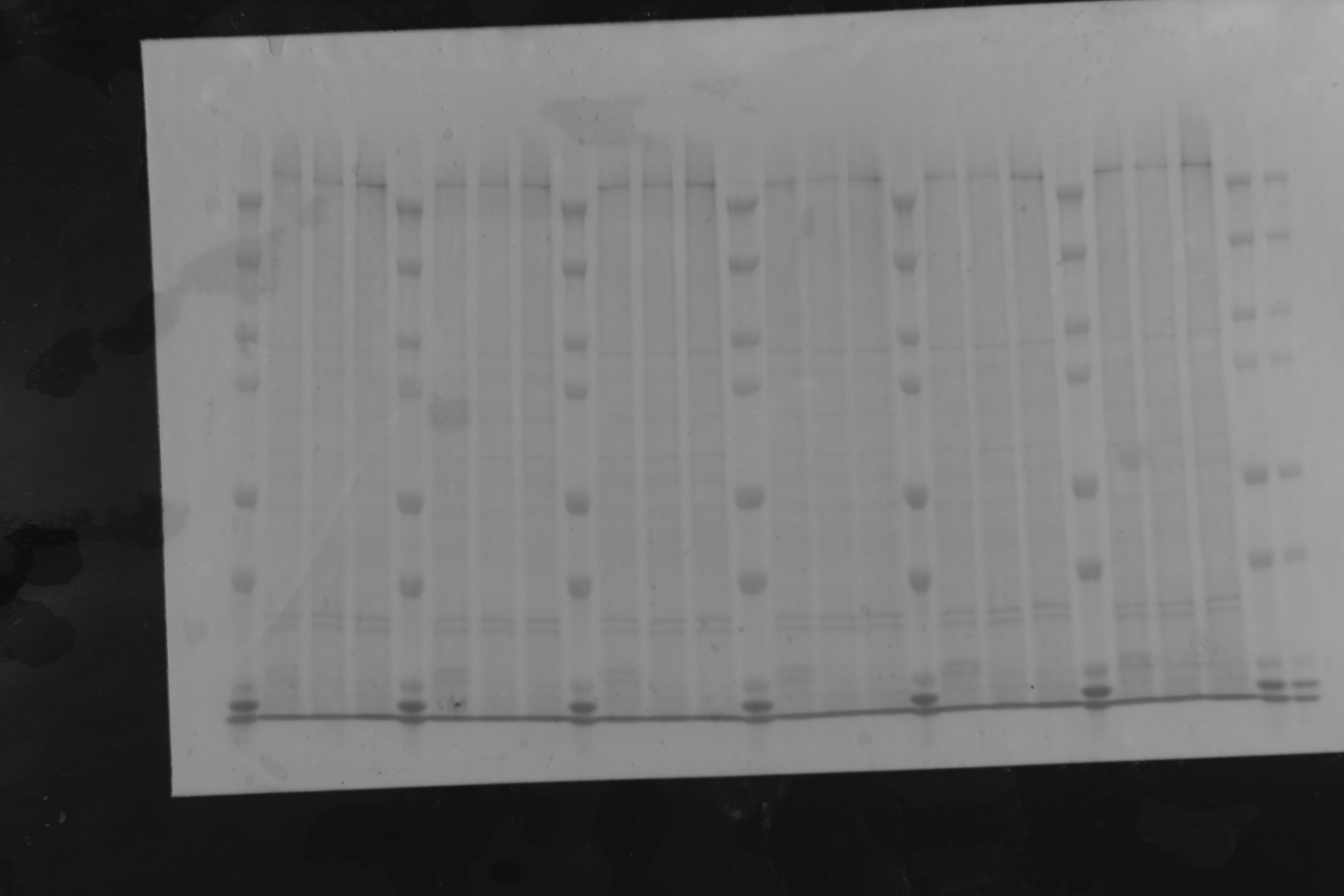

Supplement: Figure 1—source data 5. [file elife-108672-fig1-data5.zip › Fig 1B (part 2)/20210427_Blot3 Ponceau.tif]

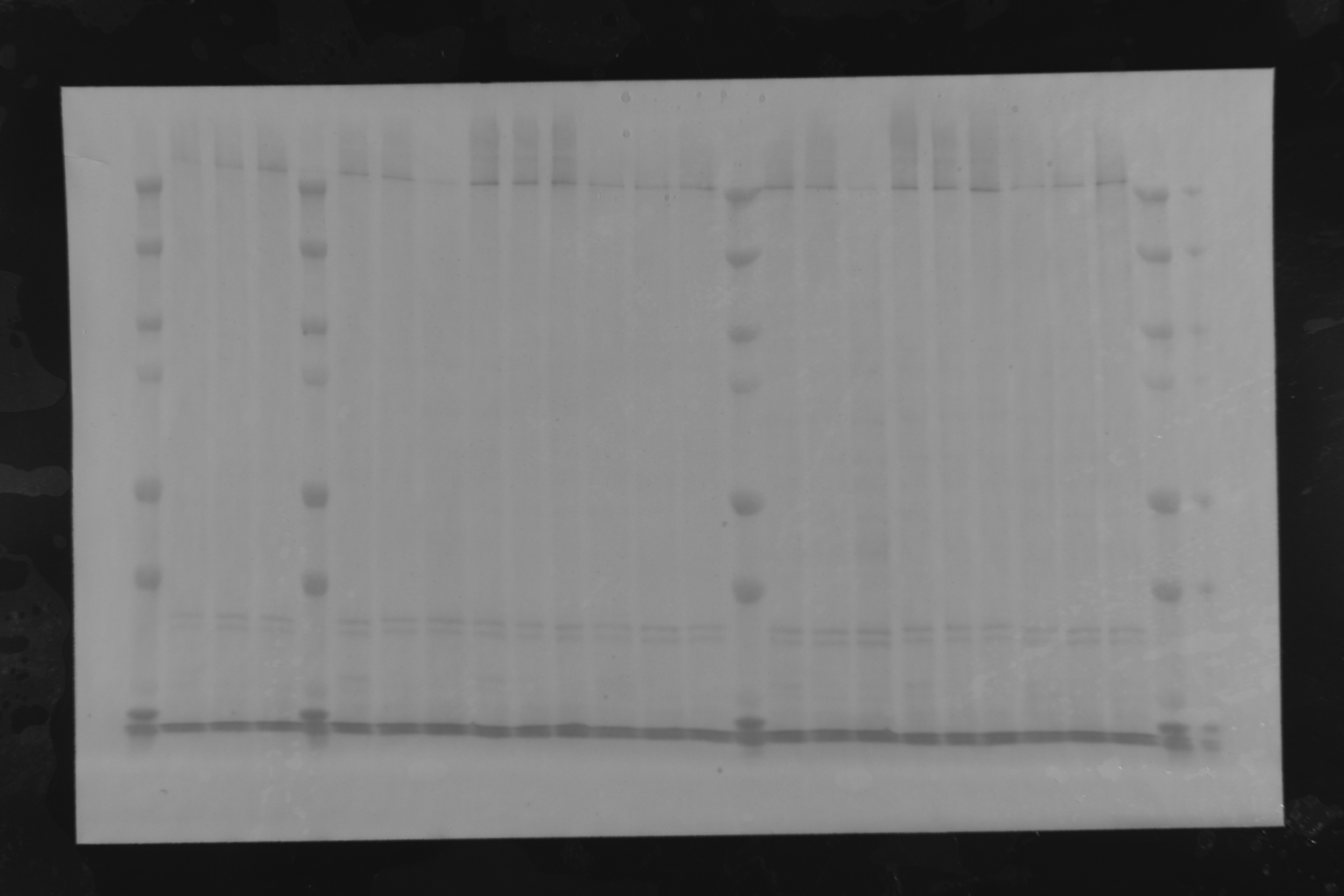

Supplement: Figure 1—source data 5. [file elife-108672-fig1-data5.zip › Fig 1B (part 2)/20210427_Blot4 Ponceau.tif]

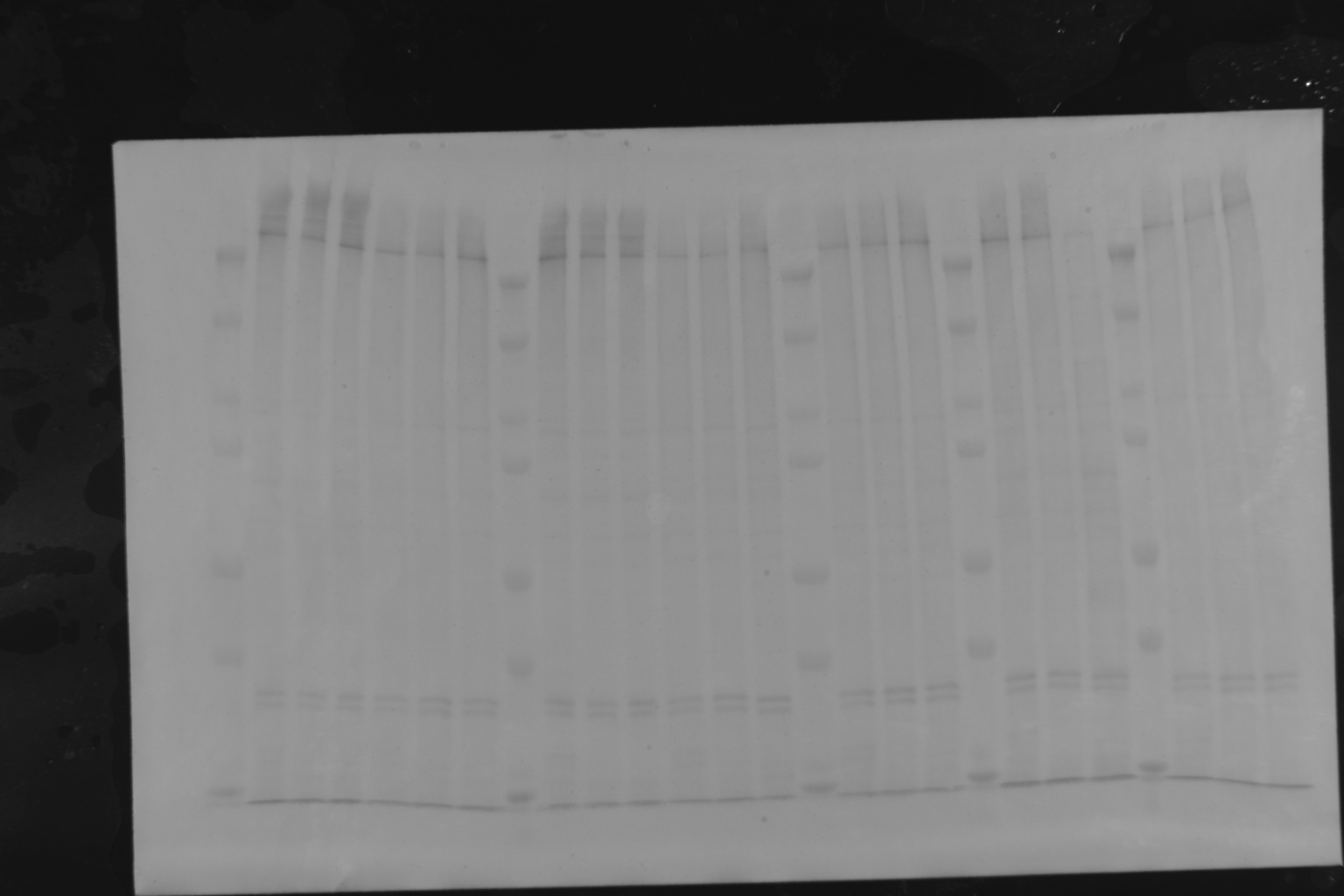

Supplement: Figure 1—source data 5. [file elife-108672-fig1-data5.zip › Fig 1B (part 2)/20210427_Blot5 Ponceau.tif]

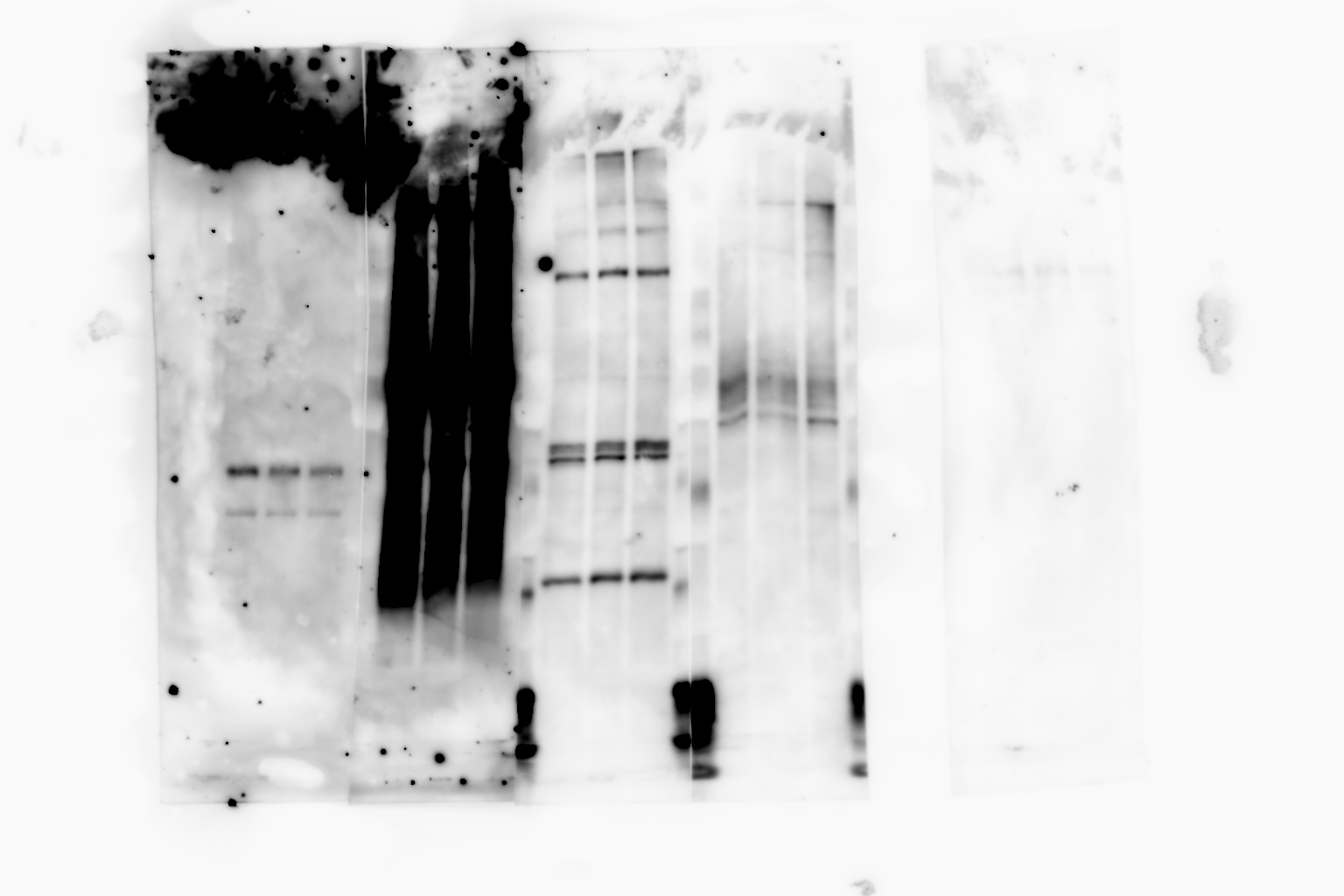

Supplement: Figure 1—source data 5. [file elife-108672-fig1-data5.zip › Fig 1B (part 2)/20210428_1AD2Aoverblot_10min_POM121.tif]

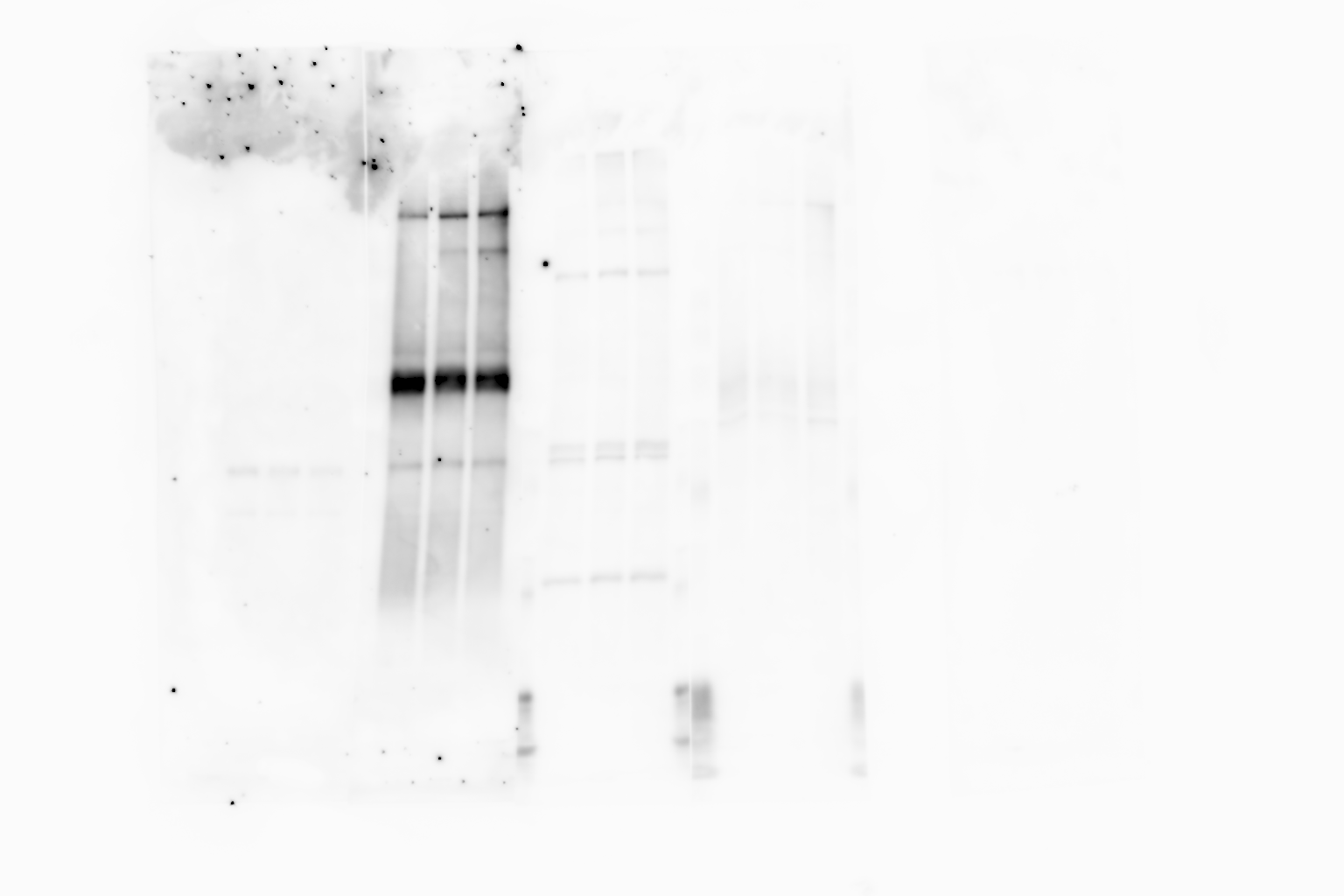

Supplement: Figure 1—source data 5. [file elife-108672-fig1-data5.zip › Fig 1B (part 2)/20210428_1AD2Aoverblot_1min.tif]

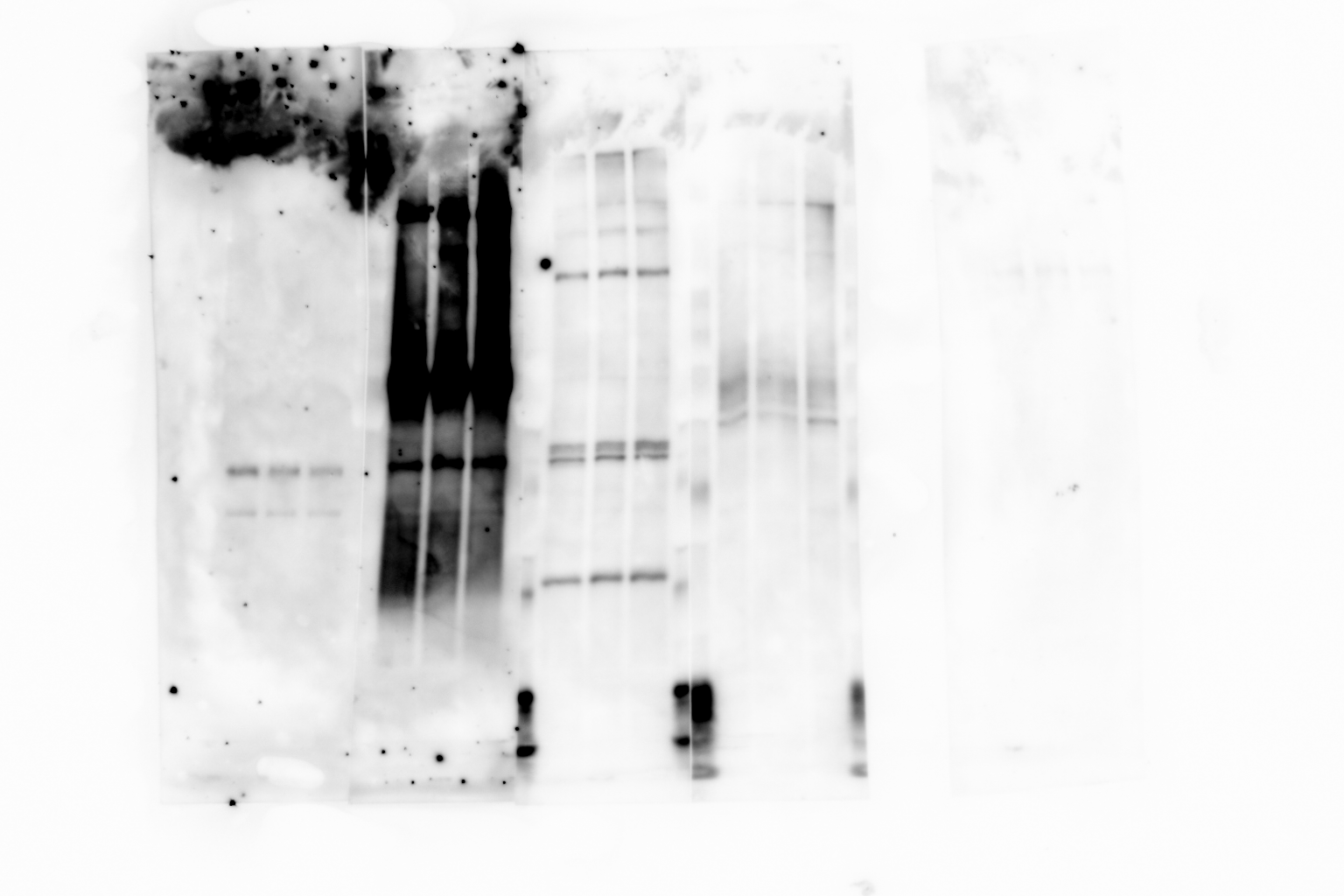

Supplement: Figure 1—source data 5. [file elife-108672-fig1-data5.zip › Fig 1B (part 2)/20210428_1AD2Aoverblot_1min_Nup62.tif]

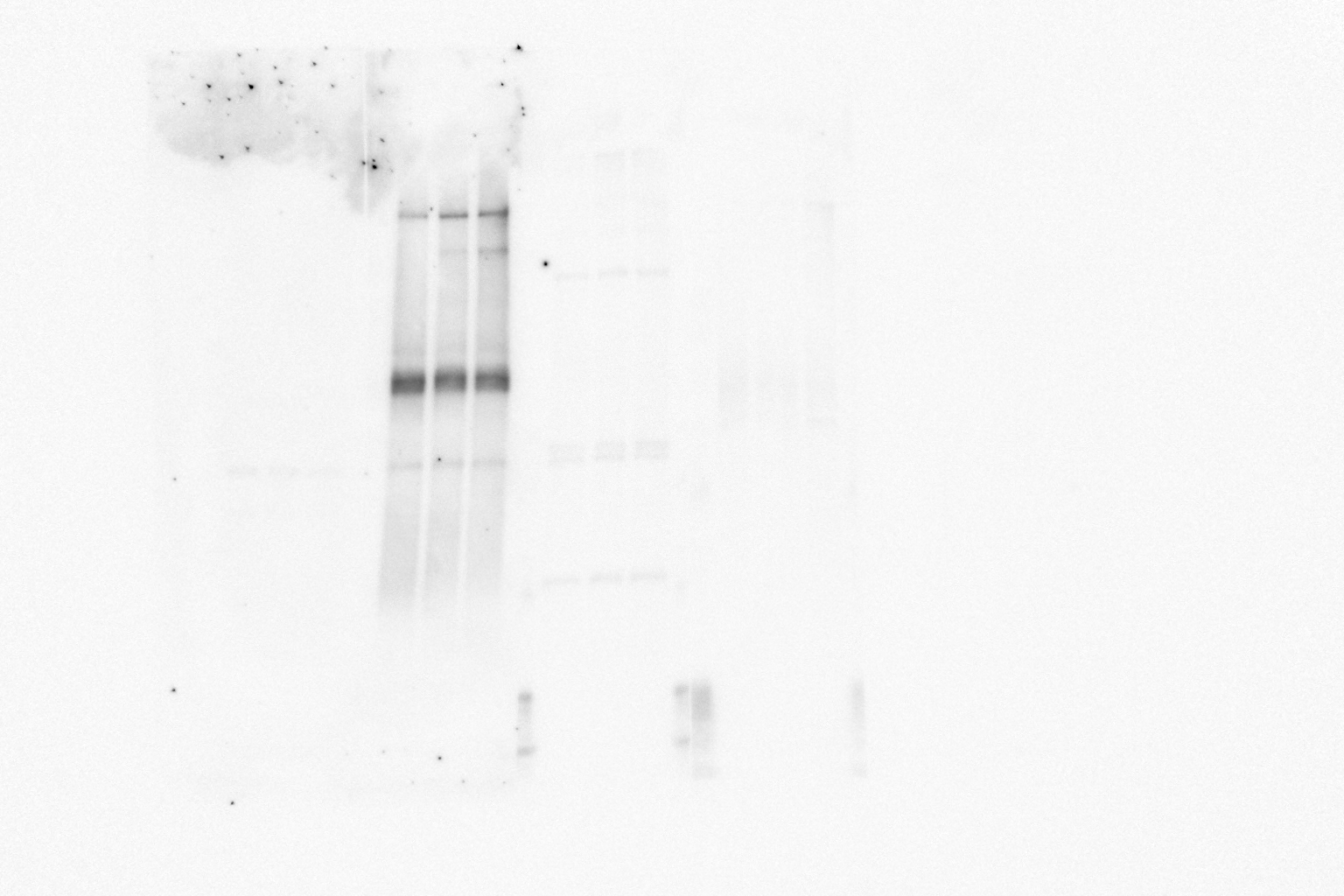

Supplement: Figure 1—source data 5. [file elife-108672-fig1-data5.zip › Fig 1B (part 2)/20210428_1AD2Aoverblot_1sec_Nup98.tif]

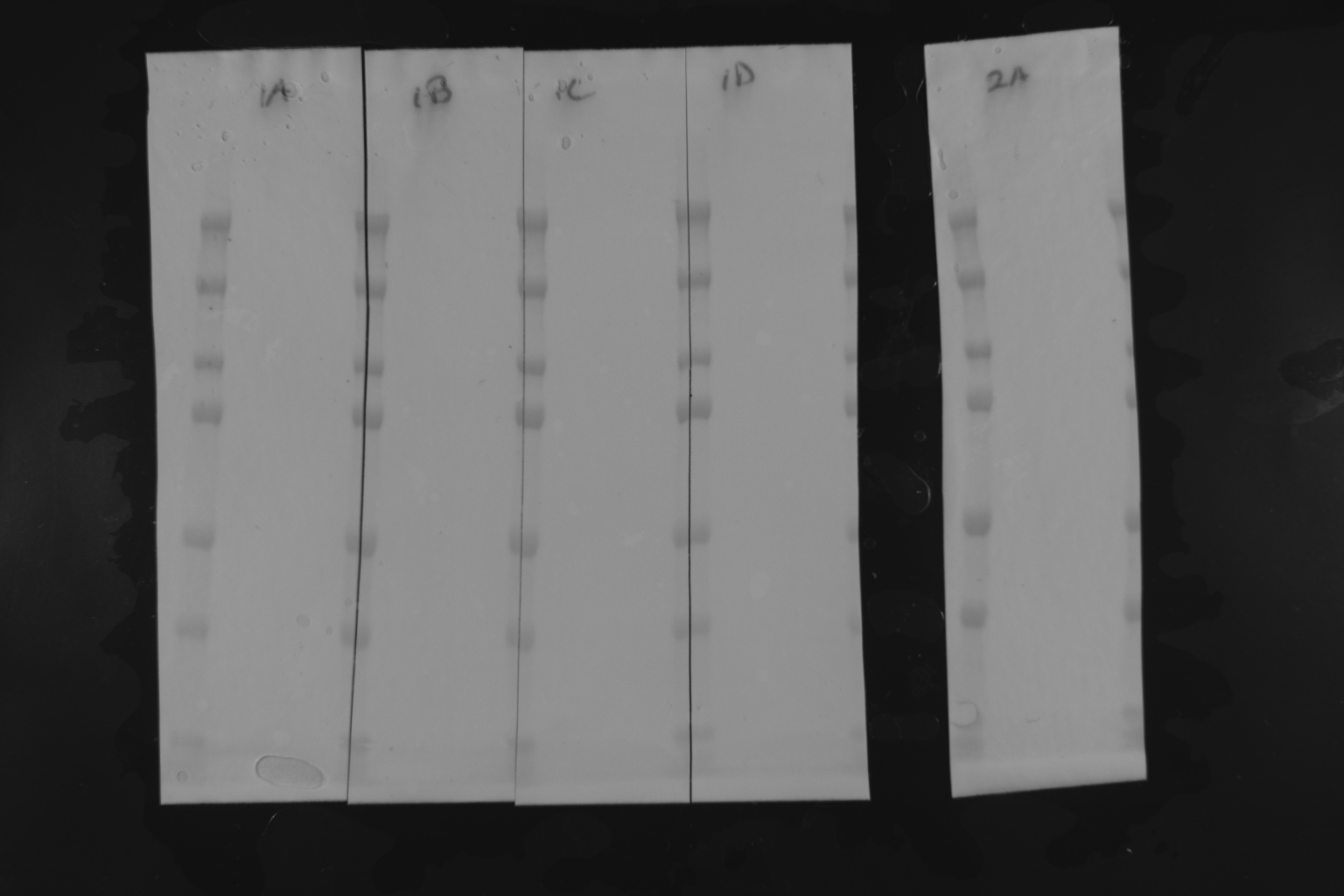

Supplement: Figure 1—source data 5. [file elife-108672-fig1-data5.zip › Fig 1B (part 2)/20210428_1AD2Aoverblot_ladder.tif]

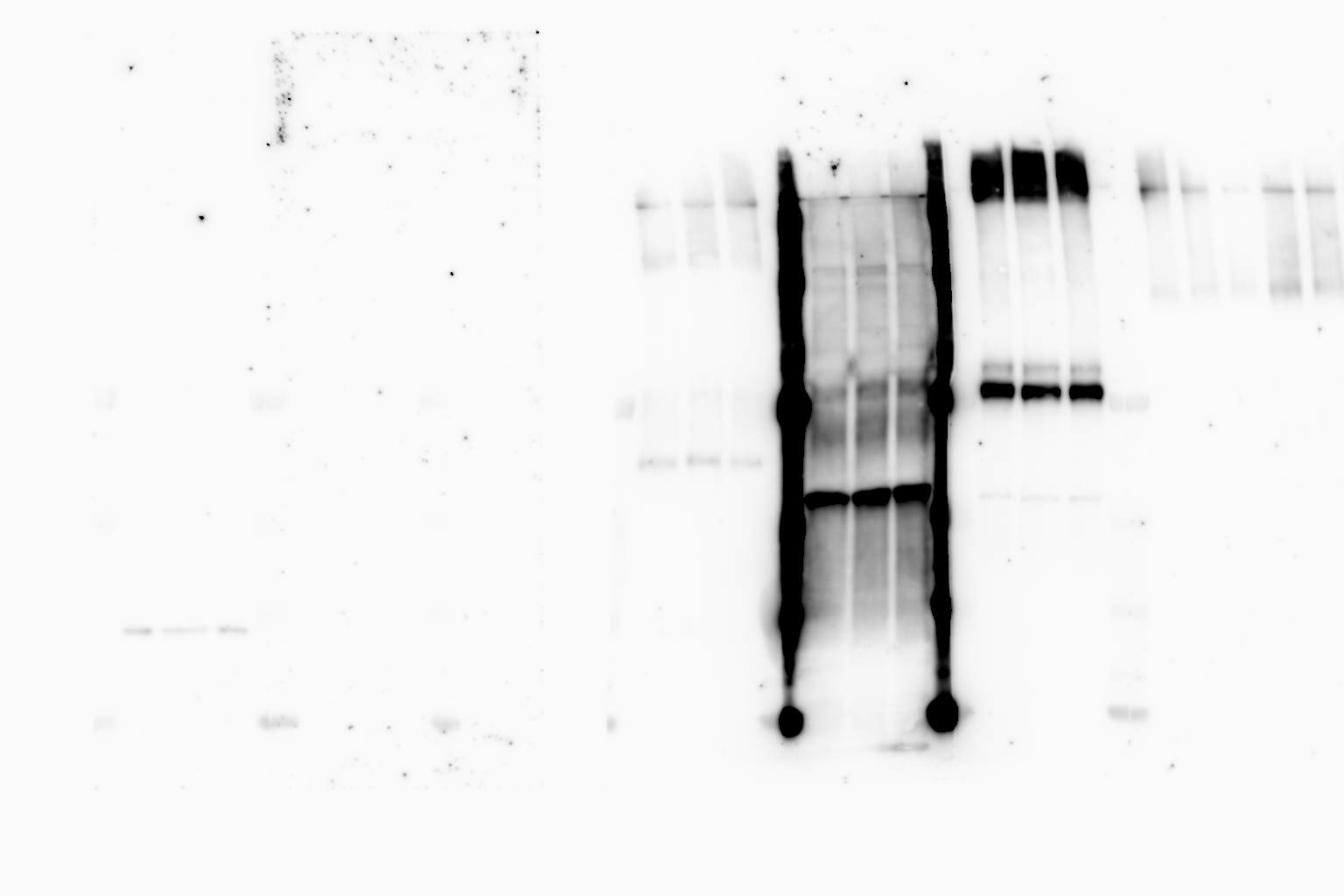

Supplement: Figure 1—source data 5. [file elife-108672-fig1-data5.zip › Fig 1B (part 2)/20210428_1EF2BEoverblot_10min.tif]

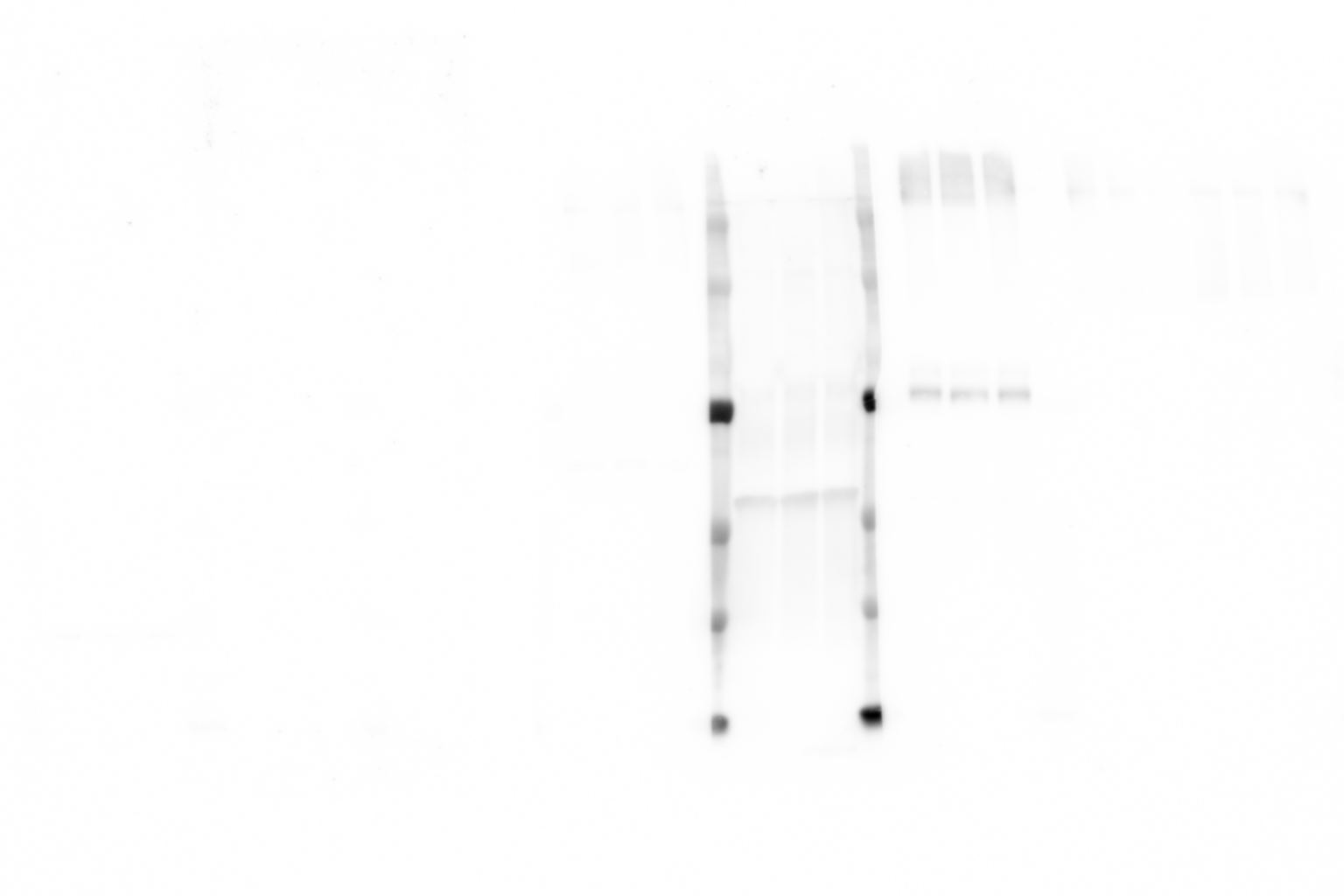

Supplement: Figure 1—source data 5. [file elife-108672-fig1-data5.zip › Fig 1B (part 2)/20210428_1EF2BEoverblot_15sec.tif]

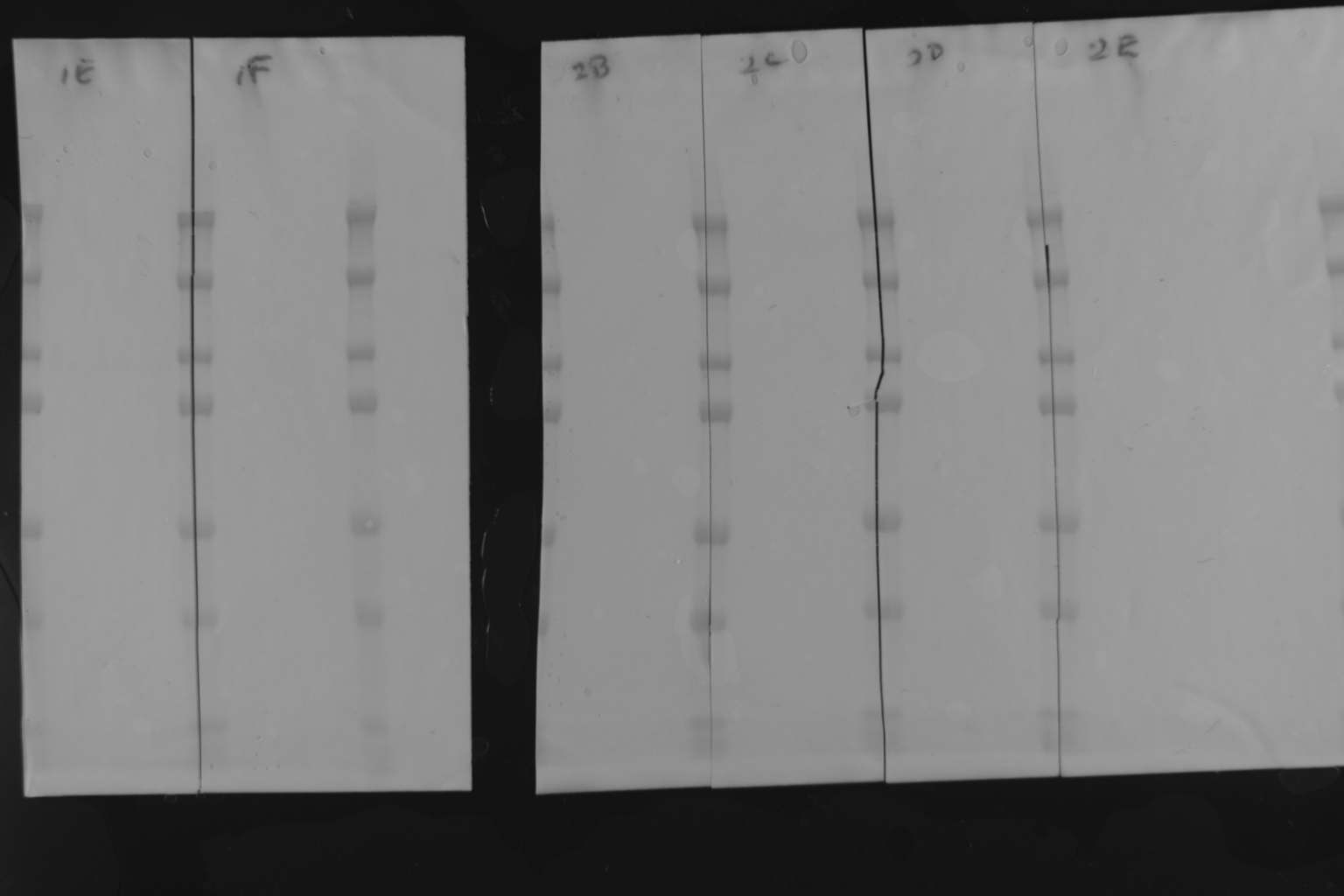

Supplement: Figure 1—source data 5. [file elife-108672-fig1-data5.zip › Fig 1B (part 2)/20210428_1EF2BEoverblot_ladder2.tif]

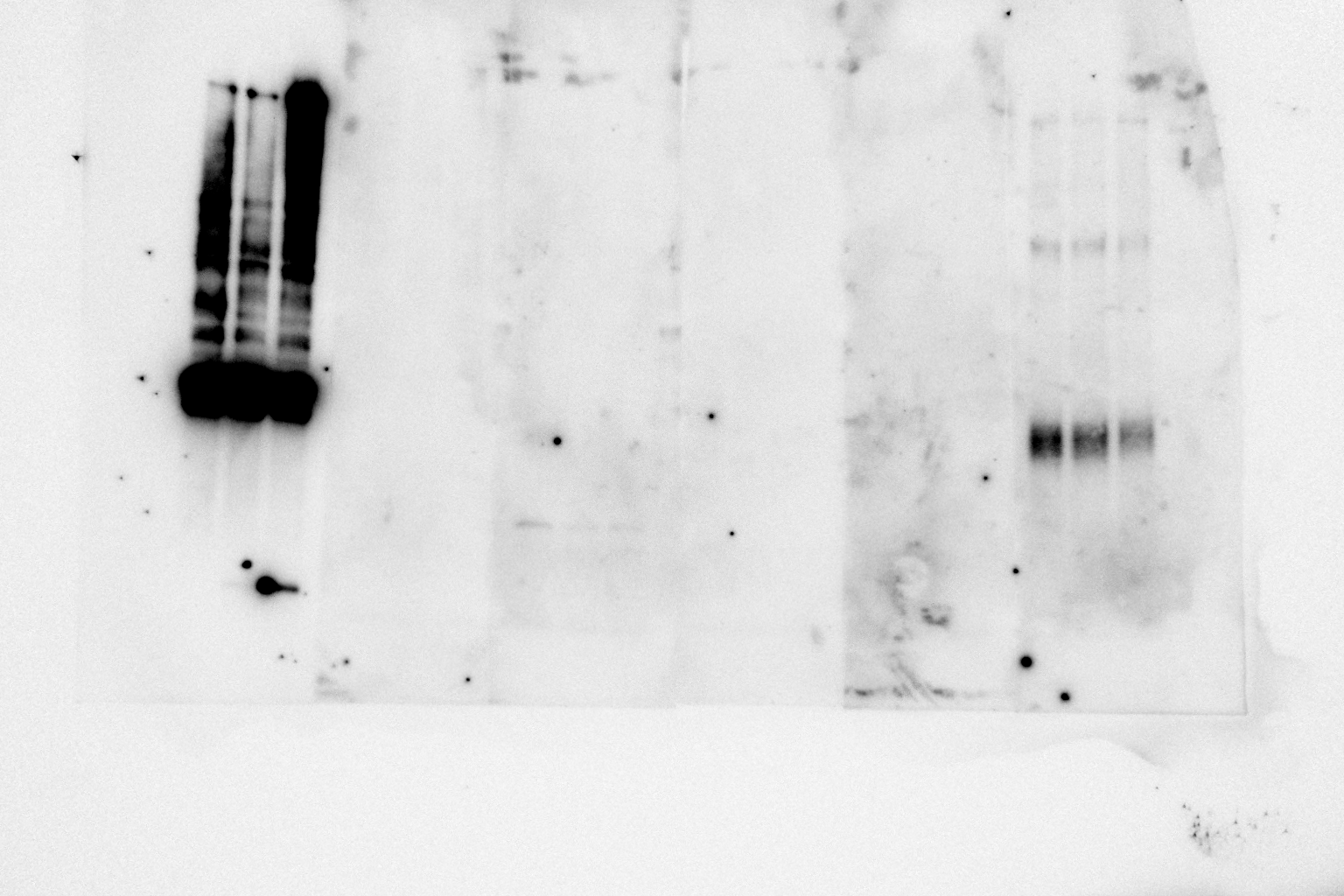

Supplement: Figure 1—source data 5. [file elife-108672-fig1-data5.zip › Fig 1B (part 2)/20210428_Blot3 10min_Nup35.tif]

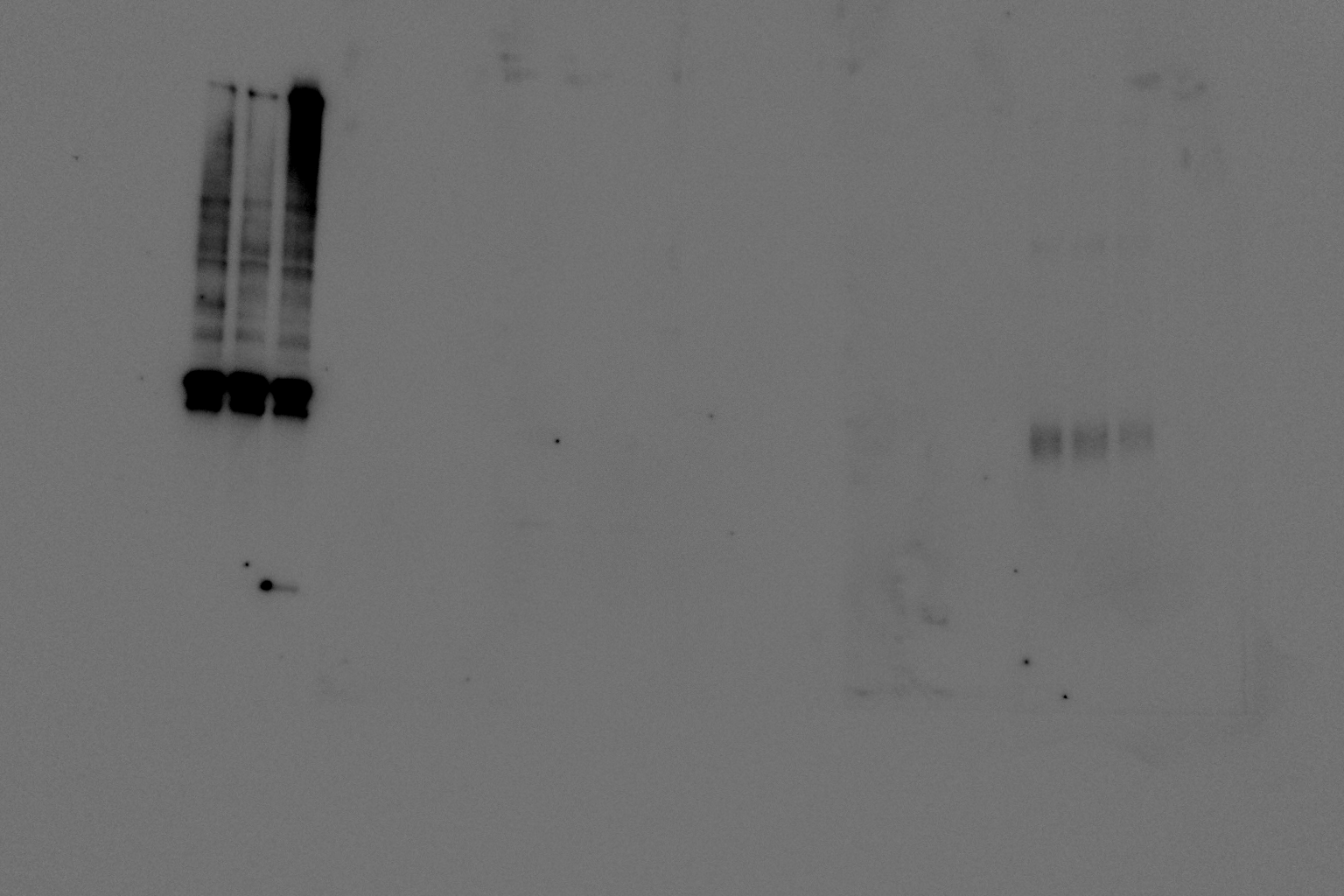

Supplement: Figure 1—source data 5. [file elife-108672-fig1-data5.zip › Fig 1B (part 2)/20210428_Blot3 1min_aladin.tif]

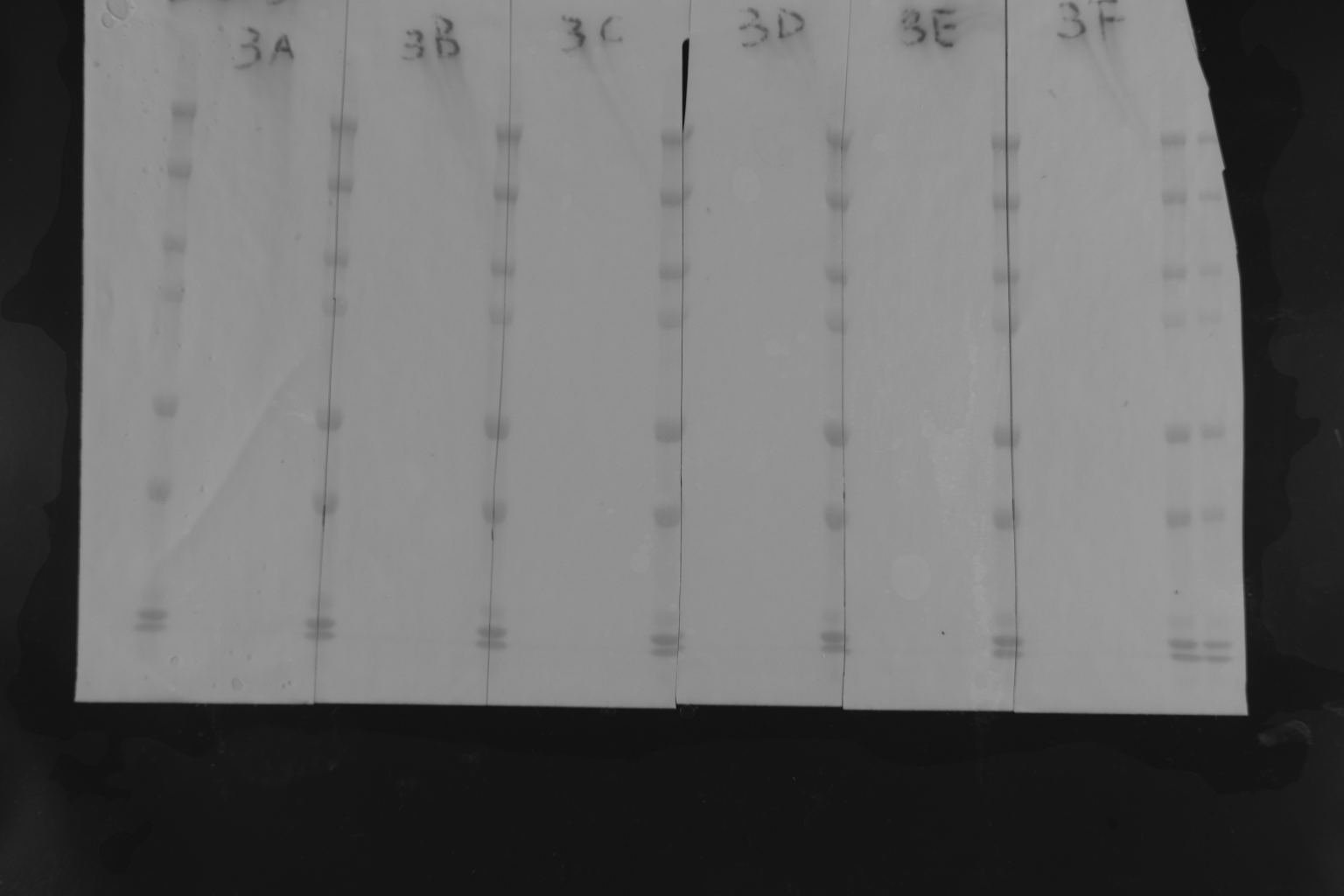

Supplement: Figure 1—source data 6. [file elife-108672-fig1-data6.zip › Fig 1B (part 3)/20210428_Blot3 ladder.tif]

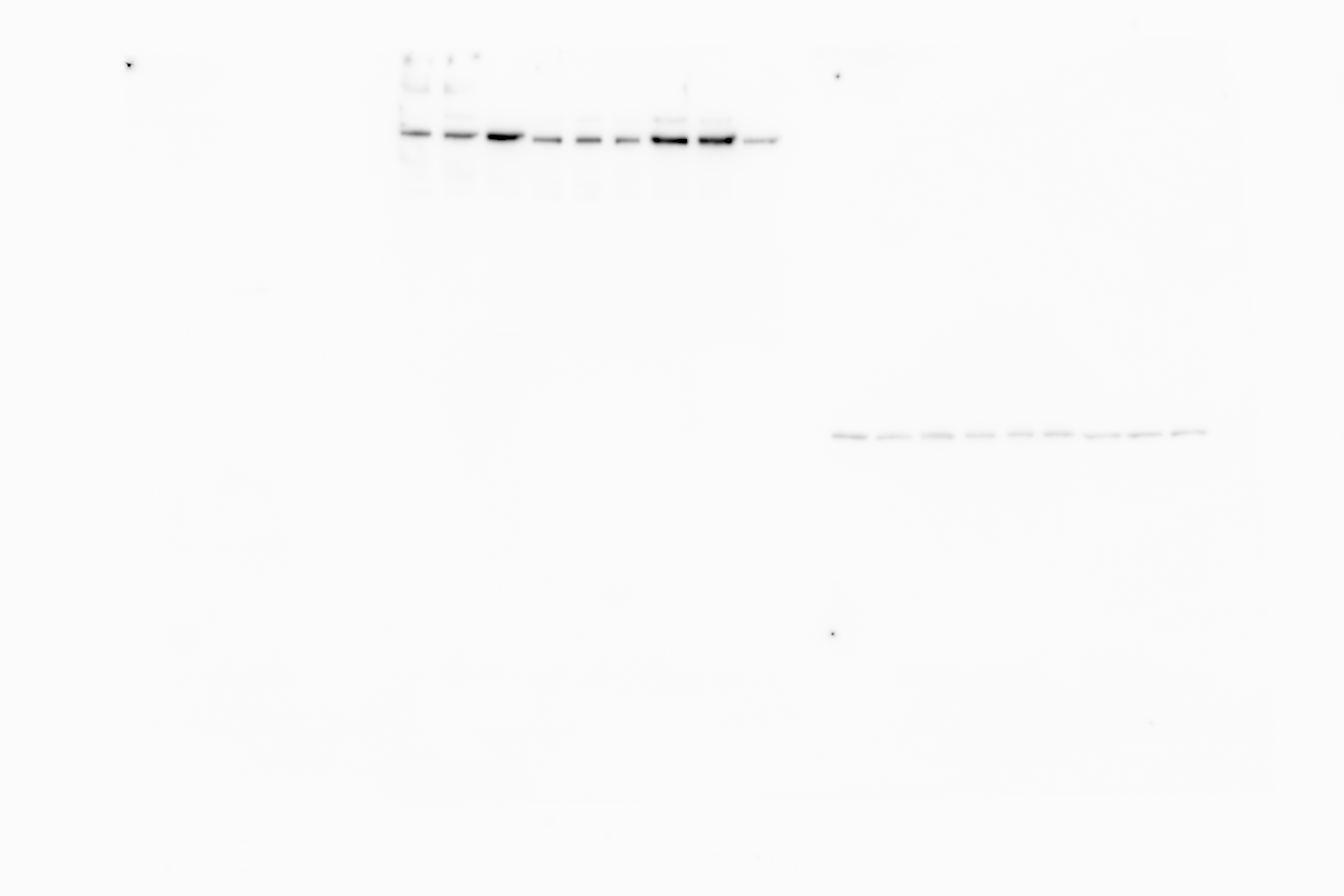

Supplement: Figure 1—source data 6. [file elife-108672-fig1-data6.zip › Fig 1B (part 3)/20210428_Blot4 10min.tif]

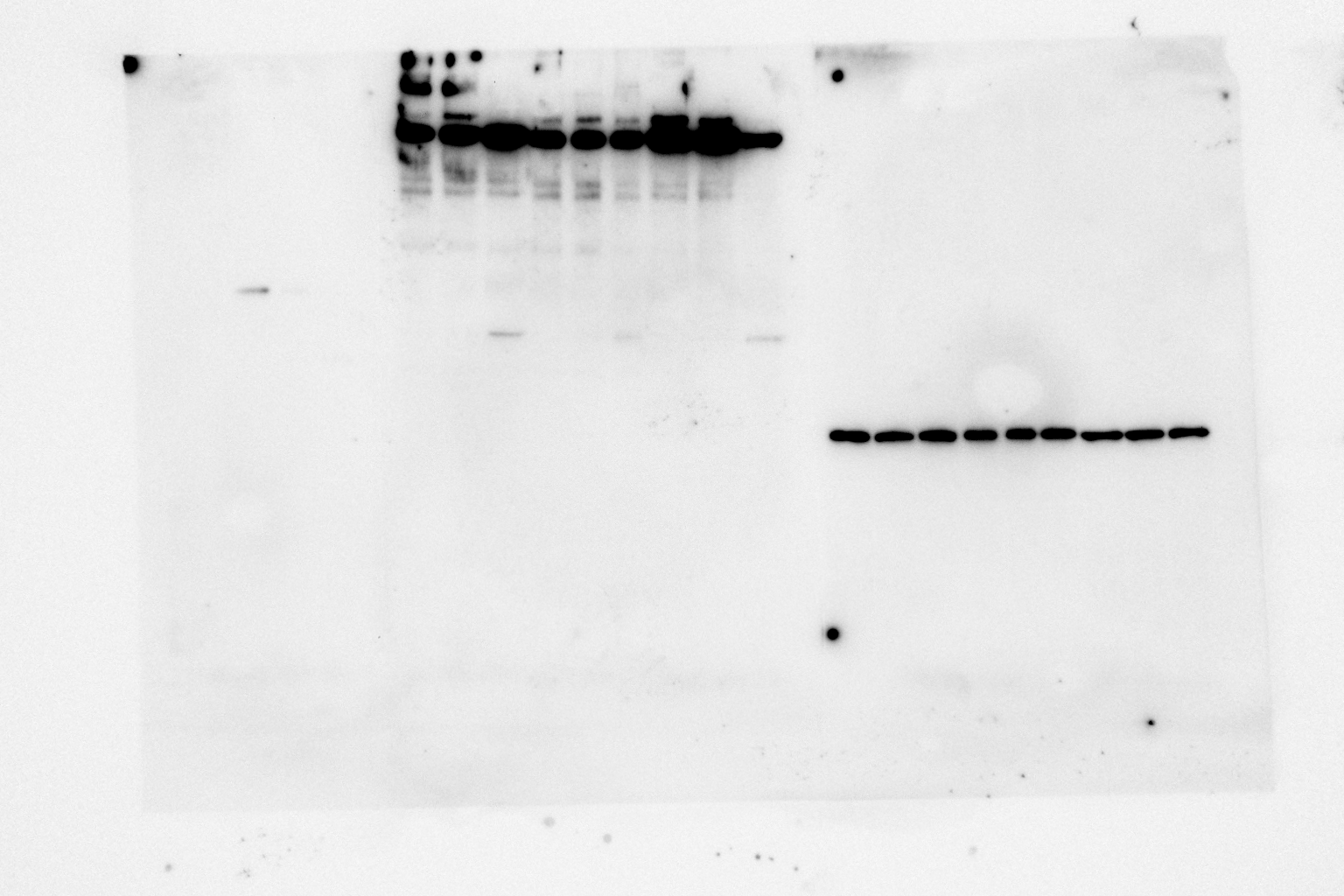

Supplement: Figure 1—source data 6. [file elife-108672-fig1-data6.zip › Fig 1B (part 3)/20210428_Blot4 10min_NDC1.tif]

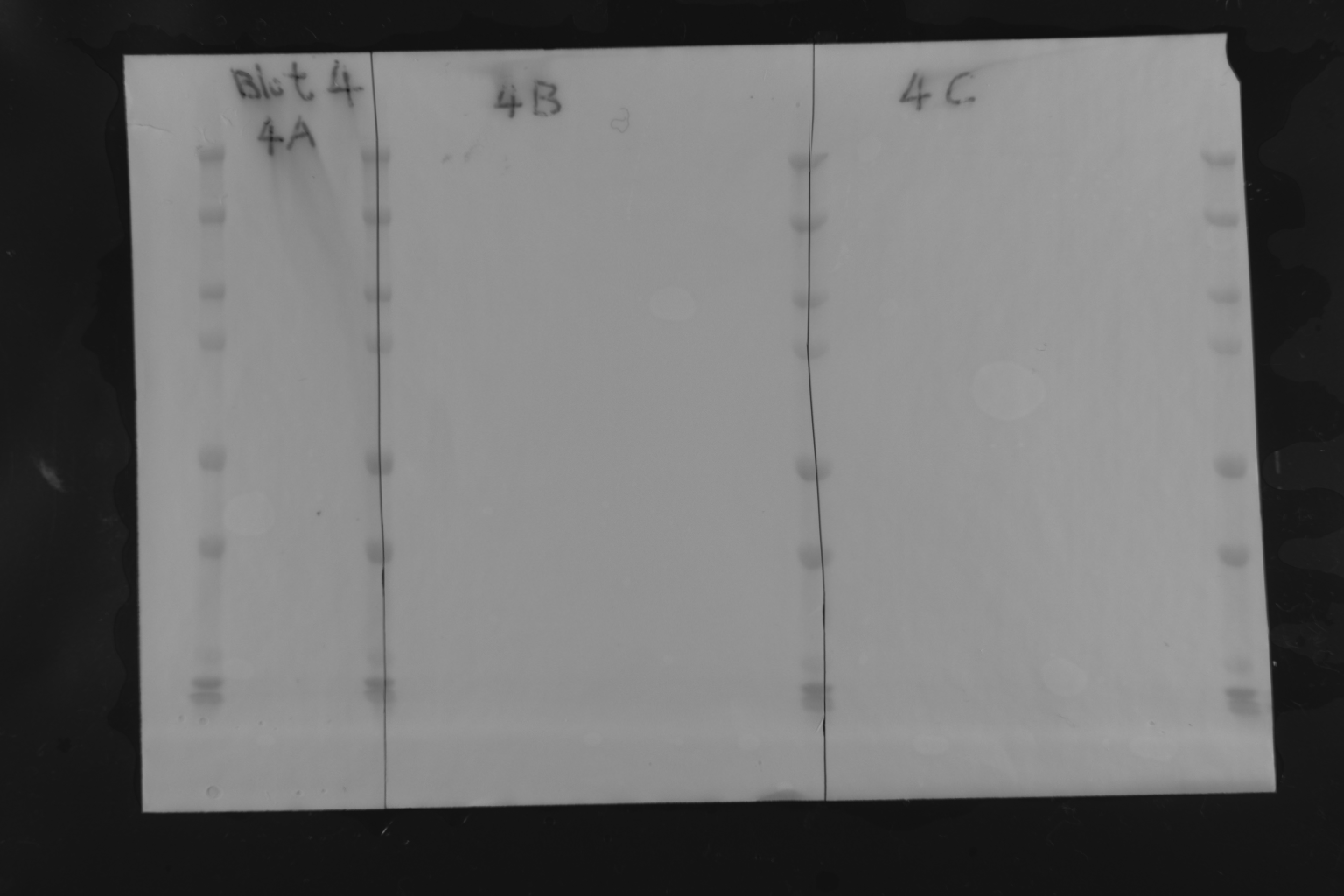

Supplement: Figure 1—source data 6. [file elife-108672-fig1-data6.zip › Fig 1B (part 3)/20210428_Blot4 ladder.tif]

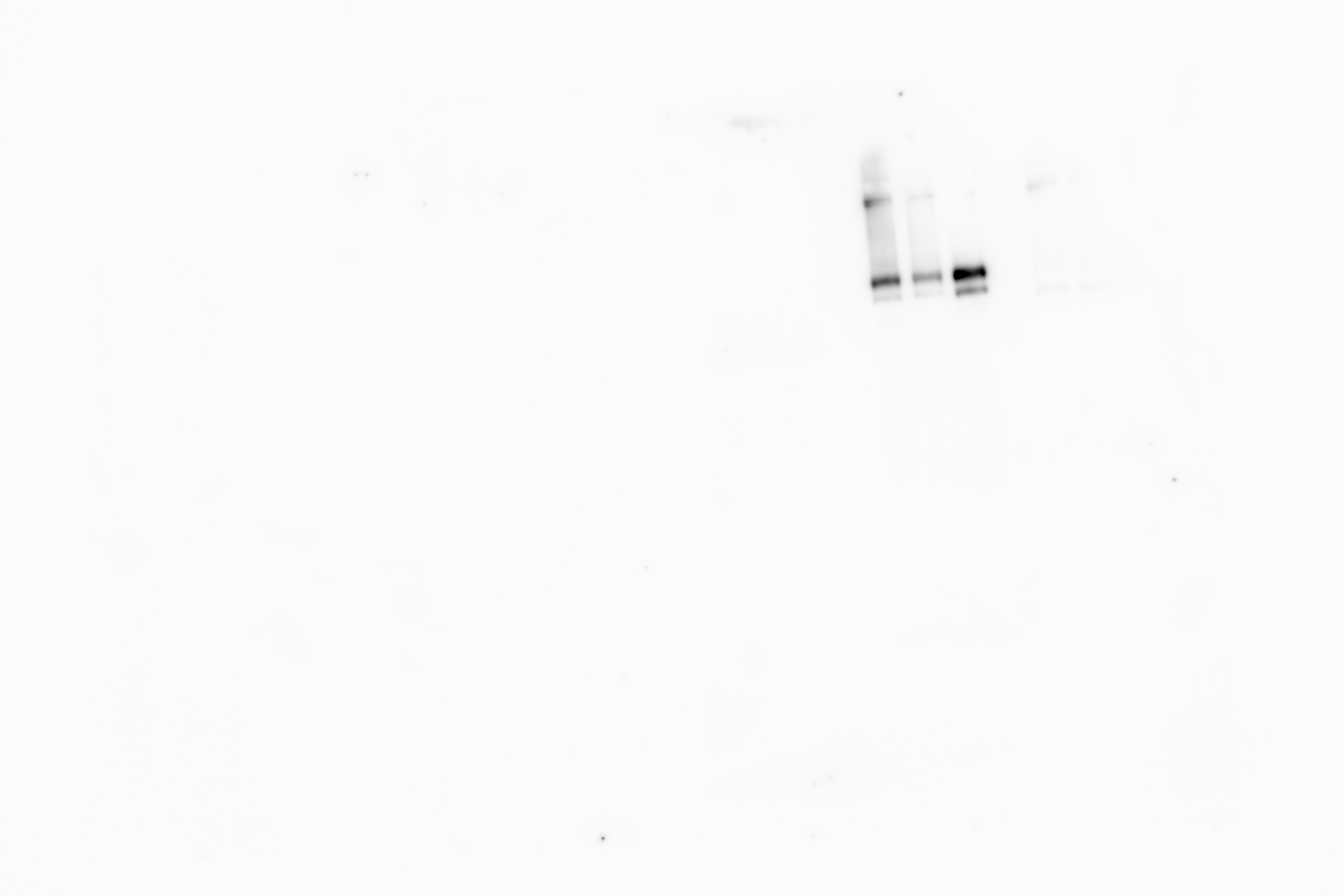

Supplement: Figure 1—source data 6. [file elife-108672-fig1-data6.zip › Fig 1B (part 3)/20210428_Blot5 10min.tif]

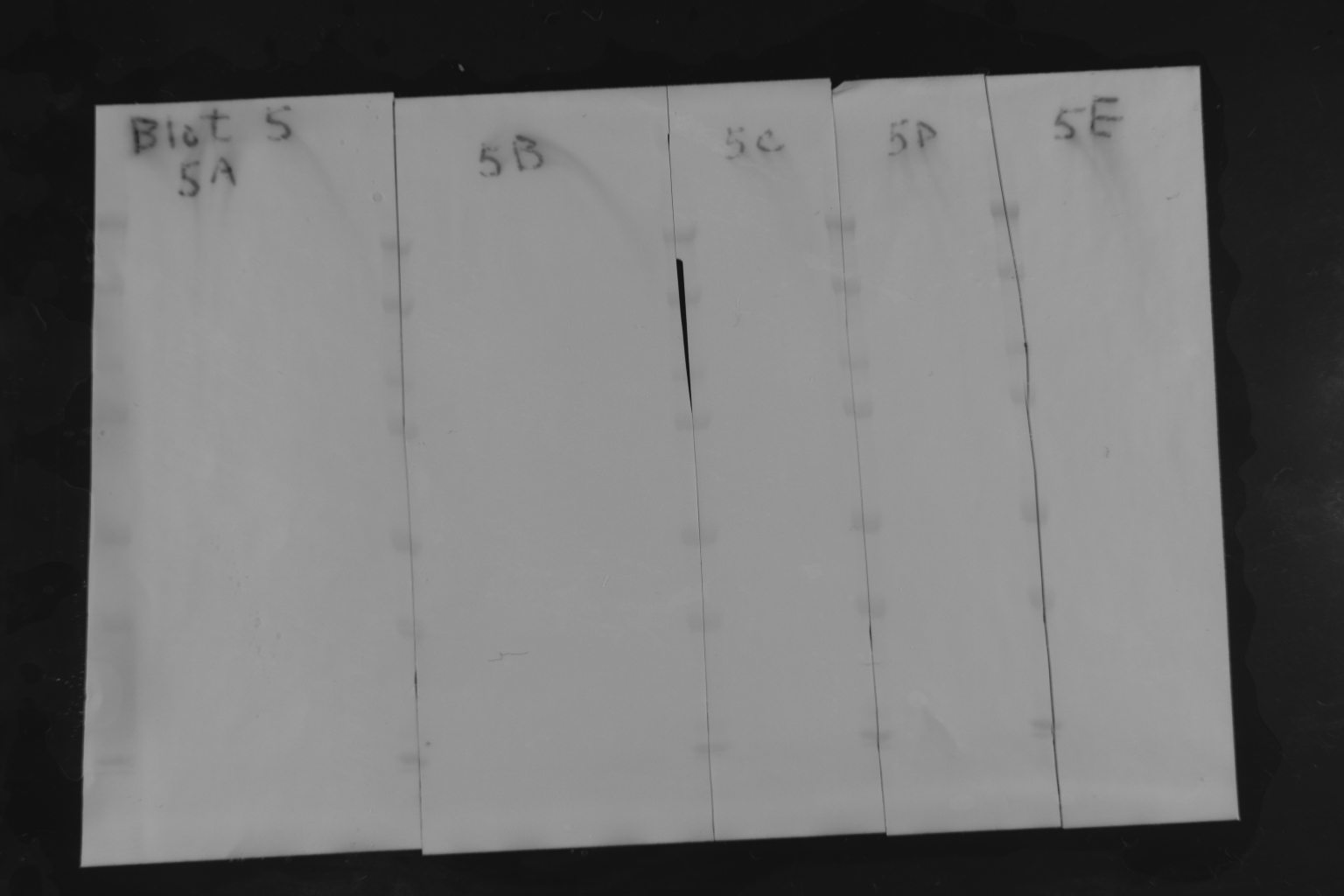

Supplement: Figure 1—source data 6. [file elife-108672-fig1-data6.zip › Fig 1B (part 3)/20210428_Blot5 ladder.tif]

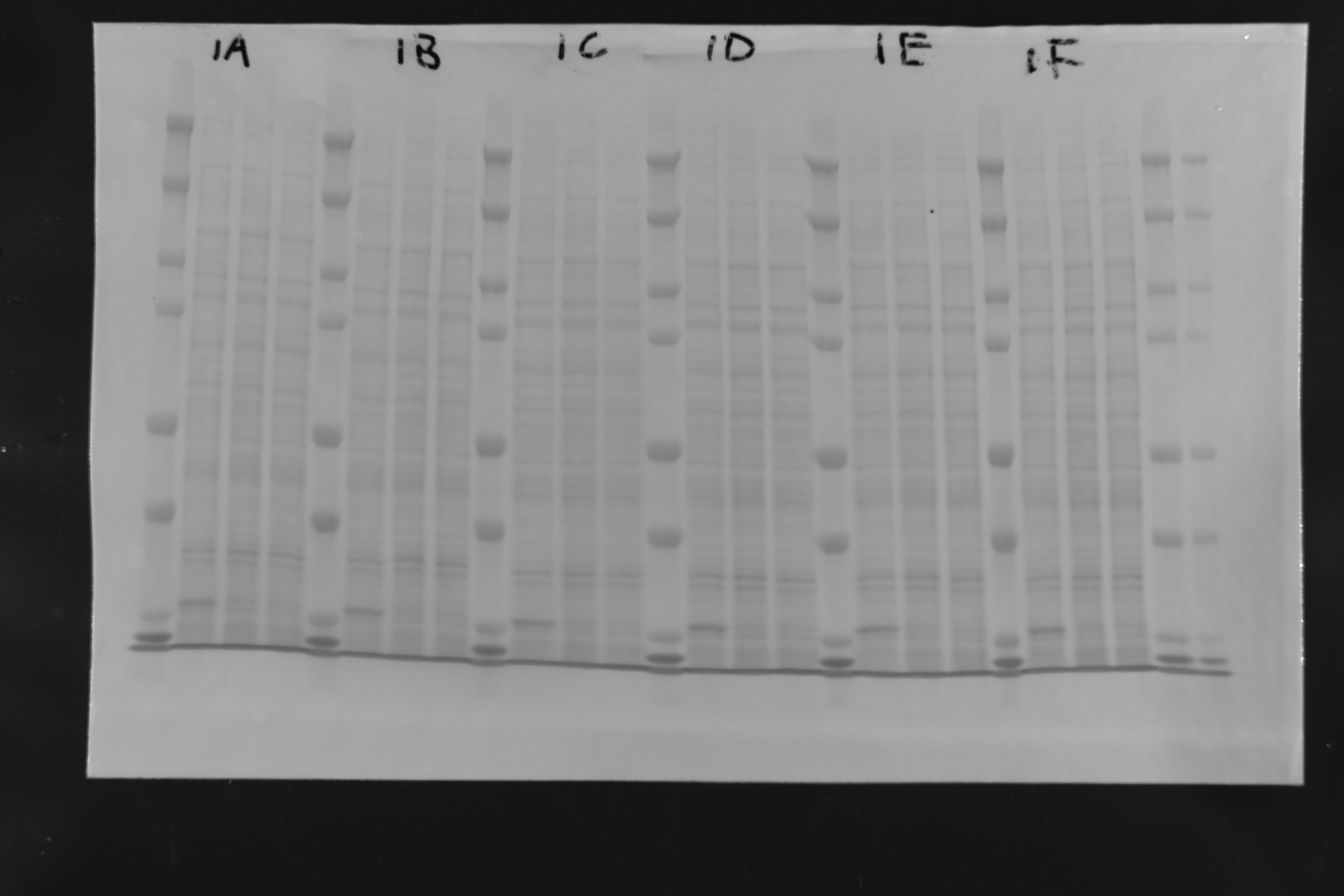

Supplement: Figure 1—source data 6. [file elife-108672-fig1-data6.zip › Fig 1B (part 3)/20210519_Blot1 Ponceau s.tif]

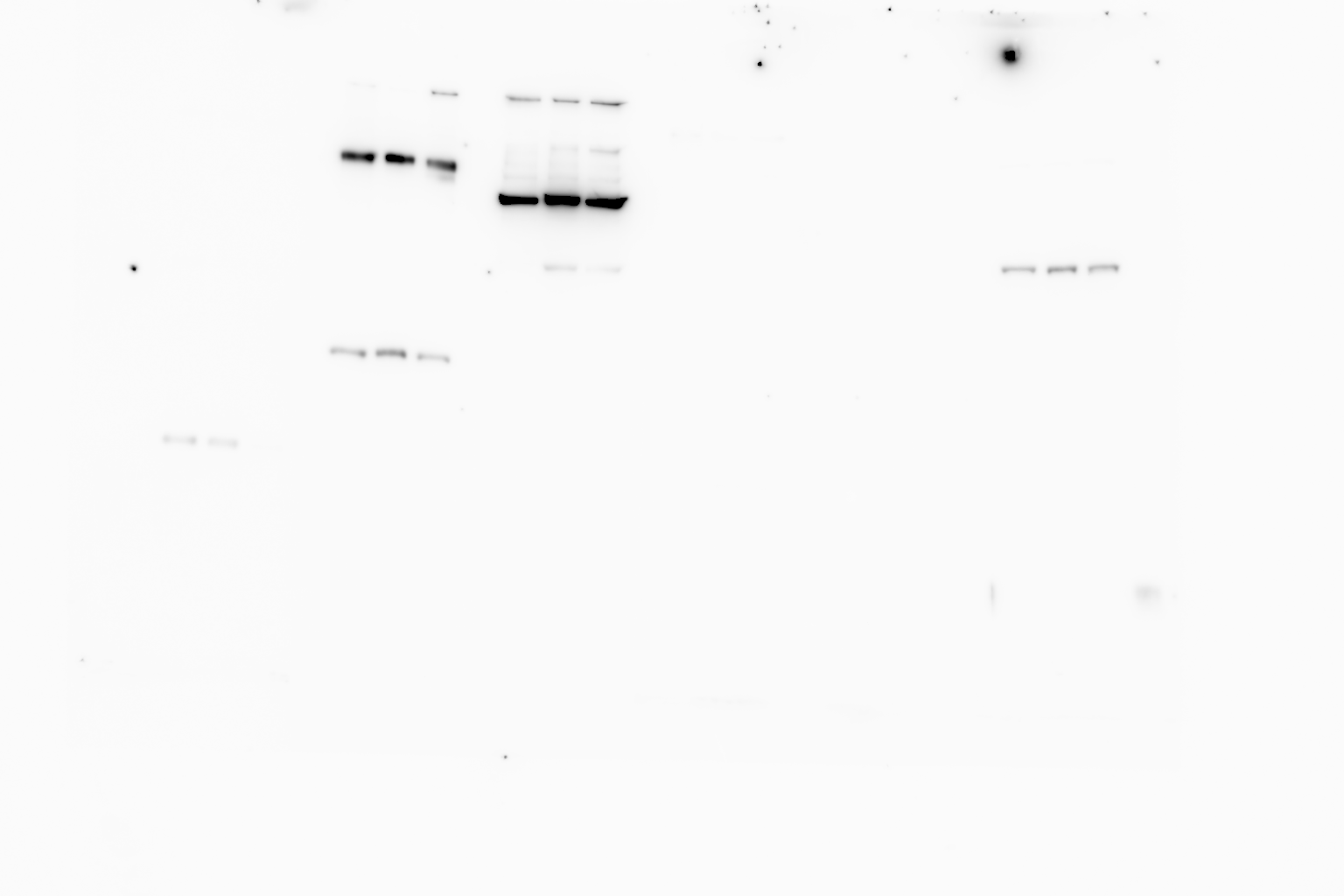

Supplement: Figure 1—source data 6. [file elife-108672-fig1-data6.zip › Fig 1B (part 3)/20210520_Blot1 10min.tif]

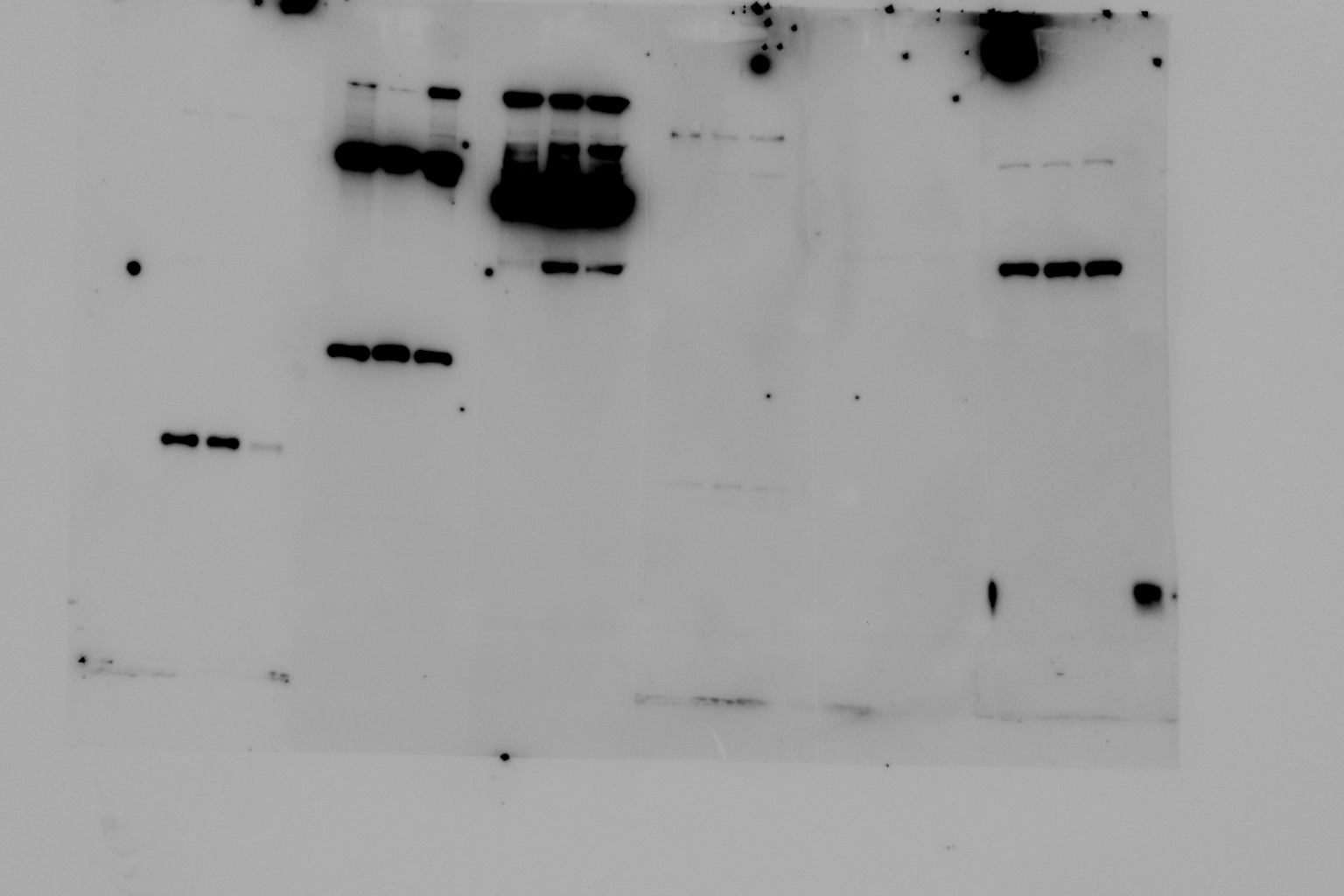

Supplement: Figure 1—source data 6. [file elife-108672-fig1-data6.zip › Fig 1B (part 3)/20210520_Blot1 10min_Nup214.tif]

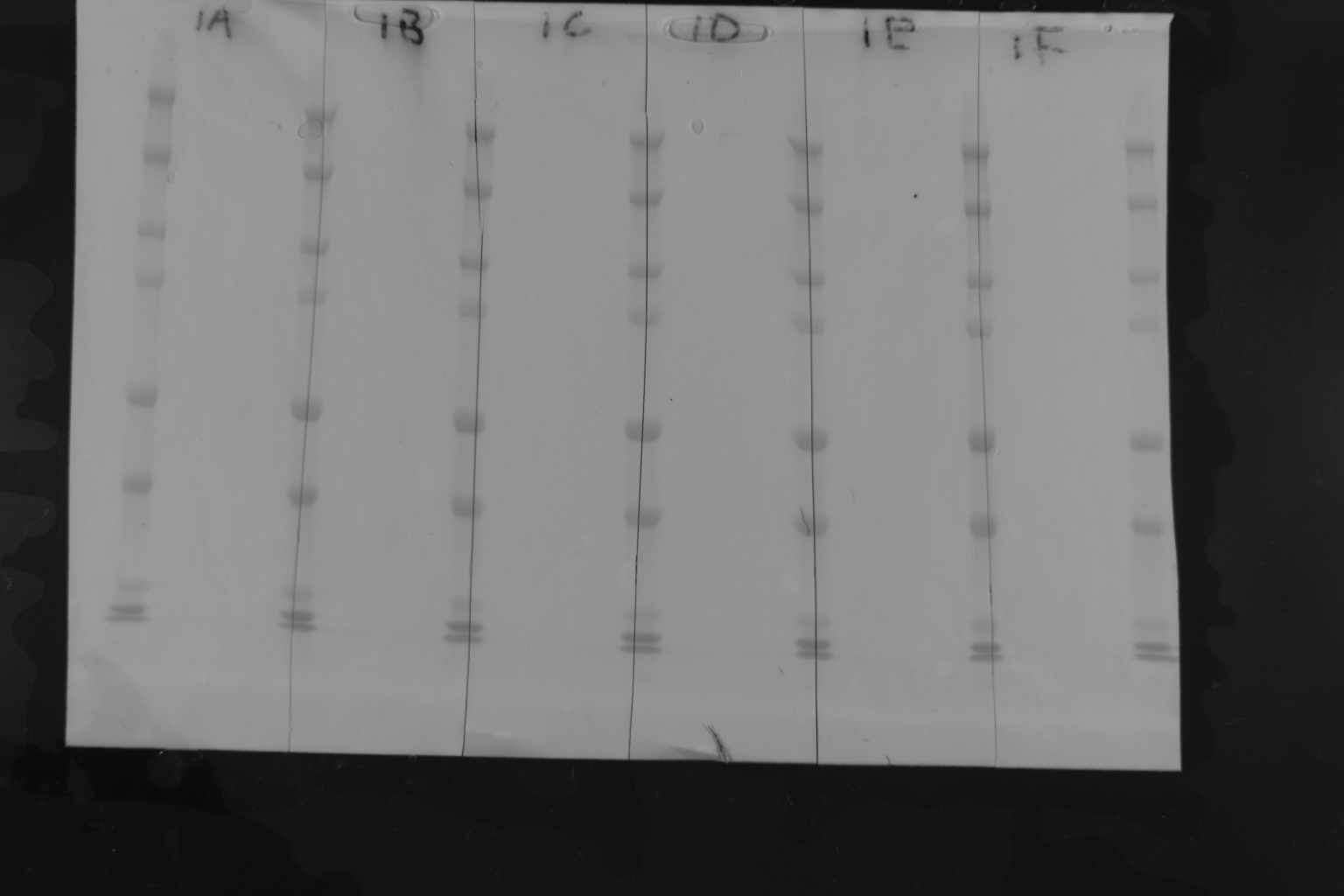

Supplement: Figure 1—source data 6. [file elife-108672-fig1-data6.zip › Fig 1B (part 3)/20210520_Blot1 ladder.tif]

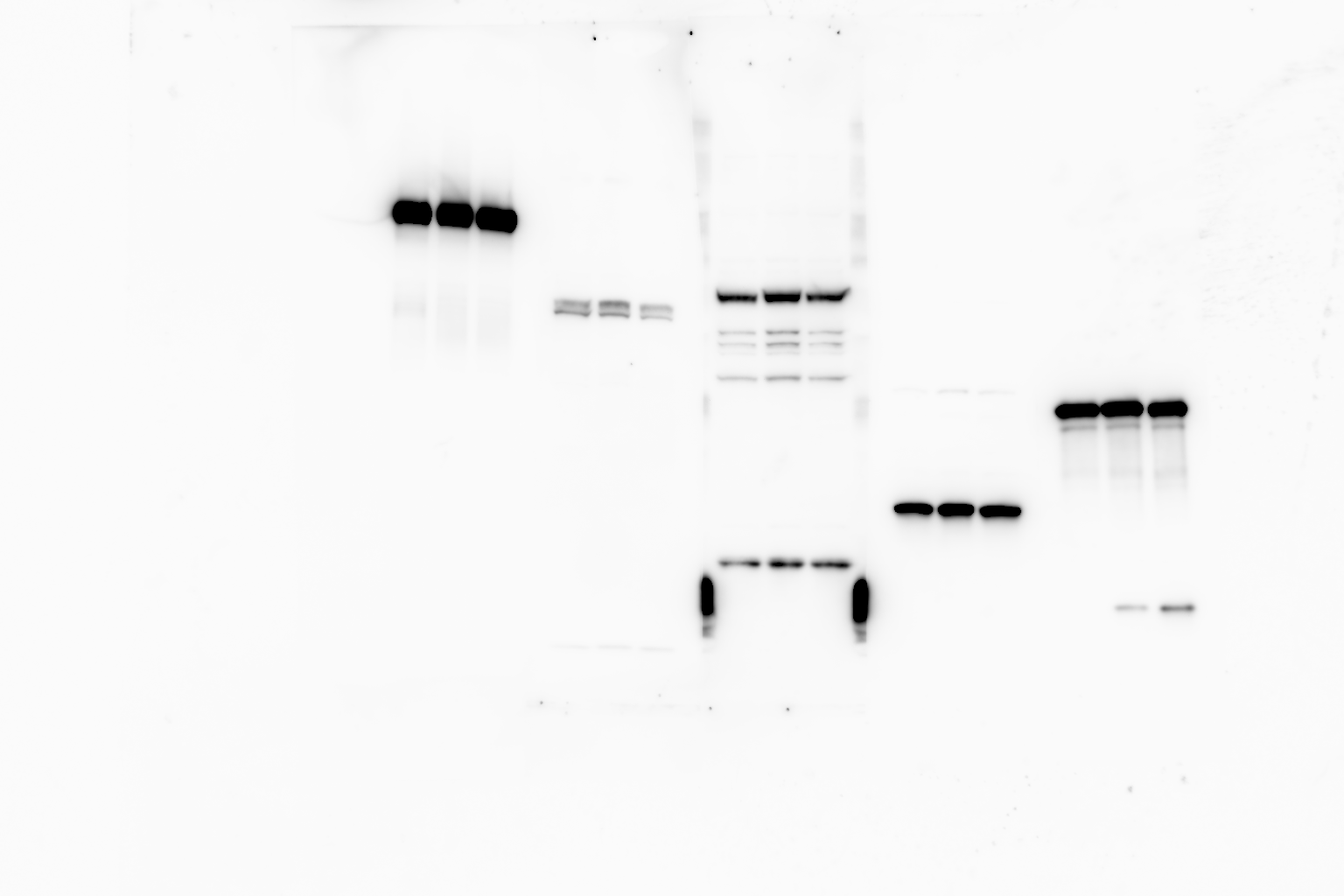

Supplement: Figure 1—source data 6. [file elife-108672-fig1-data6.zip › Fig 1B (part 3)/20210521_overblot1 10min.tif]

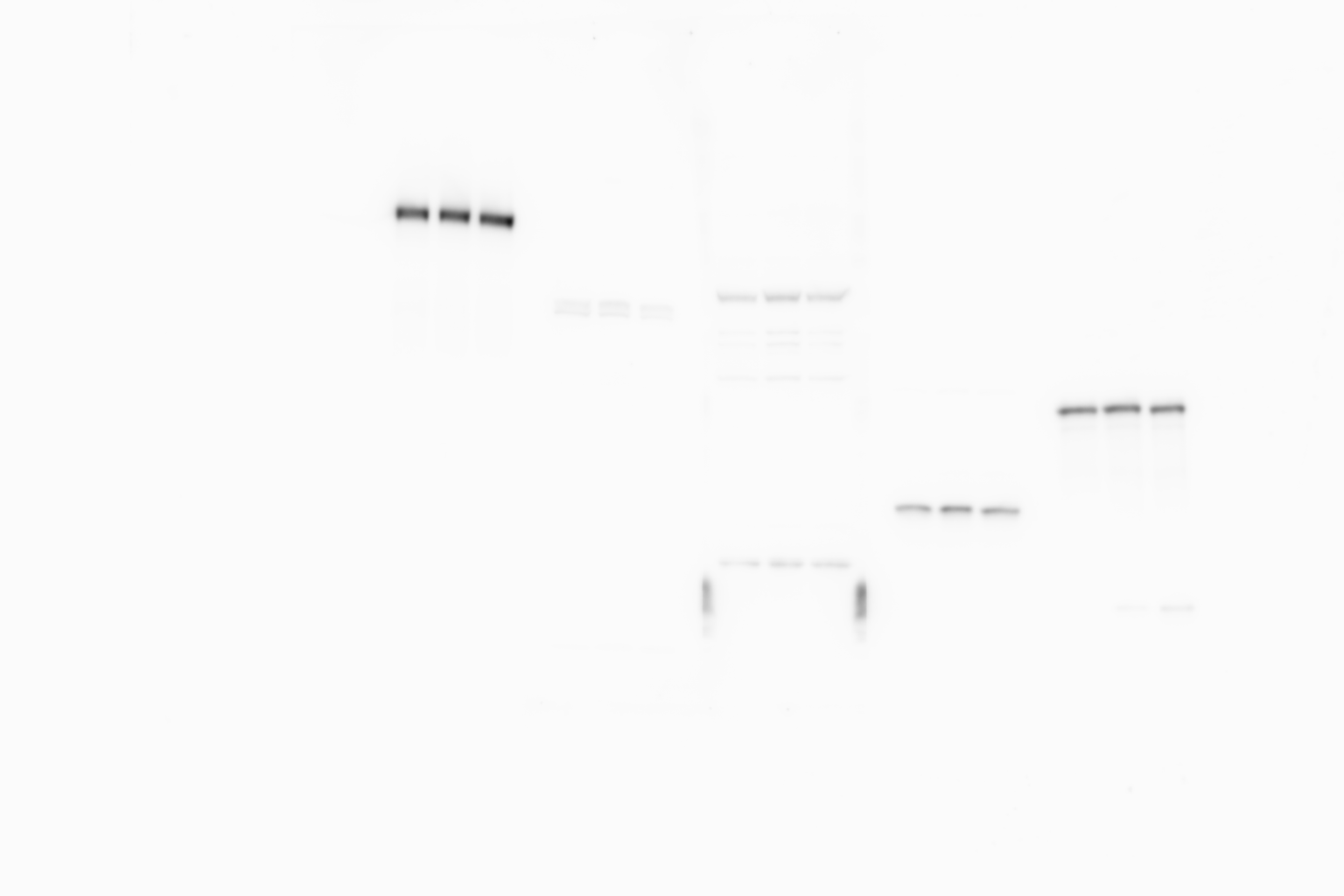

Supplement: Figure 1—source data 6. [file elife-108672-fig1-data6.zip › Fig 1B (part 3)/20210521_overblot1 1min.tif]

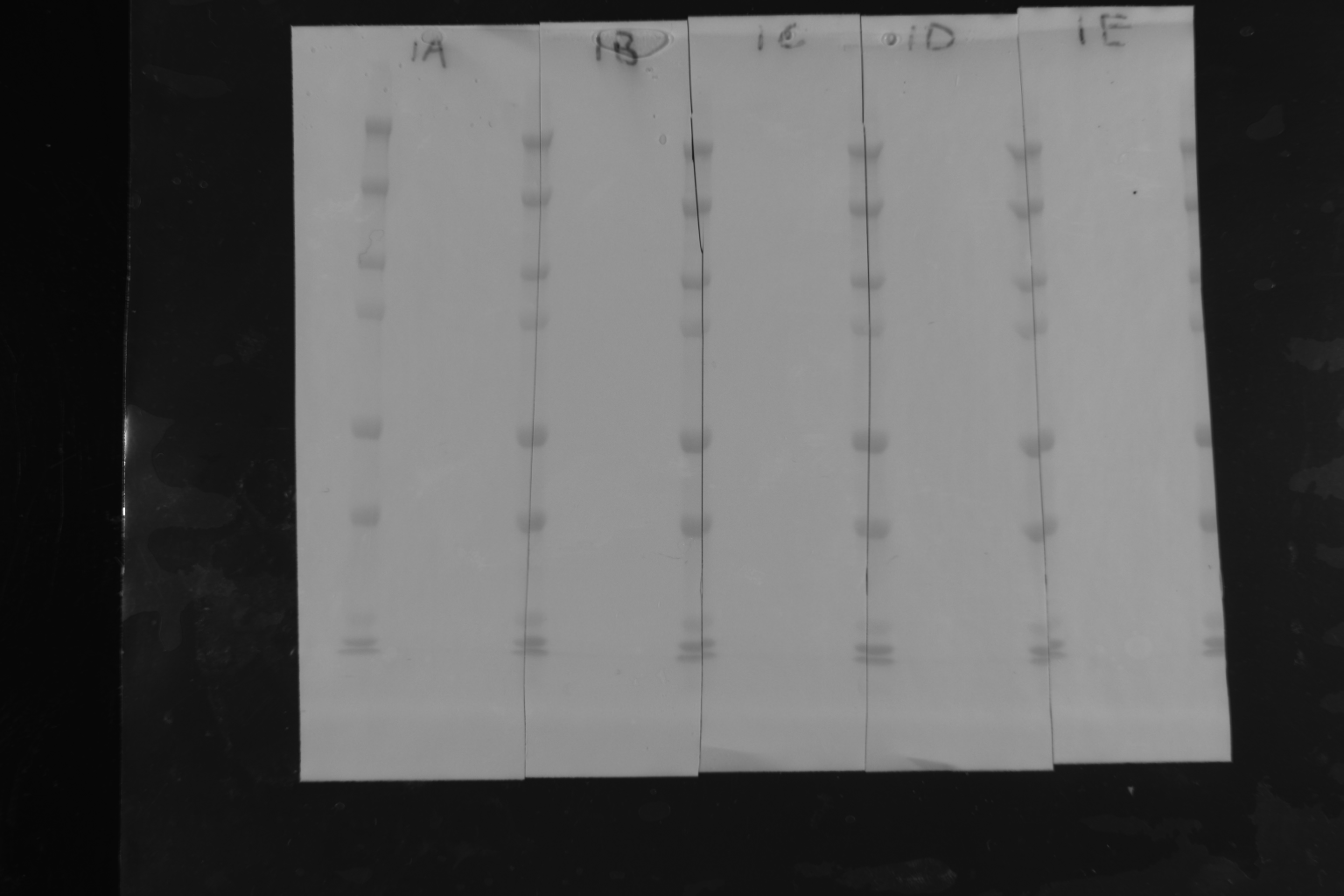

Supplement: Figure 1—source data 6. [file elife-108672-fig1-data6.zip › Fig 1B (part 3)/20210521_overblot1 ladder.tif]

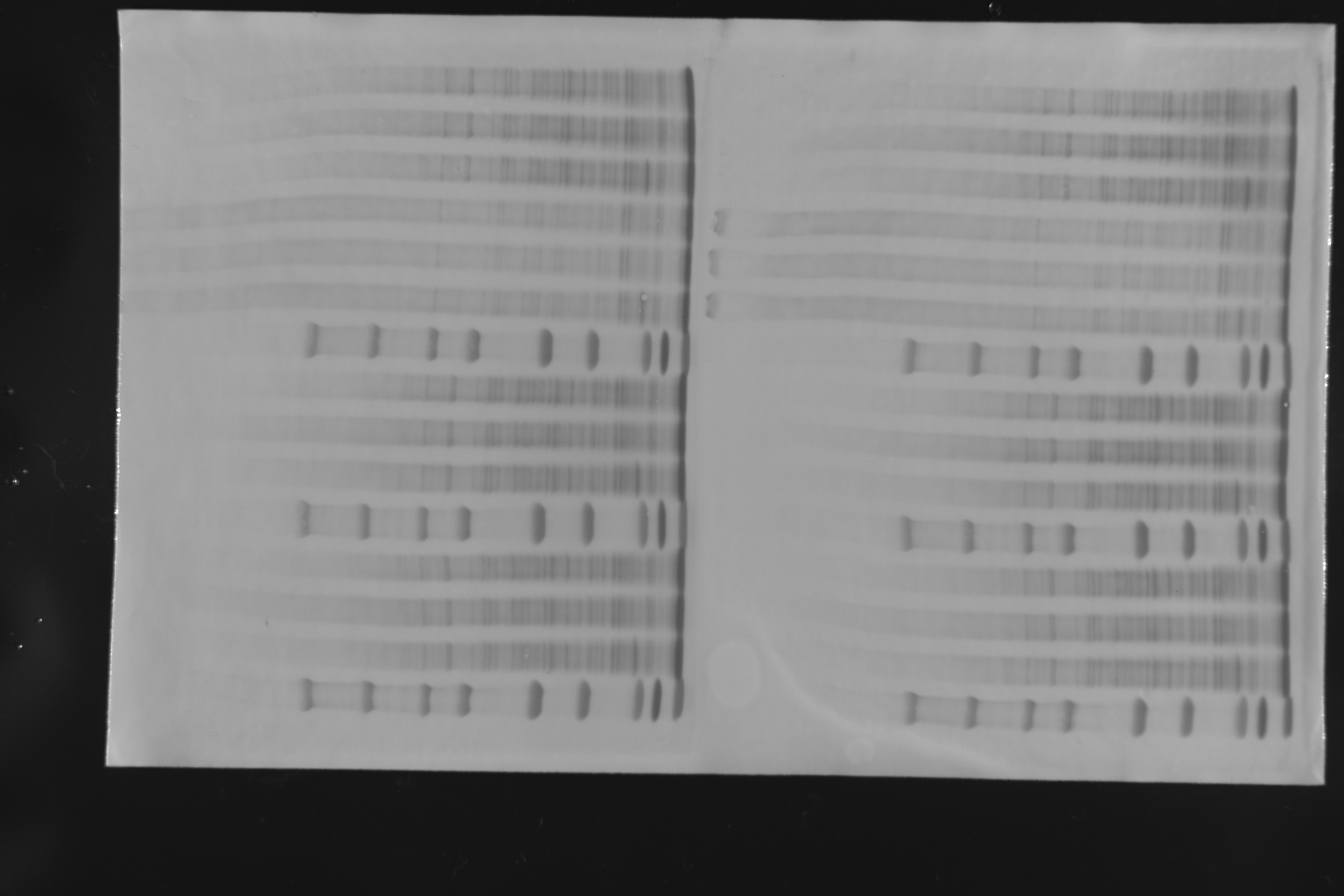

Supplement: Figure 1—source data 6. [file elife-108672-fig1-data6.zip › Fig 1B (part 3)/20210601_Blot1 and 2, Ponceau s.tif]

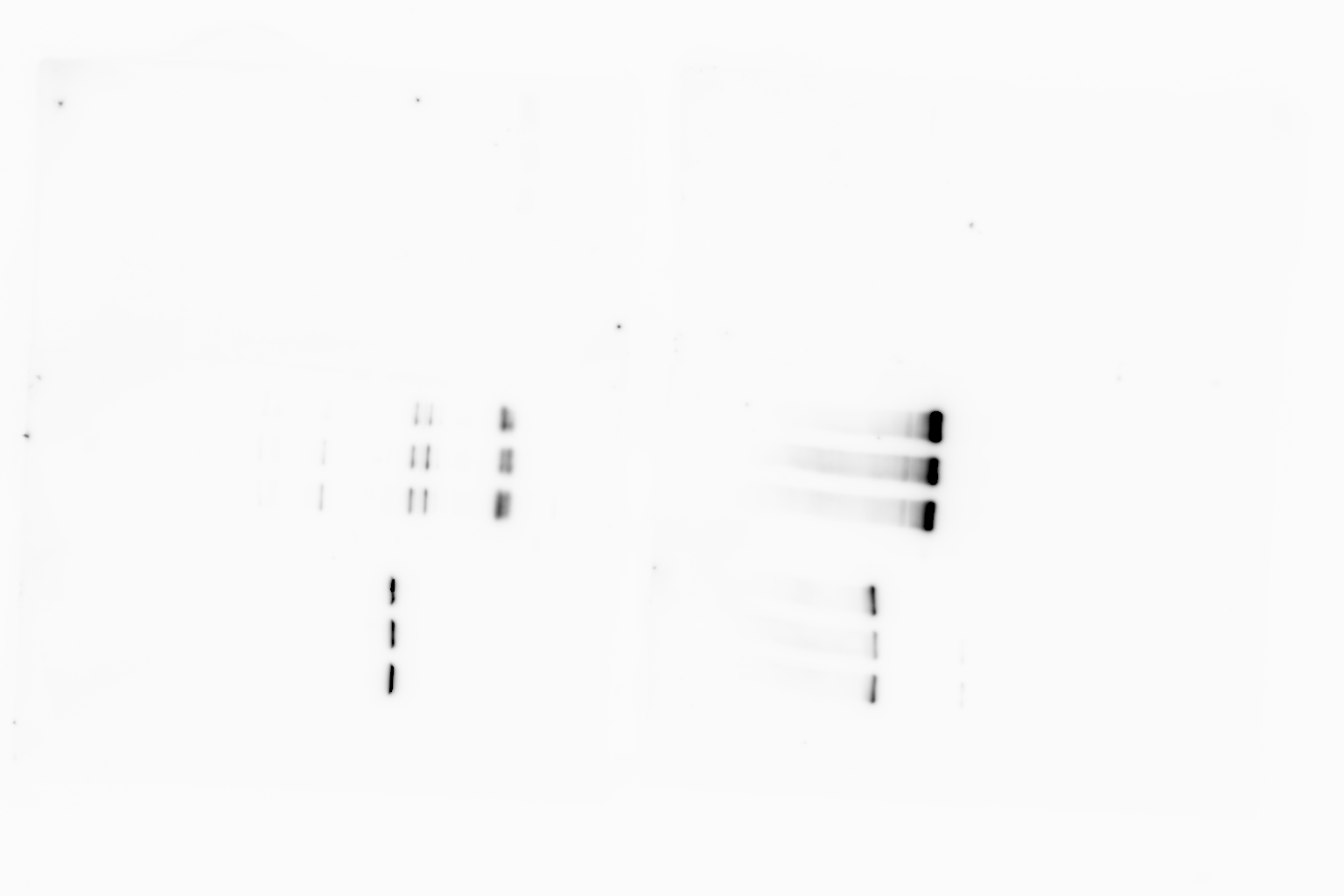

Supplement: Figure 1—source data 6. [file elife-108672-fig1-data6.zip › Fig 1B (part 3)/20210602_Blot1and 2 10min.tif]

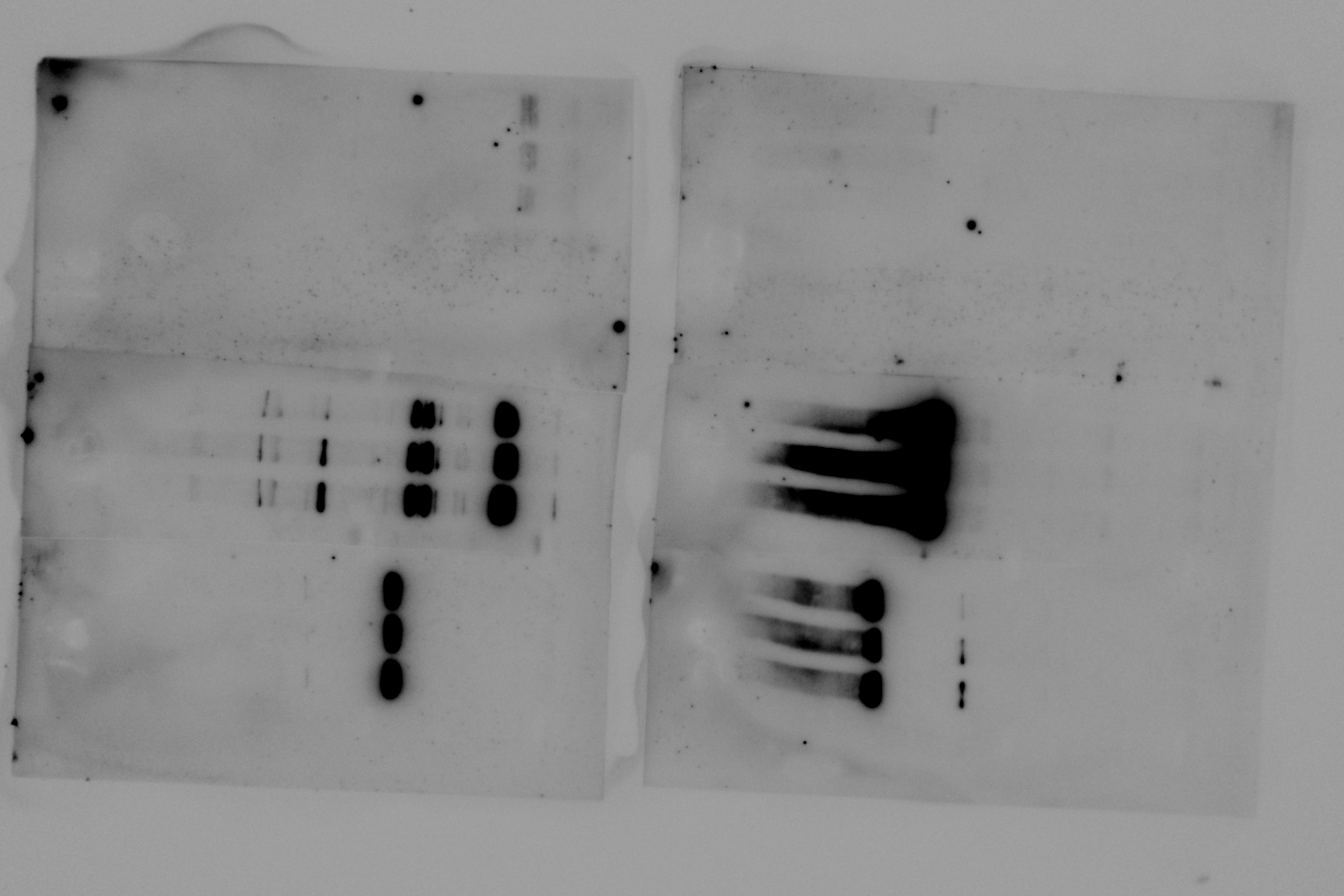

Supplement: Figure 1—source data 6. [file elife-108672-fig1-data6.zip › Fig 1B (part 3)/20210602_Blot1and 2 10min_Nup37.tif]

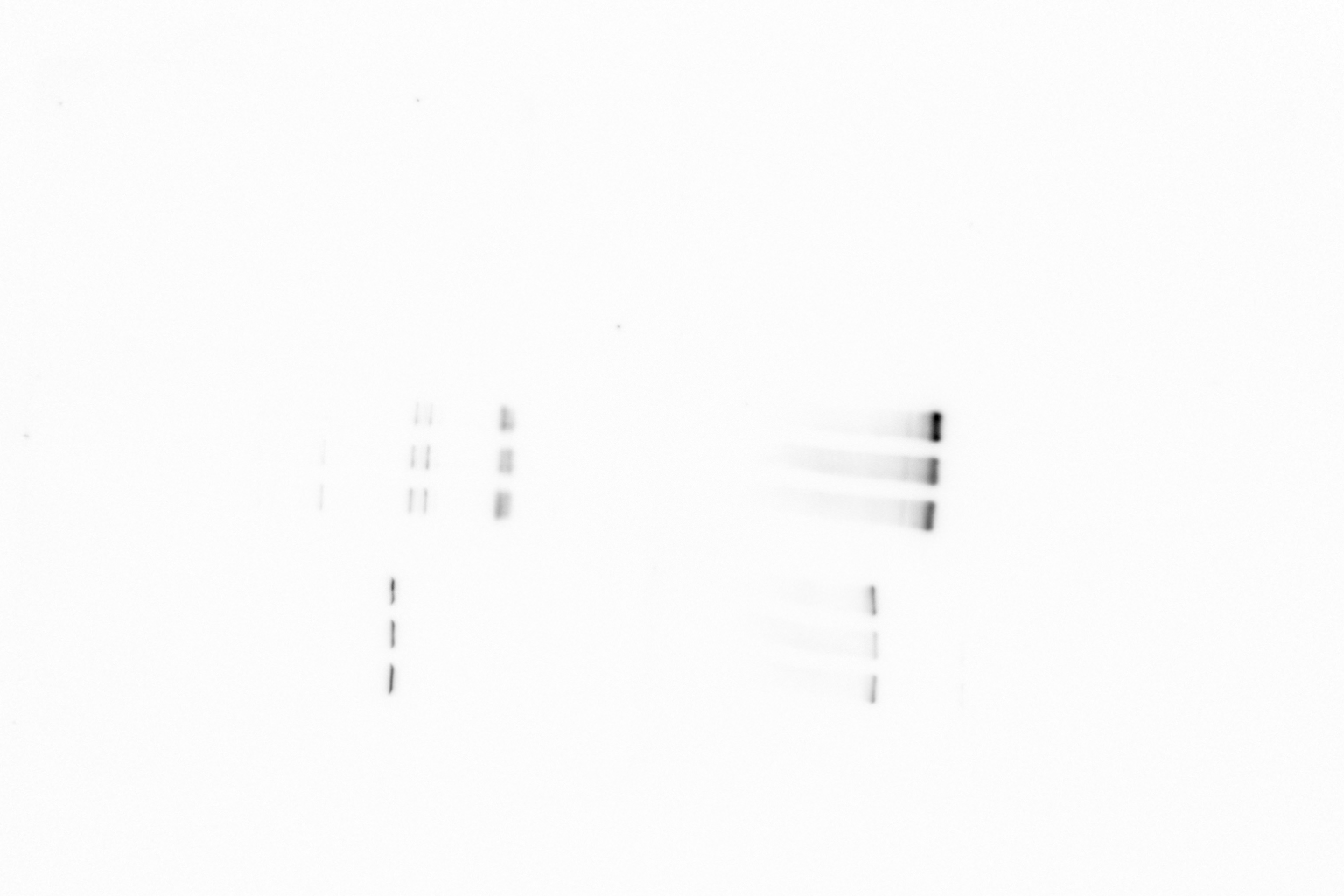

Supplement: Figure 1—source data 6. [file elife-108672-fig1-data6.zip › Fig 1B (part 3)/20210602_Blot1and 2 15sec.tif]

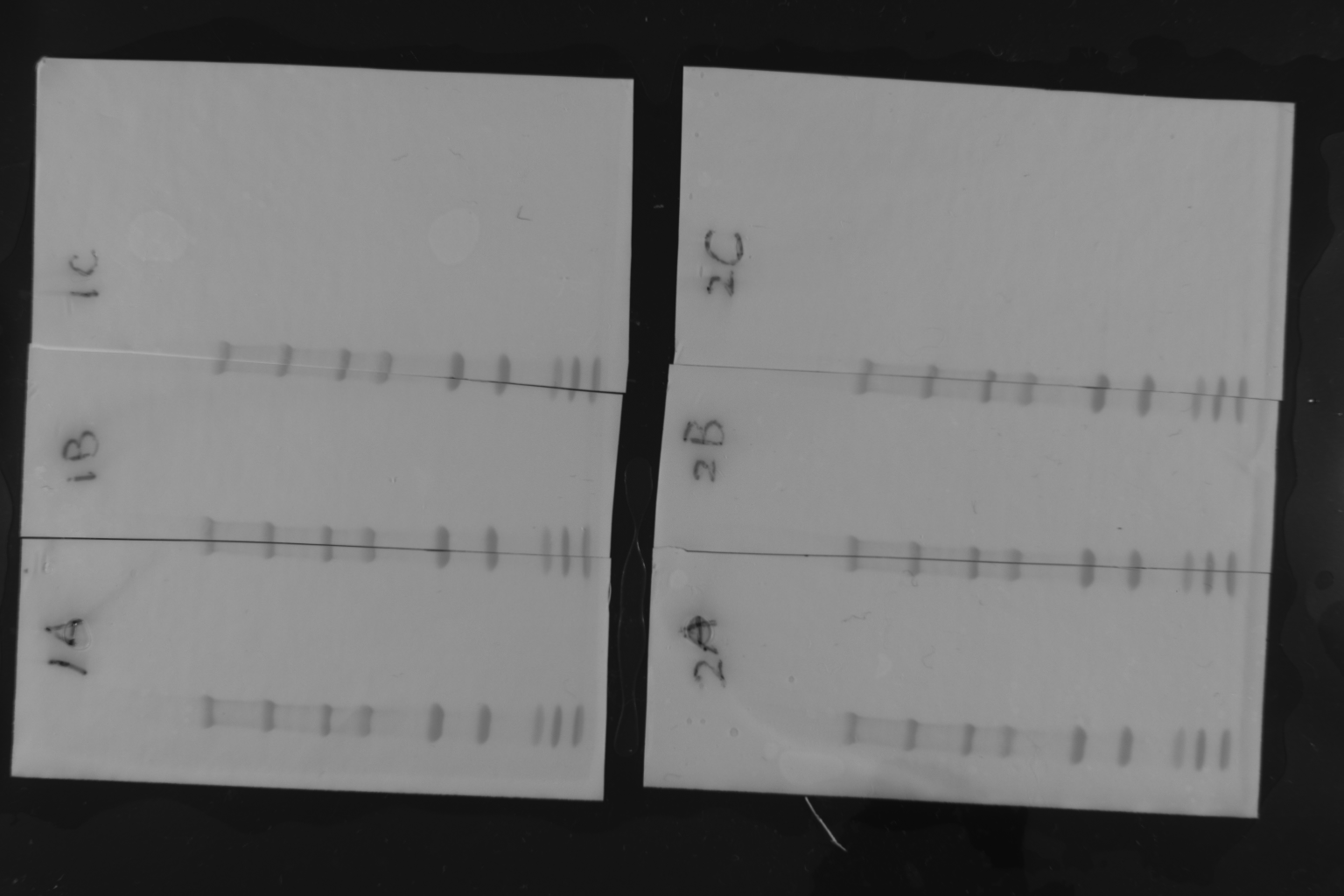

Supplement: Figure 1—source data 6. [file elife-108672-fig1-data6.zip › Fig 1B (part 3)/20210602_Blot1and 2 ladder.tif]

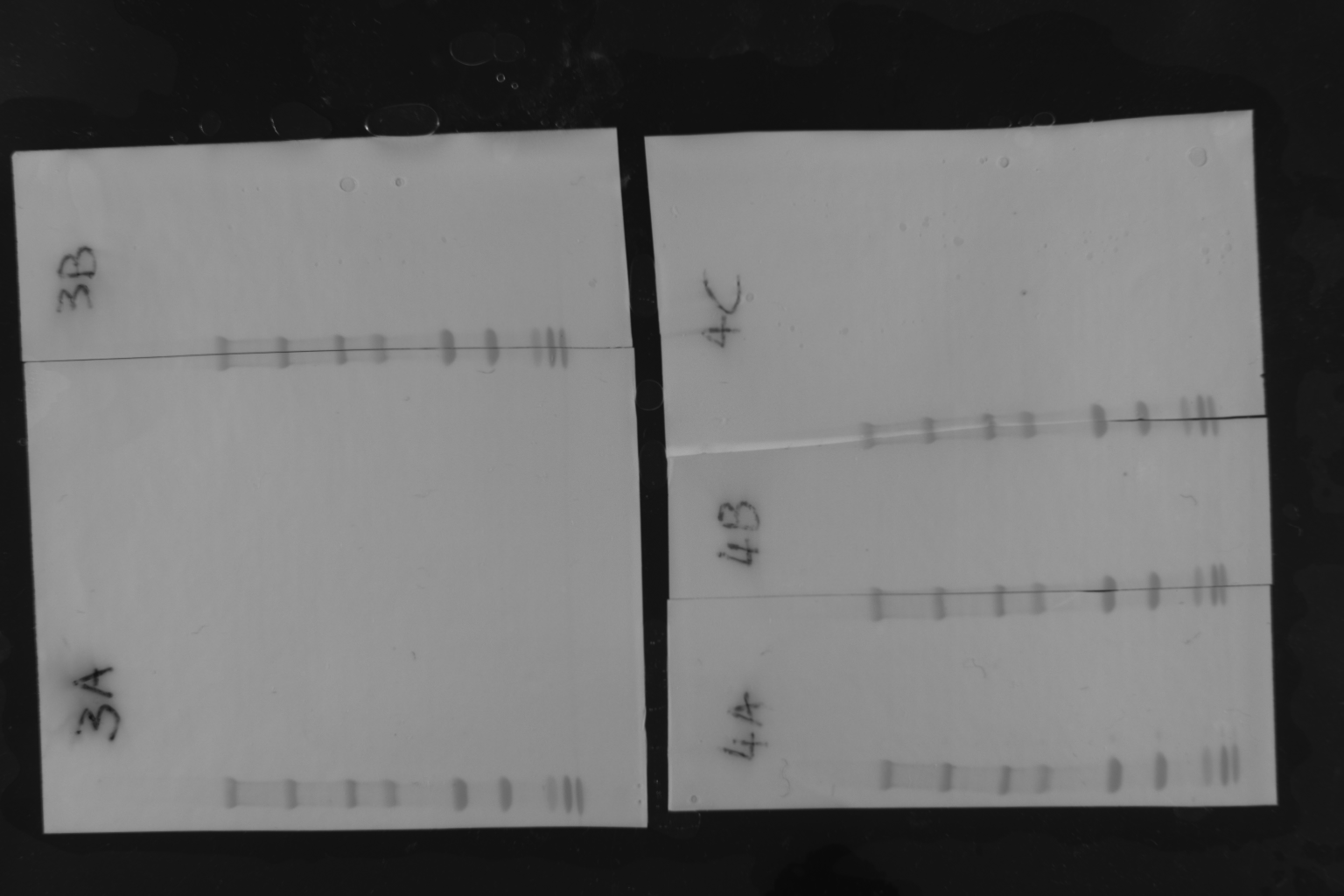

Supplement: Figure 1—source data 6. [file elife-108672-fig1-data6.zip › Fig 1B (part 3)/20210604_Blot3and4 ladder.tif]

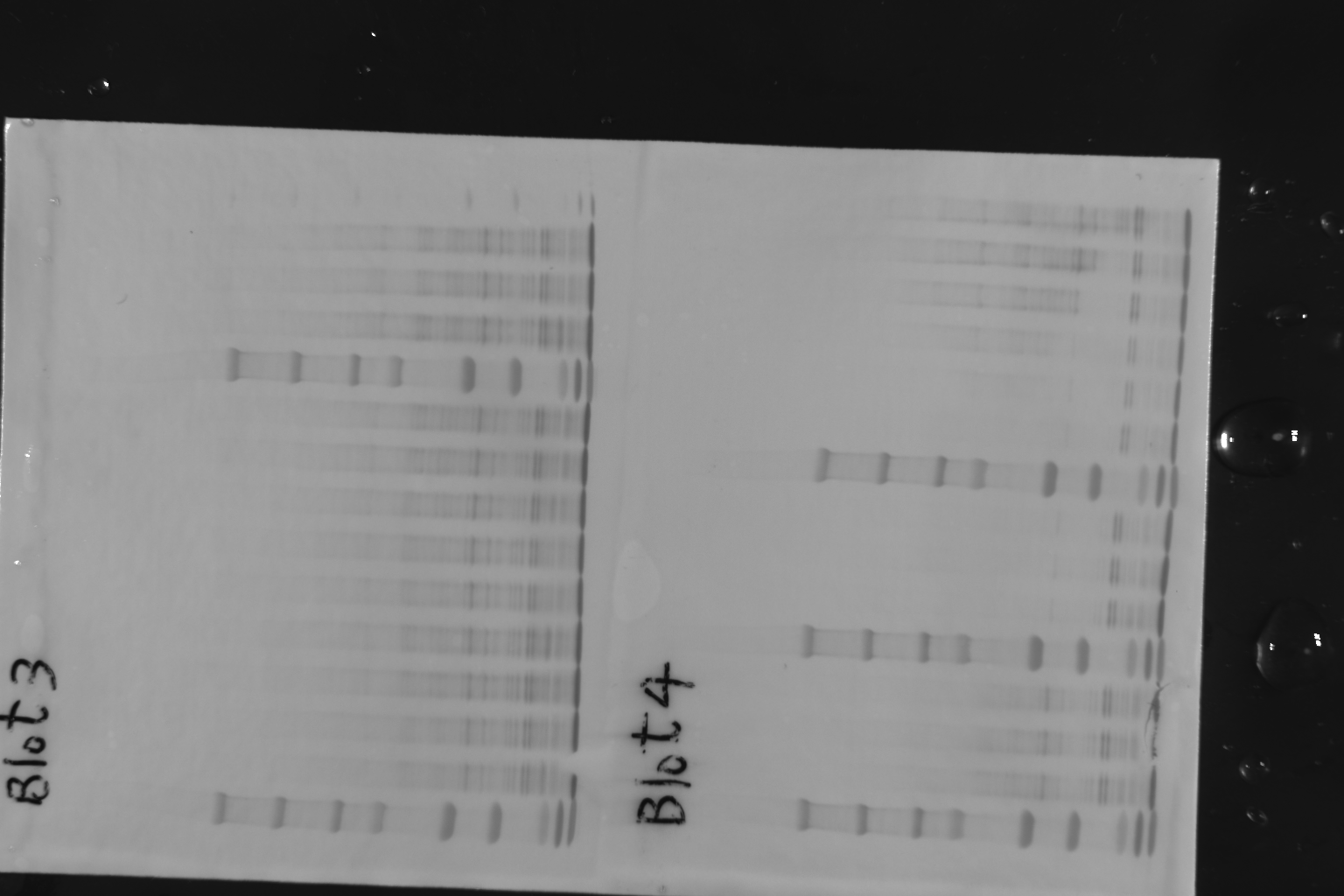

Supplement: Figure 1—source data 6. [file elife-108672-fig1-data6.zip › Fig 1B (part 3)/20210610_Blot3,4 Ponceau S.tif]

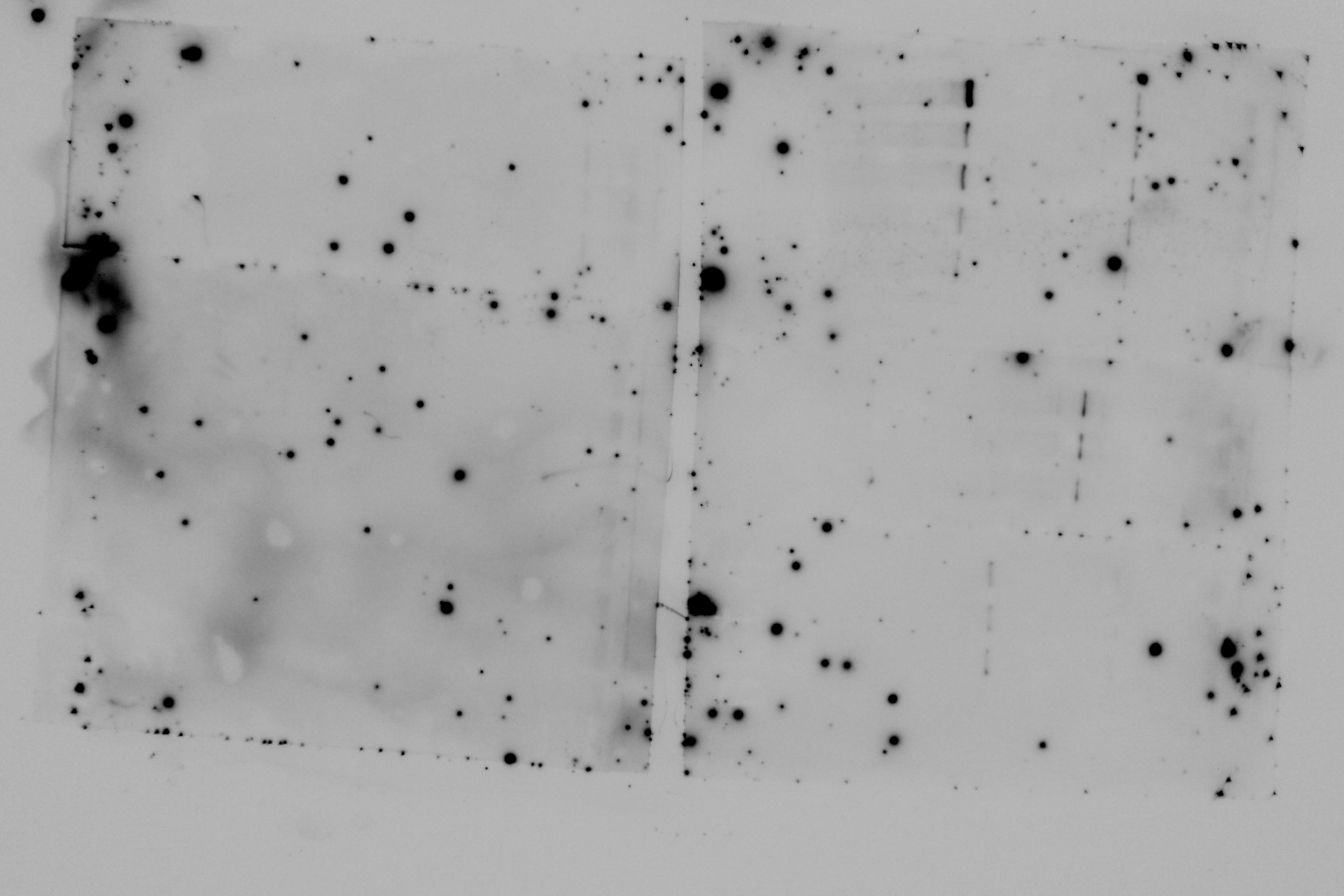

Supplement: Figure 1—source data 6. [file elife-108672-fig1-data6.zip › Fig 1B (part 3)/20210611_Blot3,4 10min_Nup96.tif]

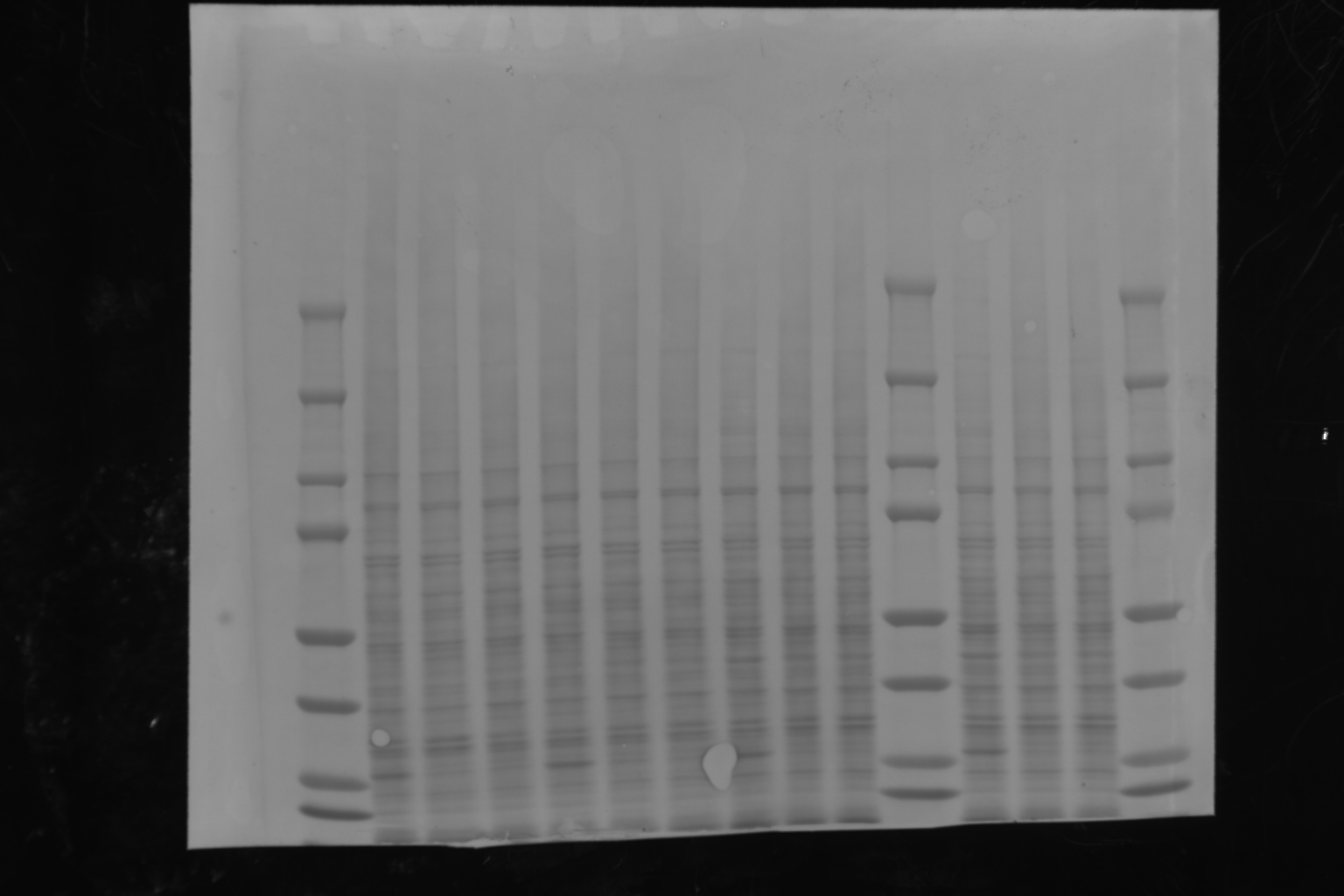

Supplement: Figure 1—source data 6. [file elife-108672-fig1-data6.zip › Fig 1B (part 3)/20210729_1_Ponceau.tif]

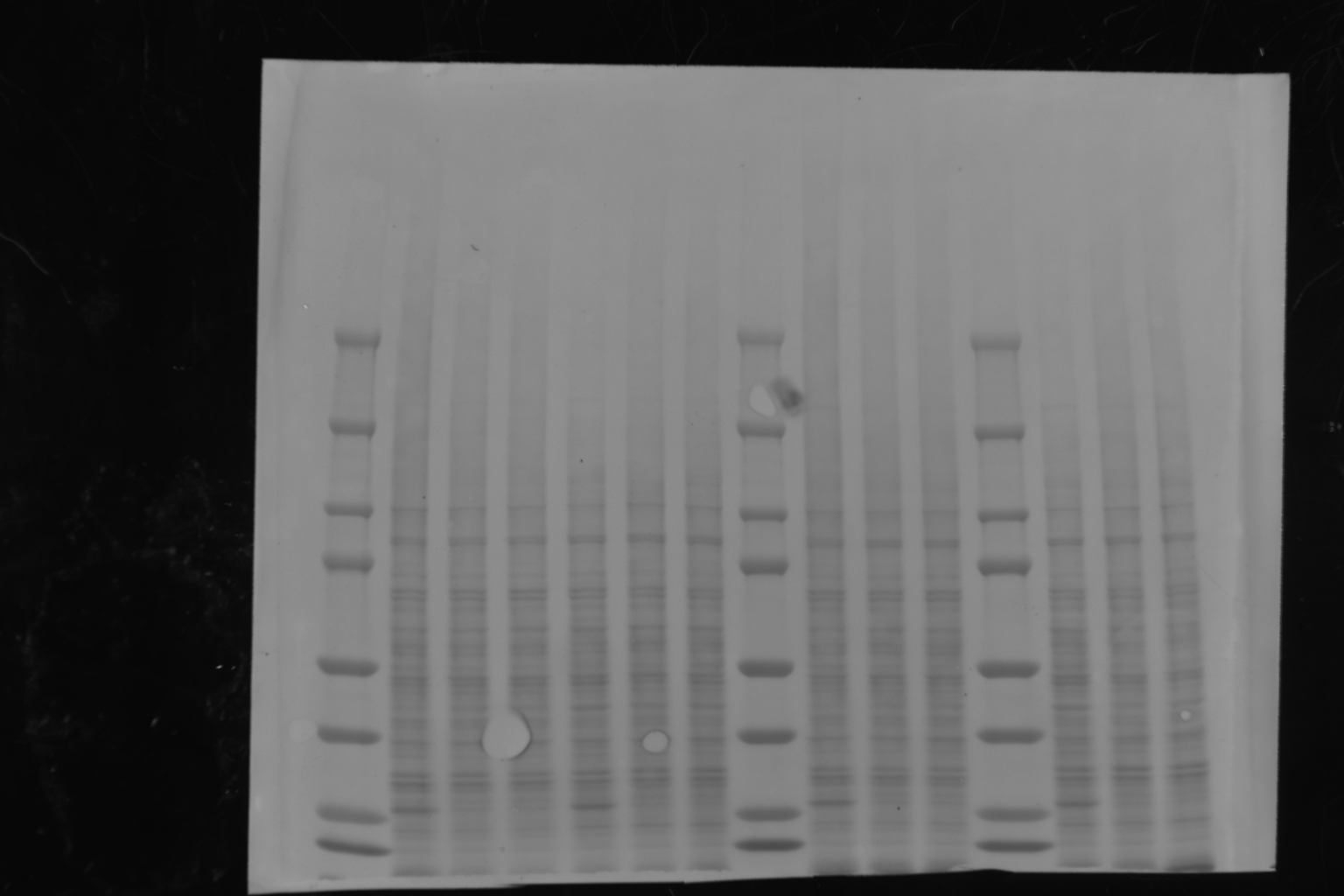

Supplement: Figure 1—source data 6. [file elife-108672-fig1-data6.zip › Fig 1B (part 3)/20210729_2_Ponceau.tif]

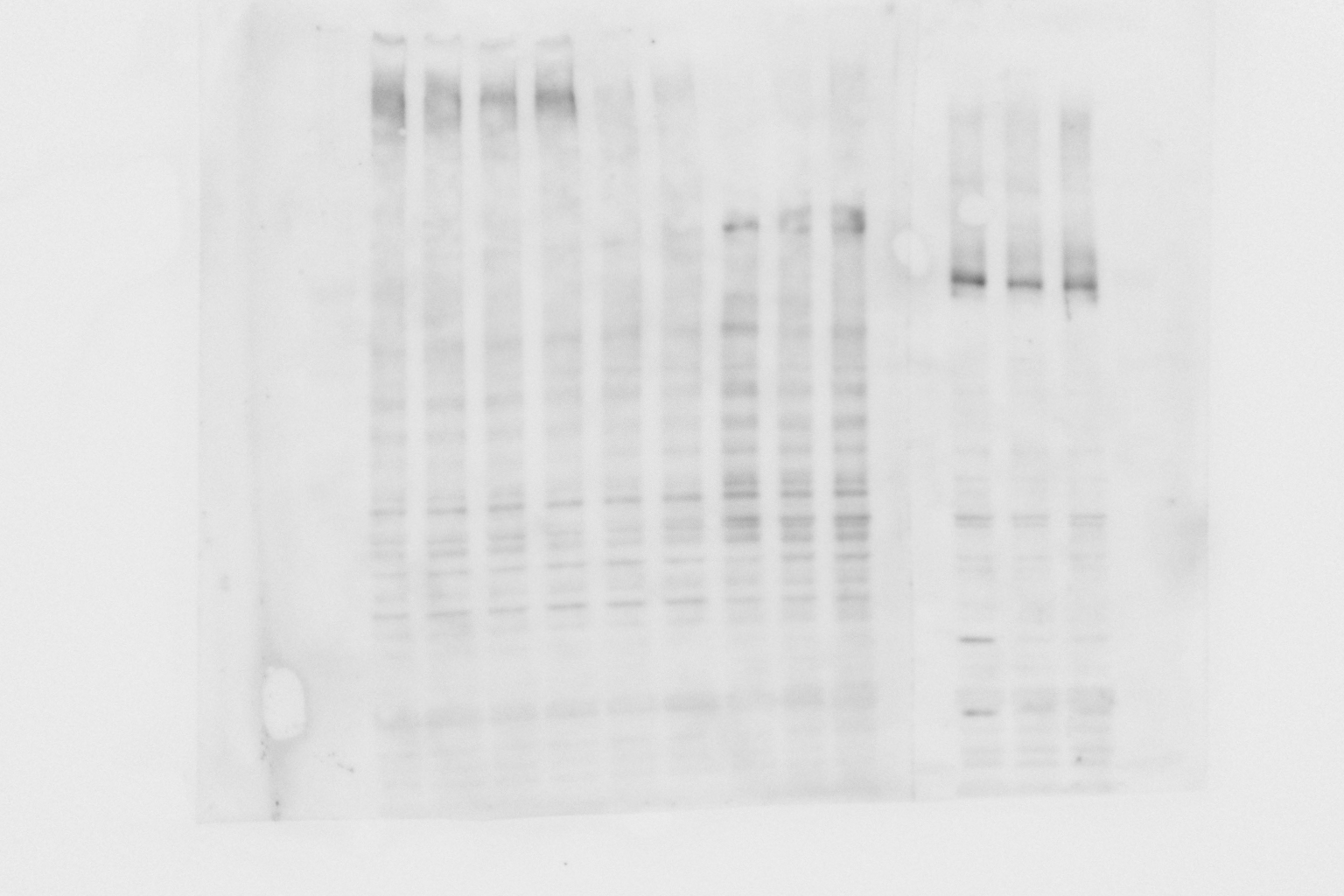

Supplement: Figure 1—source data 6. [file elife-108672-fig1-data6.zip › Fig 1B (part 3)/20210730_1_15sec2_GP210.tif]

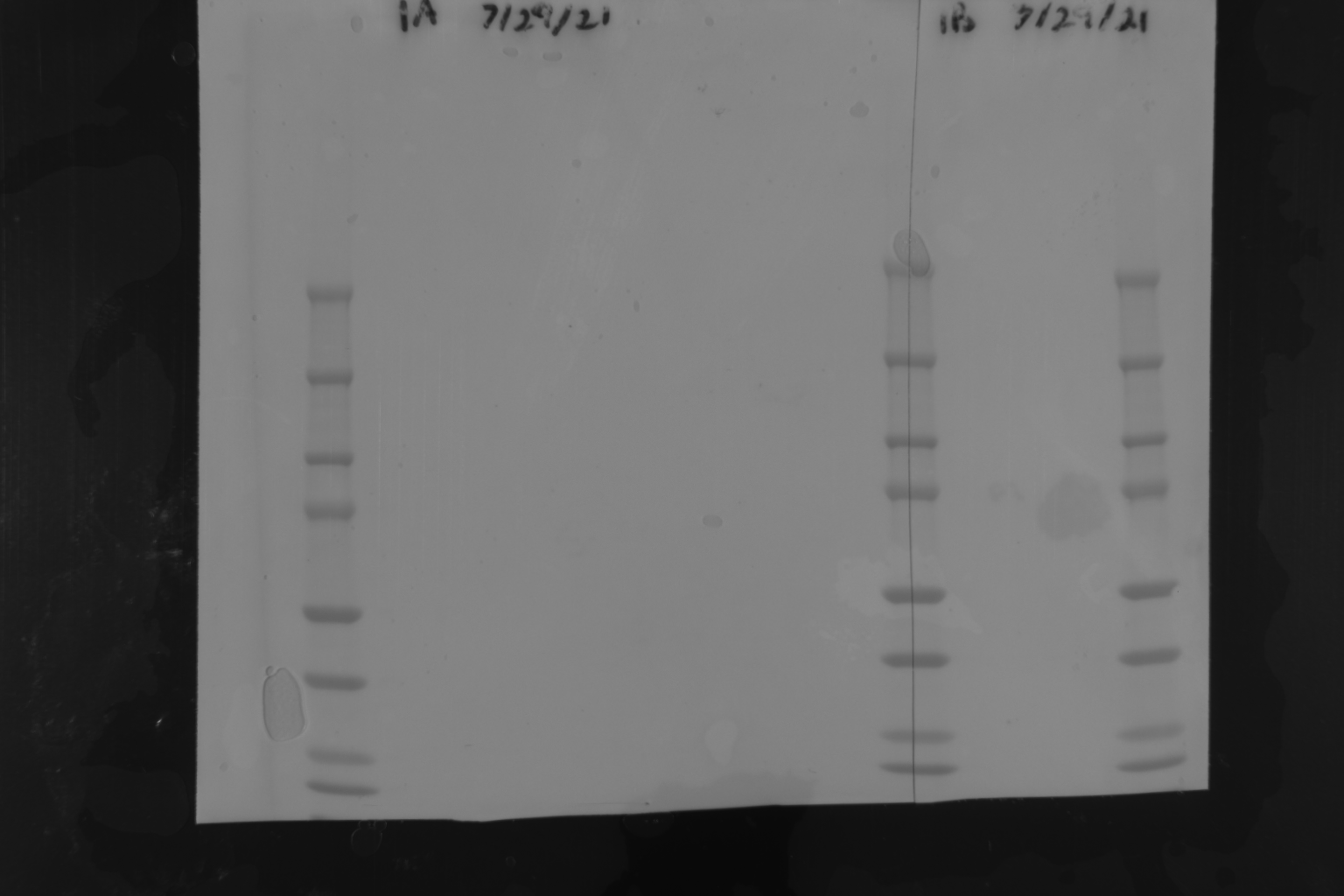

Supplement: Figure 1—source data 6. [file elife-108672-fig1-data6.zip › Fig 1B (part 3)/20210730_1_ladder.tif]

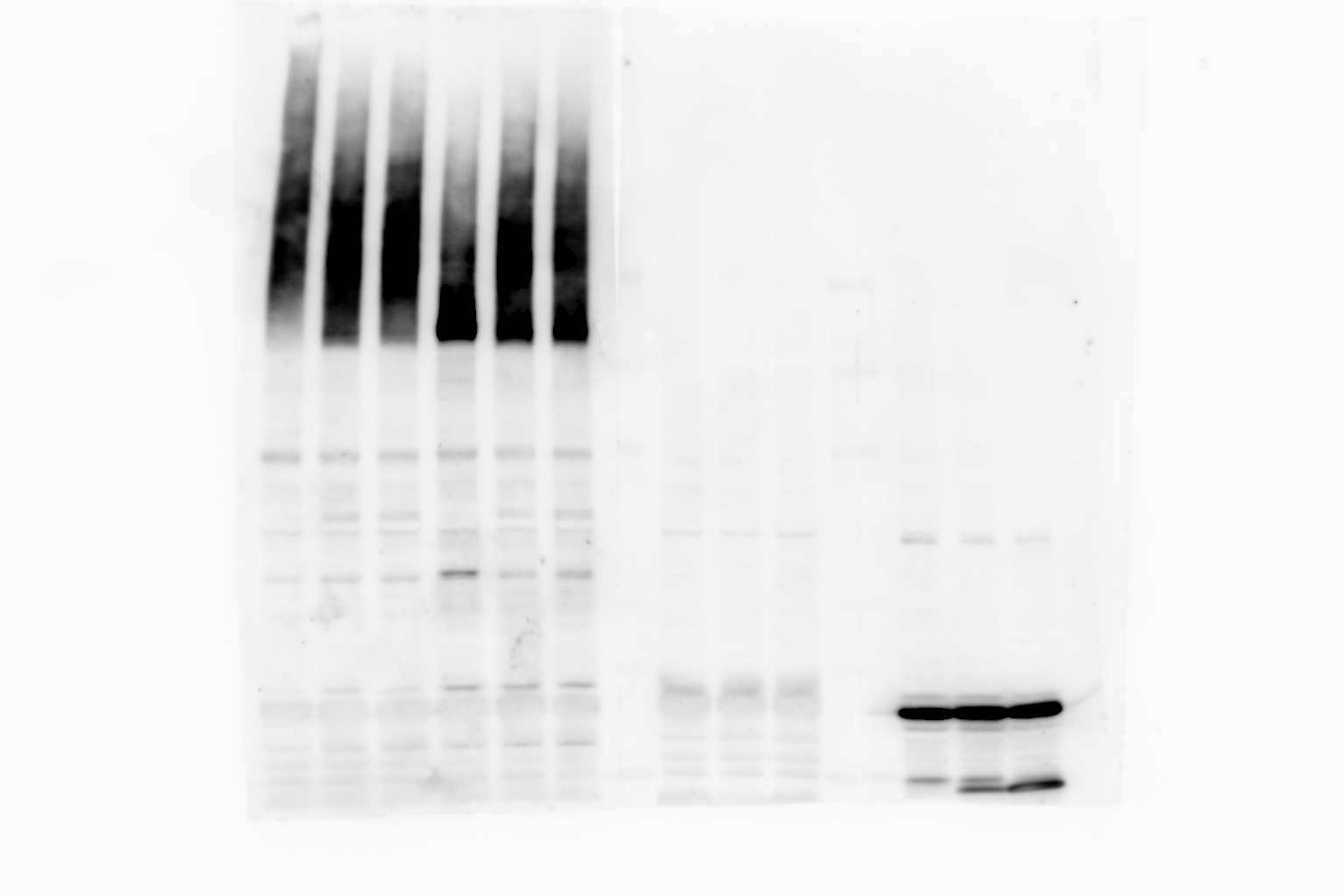

Supplement: Figure 1—source data 6. [file elife-108672-fig1-data6.zip › Fig 1B (part 3)/20210730_2_10min.tif]

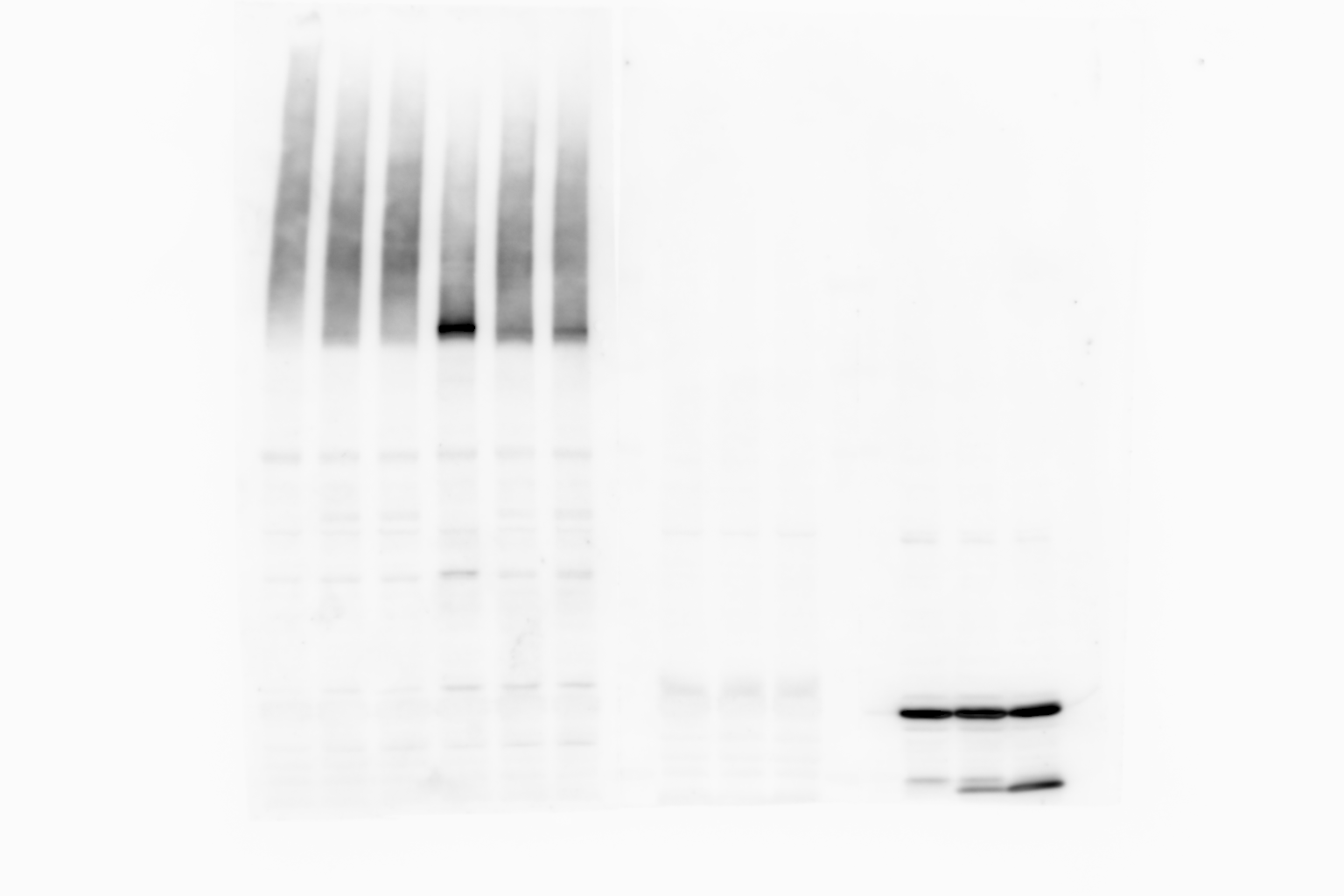

Supplement: Figure 1—source data 6. [file elife-108672-fig1-data6.zip › Fig 1B (part 3)/20210730_2_2min.tif]

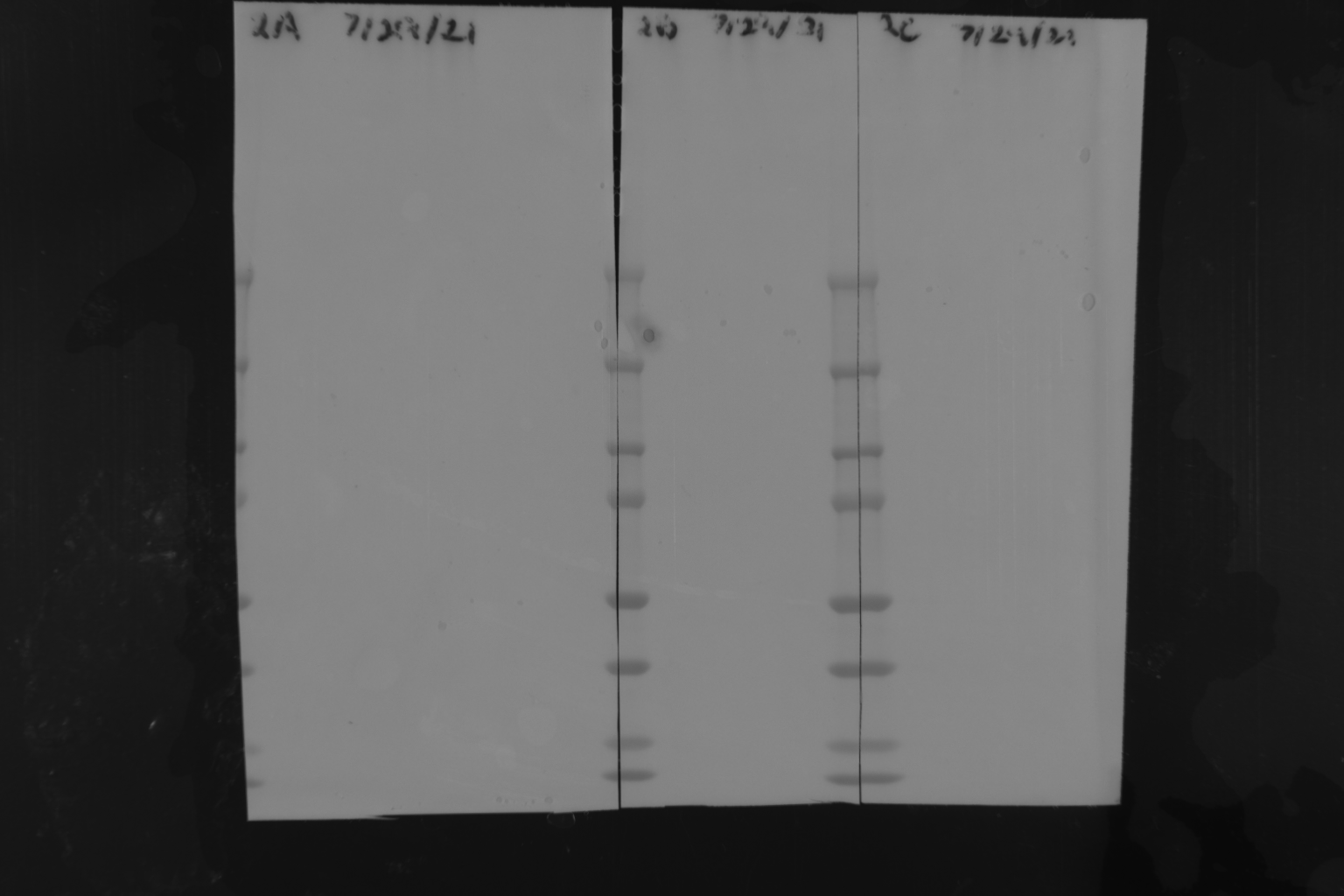

Supplement: Figure 1—source data 6. [file elife-108672-fig1-data6.zip › Fig 1B (part 3)/20210730_2_ladder.tif]

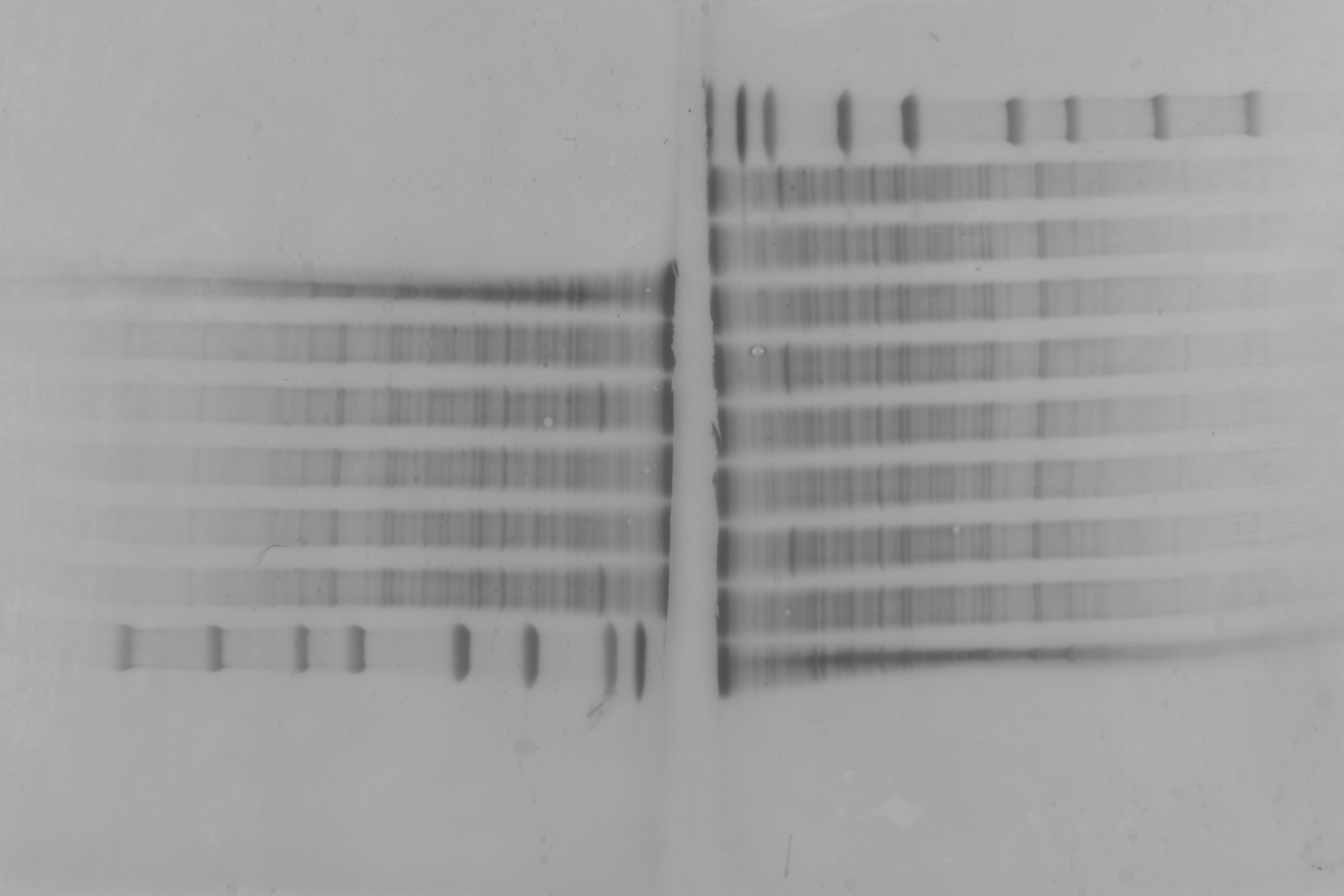

Supplement: Figure 1—source data 6. [file elife-108672-fig1-data6.zip › Fig 1B (part 3)/20210810_Ponceau.tif]

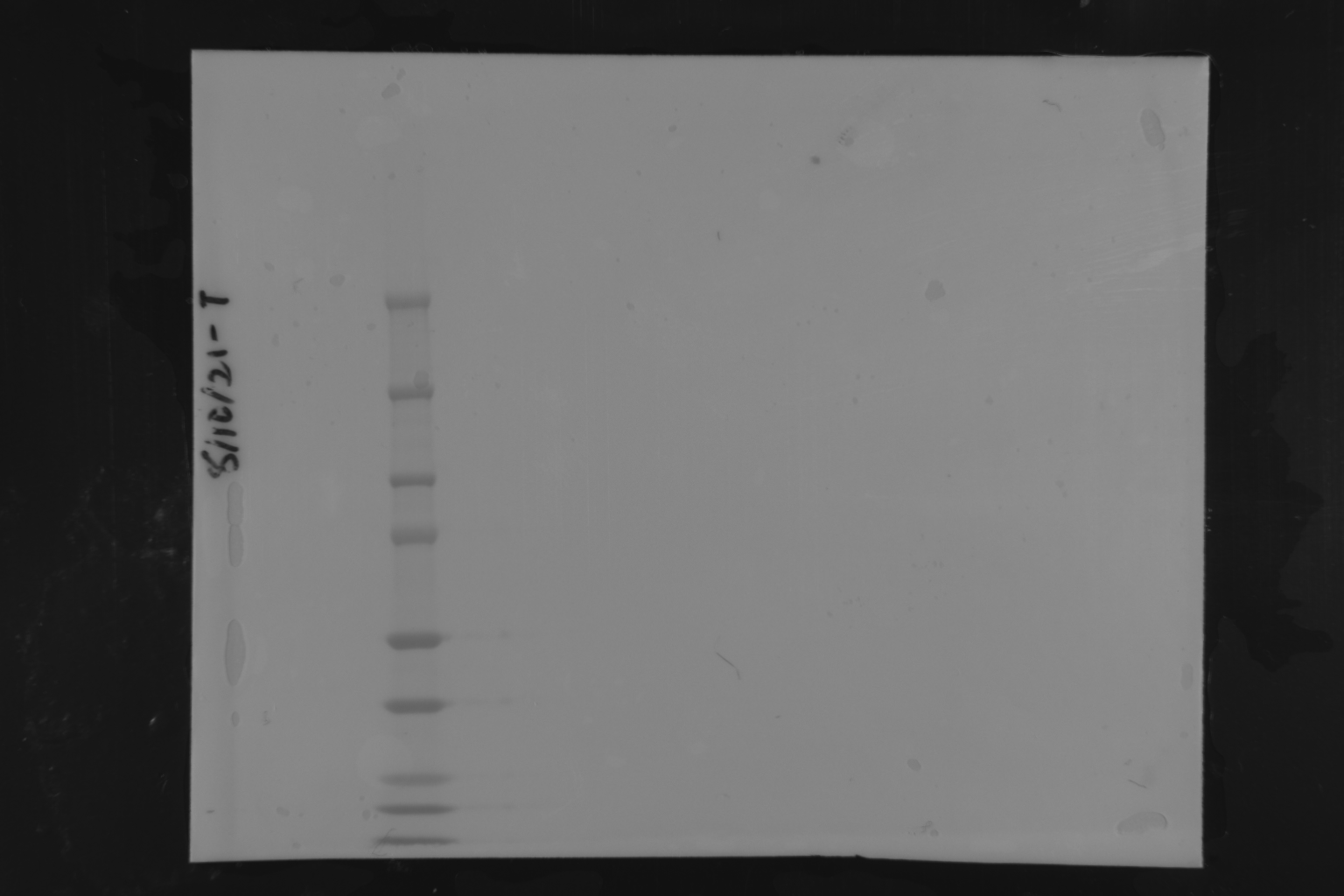

Supplement: Figure 1—source data 6. [file elife-108672-fig1-data6.zip › Fig 1B (part 3)/20210811_1_ladder.tif]

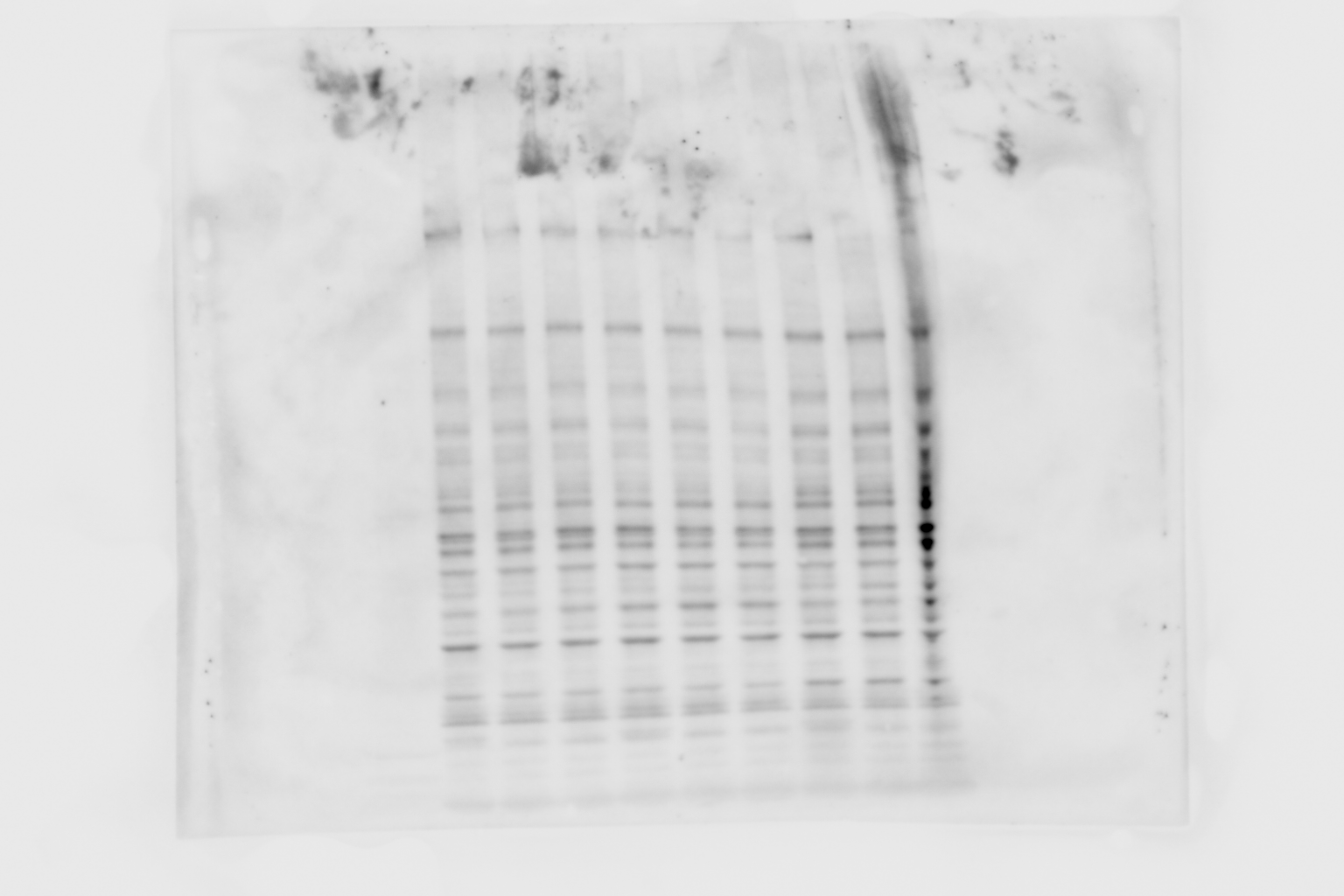

Supplement: Figure 1—source data 6. [file elife-108672-fig1-data6.zip › Fig 1B (part 3)/20210812_1repeat_1min_ELYS.tif]

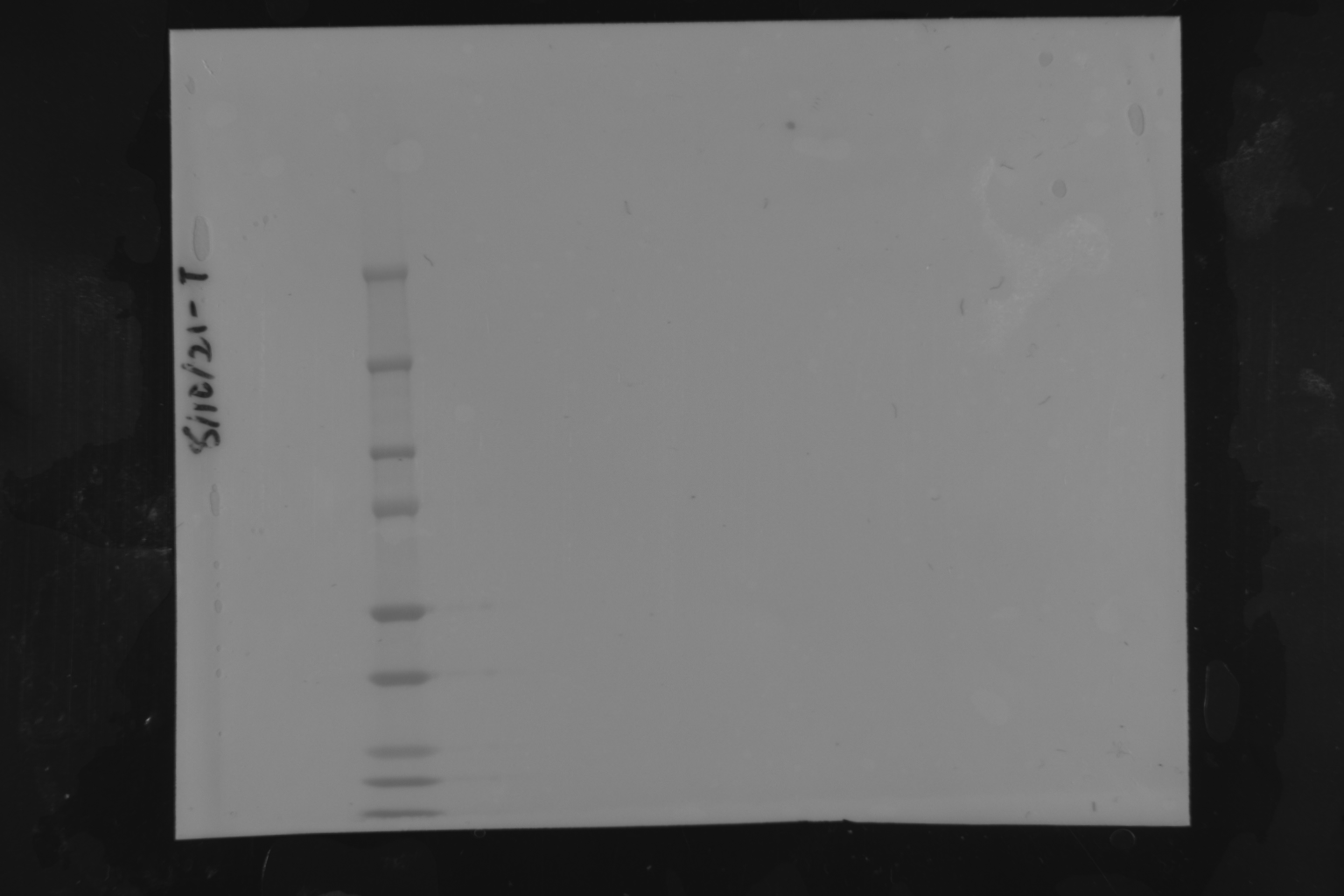

Supplement: Figure 1—source data 6. [file elife-108672-fig1-data6.zip › Fig 1B (part 3)/20210812_1repeat_ladder.tif]

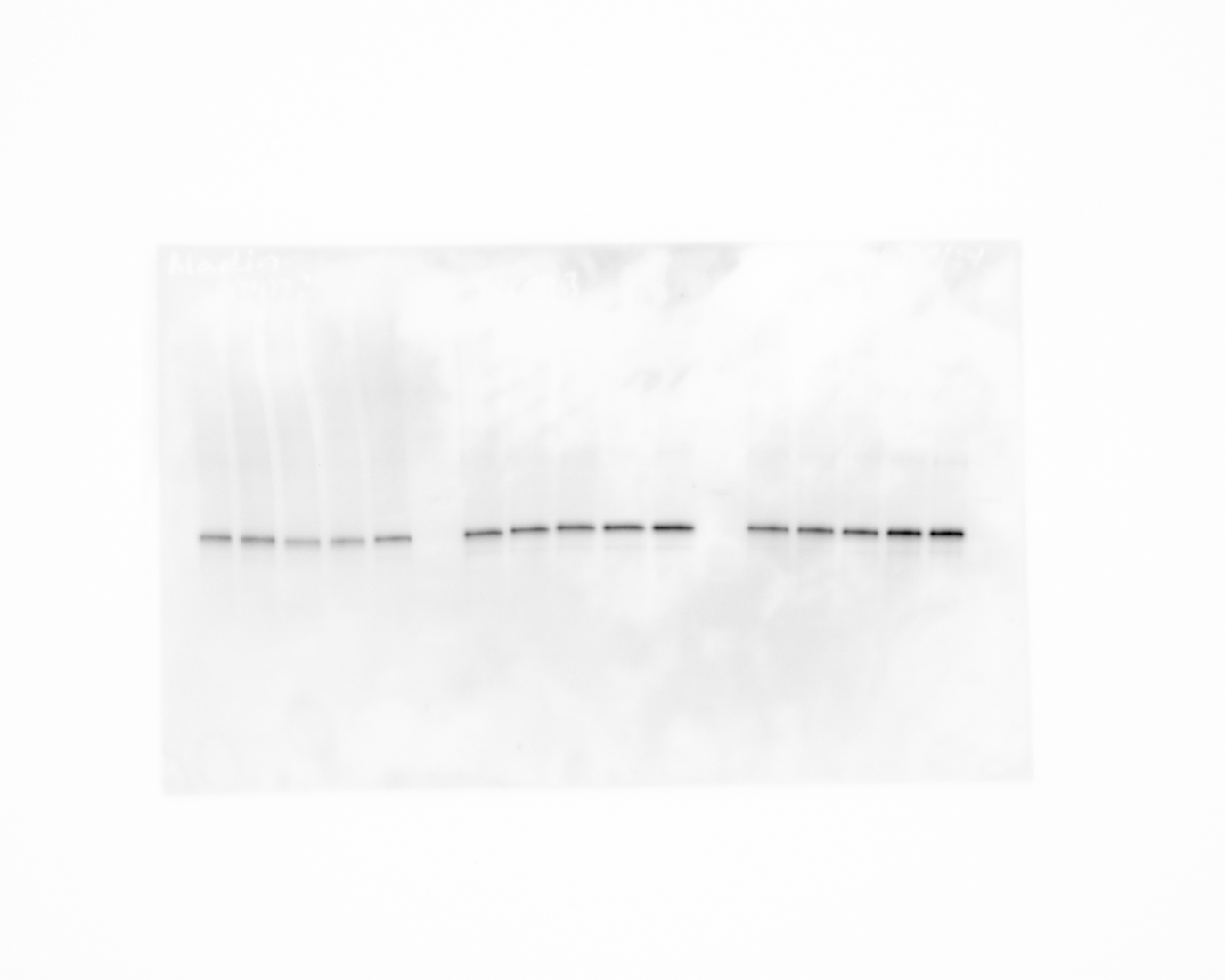

Supplement: Figure 1—source data 7. [file elife-108672-fig1-data7.zip › Fig 1C (part 1)/Aladin/Aladin_Chemi imaging_from StainFree 7DEC #1_2Apro_MM2_3_4.tif]

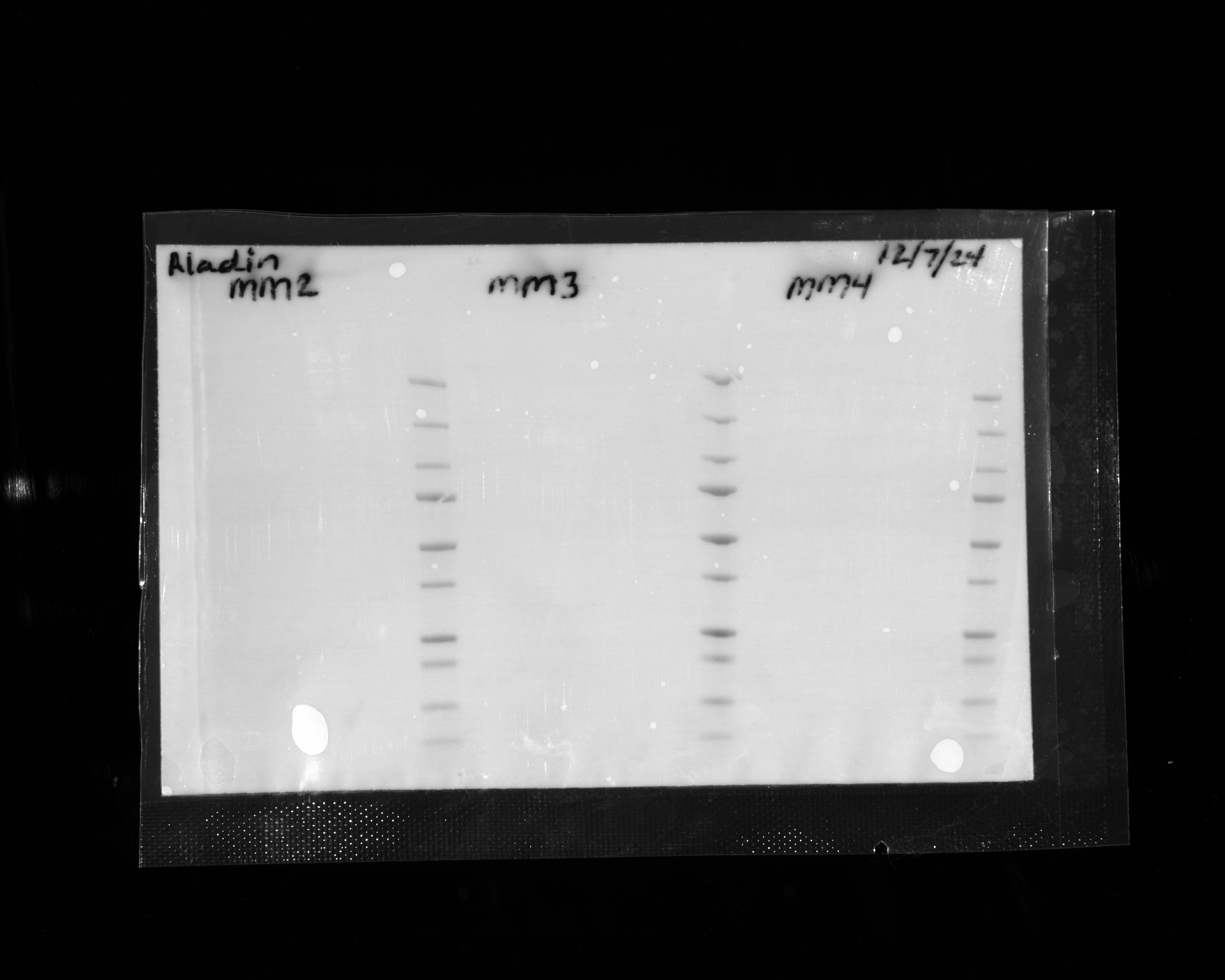

Supplement: Figure 1—source data 7. [file elife-108672-fig1-data7.zip › Fig 1C (part 1)/Aladin/Aladin_Colorimetric imaging_from StainFree 7DEC #1_2Apro_MM2_3_4.tif]

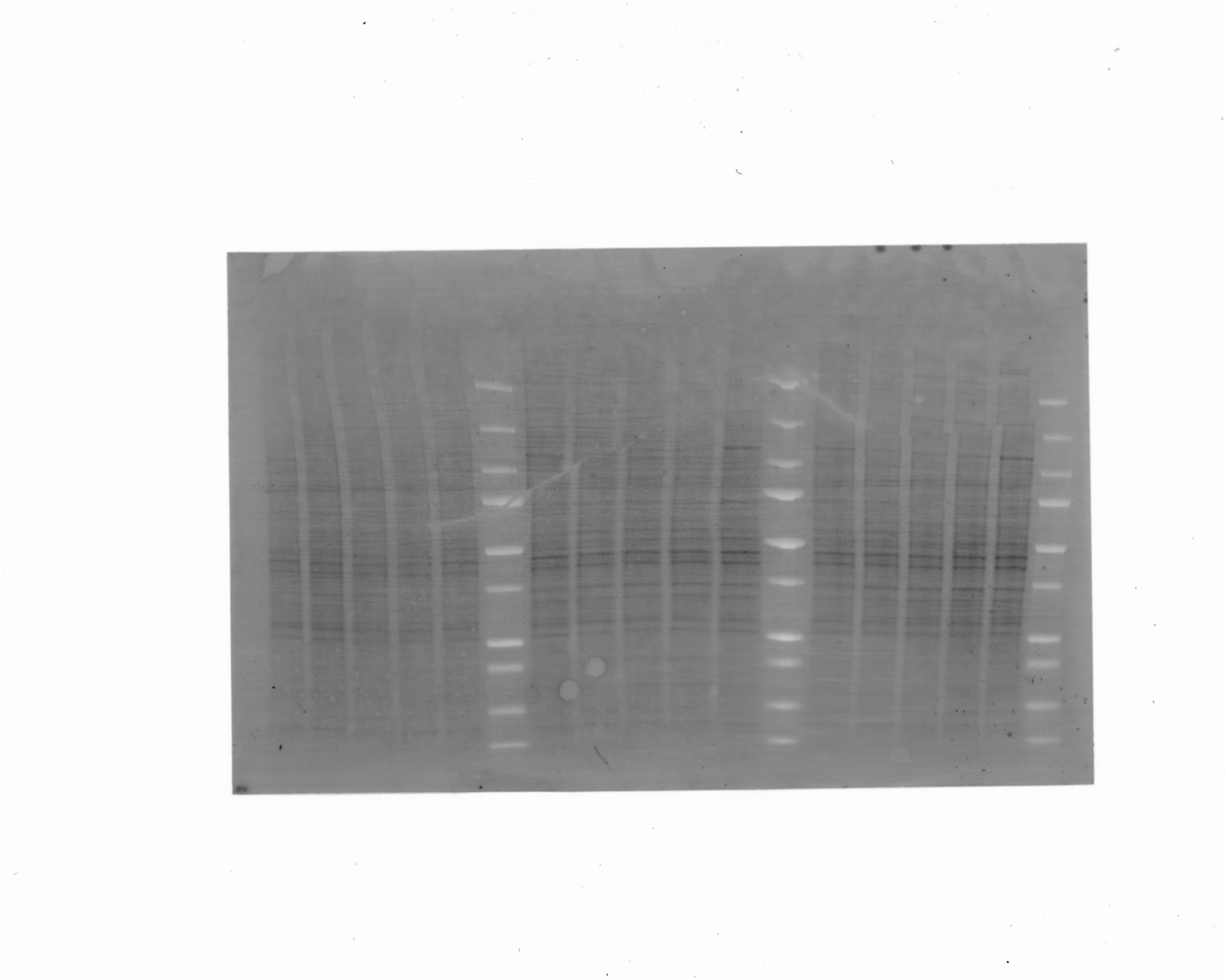

Supplement: Figure 1—source data 7. [file elife-108672-fig1-data7.zip › Fig 1C (part 1)/Aladin/Stain Free_7DEC_Blot #1_2Apro_AFTER transfer_MM2_3_4 lysates.tif]

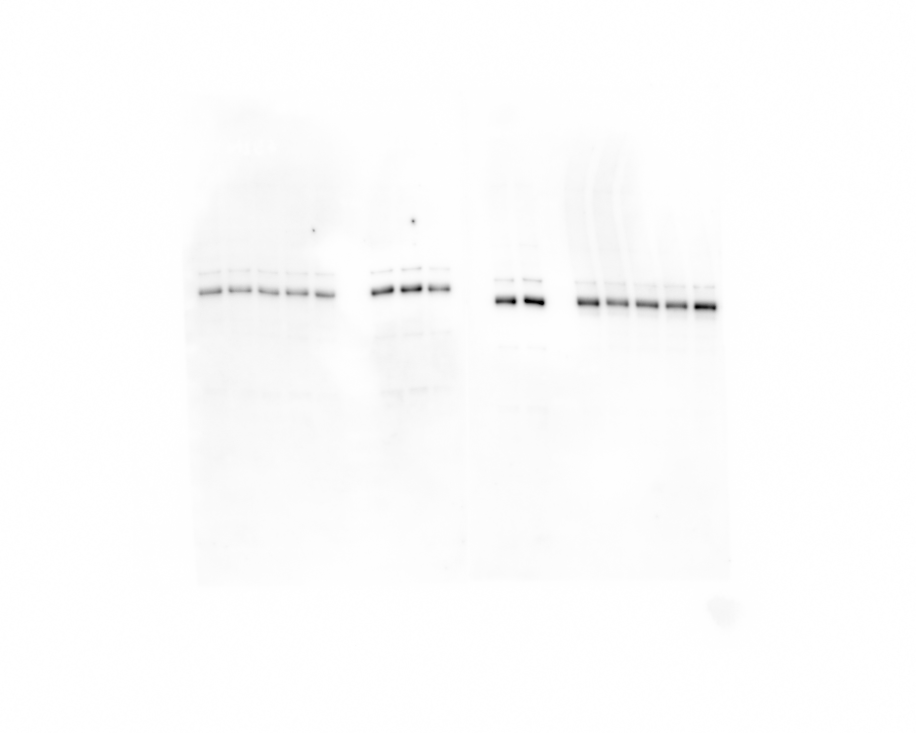

Supplement: Figure 1—source data 7. [file elife-108672-fig1-data7.zip › Fig 1C (part 1)/Gle1/Gle1_Chemi imaging_from StainFree 12AUG #3 and #4_2Apro_MJE3_5_6 Lysates.tif]

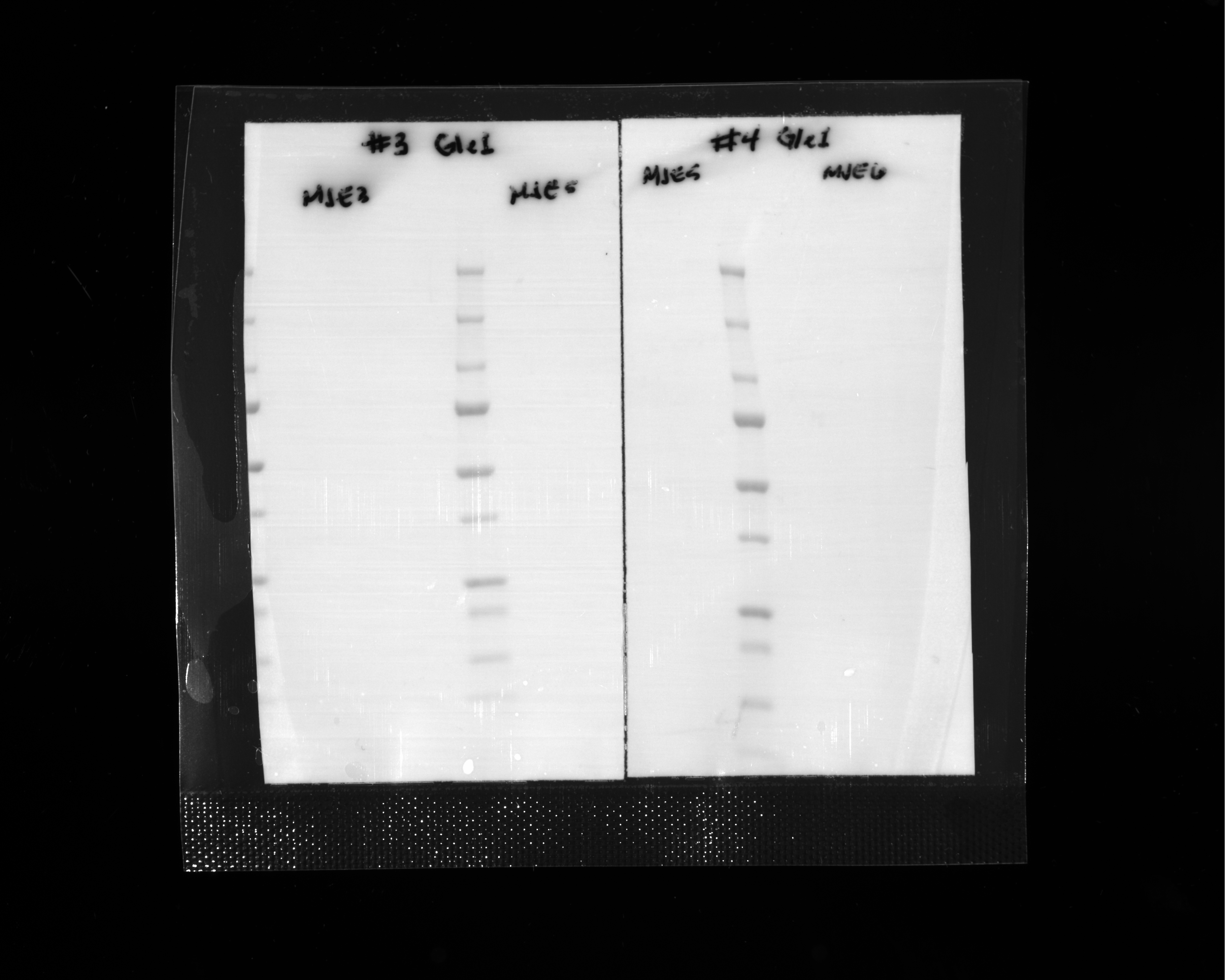

Supplement: Figure 1—source data 7. [file elife-108672-fig1-data7.zip › Fig 1C (part 1)/Gle1/Gle1_Colorimetric imaging_from StainFree 12AUG #3 and #4_2Apro_MJE3_5_6 Lysates.tif]

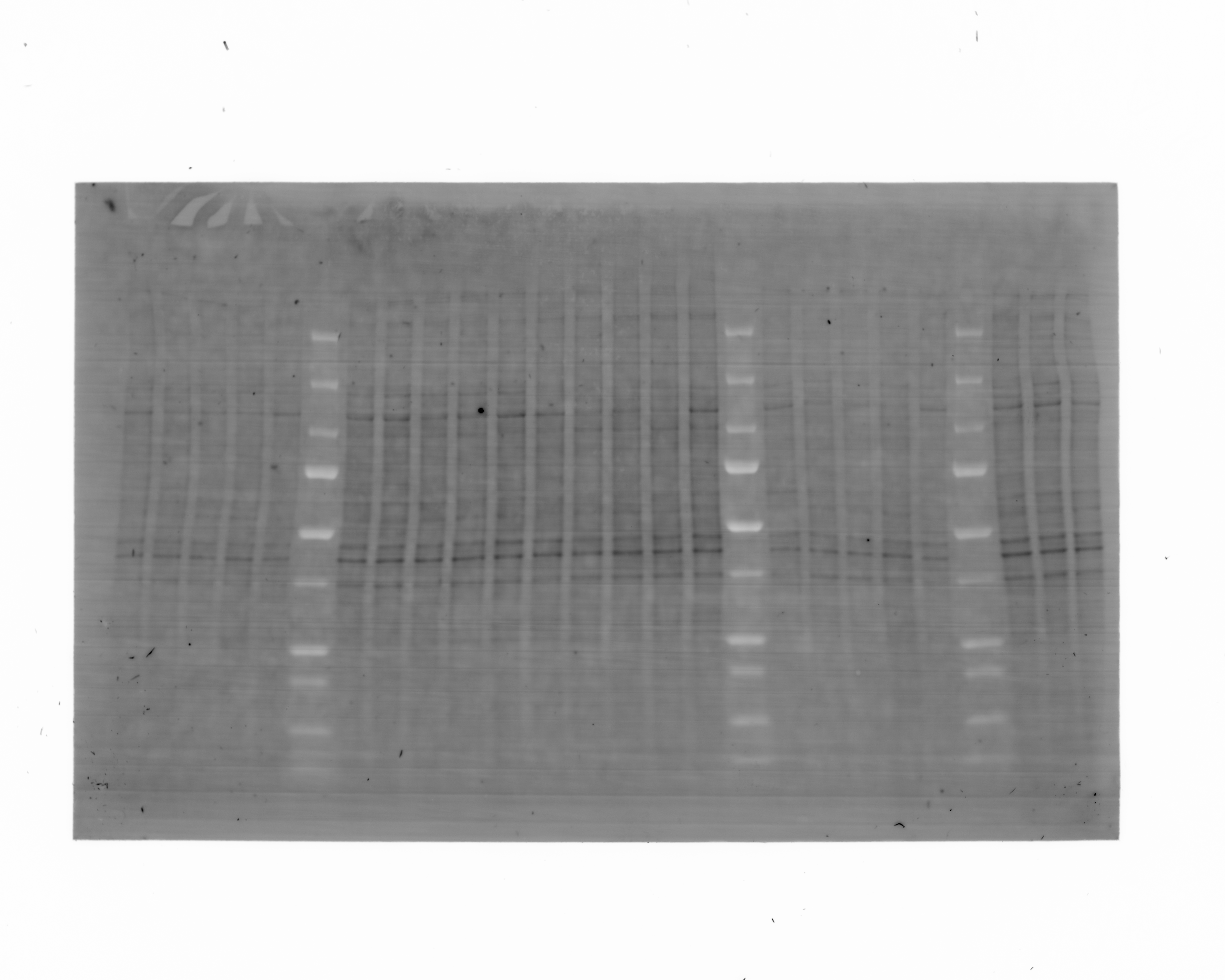

Supplement: Figure 1—source data 7. [file elife-108672-fig1-data7.zip › Fig 1C (part 1)/Gle1/Stain free_12AUG_Blot #3_2Apro_AFTER transfer_MJE3_4_5 Lysates.tif]

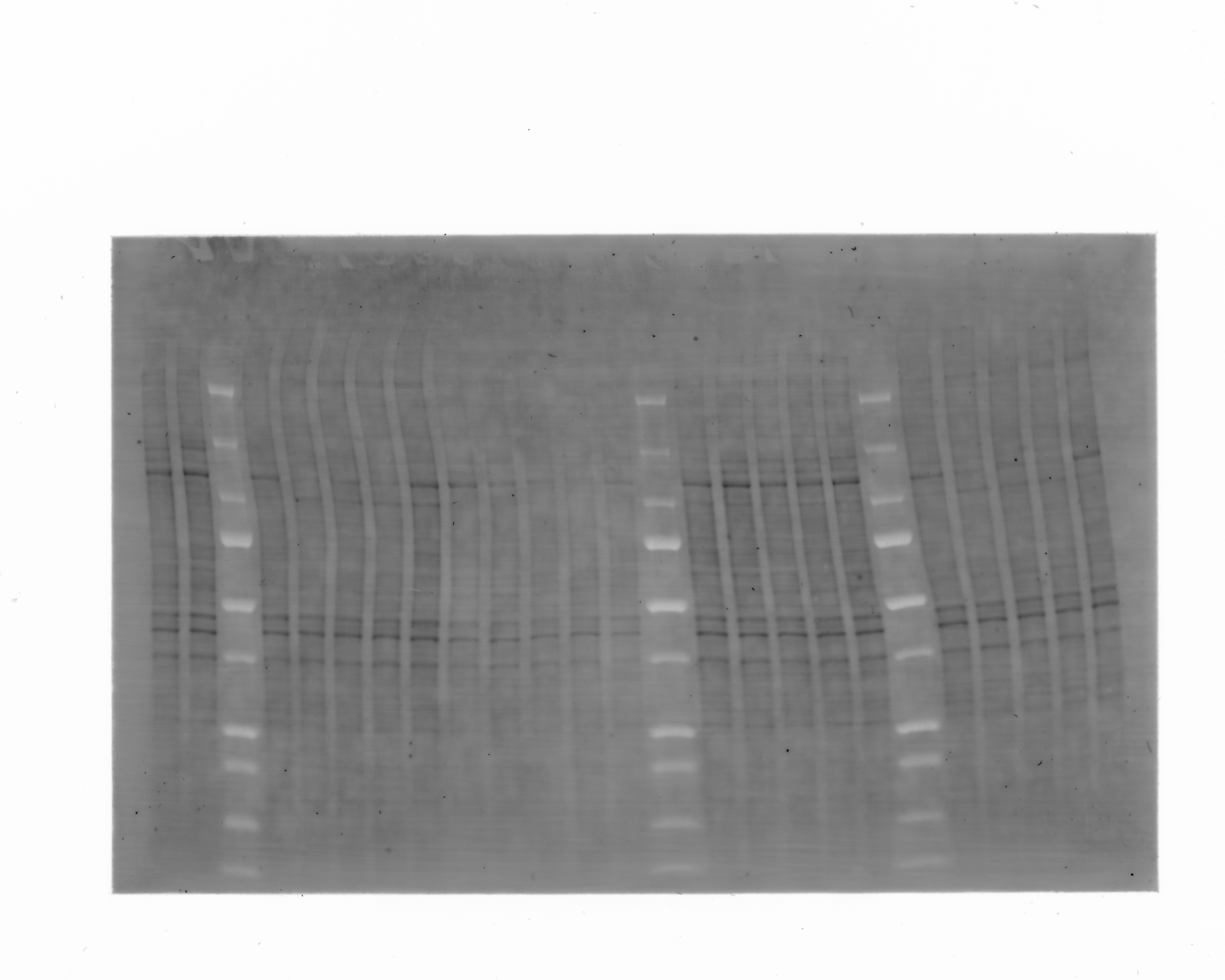

Supplement: Figure 1—source data 7. [file elife-108672-fig1-data7.zip › Fig 1C (part 1)/Gle1/Stain free_12AUG_Blot #4_2Apro_AFTER transfer_MJE3_4_5 Lysates.tif]

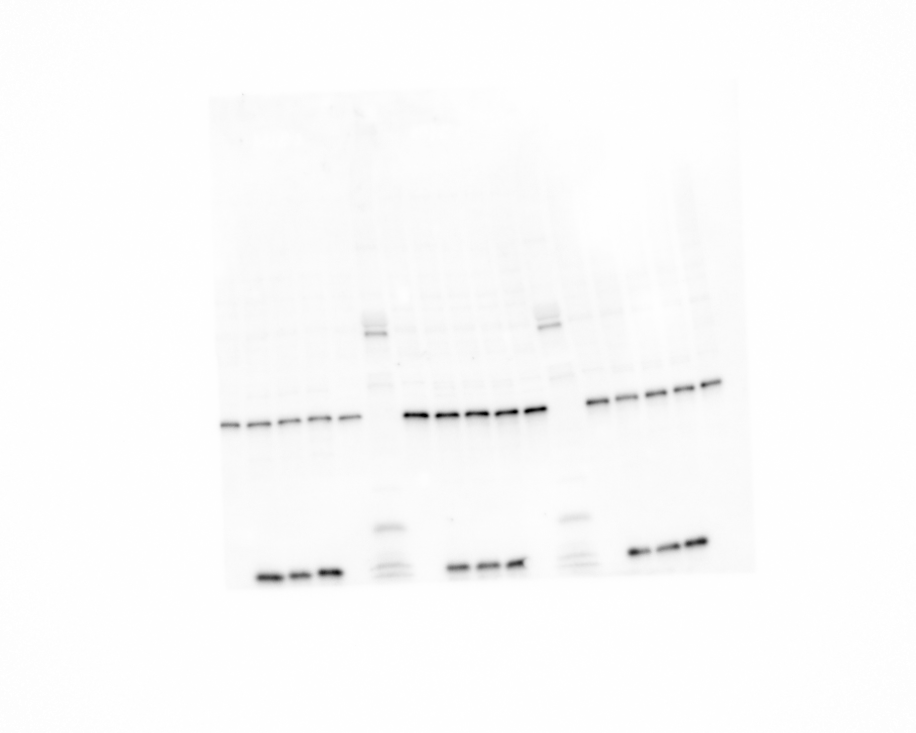

Supplement: Figure 1—source data 7. [file elife-108672-fig1-data7.zip › Fig 1C (part 1)/Nup35/Nup35-53_Chemi imaging_from StainFree 12AUG #4_2Apro_MJE3_5_6 Lysates.tif]

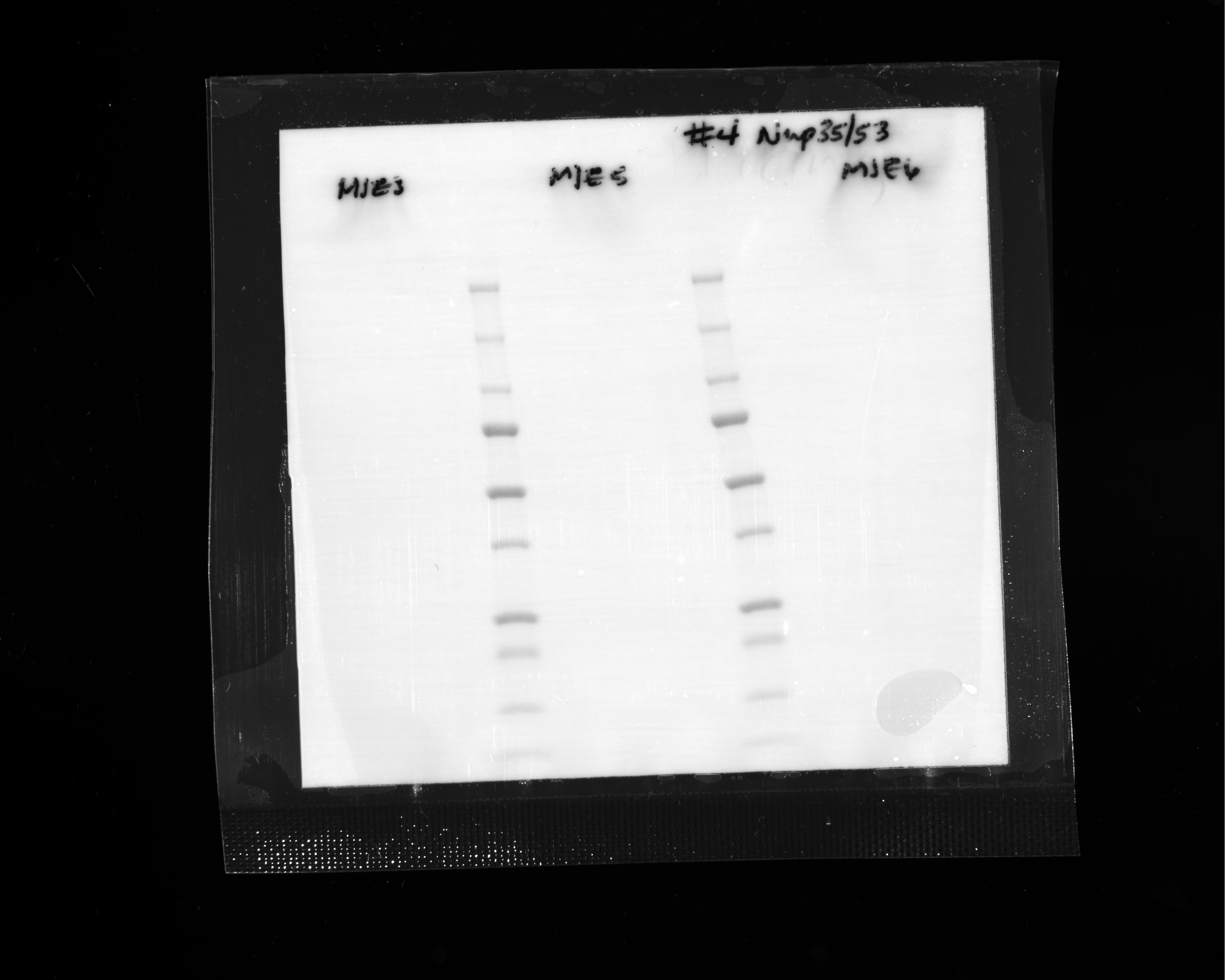

Supplement: Figure 1—source data 7. [file elife-108672-fig1-data7.zip › Fig 1C (part 1)/Nup35/Nup35-53_Colorimetric imaging_from StainFree 12AUG #4_2Apro_MJE3_5_6 Lysates.tif]

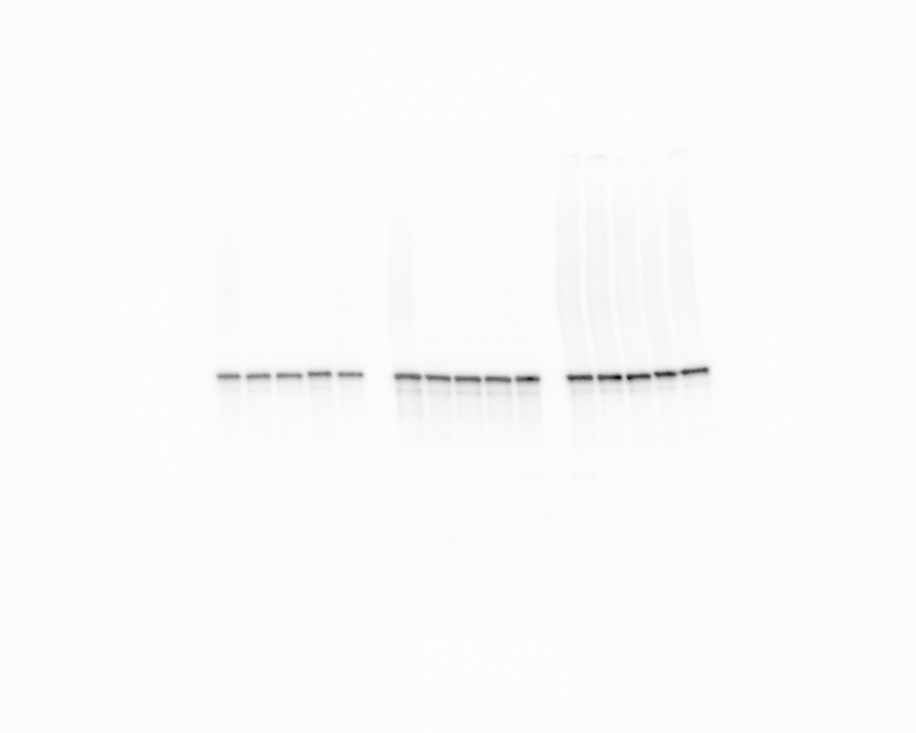

Supplement: Figure 1—source data 7. [file elife-108672-fig1-data7.zip › Fig 1C (part 1)/Nup54/Nup54_Chemi imaging_from StainFree 19AUG #6_2Apro_MJE3_5_6 Lysates.tif]
