# Supplementary figures and images for: Enterovirus D68 2A protease causes nuclear pore complex dysfunction and independently contributes to motor neuron toxicity (part 3 of 4)
Source: eLife. 2026 Jun 18;14:RP108672. doi: 10.7554/eLife.108672 (PMC13278737; doi:10.7554/eLife.108672)

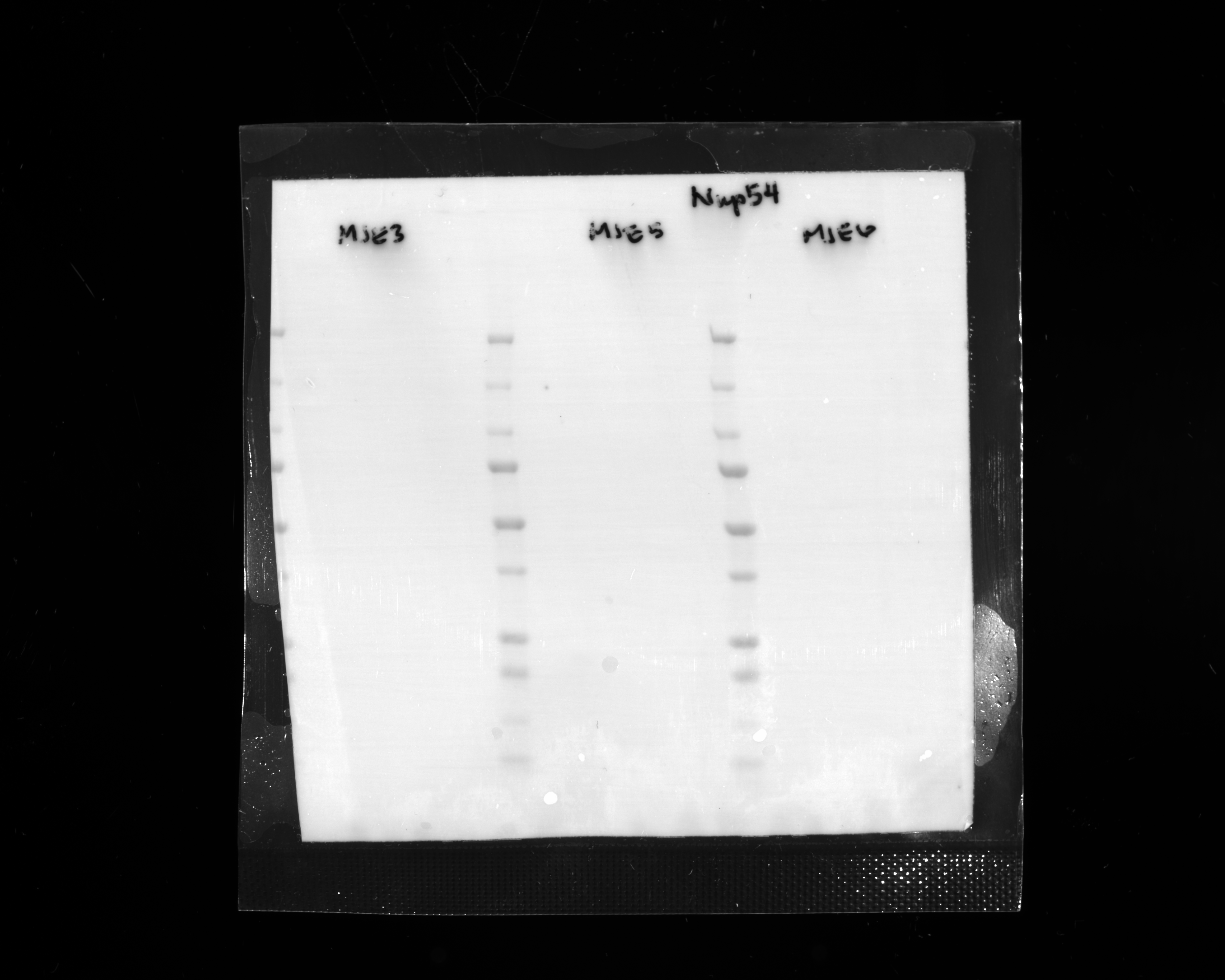

Supplement: Figure 1—source data 7. [file elife-108672-fig1-data7.zip › Fig 1C (part 1)/Nup54/Nup54_Colorimetric imaging_from StainFree 19AUG #6_2Apro_MJE3_5_6 Lysates.tif]

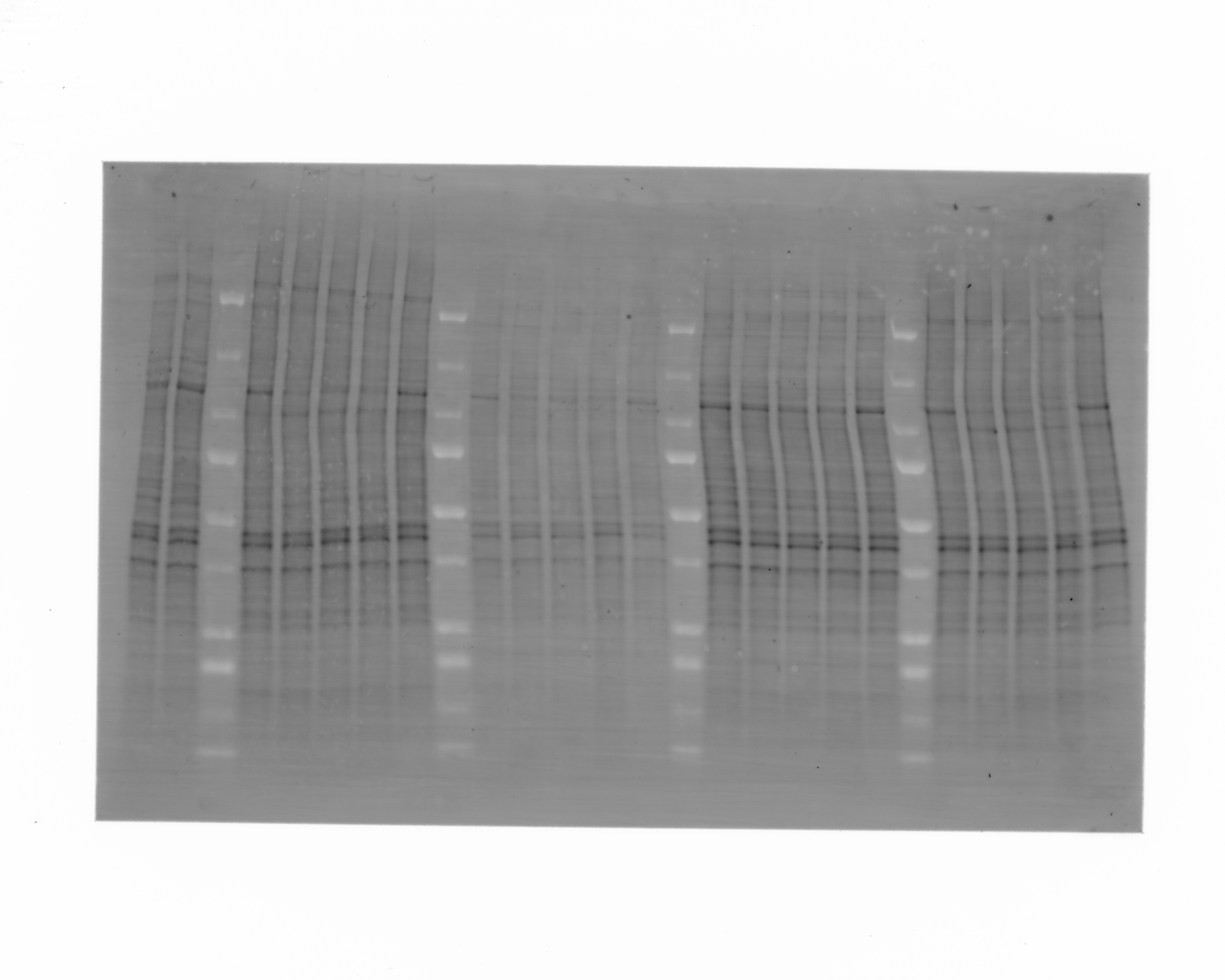

Supplement: Figure 1—source data 7. [file elife-108672-fig1-data7.zip › Fig 1C (part 1)/Nup54/Stain Free_19AUG24_Blot #6_2Apro_AFTER transfer_MJE3_5_6 Lysates.tif]

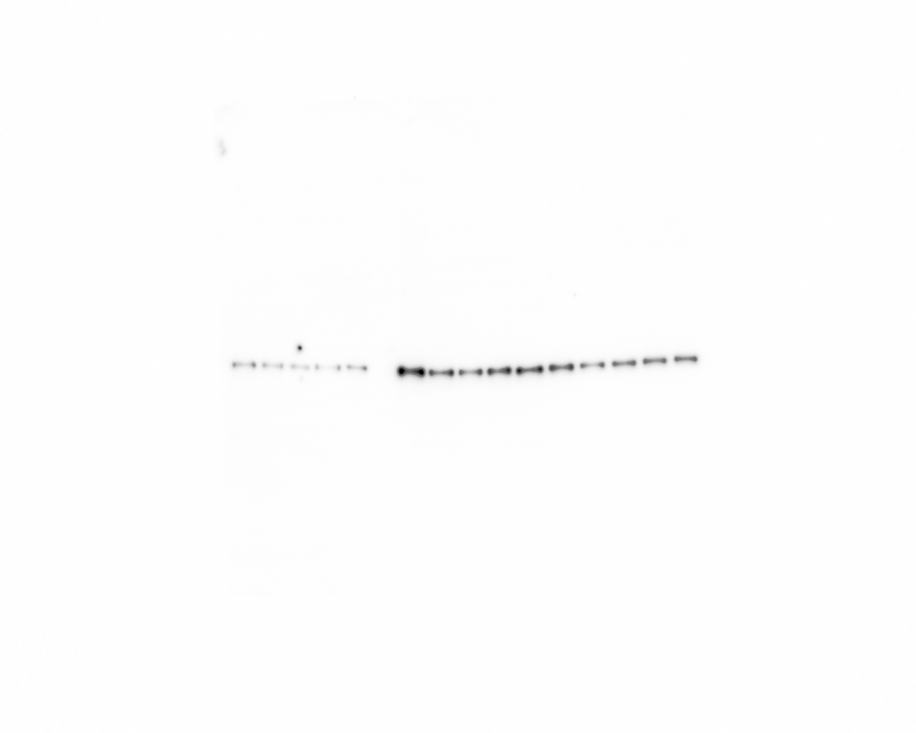

Supplement: Figure 1—source data 7. [file elife-108672-fig1-data7.zip › Fig 1C (part 1)/Nup62/Chemi Nup62 8-22_4.tif]

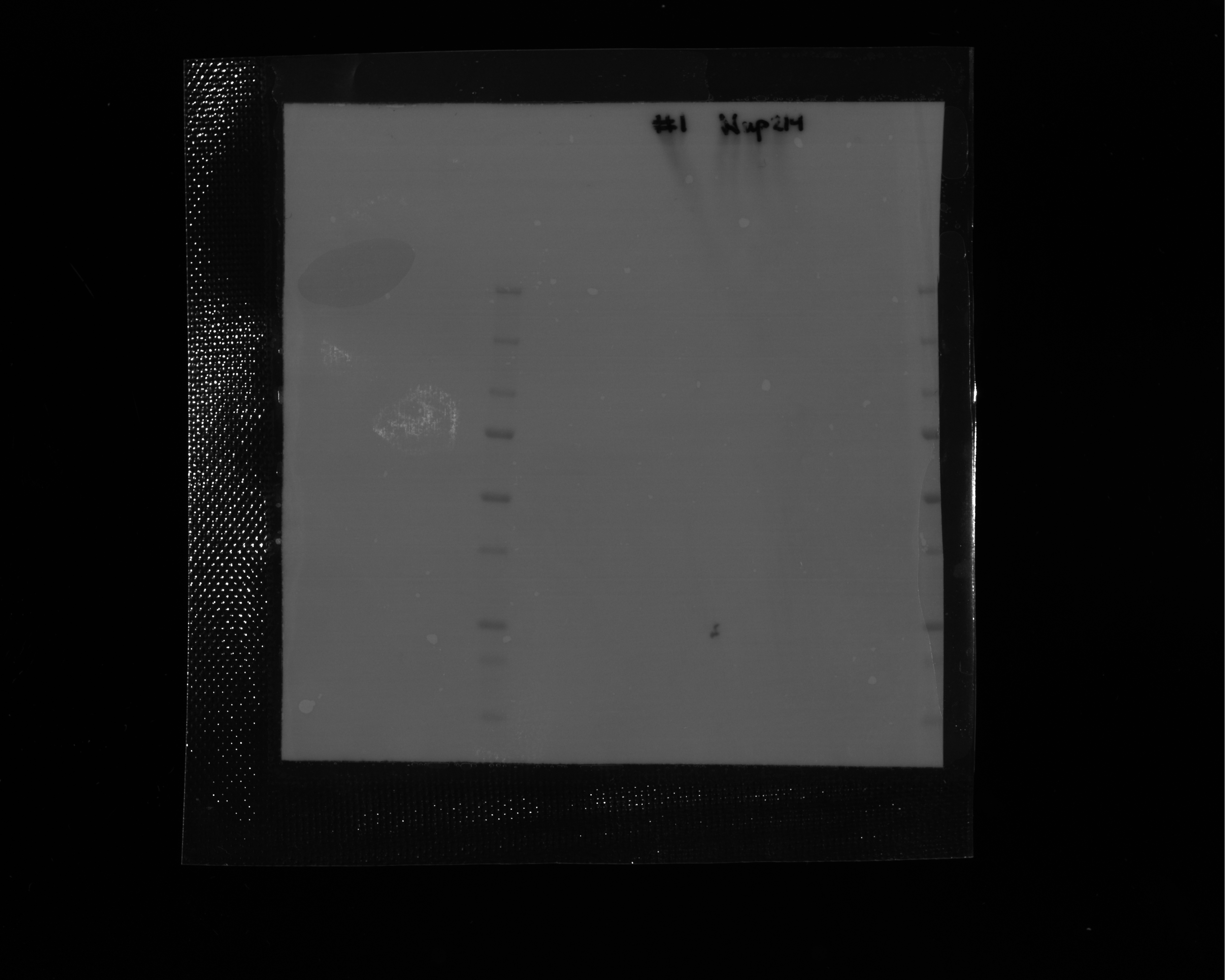

Supplement: Figure 1—source data 7. [file elife-108672-fig1-data7.zip › Fig 1C (part 1)/Nup62/Nup62 ladder (overblot of Nup214).tif]

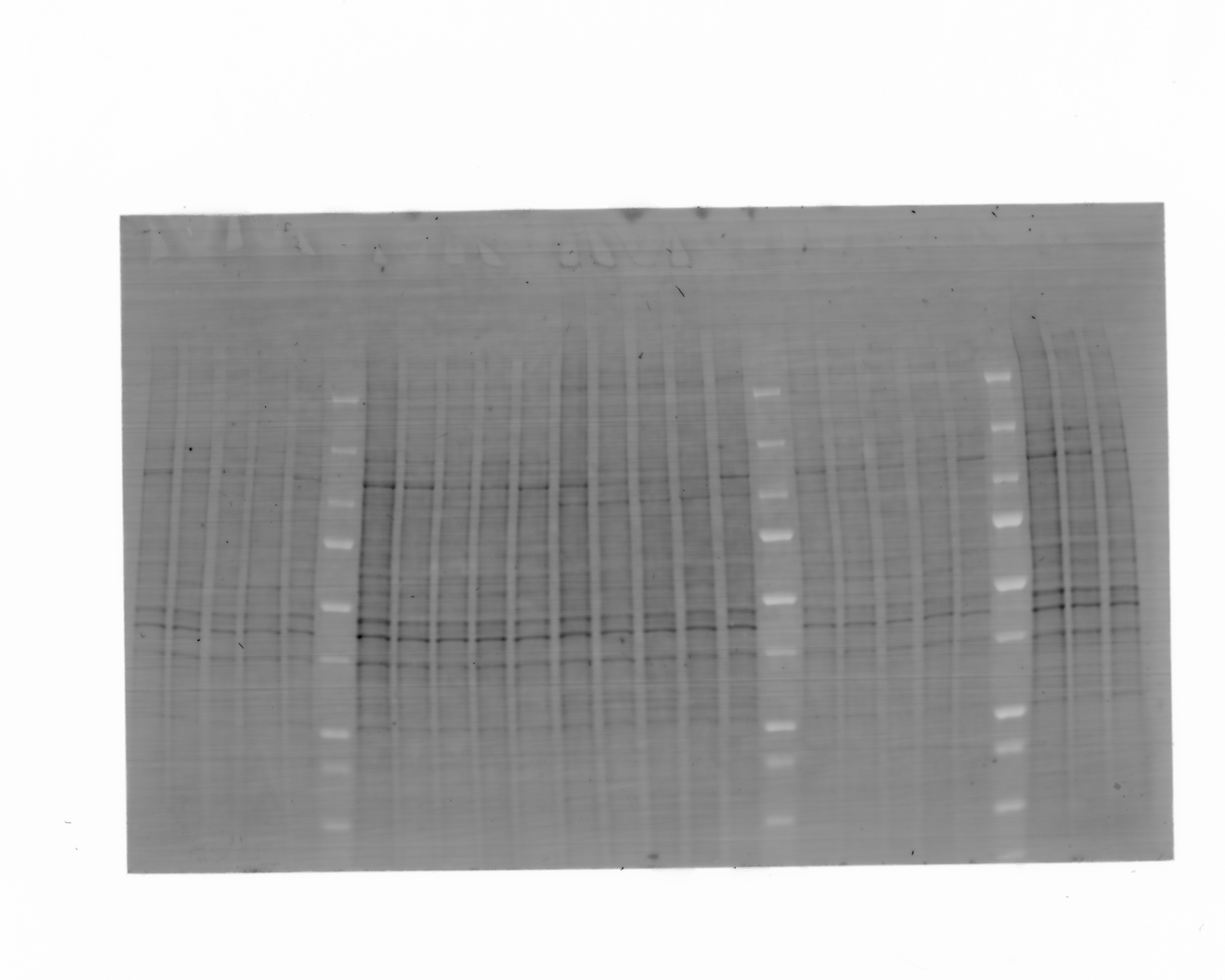

Supplement: Figure 1—source data 7. [file elife-108672-fig1-data7.zip › Fig 1C (part 1)/Nup62/Stain free_12AUG_Blot #1_2Apro_AFTER transfer_MJE3_5_6 Lysates.tif]

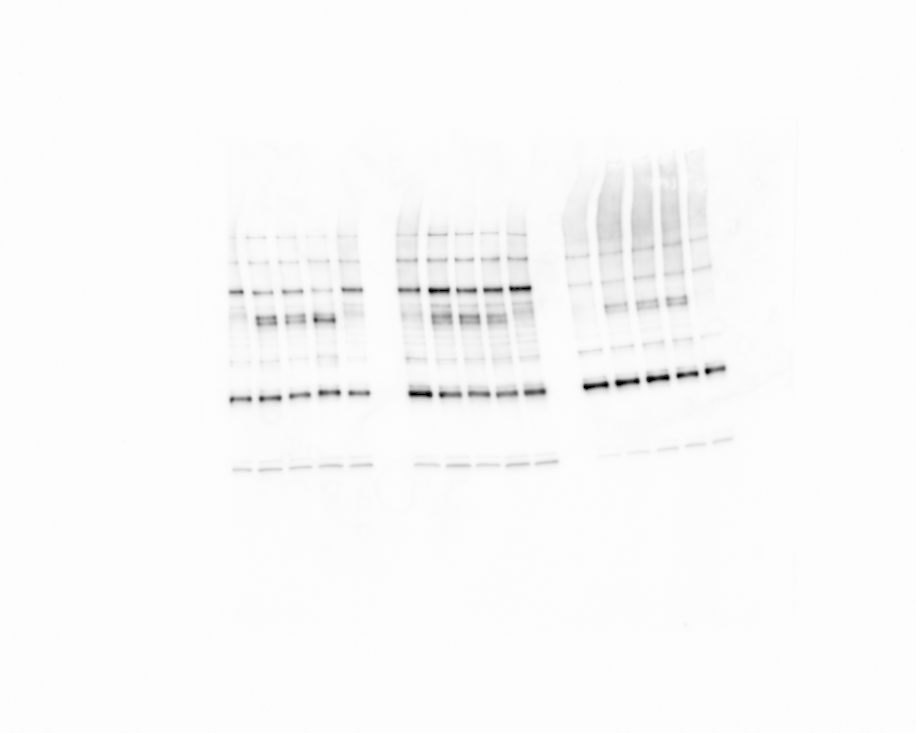

Supplement: Figure 1—source data 8. [file elife-108672-fig1-data8.zip › Fig 1C (part 2)/Nup153/Nup153_Chemi imaging_22AUG_from StainFree 12AUG #4_2Apro_MJE3_5_6 Lysates.tif]

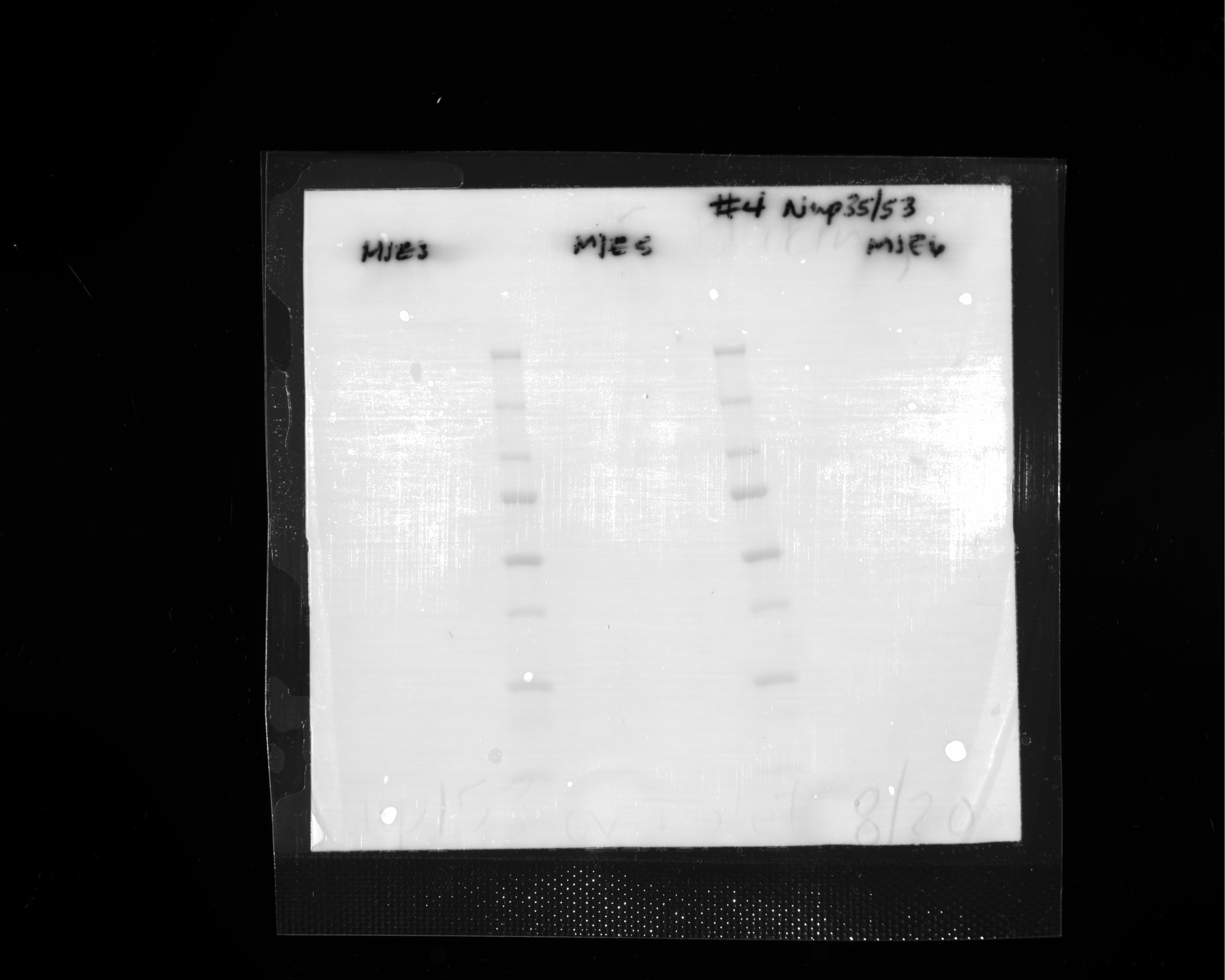

Supplement: Figure 1—source data 8. [file elife-108672-fig1-data8.zip › Fig 1C (part 2)/Nup153/Nup153_Colorimetric imaging_22AUG_from StainFree 12AUG #4_2Apro_MJE3_5_6 Lysates.tif]

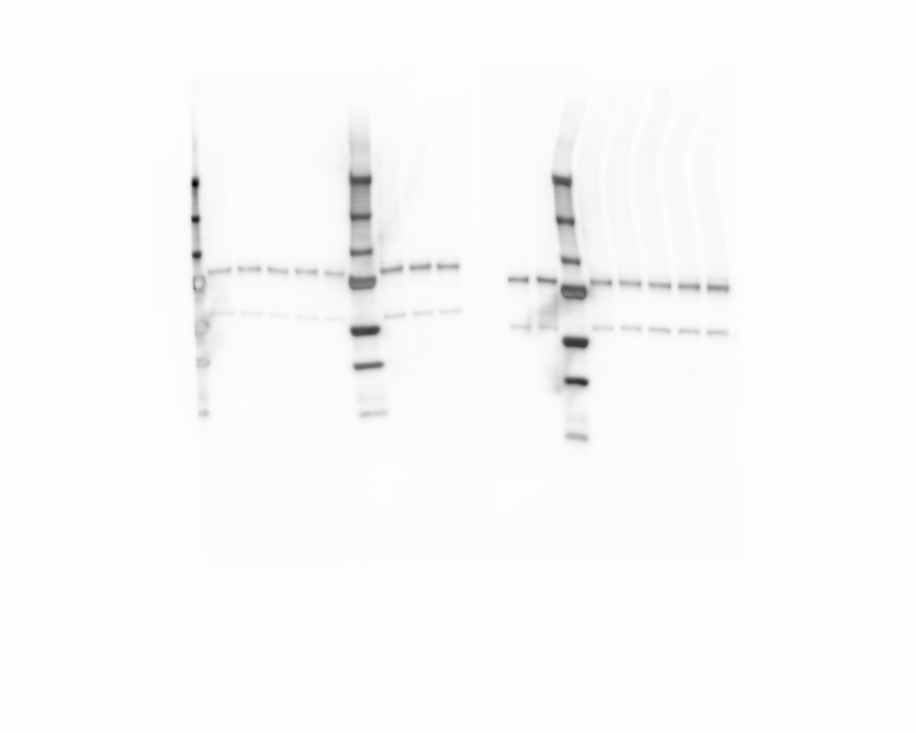

Supplement: Figure 1—source data 8. [file elife-108672-fig1-data8.zip › Fig 1C (part 2)/Nup88/Chemi Nup88.tif]

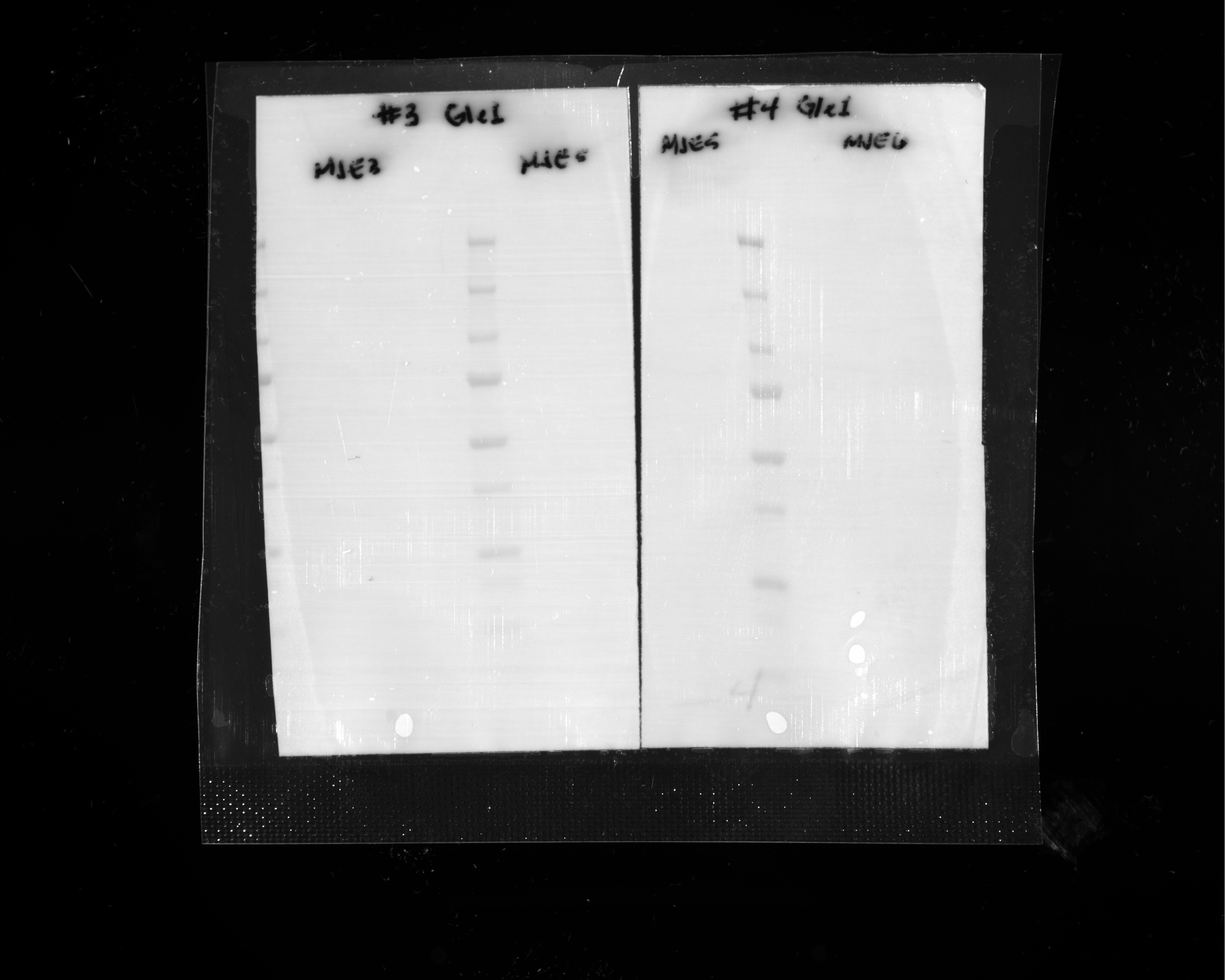

Supplement: Figure 1—source data 8. [file elife-108672-fig1-data8.zip › Fig 1C (part 2)/Nup88/Coloro Nup88.tif]

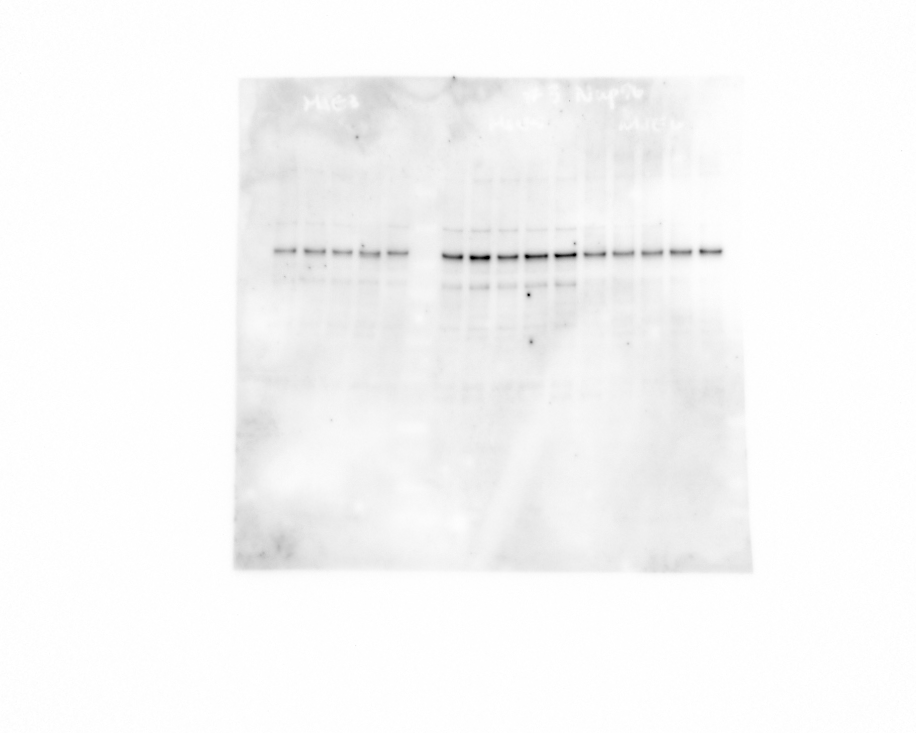

Supplement: Figure 1—source data 8. [file elife-108672-fig1-data8.zip › Fig 1C (part 2)/Nup96/Nup96_Chemi imaging_from StainFree 12AUG #3_2Apro_MJE3_5_6 Lysates.tif]

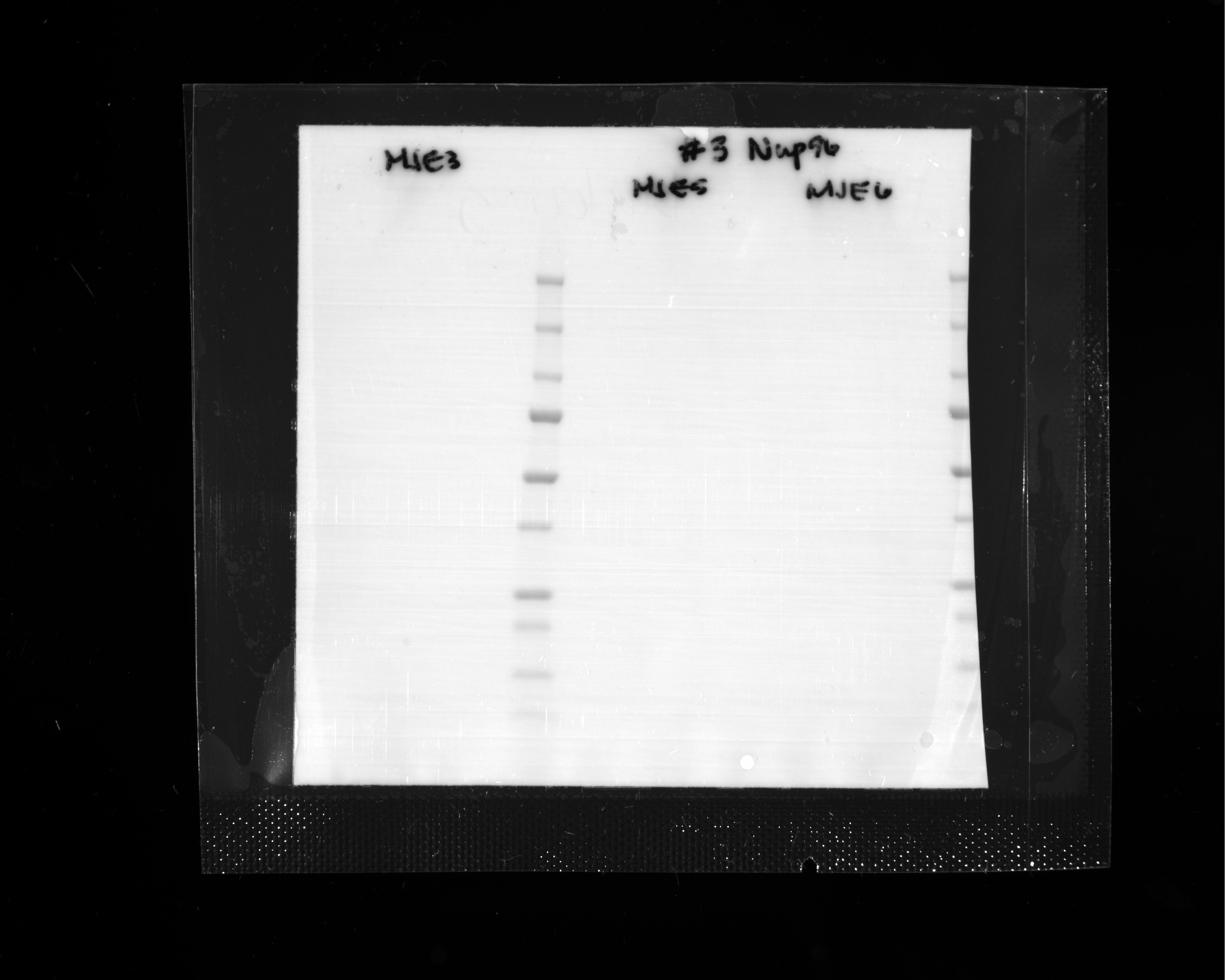

Supplement: Figure 1—source data 8. [file elife-108672-fig1-data8.zip › Fig 1C (part 2)/Nup96/Nup96_Colorimetric imaging_from StainFree 12AUG #3_2Apro_MJE3_5_6 Lysates.tif]

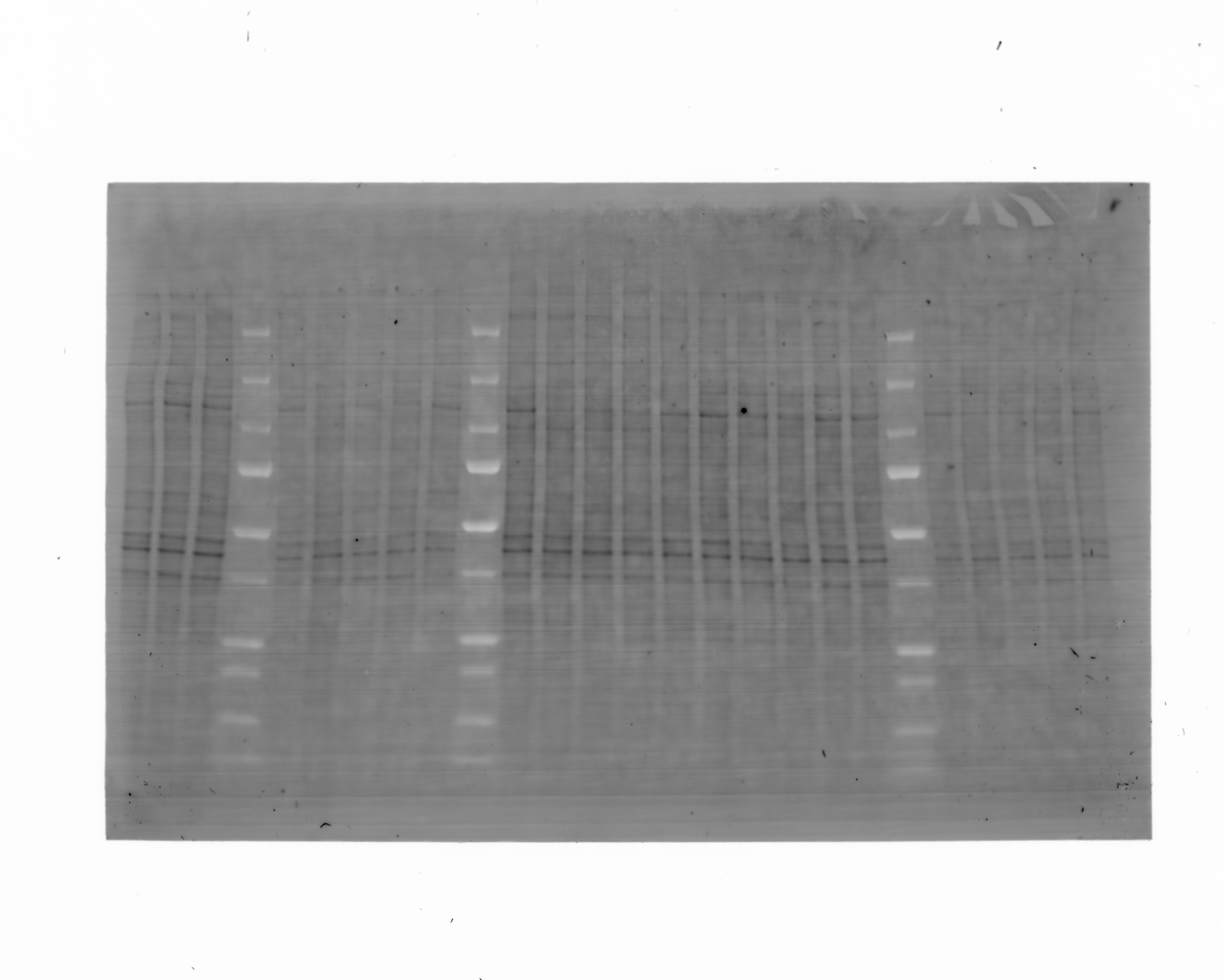

Supplement: Figure 1—source data 8. [file elife-108672-fig1-data8.zip › Fig 1C (part 2)/Nup96/Stain free_12AUG_Blot #3_2Apro_AFTER transfer_MJE3_5_6 Lysates.tif]

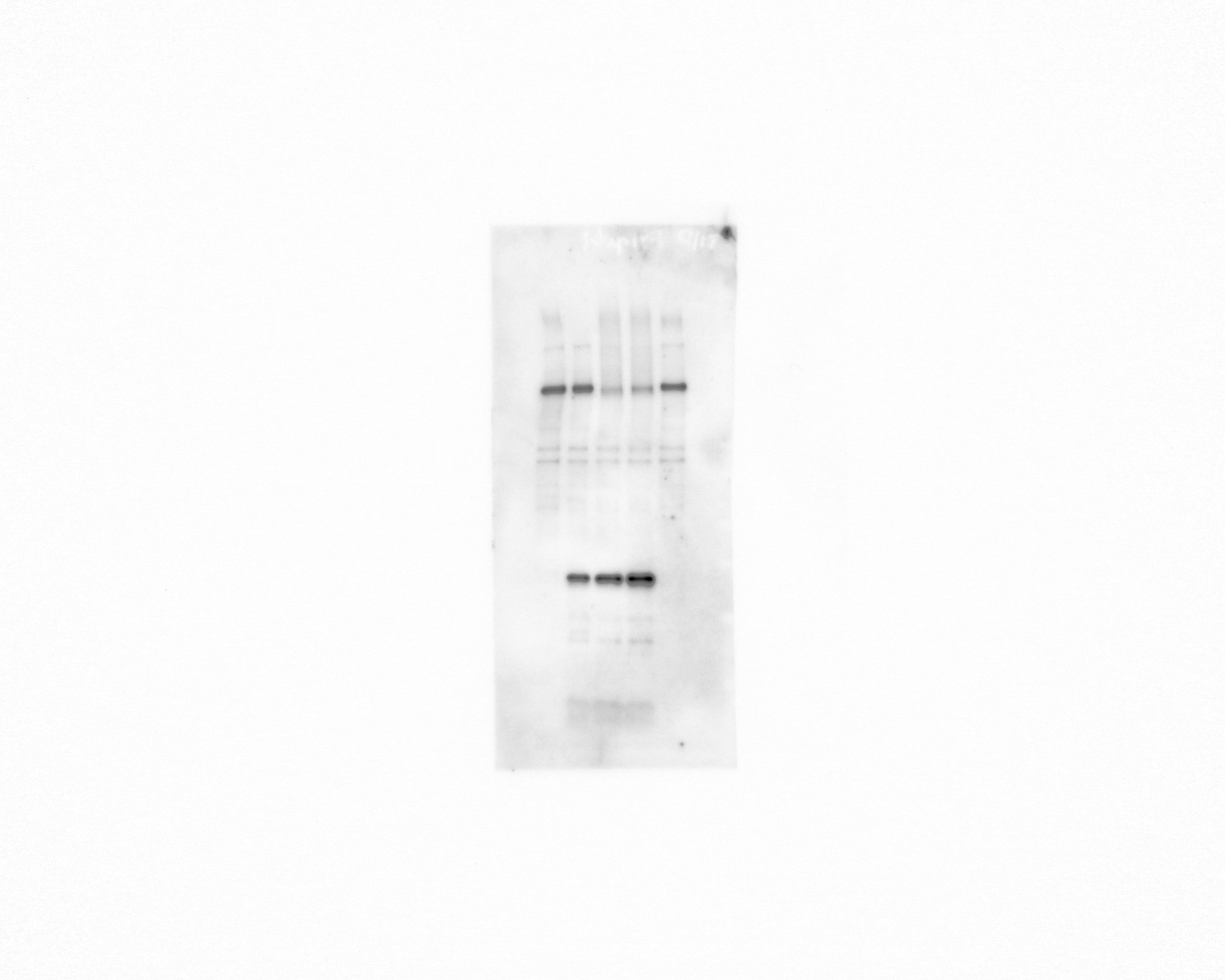

Supplement: Figure 1—source data 8. [file elife-108672-fig1-data8.zip › Fig 1C (part 2)/Nup98/A_Nup98_Chemi imaging_from StainFree 17SEP #1_2Apro_MM2 Lysate.tif]

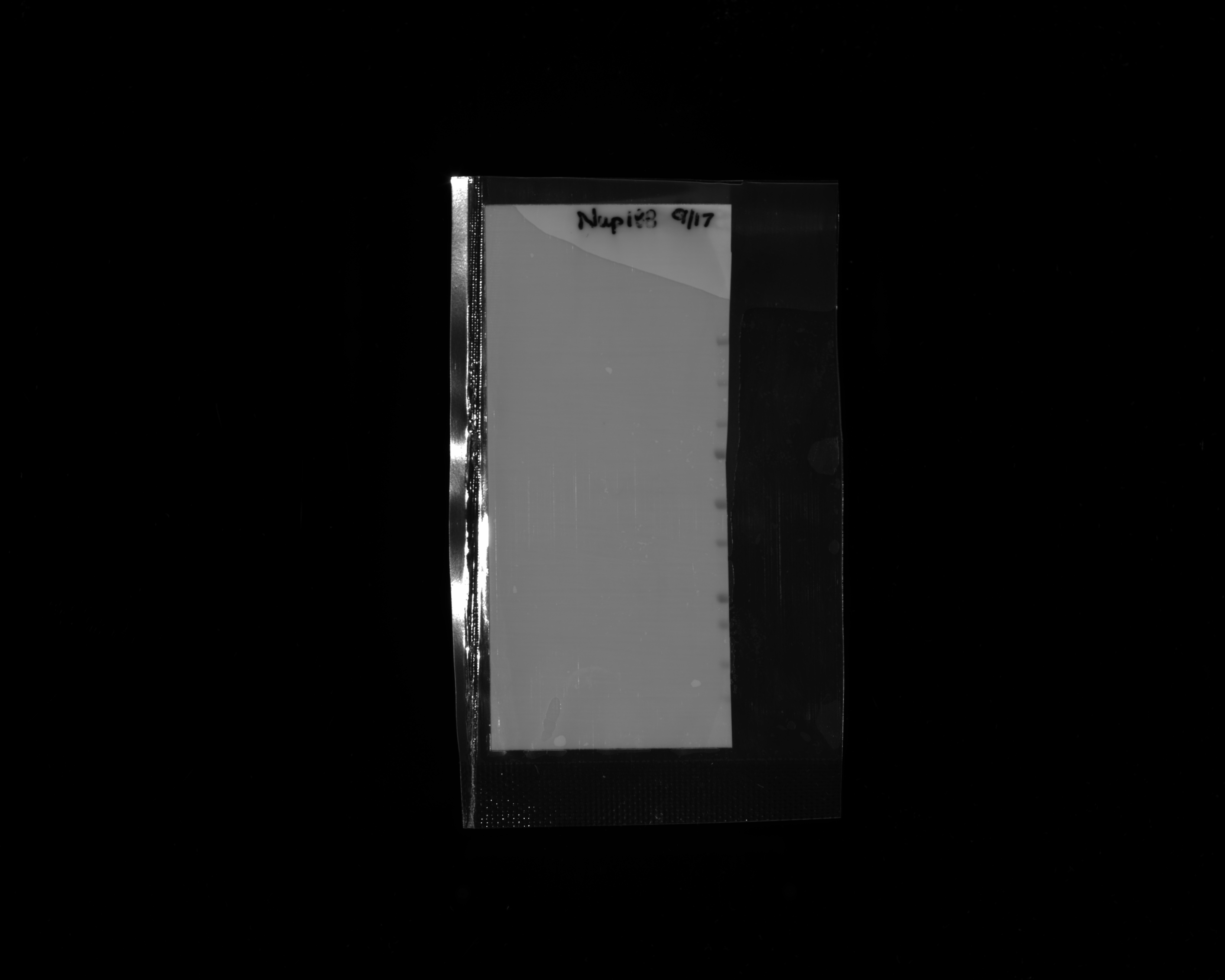

Supplement: Figure 1—source data 8. [file elife-108672-fig1-data8.zip › Fig 1C (part 2)/Nup98/A_Nup98_Colorimetric imaging_from StainFree 17SEP #1_2Apro_MM2 Lysate.tif]

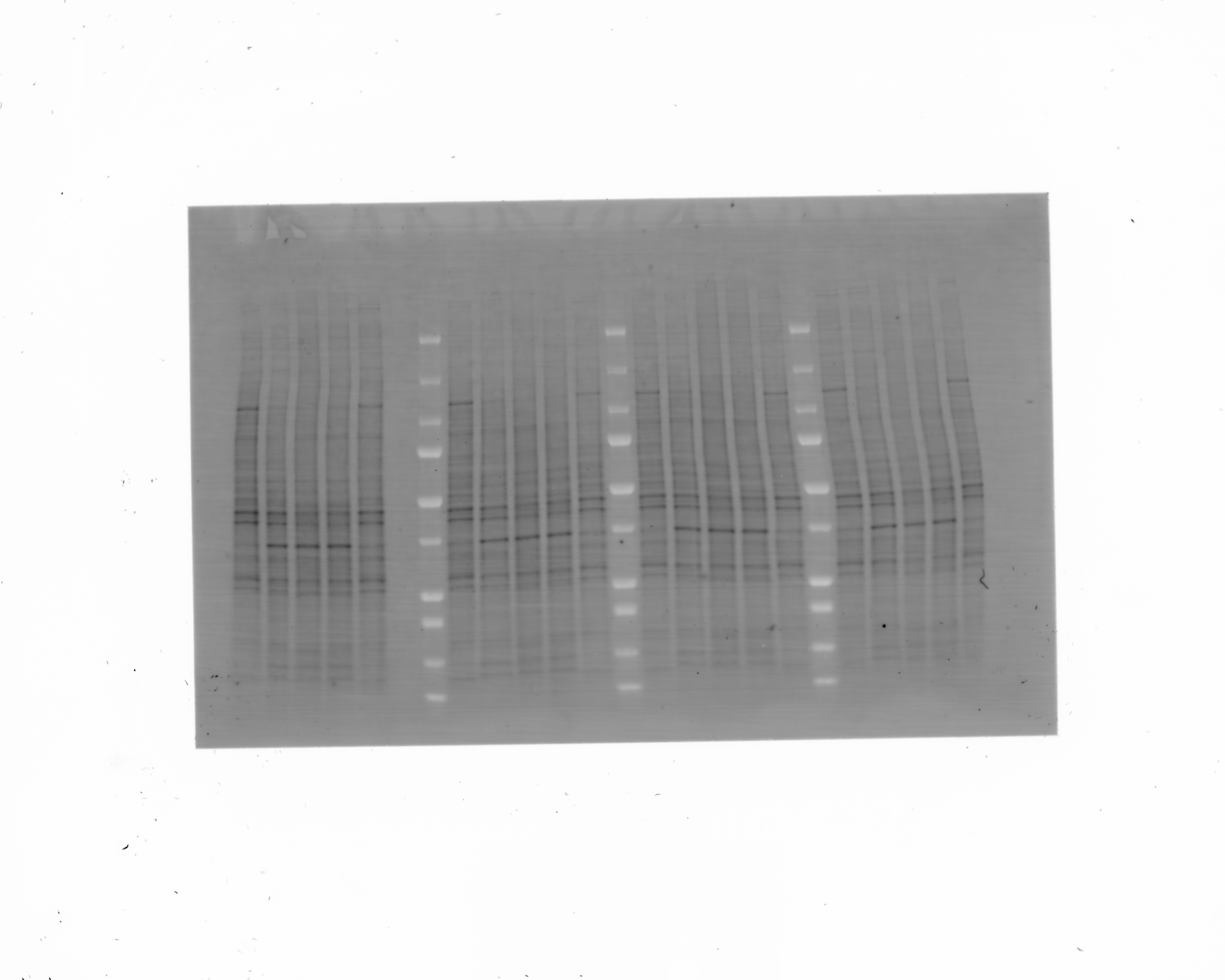

Supplement: Figure 1—source data 8. [file elife-108672-fig1-data8.zip › Fig 1C (part 2)/Nup98/A_Stain Free_17SEP_Blot #1_2Apro_AFTER transfer_MM2 lysates.tif]

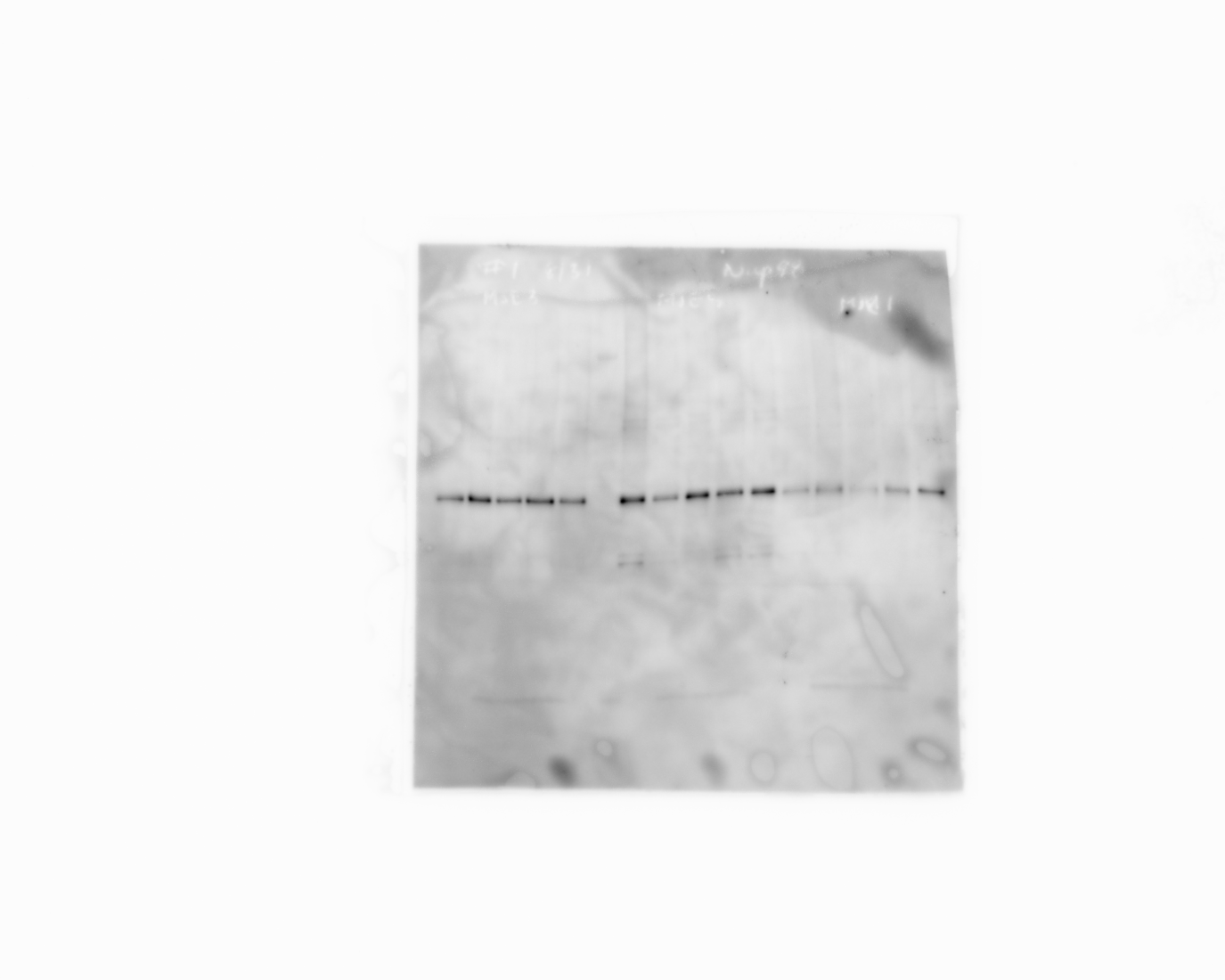

Supplement: Figure 1—source data 8. [file elife-108672-fig1-data8.zip › Fig 1C (part 2)/Nup98/BC_Nup98_Chemi imaging_from StainFree 27AUG #1_2Apro_MJE3_5 Lysates.tif]

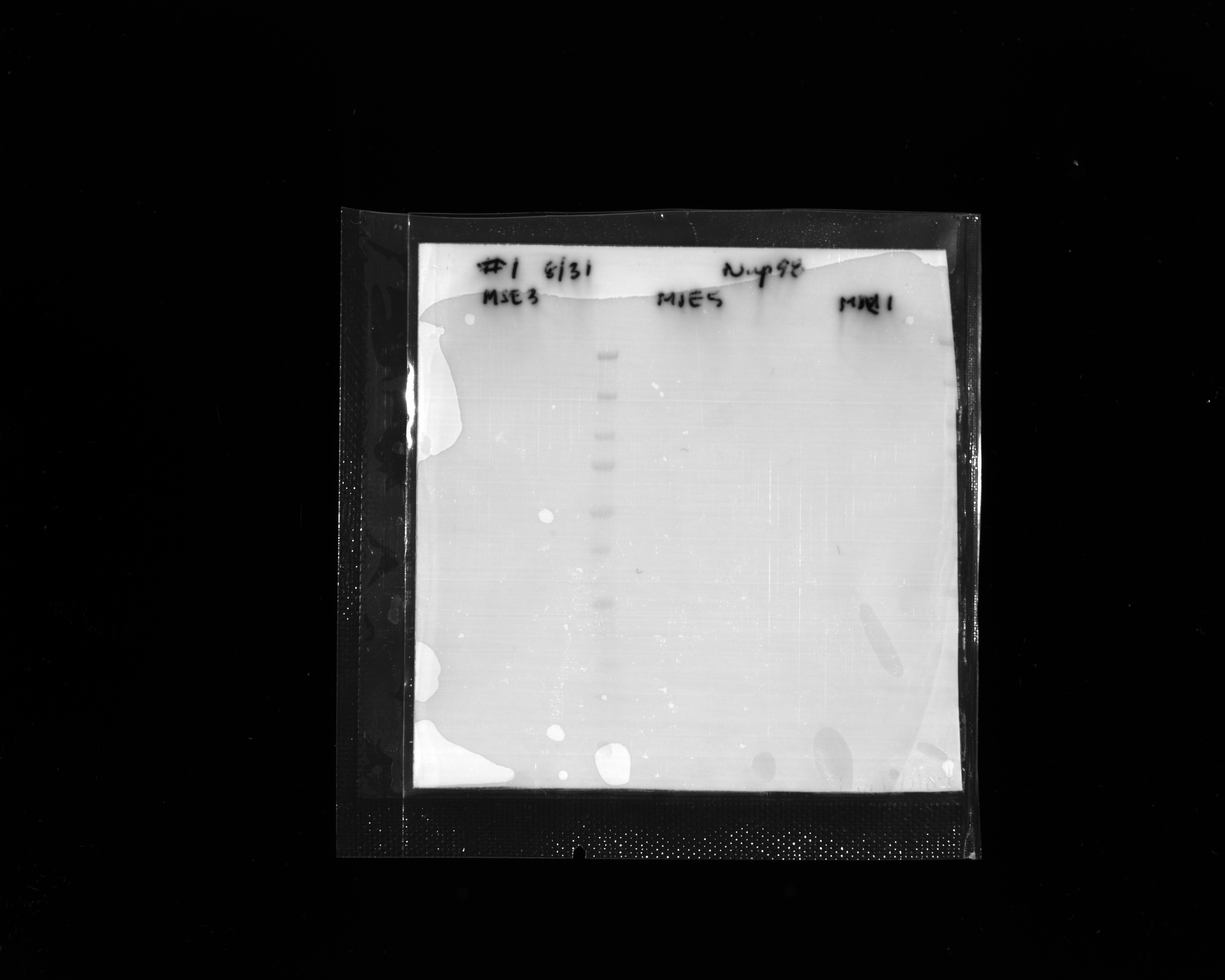

Supplement: Figure 1—source data 8. [file elife-108672-fig1-data8.zip › Fig 1C (part 2)/Nup98/BC_Nup98_Colorimetric imaging_from StainFree 27AUG #1_2Apro_MJE3_5 Lysates.tif]

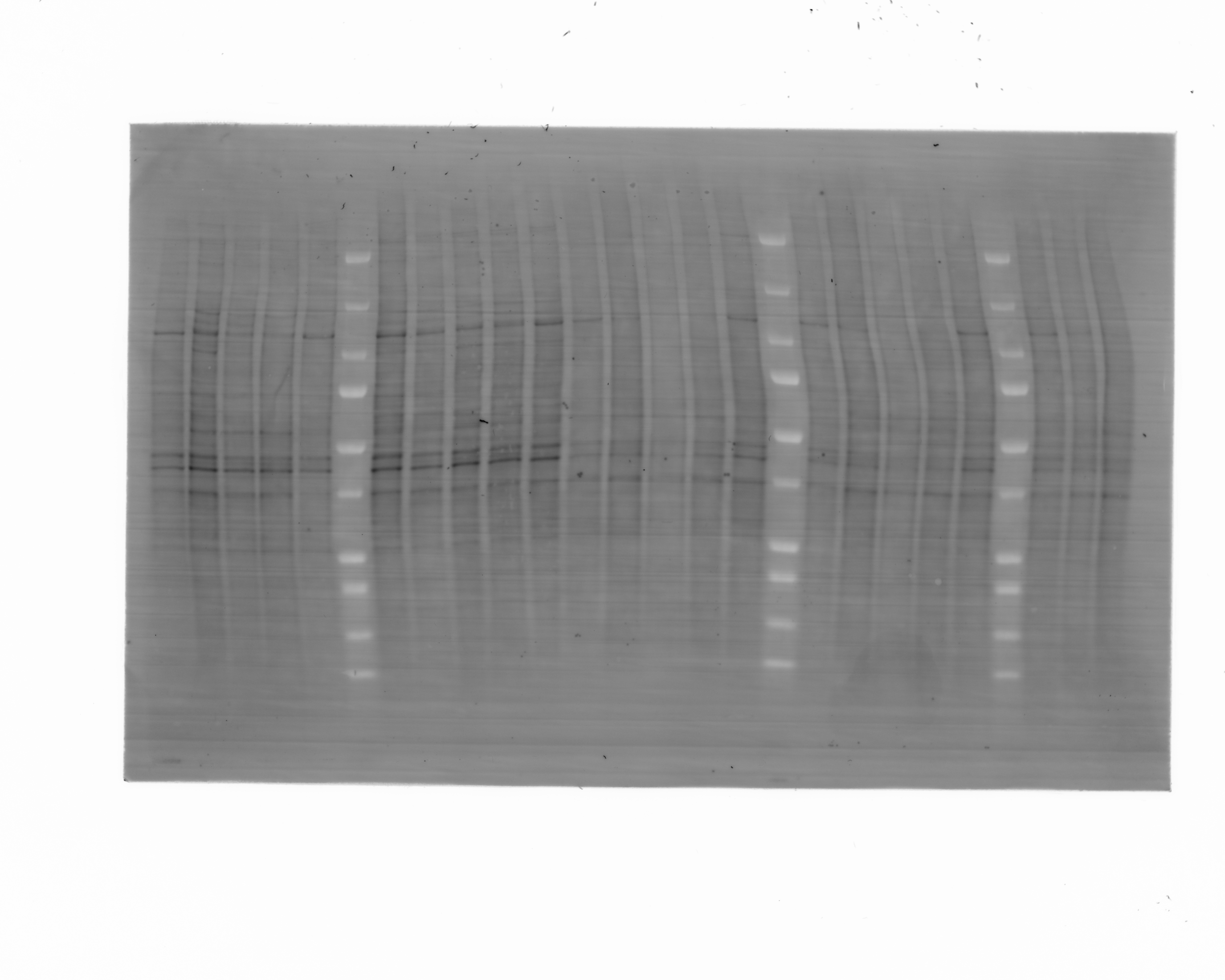

Supplement: Figure 1—source data 8. [file elife-108672-fig1-data8.zip › Fig 1C (part 2)/Nup98/BC_Stain Free_27AUG_Blot #1_2Apro_AFTER transfer_MJE3_5_MM1 Lysates.tif]

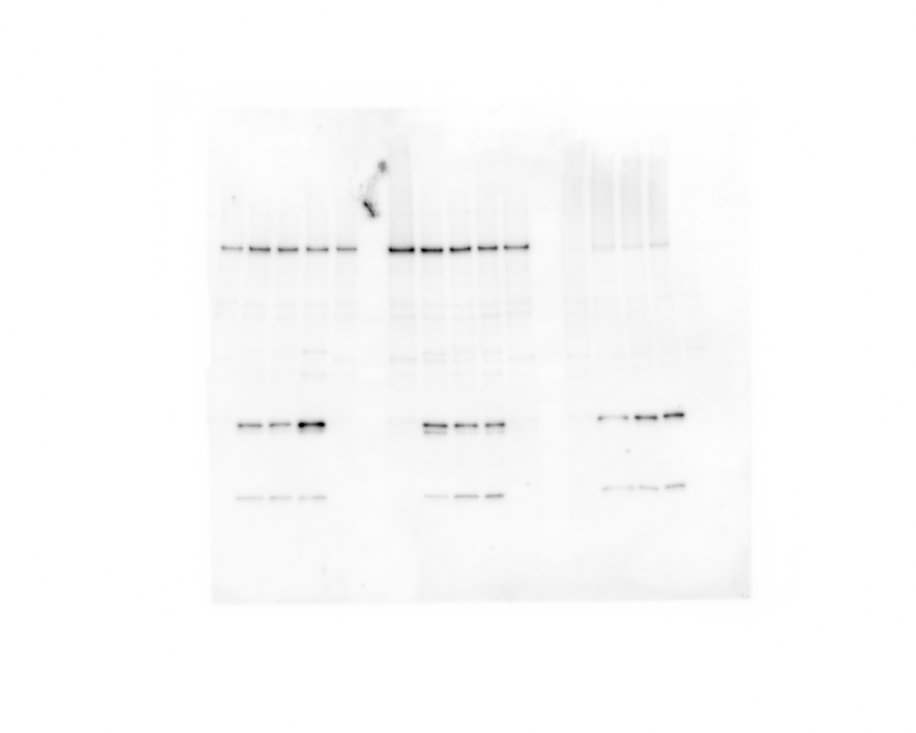

Supplement: Figure 1—source data 9. [file elife-108672-fig1-data9.zip › Fig 1C (part 3)/Nup188/AB_Nup188_Chemi imaging_from StainFree 12AUG #2_2Apro_MJE3_5 lysate.tif]

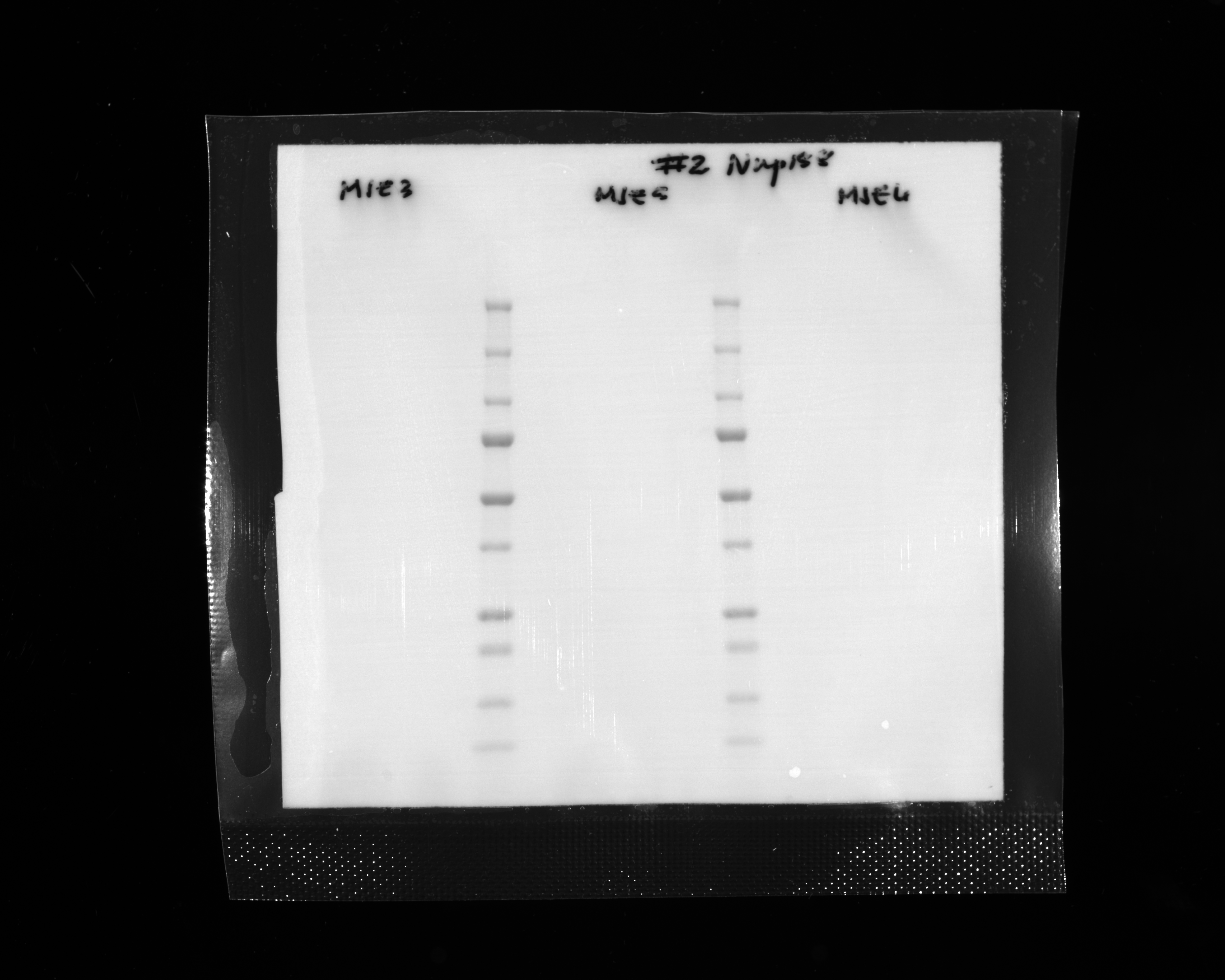

Supplement: Figure 1—source data 9. [file elife-108672-fig1-data9.zip › Fig 1C (part 3)/Nup188/AB_Nup188_Colorimetric imaging_from StainFree 12AUG #2_2Apro_MJE3_5 lysate.tif]

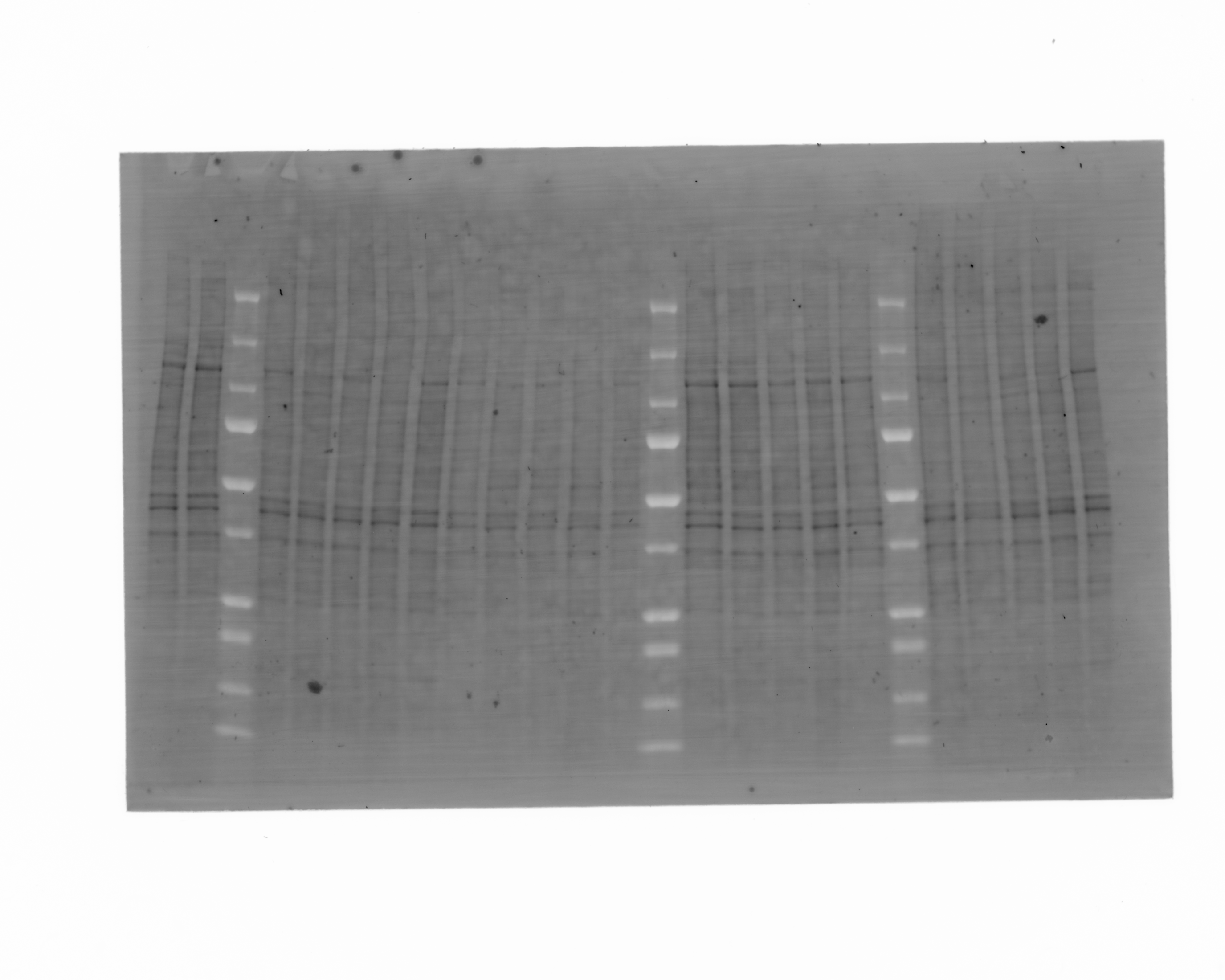

Supplement: Figure 1—source data 9. [file elife-108672-fig1-data9.zip › Fig 1C (part 3)/Nup188/AB_Stain free_12AUG_Blot #2_2Apro_AFTER transfer_MJE3_4_5 Lysates.tif]

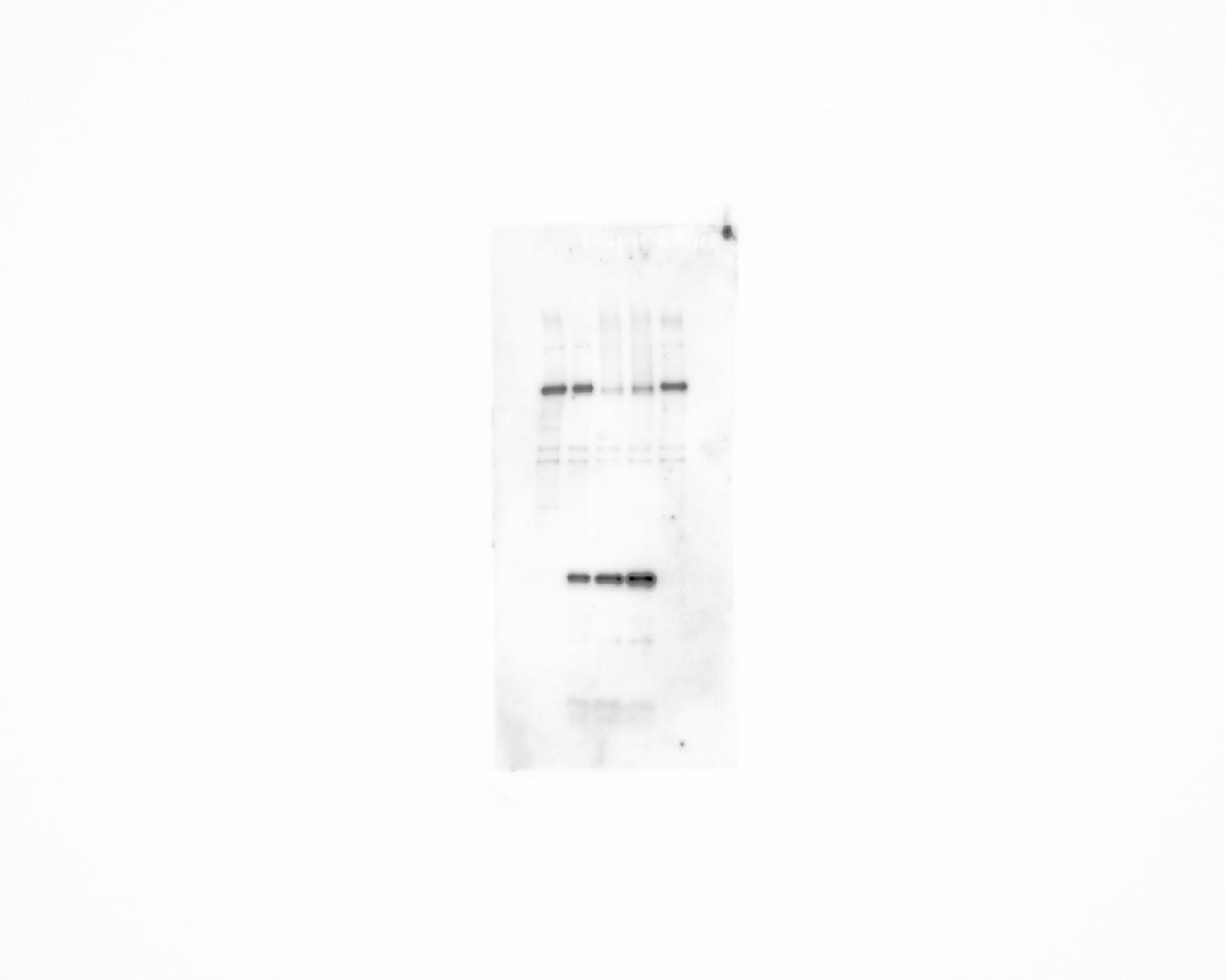

Supplement: Figure 1—source data 9. [file elife-108672-fig1-data9.zip › Fig 1C (part 3)/Nup188/C_Nup188_Chemi imaging_Stain Free 17SEP #1_2Apro_MM2 Lysate.tif]

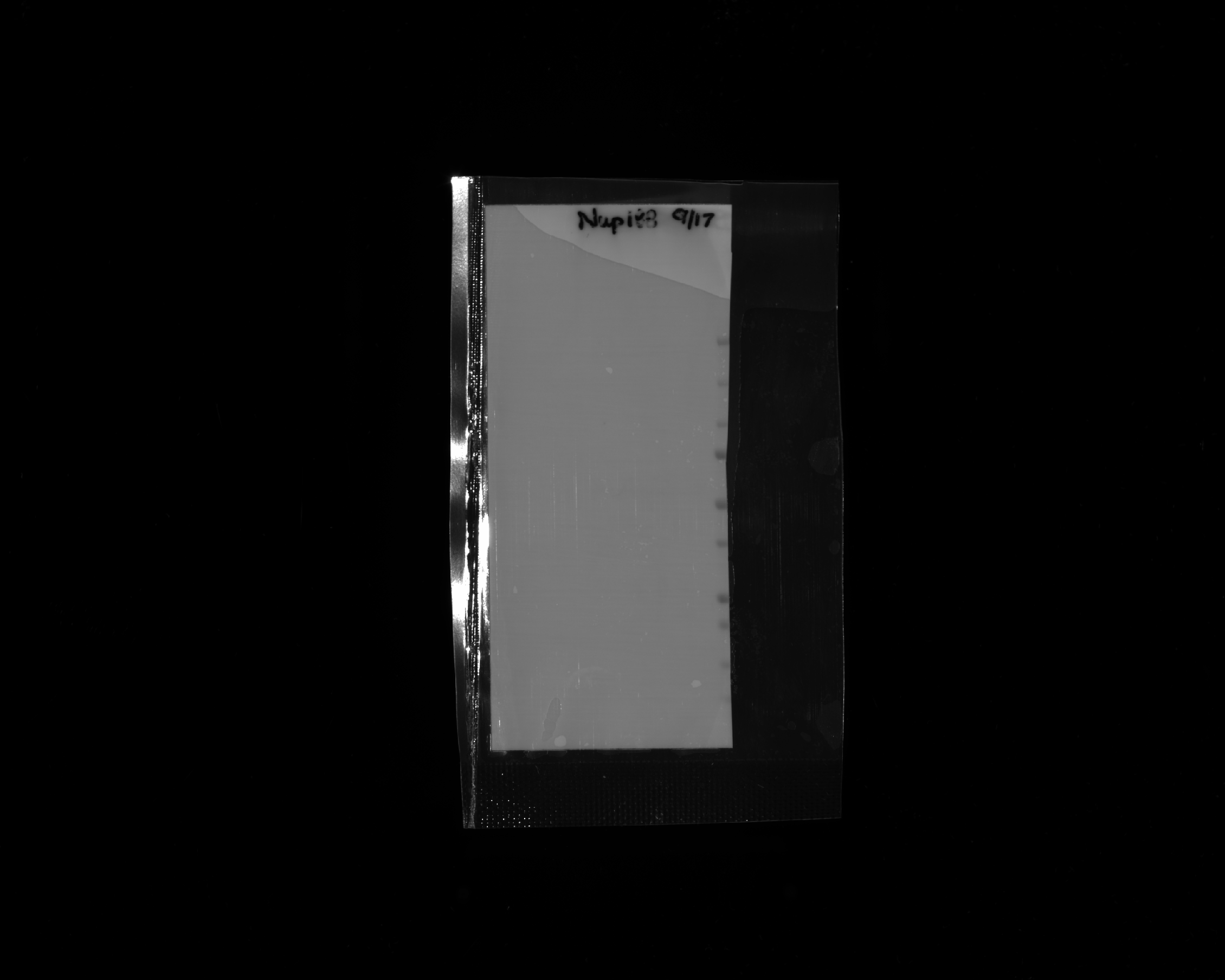

Supplement: Figure 1—source data 9. [file elife-108672-fig1-data9.zip › Fig 1C (part 3)/Nup188/C_Nup188_Colorimetric imaging_Stain Free 17SEP #1_2Apro_MM2 Lysate.tif]

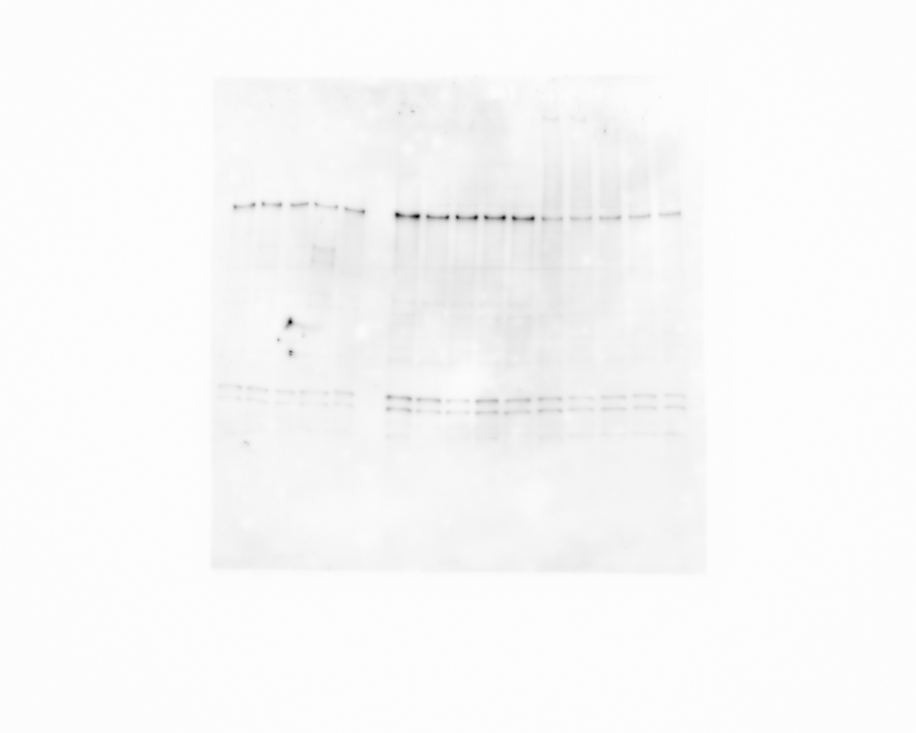

Supplement: Figure 1—source data 9. [file elife-108672-fig1-data9.zip › Fig 1C (part 3)/Nup214/Nup214_Chemi imaging_from StainFree 12AUG #1_2Apro_MJE3_5_6 Lysates.tif]

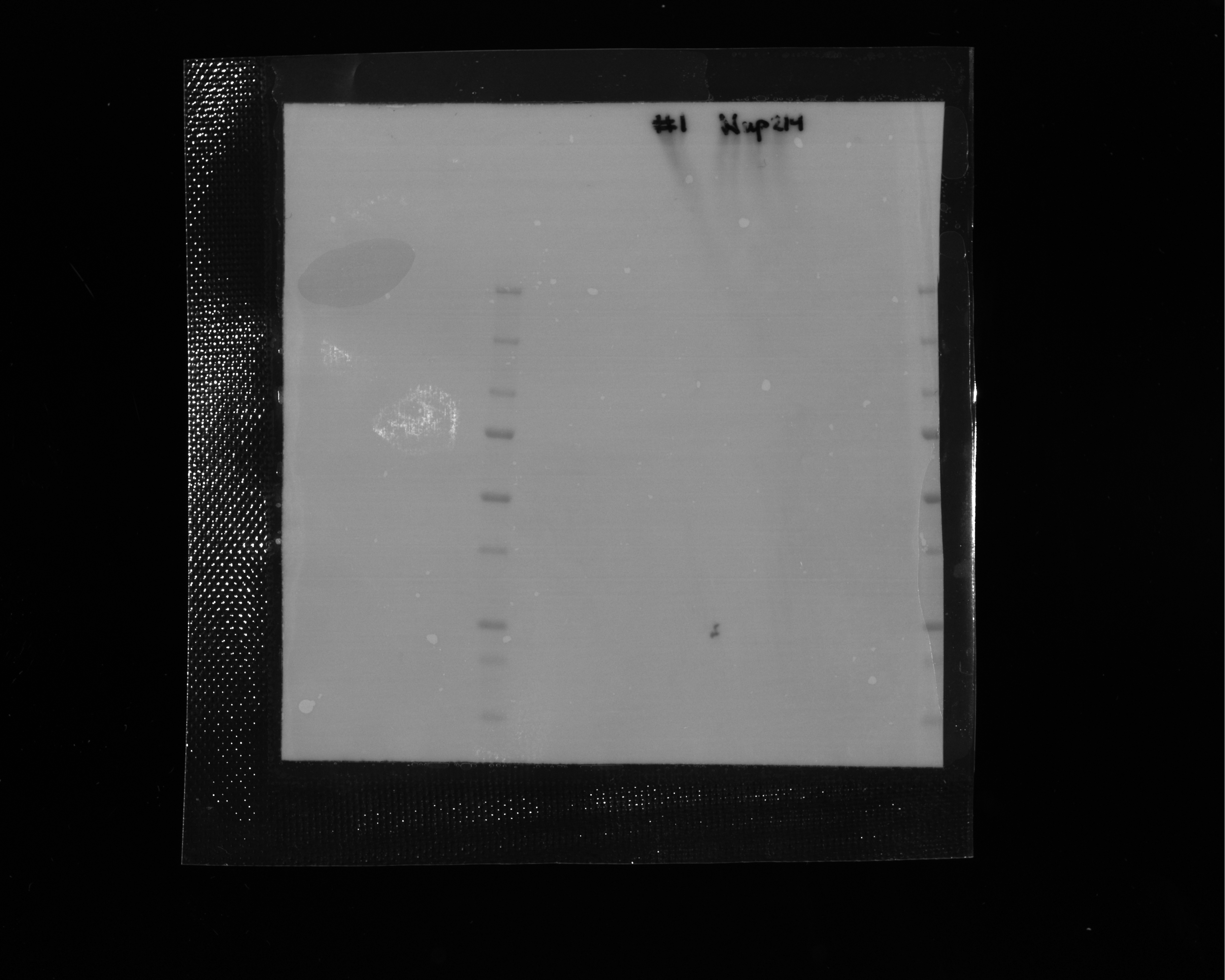

Supplement: Figure 1—source data 9. [file elife-108672-fig1-data9.zip › Fig 1C (part 3)/Nup214/Nup214_Colorimetric imaging_from StainFree 12AUG #1_2Apro_MJE3_5_6 Lysates.tif]

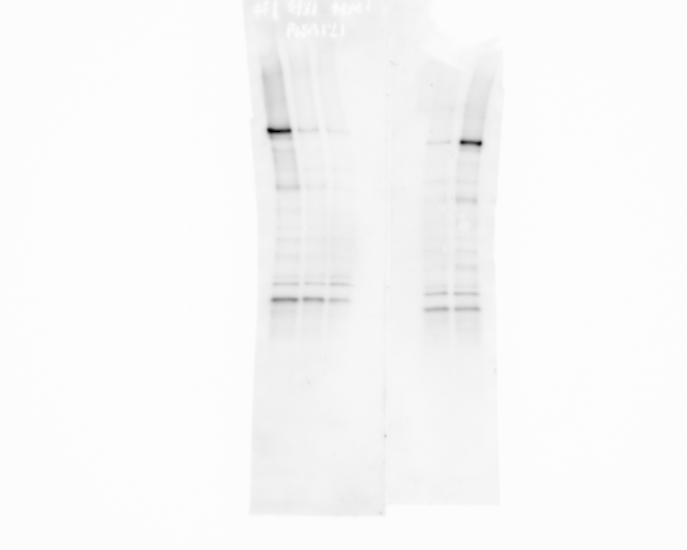

Supplement: Figure 1—source data 9. [file elife-108672-fig1-data9.zip › Fig 1C (part 3)/POM121/A_POM121_Chemi imaging_31AUG_from StainFree 27AUG #1_2Apro_MM1 Lysate.tif]

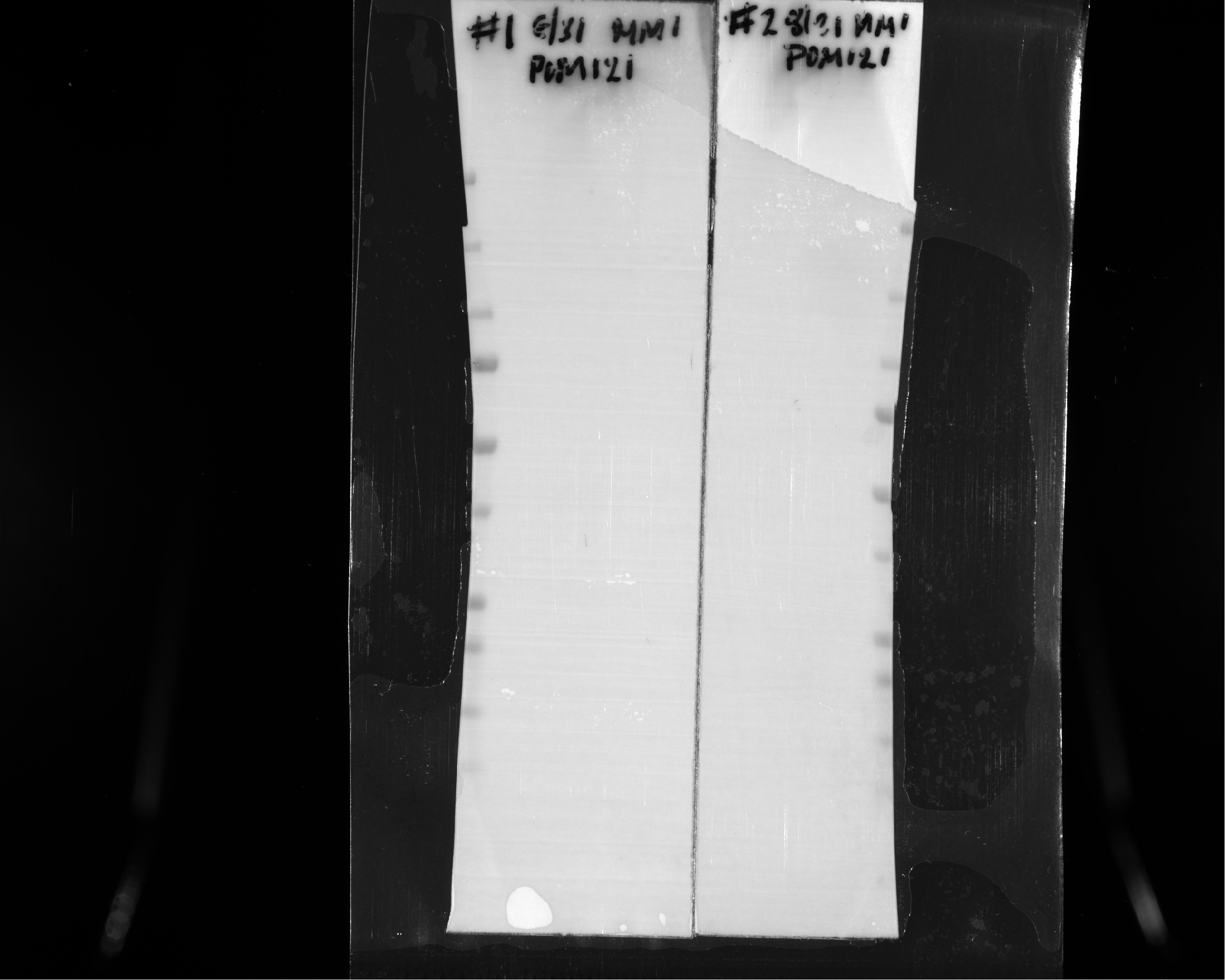

Supplement: Figure 1—source data 9. [file elife-108672-fig1-data9.zip › Fig 1C (part 3)/POM121/A_POM121_Colorimetric imaging_31AUG_from StainFree 27AUG #1_2Apro_MM1 Lysate.tif]

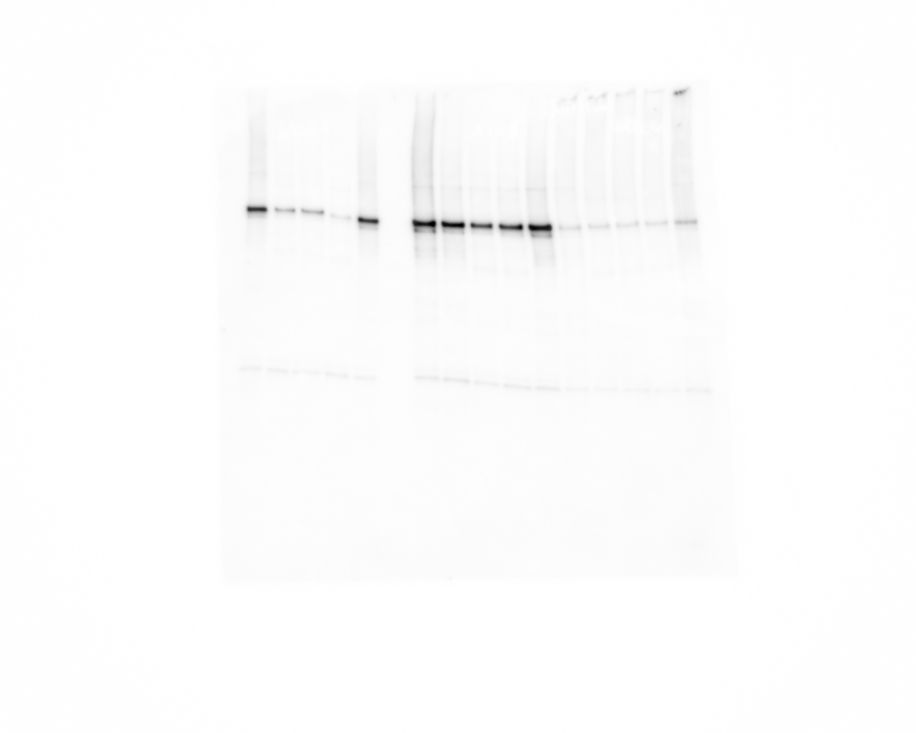

Supplement: Figure 1—source data 9. [file elife-108672-fig1-data9.zip › Fig 1C (part 3)/POM121/BC_POM121_Chemi imaging_from StainFree 19AUG #5_2Apro_MJE3_5 Lysates.tif]

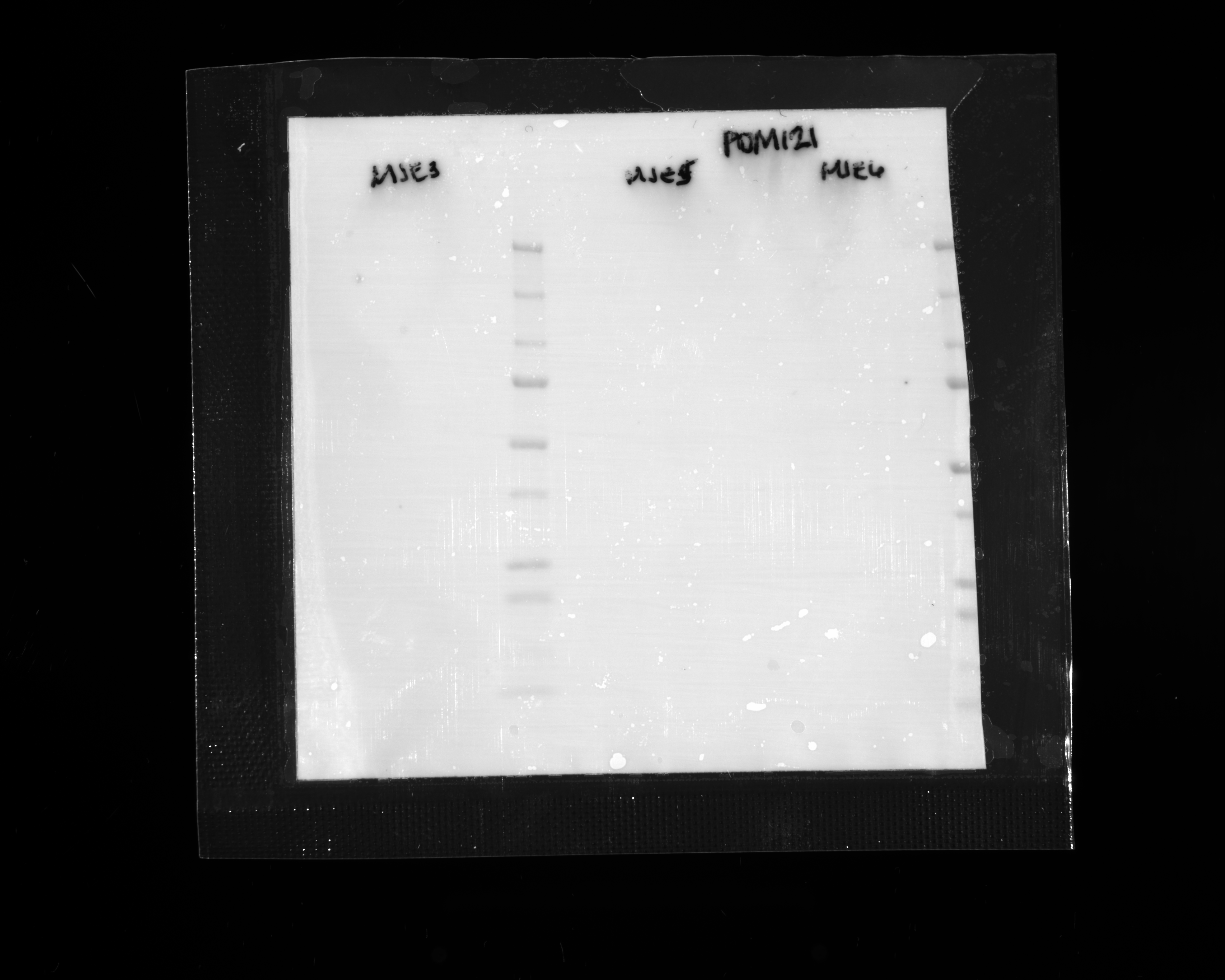

Supplement: Figure 1—source data 9. [file elife-108672-fig1-data9.zip › Fig 1C (part 3)/POM121/BC_POM121_Colorimetric imaging_from StainFree 19AUG #5_2Apro_MJE3_5 Lysates.tif]

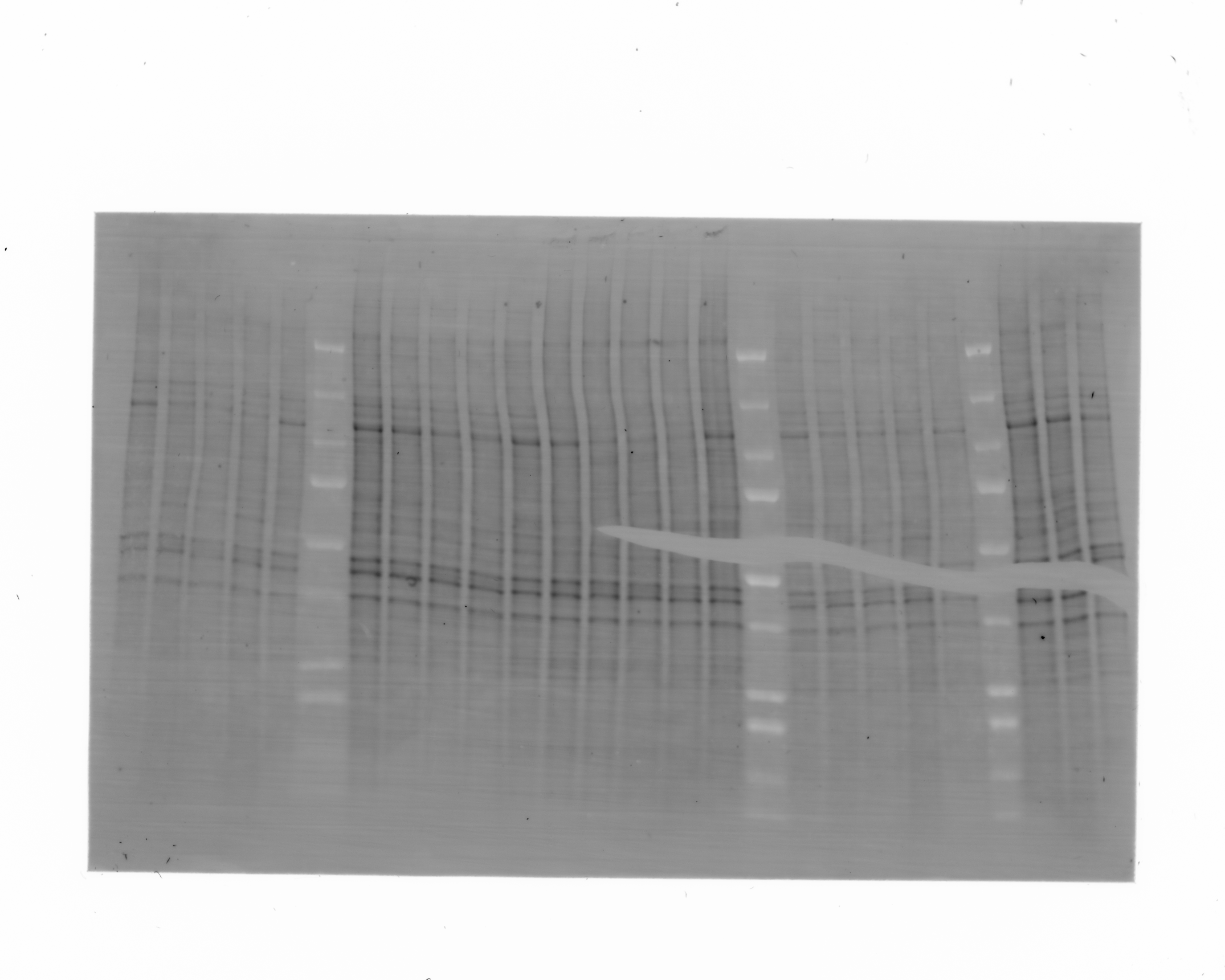

Supplement: Figure 1—source data 9. [file elife-108672-fig1-data9.zip › Fig 1C (part 3)/POM121/BC_Stain Free_19AUG24_Blot #5_2Apro_AFTER transfer_MJE3_5_6 Lysates.tif]

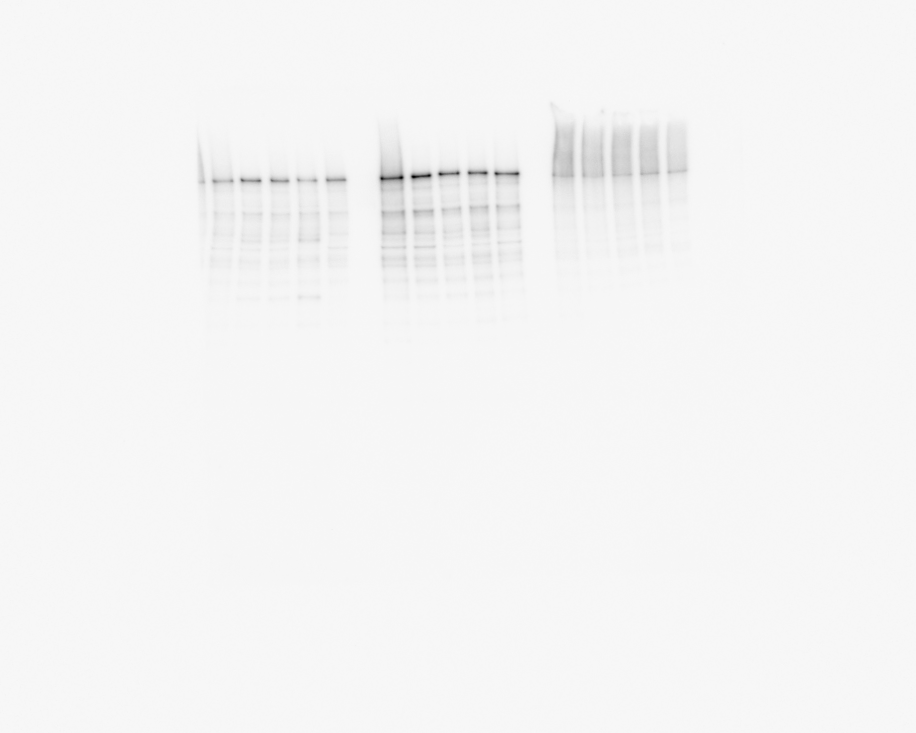

Supplement: Figure 1—source data 10. [file elife-108672-fig1-data10.zip › Fig 1C (part 4)/RanBP2/AB_RanBP2_Chemi imaging_22AUG_from StainFree 12AUG #2_2Apro_MJE3_5 Lysates.tif]

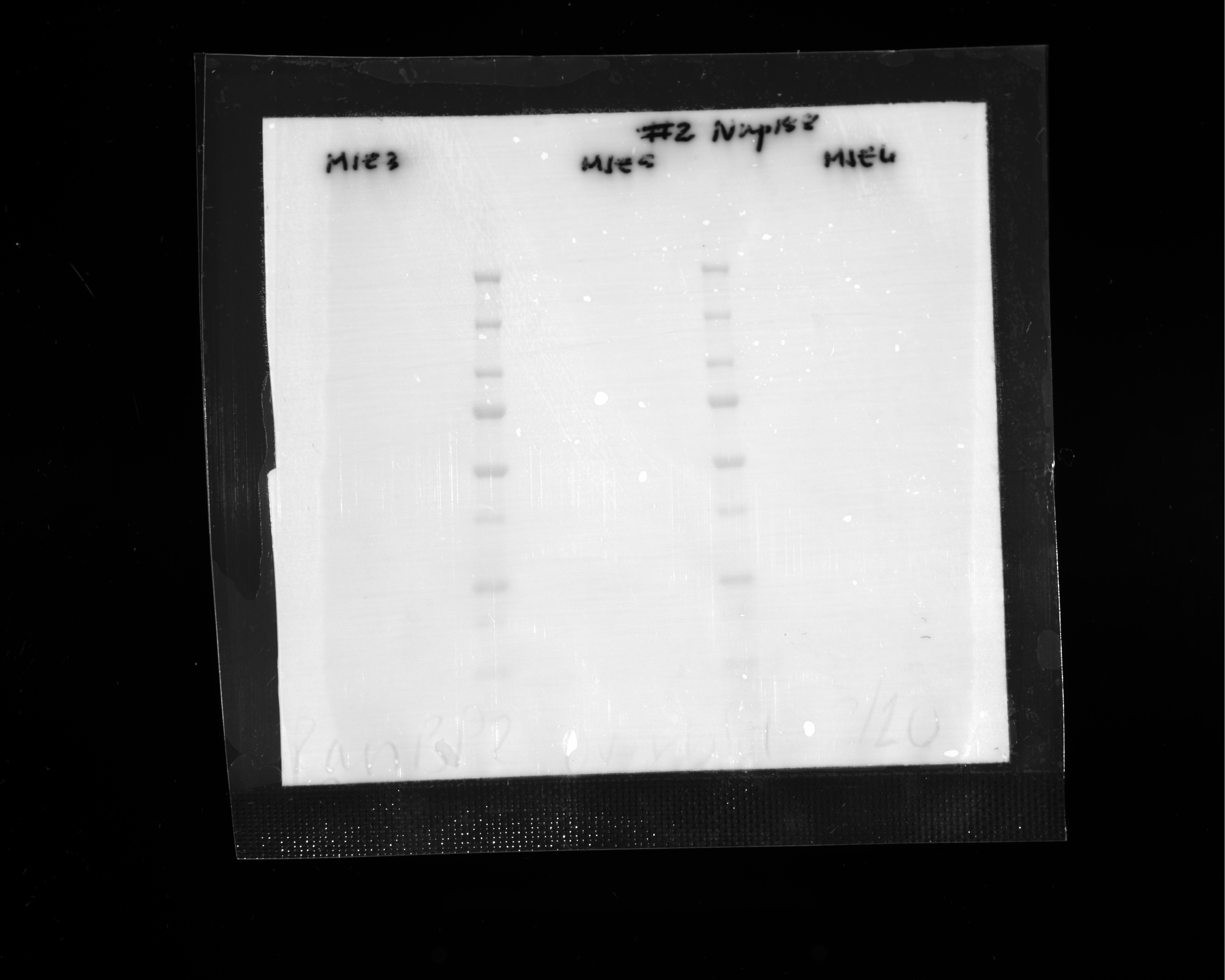

Supplement: Figure 1—source data 10. [file elife-108672-fig1-data10.zip › Fig 1C (part 4)/RanBP2/AB_RanBP2_Colorimetric imaging_22AUG_from StainFree 12AUG #2_2Apro_MJE3_5 Lysates.tif]

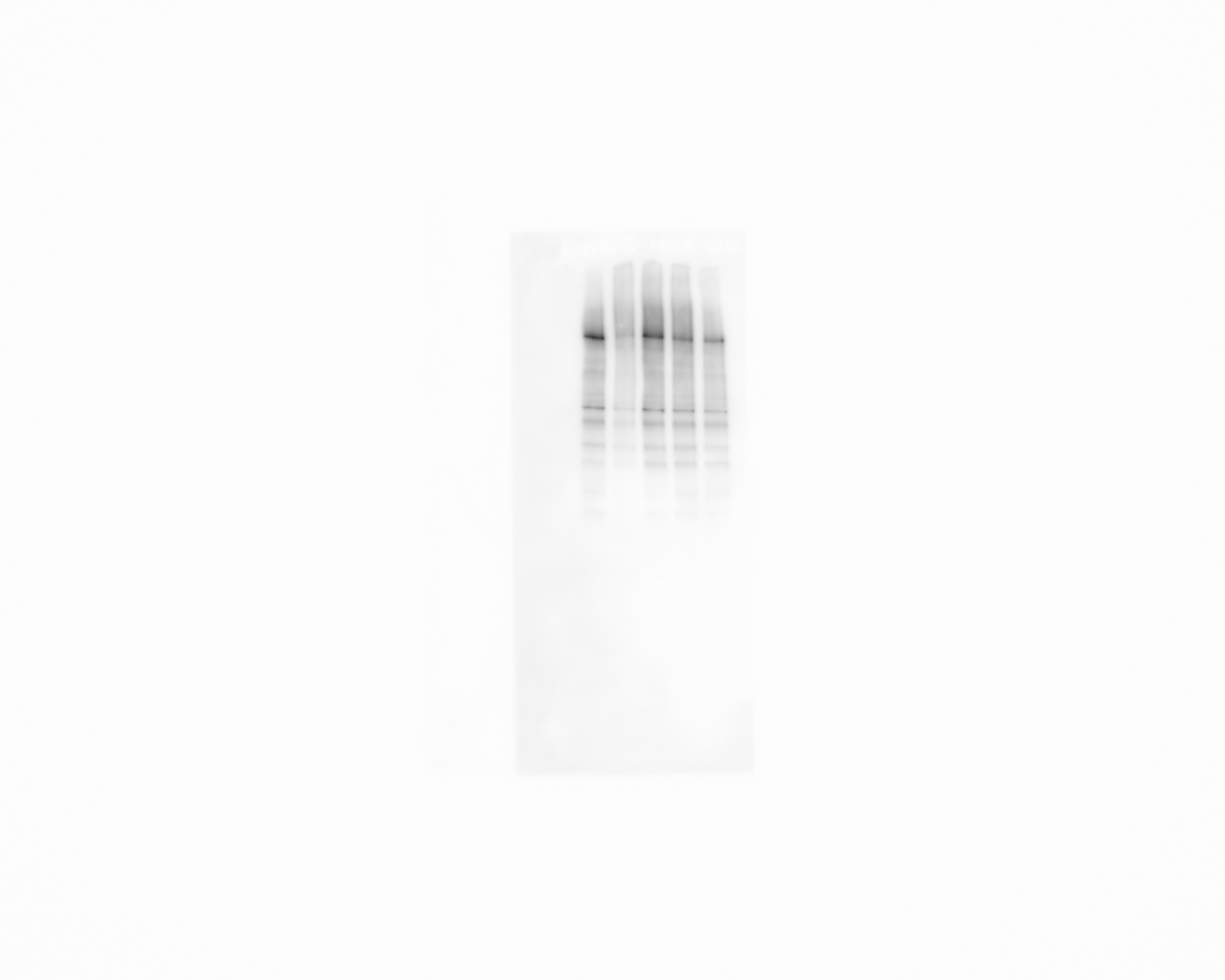

Supplement: Figure 1—source data 10. [file elife-108672-fig1-data10.zip › Fig 1C (part 4)/RanBP2/C_RanBP2_Chemi imaging_25SEP_from StainFree 25AUG #1_2Apro_MM2 Lysate.tif]

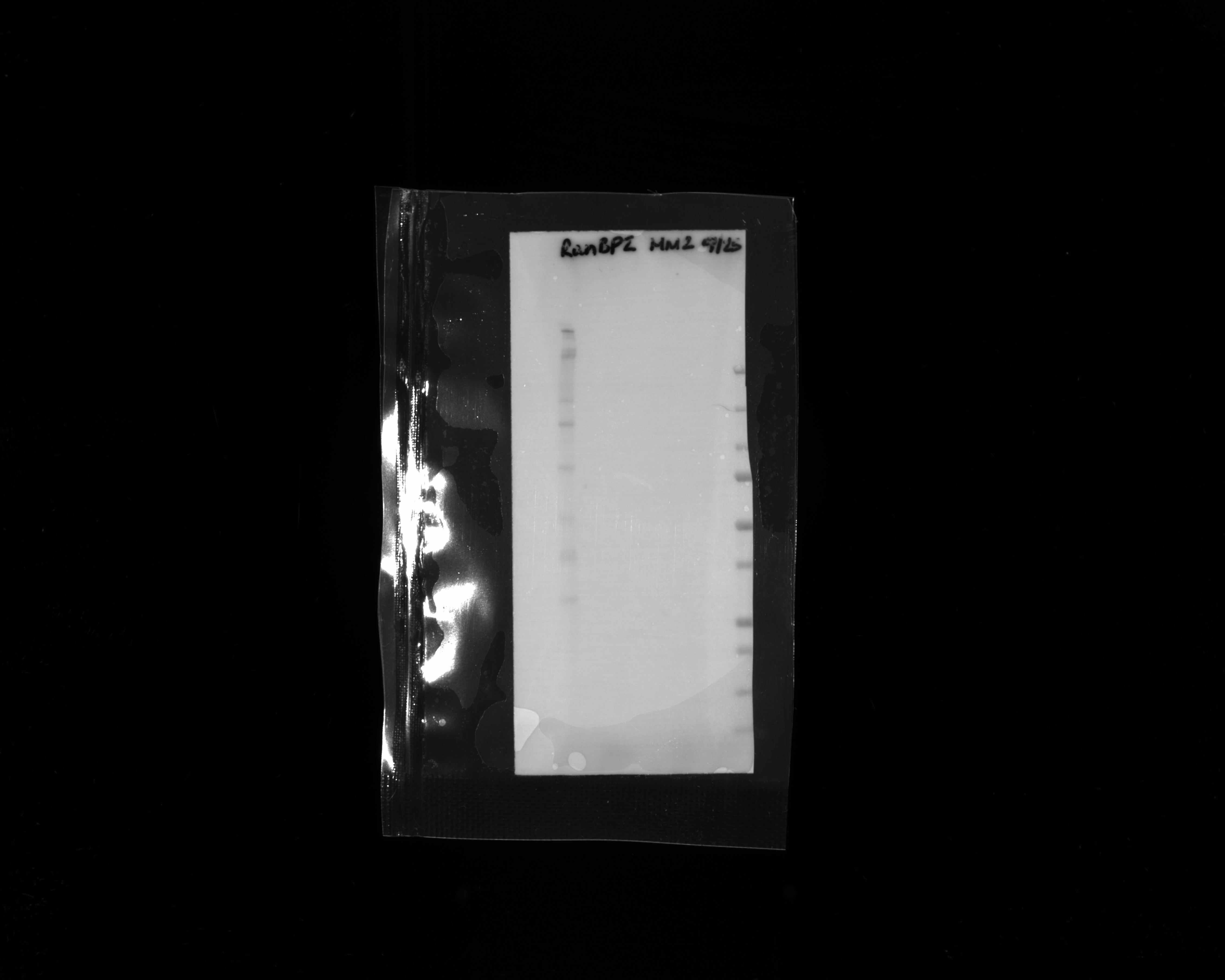

Supplement: Figure 1—source data 10. [file elife-108672-fig1-data10.zip › Fig 1C (part 4)/RanBP2/C_RanBP2_Colorimetric imaging_25SEP_from StainFree 25AUG #1_2Apro_MM2 Lysate.tif]

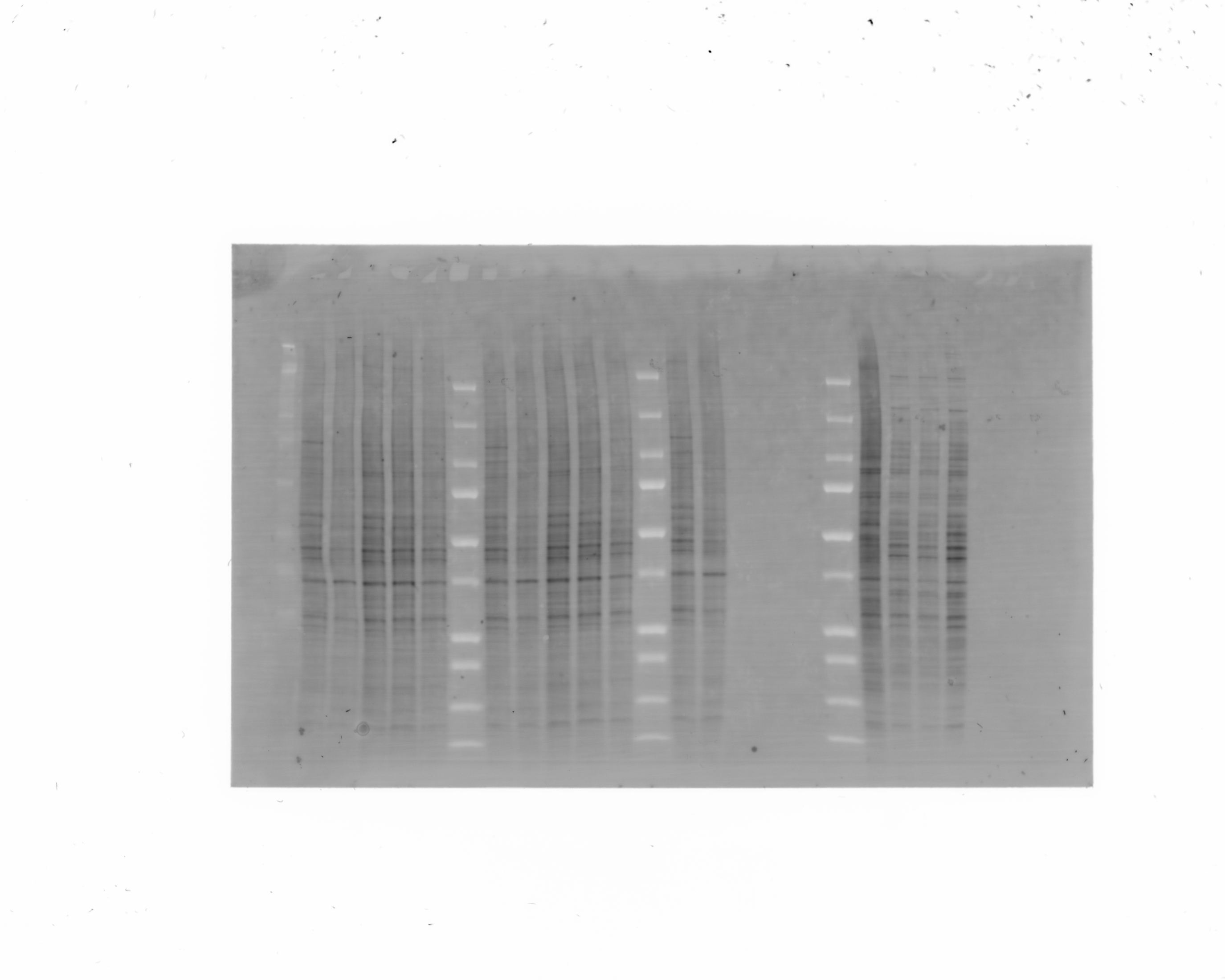

Supplement: Figure 1—source data 10. [file elife-108672-fig1-data10.zip › Fig 1C (part 4)/RanBP2/C_Stain Free_25SEP_Blot #1_2Apro_AFTER transfer_MM2 lysates.tif]

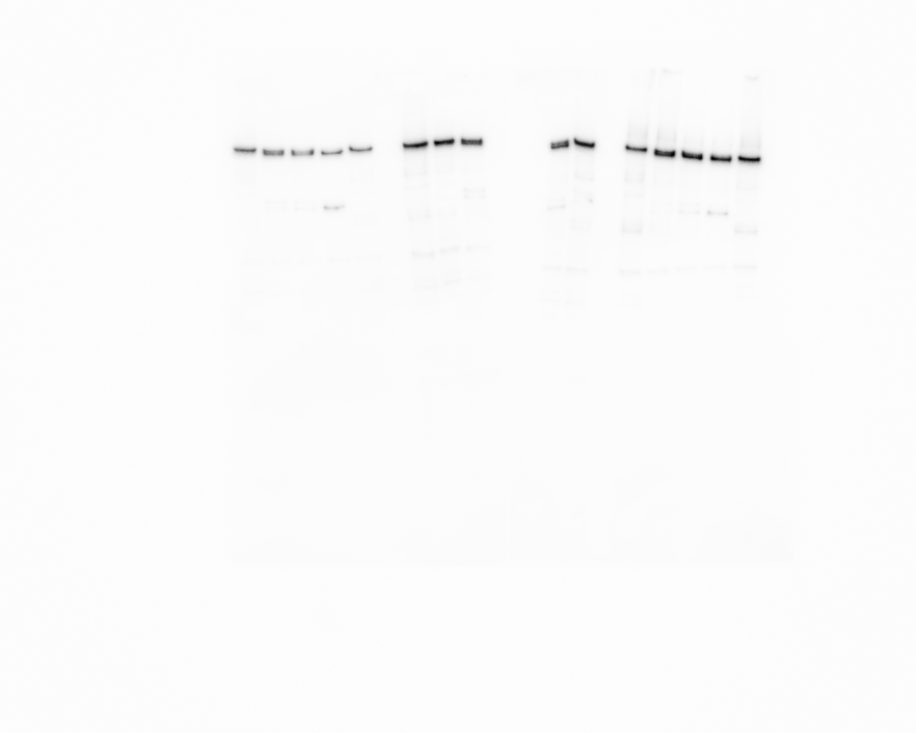

Supplement: Figure 1—source data 10. [file elife-108672-fig1-data10.zip › Fig 1C (part 4)/TPR/TPR_Chemi imaging_from StainFree 19AUG #5 and #6_2Apro_MJE3_5_6 Lysates.tif]

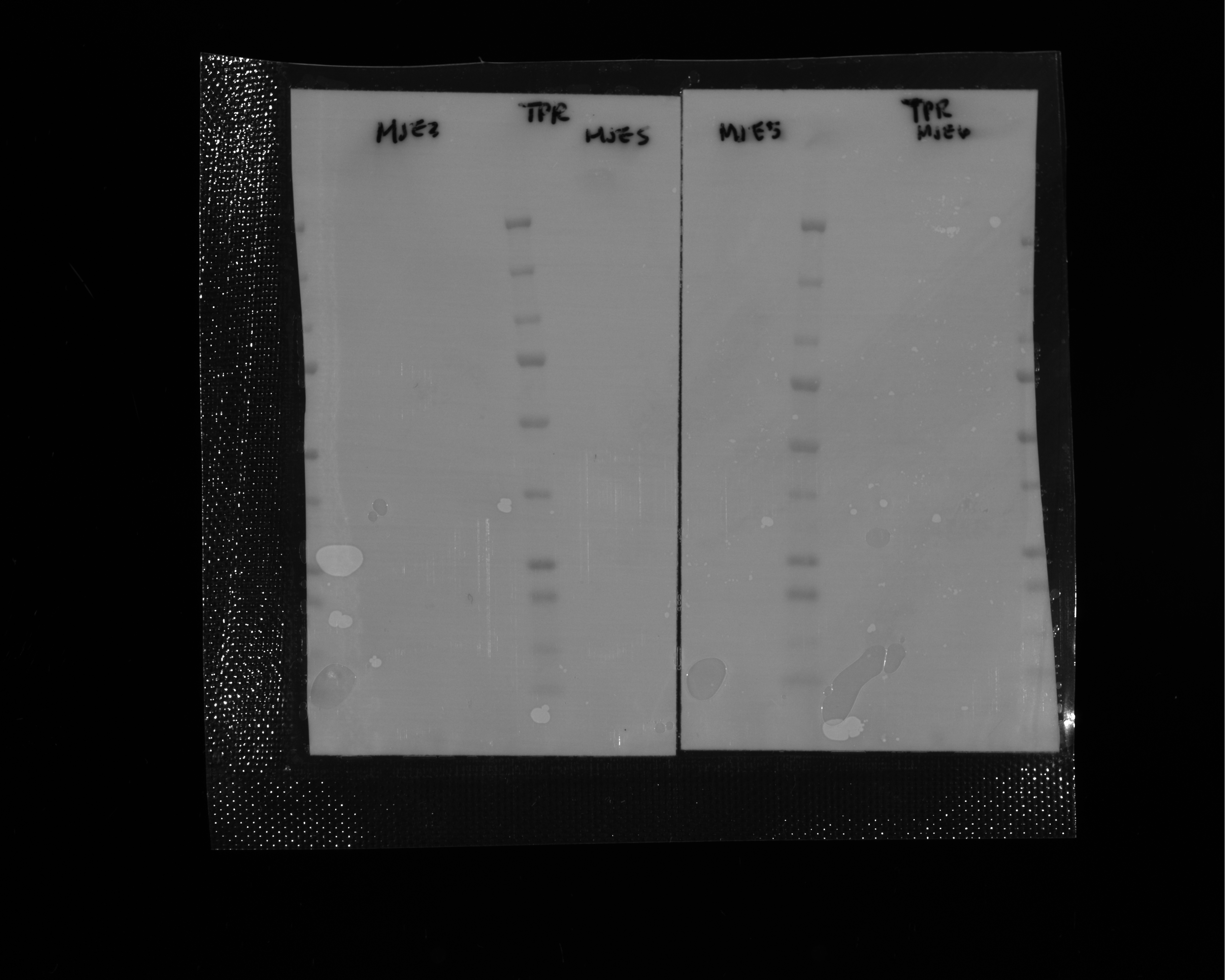

Supplement: Figure 1—source data 10. [file elife-108672-fig1-data10.zip › Fig 1C (part 4)/TPR/TPR_Colorimetric imaging_from StainFree 19AUG #5 and #6_2Apro_MJE3_5_6 Lysates.tif]

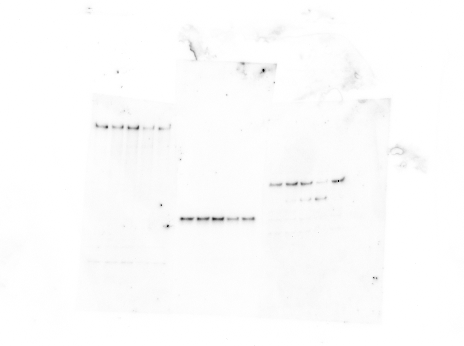

Supplement: Figure 1—source data 11. [file elife-108672-fig1-data11.zip › Fig 1D (part 1)/NDC1/A_NDC1 Blot 1 (3rd set).tif]

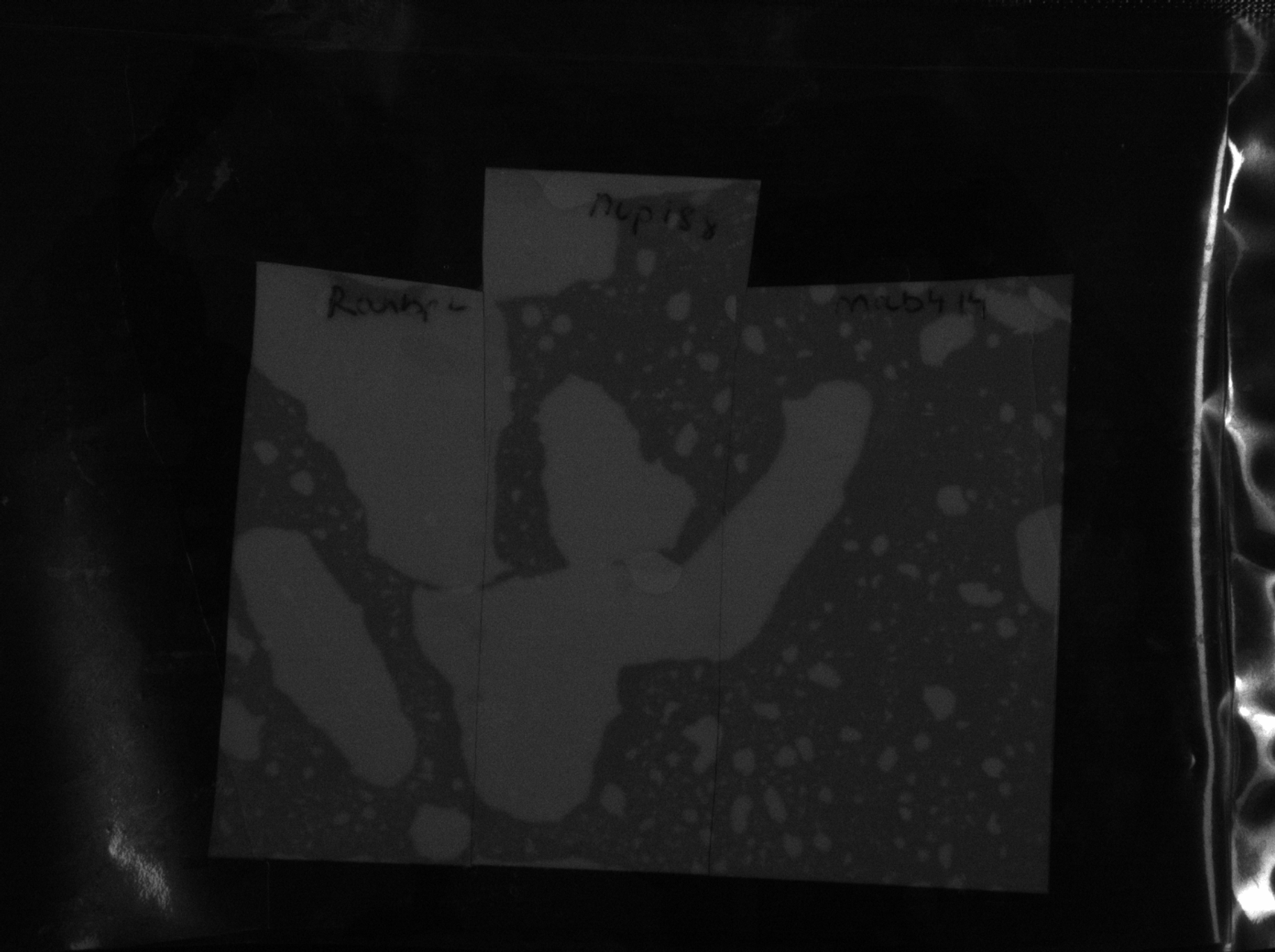

Supplement: Figure 1—source data 11. [file elife-108672-fig1-data11.zip › Fig 1D (part 1)/NDC1/A_NDC1 Ladder 1 (3rd set) - poor image.tif]

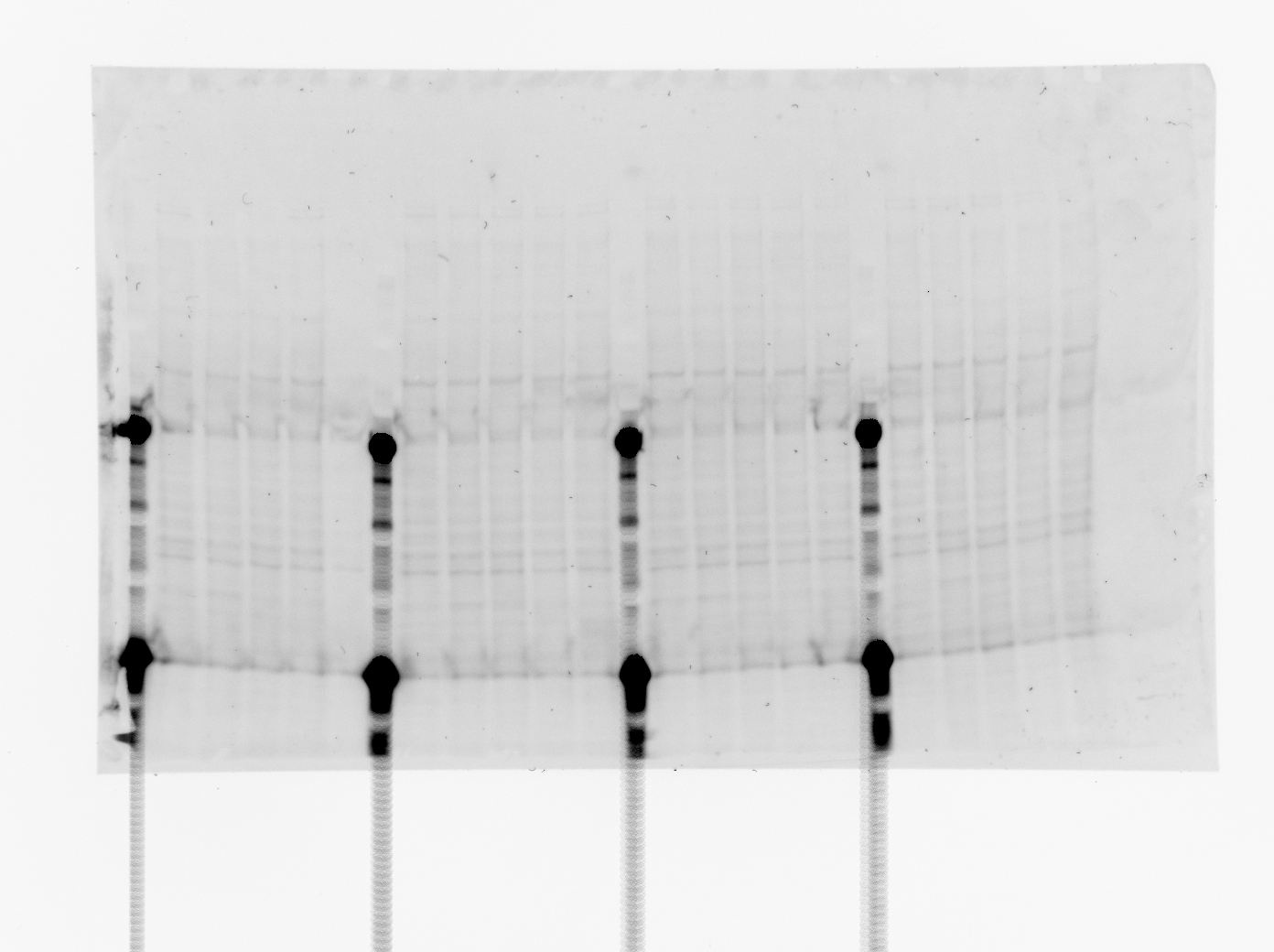

Supplement: Figure 1—source data 11. [file elife-108672-fig1-data11.zip › Fig 1D (part 1)/NDC1/A_NDC1 Loading Control 1 (4th set).tif]

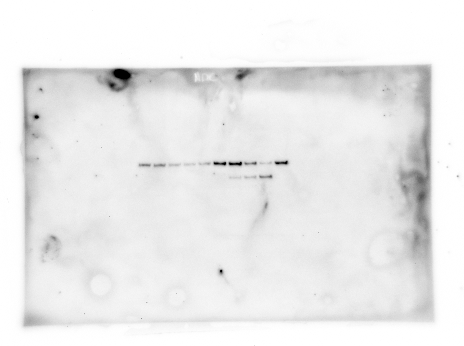

Supplement: Figure 1—source data 11. [file elife-108672-fig1-data11.zip › Fig 1D (part 1)/NDC1/B_NDC1 Blot 3 (2nd set).tif]

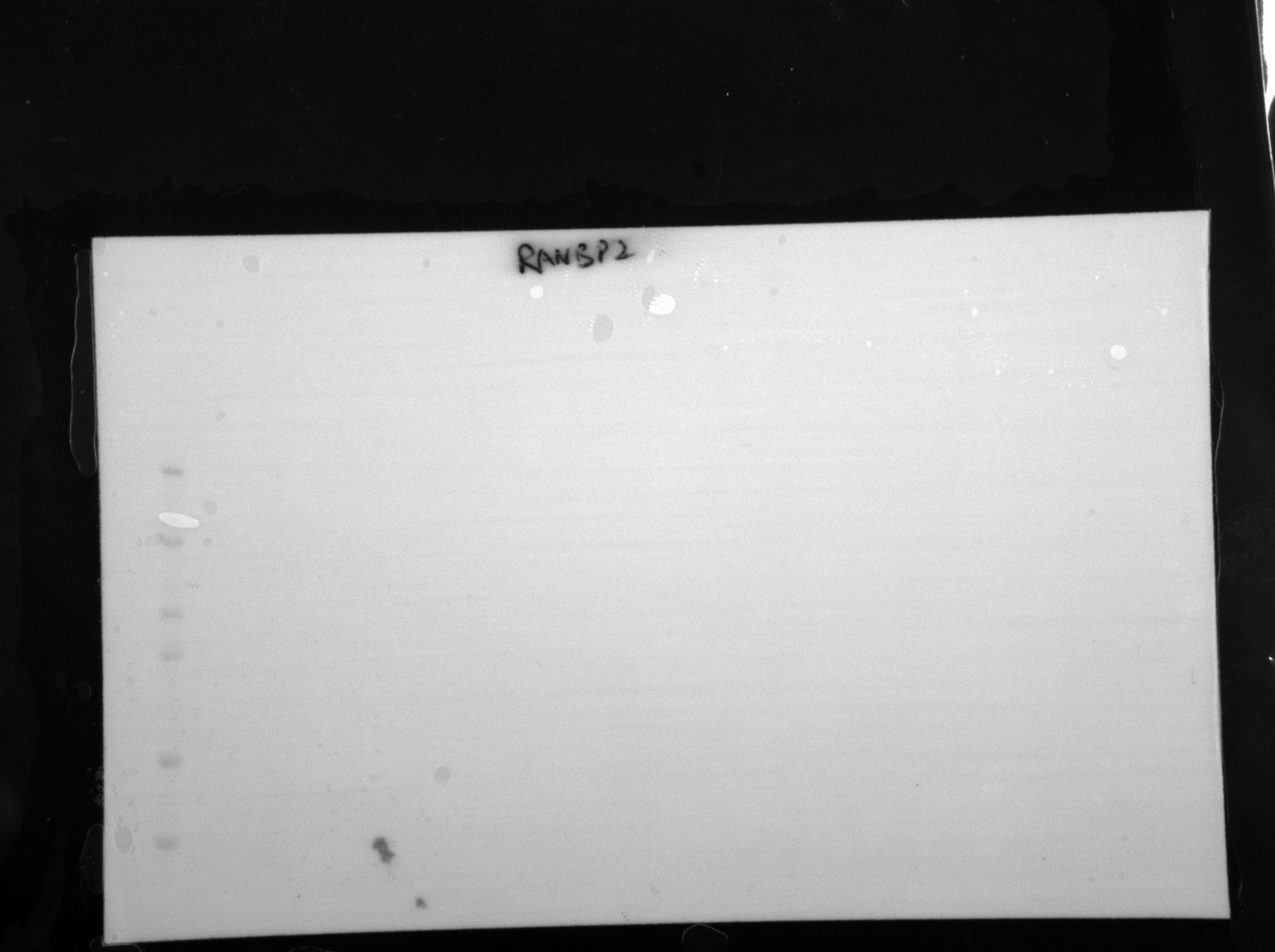

Supplement: Figure 1—source data 11. [file elife-108672-fig1-data11.zip › Fig 1D (part 1)/NDC1/B_NDC1 Ladder 3.tif]

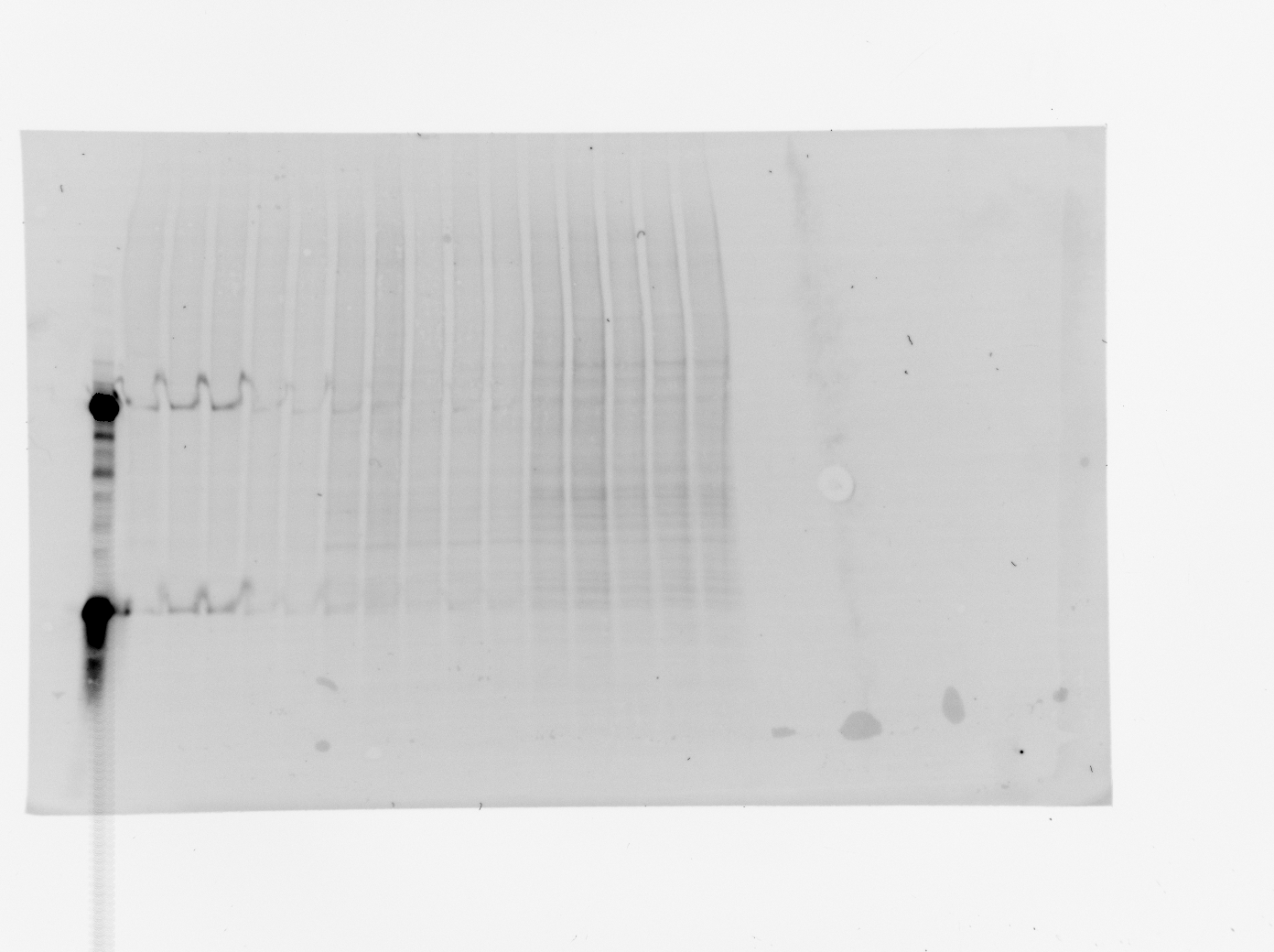

Supplement: Figure 1—source data 11. [file elife-108672-fig1-data11.zip › Fig 1D (part 1)/NDC1/B_NDC1 Loading control 3 (3rd group).tif]

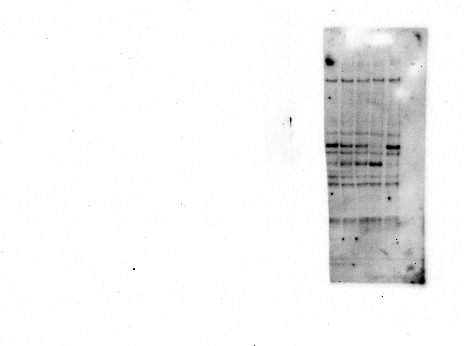

Supplement: Figure 1—source data 11. [file elife-108672-fig1-data11.zip › Fig 1D (part 1)/NDC1/C_26DEC23 3Cpro_blot CHEMI NDC1 200s Quantifed MJE2.tif]

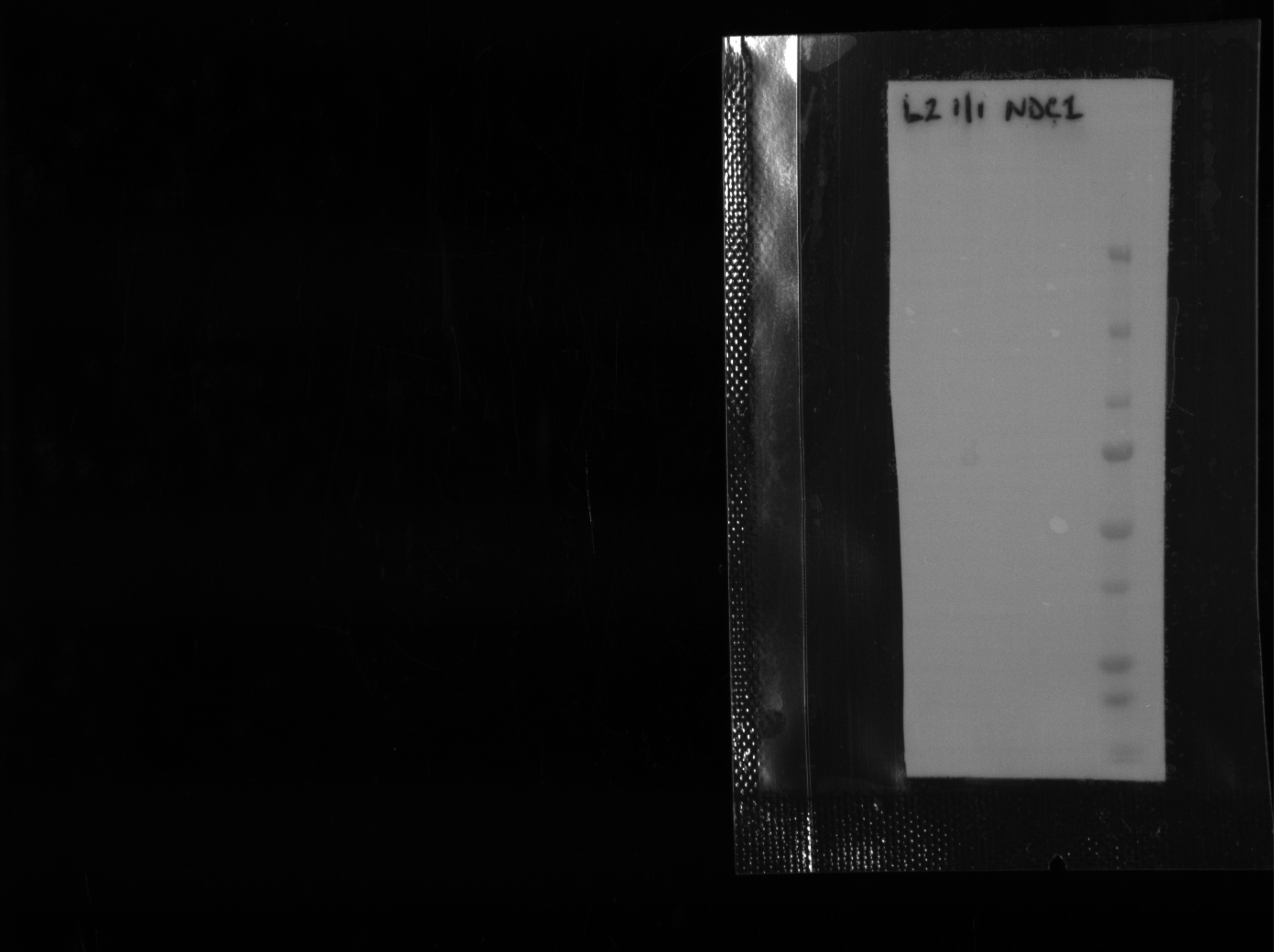

Supplement: Figure 1—source data 11. [file elife-108672-fig1-data11.zip › Fig 1D (part 1)/NDC1/C_26DEC23 3Cpro_blot COLORIMETRIC NDC1 0.1s MJE2.tif]

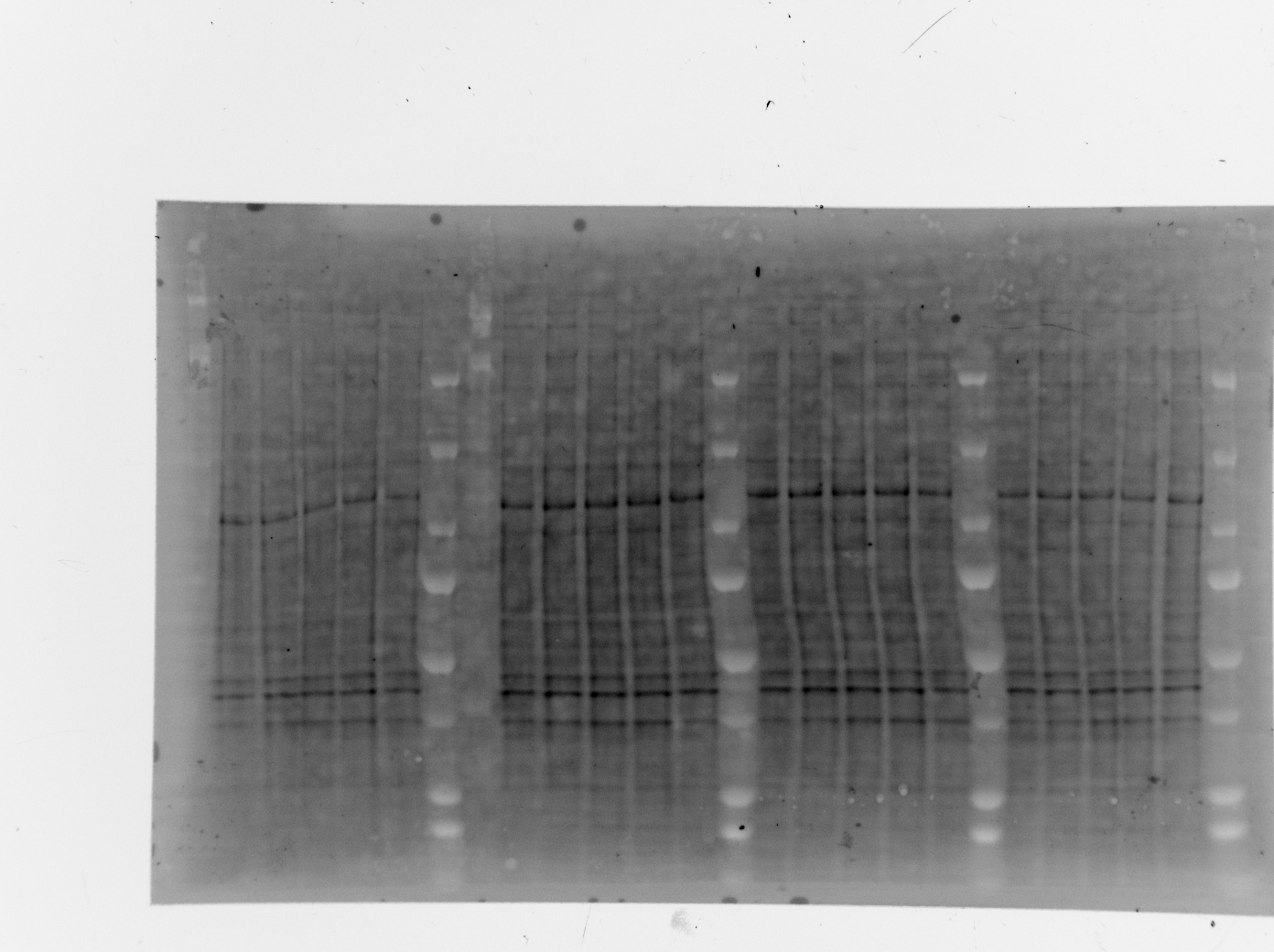

Supplement: Figure 1—source data 11. [file elife-108672-fig1-data11.zip › Fig 1D (part 1)/NDC1/C_26DEC23 3Cpro_blot stain free BLOT (RanBP2, Nup214, Nup50, NDC1) 1.3s MJE2.tif]

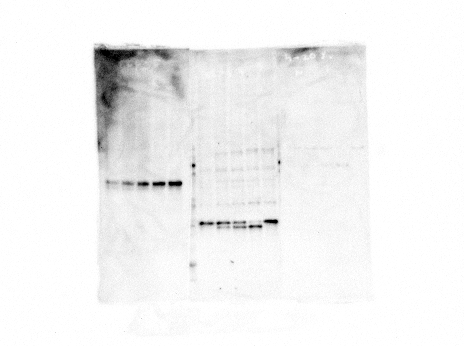

Supplement: Figure 1—source data 11. [file elife-108672-fig1-data11.zip › Fig 1D (part 1)/Nup35/Nup35 Blot 1 (set 2).tif]

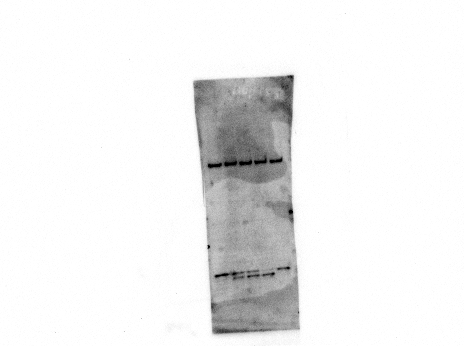

Supplement: Figure 1—source data 11. [file elife-108672-fig1-data11.zip › Fig 1D (part 1)/Nup35/Nup35 Blot 2.tif]

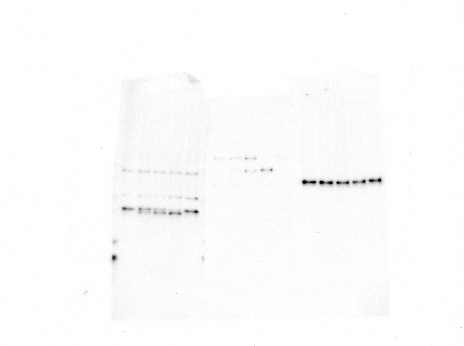

Supplement: Figure 1—source data 11. [file elife-108672-fig1-data11.zip › Fig 1D (part 1)/Nup35/Nup35 Blot 3 (set 1).tif]

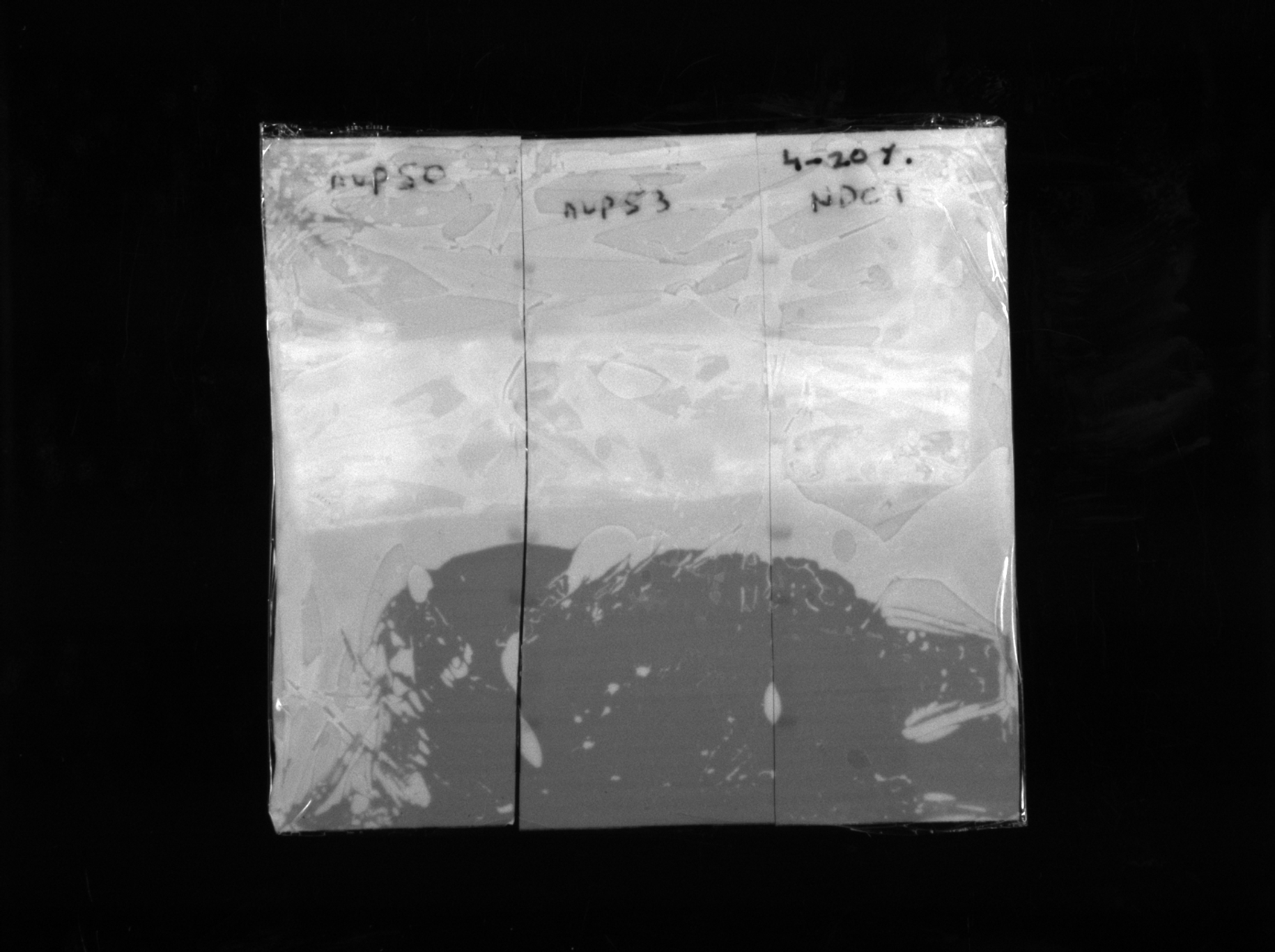

Supplement: Figure 1—source data 11. [file elife-108672-fig1-data11.zip › Fig 1D (part 1)/Nup35/Nup35 Ladder 1 (set 2).tif]

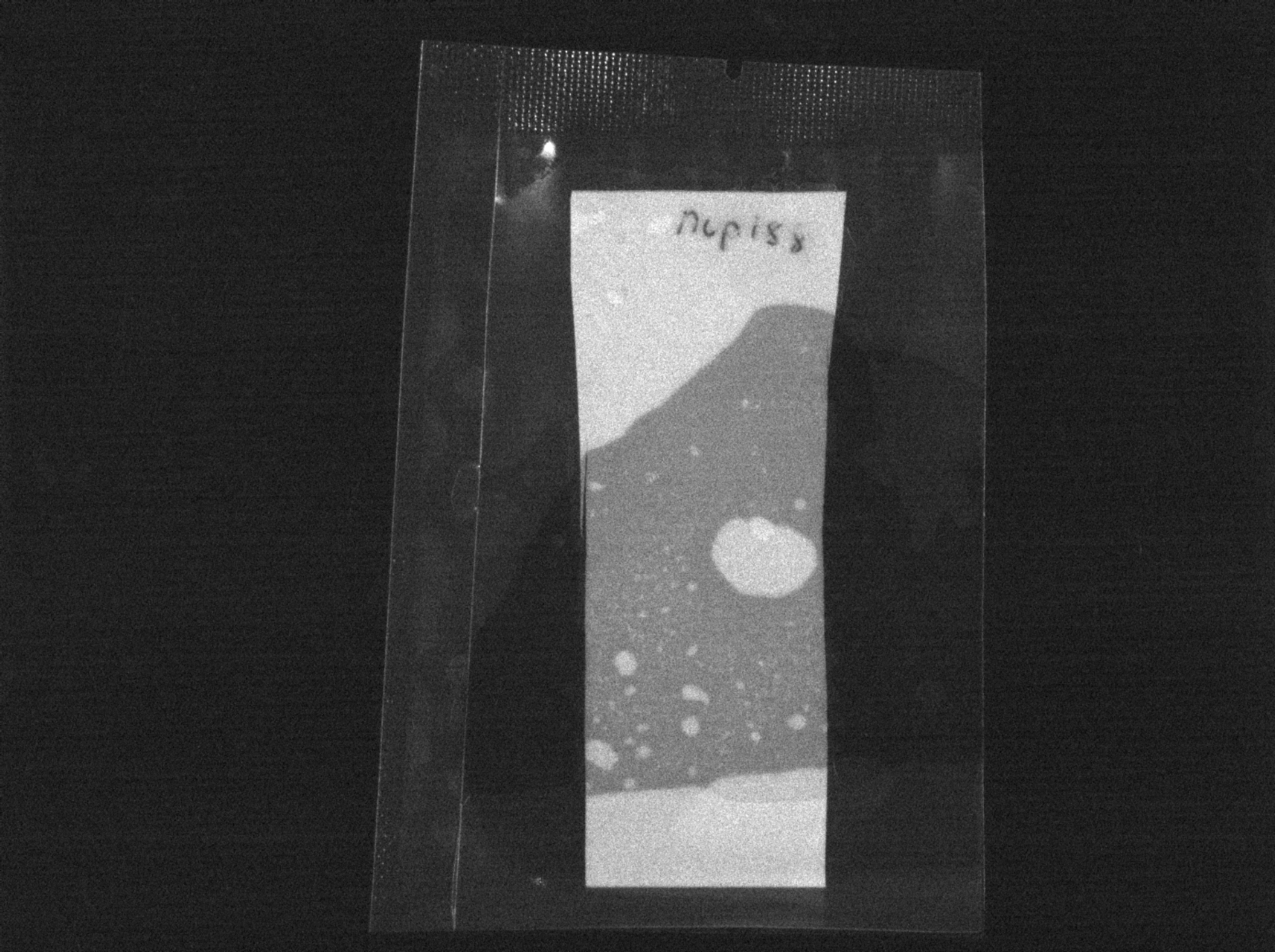

Supplement: Figure 1—source data 11. [file elife-108672-fig1-data11.zip › Fig 1D (part 1)/Nup35/Nup35 Ladder 2 (poor image).tif]

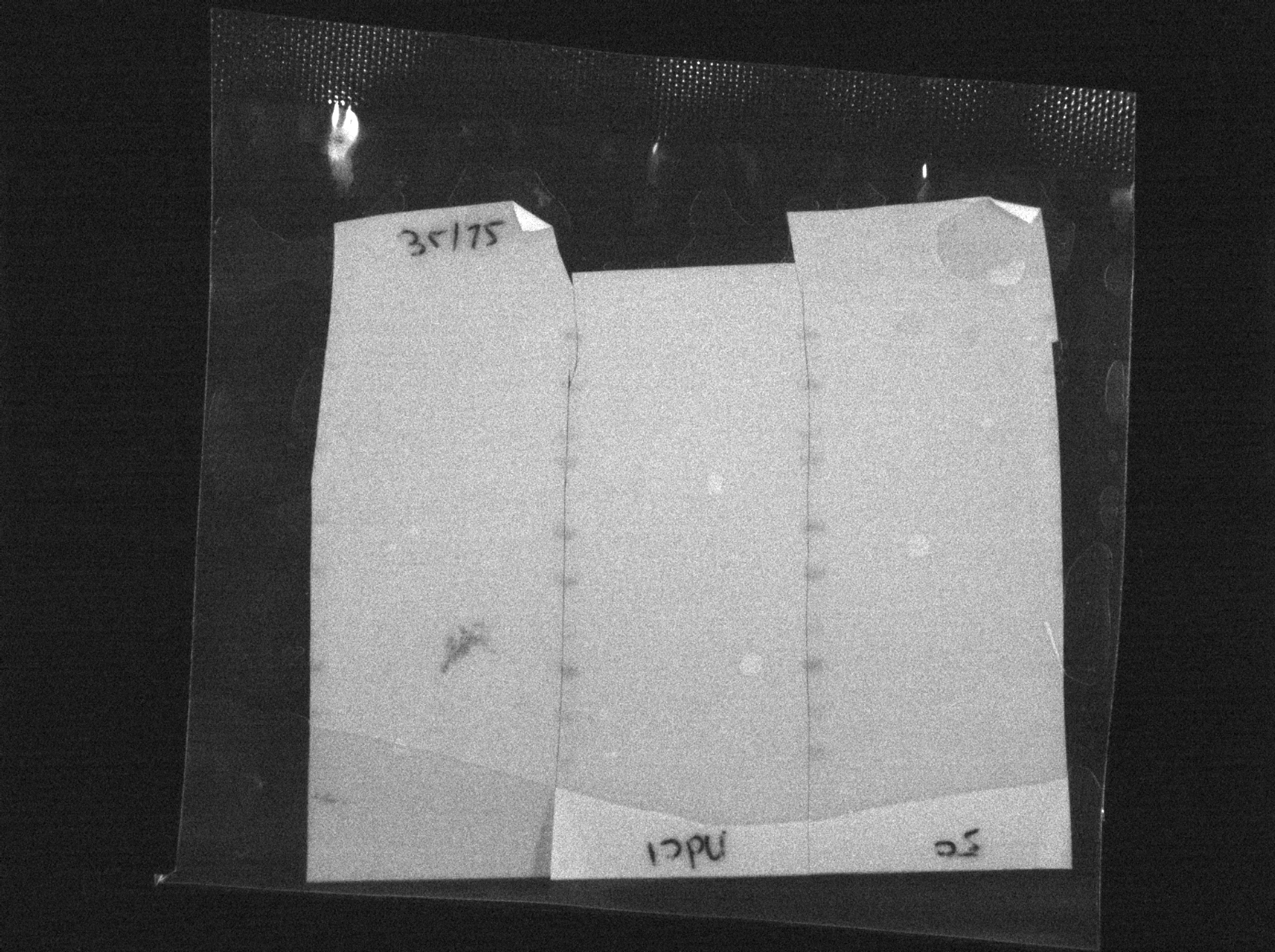

Supplement: Figure 1—source data 11. [file elife-108672-fig1-data11.zip › Fig 1D (part 1)/Nup35/Nup35 Ladder 3 (set 1).tif]

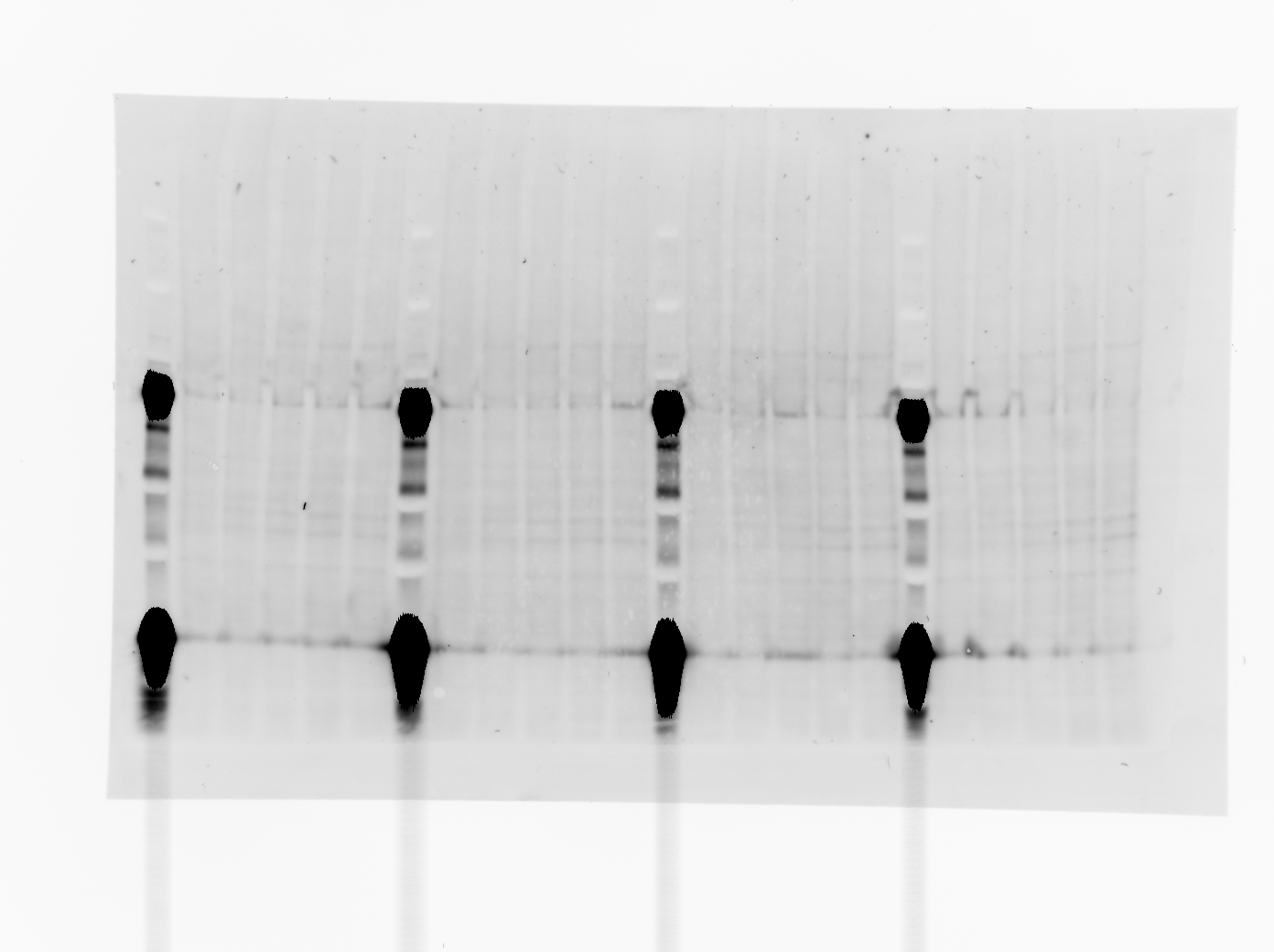

Supplement: Figure 1—source data 11. [file elife-108672-fig1-data11.zip › Fig 1D (part 1)/Nup35/Nup35 Loading control 1 (set 2).tif]

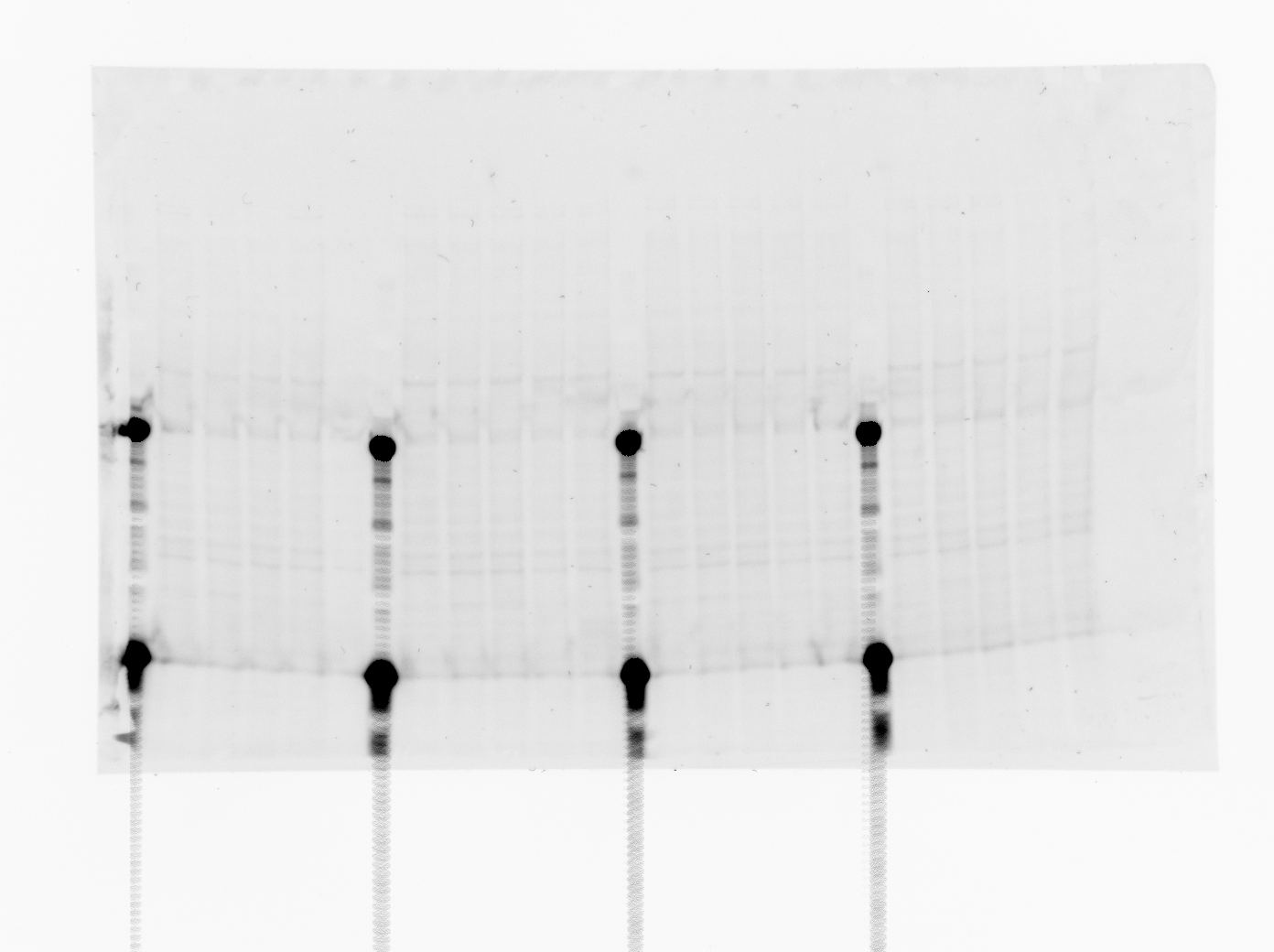

Supplement: Figure 1—source data 11. [file elife-108672-fig1-data11.zip › Fig 1D (part 1)/Nup35/Nup35 Loading control 2 (set 1).tif]

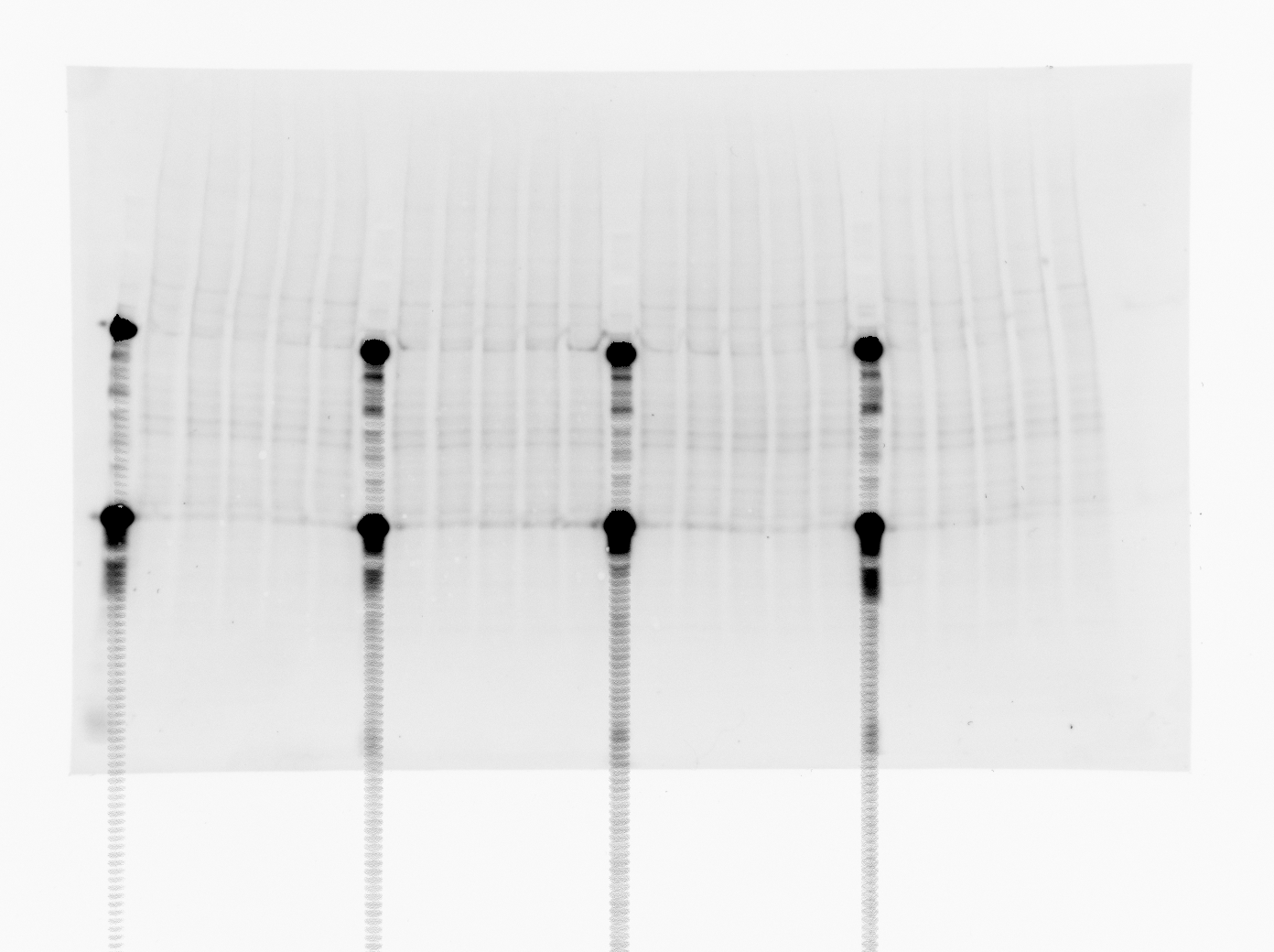

Supplement: Figure 1—source data 11. [file elife-108672-fig1-data11.zip › Fig 1D (part 1)/Nup35/Nup35 Loading control 3 (set 1).tif]

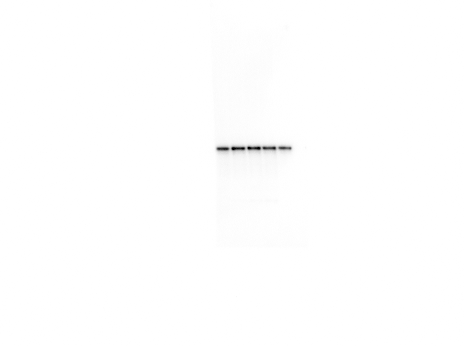

Supplement: Figure 1—source data 11. [file elife-108672-fig1-data11.zip › Fig 1D (part 1)/Nup50/A_26DEC23 3Cpro_blot CHEMI Nup50 2s Quantified MJE2.tif]

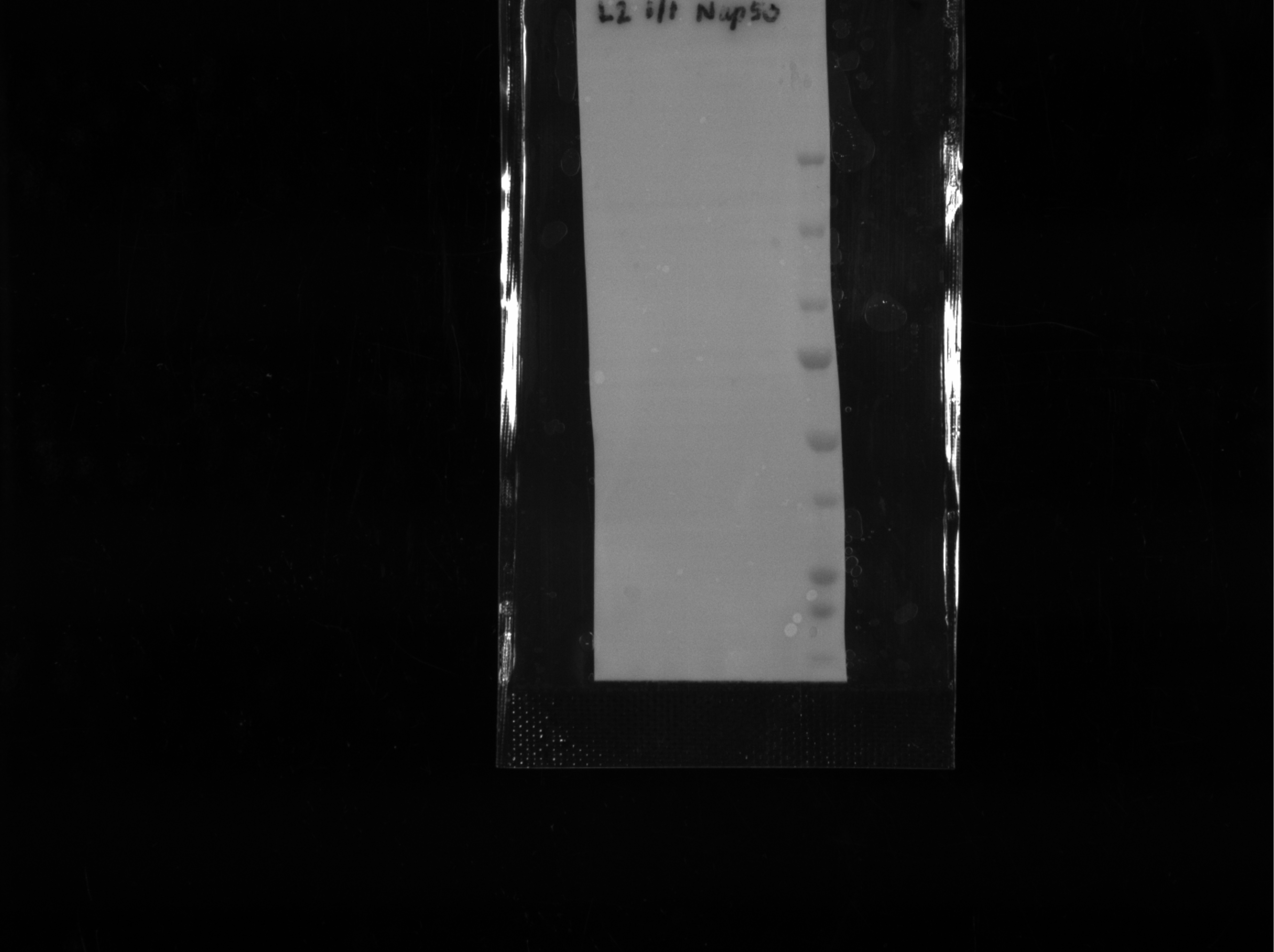

Supplement: Figure 1—source data 11. [file elife-108672-fig1-data11.zip › Fig 1D (part 1)/Nup50/A_26DEC23 3Cpro_blot COLORIMETRIC Nup50 0.1s MJE2.tif]

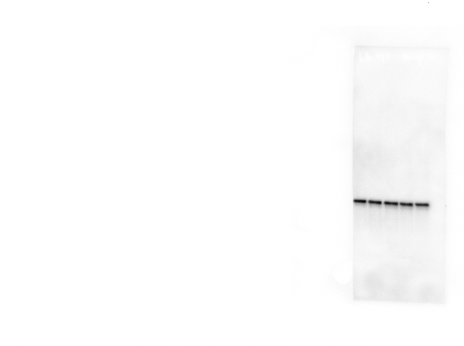

Supplement: Figure 1—source data 11. [file elife-108672-fig1-data11.zip › Fig 1D (part 1)/Nup50/B_26DEC23 3Cpro_blot CHEMI Nup50 8s Quantified MJE3.tif]

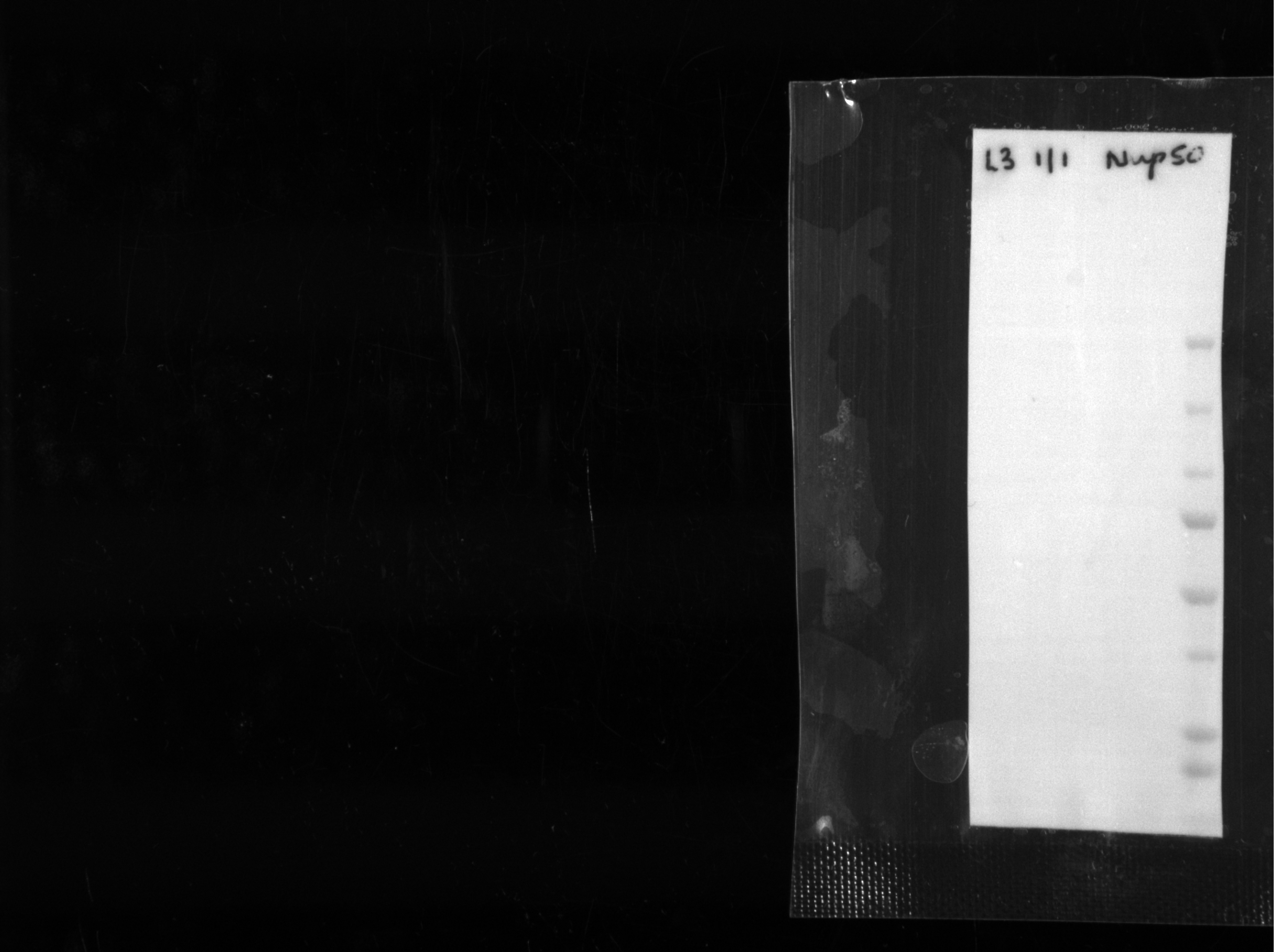

Supplement: Figure 1—source data 11. [file elife-108672-fig1-data11.zip › Fig 1D (part 1)/Nup50/B_26DEC23 3Cpro_blot COLORIMETRIC Nup50 0.1s MJE3 (1).tif]

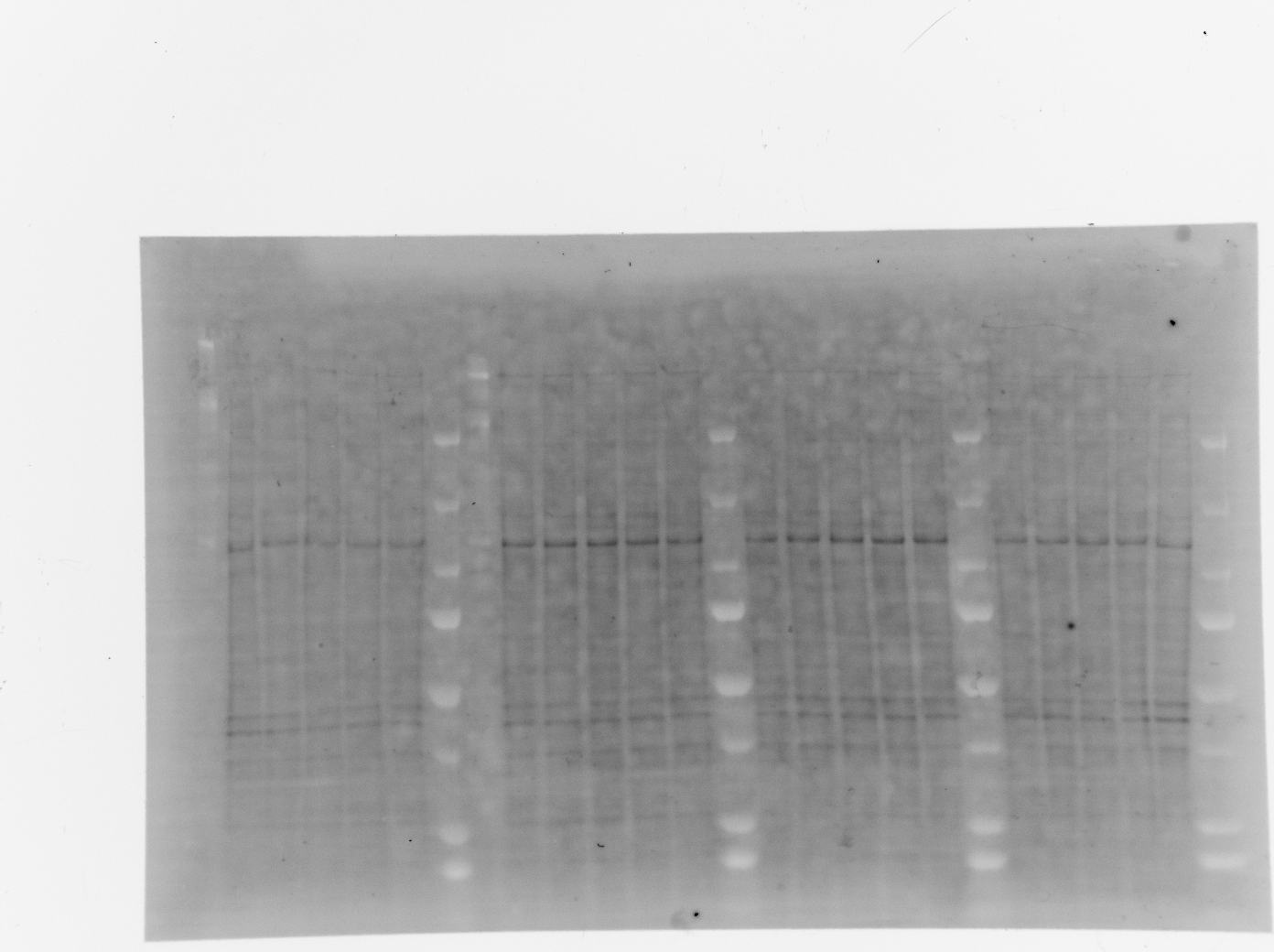

Supplement: Figure 1—source data 11. [file elife-108672-fig1-data11.zip › Fig 1D (part 1)/Nup50/B_26DEC23 3Cpro_blot stain free BLOT (RanBP2, Nup214, Nup50, Nup188) 1s MJE3.tif]

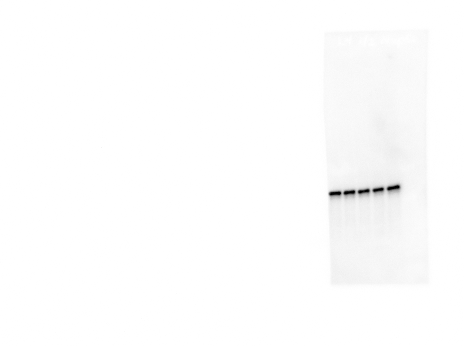

Supplement: Figure 1—source data 11. [file elife-108672-fig1-data11.zip › Fig 1D (part 1)/Nup50/C_2JAN24 3Cpro_blot CHEMI Nup50 8s Quantified MJE4.tif]

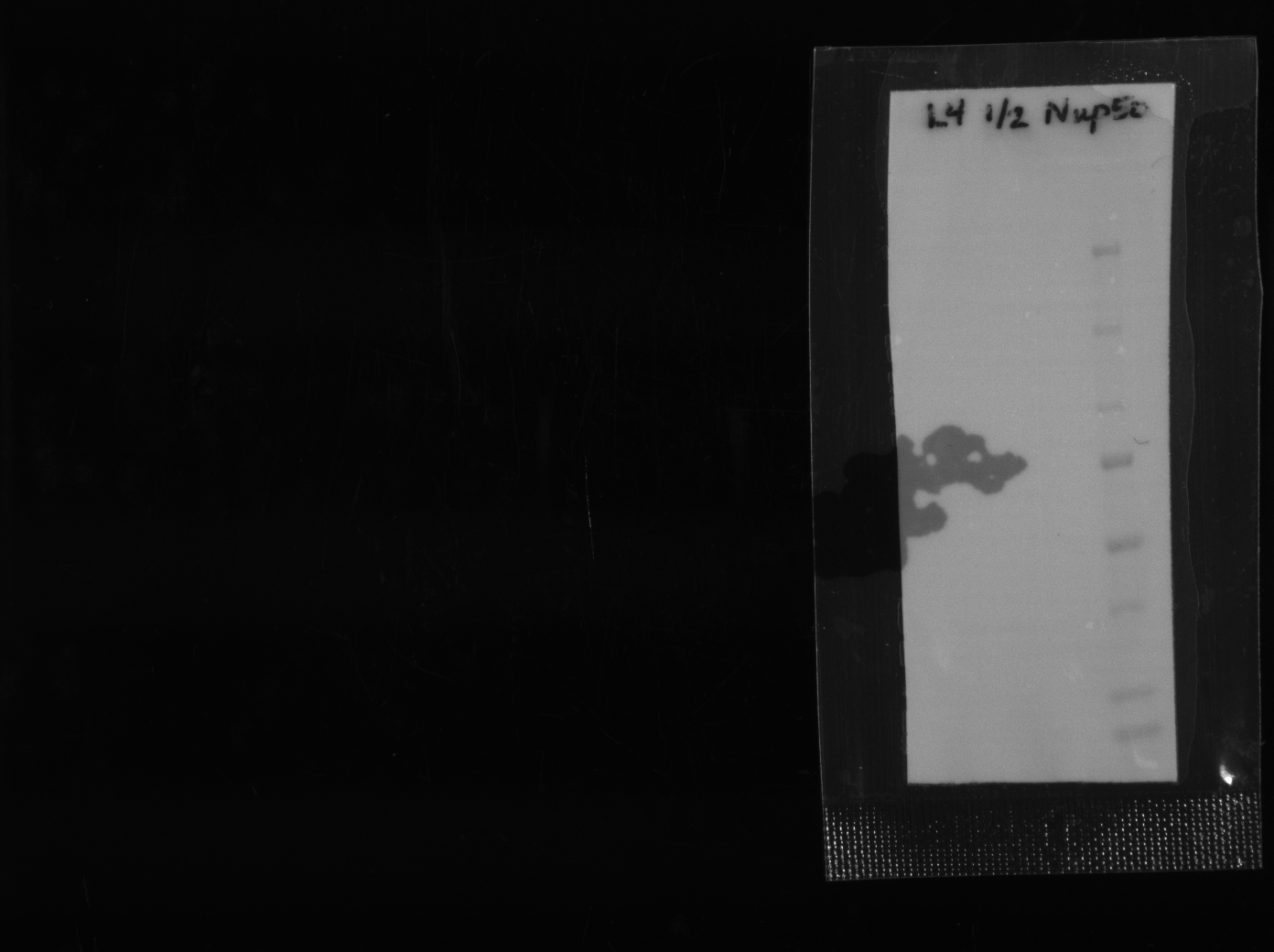

Supplement: Figure 1—source data 11. [file elife-108672-fig1-data11.zip › Fig 1D (part 1)/Nup50/C_2JAN24 3Cpro_blot COLORIMETRIC Nup50 0.1s MJE4.tif]

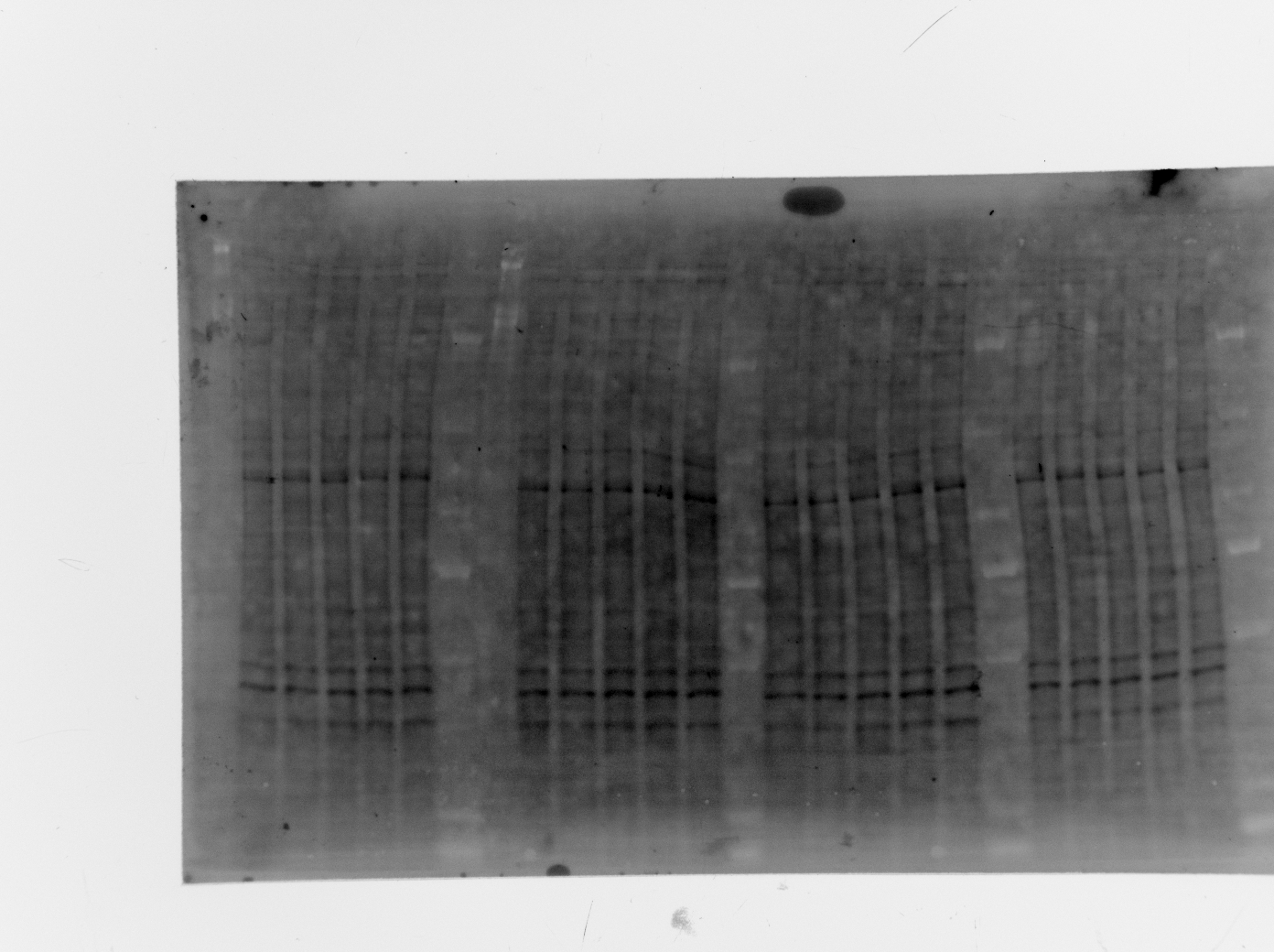

Supplement: Figure 1—source data 11. [file elife-108672-fig1-data11.zip › Fig 1D (part 1)/Nup50/C_2JAN24 3Cpro_blot stain free BLOT AFTER transfer (RanBP2, Nup214, Nup50, Nup188) 1.5s MJE4.tif]

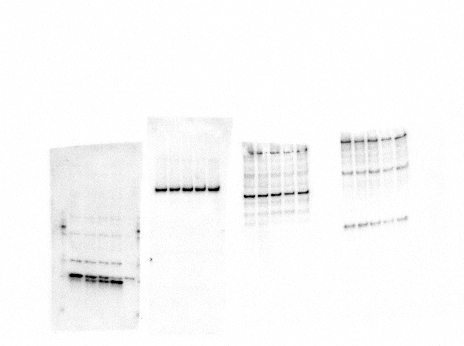

Supplement: Figure 1—source data 12. [file elife-108672-fig1-data12.zip › Fig 1D (part 2)/Nup188/A_Nup188 Blot 2 (group 2).tif]

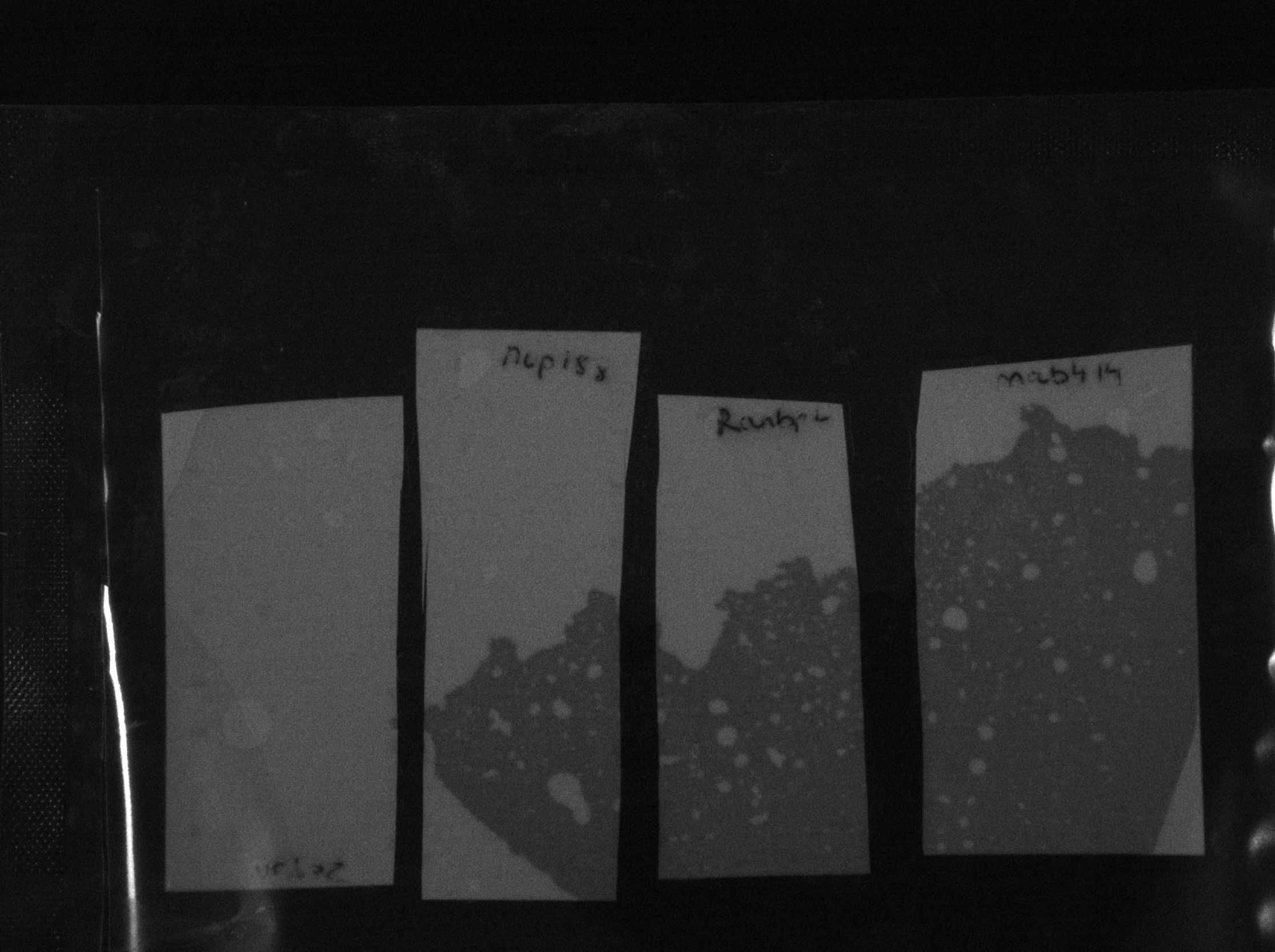

Supplement: Figure 1—source data 12. [file elife-108672-fig1-data12.zip › Fig 1D (part 2)/Nup188/A_Nup188 Ladder 2 (group 2).tif]

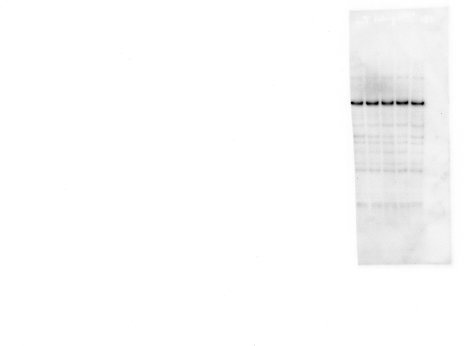

Supplement: Figure 1—source data 12. [file elife-108672-fig1-data12.zip › Fig 1D (part 2)/Nup188/B_26DEC23 3Cpro_blot CHEMI Nup188 90s Quantified MJE3.tif]

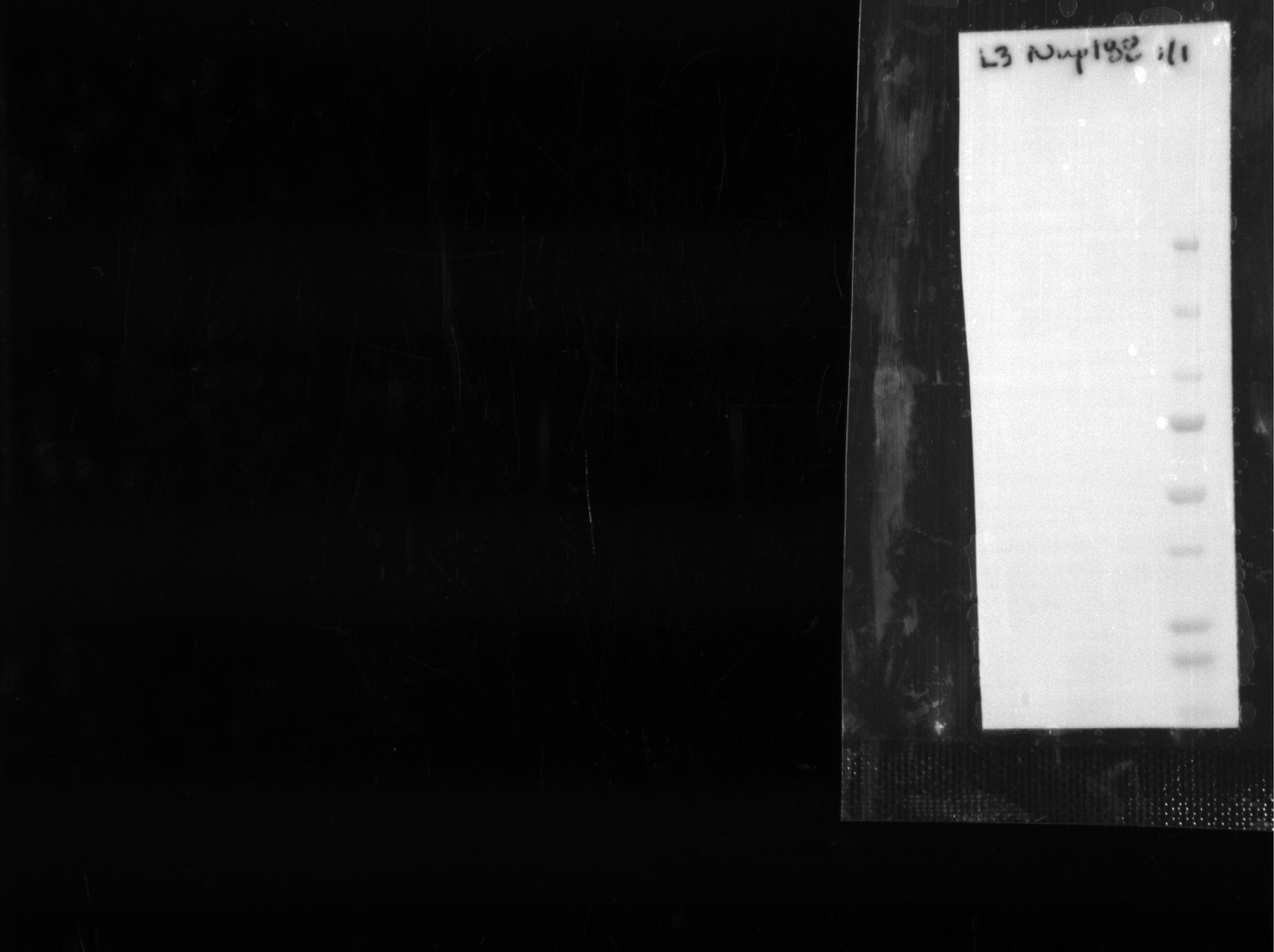

Supplement: Figure 1—source data 12. [file elife-108672-fig1-data12.zip › Fig 1D (part 2)/Nup188/B_26DEC23 3Cpro_blot COLORIMETRIC Nup188 0.1s MJE3.tif]

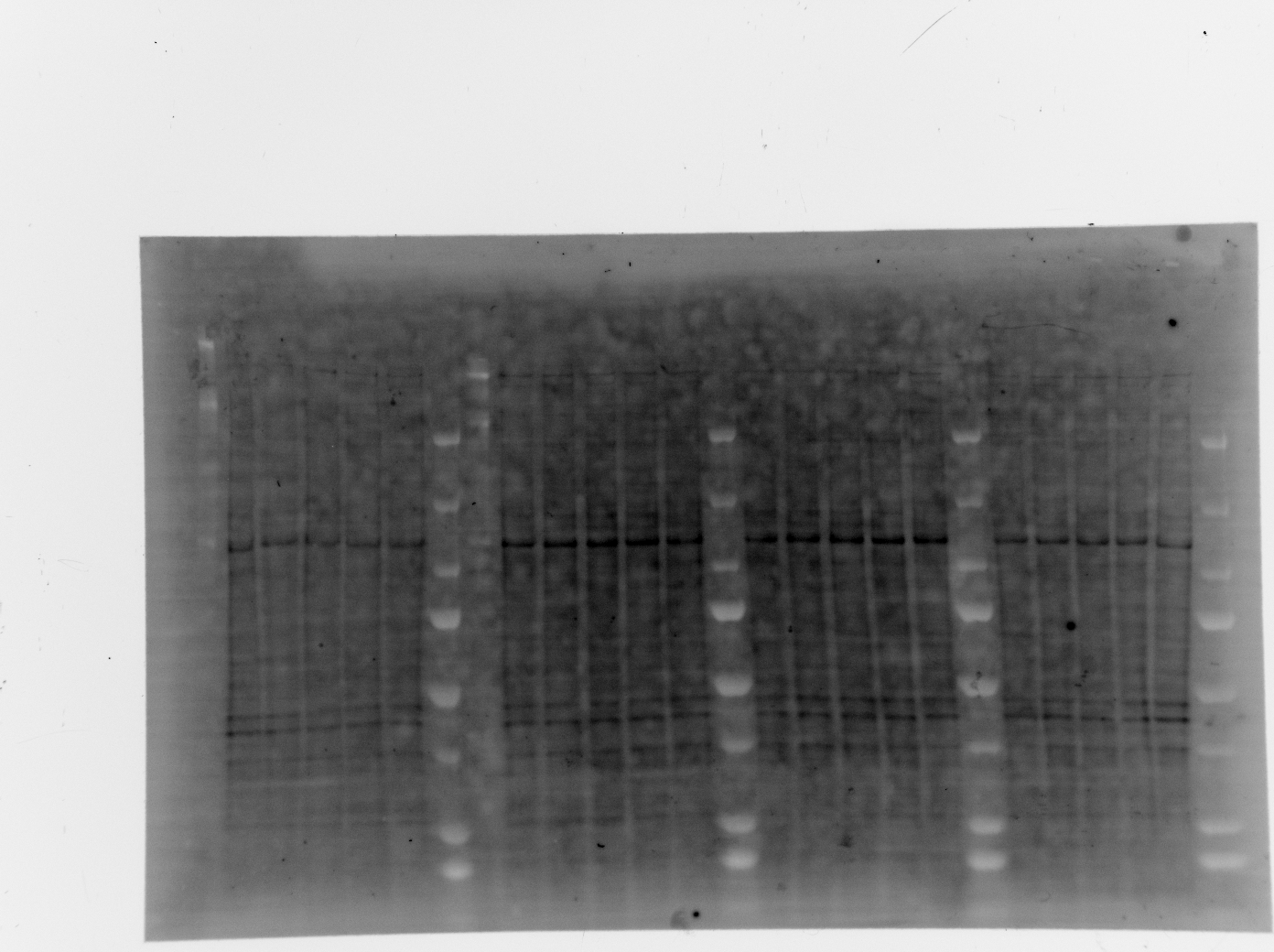

Supplement: Figure 1—source data 12. [file elife-108672-fig1-data12.zip › Fig 1D (part 2)/Nup188/B_26DEC23 3Cpro_blot stain free BLOT (RanBP2, Nup214, Nup50, Nup188) 1.5s MJE3.tif]

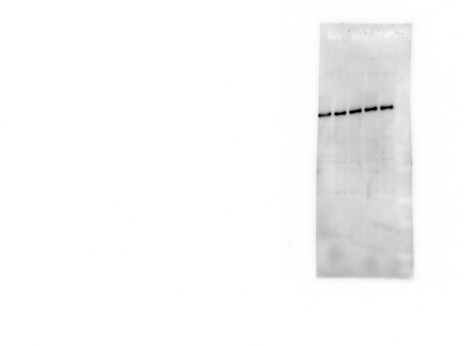

Supplement: Figure 1—source data 12. [file elife-108672-fig1-data12.zip › Fig 1D (part 2)/Nup188/C_2JAN24 3Cpro_blot CHEMI Nup188 30s Quantified MJE4.tif]

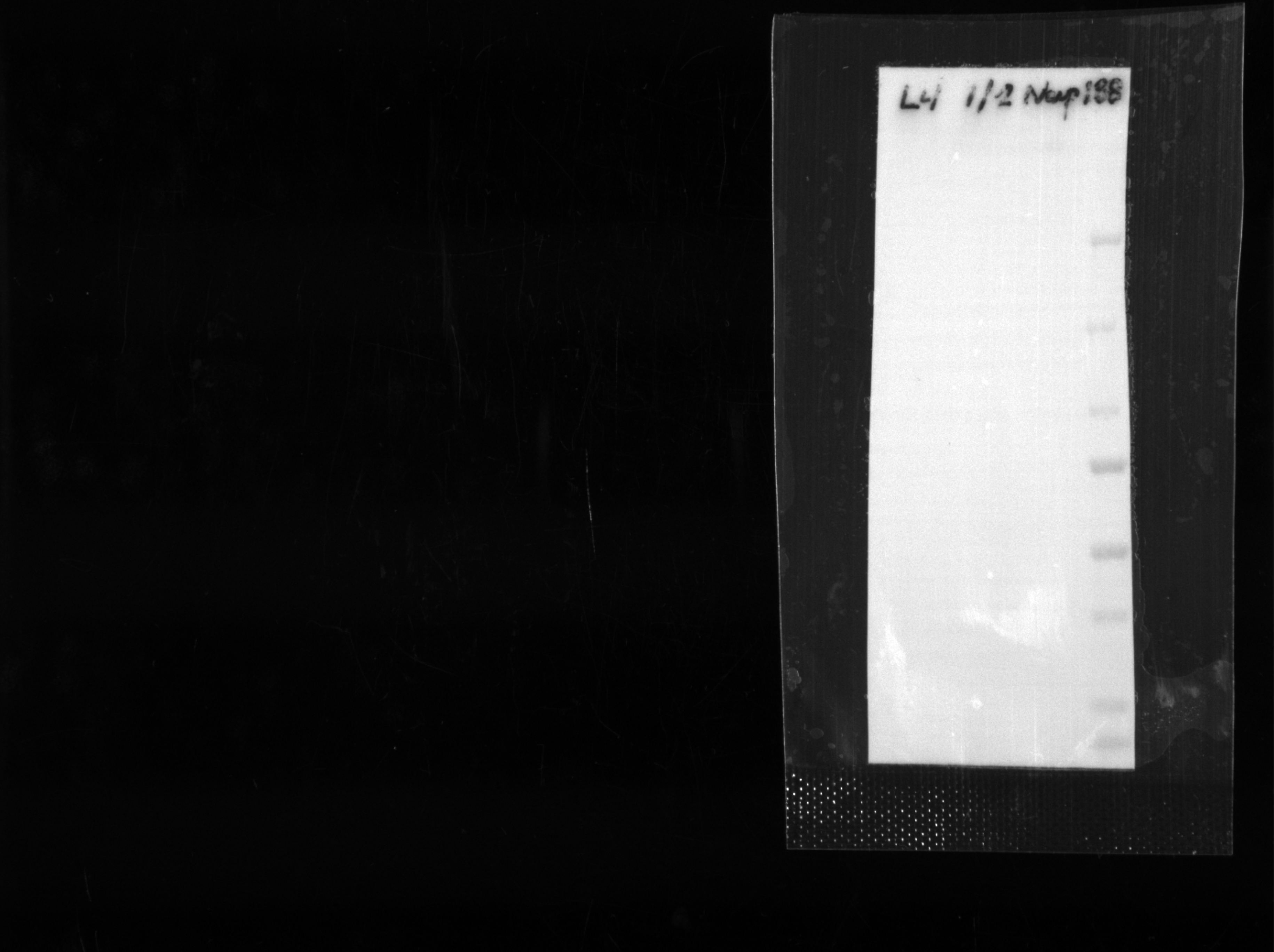

Supplement: Figure 1—source data 12. [file elife-108672-fig1-data12.zip › Fig 1D (part 2)/Nup188/C_2JAN24 3Cpro_blot COLORIMETRIC Nup188 0.1s MJE4.tif]

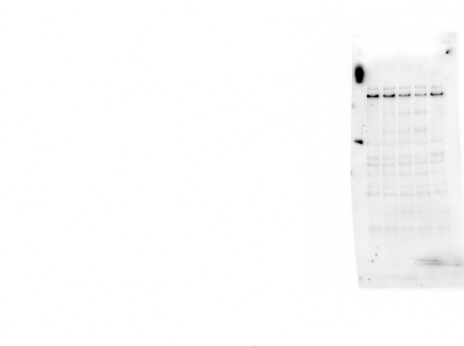

Supplement: Figure 1—source data 12. [file elife-108672-fig1-data12.zip › Fig 1D (part 2)/Nup214/A_26DEC23 3Cpro_blot CHEMI Nup214 200s Quantified MJE2.tif]

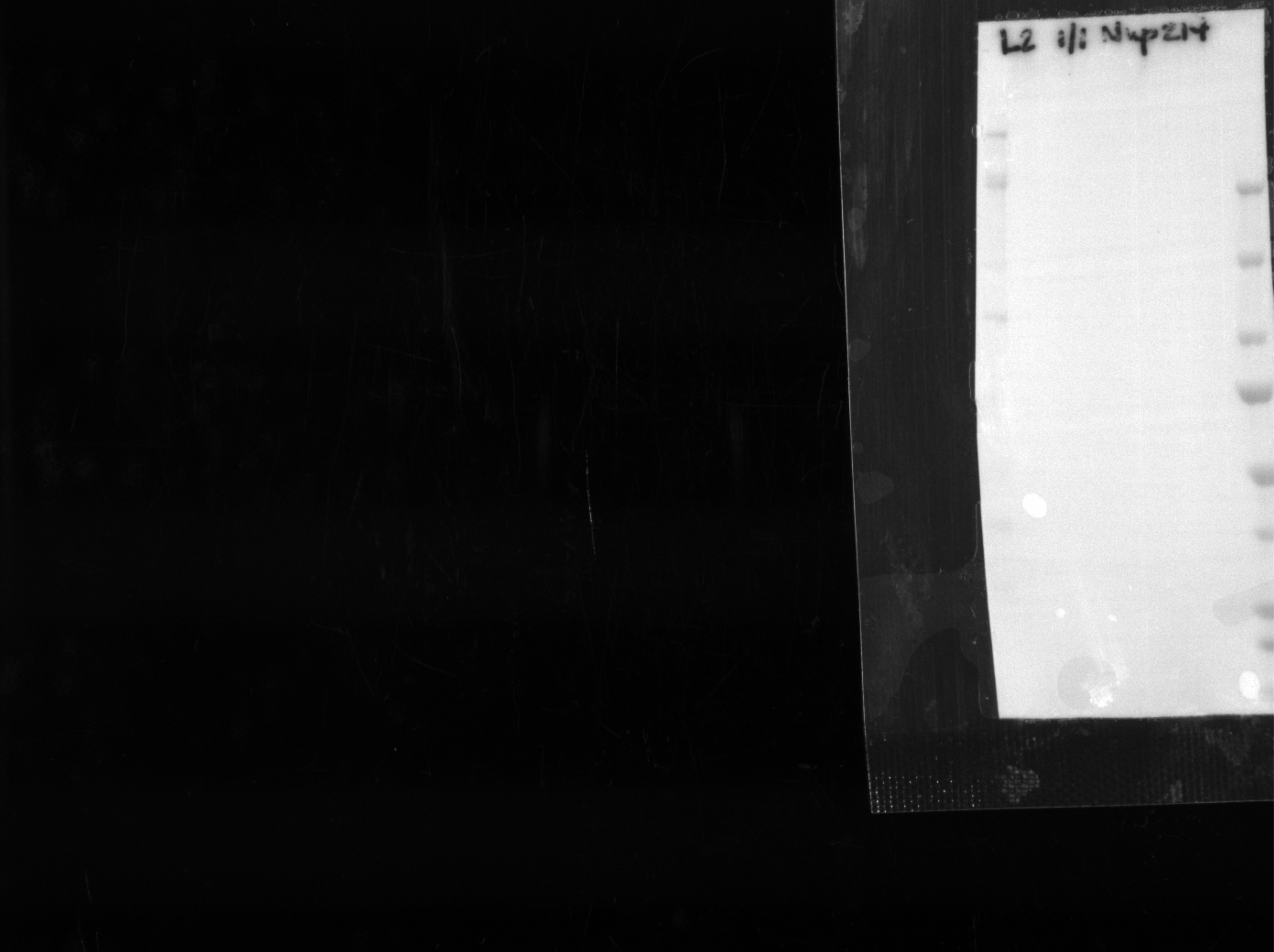

Supplement: Figure 1—source data 12. [file elife-108672-fig1-data12.zip › Fig 1D (part 2)/Nup214/A_26DEC23 3Cpro_blot COLORIMETRIC Nup214 0.1s MJE2.tif]

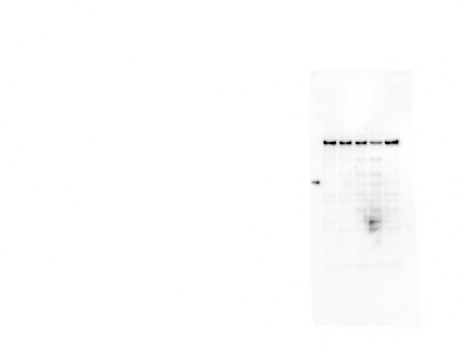

Supplement: Figure 1—source data 12. [file elife-108672-fig1-data12.zip › Fig 1D (part 2)/Nup214/B_26DEC23 3Cpro_blot CHEMI Nup214 40s Quantified MJE3.tif]

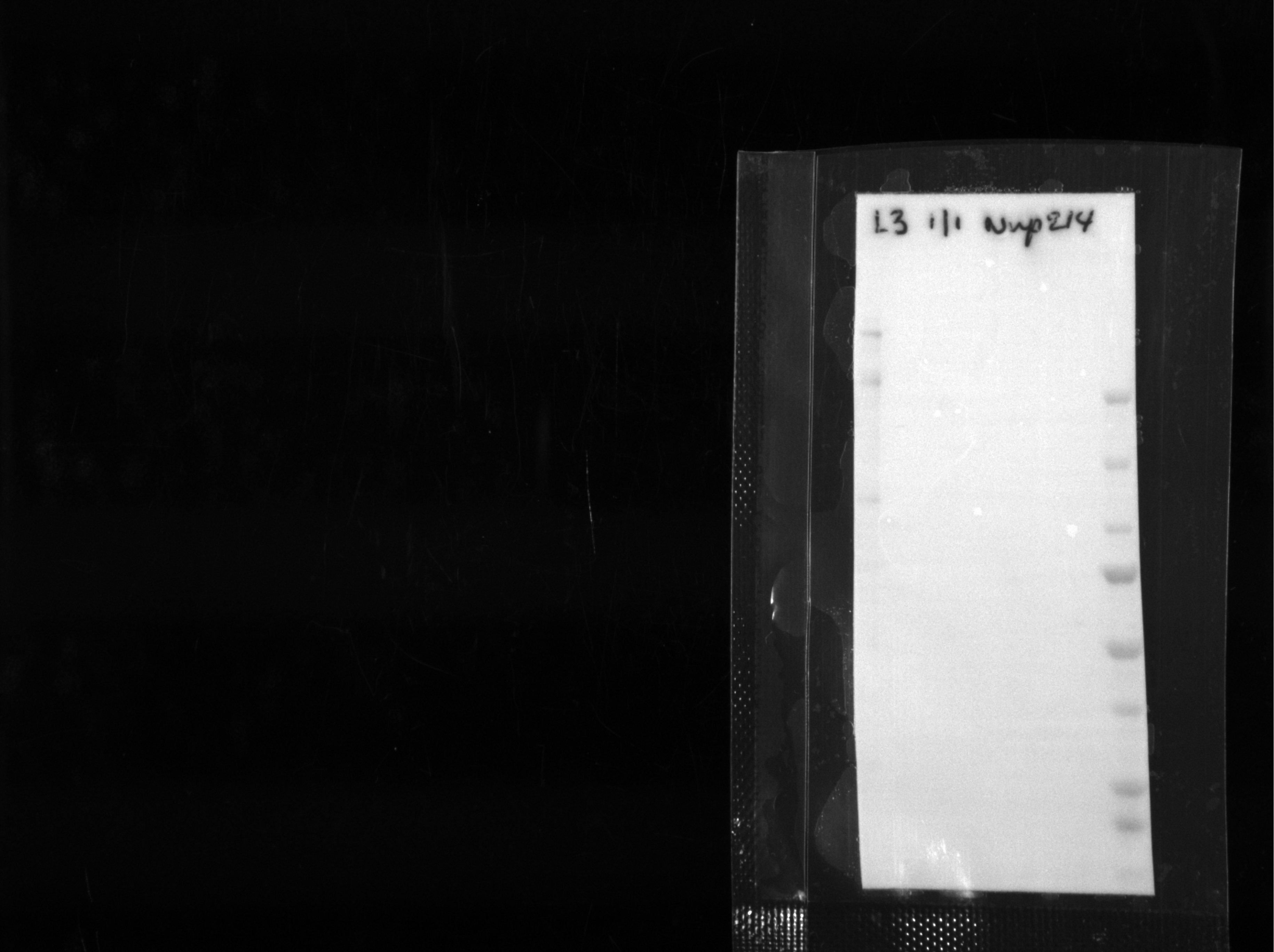

Supplement: Figure 1—source data 12. [file elife-108672-fig1-data12.zip › Fig 1D (part 2)/Nup214/B_26DEC23 3Cpro_blot COLORIMETRIC Nup214 0.1s MJE3.tif]

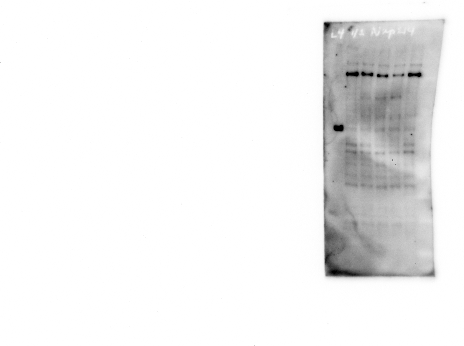

Supplement: Figure 1—source data 12. [file elife-108672-fig1-data12.zip › Fig 1D (part 2)/Nup214/C_2JAN24 3Cpro_blot CHEMI Nup214 200s Quantified MJE4.tif]

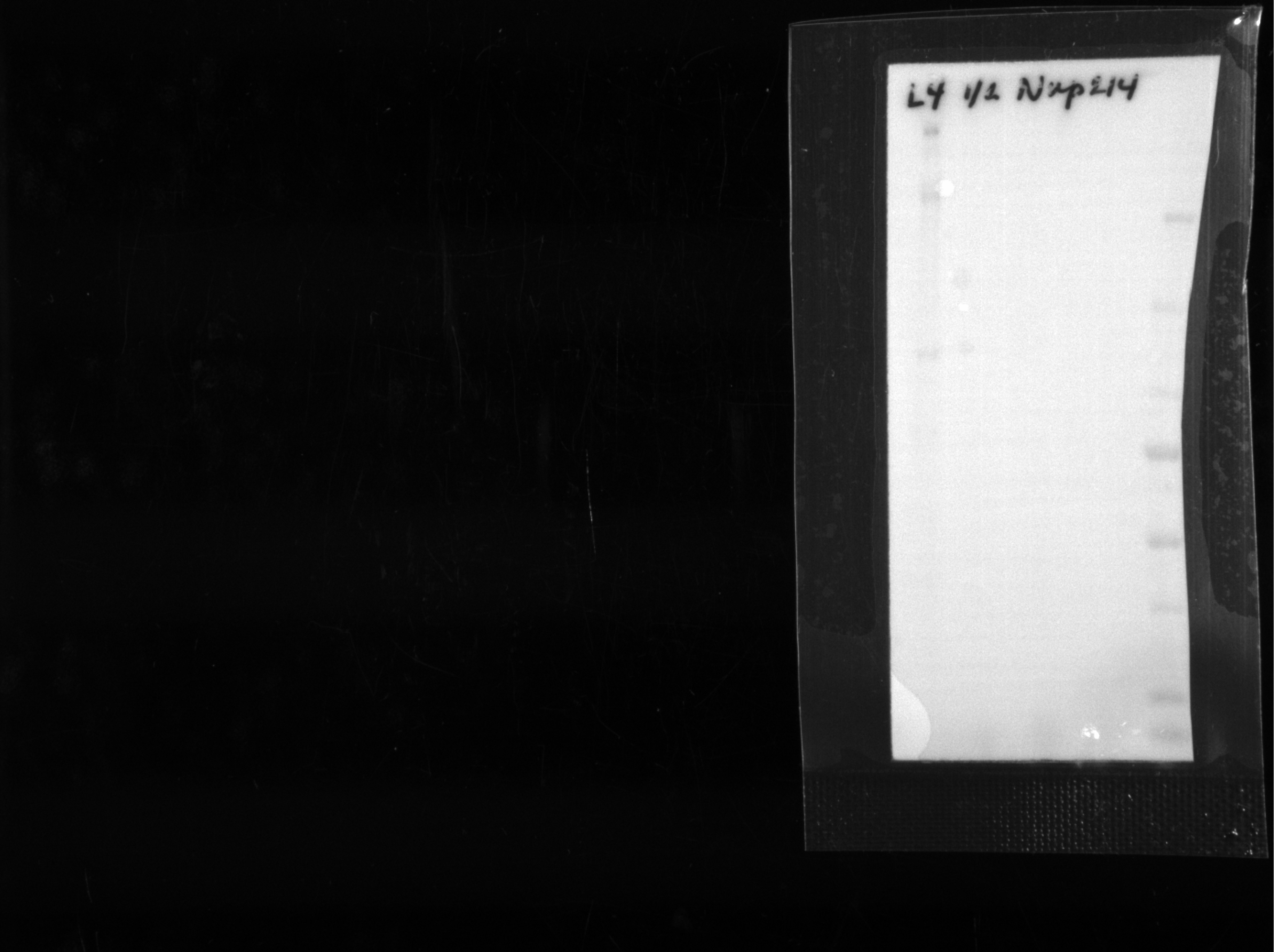

Supplement: Figure 1—source data 12. [file elife-108672-fig1-data12.zip › Fig 1D (part 2)/Nup214/C_2JAN24 3Cpro_blot COLORIMETRIC Nup214 0.1s MJE4.tif]

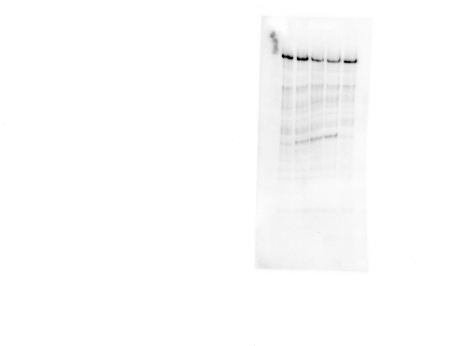

Supplement: Figure 1—source data 12. [file elife-108672-fig1-data12.zip › Fig 1D (part 2)/RanBP2/A_26DEC23 3Cpro_blot CHEMI RanBP2 60s Quantified MJE2.tif]

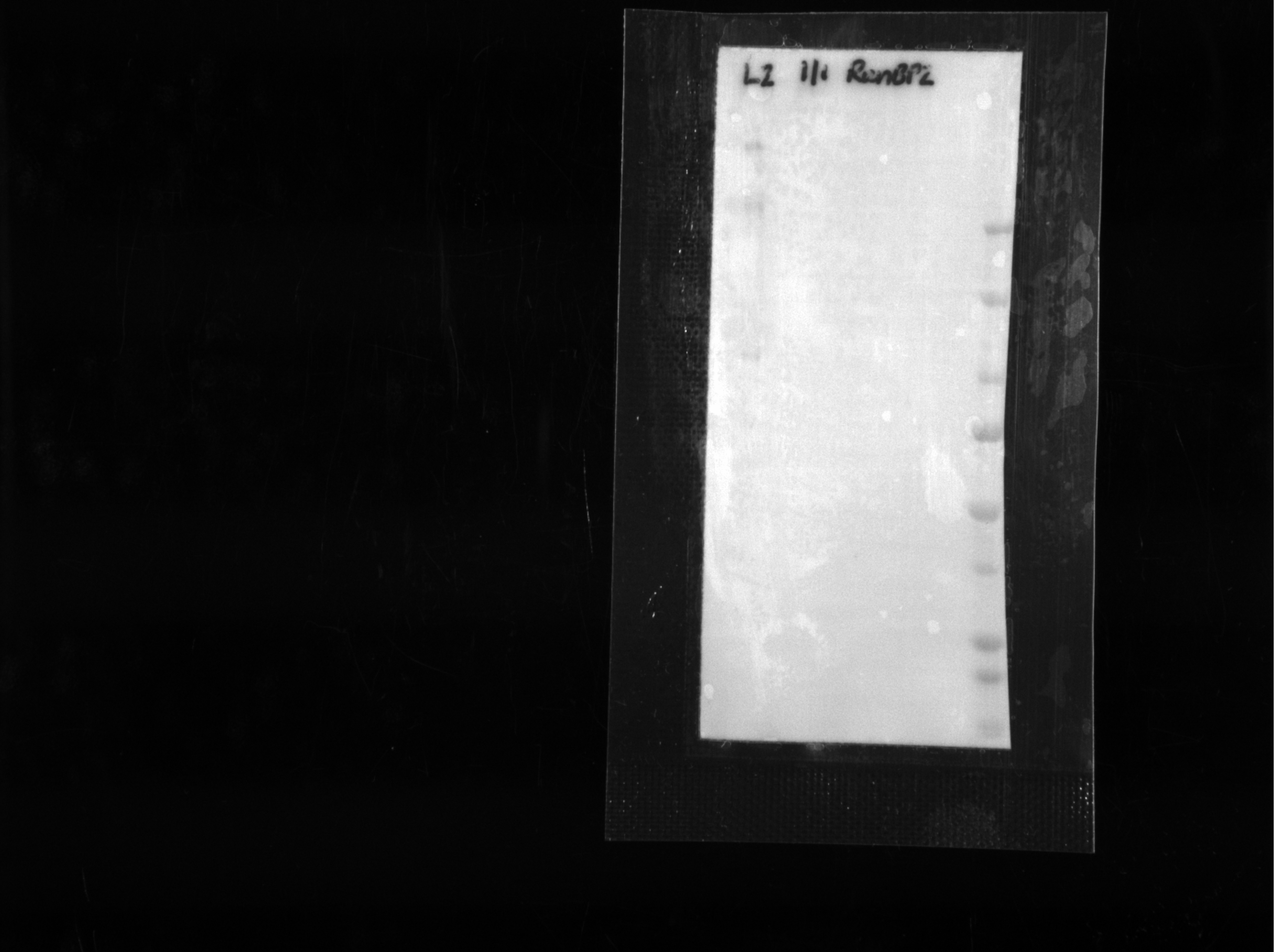

Supplement: Figure 1—source data 12. [file elife-108672-fig1-data12.zip › Fig 1D (part 2)/RanBP2/A_26DEC23 3Cpro_blot COLORIMETRIC RanBP2 0.1s MJE2.tif]

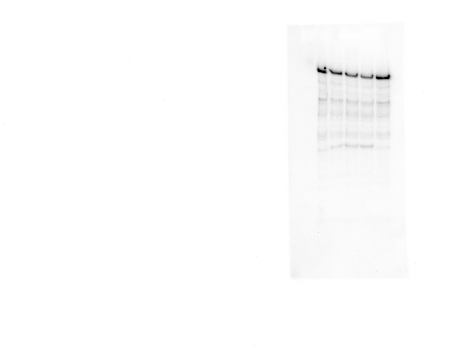

Supplement: Figure 1—source data 12. [file elife-108672-fig1-data12.zip › Fig 1D (part 2)/RanBP2/B_26DEC23 3Cpro_blot CHEMI RanBP2 60s Quantified MJE3.tif]

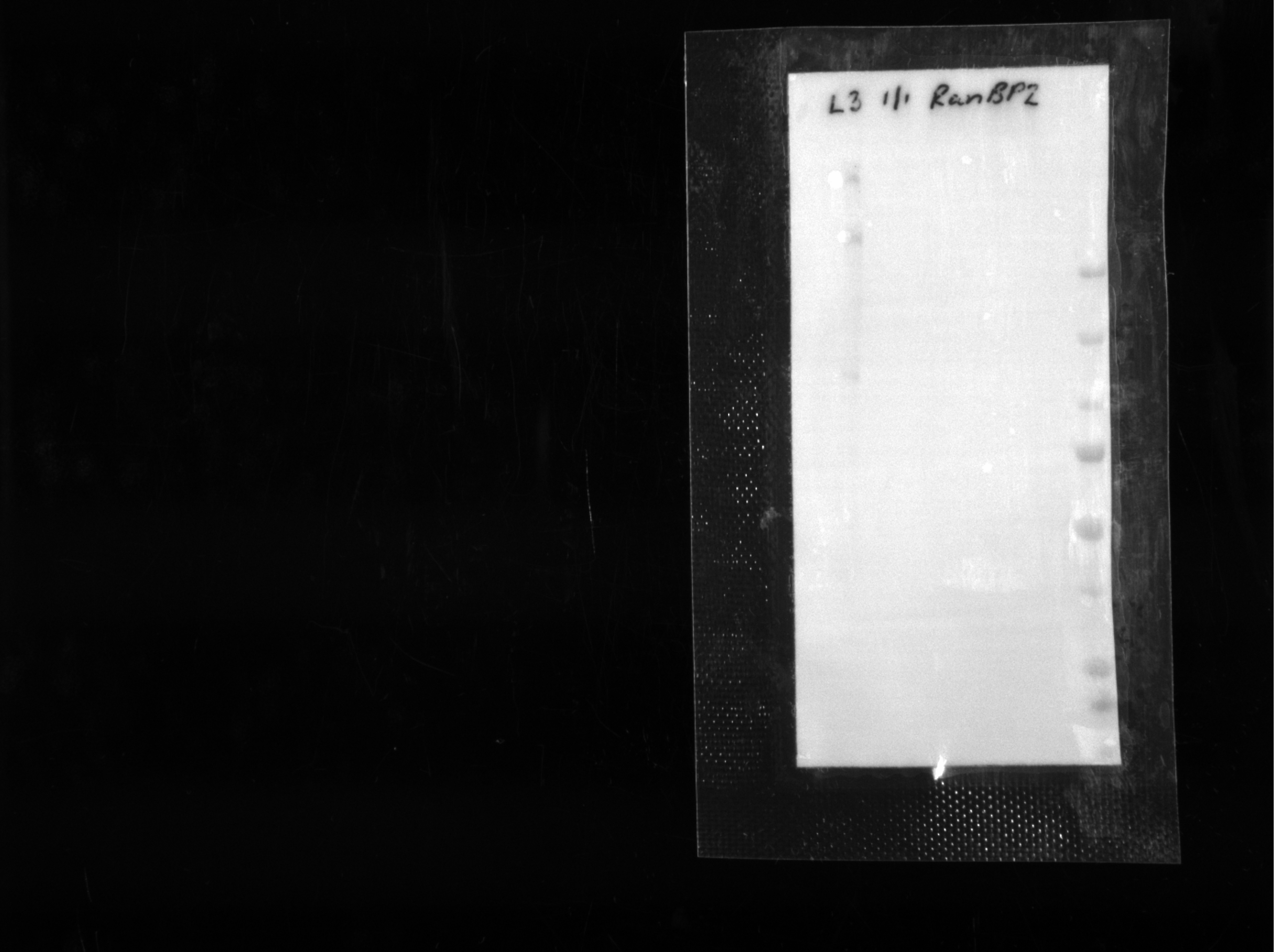

Supplement: Figure 1—source data 12. [file elife-108672-fig1-data12.zip › Fig 1D (part 2)/RanBP2/B_26DEC23 3Cpro_blot COLORIMETRIC RanBP2 0.1s MJE3.tif]

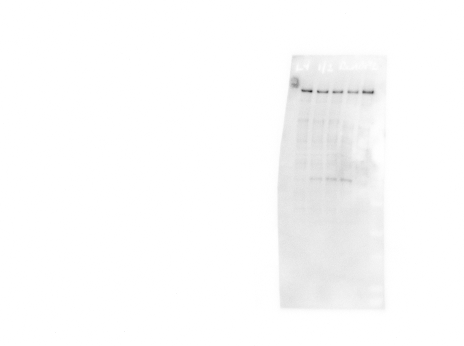

Supplement: Figure 1—source data 12. [file elife-108672-fig1-data12.zip › Fig 1D (part 2)/RanBP2/C_2JAN24 3Cpro_blot CHEMI RanBP2 30s Quantified MJE4.tif]

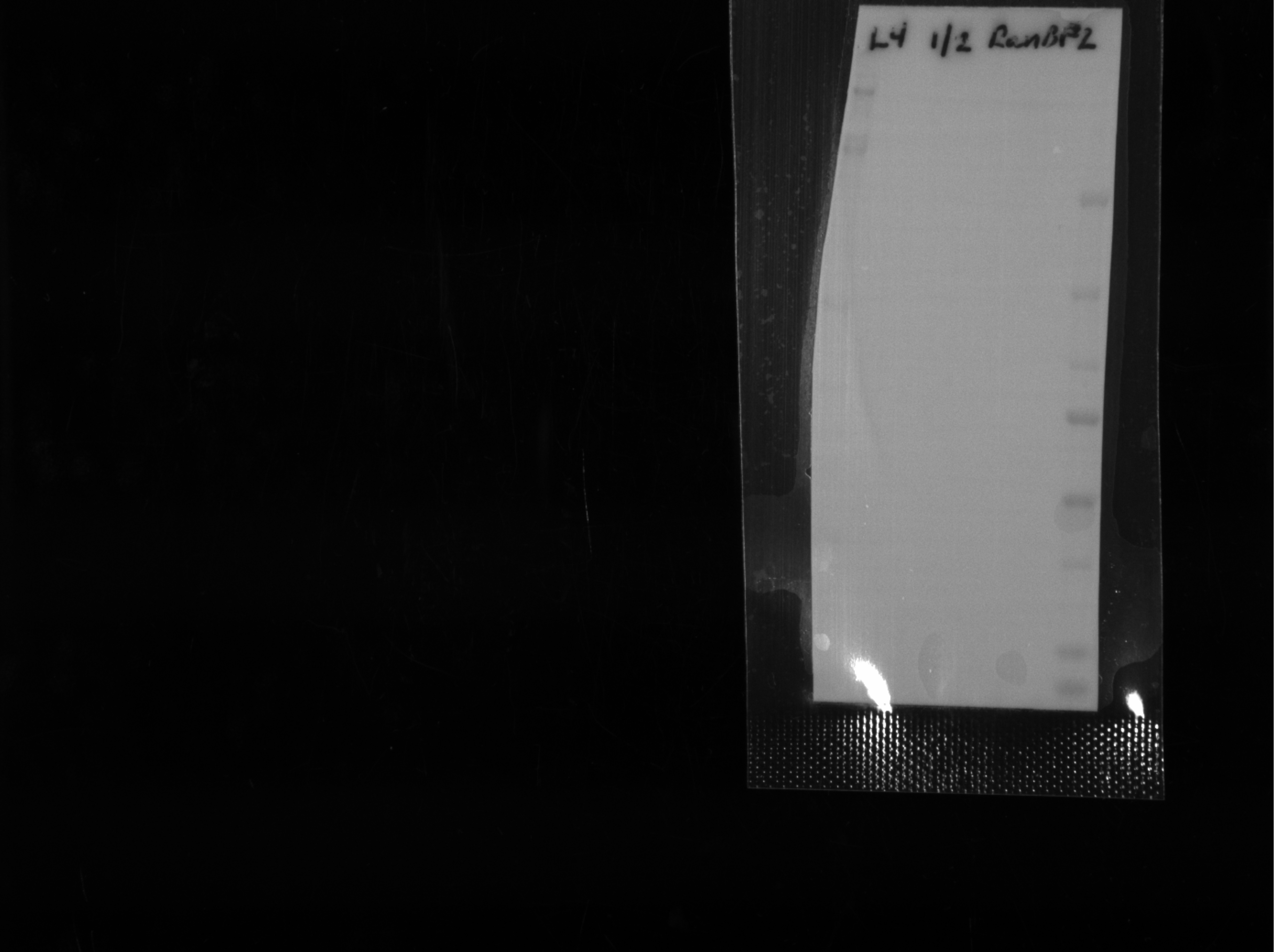

Supplement: Figure 1—source data 12. [file elife-108672-fig1-data12.zip › Fig 1D (part 2)/RanBP2/C_2JAN24 3Cpro_blot COLORIMETRIC RanBP2 0.1s MJE4.tif]

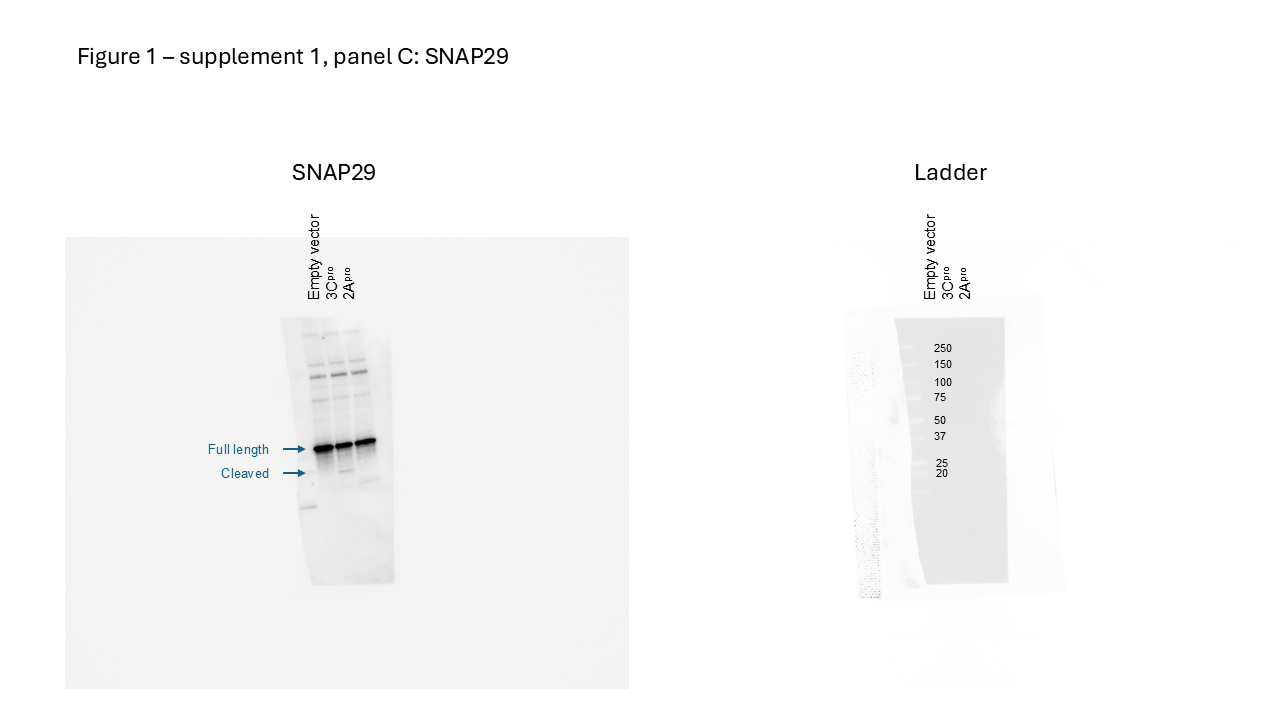

Supplement: Figure 1—figure supplement 1—source data 1. [file elife-108672-fig1-figsupp1-data1.zip › Figure 1-S1 Panel C SNAP29.tif]

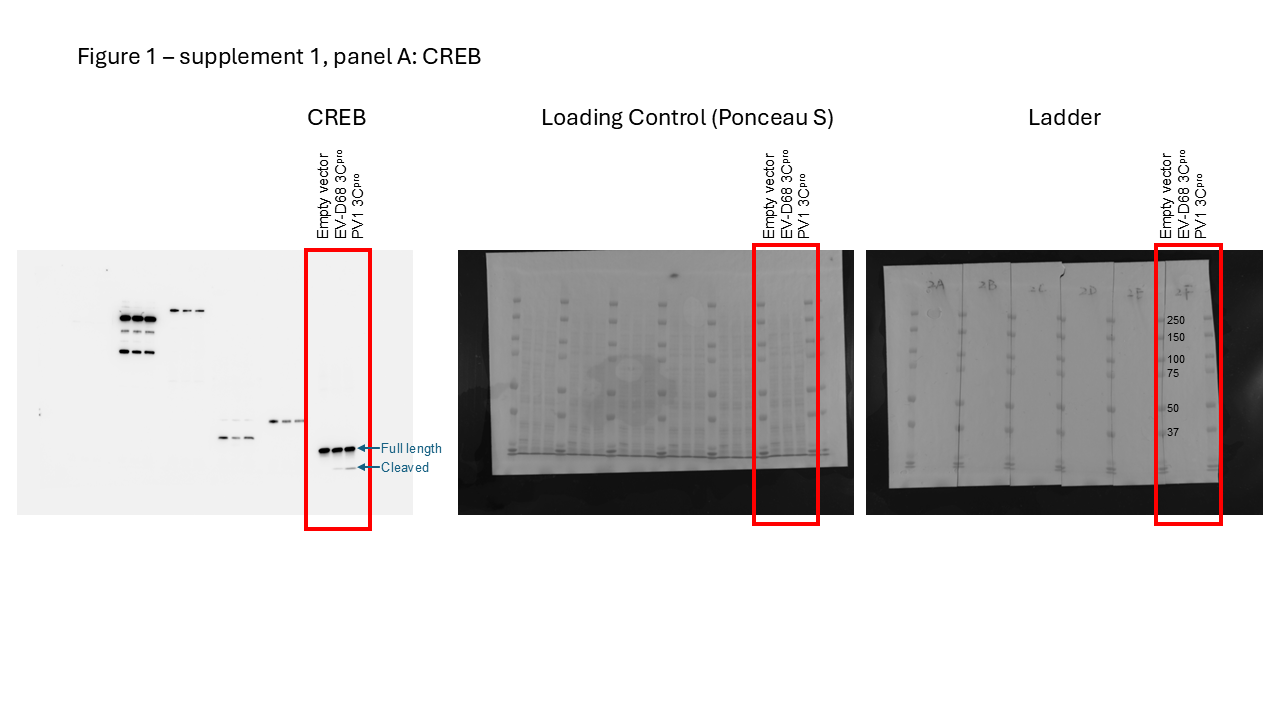

Supplement: Figure 1—figure supplement 1—source data 1. [file elife-108672-fig1-figsupp1-data1.zip › Figure 1-S1 Panel A CREB.tif]

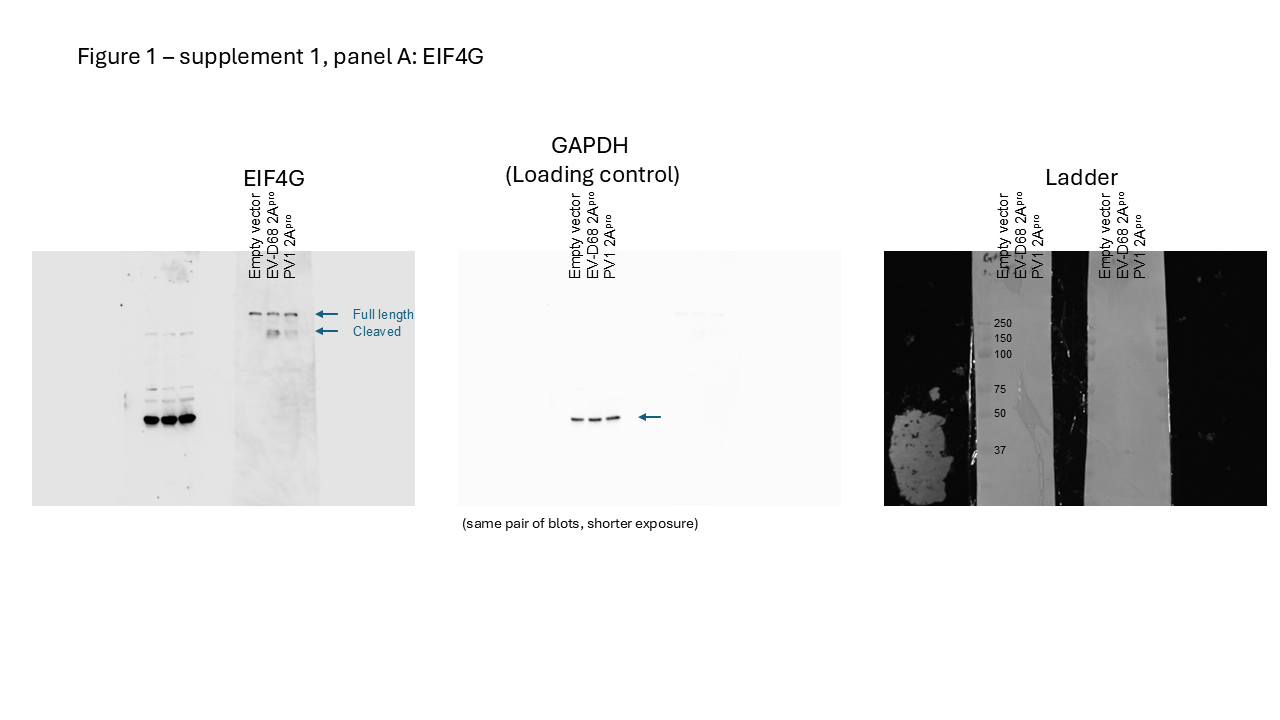

Supplement: Figure 1—figure supplement 1—source data 1. [file elife-108672-fig1-figsupp1-data1.zip › Figure 1-S1 Panel A EIF4G.tif]

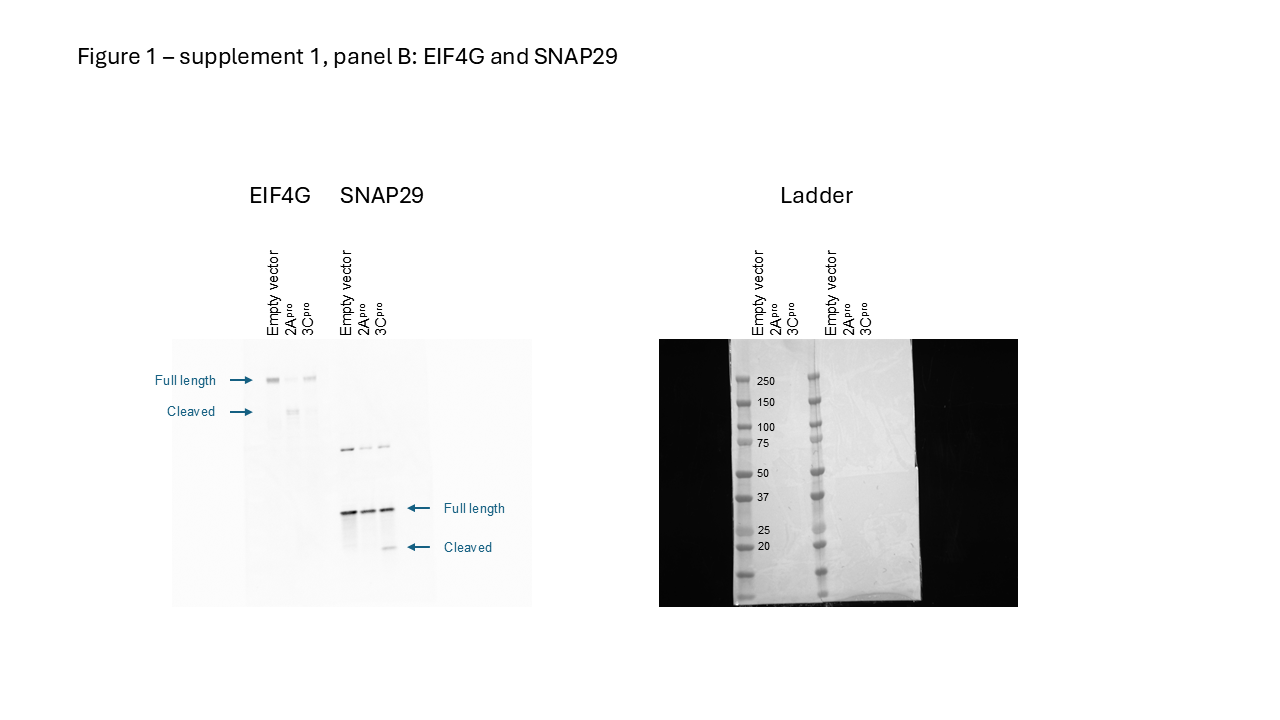

Supplement: Figure 1—figure supplement 1—source data 1. [file elife-108672-fig1-figsupp1-data1.zip › Figure 1-S1 Panel B EIF4G and SNAP29.tif]

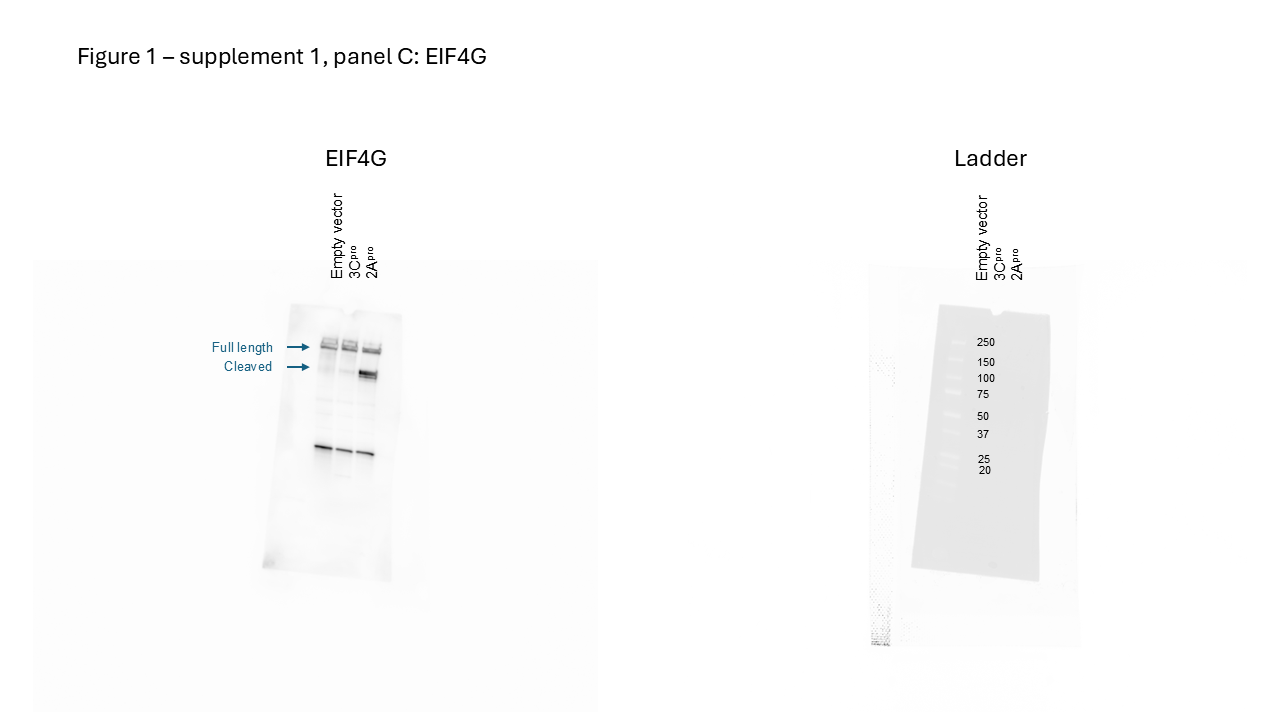

Supplement: Figure 1—figure supplement 1—source data 1. [file elife-108672-fig1-figsupp1-data1.zip › Figure 1-S1 Panel C EIF4G.tif]

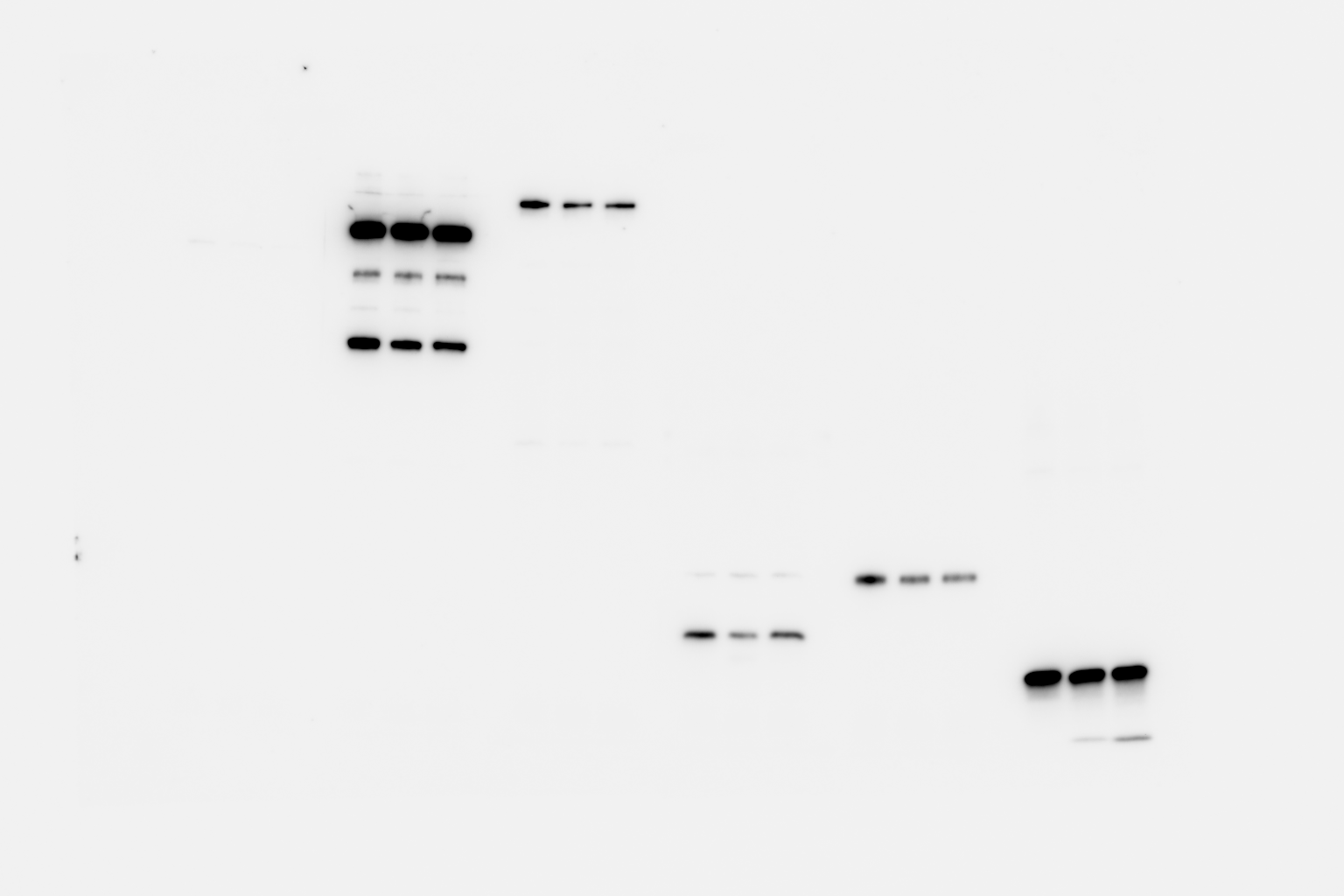

Supplement: Figure 1—figure supplement 1—source data 2. [file elife-108672-fig1-figsupp1-data2.zip › Fig 1-S1/eBFP2-N1_CREB (in 5th group).tif]

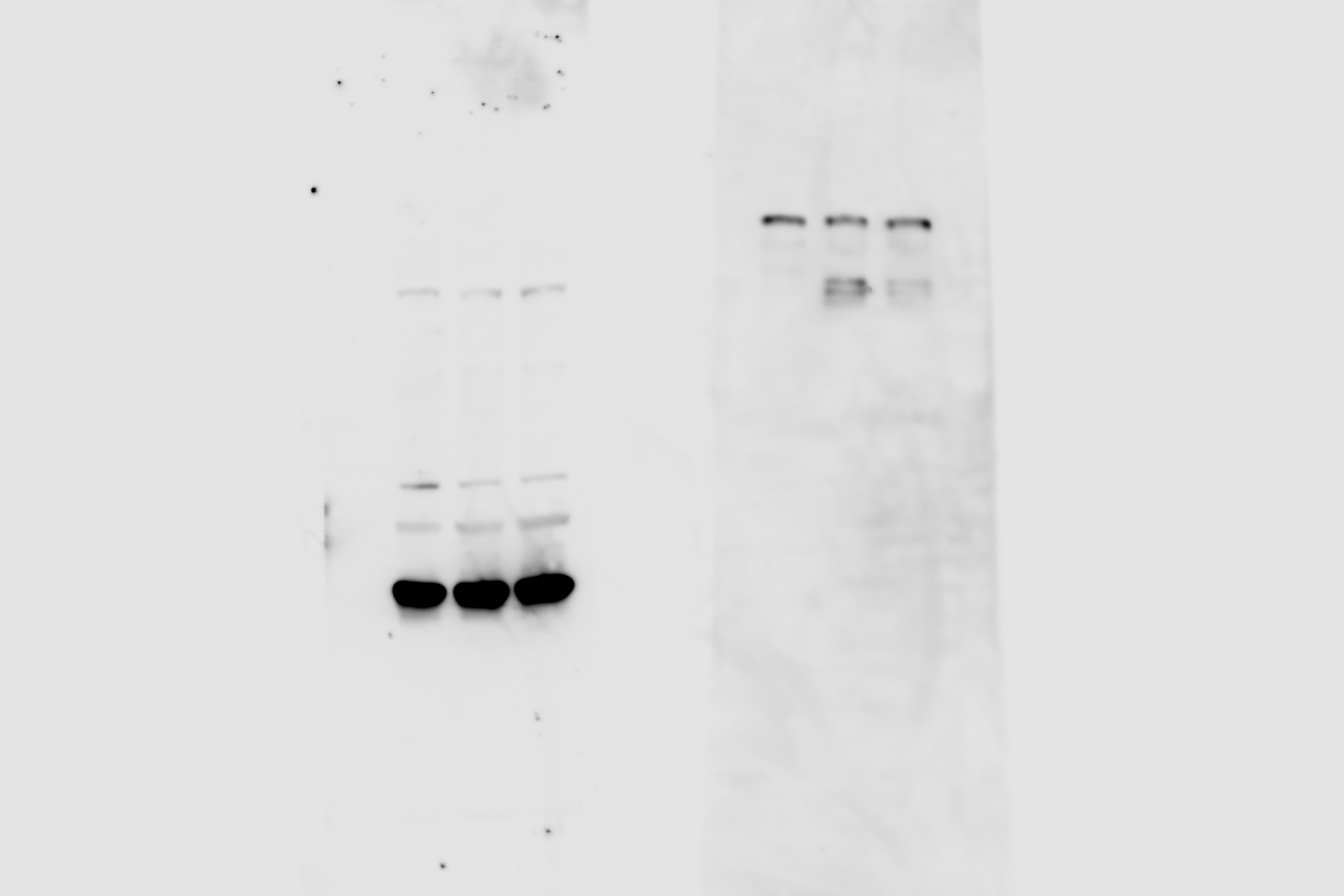

Supplement: Figure 1—figure supplement 1—source data 2. [file elife-108672-fig1-figsupp1-data2.zip › Fig 1-S1/eBFP2-N1_EIF4G (in 2nd group).tif]

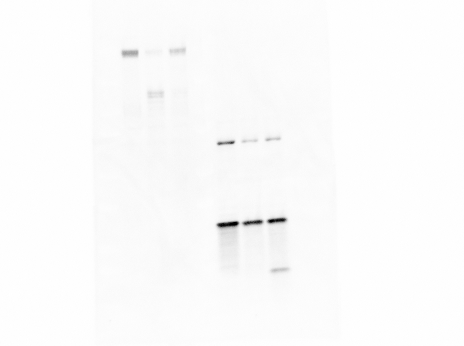

Supplement: Figure 1—figure supplement 1—source data 2. [file elife-108672-fig1-figsupp1-data2.zip › Fig 1-S1/pLenti-IRES-GFP_EIF4G-SNAP29.tif]

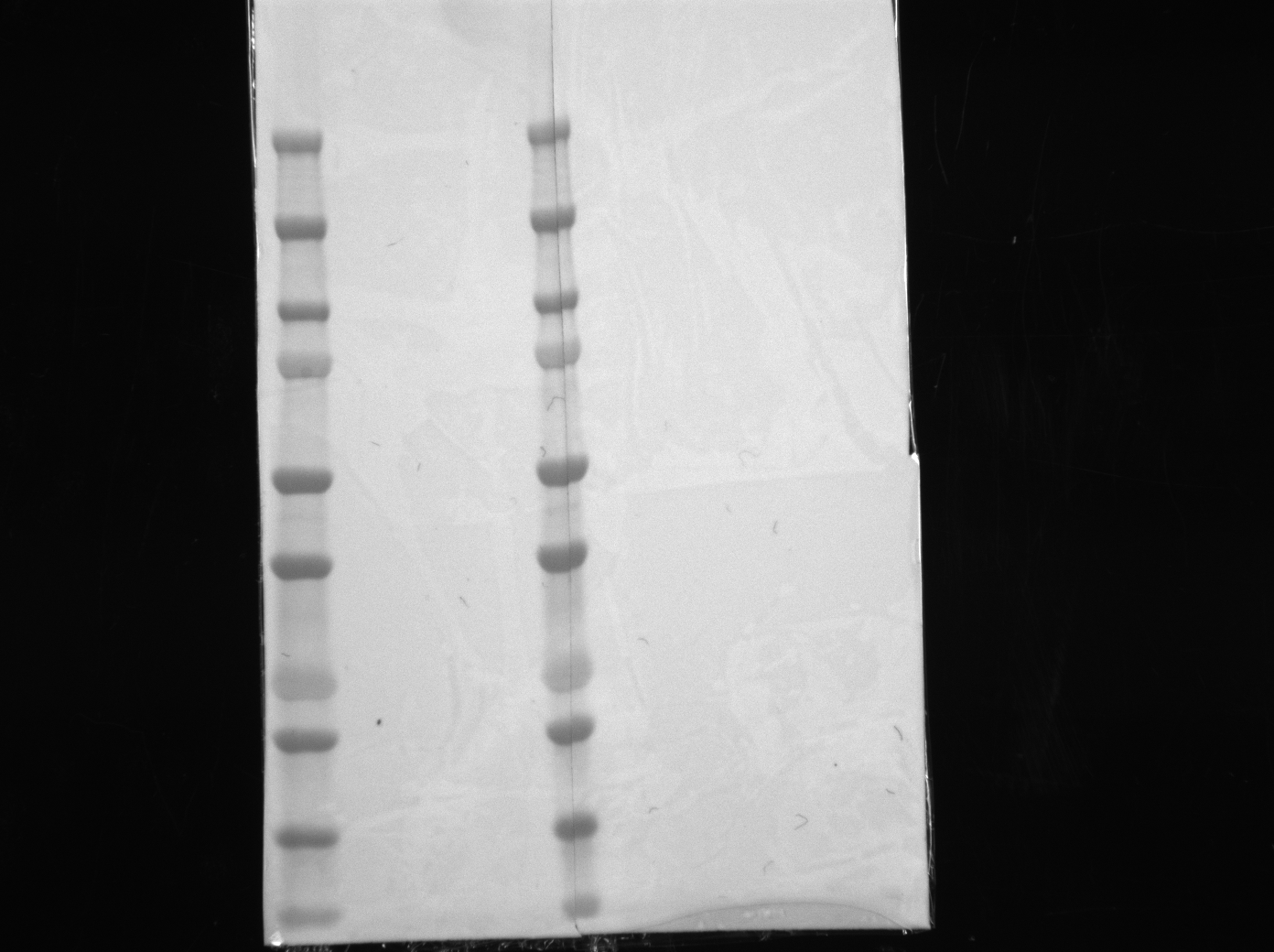

Supplement: Figure 1—figure supplement 1—source data 2. [file elife-108672-fig1-figsupp1-data2.zip › Fig 1-S1/pLenti-IRES-GFP_ladder.tif]

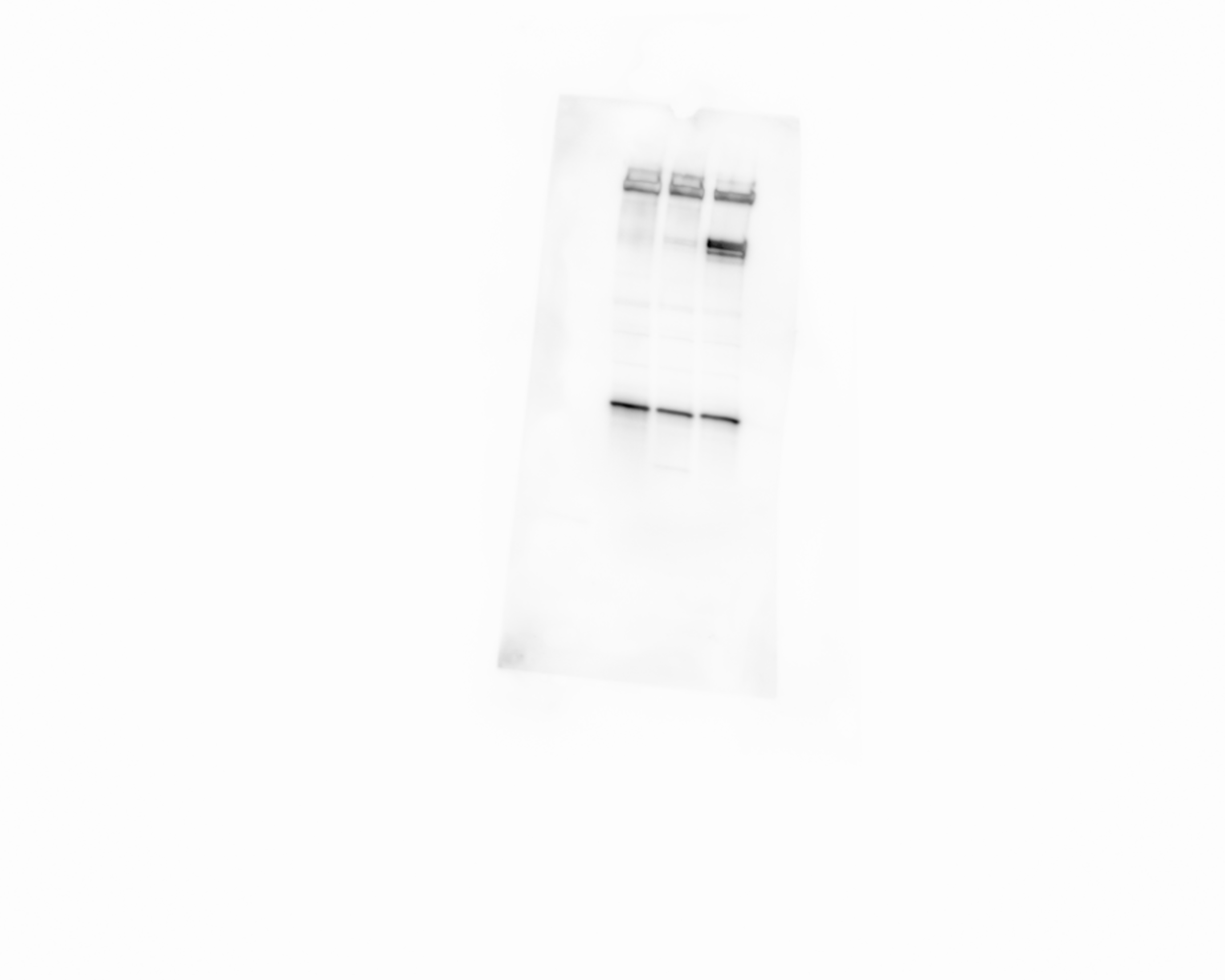

Supplement: Figure 1—figure supplement 1—source data 2. [file elife-108672-fig1-figsupp1-data2.zip › Fig 1-S1/pLenti-IRES-H2A-iRFP_EIF4G.tif]

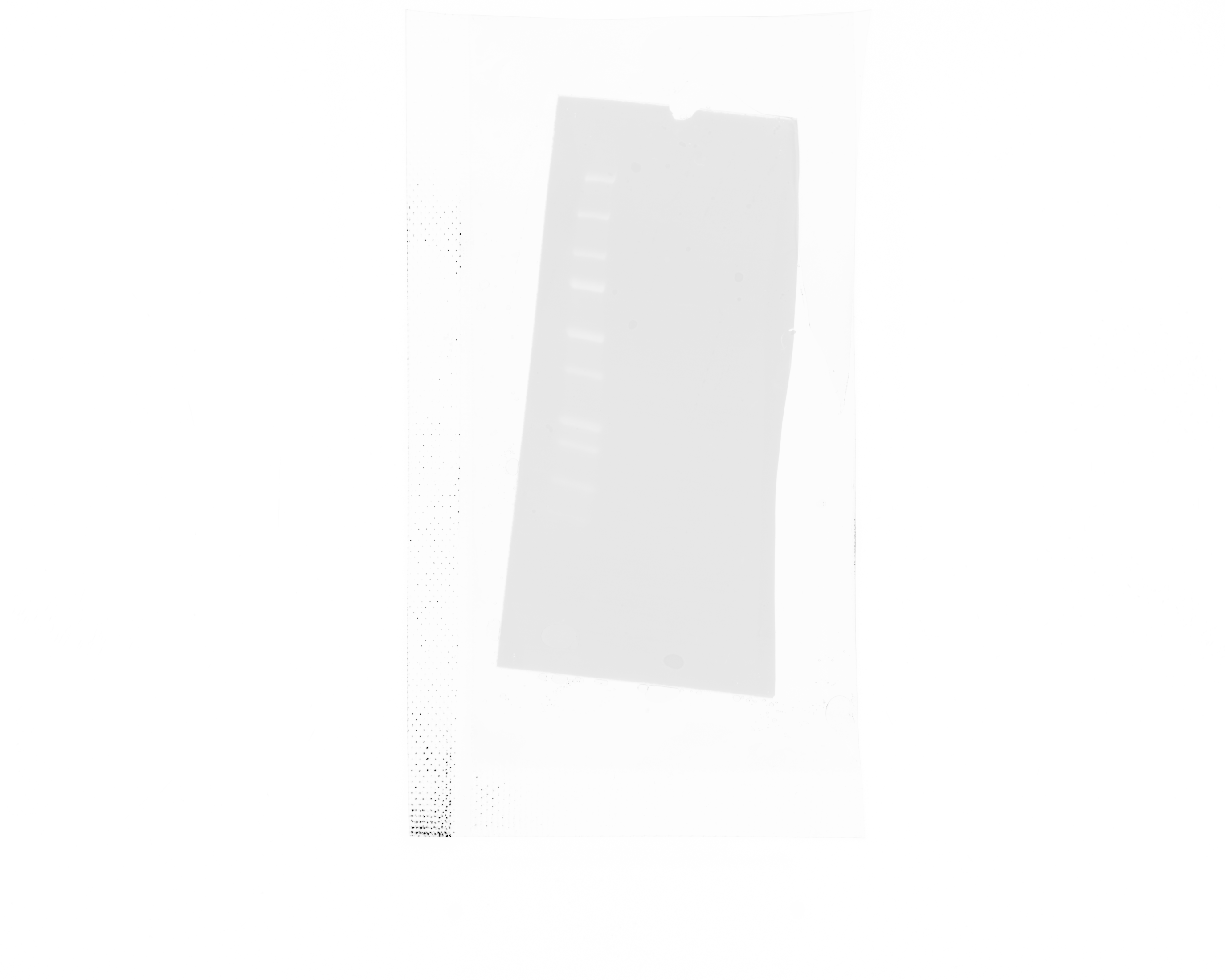

Supplement: Figure 1—figure supplement 1—source data 2. [file elife-108672-fig1-figsupp1-data2.zip › Fig 1-S1/pLenti-IRES-H2A-iRFP_EIF4G_ladder.tif]

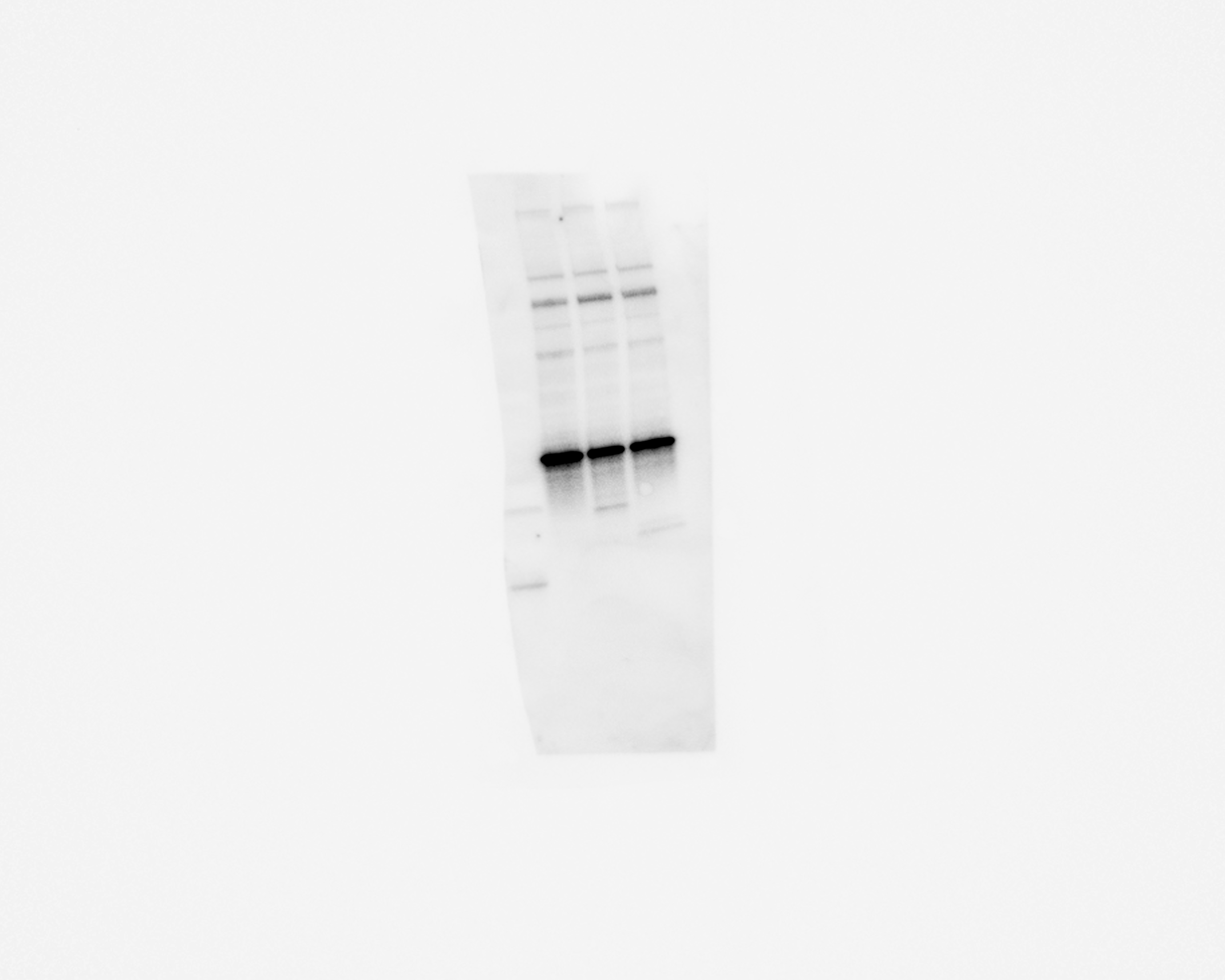

Supplement: Figure 1—figure supplement 1—source data 2. [file elife-108672-fig1-figsupp1-data2.zip › Fig 1-S1/pLenti-IRES-H2A-iRFP_SNAP29.tif]

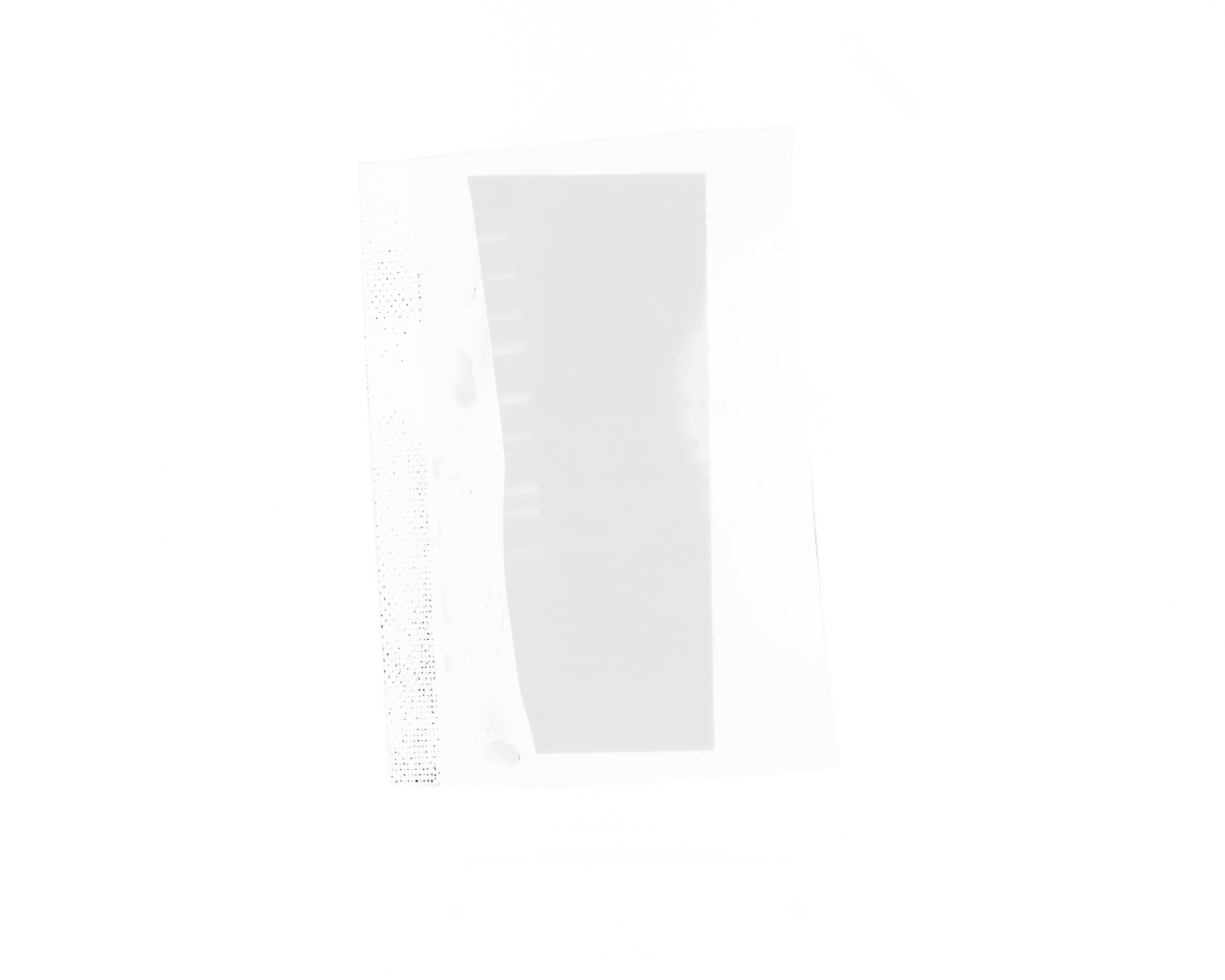

Supplement: Figure 1—figure supplement 1—source data 2. [file elife-108672-fig1-figsupp1-data2.zip › Fig 1-S1/pLenti-IRES-H2A-iRFP_SNAP29_ladder.tif]

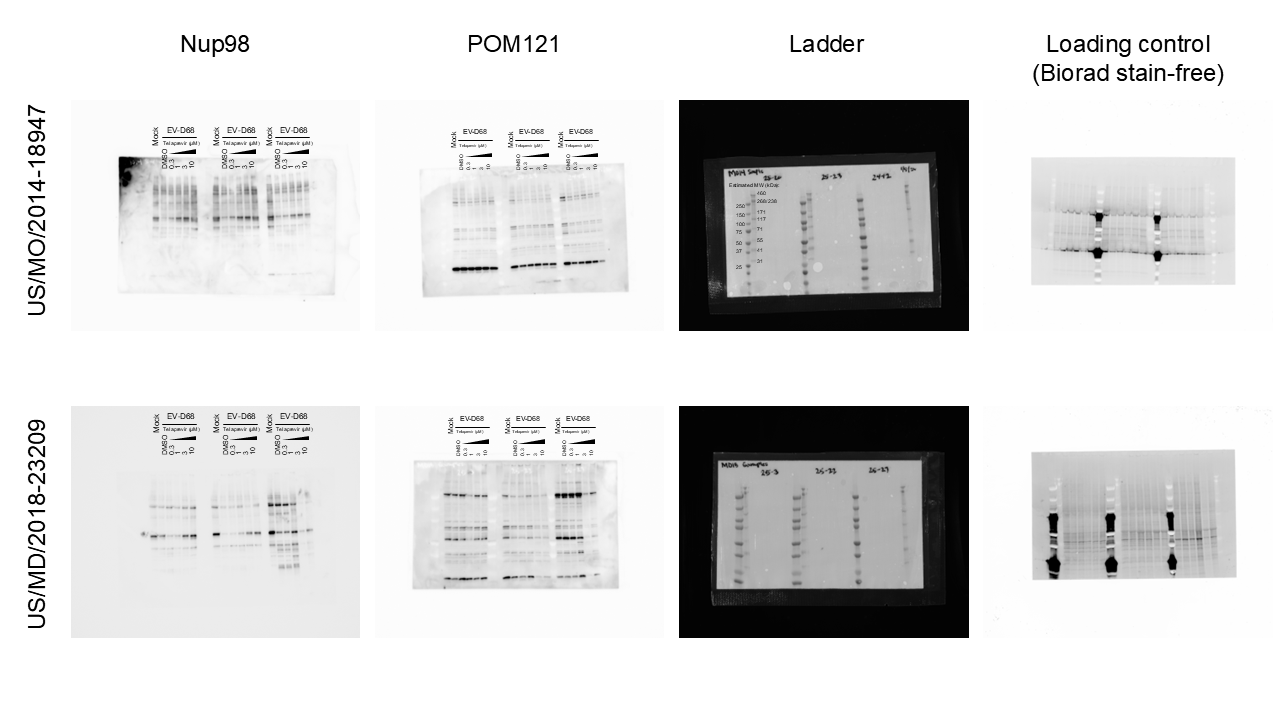

Supplement: Figure 4—source data 1. [file elife-108672-fig4-data1.zip › Fig 4A all blots.tif]

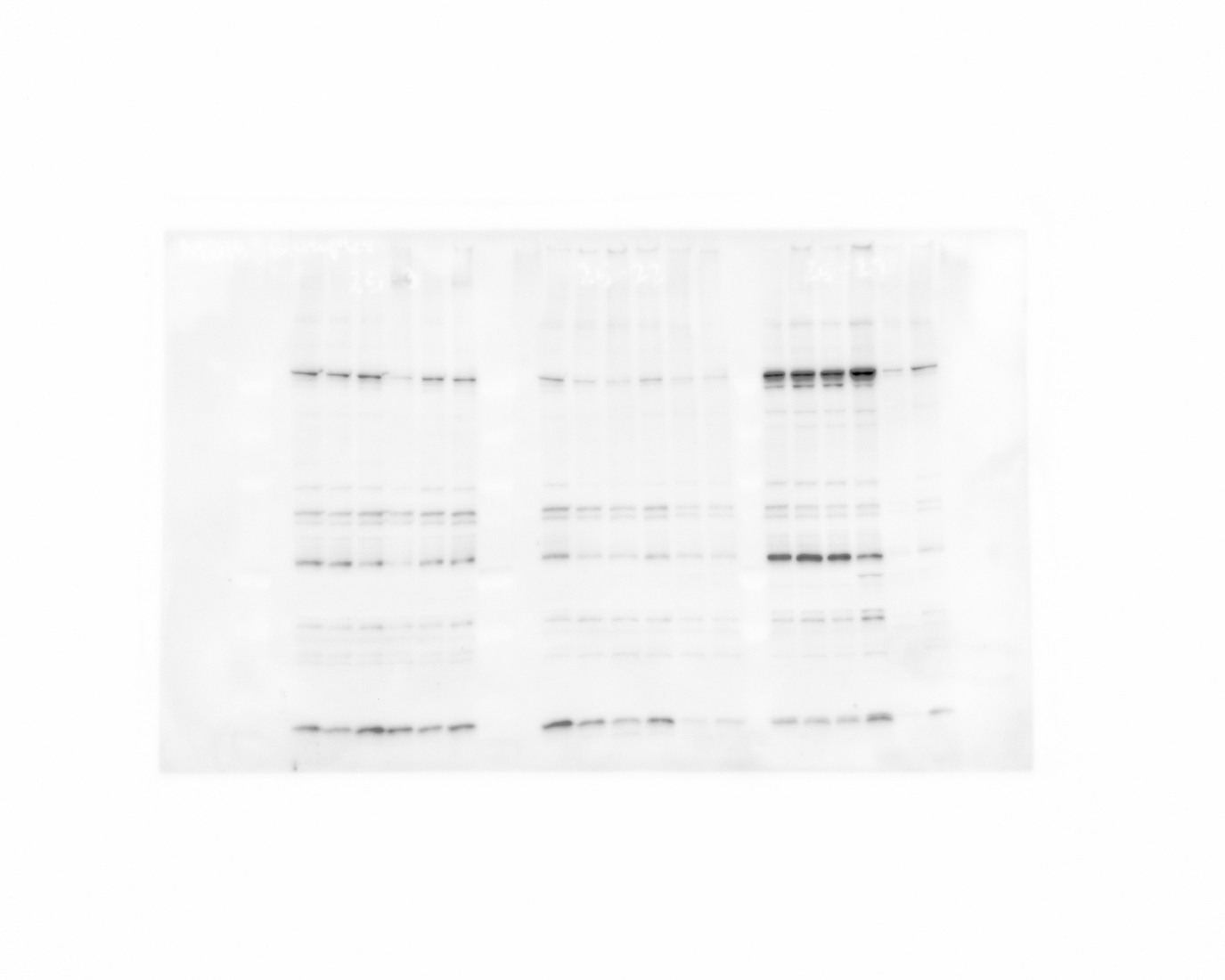

Supplement: Figure 4—source data 2. [file elife-108672-fig4-data2.zip › POM121 MD-2018 for rep 3.tif]

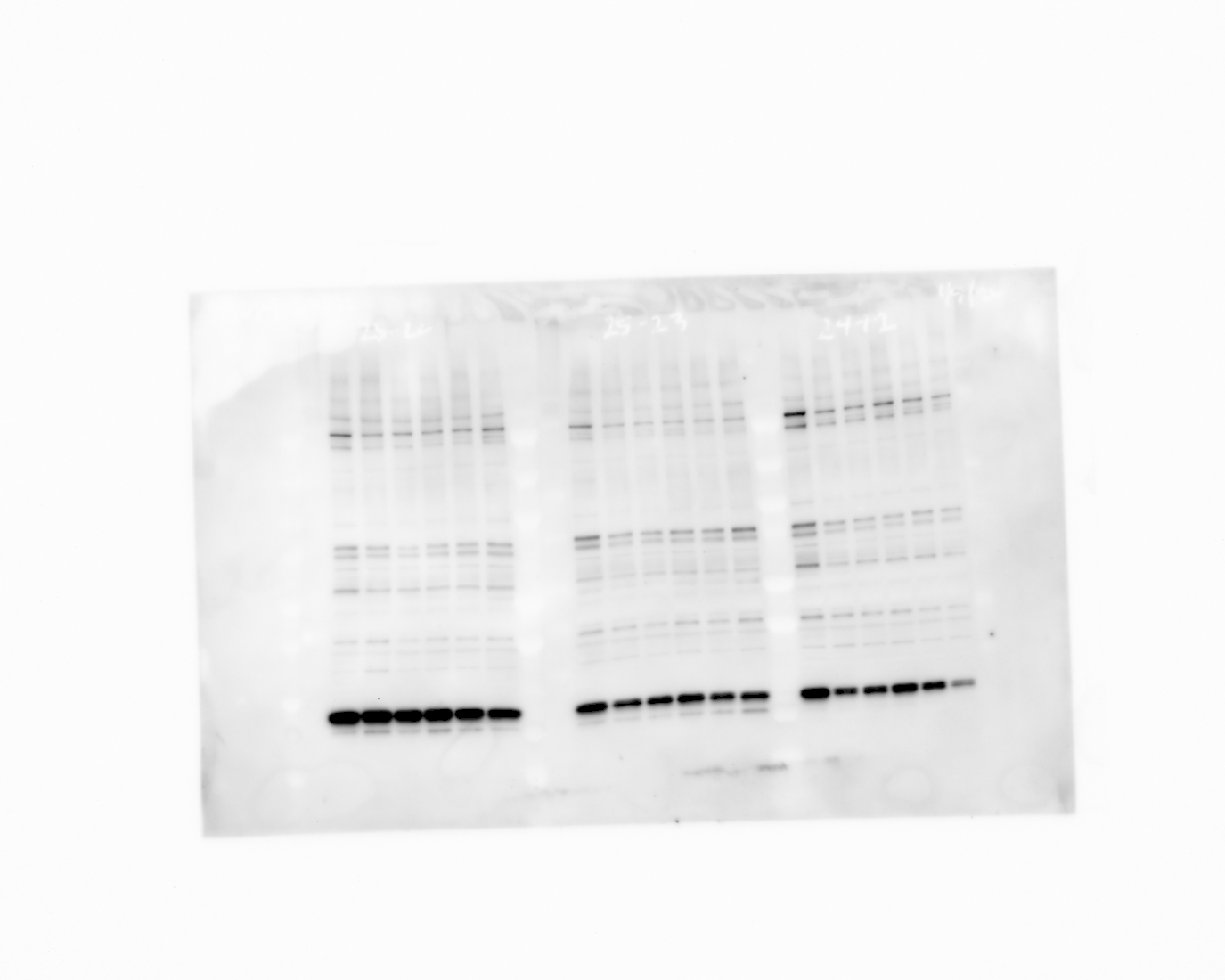

Supplement: Figure 4—source data 2. [file elife-108672-fig4-data2.zip › POM121 MO-2014.tif]

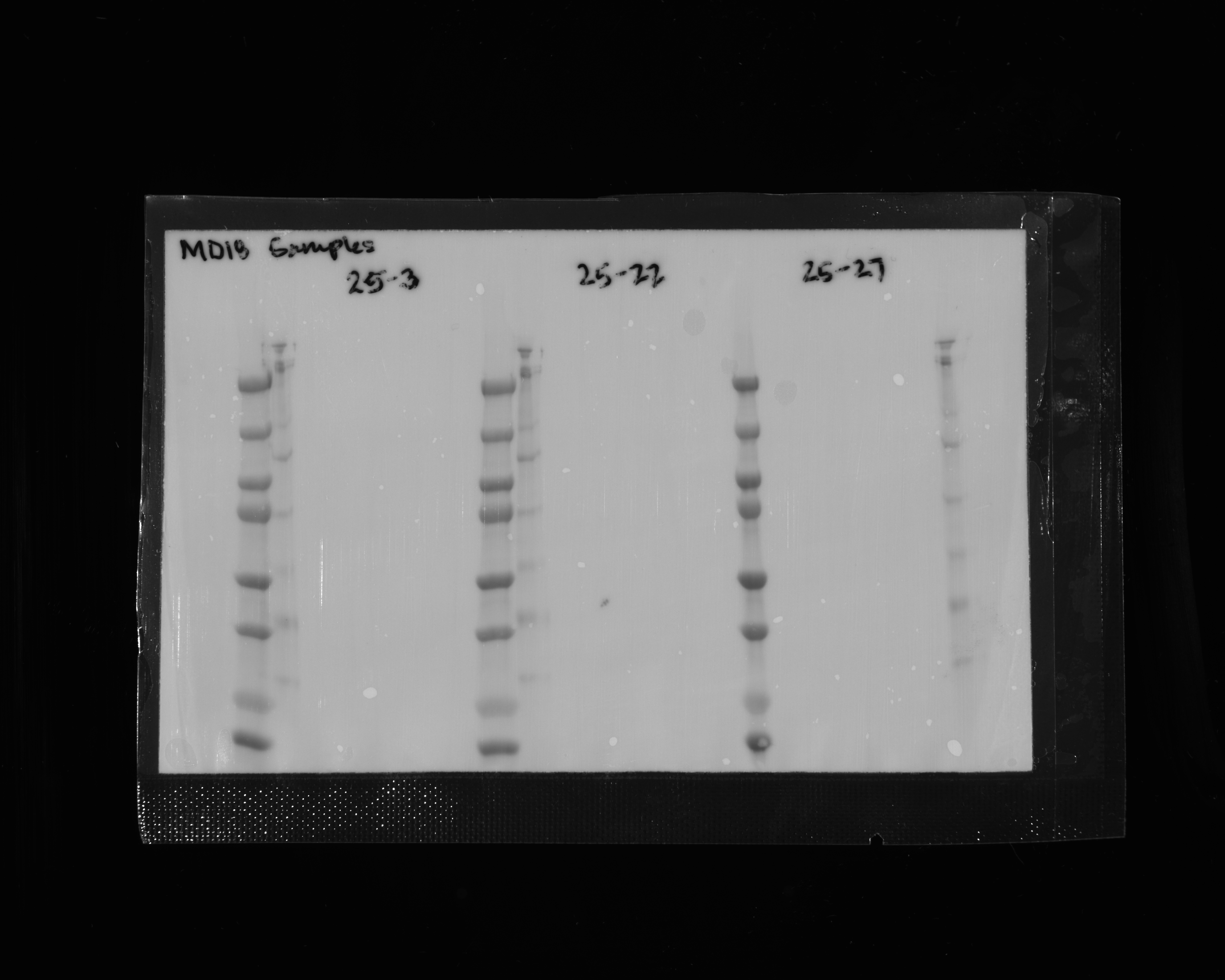

Supplement: Figure 4—source data 2. [file elife-108672-fig4-data2.zip › MD-2018 ladder.tif]

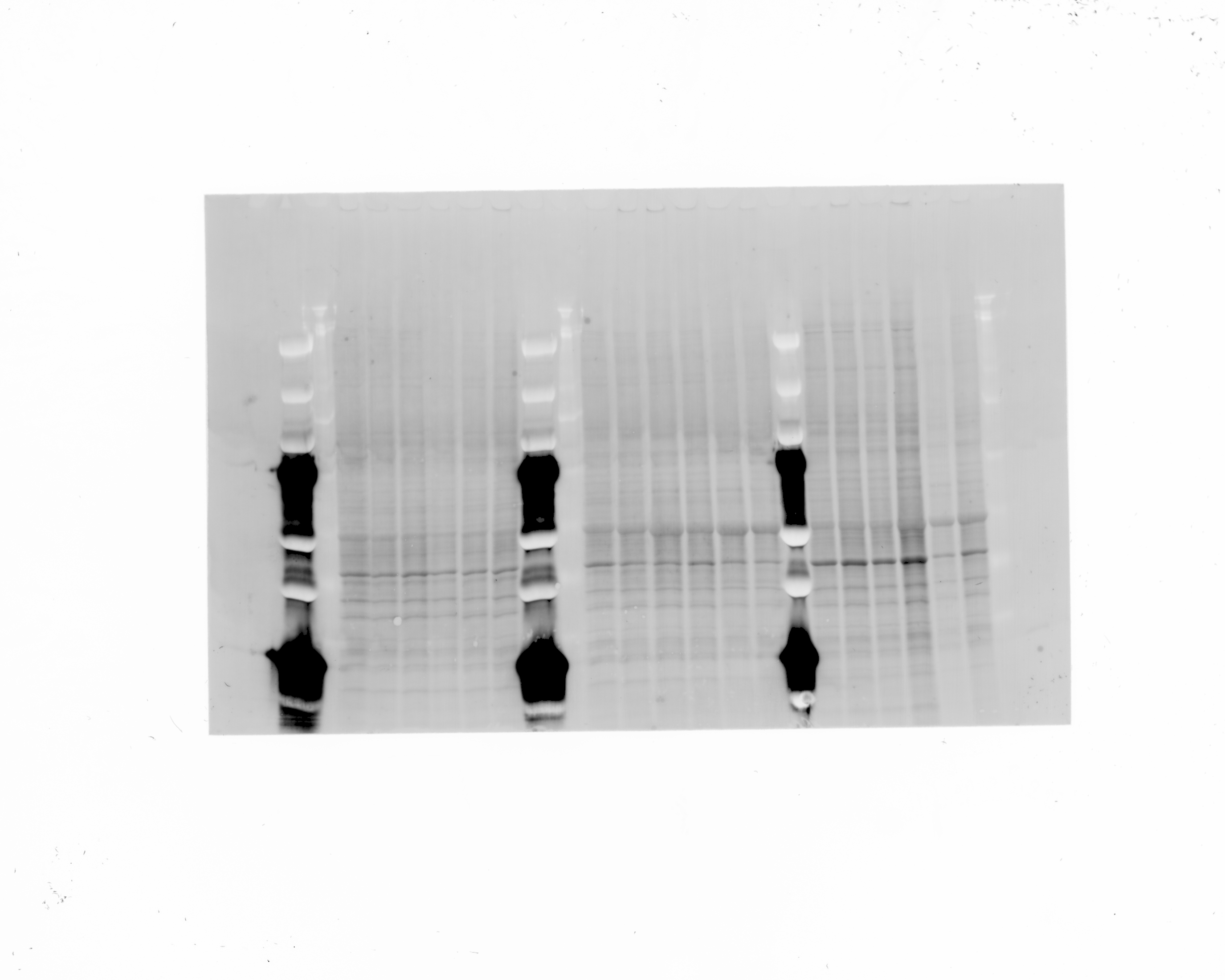

Supplement: Figure 4—source data 2. [file elife-108672-fig4-data2.zip › MD-2018 loading control.tif]
