# Supplementary figures and images for: Enterovirus D68 2A protease causes nuclear pore complex dysfunction and independently contributes to motor neuron toxicity (part 4 of 4)
Source: eLife. 2026 Jun 18;14:RP108672. doi: 10.7554/eLife.108672 (PMC13278737; doi:10.7554/eLife.108672)

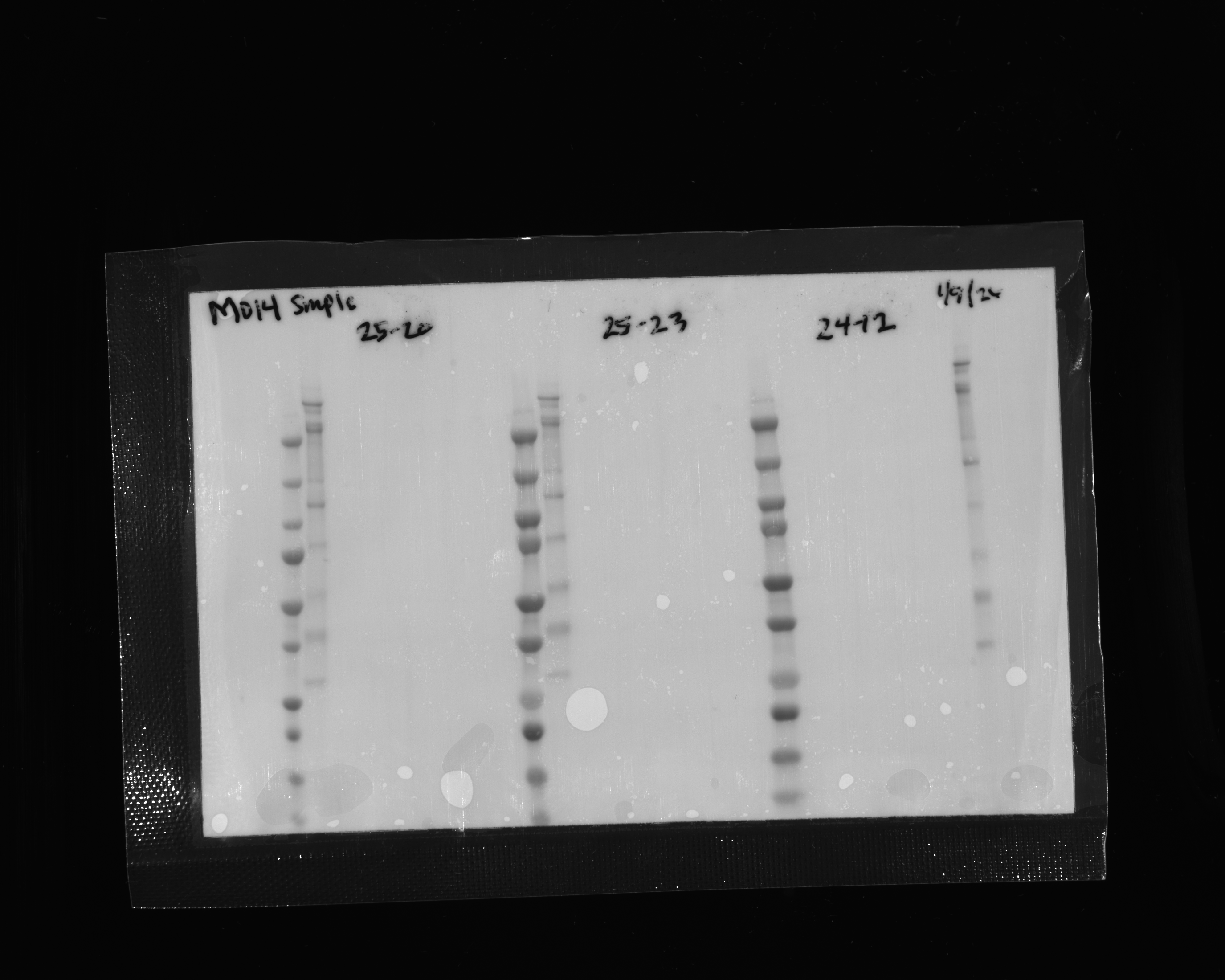

Supplement: Figure 4—source data 2. [file elife-108672-fig4-data2.zip › MO-2014 ladder.tif]

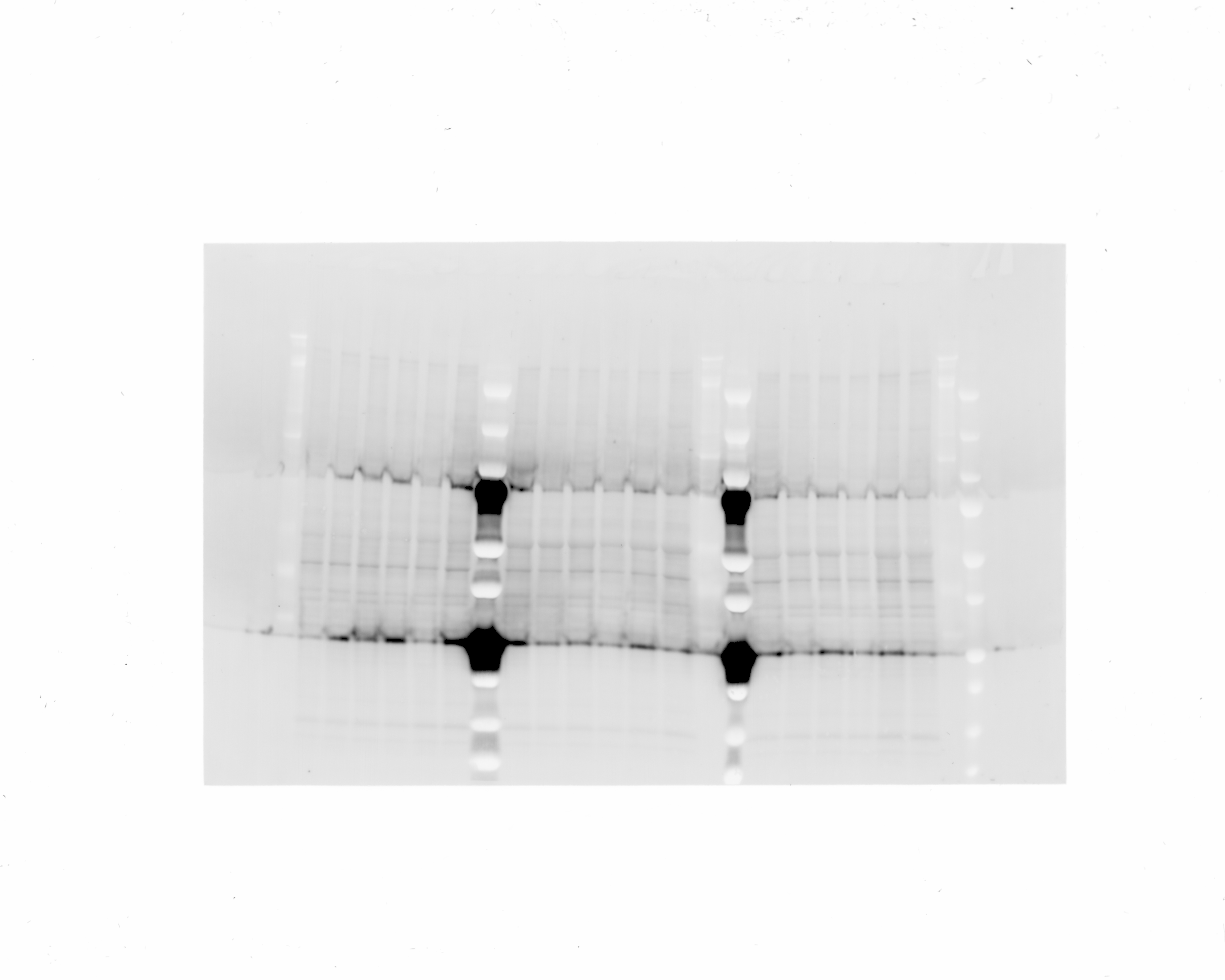

Supplement: Figure 4—source data 2. [file elife-108672-fig4-data2.zip › MO-2014 loading control.tif]

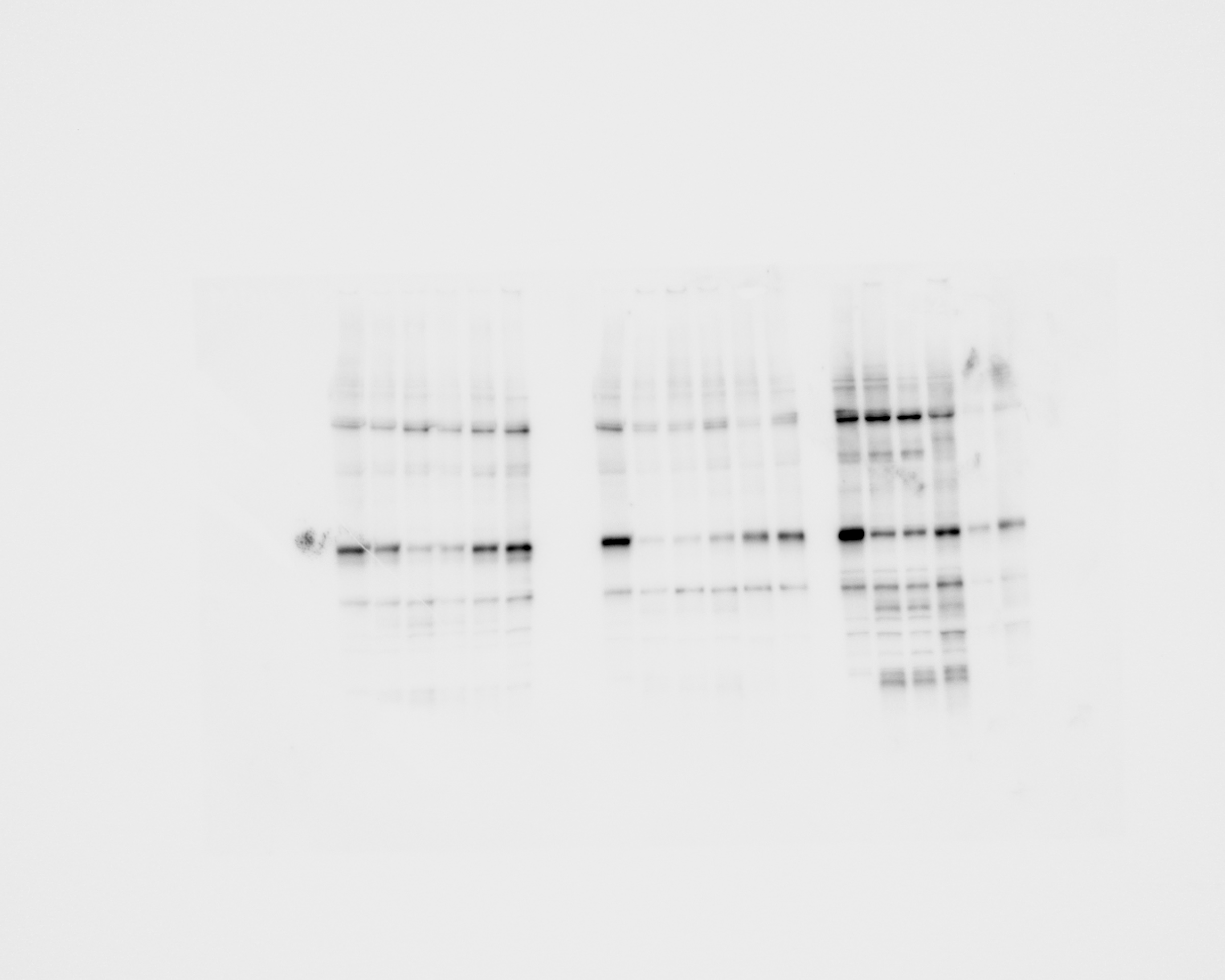

Supplement: Figure 4—source data 2. [file elife-108672-fig4-data2.zip › Nup98 MD-2018.tif]

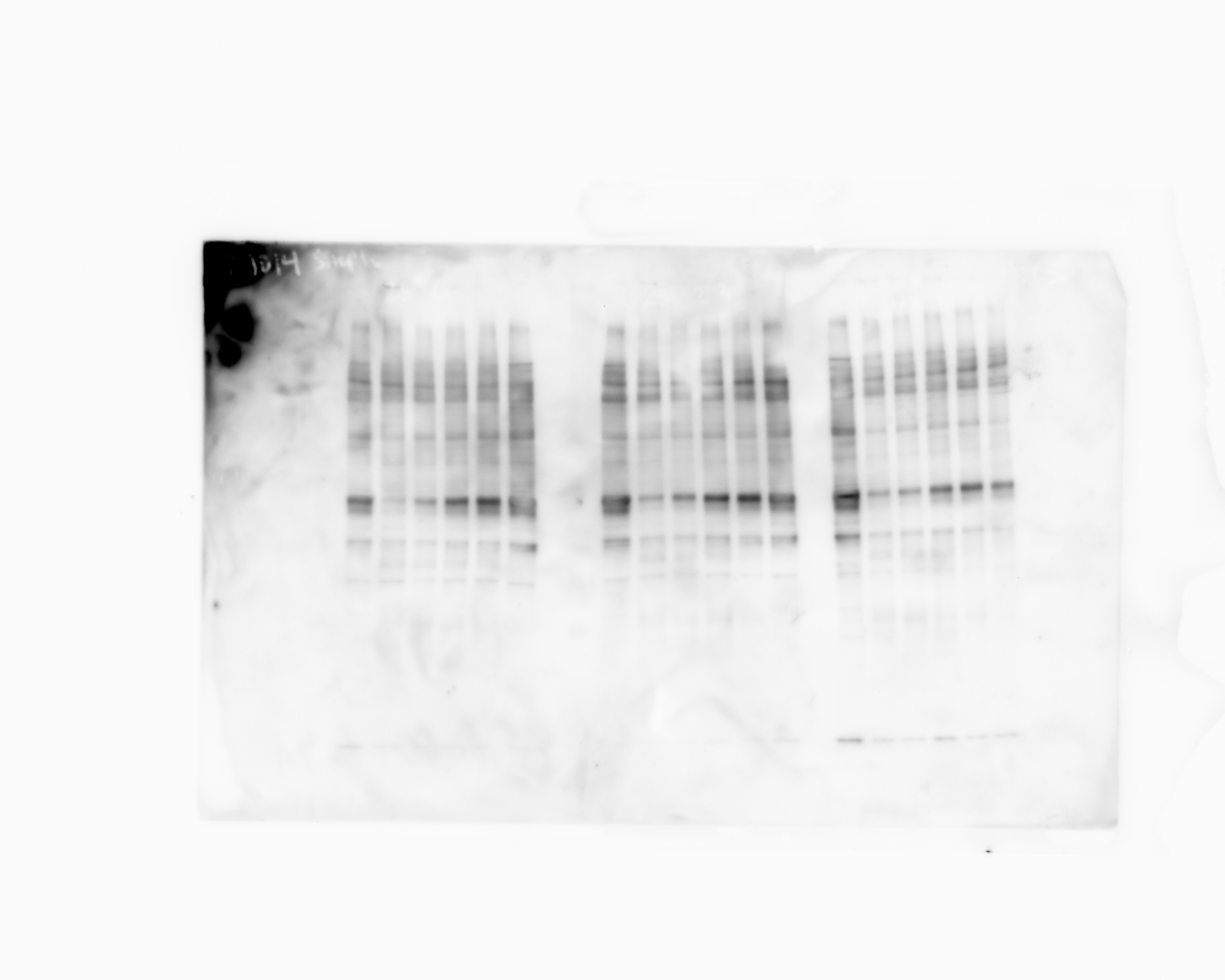

Supplement: Figure 4—source data 2. [file elife-108672-fig4-data2.zip › Nup98 MO-2014.tif]

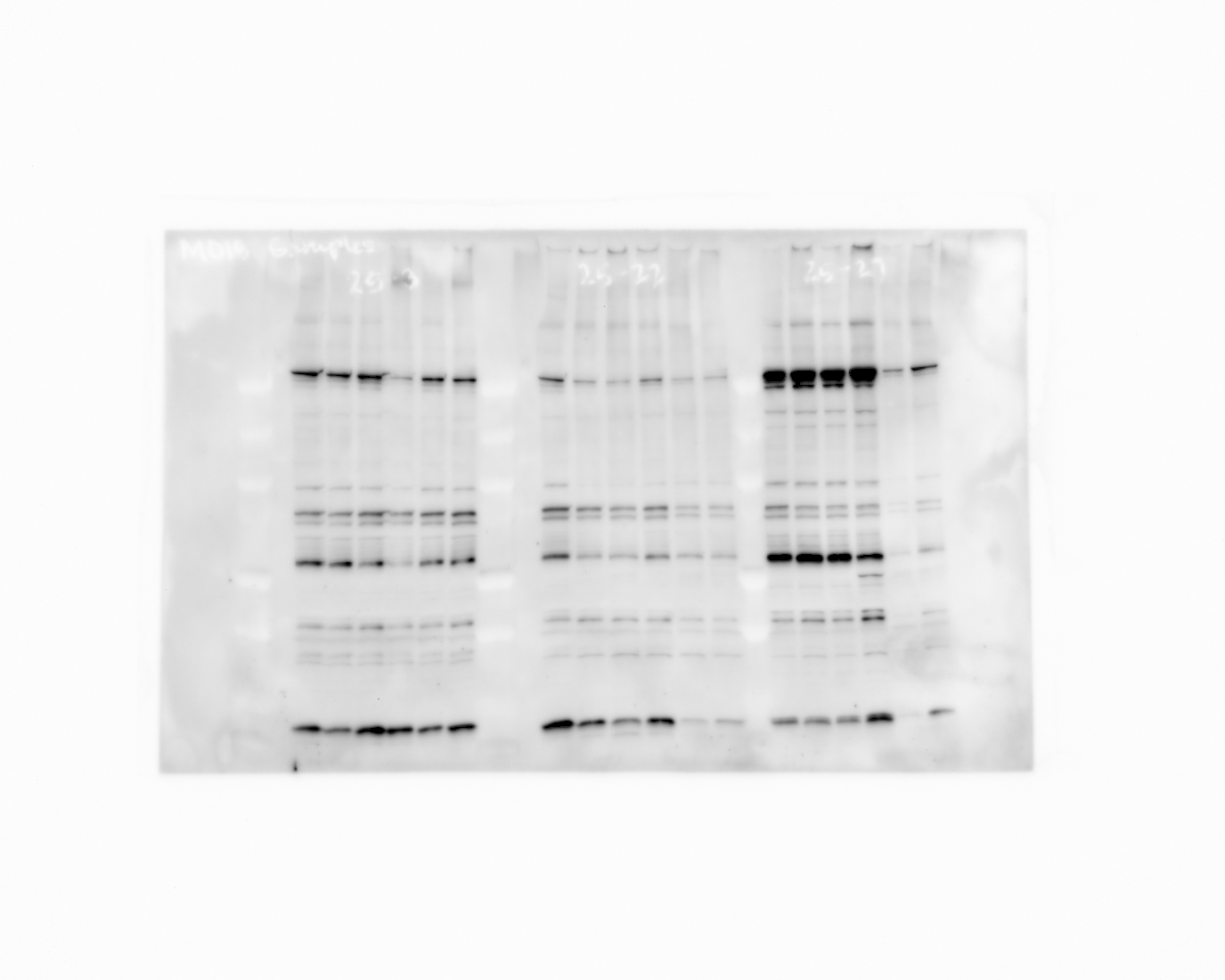

Supplement: Figure 4—source data 2. [file elife-108672-fig4-data2.zip › POM121 MD-2018 (for rep 2).tif]

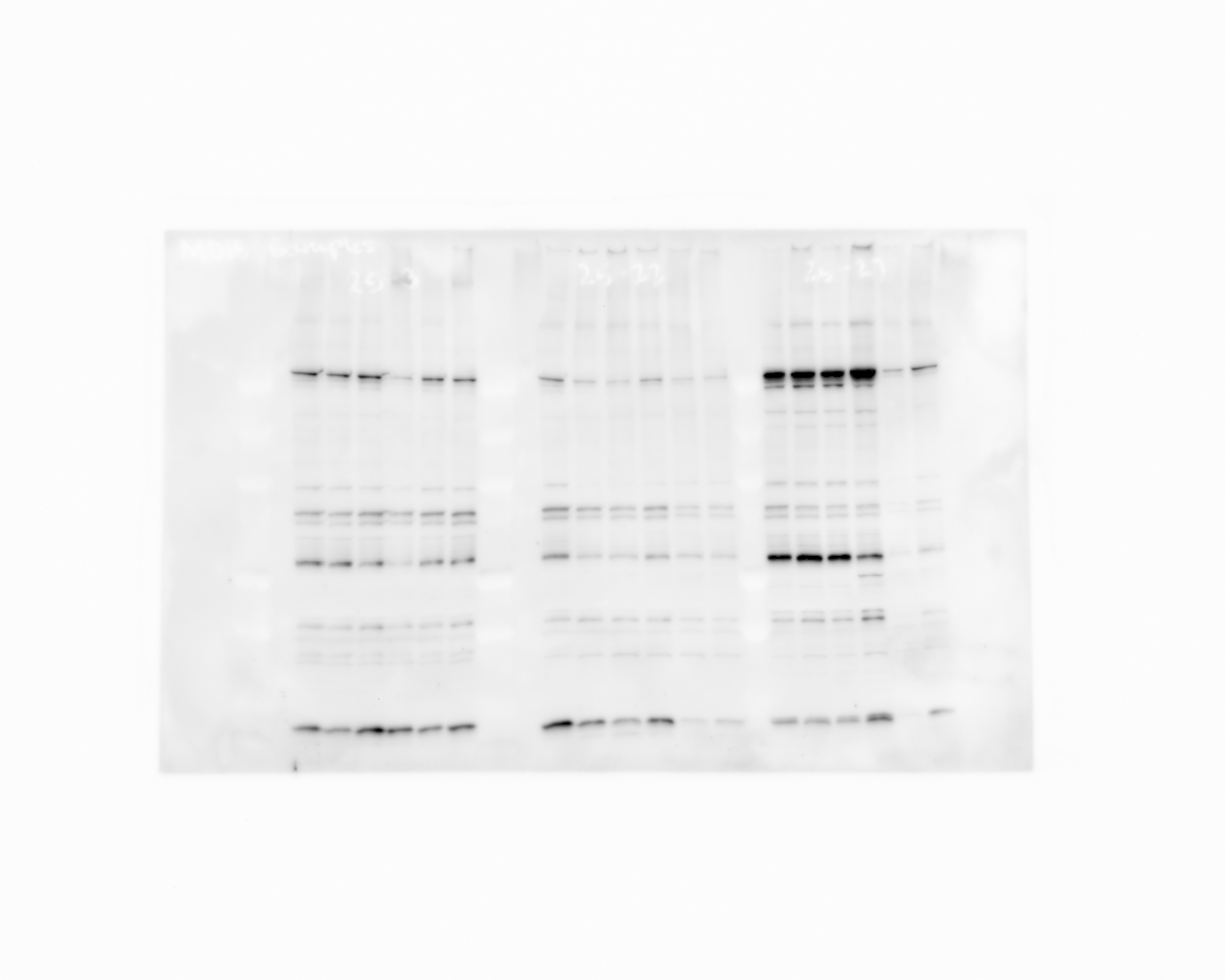

Supplement: Figure 4—source data 2. [file elife-108672-fig4-data2.zip › POM121 MD-2018 for rep 1.tif]
