# Supplementary material for: Enterovirus D68 2A protease causes nuclear pore complex dysfunction and independently contributes to motor neuron toxicity
Source: eLife. 2026 Jun 18;14:RP108672. doi: 10.7554/eLife.108672 (PMC13278737; doi:10.7554/eLife.108672)
Supplement: Peer review history

# Editor report: eLife Assessment

**DOI:** 10.7554/eLife.108672.3.sa0

**Authors:** John W Schoggins

**Affiliations:** The University of Texas Southwestern Medical Center United States

**Keywords:** Solid, Valuable

This **valuable** study examines the cleavage of motor neuron nucleoporins by proteases of enterovirus D68, a pathogen associated with acute flaccid myelitis. The evidence supporting the effects of EV-D68 proteases on nuclear import and export is generally **solid**, as is the independent examination of EV-D68 protease on spinal cord neuron toxicity. The specific conclusions related to RNA export were considered overstated relative to the data presented.

# Referee report: Reviewer #1 (Public review)

**DOI:** 10.7554/eLife.108672.3.sa1

Summary:

Zinn and colleagues investigated the role of proteases 2A and 3C of enterovirus D68 (EVD68), an emerging pathogen associated with outbreaks of acute flaccid myelitis (AFM), a polio-like disease, on the nucleocytoplasmic trafficking in different systems, including human neurons derived from pluripotent cells. They found that 2A specifically cleaved Nup98 and POM121. Using reporter proteins and RNA synthesis and trafficking assays in cells expressing viral proteases, they showed that 2A induces broad loss of the nuclear pore barrier function, but, surprisingly, the RNA export appears to be minimally affected. Since nucleocytoplasmic trafficking defects are known to be associated with neuropatologies, they propose a hypothesis that 2A-dependent cleavage of nucleoporins in motoneurons underlies the development of EVD68-induced AFM. They further show that a 2A-specific inhibitor increases the survival of human neurons differentiated from stem cells upon EVD68 infection.

Strengths:

Use of multiple methods to investigate the effect of 2A and 3C expression on nucleoporin cleavage and nucleocytoplasmic trafficking.

Comments on revisions:

The following issues remain unresolved:

First, the authors still do not show representative images confirming specific nucleoporin degradation (Fig.1), which is the main focus of the work.

Second, the conclusion that 2A-mediated degradation of the nucleo-cytoplasmic barrier does not affect export of the RNA from the nucleus is not supported by the presented data. The representative images shown in Fig 3C do not have the signal for GFP (like in Fig. 2), and therefore it is impossible to see if those cells indeed express EVD68 proteases.

Moreover, to show RNA export, not only the decrease of nuclear EU signal should be quantified, but also the increase of the cytoplasmic signal. The diminishing of the nuclear staining may not necessarily reflect RNA export, but may well be explained by nuclease activity, all the more relevant in cells expressing 2A, where the nuclear-cytoplasmic barrier is disrupted and cytoplasmic nucleases may enter the nucleus.

The same applies to images in Fig. 3D. There are no markers of infection; moreover, the experiment description indicates that EU labeling began at 24 h post-infection with an MOI of 5, i.e., essentially all cells should have been infected. This is difficult to believe as the replication cycle of most EVD68 strains in HeLa cells is no longer than 12 h, yet the images do not show any signs of CPE, and demonstrate a strong EU signal, inconsistent with the expected inhibition of nuclear transcription, a known attribute of enterovirus infections.

The claim that nuclear transcription and RNA export remain unaffected in conditions of 2A-mediated disruption of the nucleo-cytoplasmic barrier is very strong and requires equally strong evidence.

# Referee report: Reviewer #2 (Public review)

**DOI:** 10.7554/eLife.108672.3.sa2

Summary:

This manuscript investigates the role of EV-D68 proteases 2A and 3C in nuclear pore complex (NPC) dysfunction and their contribution to motor neuron toxicity. The authors demonstrate that both proteases cleave only a limited number of nucleoporins, with 2A^pro showing the strongest impact by inhibiting nuclear import and export of proteins and disrupting NPC permeability without affecting RNA export. Importantly, treatment with the 2A^pro inhibitor telaprevir reduced neuronal cell death in a dose-dependent manner, achieving neuroprotection at concentrations below those required to inhibit viral replication. The study addresses a relevant mechanism underlying EV-D68-induced neuropathology and explores a potential therapeutic intervention.

# Referee report: Reviewer #3 (Public review)

**DOI:** 10.7554/eLife.108672.3.sa3

Summary:

The author showed expression of the viral proteases 2Apro and 3Cpro of EV-D68, which cleaved specific components of the nuclear pore complex (Nup98 and POM121 by 2Apro), and 2A but not 3C expression altered nuclear import and export. Similar nucleocytoplasmic transport deficits are observed in EV-D68-infected RD cells and iPSC-derived motor neurons (diMNs). 2A inhibitor telaprevir partially rescued the nucleocytoplasmic transport deficits and suppressed neuronal cell death after infection. While it's clear that 2A can cleave NPC proteins and affect nuclear transport, the link to neurotoxicity after EV-D68 infection is less convincing.

This study opens up a very intriguing hypothesis: that EV-D68 2Apro could be directly responsible for motor neuron cell death, mediated by POM121 and possibly Nup98 cleavage, that ultimately results in paralysis known as acute flaccid myelitis. This hypothesis notably does run counter to other published data showing that human neuronal organoids derived from iPSCs can support productive EV-D68 infection for weeks without cell death and that EV-D68-infected mice can have paralysis prevented by depletion of CD8 T cells, still with EV-D68 infection of the spinal cord. However, even if 2Apro is not ultimately responsible for motor neurons dying in human infections, that does not exclude the possibility that cleavage of nups could still disrupt motor neuron function. Notably, most children with AFM have some amount of motor function return after their acute period of paralysis, but most still have some residual paralysis for years to life. It is possible that 2A pro could mediate the acute onset of weakness, while T cells killing neurons could determine the amount of long-term, residual paralysis.

Strengths:

The characterization of nuclear pore complex components that appear to be targets of both poliovirus and EV-D68 proteases is quite thorough and expansive, so this data set alone will be useful for reference to the field. And the process by which the authors narrowed their focus to EV-D68 2Apro reducing Nup98 and POM121 as consequential to both import and export of nuclear cargo but not RNA was technically impressive, thorough, and convincing. As will be detailed below, when the authors move from studying over-expressed proteases in transformed cell lines to studying actual virus infection in both transformed cell lines and iPSC-derived neurons, some of the data only indirectly support their conclusions; however, the quality of the experiments performed is still high. So even if the claim that 2Apro causes neurotoxicity is circumstantial, the data certainly are intriguing and certainly justify further study of the effects of EV-D68 2Apro on the NPC and how this impacts pathogenesis. This is a convincing start to an intriguing line of inquiry.

Comments on revisions:

The authors have returned a stronger revised manuscript, being responsive to most of the combined reviewers' comments. It was especially important to add the clarity and specificity that the data in this manuscript did not establish a direct link for 2Apro causing AFM. The authors have clarified this language adequately, such that it is appropriate to remove the "incomplete" portion of the short assessment as they have requested. Adding in experiments with EV-D68 virus infection to complement their work with recombinant proteases also strengthened their conclusions.

There are still some areas where discrepancies remain, although these are minor and can mostly be acknowledged as limitations of their approach rather than needing more experiments, unless the authors choose to do the additional experiments. To try to make this understandable, I have copied from the rebuttal letter (*) original comment, (**) author's rebuttal, and (***) a reply to the rebuttal:

(*)(2) Telaprevir was able to rescue nucleocytoplasmic transport in RD cells at low concentrations (Figure 4A). It is not shown if this correlates with its antiviral effect in RD cells, or could this correlate with inhibition of 2A cleavage of Nup98 or POM121, which is never measured.

(**) In the aforementioned new experiment in Figure 4A, we have also included a dose-response curve for telaprevir showing its inhibition of POM121 and Nup98 cleavage.

(***) Fig.4A is in diMN not RD cells. The EC50 of telaprevir could be very different in RD cell vs diMNs. This question remains unanswered.

(*) (3) Building off of the prior point, the authors' claim that the neuroprotective effect of telaprevir is independent of its antiviral effect is not well-founded. Figure 4E (neuroprotection) was done with MOI 5, and Figure 4G (virus growth) was MOI 0.5. Telaprevir neuroprotection is not shown at MOI 0.5, nor is the neuroprotective effect correlated with inhibition of 2A cleavage of Nup98 or POM121.

(**) The selection of MOIs for these two experiments was limited by technical considerations. If the viral growth curve were to be performed at MOI 5, it would be confounded by cell death. Further, a low MOI is required in order to allow multiple rounds of infection, and is therefore more sensitive for assaying the effect of telaprevir on viral replication. On the other hand, at MOI 0.5 diMN death is very gradual, and the neuroprotection assay we would have lacked the statistical power to determine whether a rescue of this small magnitude of toxicity is significant. The EC50 of telaprevir is not expected to vary at different MOIs.

(***) This should be discussed in the Discussion as a limitation of the experiment.

(**) We have also now correlated the inhibition of 2Apro cleavage of Nup98 and POM121 with the neuroprotective effect at comparable concentrations of telaprevir, as described above.

(***) Unless you quantify this, my eye disagrees with you. In Fig.4A, cleavage of NUP98 is rescued by 3uM telaprevir, but that does not seem to be the case for POM121.

Additionally, in Fig. 4D, why is only NLS but not NES is impaired in diMN? This should be discussed.

# Author comment: Author response

**DOI:** 10.7554/eLife.108672.3.sa4

**Authors:** Katrina M Zinn, Mathew W McLaren, Michael T Imai, Malavika M Jayaram, Jeffery D Rothstein, Matthew J Elrick

**Affiliations:** Hugo W. Moser Research Institute, Michael V. Johnston Center for Developmental Neuroscience, Kennedy Krieger Institute Baltimore, MD United States; Hugo W. Moser Research Institute, Michael V. Johnston Center for Developmental Neuroscience, Kennedy Krieger Institute Baltimore, MD United States; Hugo W. Moser Research Institute, Michael V. Johnston Center for Developmental Neuroscience, Kennedy Krieger Institute Baltimore, MD United States; Hugo W. Moser Research Institute, Michael V. Johnston Center for Developmental Neuroscience, Kennedy Krieger Institute Baltimore, MD United States; Johns Hopkins University Baltimore United States; Hugo W. Moser Research Institute, Michael V. Johnston Center for Developmental Neuroscience, Kennedy Krieger Institute Baltimore, MD United States

The following is the authors’ response to the original reviews.

**eLife Assessment**

This valuable study examines the cleavage of motor neuron nucleoporins by proteases 2A and 3C of enterovirus D68, a pathogen associated with acute flaccid myelitis. The evidence supporting the effects of EV-D68 proteases on nuclear import and export is solid and confirms previous results on the specific targeting of nucleoporins by proteases from other enteroviruses. However, the claim that cleavage of nucleoporins by EV-D68 2A is neurotoxic, though intriguing, is incomplete, as the evidence is largely indirect.

We appreciate that the reviewers highlighted multiple strengths of manuscript, including its detailed mechanistic dissection of the disrupted composition and function of the nuclear pore complex during EV-D68 infection, the finding that the viral 2A protease is toxic to motor neurons, and that several novel hypotheses on the pathogenesis of acute flaccid myelitis that are raised by our work.

It appears that two independent eLife Assessments were made regarding the strength of evidence in our manuscript. The evidence supporting the impact of EV-D68 proteases on the NPC was felt to be solid.

A second assessment was made as to whether our data support that “the cleavage of nucleoporins by EV-D68 2A is neurotoxic”. We would like to clarify that we did not intend to make this second claim in our manuscript and thought that we had been careful not to do so. In response to reviewer and editorial feedback, we have edited the text to improve the clarity on this issue. Although our data show that 2A^pro^ is toxic to motor neurons, it cannot yet be determined whether this toxicity is mediated via 2A^pro^’s effects on the NPC. That is a logical hypothesis that arises from our manuscript, which we are testing through ongoing work that will require a significant volume of experiments that are outside the scope of the present study. We view this manuscript as an important first step towards a comprehensive understanding of the role of the 2A protease in the pathogenesis of AFM. Please see the response to point # 3 of Reviewer 2 below for a more detailed discussion of this issue and the changes we have made to the text in response. We respectfully request that a judgement on the role of nucleoporin cleavage as the mechanism of neurotoxicity not be included in the eLife Assessment.

Also in response to reviewer feedback that our data was too reliant on the expression of recombinant viral proteins in isolation, we have added additional experiments extending our results into the context of live virus infection of cell lines and motor neurons. We feel that our revised manuscript has been improved as a result of the reviewers’ and editor’s input, and provides strong support for the following claims: (1) NPC composition and function is disrupted during EV-D68 infection, (2) 2A^pro^ is primarily responsible for functional disruption, and (3) 2A^pro^ is neurotoxic.

We appreciate your review of this revised manuscript. Detailed responses to each of the reviewers’ comments are provided below.

**Public Reviews:**

**Reviewer #1 (Public review):**

Summary:

Zinn and colleagues investigated the role of proteases 2A and 3C of enterovirus D68 (EVD68), an emerging pathogen associated with outbreaks of acute flaccid myelitis (AFM), a polio-like disease, on the nucleocytoplasmic trafficking in different systems, including human neurons derived from pluripotent cells. They found that 2A specifically cleaved Nup98 and POM121. Using reporter proteins and RNA synthesis and trafficking assays in cells expressing viral proteases, they showed that 2A induces broad loss of the nuclear pore barrier function, but, surprisingly, the RNA export appears to be minimally affected. Since nucleocytoplasmic trafficking defects are known to be associated with neuropatologies, they propose a hypothesis that 2A-dependent cleavage of nucleoporins in motoneurons underlies the development of EVD68-induced AFM. They further show that a 2A-specific inhibitor increases the survival of human neurons differentiated from stem cells upon EVD68 infection.

Strengths:

Use of multiple methods to investigate the effect of 2A and 3C expression on nucleoporin cleavage and nucleocytoplasmic trafficking.

We thank the reviewer for detailed and accurate review of our manuscript and recognition of these strengths.

Weaknesses:

Overall, the paper follows multiple others that extensively investigated the cleavage of nucleoporins by enterovirus 2As, so the results are of limited novelty. The hypothesis that infection of motoneurons is the cause of EVD68-induced neurological complications so far is supported by only one autopsy report. Other data suggest that infection of other cell types, such as astrocytes, and/or inflammatory cell infiltration in the CNS, are likely to be responsible for the symptoms. In any case, the claim that EVD68 is specifically neurotoxic because of the 2A-dependent cleavage of nucleoporins in neurons is unfounded, as the virus will be just as "toxic" for other infected cell types.

While we agree that other papers have investigated this pathway in other enteroviruses, we note that our work is the first to do so in Enterovirus D68 and the most comprehensive study, in terms of the number of nucleoporins studied. As we reviewed in paragraph 5 of the introduction section, the activities of enterovirus proteases against specific nucleoporins varies from strain to strain, and is important to understand any strain-specific effects before determining whether this pathway is relevant to toxicity in AFM.

The infection of motor neurons is strongly supported not only by the aforementioned autopsy data [1], but also by mouse model data demonstrating replication of EV-D68 within motor neurons in the anterior horn of the spinal cord.[2] There are also numerous reports of electromyography and nerve conduction studies from human AFM patients demonstrating that the site of pathology is the spinal motor neuron.[3-10]

By contrast, infection of astrocytes has been demonstrated only in primary murine astrocyte cultures in which no neurons were present [11]. Therefore, while the available data suggest that EV-D68 infection of astrocytes is possible, in the *in vivo* context of human and mouse spinal cord, tropism to motor neurons appears to be preferential. The relative toxicity of neuron-autonomous vs non-autonomous processes such as glial dysfunction and inflammatory cell infiltration remain to be elucidated, and are not mutually exclusive.

The paper also requires a more convincing presentation of the data.

We are uncertain what other specific changes the reviewer would like to see based on this comment, but feel that the revisions have improved the presentation of the data.

**Reviewer #2 (Public review):**

Summary:

This manuscript investigates the role of EV-D68 proteases 2A and 3C in nuclear pore complex (NPC) dysfunction and their contribution to motor neuron toxicity. The authors demonstrate that both proteases cleave only a limited number of nucleoporins, with 2A^pro showing the strongest impact by inhibiting nuclear import and export of proteins and disrupting NPC permeability without affecting RNA export. Importantly, treatment with the 2A^pro inhibitor telaprevir reduced neuronal cell death in a dose-dependent manner, achieving neuroprotection at concentrations below those required to inhibit viral replication. The study addresses a relevant mechanism underlying EV-D68-induced neuropathology and explores a potential therapeutic intervention.

Strengths:

(1) Provides significant mechanistic insight into how EV-D68 proteases alter NPC function and contribute to neuronal toxicity.

(2) The use of recombinant 2A and 3C proteins allows clear dissection of the specific contribution of each protease.

(3) Demonstrates a therapeutic effect of telaprevir, with neuroprotection independent of viral replication inhibition, adding translational value to the findings.

(4) The topic is highly relevant given the association of EV-D68 with acute flaccid myelitis.

We thank the reviewer for their insightful comments and recognition of these strengths in our study.

Weaknesses:

(1) Most experiments were performed with recombinant proteases, lacking validation in the context of viral infection, where both proteases act simultaneously.

In response to this concern, we have added additional experiments in the context of viral infection. We show that POM121 and Nup98 are also cleaved in motor neurons infected with EV-D68 and that their cleavage is inhibited by telaprevir (Fig 4A). We also repeated the EU pulse-chase RNA export assay in EV-D68-infected RD cells and again found no effect on RNA export (Fig 3D-E).

(2) The conclusion that RNA export is unaffected requires confirmation during actual infection.

As above, we have repeated this experiment in EV-D68 RD cells, showing no effect of EV-D68 infection on RNA export.

(3) The reduction of neurotoxicity by telaprevir does not fully demonstrate that the protective effect is solely mediated through NPC preservation; additional analyses of eIF4G cleavage, nucleoporin integrity, and stress granules are needed.

We agree that while the evidence in our manuscript raises the hypothesis that telaprevir-mediated neuroprotection is mediated via NPC preservation, it does not fully demonstrate this to be the case. As discussed above, we have been careful to state only the following conclusions: (1) NPC composition and function is disrupted during EV-D68 infection, (2) 2A^pro^ is primarily responsible for functional disruption, and (3) 2A^pro^ is neurotoxic.

Future work will determine the extent to which NPC dysfunction contributes to 2A^pro^-mediated motor neuron toxicity versus other potential targets of 2A^pro^, as suggested by the reviewer. This work is already underway in our lab and it is clear to us the additional experiments required will be extensive, likely 1-2 additional manuscripts. These experiments are therefore beyond the scope of the present study, which represents a key first step in this line of inquiry.

We specifically acknowledged in the Discussion that “A significant limitation of our study, however, is that we cannot exclude potentially toxic effects of 2A^pro^ on aspects of host neuronal biology aside from the NPC.” We have also made the following adjustments to the text to make it more clear that this remains an open question:

Change the title to more clearly separate the effects of 2Apro on NPC function and motor neuron toxicity as independent events: “Enterovirus D68 2A protease causes nuclear pore complex dysfunction and independently contributes to motor neuron toxicity”

In the abstract, shortened the following sentence: “We therefore sought to determine the impact of EV-D68 proteases on NPC composition and function” to avoid any implicit connection that a mechanistic link has been established between these two concepts. Neurotoxicity is now introduced later in the abstract by saying “Independently, we show…” instead of “We further show…”

Removed language in the last paragraph of the Results section that may have been construed to suggest a mechanistic linkage: “Because similar deficits have been reported to contribute to neurotoxicity in neurodegenerative disease…” and simply stated “We next sought to determine the extent to which 2Apro activity independently contributes to motor neuron injury during EV-D68 infection.”

Edited the opening sentence of the discussion, where it was ambiguous whether the word “their” was referring to the enterovirus protease (which was our intent) or to NPC disruption as the cause of motor neuron toxicity. We removed the discussion of toxicity from this paragraph entirely to remove such confusion.

Edited the final paragraph of the discussion to include “We have also demonstrated that 2A^pro^ activity contributes to nucleocytoplasmic transport dysfunction and separately to cell death in motor neurons infected with EV-D68”. We then go on to discuss the hypothesis that this toxicity might be mediated partially or entirely through NPC dysfunction, and propose that this be a focus of further study.

(4) The study would be strengthened by including another 2A inhibitor (e.g., boceprevir) to confirm the specificity of telaprevir's protective effects.

While we would like to be able to include multiple pharmacologic inhibitors of 2A^pro^, unfortunately telaprevir is the only known inhibitor of EV-D68 2A^pro^. The same study that identified telaprevir as an EV-D68 2A^pro^ inhibitor also evaluated boceprevir and determined that its inhibitory activity against 2A^pro^ is minimal [12].

**Reviewer #3 (Public review):**

Summary:

The author showed expression of the viral proteases 2Apro and 3Cpro of EV-D68, which cleaved specific components of the nuclear pore complex (Nup98 and POM121 by 2Apro), and 2A but not 3C expression altered nuclear import and export. Similar nucleocytoplasmic transport deficits are observed in EV-D68-infected RD cells and iPSC-derived motor neurons (diMNs). 2A inhibitor telaprevir partially rescued the nucleocytoplasmic transport deficits and suppressed neuronal cell death after infection. While it's clear that 2A can cleave NPC proteins and affect nuclear transport, the link to neurotoxicity after EV-D68 infection is less convincing.

This study opens up a very intriguing hypothesis: that EV-D68 2Apro could be directly responsible for motor neuron cell death, mediated by POM121 and possibly Nup98 cleavage, that ultimately results in paralysis known as acute flaccid myelitis. This hypothesis notably does run counter to other published data showing that human neuronal organoids derived from iPSCs can support productive EV-D68 infection for weeks without cell death and that EV-D68-infected mice can have paralysis prevented by depletion of CD8 T cells, still with EV-D68 infection of the spinal cord. However, even if 2Apro is not ultimately responsible for motor neurons dying in human infections, that does not exclude the possibility that cleavage of nups could still disrupt motor neuron function. Notably, most children with AFM have some amount of motor function return after their acute period of paralysis, but most still have some residual paralysis for years to life. It is possible that 2A pro could mediate the acute onset of weakness, while T cells killing neurons could determine the amount of long-term, residual paralysis.

We thank the reviewer for their thoughtful comments. As discussed above, we agree that the present data demonstrate that 2A^pro^ causes NPC dysfunction and is toxic in motor neurons, but has not proven that the mechanism of neurotoxicity is via NPC dysfunction.

We appreciate the commentary on novel hypotheses opened by our work. Our recent thinking on this topic has been similar and we look forward to addressing these ideas further in future studies. Motor neuron dysfunction and motor neuron death may ultimately prove to have separate causes. The infection of motor neurons is likely the initiating event, with multiple downstream consequences which may be neuron-autonomous, or mediated by glial and inflammatory responses, or a mixture thereof.

Strengths:

The characterization of nuclear pore complex components that appear to be targets of both poliovirus and EV-D68 proteases is quite thorough and expansive, so this data set alone will be useful for reference to the field. And the process by which the authors narrowed their focus to EV-D68 2Apro reducing Nup98 and POM121 as consequential to both import and export of nuclear cargo but not RNA was technically impressive, thorough, and convincing. As will be detailed below, when the authors move from studying over-expressed proteases in transformed cell lines to studying actual virus infection in both transformed cell lines and iPSC-derived neurons, some of the data only indirectly support their conclusions; however, the quality of the experiments performed is still high. So even if the claim that 2Apro causes neurotoxicity is circumstantial, the data certainly are intriguing and certainly justify further study of the effects of EV-D68 2Apro on the NPC and how this impacts pathogenesis. This is a convincing start to an intriguing line of inquiry.

We appreciate the reviewer’s recognition of our comprehensive evaluation of NPC disruption and our approach to arriving at a mechanistic understanding of this process. We agree with the reviewer’s viewpoint that the present study represents a beginning, rather than a conclusive end to this line of inquiry. For technical reasons, we were able to achieve more rigorous and mechanistic data in cell lines expressing recombinant proteins than in neurons infected with live virus. In response the reviewers’ comments, as described above, we have added additional experiments in this revision in which we further evaluate nucleoporin cleavage and RNA export during live virus infection, and performed these experiments in iPSC-derived neurons whenever it was technically feasible to do so.

Weaknesses:

This study falls a bit shy of actually showing that 2Apro effects are causing motor neuron toxicity because the evidence of this is fairly indirect. At points, the authors do admit these limitations, but at other times, they claim to have shown the link directly. The following are reasons why these claims are only indirectly supported:

We agree that we have shown direct toxicity of 2A^pro^ in motor neurons, but have not shown that the mechanism is via NPC dysfunction. We felt that we were careful to frame our conclusions as such. However, we have revised the text to improve the clarity on this point as described above.

(1) Cleavage of Nup98 and POM121 after EV-D68 infection in RD cells and diMNs is never demonstrated.

We have added data showing the cleavage of POM121 and Nup98 in EV-D68 infected diMNs (Figure 4A).

(2) Telaprevir was able to rescue nucleocytoplasmic transport in RD cells at low concentrations (Figure 4A). It is not shown if this correlates with its antiviral effect in RD cells, or could this correlate with inhibition of 2A cleavage of Nup98 or POM121, which is never measured.

In the aforementioned new experiment in Figure 4A, we have also included a dose-response curve for telaprevir showing its inhibition of POM121 and Nup98 cleavage.

(3) Building off of the prior point, the authors' claim that the neuroprotective effect of telaprevir is independent of its antiviral effect is not well-founded. Figure 4E (neuroprotection) was done with MOI 5, and Figure 4G (virus growth) was MOI 0.5. Telaprevir neuroprotection is not shown at MOI 0.5, nor is the neuroprotective effect correlated with inhibition of 2A cleavage of Nup98 or POM121.

The selection of MOIs for these two experiments was limited by technical considerations. If the viral growth curve were to be performed at MOI 5, it would be confounded by cell death. Further, a low MOI is required in order to allow multiple rounds of infection, and is therefore more sensitive for assaying the effect of telaprevir on viral replication. On the other hand, at MOI 0.5 diMN death is very gradual, and the neuroprotection assay we would have lacked the statistical power to determine whether a rescue of this small magnitude of toxicity is significant. The EC_50_ of telaprevir is not expected to vary at different MOIs.

We have also now correlated the inhibition of 2A^pro^ cleavage of Nup98 and POM121 with the neuroprotective effect at comparable concentrations of telaprevir, as described above.

(4) The use of mixed virus isolates only in the diMNs is problematic because different EV-D68 isolates are known to have drastically different effects on pathogenesis in mice. Since all initial data were generated with the MO isolate, adding the additional MD isolate to the diMN experiments actually adds uncertainty to the conclusions. It is not clear if the authors infected different cultures with the different isolates and combined the data or infected all cultures with a mixture of the two isolates. If the former, then the data should be reported separately to see the effect of each individual strain, which would be interesting to EV-D68 virologists. If the latter, then there is no way to know from these data whether one of the two isolates had increased fitness over the other and exerted a dominant effect. If the MD isolate overtook the MO isolate, from which all other data in this manuscript are derived, then we have much less of an idea how much the data from the first three figures supports the final figure.

We apologize for the lack of clarity in describing this experiment. The MO/2014 and MD/2018 isolates were not mixed. These were performed in separate experiments, each with four biologically independent replicates. The original figure showed the mean and SEM for these 8 replicates together. To improve clarity, we separated each viral strain into its own panel of the figure. We have also increased the rigor of the statistical analysis in this experiment by using Cox proportional hazard regression instead of ANOVA.

**Recommendations for the authors:**

**Reviewing Editor Comments:**

Please consider both public reviews above and recommendations for the authors below. The general consensus among reviewers is that more evidence is needed to support the claim that 2A causes motor neuron toxicity during infection.

**Reviewer #1 (Recommendations for the authors):**

Most of the conclusions are made upon analysis of images, yet the images themselves are seldom shown. It is difficult to evaluate the validity of conclusions without seeing the material that was analyzed.

(1) Figure 1. Representative Western blots should be shown.

We considered including representative western blots in this already large figure, however the figure size and complexity became un-manageable because the figure summarizes the quantification of 246 Western blots. In the original submission, we uploaded a supporting data file that included complete un-cropped Western blots for all experiments, including ladders, loading controls, and clear labeling of the samples. We believe these data allow the reader to assess the quality and reliability of our Western blot experiments while maintaining the approachability of the figures and data presentation. We have also included these supporting data again in the revised manuscript.

(2) Figure 3. Representative images should be shown. This is especially important for the ethynyl-uridine labeling experiment. It would be highly surprising that RNA transcription and processing would proceed normally in 2A-expressing cells on the background of a major redistribution of nuclear proteins. One possible explanation for that would be that cells that can be analyzed express a relatively small amount of 2A, which is known to be toxic, and thus may not fully represent the cellular changes upon infection. The results from bona fide infected cells would be much more convincing.

Representative images have been added for the ethynyl-uridine pulse-chase experiment, and this experiment has been repeated in RD cells infected with EV-D68. Transfection of proteases or infection of the cells utilized the same protocols and timeframes upon which nucleoporin cleavage and disruption of protein transport were found to be present. The timepoint for all of these experiments was selected to precede the onset of toxicity, and the representative images demonstrate normal cellular morphology. We also selected for analysis only GFP+ cells with normal morphology, ensuring that only viable 2A^pro^-GFP-expressing cells were included in the analysis. The new experiments again showed no effect on RNA export. We were equally surprised as the reviewers by this outcome. However, as we note in the text, disruption of RNA export has not been uniformly present across all enteroviruses previously studied.

(3) Figure 4 A-D. Similarly, representative images should be shown.

We have added representative images for these experiments, which are now Fig 4B-E.

(4) Figure 4G. The demonstration that the "neuroprotective" effect of 2A inhibitor is not related to the inhibition of viral replication requires a control showing that a similar inhibition of viral replication by an inhibitor with another target would not similarly diminish cell toxicity.

Neuronal survival experiments showed inhibition of toxicity with concentrations of telaprevir as low as 0.3 uM, a concentration at which there was no significant effect on viral replication. Telaprevir had only a marginal inhibitory effect on viral replication at 10uM (achieving statistical significance in only one of two strains), and no consistent effect on replication at lower concentrations. Therefore, the suggested control experiment would not be possible, because the neuroprotective concentration of telaprevir does not inhibit viral replication

**Reviewer #2 (Recommendations for the authors):**

Major concerns:

(1) Most of the experiments were performed with recombinant 2A and 3C proteins. While these experiments are highly informative for dissecting the role of each protease in NPC dysfunction, it would be important to also perform experiments in the context of infection. How are import and export processes affected when both proteases are present during infection? How is passive transport modified under these conditions?

Thank you for this important comment. Please see the above discussion of additional experiments that we added utilizing live virus infection to complement the experiments that used recombinant proteins.

(2) The results regarding RNA export in the presence of recombinant 2A and 3C proteases suggest that RNA export is not altered. It would be important to confirm this finding during infection.

We agree that this is an important experiment, and have done so as described above.

(3) While the background information suggests that NPC dysfunction contributes to neurotoxicity, the observed reduction of neurotoxicity by telaprevir does not demonstrate that this effect is solely due to the action of 2A on the NPC. It would be important to evaluate the integrity of eIF4G, nucleoporins, and stress granules during treatment.

We agree that additional experiments would be required to determine the extent to which the toxicity of 2A^pro^ is mediated through its effects on the NPC versus other potential targets. Please see above discussion for more details.

(4) Including another 2A inhibitor (e.g., boceprevir) would strengthen the conclusions by confirming the results obtained with telaprevir.

Please see above discussion of boceprevir

**Reviewer #3 (Recommendations for the authors):**

(1) Preferred ICTV nomenclature abbreviates rhinovirus as RV instead of HRV, so the authors should change their abbreviations appropriately. See Simmonds et al.

Archives of Virology (2020) 165:793-797 https://doi.org/10.1007/s00705-019-04520-6

We have updated these abbreviations accordingly.

(2) There is no mention of Figures 1C and 1D in the text.

These have been added in the appropriate locations.

(3) In the section "2A protease alters nucleocytoplasmic trafficking of protein substrates" it would be very helpful to just directly state what each construct is meant to demonstrate. Along the lines of "NLS-tdTomato should be located in the nucleus, so seeing more signal in the cytoplasm would indicate a defect in nuclear import." And something equivalent for the other two constructs.

Thank you for the suggestion. We have added descriptions of the use and interpretation for each construct.

(4) The following sentence would be more accurate with the addition of "partially" because the effect is not returned to normal levels: "The mislocalization of NLS-tdTomato was partially rescued by 3μM telaprevir."

We have edited this as recommended.

(5) SNAP29 is probably a typo and meant to be CREB in the legend of Figure 1B.

Thank you for catching this. We have corrected this to CREB.

(6) "Panel A" should likely be "Panel E" in the Figure 4F legend.

We have corrected this to refer to the appropriate panel, which has also been re-lettered due to the addition of new panels to this figure.

(7) The authors should at least show representative Western blot data used to determine the data for Figure 1 in a supplemental figure.

As discussed above, these Western blots were included as supplemental data in the original submission, and have also been included in the revised version.

(8) As suggested in the public comments, if the diMNs were infected separately with the MO and MD strains of EV-D68, those data should be separated from each other and reported individually. In any case, whatever was done (combined virus inoculum or separate inocula) needs to be clarified.

These data are now reported separately. Please see above discussion for details.

References:

(1) Vogt MR, Wright PF, Hickey WF, De Buysscher T, Boyd KL, Crowe JE, Jr. Enterovirus D68 in the Anterior Horn Cells of a Child with Acute Flaccid Myelitis. N Engl J Med. May 26 2022;386(21):2059-2060. doi:10.1056/NEJMc2118155

(2) Hixon AM, Yu G, Leser JS, et al. A mouse model of paralytic myelitis caused by enterovirus D68. PLoS Pathog. Feb 2017;13(2):e1006199. doi:10.1371/journal.ppat.1006199

(3) Andersen EW, Kornberg AJ, Freeman JL, Leventer RJ, Ryan MM. Acute flaccid myelitis in childhood: a retrospective cohort study. Eur J Neurol. Aug 2017;24(8):1077-1083. doi:10.1111/ene.13345

(4) Elrick MJ, Gordon-Lipkin E, Crawford TO, et al. Clinical Subpopulations in a Sample of North American Children Diagnosed With Acute Flaccid Myelitis, 2012-2016. JAMA Pediatr. Feb 1 2018;173(2):134-139. doi:10.1001/jamapediatrics.2018.4890

(5) Hovden IA, Pfeiffer HC. Electrodiagnostic findings in acute flaccid myelitis related to enterovirus D68. Muscle Nerve. Nov 2015;52(5):909-10. doi:10.1002/mus.24738

(6) Knoester M, Helfferich J, Poelman R, et al. Twenty-Nine Cases of Enterovirus-D68 Associated Acute Flaccid Myelitis in Europe 2016; A Case Series and Epidemiologic Overview. Pediatr Infect Dis J. Jan 2018;38(1):16-21. doi:10.1097/INF.0000000000002188

(7) Martin JA, Messacar K, Yang ML, et al. Outcomes of Colorado children with acute flaccid myelitis at 1 year. Neurology. Jul 11 2017;89(2):129-137. doi:10.1212/WNL.0000000000004081

(8) Saltzman EB, Rancy SK, Sneag DB, Feinberg Md JH, Lange DJ, Wolfe SW. Nerve Transfers for Enterovirus D68-Associated Acute Flaccid Myelitis: A Case Series. Pediatr Neurol. Nov 2018;88:25-30. doi:10.1016/j.pediatrneurol.2018.07.018

(9) Van Haren K, Ayscue P, Waubant E, et al. Acute Flaccid Myelitis of Unknown Etiology in California, 2012-2015. JAMA. Dec 22-29 2015;314(24):2663-71. doi:10.1001/jama.2015.17275

(10) Natera-de Benito D, Berciano J, Garcia A, E MdL, Ortez C, Nascimento A. Acute Flaccid Myelitis With Early, Severe Compound Muscle Action Potential Amplitude Reduction: A 3-Year Follow-up of a Child Patient. J Clin Neuromuscul Dis. Dec 2018;20(2):100-101. doi:10.1097/CND.0000000000000217

(11) Rosenfeld AB, Warren AL, Racaniello VR. Neurotropism of Enterovirus D68 Isolates Is Independent of Sialic Acid and Is Not a Recently Acquired Phenotype. Mbio. 2019;doi:10.1128/mBio

(12) Musharrafieh R, Ma C, Zhang J, et al. Validating Enterovirus D68-2A(pro) as an Antiviral Drug Target and the Discovery of Telaprevir as a Potent D68-2A(pro) Inhibitor. J Virol. Jan 23 2019;doi:10.1128/JVI.02221-18
